# Characterization of the nasopharyngeal microbiota in health and during rhinovirus challenge

**DOI:** 10.1186/2049-2618-2-22

**Published:** 2014-06-25

**Authors:** E Kaitlynn Allen, Alex F Koeppel, J Owen Hendley, Stephen D Turner, Birgit Winther, Michèle M Sale

**Affiliations:** 1 Center for Public Health Genomics, University of Virginia, PO Box 800717, Charlottesville, USA; 2 Department of Biochemistry and Molecular Genetics, University of Virginia, Charlottesville, USA; 3 Bioinformatics Core Facility, University of Virginia, Charlottesville, USA; 4 Department of Pediatrics, University of Virginia, Charlottesville, USA; 5 Department of Otolaryngology, University of Virginia, Charlottesville, USA; 6 Department of Medicine, University of Virginia, Charlottesville, USA

**Keywords:** Microbiota, nasopharynx, longitudinal, sequencing, rhinovirus illness

## Abstract

**Background:**

The bacterial communities of the nasopharynx play an important role in upper respiratory tract infections (URTIs). Our study represents the first survey of the nasopharynx during a known, controlled viral challenge. We aimed to gain a better understanding of the composition and dynamics of the nasopharyngeal microbiome during viral infection.

**Methods:**

Rhinovirus illnesses were induced by self-inoculation using the finger to nose or eye natural transmission route in ten otherwise healthy young adults. Nasal lavage fluid samples (NLF) samples were collected at specific time points before, during, and following experimental rhinovirus inoculation. Bacterial DNA from each sample (N = 97 from 10 subjects) was subjected to 16S rRNA sequencing by amplifying the V1-V2 hypervariable region followed by sequencing using the 454-FLX platform.

**Results:**

This survey of the nasopharyngeal microbiota revealed a highly complex microbial ecosystem. Taxonomic composition varied widely between subjects and between time points of the same subject. We also observed significantly higher diversity in not infected individuals compared to infected individuals. Two genera – Neisseria and Propionibacterium – differed significantly between infected and not infected individuals. Certain phyla, including Firmicutes, Actinobacteria, and Proteobacteria, were detected in all samples.

**Conclusions:**

Our results reveal the complex and diverse nature of the nasopharyngeal microbiota in both healthy and viral-challenged adults. Although some phyla were common to all samples, differences in levels of diversity and selected phyla were detected between infected and uninfected participants. Deeper, species-level metagenomic sequencing in a larger sample is warranted.

## Background

The nasopharynx is frequently colonized by both commensal and pathogenic bacteria [1]. Many pathogenic species, including *Streptococcus pneumonia*, *Haemophilus influenza*, *Moraxella catarrahlis, Staphylococcus aureus,* and *Neisseria meningitidis*, exist in the nasopharynx of apparently healthy individuals [1-4]. Ling *et al*. found the nasopharyngeal microbiota to be distinct from other body sites surveyed (saliva, dominant hands, and feces) in healthy Chinese adults [5].

Understanding the relationships between microbes of the upper respiratory tract (URT) during perturbations is anticipated to provide insights into the pathogenesis of URT infections. Studies of the nasopharyngeal microbiota in children have observed changes due to season (winter/fall versus spring) [4] and treatment with antimicrobials or the heptavalent conjugated pneumococcal polysaccharide vaccine [6,7]. Specific commensal taxa have been negatively associated with colonization of known pathogenic bacteria and with acute otitis media in children, and these relationships changed depending on antibiotic usage [8].

Like pathogenic bacteria, viruses, including rhinoviruses, enteroviruses, coronaviruses, and adenoviruses, have been found in asymptomatic, healthy individuals [9,10]. Infections of the URT display complex interactions between bacterial pathogens and viruses, both synergistic and competitive [11-14]. Viruses in the URT can alter bacterial adherence [15], bacterial colonization [16], and host immune response [17-19]. A study of the nasopharyngeal microbiota of children with severe bronchitis showed microbiotal shifts depending on the viral infection (human rhinovirus (HRV) only, respiratory syncytial virus (RSV) only, or co-infection) [20]. The lung microbiota in chronic obstructive pulmonary disease (COPD) patients and healthy individuals during a rhinovirus challenge showed significant changes after infection [21].

We previously used microarray technology to investigate bacteria in the adult nasopharynx prior to, during, and after experimental rhinovirus infection [22]. The microarray approach allowed us to detect bacteria at the species level, but we were only able to test the presence or absence of about 60 clinically informative bacteria. The goal of the present study was to characterize the relative abundance of microbial communities of the nasopharynx at the phylum and genus levels, by sequencing the 16S rRNA gene of bacteria in nasal lavage fluid (NLF) samples from the same adults during rhinovirus challenge.

## Methods

### Sample collection

**Figure 1:**
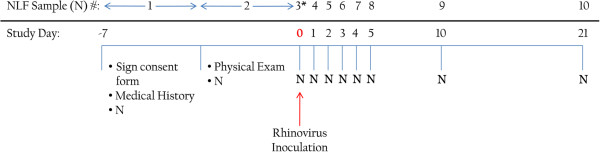
**Study timeline including when information or samples were collected from study participants.** *Day on which nasal lavage fluid (NLF) samples were obtained prior to rhinovirus inoculation.

This protocol has been previously described [22]. Briefly, participants were volunteers between the ages of 18 and 65 years, who responded to advertisements in 2010. Subjects were eligible if they had a screening serum neutralizing antibody of 1:4 or less to the challenge Rhinovirus type 39. The study was approved by the Institutional Review Board for Health Sciences Research at the University of Virginia. Informed consent was obtained from all ten participants prior to enrollment. The rhinovirus immunotype 39 inoculum pool has been safety tested and approved for use by the Food and Drug Administration (FDA) (IND12934). This study was conducted with all sample collection during the fall. Subjects were exposed to a total of 100 to 300 TCID50 of rhinovirus in 250 μL by self-inoculation by touching the medial cantus and conjunctiva of one eye or the septum in the nasal vestibulum on one side of the nose and repeated once after a 5- to 15-minute period. NLF was obtained by installation of 5 mL of 0.9% saline into each nasal cavity which was recovered into a waxed paper cup. The saline is able to reach throughout the nasopharynx due to the position of the participant’s head (tilted backwards). Ten nasal washes were obtained from each volunteer: three during the week prior to HRV inoculation, one on each of five days immediately following inoculation, and on days 10 and 21 following inoculation (Figure 1). An aliquot of 1 mL of each of the NLF sample was placed in tubes containing an equal volume of viral collection broth, kept on ice, and transported to the laboratory within one hour for rhinovirus isolation in tissue cultures. The remaining NLF was transferred to 2-mL cryo-tubes and stored frozen at -80°C.

### Assessment of infection

This protocol has previously been described [22]. Briefly, a subject was considered *infected* if the challenge rhinovirus was detected in the NLF at least once during the five days following inoculation. Additionally, the serum antibody response to the challenge rhinovirus type 39 was examined in sera obtained prior to inoculation and three weeks following inoculation by standard methods [23]. Subjects with a 4-fold increase in antibody titer to the challenge virus were considered to be infected. If either of these tests were positive, the subject was considered infected. Assessment of illness of the participants is described in Additional file 1.

### DNA extraction

DNA was isolated from NLF using bead beating and phenol-chloroform extraction methods. This protocol [24] was optimized from Ren T, *et al*. to increase DNA output from a more difficult sample type (NLF). Briefly, 1.8 mL of nasal wash was transferred to a 2-mL BeadBeater tube (BioSpec, Bartlesville, OK) and centrifuged at 2,348 *g* (5,000 rpm) for 10 minutes. Supernatant (1.3 mL) was aspirated, leaving all pelleted bacterial cells plus 500 μL supernatant for DNA extraction and subsequent sequencing. Then, 20 μL Proteinase K (20 mg/mL) (Roche, Basel, Switzerland) was added and incubated at 60˚C for two hours then 95˚C for 10 minutes. A total of 200 μL 10% SDS (Fischer Scientific, Waltham, MA), 400 μL of 0.1 mm zirconia/silica beads (BioSpec, Bartlesville, OK), 100 μL of buffer (10 mM Tris-HCl pH 7.5, 5 mM EDTA, 100 mM NaCl), and 500 μL of phenol:chloroform:isoamyl alcohol (25:24:1) (Fischer Scientific, Waltham, MA) were added to the tube. The sample was homogenized on a Biospec Mini-BeadBeater-8 for 3 minutes and then centrifuged for 10 minutes at 10,000 *g*. The top aqueous layer was transferred to a new Phase-Lock-Gel tube (VWR, Radnor, PA) with an equal volume of chloroform:isoamyl alcohol (24:1) (Acros Organics, Fischer Scientific, Waltham, MA) and centrifuged at 20,000 *g* for 10 minutes. This step was repeated twice to ensure removal of phenol. The sample was then ethanol precipitated, and the DNA pellet was resuspended in 50 μL 10 mM Tris-HCl pH 8.0. Each set of extractions was accompanied by a negative control to ensure no contaminants in the extracted DNA.

### Tag-PCR amplification of the V1-V2 regions of the bacterial 16S rRNA gene and pyrosequencing

This protocol has been previously described [24]. Briefly, the V1-V2 hypervariable regions of the 16S rRNA gene were amplified from extracted DNA samples using two primers containing the universal sequences 27 F (5′-AGRGTTTGATCMTGGCTCAG-3′) and 534R (5′-TTACCGCGGCTGCTGGCAC-3′). Each sample was tagged with a unique barcode (10 bp) added to the 5′ end of the forward primer. Conditions for amplification were 94˚C for 3 minutes, then 30 cycles of 94˚C for 30 seconds, 57˚C for 45 seconds, and 72˚C for 60 seconds with a final extension step of 72˚C for 5 minutes. Each set of samples was amplified with a negative control to ensure no contaminants from reagents. PCR products were then run on a gel to verify amplification of the correct size. Amplicons from all samples were quantified using the Qubit® 2.0 Fluorometer and then pooled in equal molar ratios with 49 samples in pool 1 and 48 samples in pool 2. To achieve a final pool of 20 μL of sample with a 10 ng/μL concentration, 40.8 ng of each sample were added to pool 1 and 41.67 ng were added from each sample to pool 2. The pooled samples were then gel-purified and the concentration of each sample was measured using the Qubit 2.0 Fluorometer to ensure the minimum of 20 μL of 10 ng/μL sample for sequencing. Each pooled sample was sequenced using titanium chemistry on a 454 Life Science Genome Sequencer FLX platform at the University of Virginia Department of Biology Genome Core Facility using the standard protocol for sequencing. The samples were run on an Agilent 2100 Bioanalyzer High-Sensitivity chip to determine the size distribution of the library samples. Then, a KAPA Biosystems qPCR assay was run to determine the effective concentration of the samples.

### Quality assessment and filtering of sequences

This quality of the read sequences was assessed using FastQC v0.10.1 [25]. This assessment revealed that a significant fraction of our reads were identical short (<100 bp) reads (homopolymers). Based on this assessment, and the distribution of read lengths, we filtered the sequences by length, removing any reads shorter than 100 bp in length, or greater than 600 bp. This filter alone reduced our initial dataset from 4.32 Mbp, to 3.32 Mbp (approximately 23.1% reduction). In addition, reads with an average quality score lower than 25 (phred scale) were removed, as were any reads that contained 50-base windows with average quality scores below that threshold. Qiime’s default filters also removed any reads with mismatches in the primer or barcode sequences, and any reads with more than six ambiguous bases.

### Clustering and filtering of operational taxonomic units (OTUs)

Sequences were clustered into operational taxonomic units (OTUs) (97% identity cutoff) using the uclust algorithm v1.2.22 (*de novo* clustering) [26] in QIIME v1.8 [27], and assigned taxonomy using RDP classifier v2.2 [9], trained with the Greengenes (gg_13_8) [28] dataset. OTUs were removed from the dataset if they could not be classified at the domain level, or were classified as archaea, or were classified as chloroplast sequences. OTUs estimated to be putatively chimeric by the uchime algorithm v4.2.40 [29] were also filtered out, as were singleton OTUs. After filtering, we were left with a mean of 1021.6 sequences per sample (SD 630.84, median 913, minimum 134, maximum 4,501). Samples containing fewer sequences than three SD below the mean count (146 sequences/sample) (n = 1) were eliminated from downstream analyses.

### Sequence analysis

Analyses of taxonomic composition and diversity were performed using QIIME, version 1.8 [27]. Default parameters were used for the core analyses. OTU representatives were aligned with pyNAST v1.2.2 [30], and phylogenies were generated using FastTree v2.1.3 [31]. Alpha diversity (within-population diversity) of samples was measured using counts of observed species (OTUs), the chao1 estimator for species richness, and the Shannon diversity index, which estimates total diversity taking into account both species richness and evenness. Beta diversity (diversity between populations) was calculated using UniFrac distances (unweighted) [32] between samples (based on the relative abundance of OTUs), and visualized using principal coordinates analysis (PCoA) [33]. In order to compare diversity of OTUs in the nasopharynx to that of other body sites, we also performed a combined beta diversity analysis of our nasopharyngeal samples with samples from several different body sites (gut, oral cavity, external auditory canal, nostril, and hair) from Costello *et al*. [34], as well as adenoid samples from Ren *et al*. [24].

### Statistical analysis

Analyses of phylum abundance between inoculation states (before, during, and after) and between subjects, was performed using analysis of variance (ANOVA). For significant results, the Tukey Kramer test was subsequently conducted. ANOVA was also used for the analyses of class, order, and family abundances between inoculation states (before, during, and after). Comparisons of alpha diversity between the infected and non-infected sample groups were done using Mann-Whitney Wilcoxon tests, as were the abundances of selected genera between the infected and non-infected samples, and the comparison of inter-individual and intra-individual Unifrac distances.

## Results

### Subject population

Ten volunteers were eligible and enrolled into the study. Forty percent of volunteers were female, and the average age of volunteers was 19.6 years. Six of the subjects were self-inoculated into the nose and four were self-inoculated into the eye. Seven out of the ten subjects were infected by the rhinovirus challenge. Out of the infected subjects, three were considered ill based on the modified Jackson cold method (Additional file 1). All subjects were sampled at all ten time points. Demographic information and information on infection status is included in Table 1.

**Table 1:** Subject information

	**Subject number**									
**Variable**	**1**	**2**	**3**	**4**	**5**	**6**	**7**	**8**	**9**	**10**
										
**Age, years**	19	19	18	18	20	21	21	20	19	21
**Sex**	F	F	F	M	F	M	M	M	M	M
**Race**	White^1^	White	White	White	White, Asian	White	White	White	African American	White
**Route of inoculation**	Eye	Nose	Nose	Nose	Nose	Eye	Eye	Eye	Nose	Nose
**HRV infection**	yes	no	yes	yes	yes	no	yes	yes	no	yes
**Criteria for infection status**	Ab	-	HRV/Ab	HRV	HRV/Ab	-	HRV/Ab	Ab	-	HRV/Ab

^1^The subject did not specify “Non-hispanic.” For criteria for infection status, HRV indicates HRV shedding in at least one sample during time points 4 to 8 (during the infection period) and Ab indicates that the subject had a 4-fold rise in serum-neutralizing antibody in response to the challenge virus. M, male; F, female.

**Figure 2:**
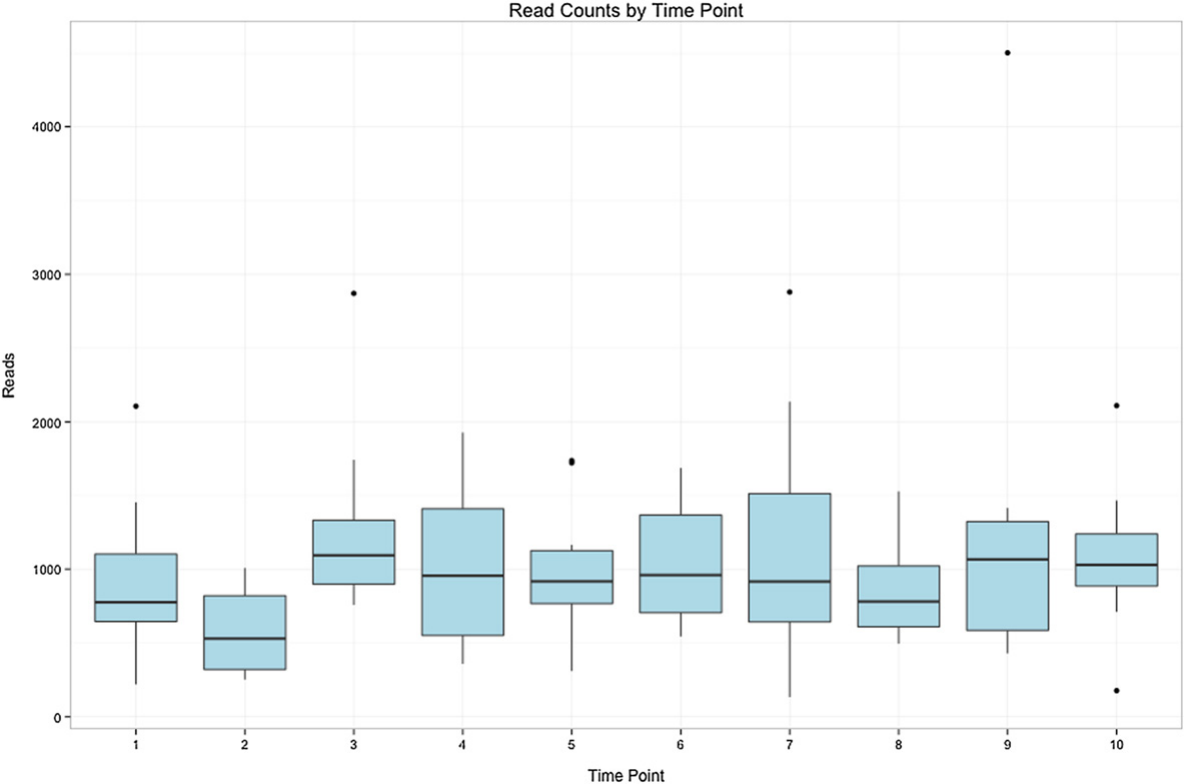
Box and whisker plot of read counts at each time point during the study after quality control filtering.

DNA was successfully extracted from 99/100 NLF samples with concentration values ranging from 5.86 ng/μL to 33.8 ng/μL, which is above the recommended threshold for microbiota studies [35]. One sample (subject number 6, day 3) did not have enough NLF for DNA extraction. PCR amplification and pyrosequencing of the V1-V2 hypervariable regions of the bacterial 16S rRNA gene was successful in 97 out of the 99 NLF samples. PCR amplification was unsuccessful in two samples (subject number 3, day 2 and subject number 10, day 3). Sequencing of the two pools of DNA extracted from the NLF samples resulted in 679,135 reads. These reads were then quality filtered to select the most reliable reads for analysis (see Methods). After all quality filters had been performed on both raw sequences and OTUs (see Methods), there were 99,095 reads remaining for analysis (median of 913 reads per sample) (Figure 2). After filtering, reads had a mean length of 376 bp (median 414 bp), and one sample (subject number 10, day 7 (134 reads)) was excluded for containing fewer sequences than 3 SD below the mean count (146 sequences/sample).

### Analysis of nasopharyngeal bacterial richness and composition by sequencing

**Figure 3:**
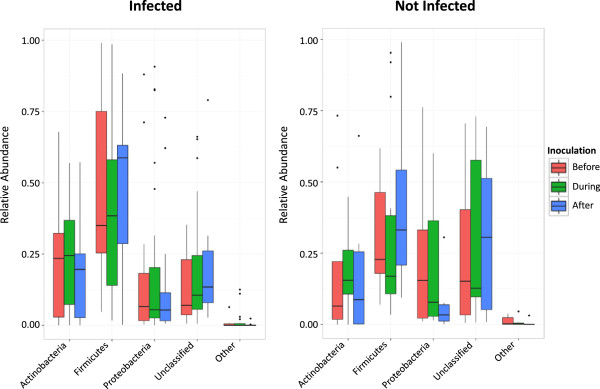
**Box and whisker plot of the relative abundance of dominant phyla in infected and not infected individuals by time period.** Sample counts for Infected samples: (before (n = 19), during (n = 34), and after (n = 14)). Sample counts for Non-Infected samples: (before (n = 8), during (n = 15), and after (n = 6)).

The sequences were clustered into 3,229 distinct OTUs (97% identity cutoff). Singleton OTUs were filtered out, leaving 1,608 OTUs for downstream analysis. Across all ten subjects, the dominant phyla were Firmicutes (40.6%), Actinobacteria (20.9%), Proteobacteria (17.1%), and unclassified (20.6%), with the relative abundance of each phylum being highly variable across the subjects (Additional files 2 and 3) and time points (Additional files 2 and 4). To determine if bacterial composition changes occur due to viral infection, we compared the relative abundance of the dominant phyla before (time points 1 to 3), during (time points 4 to 8), and after (time points 9 to 10) Rhinovirus infection in infected and not infection subjects (Figure 3). No phylum showed a significant change in relative abundance between time points. Additionally, we performed ANOVA on the relative abundances (log-transformed values), which revealed no significant differences (after FDR correction) in the abundance of the three dominant phyla before, during, or after inoculation, in either the infected or uninfected categories. Significant differences were found in two subjects: subject number 5, in which Proteobacteria abundance was significantly different before inoculation than during or after inoculation (*P* = 0.039), and subject number 9, in which Actinobacteria abundance was significantly different between the before, during, and after time points (*P* = 0.0001) (Additional file 5). No significant differences were found in the abundances of dominant classes, orders, or families before, during, or after inoculation, in either infected or not infected individuals (all *P*-values >0.11). ANOVA was also performed to test the null hypothesis that the relative abundances (log-transformed values) of the dominant phyla were the same between subjects (Additional file 6). After FDR correction, there was a significant difference in the relative abundance of Firmicutes between subjects (*P*_adj_ = 0.009 in not infected subjects, *P*_adj_ = 0.001 in infected subjects).

**Figure 4:**
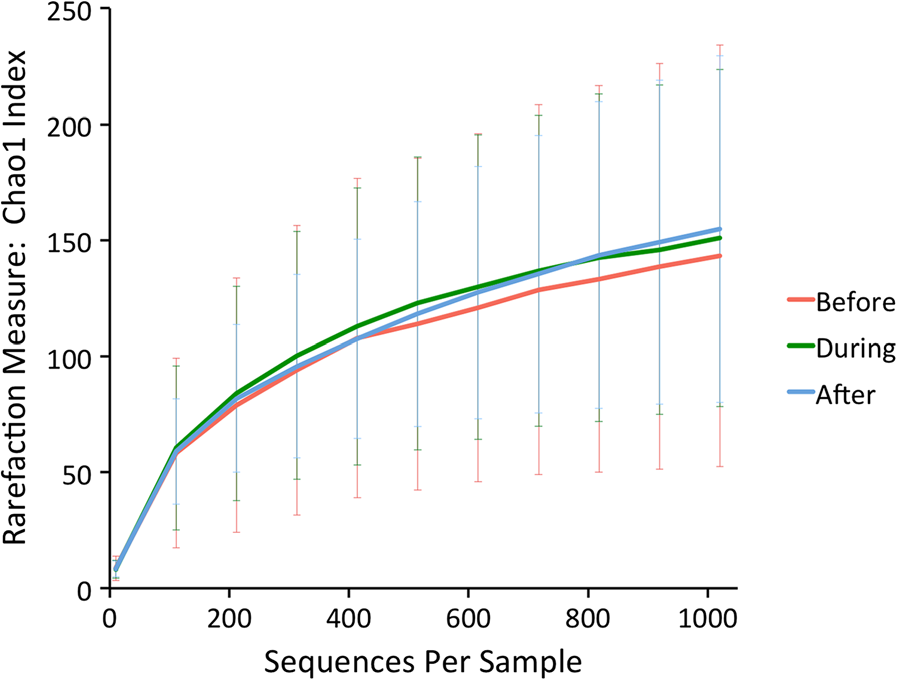
Rarefaction plots displaying the relationship between the sample size and the number of observed species.

We also carried out a rarefaction analysis to compare bacterial species richness between the samples before, during, and after infection by plotting the rarefaction measure (the Chao1 diversity index for OTUs at 97% identity threshold) by the number of sequences per sample (Figure 4; Additional files 7 and 8). The rarefaction plot curves indicate that the nasopharynx, sampled by NLF, is a very complex microbial environment. Deeper sequencing could reveal even more diversity in this very complex microbiota. Additionally, there is no significant difference between the species richness between the three time points, though there is a trend for more bacterial richness in the time points during and after inoculation than in the time points before inoculation.

The most abundant genus in the nasopharyngeal microbiota across samples was *Alloiococcus* (21.2% of total abundance), followed by *Corynebacterium* (14.7%), *Staphylococcus* (8.9%), *Haemophilus* (5.3%), *Propionibacterium* (4.6%), and *Streptococcus* (3.2%). All other classified OTUs belonged to genera comprising less than 2% of the total abundance. It should be noted that these abundances reflect total abundance across all samples. Taxonomic composition varied widely between samples (Additional file 2).

**Figure 5:**
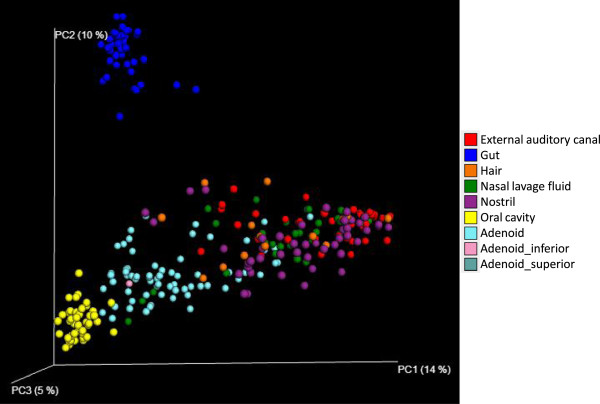
**Nasopharyngeal microbiota is similar to neighboring body parts, but distinct from gut and oral cavity.** Principal coordinates analysis (PCoA) was performed using the unweighted UniFrac distance matrix using nasal lavage fluid (NLF) samples from pre-inoculation time points. Each sample is represented by a point with NLF (n = 27) in green, gut (n = 45) in dark blue, oral cavity (n = 46) in yellow, external auditory canal (n = 44) in red, nostril (n = 46) in purple, hair (n = 14) in orange, and adenoids (n = 69) in light blue.

**Figure 6:**
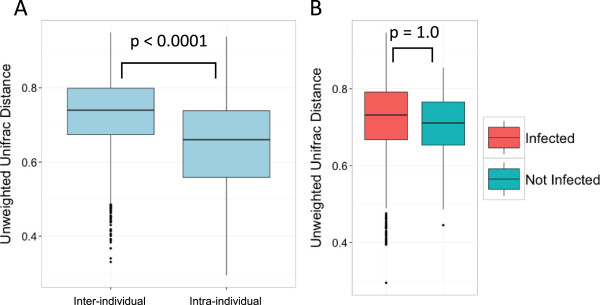
**Unifrac distance comparisons. (A)** Unweighted Unifrac distances between samples from different individuals (inter-individual) and the distances between samples from the same individual (intra-individual**). (B)** Inter-individual unweighted Unifrac distances compared between infected and not infected subjects**.***P*-values displayed are the results of Mann-Whitney Wilcoxon tests.

We compared the microbiota of the nasopharynx (NLF samples) to those of other body sites including the gut, oral cavity, external auditory canal, nostril, hair, and adenoid using PCoA (Figure 5; Additional files 9 and 10). For the purpose of comparison, only the NLF samples taken prior to inoculation were included for this analysis. The microbiota of the nasopharynx is most similar to the external auditory canal, nostril, hair, and adenoid. The gut and oral cavity are distinct from the rest of the samples. When we used PCoA to compare the NLF samples before, during, and after infection; infected versus not infected; ill versus not ill; and by route of infection, we saw no distinct differences in the samples (Additional files 11 and 12). When looking at the PCoA to compare NLF samples between each subject (Additional file 11), there was no distinct clustering by subject. When comparing NLF samples within each subject (Additional file 12), there was also no distinct clustering. Unifrac distances between samples from different individuals was significantly greater than the distances between samples from the same individual (Figure 6A). Inter-individual Unifrac distances were not significantly different between infected and not infected subjects (Figure 6B).

### Bacterial community structure of infected and not infected subjects

**Figure 7:**
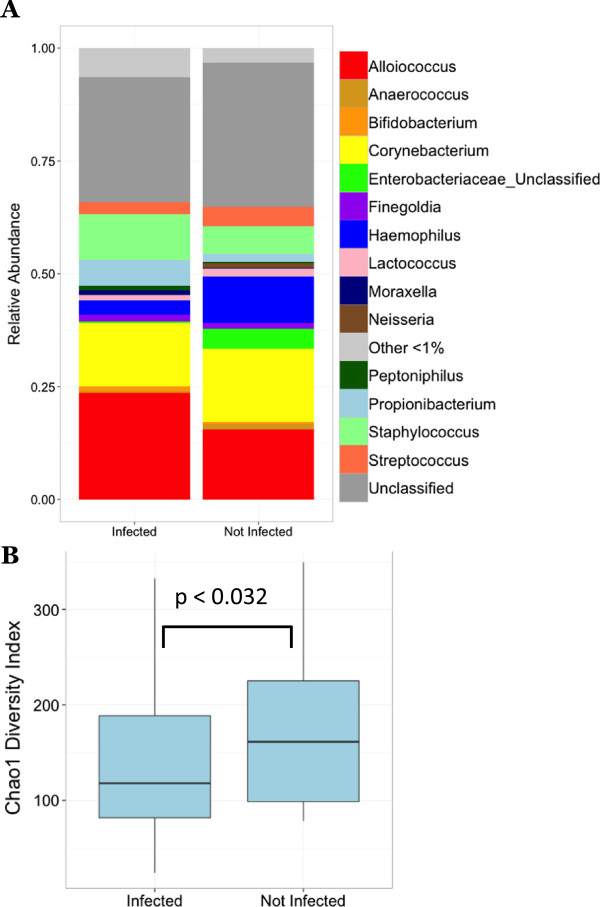
**Stacked taxonomic bar chart (A) with box and whisker plot (B) - Shannon diversity index (Not infected (n = 29) versus Infected (n = 67).** Operational taxonomic units (OTUs) were categorized as “Unclassified”, if RDP classifier could not classify them to at least the family level. Genera that could be classified to the family level, but not the genus level were categorized by the family name, followed by “_Unclassified” to indicate that the genus is not known. All genera comprising less than 1% of the total abundance (whether classified or not) were combined into the “Other <1%” category.

The community structure of bacteria in the NLF samples of infected (n = 7) and not infected (n = 3) subjects were compared at the genus level. Both groups showed similar patterns of genera distribution (Figure 7A), but there were significant differences (*P* = 0.031) in the overall community diversity (Chao1 index) between infected and not infected subjects (Figure 7B). The Mann-Whitney Wilcoxon test was used to compare the relative abundances between infected and not infected samples of all genera comprising 1% or more of the total abundance across samples. Significant differences were detected in two genera (*Neisseria* and *Propionibacterium*) after using the Benjamini and Hochberg false discovery rate correction for multiple comparisons (Table 2). Prior to correction for multiple comparisons, the genus *Haemophilus* was significantly less abundant in the infected samples, suggesting a potential association that could be further investigated with additional samples.

**Table 2:** Comparisons of genus abundance between infected and uninfected samples, using the Mann-Whintey Wilcoxon test

	**Mean Relative Abundance**				
**Genus**	**Infected**	**Not infected**	**W**	***P*-value**	***P*-adj**
*Alloiococcus*	0.237	0.155	1198	0.071	0.227
*Anaerococcus*	0.004	0.013	801	0.137	0.274
*Bifidobacterium*	0.010	0.004	975.5	0.969	0.970
*Corynebacterium*	0.141	0.162	946	0.842	0.970
*Enterobacteriaceae*_unclassified	0.003	0.043	864	0.343	0.534
*Finegoldia*	0.015	0.013	1166	0.113	0.263
*Haemophilus*	0.032	0.104	709	**0.020**	0.095
*Lactococcus*	0.013	0.016	898.5	0.227	0.398
*Moraxella*	0.009	0.002	976	0.970	0.970
*Neisseria*	0.001	0.011	667	**0.001**	**0.020**
*Peptoniphilus*	0.009	0.003	1176.5	0.081	0.227
*Propionibacterium*	0.058	0.018	1315.5	**0.006**	**0.042**
*Staphylococcus*	0.101	0.061	964.5	0.959	0.970
*Streptococcaceae*_unclassified	0.027	0.043	871.5	0.417	0.583

*P*-values < =0.05 (with and without false discovery rate correction) are bolded.

## Discussion

This study is the first to survey and describe the microbiota of the nasopharynx in healthy adults during a rhinovirus challenge. We found a highly complex microbiome that varied between individuals, and significant differences in two genera (*Neisseria* and *Propionibacterium*) between infected and not infected individuals. Strengths of our study include a controlled rhinovirus study, longitudinal analyses, and use of high-throughput sequencing technology. Limitations include the modest sample size and sequencing depth.

The results from this survey have been compared to the results of the microarray study [22] using the same NLF samples. Due to the design of the microarray study, it can only detect the presence or absence of bacterial species on the panel included, while the 16S method is able to determine relative abundance of all bacteria in the sample. We can only compare the number of mismatches in those genera that the microarray was able to detect (n = 8). Out of the total 96 samples, the percent of samples which were detected in one study but not the other ranged from 4% to 53% in each genus. These results can be due to two aliquots from the same NLF sample extracted using two different methods. It could also be due to the microarray detecting species in very low abundance because of enrichment due to PCR.

Due to the level of complexity of the nasopharyngeal microbiota, it is possible that significant changes in the bacterial community before, during, and after infection could likely be detected with deeper sequencing and/or recruiting more subjects. Some large changes in relative abundances of phyla within individuals at close time points were observed. It will be important to investigate whether these reflect true biological fluctuations in this dynamic ecological niche or low microbial density via greater sequencing depth. Additionally, using true shotgun metagenomics and taxonomic classification [36] to distinguish bacteria at the species level may allow significant changes in pathogenic and opportunistic bacteria during infection to be discovered. Information on the species level is especially important because, in numerous cases, there are pathogenic and commensal bacteria within the same genus, for example, *Haemophilius influenzae*, a known pathogen, and its closest phylogenetic relative *Haemophilus haemolyticus*, a commensal bacterium [37,38].

PCoA of the nasopharyngeal microbiota samples along with samples from various other body habitats shows logical clustering with similar locations throughout the URT. The nasopharynx, adenoid, ear, hair, and nostril samples all formed a single large cluster. While this cluster was distinct from the separate clusters formed by the gut and oral samples, there was not a clear differentiation between the other body sites. Bacterial niches in close proximity have some sharing of bacteria due to physiological events like sneezing and nose blowing [39-41], which could help to explain the overlap between the microbiota of the nasopharynx and other proximal body habitats. Differences in age and health status of subjects along with season of sample collection may impact results. Participants in the Costello, *et al*. study included healthy adults, with two samples collected during June and two during the following September [34]. Adenoids in the Ren, *et al*. study were from children undergoing adenoidectomy, collected year round [24]. Demographic data was not accessible because these were discarded surgical specimens. NLF samples in our study were collected during the fall season in healthy adults. Future studies will need to sample complementary niches in the URT to understand coordinated changes of bacterial communities in response to viral infection.

In our study, we surveyed the microbiota of the healthy nasopharynx during viral infection and cataloged a highly diverse population of microbes. The nine dominant phyla of the adenoid microbiota [24] contained the dominant phyla found in our study including Firmicutes, Proteobacteria, and Actinobacteria. We found the same dominant phyla as a study of nasopharyngeal microbiota of healthy Chinese adults [5]. We did not observe an effect of viral infection on microbial composition, which is likely due to a small population, insufficient sequencing depth to detect the full complexity of the microbiota, and the inability to classify OTUs to the species level. In addition to deeper sequencing to achieve species level information, shotgun metatranscriptomics could be used as a complementary approach, to characterize the changes in gene expression that the invasion of a virus can elicit in a microbial community [42]. This information would help us to address the open question of the functional role that the microbiota plays in viral infections. It is uncertain, for instance, whether the microbiota can help to protect the host from viral infection by providing a barrier or by initiating an immune response. Alternatively, it is also possible that the microbiota can aid a virus in invading the host mucosa. One metagenomic study of the airway DNA virome of cystic fibrosis (CF) and non-CF individuals showed that the metabolic profiles of CF and non-CF individuals were distinctly different [43]. This study used the approach of finding target pathways that have altered expression levels in the diseased state, rather than taxonomic composition differences of the microbiota to discover novel therapeutic strategies. Our study has highlighted the highly diverse microbial composition of the adult nasopharynx in both healthy individuals and during viral infection.

Our finding that each individual had a unique nasopharyngeal microbiota broadened our views on how to study and treat URTIs. Most prior studies have looked at a population of individuals with a specific URTI or complication (*that is,* sinusitis) and sampled each individual for specific pathogens. From the findings of such studies, conclusions may be drawn as to which pathogens are likely involved and targeted treatments may be created. However, with a new understanding of the complexity of the NP microbiota at the individual level, we can apply this understanding by recognizing that future studies need to sample patients with URTIs or complications at baseline comprehensively, and then during acute disease to determine which bacteria respond, expand, disappear. This approach is anticipated to lead to improved understanding of disease and more tailored approaches to treatments of these diseases.

## Conclusions

In conclusion, we have conducted a longitudinal survey of the nasopharyngeal microbiota of healthy adults during rhinovirus challenge. This microbiota is highly diverse and varies greatly between individuals. The microbiota of each individual differs between time points throughout the viral challenge, though variability within individuals was less than between individuals. No significant changes in bacterial presence or relative abundance between time points were found due to viral infection. Further studies are needed to further characterize this microbiota and the response of the microbiota to viral infection down to the species level using deeper sequencing. Additionally, metagenomic sequencing will give us insight into transcriptional changes of the host and microbiota during viral infection. With this knowledge, we will better understand the pathogenesis of URT infections.

## Availability of supporting data

The additional files and data set supporting the results of this article are available in Figshare, http://dx.doi.org/10.6084/m9.figshare.832471.

## Abbreviations

ANOVA: analysis of variance; Bp: base pairs; CF: cystic fibrosis; COPD: chronic obstructive pulmonary disease; NLF: nasal lavage fluid - the sample type used to comprehensively survey the microbiota of the nasopharynx; OTU: operational taxonomic unit - approximations of bacterial species based on a percent similarity threshold for DNA sequence identity at a specified locus (usually 16S rRNA). PCoA: principal coordinates analysis - method used to visualize beta diversity between samples using UniFrac distances; URT: upper respiratory tract - URT refers to the parts of the respiratory tract including the nasal cavity the pharynx, and the larynx; URTI: upper respiratory tract infection - general term used to refer to acute infections of the URT which are usually caused by viral infection.

## Competing interests

The authors declare that they have no competing interests.

## Authors’ contributions

EKA carried out molecular genetic studies (DNA extraction, PCR amplification, sequencing preparation), participated in data analysis, and drafted the manuscript. AK carried out data analysis, carried out statistical analyses, and helped in drafting the manuscript. JOH participated in study design and sample recruitment. SDT participated in data analysis and statistical analyses. BW conceived of the viral challenge study, coordinated study recruitment, and carried out sample processing and storage. MMS conceived of the microbiota study and coordinated molecular genetic studies. All authors read and approved the final manuscript.

## Supplementary Material

Additional file 1 **Word document.** Assessment of illness. This document describes how subjects were assessed for clinical illness. This information was only used for Beta diversity analyses (Illness; Additional files 11 and 12). Click here for file

Additional file 2 **Qiime taxonomic composition results (compressed).** Taxa summary plots for all samples. Bar and area charts describing the taxonomic composition of all samples analyzed at all taxonomic ranks from genus to phylum. Click here for file

Additional file 3 **Qiime taxonomic composition results (compressed).** Taxa summary plots organized by subject. Bar and area charts describing the taxonomic composition of all samples analyzed at all taxonomic ranks from genus to phylum, organized by subject. Click here for file

Additional file 4 **Qiime taxonomic composition results (compressed).** Taxa summary plots organized by time point. Bar and area charts describing the taxonomic composition of all samples analyzed at all taxonomic ranks from genus to phylum, organized by time point. Click here for file

Additional file 5 **Excel file.** Analysis of variance (ANOVA) test of dominant phyla in each of the subjects. Results for ANOVA test on the relative abundances (log-transformed values) of the three dominant phyla before, during, or after inoculation, in either the infected or uninfected categories. Click here for file

Additional file 6 **Excel file.** Analysis of variance (ANOVA) test of dominant phyla between subjects. ANOVA to test the null hypothesis that the relative abundances (log transformed values) of the dominant phyla were the same between subjects in infected and not infected groups. Click here for file

Additional file 7 **Compressed file.** Alpha diversity_150. Alpha diversity analyses including rarefaction plots. Plots go to minimum sequences/sample (approximately 150). Click here for file

Additional file 8 **Compressed file.** Alpha diversity_1000. Alpha diversity analyses including rarefaction plots. Plots exclude samples with fewer than the mean sequences/sample (approximately 1000). Click here for file

Additional file 9 **Compressed file.** Beta Diversity_All body sites_pre-inoculation nasal lavage fluid (NLF). Beta diversity (both weighted and unweighted Unifrac) analyses/emperor plots for all body sites, including pre-inoculation nasal wash samples. Click here for file

Additional file 10 **Compressed file.** Beta Diversity_All body sites_all nasal lavage fluid (NLF). Beta diversity (both weighted and unweighted Unifrac) analyses/emperor plots for all body sites, including all nasal wash samples. Click here for file

Additional file 11 **Compressed file.** Beta Diversity_ nasal lavage fluid (NLF) samples only. Beta diversity (both weighted and unweighted Unifrac) analyses/emperor plots for the nasal wash data only. Click here for file

Additional file 12 **Compressed file.** Beta Diversity_ nasal lavage fluid (NLF) samples only_by subject. Beta diversity (both weighted and unweighted Unifrac) analyses/emperor plots for the nasal wash data only, split up by subject. Click here for file
