# Supplementary material for: Characterization of the nasopharyngeal microbiota in health and during rhinovirus challenge
Source: Microbiome. 2014 Jun 25;2:22. doi: 10.1186/2049-2618-2-22 (PMC4098959; doi:10.1186/2049-2618-2-22)
Supplement: Additional file 1 — Word document. Assessment of illness. This document describes how subjects were assessed for clinical illness. This information was only used for Beta diversity analyses (Illness; Additional files 11 and 12). [file 2049-2618-2-22-S1.docx]

*Assessment of illness*

A subject would be considered to have illness based on the severity of symptoms on days following inoculation. Upper respiratory tract infection symptoms/nasal symptoms included URTI/nasal symptoms included sneezing, runny nose, nasal obstruction, and sore throat. Non-nasal symptoms included malaise, chilliness, cough and headache. Subjects were asked to judge their symptoms on a scale from 0 (absent) to 4 (very severe). The diagnosis of a cold illness (modified Jackson cold [[1](#_ENREF_1)]) required a total symptom score of ≥ 6 for 5 days and either the presence of rhinorrhea on 3 or more days or the subjective impression of having a cold.

*Reference*

1. Jackson GG: **Transmission of the common cold to volunteers under controlled conditions. I. The common cold as a clinical entity.** *Archives of Internal Medicine* 1958, **101:**267.
