# Supplementary material for: Characterization of the nasopharyngeal microbiota in health and during rhinovirus challenge
Source: Microbiome. 2014 Jun 25;2:22. doi: 10.1186/2049-2618-2-22 (PMC4098959; doi:10.1186/2049-2618-2-22)
Supplement: Additional file 2 — Qiime taxonomic composition results (compressed). Taxa summary plots for all samples. Bar and area charts describing the taxonomic composition of all samples analyzed at all taxonomic ranks from genus to phylum. [file 2049-2618-2-22-S2.zip › 8440692981118328_add2.tgz!/Tax_composition_less1/taxa_summary_plots/charts/Ai3KOSJ4KG8IA8AB8fHcruTG1A1m03_legend.pdf]

Unclassified;Other;Other;Other;Other
k__Bacteria;Other;Other;Other;Other
k__Bacteria;p__Actinobacteria;Other;Other;Other
k__Bacteria;p__Actinobacteria;c__Actinobacteria;Other;Other
k__Bacteria;p__Actinobacteria;c__Actinobacteria;o__Actinomycetales;Other
k__Bacteria;p__Actinobacteria;c__Actinobacteria;o__Actinomycetales;f__Actinomycetaceae
k__Bacteria;p__Actinobacteria;c__Actinobacteria;o__Actinomycetales;f__Corynebacteriaceae
k__Bacteria;p__Actinobacteria;c__Actinobacteria;o__Actinomycetales;f__Dermabacteraceae
k__Bacteria;p__Actinobacteria;c__Actinobacteria;o__Actinomycetales;f__Dietziaceae
k__Bacteria;p__Actinobacteria;c__Actinobacteria;o__Actinomycetales;f__Geodermatophilaceae
k__Bacteria;p__Actinobacteria;c__Actinobacteria;o__Actinomycetales;f__Microbacteriaceae
k__Bacteria;p__Actinobacteria;c__Actinobacteria;o__Actinomycetales;f__Micrococcaceae
k__Bacteria;p__Actinobacteria;c__Actinobacteria;o__Actinomycetales;f__Nocardioidaceae
k__Bacteria;p__Actinobacteria;c__Actinobacteria;o__Actinomycetales;f__Propionibacteriaceae
k__Bacteria;p__Actinobacteria;c__Actinobacteria;o__Bifidobacteriales;f__Bifidobacteriaceae
k__Bacteria;p__Actinobacteria;c__Coriobacteriia;o__Coriobacteriales;f__Coriobacteriaceae
k__Bacteria;p__Bacteroidetes;Other;Other;Other
k__Bacteria;p__Bacteroidetes;c__Bacteroidia;o__Bacteroidales;Other
k__Bacteria;p__Bacteroidetes;c__Bacteroidia;o__Bacteroidales;f__Porphyromonadaceae
k__Bacteria;p__Bacteroidetes;c__Bacteroidia;o__Bacteroidales;f__Prevotellaceae
k__Bacteria;p__Bacteroidetes;c__Bacteroidia;o__Bacteroidales;f__[Paraprevotellaceae]
k__Bacteria;p__Bacteroidetes;c__Cytophagia;o__Cytophagales;Other
k__Bacteria;p__Bacteroidetes;c__Cytophagia;o__Cytophagales;f__Cytophagaceae
k__Bacteria;p__Bacteroidetes;c__Flavobacteriia;o__Flavobacteriales;f__[Weeksellaceae]
k__Bacteria;p__Cyanobacteria;Other;Other;Other
k__Bacteria;p__Cyanobacteria;c__4C0d-2;o__YS2;f__
k__Bacteria;p__Cyanobacteria;c__Synechococcophycideae;o__Synechococcales;f__Synechococcaceae
k__Bacteria;p__Firmicutes;Other;Other;Other
k__Bacteria;p__Firmicutes;c__Bacilli;Other;Other
k__Bacteria;p__Firmicutes;c__Bacilli;o__Bacillales;Other
k__Bacteria;p__Firmicutes;c__Bacilli;o__Bacillales;f__Bacillaceae
k__Bacteria;p__Firmicutes;c__Bacilli;o__Bacillales;f__Paenibacillaceae
k__Bacteria;p__Firmicutes;c__Bacilli;o__Bacillales;f__Planococcaceae
k__Bacteria;p__Firmicutes;c__Bacilli;o__Bacillales;f__Staphylococcaceae
k__Bacteria;p__Firmicutes;c__Bacilli;o__Gemellales;f__
k__Bacteria;p__Firmicutes;c__Bacilli;o__Gemellales;f__Gemellaceae
k__Bacteria;p__Firmicutes;c__Bacilli;o__Lactobacillales;Other
k__Bacteria;p__Firmicutes;c__Bacilli;o__Lactobacillales;f__
k__Bacteria;p__Firmicutes;c__Bacilli;o__Lactobacillales;f__Aerococcaceae
k__Bacteria;p__Firmicutes;c__Bacilli;o__Lactobacillales;f__Carnobacteriaceae
k__Bacteria;p__Firmicutes;c__Bacilli;o__Lactobacillales;f__Enterococcaceae
k__Bacteria;p__Firmicutes;c__Bacilli;o__Lactobacillales;f__Lactobacillaceae
k__Bacteria;p__Firmicutes;c__Bacilli;o__Lactobacillales;f__Streptococcaceae
k__Bacteria;p__Firmicutes;c__Bacilli;o__Turicibacterales;f__Turicibacteraceae
k__Bacteria;p__Firmicutes;c__Clostridia;o__Clostridiales;Other
k__Bacteria;p__Firmicutes;c__Clostridia;o__Clostridiales;f__
k__Bacteria;p__Firmicutes;c__Clostridia;o__Clostridiales;f__Christensenellaceae
k__Bacteria;p__Firmicutes;c__Clostridia;o__Clostridiales;f__Clostridiaceae
k__Bacteria;p__Firmicutes;c__Clostridia;o__Clostridiales;f__Lachnospiraceae
k__Bacteria;p__Firmicutes;c__Clostridia;o__Clostridiales;f__Peptostreptococcaceae
k__Bacteria;p__Firmicutes;c__Clostridia;o__Clostridiales;f__Ruminococcaceae
k__Bacteria;p__Firmicutes;c__Clostridia;o__Clostridiales;f__Veillonellaceae
k__Bacteria;p__Firmicutes;c__Clostridia;o__Clostridiales;f__[Tissierellaceae]
k__Bacteria;p__Firmicutes;c__Erysipelotrichi;o__Erysipelotrichales;f__Erysipelotrichaceae
k__Bacteria;p__Fusobacteria;c__Fusobacteriia;o__Fusobacteriales;f__Fusobacteriaceae
k__Bacteria;p__Fusobacteria;c__Fusobacteriia;o__Fusobacteriales;f__Leptotrichiaceae
k__Bacteria;p__Lentisphaerae;c__[Lentisphaeria];o__Victivallales;f__Victivallaceae
k__Bacteria;p__Proteobacteria;Other;Other;Other
k__Bacteria;p__Proteobacteria;c__Alphaproteobacteria;Other;Other
k__Bacteria;p__Proteobacteria;c__Alphaproteobacteria;o__;f__
k__Bacteria;p__Proteobacteria;c__Alphaproteobacteria;o__BD7-3;f__
k__Bacteria;p__Proteobacteria;c__Alphaproteobacteria;o__Caulobacterales;f__Caulobacteraceae
k__Bacteria;p__Proteobacteria;c__Alphaproteobacteria;o__RF32;f__
k__Bacteria;p__Proteobacteria;c__Alphaproteobacteria;o__Rhizobiales;Other
k__Bacteria;p__Proteobacteria;c__Alphaproteobacteria;o__Rhizobiales;f__Beijerinckiaceae
k__Bacteria;p__Proteobacteria;c__Alphaproteobacteria;o__Rhizobiales;f__Bradyrhizobiaceae
k__Bacteria;p__Proteobacteria;c__Alphaproteobacteria;o__Rhizobiales;f__Hyphomicrobiaceae
k__Bacteria;p__Proteobacteria;c__Alphaproteobacteria;o__Rhizobiales;f__Methylobacteriaceae
k__Bacteria;p__Proteobacteria;c__Alphaproteobacteria;o__Rhizobiales;f__Methylocystaceae
k__Bacteria;p__Proteobacteria;c__Alphaproteobacteria;o__Rhizobiales;f__Rhizobiaceae
k__Bacteria;p__Proteobacteria;c__Alphaproteobacteria;o__Rhodospirillales;f__
k__Bacteria;p__Proteobacteria;c__Alphaproteobacteria;o__Rhodospirillales;f__Acetobacteraceae
k__Bacteria;p__Proteobacteria;c__Alphaproteobacteria;o__Rickettsiales;Other
k__Bacteria;p__Proteobacteria;c__Alphaproteobacteria;o__Rickettsiales;f__
k__Bacteria;p__Proteobacteria;c__Alphaproteobacteria;o__Rickettsiales;f__Rickettsiaceae
k__Bacteria;p__Proteobacteria;c__Alphaproteobacteria;o__Rickettsiales;f__mitochondria
k__Bacteria;p__Proteobacteria;c__Alphaproteobacteria;o__Sphingomonadales;f__Erythrobacteraceae
k__Bacteria;p__Proteobacteria;c__Alphaproteobacteria;o__Sphingomonadales;f__Sphingomonadaceae
k__Bacteria;p__Proteobacteria;c__Betaproteobacteria;Other;Other
k__Bacteria;p__Proteobacteria;c__Betaproteobacteria;o__Burkholderiales;Other
k__Bacteria;p__Proteobacteria;c__Betaproteobacteria;o__Burkholderiales;f__Burkholderiaceae
k__Bacteria;p__Proteobacteria;c__Betaproteobacteria;o__Burkholderiales;f__Comamonadaceae
k__Bacteria;p__Proteobacteria;c__Betaproteobacteria;o__Burkholderiales;f__Oxalobacteraceae
k__Bacteria;p__Proteobacteria;c__Betaproteobacteria;o__Neisseriales;f__Neisseriaceae
k__Bacteria;p__Proteobacteria;c__Betaproteobacteria;o__Rhodocyclales;f__Rhodocyclaceae
k__Bacteria;p__Proteobacteria;c__Deltaproteobacteria;o__Bdellovibrionales;f__Bdellovibrionaceae
k__Bacteria;p__Proteobacteria;c__Epsilonproteobacteria;o__Campylobacterales;f__Campylobacteraceae
k__Bacteria;p__Proteobacteria;c__Epsilonproteobacteria;o__Campylobacterales;f__Helicobacteraceae
k__Bacteria;p__Proteobacteria;c__Gammaproteobacteria;Other;Other
k__Bacteria;p__Proteobacteria;c__Gammaproteobacteria;o__Aeromonadales;f__Aeromonadaceae
k__Bacteria;p__Proteobacteria;c__Gammaproteobacteria;o__Aeromonadales;f__Succinivibrionaceae
k__Bacteria;p__Proteobacteria;c__Gammaproteobacteria;o__Alteromonadales;f__Shewanellaceae
k__Bacteria;p__Proteobacteria;c__Gammaproteobacteria;o__Enterobacteriales;f__Enterobacteriaceae
k__Bacteria;p__Proteobacteria;c__Gammaproteobacteria;o__Pasteurellales;f__Pasteurellaceae
k__Bacteria;p__Proteobacteria;c__Gammaproteobacteria;o__Pseudomonadales;f__Moraxellaceae
k__Bacteria;p__Proteobacteria;c__Gammaproteobacteria;o__Pseudomonadales;f__Pseudomonadaceae
k__Bacteria;p__Proteobacteria;c__Gammaproteobacteria;o__Vibrionales;Other
k__Bacteria;p__Proteobacteria;c__Gammaproteobacteria;o__Vibrionales;f__Vibrionaceae
k__Bacteria;p__Proteobacteria;c__Gammaproteobacteria;o__Xanthomonadales;f__Xanthomonadaceae
k__Bacteria;p__Spirochaetes;c__[Brachyspirae];o__[Brachyspirales];f__Brachyspiraceae
k__Bacteria;p__Tenericutes;c__Mollicutes;o__Mycoplasmatales;f__Mycoplasmataceae
k__Bacteria;p__Tenericutes;c__Mollicutes;o__RF39;f__
k__Bacteria;p__Verrucomicrobia;c__Verruco-5;o__WCHB1-41;f__RFP12
k__Bacteria;p__Verrucomicrobia;c__Verrucomicrobiae;o__Verrucomicrobiales;f__Verrucomicrobiaceae
k__Bacteria;p__WPS-2;c__;o__;f__
k__Bacteria;p__[Thermi];c__Deinococci;o__Deinococcales;f__Deinococcaceae
