# Supplementary material for: Characterization of the nasopharyngeal microbiota in health and during rhinovirus challenge
Source: Microbiome. 2014 Jun 25;2:22. doi: 10.1186/2049-2618-2-22 (PMC4098959; doi:10.1186/2049-2618-2-22)
Supplement: Additional file 2 — Qiime taxonomic composition results (compressed). Taxa summary plots for all samples. Bar and area charts describing the taxonomic composition of all samples analyzed at all taxonomic ranks from genus to phylum. [file 2049-2618-2-22-S2.zip › 8440692981118328_add2.tgz!/Tax_composition_less1/taxa_summary_plots/charts/oZbDHCfnAWlKcWEn3kqDCEycRwzAXb_legend.pdf]

Unclassified;Other;Other;Other
k__Bacteria;Other;Other;Other
k__Bacteria;p__Actinobacteria;Other;Other
k__Bacteria;p__Actinobacteria;c__Actinobacteria;Other
k__Bacteria;p__Actinobacteria;c__Actinobacteria;o__Actinomycetales
k__Bacteria;p__Actinobacteria;c__Actinobacteria;o__Bifidobacteriales
k__Bacteria;p__Actinobacteria;c__Coriobacteriia;o__Coriobacteriales
k__Bacteria;p__Bacteroidetes;Other;Other
k__Bacteria;p__Bacteroidetes;c__Bacteroidia;o__Bacteroidales
k__Bacteria;p__Bacteroidetes;c__Cytophagia;o__Cytophagales
k__Bacteria;p__Bacteroidetes;c__Flavobacteriia;o__Flavobacteriales
k__Bacteria;p__Cyanobacteria;Other;Other
k__Bacteria;p__Cyanobacteria;c__4C0d-2;o__YS2
k__Bacteria;p__Cyanobacteria;c__Synechococcophycideae;o__Synechococcales
k__Bacteria;p__Firmicutes;Other;Other
k__Bacteria;p__Firmicutes;c__Bacilli;Other
k__Bacteria;p__Firmicutes;c__Bacilli;o__Bacillales
k__Bacteria;p__Firmicutes;c__Bacilli;o__Gemellales
k__Bacteria;p__Firmicutes;c__Bacilli;o__Lactobacillales
k__Bacteria;p__Firmicutes;c__Bacilli;o__Turicibacterales
k__Bacteria;p__Firmicutes;c__Clostridia;o__Clostridiales
k__Bacteria;p__Firmicutes;c__Erysipelotrichi;o__Erysipelotrichales
k__Bacteria;p__Fusobacteria;c__Fusobacteriia;o__Fusobacteriales
k__Bacteria;p__Lentisphaerae;c__[Lentisphaeria];o__Victivallales
k__Bacteria;p__Proteobacteria;Other;Other
k__Bacteria;p__Proteobacteria;c__Alphaproteobacteria;Other
k__Bacteria;p__Proteobacteria;c__Alphaproteobacteria;o__
k__Bacteria;p__Proteobacteria;c__Alphaproteobacteria;o__BD7-3
k__Bacteria;p__Proteobacteria;c__Alphaproteobacteria;o__Caulobacterales
k__Bacteria;p__Proteobacteria;c__Alphaproteobacteria;o__RF32
k__Bacteria;p__Proteobacteria;c__Alphaproteobacteria;o__Rhizobiales
k__Bacteria;p__Proteobacteria;c__Alphaproteobacteria;o__Rhodospirillales
k__Bacteria;p__Proteobacteria;c__Alphaproteobacteria;o__Rickettsiales
k__Bacteria;p__Proteobacteria;c__Alphaproteobacteria;o__Sphingomonadales
k__Bacteria;p__Proteobacteria;c__Betaproteobacteria;Other
k__Bacteria;p__Proteobacteria;c__Betaproteobacteria;o__Burkholderiales
k__Bacteria;p__Proteobacteria;c__Betaproteobacteria;o__Neisseriales
k__Bacteria;p__Proteobacteria;c__Betaproteobacteria;o__Rhodocyclales
k__Bacteria;p__Proteobacteria;c__Deltaproteobacteria;o__Bdellovibrionales
k__Bacteria;p__Proteobacteria;c__Epsilonproteobacteria;o__Campylobacterales
k__Bacteria;p__Proteobacteria;c__Gammaproteobacteria;Other
k__Bacteria;p__Proteobacteria;c__Gammaproteobacteria;o__Aeromonadales
k__Bacteria;p__Proteobacteria;c__Gammaproteobacteria;o__Alteromonadales
k__Bacteria;p__Proteobacteria;c__Gammaproteobacteria;o__Enterobacteriales
k__Bacteria;p__Proteobacteria;c__Gammaproteobacteria;o__Pasteurellales
k__Bacteria;p__Proteobacteria;c__Gammaproteobacteria;o__Pseudomonadales
k__Bacteria;p__Proteobacteria;c__Gammaproteobacteria;o__Vibrionales
k__Bacteria;p__Proteobacteria;c__Gammaproteobacteria;o__Xanthomonadales
k__Bacteria;p__Spirochaetes;c__[Brachyspirae];o__[Brachyspirales]
k__Bacteria;p__Tenericutes;c__Mollicutes;o__Mycoplasmatales
k__Bacteria;p__Tenericutes;c__Mollicutes;o__RF39
k__Bacteria;p__Verrucomicrobia;c__Verruco-5;o__WCHB1-41
k__Bacteria;p__Verrucomicrobia;c__Verrucomicrobiae;o__Verrucomicrobiales
k__Bacteria;p__WPS-2;c__;o__
k__Bacteria;p__[Thermi];c__Deinococci;o__Deinococcales
