# Supplementary material for: Characterization of the nasopharyngeal microbiota in health and during rhinovirus challenge
Source: Microbiome. 2014 Jun 25;2:22. doi: 10.1186/2049-2618-2-22 (PMC4098959; doi:10.1186/2049-2618-2-22)
Supplement: Additional file 2 — Qiime taxonomic composition results (compressed). Taxa summary plots for all samples. Bar and area charts describing the taxonomic composition of all samples analyzed at all taxonomic ranks from genus to phylum. [file 2049-2618-2-22-S2.zip › 8440692981118328_add2.tgz!/Tax_composition_less1/taxa_summary_plots/charts/Su8Rm7hYNhkGyDAZ2KHh7eHKsfn8Lu.pdf]

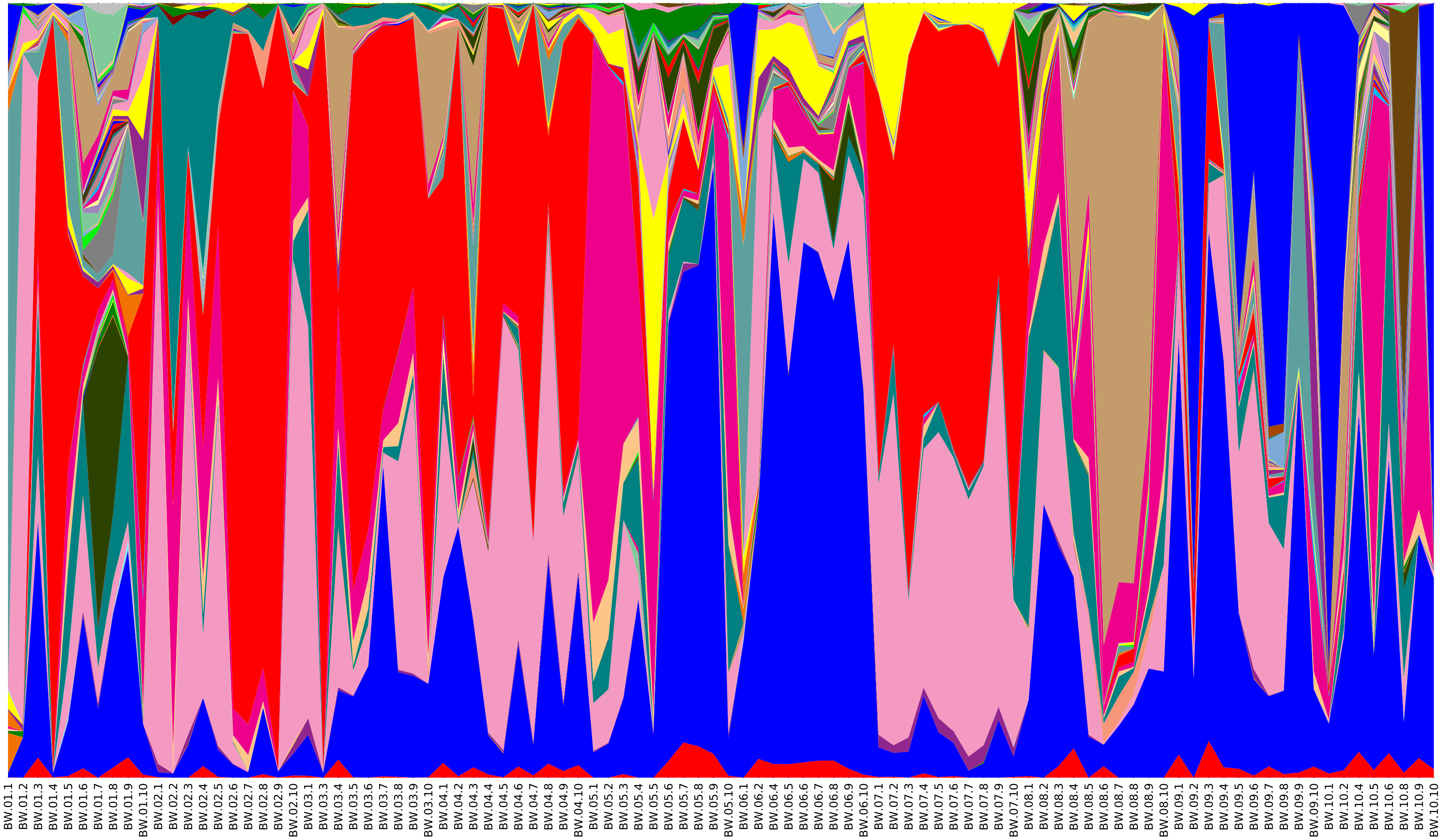
BW.01.1
BW.01.2
BW.01.3
BW.01.4
BW.01.5
BW.01.6
BW.01.7
BW.01.8
BW.01.9
BW.01.10
BW.02.1
BW.02.2
BW.02.3
BW.02.4
BW.02.5
BW.02.6
BW.02.7
BW.02.8
BW.02.9
BW.02.10
BW.03.1
BW.03.3
BW.03.4
BW.03.5
BW.03.6
BW.03.7
BW.03.8
BW.03.9
BW.03.10
BW.04.1
BW.04.2
BW.04.3
BW.04.4
BW.04.5
BW.04.6
BW.04.7
BW.04.8
BW.04.9
BW.04.10
BW.05.1
BW.05.2
BW.05.3
BW.05.4
BW.05.5
BW.05.6
BW.05.7
BW.05.8
BW.05.9
BW.05.10
BW.06.1
BW.06.2
BW.06.4
BW.06.5
BW.06.6
BW.06.7
BW.06.8
BW.06.9
BW.06.10
BW.07.1
BW.07.2
BW.07.3
BW.07.4
BW.07.5
BW.07.6
BW.07.7
BW.07.8
BW.07.9
BW.07.10
BW.08.1
BW.08.2
BW.08.3
BW.08.4
BW.08.5
BW.08.6
BW.08.7
BW.08.8
BW.08.9
BW.08.10
BW.09.1
BW.09.2
BW.09.3
BW.09.4
BW.09.5
BW.09.6
BW.09.7
BW.09.8
BW.09.9
BW.09.10
BW.10.1
BW.10.2
BW.10.4
BW.10.5
BW.10.6
BW.10.8
BW.10.9
BW.10.10
