# Supplementary material for: Characterization of the nasopharyngeal microbiota in health and during rhinovirus challenge
Source: Microbiome. 2014 Jun 25;2:22. doi: 10.1186/2049-2618-2-22 (PMC4098959; doi:10.1186/2049-2618-2-22)
Supplement: Additional file 3 — Qiime taxonomic composition results (compressed). Taxa summary plots organized by subject. Bar and area charts describing the taxonomic composition of all samples analyzed at all taxonomic ranks from genus to phylum, organized by subject. [file 2049-2618-2-22-S3.zip › 8440692981118328_add3.tgz!/Tax_composition_less1_bySubject/taxa_summary_plots/charts/Ng0tcAQSMnsztElMlPuK7pz7jLs9Q0_legend.pdf]

Unclassified;Other;Other
k__Bacteria;Other;Other
k__Bacteria;p__Actinobacteria;Other
k__Bacteria;p__Actinobacteria;c__Actinobacteria
k__Bacteria;p__Actinobacteria;c__Coriobacteriia
k__Bacteria;p__Bacteroidetes;Other
k__Bacteria;p__Bacteroidetes;c__Bacteroidia
k__Bacteria;p__Bacteroidetes;c__Cytophagia
k__Bacteria;p__Bacteroidetes;c__Flavobacteriia
k__Bacteria;p__Cyanobacteria;Other
k__Bacteria;p__Cyanobacteria;c__4C0d-2
k__Bacteria;p__Cyanobacteria;c__Synechococcophycideae
k__Bacteria;p__Firmicutes;Other
k__Bacteria;p__Firmicutes;c__Bacilli
k__Bacteria;p__Firmicutes;c__Clostridia
k__Bacteria;p__Firmicutes;c__Erysipelotrichi
k__Bacteria;p__Fusobacteria;c__Fusobacteriia
k__Bacteria;p__Lentisphaerae;c__[Lentisphaeria]
k__Bacteria;p__Proteobacteria;Other
k__Bacteria;p__Proteobacteria;c__Alphaproteobacteria
k__Bacteria;p__Proteobacteria;c__Betaproteobacteria
k__Bacteria;p__Proteobacteria;c__Deltaproteobacteria
k__Bacteria;p__Proteobacteria;c__Epsilonproteobacteria
k__Bacteria;p__Proteobacteria;c__Gammaproteobacteria
k__Bacteria;p__Spirochaetes;c__[Brachyspirae]
k__Bacteria;p__Tenericutes;c__Mollicutes
k__Bacteria;p__Verrucomicrobia;c__Verruco-5
k__Bacteria;p__Verrucomicrobia;c__Verrucomicrobiae
k__Bacteria;p__WPS-2;c__
k__Bacteria;p__[Thermi];c__Deinococci
