# Supplementary material for: Characterization of the nasopharyngeal microbiota in health and during rhinovirus challenge
Source: Microbiome. 2014 Jun 25;2:22. doi: 10.1186/2049-2618-2-22 (PMC4098959; doi:10.1186/2049-2618-2-22)
Supplement: Additional file 4 — Qiime taxonomic composition results (compressed). Taxa summary plots organized by time point. Bar and area charts describing the taxonomic composition of all samples analyzed at all taxonomic ranks from genus to phylum, organized by time point. [file 2049-2618-2-22-S4.zip › 8440692981118328_add4.tgz!/Tax_composition_less1_byTime/taxa_summary_plots/area_charts.html]

Taxa Summaries


|  |  |
| --- | --- |
|  | |
| Taxonomy Summary. Current Level: | |
| View Figure (.pdf)  View Legend (.pdf) |  |
|  |  |


View Table (.txt)

|  |  |  |  |  |  |  |  |  |  |  |  |  |  |
| --- | --- | --- | --- | --- | --- | --- | --- | --- | --- | --- | --- | --- | --- |
|  | | Total | | 1 | 2 | 3 | 4 | 5 | 6 | 7 | 8 | 9 | 10 |
| Legend | Taxonomy | count | % | % | % | % | % | % | % | % | % | % | % |
|  | Unclassified;Other | 0 | 0.8% | 0.3% | 0.3% | 1.3% | 1.1% | 0.4% | 1.1% | 0.9% | 1.1% | 0.8% | 0.7% |
|  | k\_\_Bacteria;Other | 2 | 18.1% | 7.9% | 12.5% | 19.7% | 24.4% | 11.9% | 24.0% | 19.0% | 20.8% | 24.6% | 16.6% |
|  | k\_\_Bacteria;p\_\_Actinobacteria | 2 | 19.4% | 26.6% | 13.5% | 19.4% | 17.3% | 29.4% | 20.4% | 15.8% | 21.3% | 12.7% | 17.5% |
|  | k\_\_Bacteria;p\_\_Bacteroidetes | 0 | 0.3% | 1.5% | 0.2% | 0.1% | 0.3% | 0.1% | 0.2% | 0.1% | 0.3% | 0.1% | 0.1% |
|  | k\_\_Bacteria;p\_\_Cyanobacteria | 0 | 0.0% | 0.0% | 0.0% | 0.0% | 0.0% | 0.0% | 0.0% | 0.0% | 0.0% | 0.0% | 0.0% |
|  | k\_\_Bacteria;p\_\_Firmicutes | 5 | 47.4% | 47.7% | 44.2% | 53.3% | 48.0% | 48.8% | 40.9% | 50.5% | 35.3% | 56.3% | 49.0% |
|  | k\_\_Bacteria;p\_\_Fusobacteria | 0 | 0.1% | 0.5% | 0.0% | 0.0% | 0.0% | 0.0% | 0.0% | 0.0% | 0.0% | 0.0% | 0.1% |
|  | k\_\_Bacteria;p\_\_Lentisphaerae | 0 | 0.0% | 0.0% | 0.0% | 0.0% | 0.0% | 0.0% | 0.0% | 0.0% | 0.0% | 0.0% | 0.0% |
|  | k\_\_Bacteria;p\_\_Proteobacteria | 1 | 13.7% | 15.5% | 29.1% | 6.1% | 8.8% | 9.4% | 13.2% | 13.1% | 20.2% | 5.2% | 15.9% |
|  | k\_\_Bacteria;p\_\_Spirochaetes | 0 | 0.0% | 0.0% | 0.0% | 0.0% | 0.0% | 0.0% | 0.0% | 0.0% | 0.0% | 0.0% | 0.0% |
|  | k\_\_Bacteria;p\_\_Tenericutes | 0 | 0.0% | 0.0% | 0.0% | 0.0% | 0.0% | 0.0% | 0.0% | 0.0% | 0.1% | 0.0% | 0.0% |
|  | k\_\_Bacteria;p\_\_Verrucomicrobia | 0 | 0.1% | 0.0% | 0.0% | 0.0% | 0.0% | 0.0% | 0.0% | 0.5% | 0.6% | 0.1% | 0.0% |
|  | k\_\_Bacteria;p\_\_WPS-2 | 0 | 0.0% | 0.0% | 0.0% | 0.0% | 0.0% | 0.0% | 0.0% | 0.1% | 0.1% | 0.0% | 0.0% |
|  | k\_\_Bacteria;p\_\_[Thermi] | 0 | 0.0% | 0.0% | 0.0% | 0.0% | 0.0% | 0.0% | 0.0% | 0.0% | 0.0% | 0.0% | 0.0% |

|  |  |
| --- | --- |
|  | |
| Taxonomy Summary. Current Level: | |
| View Figure (.pdf)  View Legend (.pdf) |  |
|  |  |


View Table (.txt)

|  |  |  |  |  |  |  |  |  |  |  |  |  |  |
| --- | --- | --- | --- | --- | --- | --- | --- | --- | --- | --- | --- | --- | --- |
|  | | Total | | 1 | 2 | 3 | 4 | 5 | 6 | 7 | 8 | 9 | 10 |
| Legend | Taxonomy | count | % | % | % | % | % | % | % | % | % | % | % |
|  | Unclassified;Other;Other | 0 | 0.8% | 0.3% | 0.3% | 1.3% | 1.1% | 0.4% | 1.1% | 0.9% | 1.1% | 0.8% | 0.7% |
|  | k\_\_Bacteria;Other;Other | 2 | 18.1% | 7.9% | 12.5% | 19.7% | 24.4% | 11.9% | 24.0% | 19.0% | 20.8% | 24.6% | 16.6% |
|  | k\_\_Bacteria;p\_\_Actinobacteria;Other | 0 | 0.0% | 0.0% | 0.0% | 0.0% | 0.0% | 0.0% | 0.0% | 0.0% | 0.0% | 0.0% | 0.0% |
|  | k\_\_Bacteria;p\_\_Actinobacteria;c\_\_Actinobacteria | 2 | 19.4% | 26.6% | 13.5% | 19.3% | 17.3% | 29.3% | 20.4% | 15.8% | 21.3% | 12.7% | 17.5% |
|  | k\_\_Bacteria;p\_\_Actinobacteria;c\_\_Coriobacteriia | 0 | 0.0% | 0.0% | 0.0% | 0.0% | 0.0% | 0.0% | 0.0% | 0.0% | 0.0% | 0.0% | 0.0% |
|  | k\_\_Bacteria;p\_\_Bacteroidetes;Other | 0 | 0.0% | 0.0% | 0.0% | 0.0% | 0.0% | 0.0% | 0.0% | 0.0% | 0.0% | 0.0% | 0.0% |
|  | k\_\_Bacteria;p\_\_Bacteroidetes;c\_\_Bacteroidia | 0 | 0.3% | 1.5% | 0.2% | 0.1% | 0.2% | 0.1% | 0.2% | 0.1% | 0.2% | 0.1% | 0.1% |
|  | k\_\_Bacteria;p\_\_Bacteroidetes;c\_\_Cytophagia | 0 | 0.0% | 0.0% | 0.0% | 0.0% | 0.1% | 0.0% | 0.0% | 0.0% | 0.0% | 0.0% | 0.0% |
|  | k\_\_Bacteria;p\_\_Bacteroidetes;c\_\_Flavobacteriia | 0 | 0.0% | 0.0% | 0.0% | 0.0% | 0.0% | 0.0% | 0.0% | 0.0% | 0.1% | 0.0% | 0.0% |
|  | k\_\_Bacteria;p\_\_Cyanobacteria;Other | 0 | 0.0% | 0.0% | 0.0% | 0.0% | 0.0% | 0.0% | 0.0% | 0.0% | 0.0% | 0.0% | 0.0% |
|  | k\_\_Bacteria;p\_\_Cyanobacteria;c\_\_4C0d-2 | 0 | 0.0% | 0.0% | 0.0% | 0.0% | 0.0% | 0.0% | 0.0% | 0.0% | 0.0% | 0.0% | 0.0% |
|  | k\_\_Bacteria;p\_\_Cyanobacteria;c\_\_Synechococcophycideae | 0 | 0.0% | 0.0% | 0.0% | 0.0% | 0.0% | 0.0% | 0.0% | 0.0% | 0.0% | 0.0% | 0.0% |
|  | k\_\_Bacteria;p\_\_Firmicutes;Other | 0 | 0.0% | 0.0% | 0.0% | 0.0% | 0.0% | 0.0% | 0.0% | 0.1% | 0.0% | 0.0% | 0.0% |
|  | k\_\_Bacteria;p\_\_Firmicutes;c\_\_Bacilli | 4 | 44.5% | 44.8% | 41.2% | 51.7% | 45.8% | 44.2% | 37.9% | 47.9% | 33.1% | 55.3% | 43.0% |
|  | k\_\_Bacteria;p\_\_Firmicutes;c\_\_Clostridia | 0 | 2.9% | 2.9% | 3.0% | 1.6% | 2.2% | 4.6% | 3.0% | 2.5% | 2.2% | 1.0% | 5.9% |
|  | k\_\_Bacteria;p\_\_Firmicutes;c\_\_Erysipelotrichi | 0 | 0.0% | 0.0% | 0.0% | 0.0% | 0.0% | 0.0% | 0.0% | 0.0% | 0.1% | 0.0% | 0.0% |
|  | k\_\_Bacteria;p\_\_Fusobacteria;c\_\_Fusobacteriia | 0 | 0.1% | 0.5% | 0.0% | 0.0% | 0.0% | 0.0% | 0.0% | 0.0% | 0.0% | 0.0% | 0.1% |
|  | k\_\_Bacteria;p\_\_Lentisphaerae;c\_\_[Lentisphaeria] | 0 | 0.0% | 0.0% | 0.0% | 0.0% | 0.0% | 0.0% | 0.0% | 0.0% | 0.0% | 0.0% | 0.0% |
|  | k\_\_Bacteria;p\_\_Proteobacteria;Other | 0 | 0.1% | 0.0% | 0.1% | 0.1% | 0.0% | 0.0% | 0.1% | 0.0% | 0.1% | 0.0% | 0.0% |
|  | k\_\_Bacteria;p\_\_Proteobacteria;c\_\_Alphaproteobacteria | 0 | 4.2% | 1.1% | 2.5% | 2.5% | 4.1% | 3.2% | 8.5% | 5.5% | 7.9% | 2.9% | 3.5% |
|  | k\_\_Bacteria;p\_\_Proteobacteria;c\_\_Betaproteobacteria | 0 | 1.0% | 1.7% | 0.3% | 0.3% | 0.9% | 0.3% | 1.1% | 1.4% | 2.2% | 0.9% | 0.5% |
|  | k\_\_Bacteria;p\_\_Proteobacteria;c\_\_Deltaproteobacteria | 0 | 0.0% | 0.0% | 0.0% | 0.0% | 0.0% | 0.0% | 0.0% | 0.0% | 0.0% | 0.0% | 0.0% |
|  | k\_\_Bacteria;p\_\_Proteobacteria;c\_\_Epsilonproteobacteria | 0 | 0.0% | 0.0% | 0.1% | 0.0% | 0.0% | 0.0% | 0.0% | 0.0% | 0.0% | 0.0% | 0.0% |
|  | k\_\_Bacteria;p\_\_Proteobacteria;c\_\_Gammaproteobacteria | 1 | 8.5% | 12.7% | 26.2% | 3.2% | 3.8% | 5.9% | 3.5% | 6.2% | 10.0% | 1.4% | 11.8% |
|  | k\_\_Bacteria;p\_\_Spirochaetes;c\_\_[Brachyspirae] | 0 | 0.0% | 0.0% | 0.0% | 0.0% | 0.0% | 0.0% | 0.0% | 0.0% | 0.0% | 0.0% | 0.0% |
|  | k\_\_Bacteria;p\_\_Tenericutes;c\_\_Mollicutes | 0 | 0.0% | 0.0% | 0.0% | 0.0% | 0.0% | 0.0% | 0.0% | 0.0% | 0.1% | 0.0% | 0.0% |
|  | k\_\_Bacteria;p\_\_Verrucomicrobia;c\_\_Verruco-5 | 0 | 0.1% | 0.0% | 0.0% | 0.0% | 0.0% | 0.0% | 0.0% | 0.5% | 0.6% | 0.1% | 0.0% |
|  | k\_\_Bacteria;p\_\_Verrucomicrobia;c\_\_Verrucomicrobiae | 0 | 0.0% | 0.0% | 0.0% | 0.0% | 0.0% | 0.0% | 0.0% | 0.0% | 0.0% | 0.0% | 0.0% |
|  | k\_\_Bacteria;p\_\_WPS-2;c\_\_ | 0 | 0.0% | 0.0% | 0.0% | 0.0% | 0.0% | 0.0% | 0.0% | 0.1% | 0.1% | 0.0% | 0.0% |
|  | k\_\_Bacteria;p\_\_[Thermi];c\_\_Deinococci | 0 | 0.0% | 0.0% | 0.0% | 0.0% | 0.0% | 0.0% | 0.0% | 0.0% | 0.0% | 0.0% | 0.0% |

|  |  |
| --- | --- |
|  | |
| Taxonomy Summary. Current Level: | |
| View Figure (.pdf)  View Legend (.pdf) |  |
|  |  |


View Table (.txt)

|  |  |  |  |  |  |  |  |  |  |  |  |  |  |
| --- | --- | --- | --- | --- | --- | --- | --- | --- | --- | --- | --- | --- | --- |
|  | | Total | | 1 | 2 | 3 | 4 | 5 | 6 | 7 | 8 | 9 | 10 |
| Legend | Taxonomy | count | % | % | % | % | % | % | % | % | % | % | % |
|  | Unclassified;Other;Other;Other | 0 | 0.8% | 0.3% | 0.3% | 1.3% | 1.1% | 0.4% | 1.1% | 0.9% | 1.1% | 0.8% | 0.7% |
|  | k\_\_Bacteria;Other;Other;Other | 2 | 18.1% | 7.9% | 12.5% | 19.7% | 24.4% | 11.9% | 24.0% | 19.0% | 20.8% | 24.6% | 16.6% |
|  | k\_\_Bacteria;p\_\_Actinobacteria;Other;Other | 0 | 0.0% | 0.0% | 0.0% | 0.0% | 0.0% | 0.0% | 0.0% | 0.0% | 0.0% | 0.0% | 0.0% |
|  | k\_\_Bacteria;p\_\_Actinobacteria;c\_\_Actinobacteria;Other | 0 | 0.0% | 0.0% | 0.0% | 0.0% | 0.0% | 0.0% | 0.0% | 0.0% | 0.0% | 0.0% | 0.0% |
|  | k\_\_Bacteria;p\_\_Actinobacteria;c\_\_Actinobacteria;o\_\_Actinomycetales | 2 | 18.9% | 26.5% | 13.5% | 19.2% | 17.2% | 29.3% | 20.4% | 13.7% | 19.2% | 12.4% | 17.5% |
|  | k\_\_Bacteria;p\_\_Actinobacteria;c\_\_Actinobacteria;o\_\_Bifidobacteriales | 0 | 0.5% | 0.0% | 0.0% | 0.1% | 0.1% | 0.0% | 0.0% | 2.1% | 2.1% | 0.3% | 0.0% |
|  | k\_\_Bacteria;p\_\_Actinobacteria;c\_\_Coriobacteriia;o\_\_Coriobacteriales | 0 | 0.0% | 0.0% | 0.0% | 0.0% | 0.0% | 0.0% | 0.0% | 0.0% | 0.0% | 0.0% | 0.0% |
|  | k\_\_Bacteria;p\_\_Bacteroidetes;Other;Other | 0 | 0.0% | 0.0% | 0.0% | 0.0% | 0.0% | 0.0% | 0.0% | 0.0% | 0.0% | 0.0% | 0.0% |
|  | k\_\_Bacteria;p\_\_Bacteroidetes;c\_\_Bacteroidia;o\_\_Bacteroidales | 0 | 0.3% | 1.5% | 0.2% | 0.1% | 0.2% | 0.1% | 0.2% | 0.1% | 0.2% | 0.1% | 0.1% |
|  | k\_\_Bacteria;p\_\_Bacteroidetes;c\_\_Cytophagia;o\_\_Cytophagales | 0 | 0.0% | 0.0% | 0.0% | 0.0% | 0.1% | 0.0% | 0.0% | 0.0% | 0.0% | 0.0% | 0.0% |
|  | k\_\_Bacteria;p\_\_Bacteroidetes;c\_\_Flavobacteriia;o\_\_Flavobacteriales | 0 | 0.0% | 0.0% | 0.0% | 0.0% | 0.0% | 0.0% | 0.0% | 0.0% | 0.1% | 0.0% | 0.0% |
|  | k\_\_Bacteria;p\_\_Cyanobacteria;Other;Other | 0 | 0.0% | 0.0% | 0.0% | 0.0% | 0.0% | 0.0% | 0.0% | 0.0% | 0.0% | 0.0% | 0.0% |
|  | k\_\_Bacteria;p\_\_Cyanobacteria;c\_\_4C0d-2;o\_\_YS2 | 0 | 0.0% | 0.0% | 0.0% | 0.0% | 0.0% | 0.0% | 0.0% | 0.0% | 0.0% | 0.0% | 0.0% |
|  | k\_\_Bacteria;p\_\_Cyanobacteria;c\_\_Synechococcophycideae;o\_\_Synechococcales | 0 | 0.0% | 0.0% | 0.0% | 0.0% | 0.0% | 0.0% | 0.0% | 0.0% | 0.0% | 0.0% | 0.0% |
|  | k\_\_Bacteria;p\_\_Firmicutes;Other;Other | 0 | 0.0% | 0.0% | 0.0% | 0.0% | 0.0% | 0.0% | 0.0% | 0.1% | 0.0% | 0.0% | 0.0% |
|  | k\_\_Bacteria;p\_\_Firmicutes;c\_\_Bacilli;Other | 0 | 1.2% | 1.4% | 2.3% | 1.1% | 1.1% | 1.8% | 0.9% | 0.7% | 0.8% | 0.6% | 1.5% |
|  | k\_\_Bacteria;p\_\_Firmicutes;c\_\_Bacilli;o\_\_Bacillales | 1 | 7.4% | 12.0% | 18.6% | 6.8% | 4.0% | 10.3% | 3.0% | 2.0% | 3.0% | 3.8% | 10.5% |
|  | k\_\_Bacteria;p\_\_Firmicutes;c\_\_Bacilli;o\_\_Gemellales | 0 | 0.1% | 0.2% | 0.1% | 0.2% | 0.1% | 0.2% | 0.1% | 0.0% | 0.1% | 0.0% | 0.3% |
|  | k\_\_Bacteria;p\_\_Firmicutes;c\_\_Bacilli;o\_\_Lactobacillales | 4 | 35.7% | 31.2% | 20.3% | 43.6% | 40.6% | 31.9% | 33.9% | 45.1% | 29.3% | 50.8% | 30.8% |
|  | k\_\_Bacteria;p\_\_Firmicutes;c\_\_Bacilli;o\_\_Turicibacterales | 0 | 0.0% | 0.0% | 0.0% | 0.0% | 0.0% | 0.0% | 0.0% | 0.1% | 0.0% | 0.0% | 0.0% |
|  | k\_\_Bacteria;p\_\_Firmicutes;c\_\_Clostridia;o\_\_Clostridiales | 0 | 2.9% | 2.9% | 3.0% | 1.6% | 2.2% | 4.6% | 3.0% | 2.5% | 2.2% | 1.0% | 5.9% |
|  | k\_\_Bacteria;p\_\_Firmicutes;c\_\_Erysipelotrichi;o\_\_Erysipelotrichales | 0 | 0.0% | 0.0% | 0.0% | 0.0% | 0.0% | 0.0% | 0.0% | 0.0% | 0.1% | 0.0% | 0.0% |
|  | k\_\_Bacteria;p\_\_Fusobacteria;c\_\_Fusobacteriia;o\_\_Fusobacteriales | 0 | 0.1% | 0.5% | 0.0% | 0.0% | 0.0% | 0.0% | 0.0% | 0.0% | 0.0% | 0.0% | 0.1% |
|  | k\_\_Bacteria;p\_\_Lentisphaerae;c\_\_[Lentisphaeria];o\_\_Victivallales | 0 | 0.0% | 0.0% | 0.0% | 0.0% | 0.0% | 0.0% | 0.0% | 0.0% | 0.0% | 0.0% | 0.0% |
|  | k\_\_Bacteria;p\_\_Proteobacteria;Other;Other | 0 | 0.1% | 0.0% | 0.1% | 0.1% | 0.0% | 0.0% | 0.1% | 0.0% | 0.1% | 0.0% | 0.0% |
|  | k\_\_Bacteria;p\_\_Proteobacteria;c\_\_Alphaproteobacteria;Other | 0 | 0.4% | 0.1% | 0.3% | 0.3% | 0.3% | 0.4% | 0.8% | 0.6% | 0.7% | 0.4% | 0.3% |
|  | k\_\_Bacteria;p\_\_Proteobacteria;c\_\_Alphaproteobacteria;o\_\_ | 0 | 0.0% | 0.0% | 0.0% | 0.0% | 0.0% | 0.0% | 0.0% | 0.0% | 0.0% | 0.0% | 0.0% |
|  | k\_\_Bacteria;p\_\_Proteobacteria;c\_\_Alphaproteobacteria;o\_\_BD7-3 | 0 | 3.1% | 0.7% | 1.9% | 1.8% | 2.5% | 2.3% | 6.3% | 4.3% | 5.6% | 2.3% | 3.0% |
|  | k\_\_Bacteria;p\_\_Proteobacteria;c\_\_Alphaproteobacteria;o\_\_Caulobacterales | 0 | 0.0% | 0.0% | 0.0% | 0.0% | 0.1% | 0.0% | 0.0% | 0.0% | 0.0% | 0.0% | 0.0% |
|  | k\_\_Bacteria;p\_\_Proteobacteria;c\_\_Alphaproteobacteria;o\_\_RF32 | 0 | 0.0% | 0.0% | 0.0% | 0.0% | 0.0% | 0.0% | 0.0% | 0.0% | 0.0% | 0.0% | 0.0% |
|  | k\_\_Bacteria;p\_\_Proteobacteria;c\_\_Alphaproteobacteria;o\_\_Rhizobiales | 0 | 0.1% | 0.0% | 0.0% | 0.2% | 0.2% | 0.0% | 0.2% | 0.0% | 0.2% | 0.0% | 0.0% |
|  | k\_\_Bacteria;p\_\_Proteobacteria;c\_\_Alphaproteobacteria;o\_\_Rhodospirillales | 0 | 0.0% | 0.0% | 0.0% | 0.0% | 0.0% | 0.0% | 0.1% | 0.0% | 0.0% | 0.0% | 0.0% |
|  | k\_\_Bacteria;p\_\_Proteobacteria;c\_\_Alphaproteobacteria;o\_\_Rickettsiales | 0 | 0.4% | 0.1% | 0.2% | 0.1% | 0.5% | 0.2% | 0.7% | 0.6% | 1.1% | 0.0% | 0.1% |
|  | k\_\_Bacteria;p\_\_Proteobacteria;c\_\_Alphaproteobacteria;o\_\_Sphingomonadales | 0 | 0.2% | 0.1% | 0.1% | 0.1% | 0.4% | 0.2% | 0.4% | 0.1% | 0.3% | 0.1% | 0.2% |
|  | k\_\_Bacteria;p\_\_Proteobacteria;c\_\_Betaproteobacteria;Other | 0 | 0.0% | 0.0% | 0.0% | 0.0% | 0.0% | 0.0% | 0.0% | 0.0% | 0.0% | 0.0% | 0.0% |
|  | k\_\_Bacteria;p\_\_Proteobacteria;c\_\_Betaproteobacteria;o\_\_Burkholderiales | 0 | 0.5% | 0.7% | 0.2% | 0.2% | 0.6% | 0.1% | 0.9% | 0.2% | 1.4% | 0.6% | 0.2% |
|  | k\_\_Bacteria;p\_\_Proteobacteria;c\_\_Betaproteobacteria;o\_\_Neisseriales | 0 | 0.4% | 0.8% | 0.1% | 0.1% | 0.2% | 0.1% | 0.1% | 1.1% | 0.7% | 0.2% | 0.3% |
|  | k\_\_Bacteria;p\_\_Proteobacteria;c\_\_Betaproteobacteria;o\_\_Rhodocyclales | 0 | 0.1% | 0.2% | 0.0% | 0.0% | 0.1% | 0.0% | 0.1% | 0.0% | 0.2% | 0.0% | 0.0% |
|  | k\_\_Bacteria;p\_\_Proteobacteria;c\_\_Deltaproteobacteria;o\_\_Bdellovibrionales | 0 | 0.0% | 0.0% | 0.0% | 0.0% | 0.0% | 0.0% | 0.0% | 0.0% | 0.0% | 0.0% | 0.0% |
|  | k\_\_Bacteria;p\_\_Proteobacteria;c\_\_Epsilonproteobacteria;o\_\_Campylobacterales | 0 | 0.0% | 0.0% | 0.1% | 0.0% | 0.0% | 0.0% | 0.0% | 0.0% | 0.0% | 0.0% | 0.0% |
|  | k\_\_Bacteria;p\_\_Proteobacteria;c\_\_Gammaproteobacteria;Other | 0 | 0.0% | 0.0% | 0.0% | 0.0% | 0.0% | 0.0% | 0.0% | 0.0% | 0.0% | 0.0% | 0.0% |
|  | k\_\_Bacteria;p\_\_Proteobacteria;c\_\_Gammaproteobacteria;o\_\_Aeromonadales | 0 | 0.0% | 0.0% | 0.0% | 0.0% | 0.0% | 0.0% | 0.0% | 0.0% | 0.0% | 0.0% | 0.0% |
|  | k\_\_Bacteria;p\_\_Proteobacteria;c\_\_Gammaproteobacteria;o\_\_Alteromonadales | 0 | 0.0% | 0.0% | 0.0% | 0.0% | 0.0% | 0.0% | 0.0% | 0.0% | 0.0% | 0.0% | 0.0% |
|  | k\_\_Bacteria;p\_\_Proteobacteria;c\_\_Gammaproteobacteria;o\_\_Enterobacteriales | 0 | 1.8% | 0.3% | 7.5% | 1.6% | 2.6% | 1.6% | 0.6% | 1.0% | 1.2% | 0.3% | 0.8% |
|  | k\_\_Bacteria;p\_\_Proteobacteria;c\_\_Gammaproteobacteria;o\_\_Pasteurellales | 1 | 5.2% | 11.0% | 15.1% | 0.5% | 0.2% | 3.7% | 1.7% | 4.5% | 4.4% | 0.6% | 10.6% |
|  | k\_\_Bacteria;p\_\_Proteobacteria;c\_\_Gammaproteobacteria;o\_\_Pseudomonadales | 0 | 1.1% | 1.4% | 3.4% | 0.9% | 0.6% | 0.5% | 1.1% | 0.6% | 1.4% | 0.5% | 0.4% |
|  | k\_\_Bacteria;p\_\_Proteobacteria;c\_\_Gammaproteobacteria;o\_\_Vibrionales | 0 | 0.3% | 0.0% | 0.1% | 0.0% | 0.0% | 0.0% | 0.1% | 0.0% | 2.9% | 0.0% | 0.0% |
|  | k\_\_Bacteria;p\_\_Proteobacteria;c\_\_Gammaproteobacteria;o\_\_Xanthomonadales | 0 | 0.0% | 0.0% | 0.0% | 0.1% | 0.2% | 0.0% | 0.0% | 0.0% | 0.0% | 0.0% | 0.0% |
|  | k\_\_Bacteria;p\_\_Spirochaetes;c\_\_[Brachyspirae];o\_\_[Brachyspirales] | 0 | 0.0% | 0.0% | 0.0% | 0.0% | 0.0% | 0.0% | 0.0% | 0.0% | 0.0% | 0.0% | 0.0% |
|  | k\_\_Bacteria;p\_\_Tenericutes;c\_\_Mollicutes;o\_\_Mycoplasmatales | 0 | 0.0% | 0.0% | 0.0% | 0.0% | 0.0% | 0.0% | 0.0% | 0.0% | 0.0% | 0.0% | 0.0% |
|  | k\_\_Bacteria;p\_\_Tenericutes;c\_\_Mollicutes;o\_\_RF39 | 0 | 0.0% | 0.0% | 0.0% | 0.0% | 0.0% | 0.0% | 0.0% | 0.0% | 0.1% | 0.0% | 0.0% |
|  | k\_\_Bacteria;p\_\_Verrucomicrobia;c\_\_Verruco-5;o\_\_WCHB1-41 | 0 | 0.1% | 0.0% | 0.0% | 0.0% | 0.0% | 0.0% | 0.0% | 0.5% | 0.6% | 0.1% | 0.0% |
|  | k\_\_Bacteria;p\_\_Verrucomicrobia;c\_\_Verrucomicrobiae;o\_\_Verrucomicrobiales | 0 | 0.0% | 0.0% | 0.0% | 0.0% | 0.0% | 0.0% | 0.0% | 0.0% | 0.0% | 0.0% | 0.0% |
|  | k\_\_Bacteria;p\_\_WPS-2;c\_\_;o\_\_ | 0 | 0.0% | 0.0% | 0.0% | 0.0% | 0.0% | 0.0% | 0.0% | 0.1% | 0.1% | 0.0% | 0.0% |
|  | k\_\_Bacteria;p\_\_[Thermi];c\_\_Deinococci;o\_\_Deinococcales | 0 | 0.0% | 0.0% | 0.0% | 0.0% | 0.0% | 0.0% | 0.0% | 0.0% | 0.0% | 0.0% | 0.0% |

|  |  |
| --- | --- |
|  | |
| Taxonomy Summary. Current Level: | |
| View Figure (.pdf)  View Legend (.pdf) |  |
|  |  |


View Table (.txt)

|  |  |  |  |  |  |  |  |  |  |  |  |  |  |
| --- | --- | --- | --- | --- | --- | --- | --- | --- | --- | --- | --- | --- | --- |
|  | | Total | | 1 | 2 | 3 | 4 | 5 | 6 | 7 | 8 | 9 | 10 |
| Legend | Taxonomy | count | % | % | % | % | % | % | % | % | % | % | % |
|  | Unclassified;Other;Other;Other;Other | 0 | 0.8% | 0.3% | 0.3% | 1.3% | 1.1% | 0.4% | 1.1% | 0.9% | 1.1% | 0.8% | 0.7% |
|  | k\_\_Bacteria;Other;Other;Other;Other | 2 | 18.1% | 7.9% | 12.5% | 19.7% | 24.4% | 11.9% | 24.0% | 19.0% | 20.8% | 24.6% | 16.6% |
|  | k\_\_Bacteria;p\_\_Actinobacteria;Other;Other;Other | 0 | 0.0% | 0.0% | 0.0% | 0.0% | 0.0% | 0.0% | 0.0% | 0.0% | 0.0% | 0.0% | 0.0% |
|  | k\_\_Bacteria;p\_\_Actinobacteria;c\_\_Actinobacteria;Other;Other | 0 | 0.0% | 0.0% | 0.0% | 0.0% | 0.0% | 0.0% | 0.0% | 0.0% | 0.0% | 0.0% | 0.0% |
|  | k\_\_Bacteria;p\_\_Actinobacteria;c\_\_Actinobacteria;o\_\_Actinomycetales;Other | 0 | 0.4% | 0.6% | 0.2% | 0.5% | 0.2% | 0.4% | 0.4% | 0.4% | 0.5% | 0.2% | 0.4% |
|  | k\_\_Bacteria;p\_\_Actinobacteria;c\_\_Actinobacteria;o\_\_Actinomycetales;f\_\_Actinomycetaceae | 0 | 0.0% | 0.0% | 0.0% | 0.0% | 0.0% | 0.1% | 0.0% | 0.0% | 0.0% | 0.1% | 0.0% |
|  | k\_\_Bacteria;p\_\_Actinobacteria;c\_\_Actinobacteria;o\_\_Actinomycetales;f\_\_Corynebacteriaceae | 1 | 14.9% | 21.8% | 10.2% | 13.7% | 13.5% | 23.7% | 15.2% | 11.4% | 15.5% | 9.4% | 14.3% |
|  | k\_\_Bacteria;p\_\_Actinobacteria;c\_\_Actinobacteria;o\_\_Actinomycetales;f\_\_Dermabacteraceae | 0 | 0.0% | 0.0% | 0.0% | 0.0% | 0.1% | 0.1% | 0.0% | 0.0% | 0.0% | 0.0% | 0.0% |
|  | k\_\_Bacteria;p\_\_Actinobacteria;c\_\_Actinobacteria;o\_\_Actinomycetales;f\_\_Dietziaceae | 0 | 0.0% | 0.0% | 0.0% | 0.0% | 0.0% | 0.0% | 0.0% | 0.0% | 0.0% | 0.0% | 0.0% |
|  | k\_\_Bacteria;p\_\_Actinobacteria;c\_\_Actinobacteria;o\_\_Actinomycetales;f\_\_Geodermatophilaceae | 0 | 0.0% | 0.0% | 0.0% | 0.0% | 0.0% | 0.0% | 0.0% | 0.0% | 0.0% | 0.0% | 0.0% |
|  | k\_\_Bacteria;p\_\_Actinobacteria;c\_\_Actinobacteria;o\_\_Actinomycetales;f\_\_Microbacteriaceae | 0 | 0.2% | 0.0% | 0.0% | 0.2% | 0.3% | 0.1% | 0.3% | 0.2% | 0.2% | 0.1% | 0.2% |
|  | k\_\_Bacteria;p\_\_Actinobacteria;c\_\_Actinobacteria;o\_\_Actinomycetales;f\_\_Micrococcaceae | 0 | 0.0% | 0.1% | 0.0% | 0.1% | 0.1% | 0.0% | 0.0% | 0.0% | 0.0% | 0.0% | 0.0% |
|  | k\_\_Bacteria;p\_\_Actinobacteria;c\_\_Actinobacteria;o\_\_Actinomycetales;f\_\_Nocardioidaceae | 0 | 0.0% | 0.0% | 0.0% | 0.0% | 0.0% | 0.0% | 0.0% | 0.0% | 0.0% | 0.0% | 0.0% |
|  | k\_\_Bacteria;p\_\_Actinobacteria;c\_\_Actinobacteria;o\_\_Actinomycetales;f\_\_Propionibacteriaceae | 0 | 3.4% | 4.0% | 3.0% | 4.7% | 2.9% | 4.9% | 4.4% | 1.6% | 3.0% | 2.6% | 2.5% |
|  | k\_\_Bacteria;p\_\_Actinobacteria;c\_\_Actinobacteria;o\_\_Bifidobacteriales;f\_\_Bifidobacteriaceae | 0 | 0.5% | 0.0% | 0.0% | 0.1% | 0.1% | 0.0% | 0.0% | 2.1% | 2.1% | 0.3% | 0.0% |
|  | k\_\_Bacteria;p\_\_Actinobacteria;c\_\_Coriobacteriia;o\_\_Coriobacteriales;f\_\_Coriobacteriaceae | 0 | 0.0% | 0.0% | 0.0% | 0.0% | 0.0% | 0.0% | 0.0% | 0.0% | 0.0% | 0.0% | 0.0% |
|  | k\_\_Bacteria;p\_\_Bacteroidetes;Other;Other;Other | 0 | 0.0% | 0.0% | 0.0% | 0.0% | 0.0% | 0.0% | 0.0% | 0.0% | 0.0% | 0.0% | 0.0% |
|  | k\_\_Bacteria;p\_\_Bacteroidetes;c\_\_Bacteroidia;o\_\_Bacteroidales;Other | 0 | 0.0% | 0.1% | 0.0% | 0.0% | 0.0% | 0.0% | 0.0% | 0.0% | 0.0% | 0.0% | 0.0% |
|  | k\_\_Bacteria;p\_\_Bacteroidetes;c\_\_Bacteroidia;o\_\_Bacteroidales;f\_\_Porphyromonadaceae | 0 | 0.2% | 1.3% | 0.0% | 0.0% | 0.0% | 0.1% | 0.0% | 0.0% | 0.1% | 0.0% | 0.0% |
|  | k\_\_Bacteria;p\_\_Bacteroidetes;c\_\_Bacteroidia;o\_\_Bacteroidales;f\_\_Prevotellaceae | 0 | 0.1% | 0.1% | 0.2% | 0.0% | 0.1% | 0.0% | 0.1% | 0.1% | 0.2% | 0.1% | 0.1% |
|  | k\_\_Bacteria;p\_\_Bacteroidetes;c\_\_Bacteroidia;o\_\_Bacteroidales;f\_\_[Paraprevotellaceae] | 0 | 0.0% | 0.0% | 0.0% | 0.0% | 0.0% | 0.0% | 0.0% | 0.0% | 0.0% | 0.0% | 0.0% |
|  | k\_\_Bacteria;p\_\_Bacteroidetes;c\_\_Cytophagia;o\_\_Cytophagales;Other | 0 | 0.0% | 0.0% | 0.0% | 0.0% | 0.0% | 0.0% | 0.0% | 0.0% | 0.0% | 0.0% | 0.0% |
|  | k\_\_Bacteria;p\_\_Bacteroidetes;c\_\_Cytophagia;o\_\_Cytophagales;f\_\_Cytophagaceae | 0 | 0.0% | 0.0% | 0.0% | 0.0% | 0.1% | 0.0% | 0.0% | 0.0% | 0.0% | 0.0% | 0.0% |
|  | k\_\_Bacteria;p\_\_Bacteroidetes;c\_\_Flavobacteriia;o\_\_Flavobacteriales;f\_\_[Weeksellaceae] | 0 | 0.0% | 0.0% | 0.0% | 0.0% | 0.0% | 0.0% | 0.0% | 0.0% | 0.1% | 0.0% | 0.0% |
|  | k\_\_Bacteria;p\_\_Cyanobacteria;Other;Other;Other | 0 | 0.0% | 0.0% | 0.0% | 0.0% | 0.0% | 0.0% | 0.0% | 0.0% | 0.0% | 0.0% | 0.0% |
|  | k\_\_Bacteria;p\_\_Cyanobacteria;c\_\_4C0d-2;o\_\_YS2;f\_\_ | 0 | 0.0% | 0.0% | 0.0% | 0.0% | 0.0% | 0.0% | 0.0% | 0.0% | 0.0% | 0.0% | 0.0% |
|  | k\_\_Bacteria;p\_\_Cyanobacteria;c\_\_Synechococcophycideae;o\_\_Synechococcales;f\_\_Synechococcaceae | 0 | 0.0% | 0.0% | 0.0% | 0.0% | 0.0% | 0.0% | 0.0% | 0.0% | 0.0% | 0.0% | 0.0% |
|  | k\_\_Bacteria;p\_\_Firmicutes;Other;Other;Other | 0 | 0.0% | 0.0% | 0.0% | 0.0% | 0.0% | 0.0% | 0.0% | 0.1% | 0.0% | 0.0% | 0.0% |
|  | k\_\_Bacteria;p\_\_Firmicutes;c\_\_Bacilli;Other;Other | 0 | 1.2% | 1.4% | 2.3% | 1.1% | 1.1% | 1.8% | 0.9% | 0.7% | 0.8% | 0.6% | 1.5% |
|  | k\_\_Bacteria;p\_\_Firmicutes;c\_\_Bacilli;o\_\_Bacillales;Other | 0 | 0.0% | 0.0% | 0.0% | 0.0% | 0.0% | 0.0% | 0.0% | 0.0% | 0.0% | 0.0% | 0.0% |
|  | k\_\_Bacteria;p\_\_Firmicutes;c\_\_Bacilli;o\_\_Bacillales;f\_\_Bacillaceae | 0 | 0.0% | 0.0% | 0.0% | 0.0% | 0.0% | 0.0% | 0.0% | 0.0% | 0.0% | 0.0% | 0.0% |
|  | k\_\_Bacteria;p\_\_Firmicutes;c\_\_Bacilli;o\_\_Bacillales;f\_\_Paenibacillaceae | 0 | 0.0% | 0.0% | 0.0% | 0.0% | 0.0% | 0.0% | 0.0% | 0.0% | 0.0% | 0.0% | 0.0% |
|  | k\_\_Bacteria;p\_\_Firmicutes;c\_\_Bacilli;o\_\_Bacillales;f\_\_Planococcaceae | 0 | 0.0% | 0.0% | 0.0% | 0.0% | 0.0% | 0.0% | 0.0% | 0.0% | 0.0% | 0.0% | 0.0% |
|  | k\_\_Bacteria;p\_\_Firmicutes;c\_\_Bacilli;o\_\_Bacillales;f\_\_Staphylococcaceae | 1 | 7.4% | 12.0% | 18.6% | 6.8% | 4.0% | 10.3% | 3.0% | 2.0% | 3.0% | 3.8% | 10.5% |
|  | k\_\_Bacteria;p\_\_Firmicutes;c\_\_Bacilli;o\_\_Gemellales;f\_\_ | 0 | 0.1% | 0.1% | 0.1% | 0.1% | 0.1% | 0.1% | 0.0% | 0.0% | 0.0% | 0.0% | 0.3% |
|  | k\_\_Bacteria;p\_\_Firmicutes;c\_\_Bacilli;o\_\_Gemellales;f\_\_Gemellaceae | 0 | 0.0% | 0.1% | 0.0% | 0.1% | 0.0% | 0.0% | 0.0% | 0.0% | 0.1% | 0.0% | 0.0% |
|  | k\_\_Bacteria;p\_\_Firmicutes;c\_\_Bacilli;o\_\_Lactobacillales;Other | 0 | 0.0% | 0.0% | 0.0% | 0.0% | 0.0% | 0.0% | 0.0% | 0.0% | 0.0% | 0.0% | 0.0% |
|  | k\_\_Bacteria;p\_\_Firmicutes;c\_\_Bacilli;o\_\_Lactobacillales;f\_\_ | 0 | 0.0% | 0.0% | 0.0% | 0.0% | 0.0% | 0.0% | 0.0% | 0.0% | 0.0% | 0.0% | 0.0% |
|  | k\_\_Bacteria;p\_\_Firmicutes;c\_\_Bacilli;o\_\_Lactobacillales;f\_\_Aerococcaceae | 3 | 30.9% | 9.2% | 12.5% | 41.3% | 37.7% | 29.0% | 32.9% | 44.8% | 27.8% | 44.8% | 28.8% |
|  | k\_\_Bacteria;p\_\_Firmicutes;c\_\_Bacilli;o\_\_Lactobacillales;f\_\_Carnobacteriaceae | 0 | 0.2% | 0.8% | 0.0% | 0.4% | 0.1% | 0.1% | 0.1% | 0.0% | 0.1% | 0.4% | 0.1% |
|  | k\_\_Bacteria;p\_\_Firmicutes;c\_\_Bacilli;o\_\_Lactobacillales;f\_\_Enterococcaceae | 0 | 0.0% | 0.0% | 0.0% | 0.0% | 0.0% | 0.0% | 0.0% | 0.0% | 0.0% | 0.0% | 0.0% |
|  | k\_\_Bacteria;p\_\_Firmicutes;c\_\_Bacilli;o\_\_Lactobacillales;f\_\_Lactobacillaceae | 0 | 0.1% | 0.0% | 0.1% | 0.1% | 0.1% | 0.1% | 0.1% | 0.1% | 0.0% | 0.0% | 0.1% |
|  | k\_\_Bacteria;p\_\_Firmicutes;c\_\_Bacilli;o\_\_Lactobacillales;f\_\_Streptococcaceae | 0 | 4.6% | 21.1% | 7.7% | 1.9% | 2.7% | 2.7% | 0.8% | 0.2% | 1.3% | 5.5% | 1.8% |
|  | k\_\_Bacteria;p\_\_Firmicutes;c\_\_Bacilli;o\_\_Turicibacterales;f\_\_Turicibacteraceae | 0 | 0.0% | 0.0% | 0.0% | 0.0% | 0.0% | 0.0% | 0.0% | 0.1% | 0.0% | 0.0% | 0.0% |
|  | k\_\_Bacteria;p\_\_Firmicutes;c\_\_Clostridia;o\_\_Clostridiales;Other | 0 | 0.1% | 0.0% | 0.1% | 0.0% | 0.1% | 0.1% | 0.1% | 0.3% | 0.6% | 0.1% | 0.0% |
|  | k\_\_Bacteria;p\_\_Firmicutes;c\_\_Clostridia;o\_\_Clostridiales;f\_\_ | 0 | 0.0% | 0.0% | 0.0% | 0.0% | 0.0% | 0.0% | 0.1% | 0.1% | 0.1% | 0.0% | 0.0% |
|  | k\_\_Bacteria;p\_\_Firmicutes;c\_\_Clostridia;o\_\_Clostridiales;f\_\_Christensenellaceae | 0 | 0.0% | 0.0% | 0.0% | 0.0% | 0.0% | 0.0% | 0.0% | 0.0% | 0.1% | 0.0% | 0.0% |
|  | k\_\_Bacteria;p\_\_Firmicutes;c\_\_Clostridia;o\_\_Clostridiales;f\_\_Clostridiaceae | 0 | 0.0% | 0.0% | 0.0% | 0.0% | 0.0% | 0.0% | 0.2% | 0.1% | 0.0% | 0.0% | 0.0% |
|  | k\_\_Bacteria;p\_\_Firmicutes;c\_\_Clostridia;o\_\_Clostridiales;f\_\_Lachnospiraceae | 0 | 0.1% | 0.0% | 0.1% | 0.1% | 0.3% | 0.0% | 0.2% | 0.2% | 0.3% | 0.1% | 0.0% |
|  | k\_\_Bacteria;p\_\_Firmicutes;c\_\_Clostridia;o\_\_Clostridiales;f\_\_Peptostreptococcaceae | 0 | 0.0% | 0.0% | 0.0% | 0.0% | 0.0% | 0.0% | 0.0% | 0.0% | 0.0% | 0.0% | 0.0% |
|  | k\_\_Bacteria;p\_\_Firmicutes;c\_\_Clostridia;o\_\_Clostridiales;f\_\_Ruminococcaceae | 0 | 0.1% | 0.0% | 0.0% | 0.1% | 0.3% | 0.0% | 0.2% | 0.2% | 0.3% | 0.1% | 0.0% |
|  | k\_\_Bacteria;p\_\_Firmicutes;c\_\_Clostridia;o\_\_Clostridiales;f\_\_Veillonellaceae | 0 | 0.2% | 0.9% | 0.0% | 0.1% | 0.1% | 0.2% | 0.1% | 0.1% | 0.3% | 0.0% | 0.1% |
|  | k\_\_Bacteria;p\_\_Firmicutes;c\_\_Clostridia;o\_\_Clostridiales;f\_\_[Tissierellaceae] | 0 | 2.2% | 1.9% | 2.8% | 1.2% | 1.4% | 4.3% | 2.0% | 1.4% | 0.4% | 0.7% | 5.8% |
|  | k\_\_Bacteria;p\_\_Firmicutes;c\_\_Erysipelotrichi;o\_\_Erysipelotrichales;f\_\_Erysipelotrichaceae | 0 | 0.0% | 0.0% | 0.0% | 0.0% | 0.0% | 0.0% | 0.0% | 0.0% | 0.1% | 0.0% | 0.0% |
|  | k\_\_Bacteria;p\_\_Fusobacteria;c\_\_Fusobacteriia;o\_\_Fusobacteriales;f\_\_Fusobacteriaceae | 0 | 0.1% | 0.4% | 0.0% | 0.0% | 0.0% | 0.0% | 0.0% | 0.0% | 0.0% | 0.0% | 0.1% |
|  | k\_\_Bacteria;p\_\_Fusobacteria;c\_\_Fusobacteriia;o\_\_Fusobacteriales;f\_\_Leptotrichiaceae | 0 | 0.0% | 0.1% | 0.0% | 0.0% | 0.0% | 0.0% | 0.0% | 0.0% | 0.0% | 0.0% | 0.0% |
|  | k\_\_Bacteria;p\_\_Lentisphaerae;c\_\_[Lentisphaeria];o\_\_Victivallales;f\_\_Victivallaceae | 0 | 0.0% | 0.0% | 0.0% | 0.0% | 0.0% | 0.0% | 0.0% | 0.0% | 0.0% | 0.0% | 0.0% |
|  | k\_\_Bacteria;p\_\_Proteobacteria;Other;Other;Other | 0 | 0.1% | 0.0% | 0.1% | 0.1% | 0.0% | 0.0% | 0.1% | 0.0% | 0.1% | 0.0% | 0.0% |
|  | k\_\_Bacteria;p\_\_Proteobacteria;c\_\_Alphaproteobacteria;Other;Other | 0 | 0.4% | 0.1% | 0.3% | 0.3% | 0.3% | 0.4% | 0.8% | 0.6% | 0.7% | 0.4% | 0.3% |
|  | k\_\_Bacteria;p\_\_Proteobacteria;c\_\_Alphaproteobacteria;o\_\_;f\_\_ | 0 | 0.0% | 0.0% | 0.0% | 0.0% | 0.0% | 0.0% | 0.0% | 0.0% | 0.0% | 0.0% | 0.0% |
|  | k\_\_Bacteria;p\_\_Proteobacteria;c\_\_Alphaproteobacteria;o\_\_BD7-3;f\_\_ | 0 | 3.1% | 0.7% | 1.9% | 1.8% | 2.5% | 2.3% | 6.3% | 4.3% | 5.6% | 2.3% | 3.0% |
|  | k\_\_Bacteria;p\_\_Proteobacteria;c\_\_Alphaproteobacteria;o\_\_Caulobacterales;f\_\_Caulobacteraceae | 0 | 0.0% | 0.0% | 0.0% | 0.0% | 0.1% | 0.0% | 0.0% | 0.0% | 0.0% | 0.0% | 0.0% |
|  | k\_\_Bacteria;p\_\_Proteobacteria;c\_\_Alphaproteobacteria;o\_\_RF32;f\_\_ | 0 | 0.0% | 0.0% | 0.0% | 0.0% | 0.0% | 0.0% | 0.0% | 0.0% | 0.0% | 0.0% | 0.0% |
|  | k\_\_Bacteria;p\_\_Proteobacteria;c\_\_Alphaproteobacteria;o\_\_Rhizobiales;Other | 0 | 0.0% | 0.0% | 0.0% | 0.0% | 0.0% | 0.0% | 0.0% | 0.0% | 0.0% | 0.0% | 0.0% |
|  | k\_\_Bacteria;p\_\_Proteobacteria;c\_\_Alphaproteobacteria;o\_\_Rhizobiales;f\_\_Beijerinckiaceae | 0 | 0.0% | 0.0% | 0.0% | 0.0% | 0.0% | 0.0% | 0.0% | 0.0% | 0.0% | 0.0% | 0.0% |
|  | k\_\_Bacteria;p\_\_Proteobacteria;c\_\_Alphaproteobacteria;o\_\_Rhizobiales;f\_\_Bradyrhizobiaceae | 0 | 0.0% | 0.0% | 0.0% | 0.1% | 0.0% | 0.0% | 0.0% | 0.0% | 0.0% | 0.0% | 0.0% |
|  | k\_\_Bacteria;p\_\_Proteobacteria;c\_\_Alphaproteobacteria;o\_\_Rhizobiales;f\_\_Hyphomicrobiaceae | 0 | 0.0% | 0.0% | 0.0% | 0.0% | 0.0% | 0.0% | 0.0% | 0.0% | 0.0% | 0.0% | 0.0% |
|  | k\_\_Bacteria;p\_\_Proteobacteria;c\_\_Alphaproteobacteria;o\_\_Rhizobiales;f\_\_Methylobacteriaceae | 0 | 0.0% | 0.0% | 0.0% | 0.0% | 0.0% | 0.0% | 0.1% | 0.0% | 0.0% | 0.0% | 0.0% |
|  | k\_\_Bacteria;p\_\_Proteobacteria;c\_\_Alphaproteobacteria;o\_\_Rhizobiales;f\_\_Methylocystaceae | 0 | 0.0% | 0.0% | 0.0% | 0.0% | 0.1% | 0.0% | 0.1% | 0.0% | 0.0% | 0.0% | 0.0% |
|  | k\_\_Bacteria;p\_\_Proteobacteria;c\_\_Alphaproteobacteria;o\_\_Rhizobiales;f\_\_Rhizobiaceae | 0 | 0.0% | 0.0% | 0.0% | 0.0% | 0.0% | 0.0% | 0.0% | 0.0% | 0.1% | 0.0% | 0.0% |
|  | k\_\_Bacteria;p\_\_Proteobacteria;c\_\_Alphaproteobacteria;o\_\_Rhodospirillales;f\_\_ | 0 | 0.0% | 0.0% | 0.0% | 0.0% | 0.0% | 0.0% | 0.0% | 0.0% | 0.0% | 0.0% | 0.0% |
|  | k\_\_Bacteria;p\_\_Proteobacteria;c\_\_Alphaproteobacteria;o\_\_Rhodospirillales;f\_\_Acetobacteraceae | 0 | 0.0% | 0.0% | 0.0% | 0.0% | 0.0% | 0.0% | 0.1% | 0.0% | 0.0% | 0.0% | 0.0% |
|  | k\_\_Bacteria;p\_\_Proteobacteria;c\_\_Alphaproteobacteria;o\_\_Rickettsiales;Other | 0 | 0.0% | 0.0% | 0.0% | 0.0% | 0.0% | 0.0% | 0.0% | 0.0% | 0.1% | 0.0% | 0.0% |
|  | k\_\_Bacteria;p\_\_Proteobacteria;c\_\_Alphaproteobacteria;o\_\_Rickettsiales;f\_\_ | 0 | 0.0% | 0.0% | 0.0% | 0.0% | 0.0% | 0.0% | 0.0% | 0.0% | 0.0% | 0.0% | 0.0% |
|  | k\_\_Bacteria;p\_\_Proteobacteria;c\_\_Alphaproteobacteria;o\_\_Rickettsiales;f\_\_Rickettsiaceae | 0 | 0.3% | 0.0% | 0.2% | 0.1% | 0.3% | 0.1% | 0.3% | 0.4% | 1.1% | 0.0% | 0.1% |
|  | k\_\_Bacteria;p\_\_Proteobacteria;c\_\_Alphaproteobacteria;o\_\_Rickettsiales;f\_\_mitochondria | 0 | 0.1% | 0.0% | 0.0% | 0.0% | 0.2% | 0.1% | 0.4% | 0.1% | 0.0% | 0.0% | 0.0% |
|  | k\_\_Bacteria;p\_\_Proteobacteria;c\_\_Alphaproteobacteria;o\_\_Sphingomonadales;f\_\_Erythrobacteraceae | 0 | 0.1% | 0.0% | 0.1% | 0.0% | 0.2% | 0.1% | 0.2% | 0.0% | 0.0% | 0.0% | 0.0% |
|  | k\_\_Bacteria;p\_\_Proteobacteria;c\_\_Alphaproteobacteria;o\_\_Sphingomonadales;f\_\_Sphingomonadaceae | 0 | 0.1% | 0.1% | 0.0% | 0.1% | 0.2% | 0.1% | 0.3% | 0.0% | 0.3% | 0.1% | 0.1% |
|  | k\_\_Bacteria;p\_\_Proteobacteria;c\_\_Betaproteobacteria;Other;Other | 0 | 0.0% | 0.0% | 0.0% | 0.0% | 0.0% | 0.0% | 0.0% | 0.0% | 0.0% | 0.0% | 0.0% |
|  | k\_\_Bacteria;p\_\_Proteobacteria;c\_\_Betaproteobacteria;o\_\_Burkholderiales;Other | 0 | 0.0% | 0.0% | 0.0% | 0.0% | 0.0% | 0.0% | 0.0% | 0.0% | 0.0% | 0.0% | 0.0% |
|  | k\_\_Bacteria;p\_\_Proteobacteria;c\_\_Betaproteobacteria;o\_\_Burkholderiales;f\_\_Burkholderiaceae | 0 | 0.0% | 0.0% | 0.0% | 0.0% | 0.0% | 0.0% | 0.0% | 0.0% | 0.0% | 0.0% | 0.0% |
|  | k\_\_Bacteria;p\_\_Proteobacteria;c\_\_Betaproteobacteria;o\_\_Burkholderiales;f\_\_Comamonadaceae | 0 | 0.5% | 0.7% | 0.2% | 0.2% | 0.5% | 0.1% | 0.8% | 0.2% | 1.4% | 0.6% | 0.2% |
|  | k\_\_Bacteria;p\_\_Proteobacteria;c\_\_Betaproteobacteria;o\_\_Burkholderiales;f\_\_Oxalobacteraceae | 0 | 0.0% | 0.0% | 0.0% | 0.0% | 0.1% | 0.0% | 0.0% | 0.0% | 0.0% | 0.0% | 0.0% |
|  | k\_\_Bacteria;p\_\_Proteobacteria;c\_\_Betaproteobacteria;o\_\_Neisseriales;f\_\_Neisseriaceae | 0 | 0.4% | 0.8% | 0.1% | 0.1% | 0.2% | 0.1% | 0.1% | 1.1% | 0.7% | 0.2% | 0.3% |
|  | k\_\_Bacteria;p\_\_Proteobacteria;c\_\_Betaproteobacteria;o\_\_Rhodocyclales;f\_\_Rhodocyclaceae | 0 | 0.1% | 0.2% | 0.0% | 0.0% | 0.1% | 0.0% | 0.1% | 0.0% | 0.2% | 0.0% | 0.0% |
|  | k\_\_Bacteria;p\_\_Proteobacteria;c\_\_Deltaproteobacteria;o\_\_Bdellovibrionales;f\_\_Bdellovibrionaceae | 0 | 0.0% | 0.0% | 0.0% | 0.0% | 0.0% | 0.0% | 0.0% | 0.0% | 0.0% | 0.0% | 0.0% |
|  | k\_\_Bacteria;p\_\_Proteobacteria;c\_\_Epsilonproteobacteria;o\_\_Campylobacterales;f\_\_Campylobacteraceae | 0 | 0.0% | 0.0% | 0.1% | 0.0% | 0.0% | 0.0% | 0.0% | 0.0% | 0.0% | 0.0% | 0.0% |
|  | k\_\_Bacteria;p\_\_Proteobacteria;c\_\_Epsilonproteobacteria;o\_\_Campylobacterales;f\_\_Helicobacteraceae | 0 | 0.0% | 0.0% | 0.0% | 0.0% | 0.0% | 0.0% | 0.0% | 0.0% | 0.0% | 0.0% | 0.0% |
|  | k\_\_Bacteria;p\_\_Proteobacteria;c\_\_Gammaproteobacteria;Other;Other | 0 | 0.0% | 0.0% | 0.0% | 0.0% | 0.0% | 0.0% | 0.0% | 0.0% | 0.0% | 0.0% | 0.0% |
|  | k\_\_Bacteria;p\_\_Proteobacteria;c\_\_Gammaproteobacteria;o\_\_Aeromonadales;f\_\_Aeromonadaceae | 0 | 0.0% | 0.0% | 0.0% | 0.0% | 0.0% | 0.0% | 0.0% | 0.0% | 0.0% | 0.0% | 0.0% |
|  | k\_\_Bacteria;p\_\_Proteobacteria;c\_\_Gammaproteobacteria;o\_\_Aeromonadales;f\_\_Succinivibrionaceae | 0 | 0.0% | 0.0% | 0.0% | 0.0% | 0.0% | 0.0% | 0.0% | 0.0% | 0.0% | 0.0% | 0.0% |
|  | k\_\_Bacteria;p\_\_Proteobacteria;c\_\_Gammaproteobacteria;o\_\_Alteromonadales;f\_\_Shewanellaceae | 0 | 0.0% | 0.0% | 0.0% | 0.0% | 0.0% | 0.0% | 0.0% | 0.0% | 0.0% | 0.0% | 0.0% |
|  | k\_\_Bacteria;p\_\_Proteobacteria;c\_\_Gammaproteobacteria;o\_\_Enterobacteriales;f\_\_Enterobacteriaceae | 0 | 1.8% | 0.3% | 7.5% | 1.6% | 2.6% | 1.6% | 0.6% | 1.0% | 1.2% | 0.3% | 0.8% |
|  | k\_\_Bacteria;p\_\_Proteobacteria;c\_\_Gammaproteobacteria;o\_\_Pasteurellales;f\_\_Pasteurellaceae | 1 | 5.2% | 11.0% | 15.1% | 0.5% | 0.2% | 3.7% | 1.7% | 4.5% | 4.4% | 0.6% | 10.6% |
|  | k\_\_Bacteria;p\_\_Proteobacteria;c\_\_Gammaproteobacteria;o\_\_Pseudomonadales;f\_\_Moraxellaceae | 0 | 1.1% | 1.4% | 3.4% | 0.9% | 0.5% | 0.5% | 1.0% | 0.6% | 1.4% | 0.5% | 0.4% |
|  | k\_\_Bacteria;p\_\_Proteobacteria;c\_\_Gammaproteobacteria;o\_\_Pseudomonadales;f\_\_Pseudomonadaceae | 0 | 0.0% | 0.0% | 0.0% | 0.0% | 0.1% | 0.1% | 0.1% | 0.0% | 0.0% | 0.0% | 0.0% |
|  | k\_\_Bacteria;p\_\_Proteobacteria;c\_\_Gammaproteobacteria;o\_\_Vibrionales;Other | 0 | 0.0% | 0.0% | 0.0% | 0.0% | 0.0% | 0.0% | 0.0% | 0.0% | 0.0% | 0.0% | 0.0% |
|  | k\_\_Bacteria;p\_\_Proteobacteria;c\_\_Gammaproteobacteria;o\_\_Vibrionales;f\_\_Vibrionaceae | 0 | 0.3% | 0.0% | 0.1% | 0.0% | 0.0% | 0.0% | 0.1% | 0.0% | 2.8% | 0.0% | 0.0% |
|  | k\_\_Bacteria;p\_\_Proteobacteria;c\_\_Gammaproteobacteria;o\_\_Xanthomonadales;f\_\_Xanthomonadaceae | 0 | 0.0% | 0.0% | 0.0% | 0.1% | 0.2% | 0.0% | 0.0% | 0.0% | 0.0% | 0.0% | 0.0% |
|  | k\_\_Bacteria;p\_\_Spirochaetes;c\_\_[Brachyspirae];o\_\_[Brachyspirales];f\_\_Brachyspiraceae | 0 | 0.0% | 0.0% | 0.0% | 0.0% | 0.0% | 0.0% | 0.0% | 0.0% | 0.0% | 0.0% | 0.0% |
|  | k\_\_Bacteria;p\_\_Tenericutes;c\_\_Mollicutes;o\_\_Mycoplasmatales;f\_\_Mycoplasmataceae | 0 | 0.0% | 0.0% | 0.0% | 0.0% | 0.0% | 0.0% | 0.0% | 0.0% | 0.0% | 0.0% | 0.0% |
|  | k\_\_Bacteria;p\_\_Tenericutes;c\_\_Mollicutes;o\_\_RF39;f\_\_ | 0 | 0.0% | 0.0% | 0.0% | 0.0% | 0.0% | 0.0% | 0.0% | 0.0% | 0.1% | 0.0% | 0.0% |
|  | k\_\_Bacteria;p\_\_Verrucomicrobia;c\_\_Verruco-5;o\_\_WCHB1-41;f\_\_RFP12 | 0 | 0.1% | 0.0% | 0.0% | 0.0% | 0.0% | 0.0% | 0.0% | 0.5% | 0.6% | 0.1% | 0.0% |
|  | k\_\_Bacteria;p\_\_Verrucomicrobia;c\_\_Verrucomicrobiae;o\_\_Verrucomicrobiales;f\_\_Verrucomicrobiaceae | 0 | 0.0% | 0.0% | 0.0% | 0.0% | 0.0% | 0.0% | 0.0% | 0.0% | 0.0% | 0.0% | 0.0% |
|  | k\_\_Bacteria;p\_\_WPS-2;c\_\_;o\_\_;f\_\_ | 0 | 0.0% | 0.0% | 0.0% | 0.0% | 0.0% | 0.0% | 0.0% | 0.1% | 0.1% | 0.0% | 0.0% |
|  | k\_\_Bacteria;p\_\_[Thermi];c\_\_Deinococci;o\_\_Deinococcales;f\_\_Deinococcaceae | 0 | 0.0% | 0.0% | 0.0% | 0.0% | 0.0% | 0.0% | 0.0% | 0.0% | 0.0% | 0.0% | 0.0% |

|  |  |
| --- | --- |
|  | |
| Taxonomy Summary. Current Level: | |
| View Figure (.pdf)  View Legend (.pdf) |  |
|  |  |


View Table (.txt)

|  |  |  |  |  |  |  |  |  |  |  |  |  |  |
| --- | --- | --- | --- | --- | --- | --- | --- | --- | --- | --- | --- | --- | --- |
|  | | Total | | 1 | 2 | 3 | 4 | 5 | 6 | 7 | 8 | 9 | 10 |
| Legend | Taxonomy | count | % | % | % | % | % | % | % | % | % | % | % |
|  | Unclassified;Other;Other;Other;Other;Other | 0 | 0.8% | 0.3% | 0.3% | 1.3% | 1.1% | 0.4% | 1.1% | 0.9% | 1.1% | 0.8% | 0.7% |
|  | k\_\_Bacteria;Other;Other;Other;Other;Other | 2 | 18.1% | 7.9% | 12.5% | 19.7% | 24.4% | 11.9% | 24.0% | 19.0% | 20.8% | 24.6% | 16.6% |
|  | k\_\_Bacteria;p\_\_Actinobacteria;Other;Other;Other;Other | 0 | 0.0% | 0.0% | 0.0% | 0.0% | 0.0% | 0.0% | 0.0% | 0.0% | 0.0% | 0.0% | 0.0% |
|  | k\_\_Bacteria;p\_\_Actinobacteria;c\_\_Actinobacteria;Other;Other;Other | 0 | 0.0% | 0.0% | 0.0% | 0.0% | 0.0% | 0.0% | 0.0% | 0.0% | 0.0% | 0.0% | 0.0% |
|  | k\_\_Bacteria;p\_\_Actinobacteria;c\_\_Actinobacteria;o\_\_Actinomycetales;Other;Other | 0 | 0.4% | 0.6% | 0.2% | 0.5% | 0.2% | 0.4% | 0.4% | 0.4% | 0.5% | 0.2% | 0.4% |
|  | k\_\_Bacteria;p\_\_Actinobacteria;c\_\_Actinobacteria;o\_\_Actinomycetales;f\_\_Actinomycetaceae;g\_\_ | 0 | 0.0% | 0.0% | 0.0% | 0.0% | 0.0% | 0.0% | 0.0% | 0.0% | 0.0% | 0.0% | 0.0% |
|  | k\_\_Bacteria;p\_\_Actinobacteria;c\_\_Actinobacteria;o\_\_Actinomycetales;f\_\_Actinomycetaceae;g\_\_Actinomyces | 0 | 0.0% | 0.0% | 0.0% | 0.0% | 0.0% | 0.1% | 0.0% | 0.0% | 0.0% | 0.1% | 0.0% |
|  | k\_\_Bacteria;p\_\_Actinobacteria;c\_\_Actinobacteria;o\_\_Actinomycetales;f\_\_Corynebacteriaceae;g\_\_Corynebacterium | 1 | 14.9% | 21.8% | 10.2% | 13.7% | 13.5% | 23.7% | 15.2% | 11.4% | 15.5% | 9.4% | 14.3% |
|  | k\_\_Bacteria;p\_\_Actinobacteria;c\_\_Actinobacteria;o\_\_Actinomycetales;f\_\_Dermabacteraceae;g\_\_Brachybacterium | 0 | 0.0% | 0.0% | 0.0% | 0.0% | 0.0% | 0.0% | 0.0% | 0.0% | 0.0% | 0.0% | 0.0% |
|  | k\_\_Bacteria;p\_\_Actinobacteria;c\_\_Actinobacteria;o\_\_Actinomycetales;f\_\_Dermabacteraceae;g\_\_Dermabacter | 0 | 0.0% | 0.0% | 0.0% | 0.0% | 0.1% | 0.1% | 0.0% | 0.0% | 0.0% | 0.0% | 0.0% |
|  | k\_\_Bacteria;p\_\_Actinobacteria;c\_\_Actinobacteria;o\_\_Actinomycetales;f\_\_Dietziaceae;Other | 0 | 0.0% | 0.0% | 0.0% | 0.0% | 0.0% | 0.0% | 0.0% | 0.0% | 0.0% | 0.0% | 0.0% |
|  | k\_\_Bacteria;p\_\_Actinobacteria;c\_\_Actinobacteria;o\_\_Actinomycetales;f\_\_Geodermatophilaceae;Other | 0 | 0.0% | 0.0% | 0.0% | 0.0% | 0.0% | 0.0% | 0.0% | 0.0% | 0.0% | 0.0% | 0.0% |
|  | k\_\_Bacteria;p\_\_Actinobacteria;c\_\_Actinobacteria;o\_\_Actinomycetales;f\_\_Microbacteriaceae;Other | 0 | 0.1% | 0.0% | 0.0% | 0.2% | 0.1% | 0.1% | 0.2% | 0.2% | 0.2% | 0.1% | 0.2% |
|  | k\_\_Bacteria;p\_\_Actinobacteria;c\_\_Actinobacteria;o\_\_Actinomycetales;f\_\_Microbacteriaceae;g\_\_ | 0 | 0.0% | 0.0% | 0.0% | 0.0% | 0.0% | 0.0% | 0.0% | 0.0% | 0.0% | 0.0% | 0.0% |
|  | k\_\_Bacteria;p\_\_Actinobacteria;c\_\_Actinobacteria;o\_\_Actinomycetales;f\_\_Microbacteriaceae;g\_\_Agromyces | 0 | 0.0% | 0.0% | 0.0% | 0.0% | 0.0% | 0.0% | 0.0% | 0.0% | 0.0% | 0.0% | 0.0% |
|  | k\_\_Bacteria;p\_\_Actinobacteria;c\_\_Actinobacteria;o\_\_Actinomycetales;f\_\_Microbacteriaceae;g\_\_Curtobacterium | 0 | 0.0% | 0.0% | 0.0% | 0.0% | 0.1% | 0.0% | 0.0% | 0.0% | 0.0% | 0.0% | 0.0% |
|  | k\_\_Bacteria;p\_\_Actinobacteria;c\_\_Actinobacteria;o\_\_Actinomycetales;f\_\_Microbacteriaceae;g\_\_Herbiconiux | 0 | 0.0% | 0.0% | 0.0% | 0.0% | 0.0% | 0.0% | 0.0% | 0.0% | 0.0% | 0.0% | 0.0% |
|  | k\_\_Bacteria;p\_\_Actinobacteria;c\_\_Actinobacteria;o\_\_Actinomycetales;f\_\_Microbacteriaceae;g\_\_Microbacterium | 0 | 0.0% | 0.0% | 0.0% | 0.0% | 0.1% | 0.0% | 0.0% | 0.0% | 0.0% | 0.0% | 0.0% |
|  | k\_\_Bacteria;p\_\_Actinobacteria;c\_\_Actinobacteria;o\_\_Actinomycetales;f\_\_Micrococcaceae;g\_\_Arthrobacter | 0 | 0.0% | 0.0% | 0.0% | 0.0% | 0.0% | 0.0% | 0.0% | 0.0% | 0.0% | 0.0% | 0.0% |
|  | k\_\_Bacteria;p\_\_Actinobacteria;c\_\_Actinobacteria;o\_\_Actinomycetales;f\_\_Micrococcaceae;g\_\_Kocuria | 0 | 0.0% | 0.0% | 0.0% | 0.0% | 0.0% | 0.0% | 0.0% | 0.0% | 0.0% | 0.0% | 0.0% |
|  | k\_\_Bacteria;p\_\_Actinobacteria;c\_\_Actinobacteria;o\_\_Actinomycetales;f\_\_Micrococcaceae;g\_\_Micrococcus | 0 | 0.0% | 0.0% | 0.0% | 0.0% | 0.0% | 0.0% | 0.0% | 0.0% | 0.0% | 0.0% | 0.0% |
|  | k\_\_Bacteria;p\_\_Actinobacteria;c\_\_Actinobacteria;o\_\_Actinomycetales;f\_\_Micrococcaceae;g\_\_Rothia | 0 | 0.0% | 0.1% | 0.0% | 0.1% | 0.0% | 0.0% | 0.0% | 0.0% | 0.0% | 0.0% | 0.0% |
|  | k\_\_Bacteria;p\_\_Actinobacteria;c\_\_Actinobacteria;o\_\_Actinomycetales;f\_\_Nocardioidaceae;Other | 0 | 0.0% | 0.0% | 0.0% | 0.0% | 0.0% | 0.0% | 0.0% | 0.0% | 0.0% | 0.0% | 0.0% |
|  | k\_\_Bacteria;p\_\_Actinobacteria;c\_\_Actinobacteria;o\_\_Actinomycetales;f\_\_Nocardioidaceae;g\_\_Friedmanniella | 0 | 0.0% | 0.0% | 0.0% | 0.0% | 0.0% | 0.0% | 0.0% | 0.0% | 0.0% | 0.0% | 0.0% |
|  | k\_\_Bacteria;p\_\_Actinobacteria;c\_\_Actinobacteria;o\_\_Actinomycetales;f\_\_Propionibacteriaceae;Other | 0 | 0.0% | 0.0% | 0.0% | 0.0% | 0.0% | 0.0% | 0.0% | 0.0% | 0.0% | 0.0% | 0.0% |
|  | k\_\_Bacteria;p\_\_Actinobacteria;c\_\_Actinobacteria;o\_\_Actinomycetales;f\_\_Propionibacteriaceae;g\_\_Propionibacterium | 0 | 3.4% | 4.0% | 3.0% | 4.7% | 2.9% | 4.9% | 4.3% | 1.6% | 3.0% | 2.6% | 2.5% |
|  | k\_\_Bacteria;p\_\_Actinobacteria;c\_\_Actinobacteria;o\_\_Bifidobacteriales;f\_\_Bifidobacteriaceae;Other | 0 | 0.0% | 0.0% | 0.0% | 0.0% | 0.0% | 0.0% | 0.0% | 0.0% | 0.0% | 0.0% | 0.0% |
|  | k\_\_Bacteria;p\_\_Actinobacteria;c\_\_Actinobacteria;o\_\_Bifidobacteriales;f\_\_Bifidobacteriaceae;g\_\_Bifidobacterium | 0 | 0.5% | 0.0% | 0.0% | 0.1% | 0.1% | 0.0% | 0.0% | 2.0% | 2.1% | 0.3% | 0.0% |
|  | k\_\_Bacteria;p\_\_Actinobacteria;c\_\_Coriobacteriia;o\_\_Coriobacteriales;f\_\_Coriobacteriaceae;g\_\_Atopobium | 0 | 0.0% | 0.0% | 0.0% | 0.0% | 0.0% | 0.0% | 0.0% | 0.0% | 0.0% | 0.0% | 0.0% |
|  | k\_\_Bacteria;p\_\_Bacteroidetes;Other;Other;Other;Other | 0 | 0.0% | 0.0% | 0.0% | 0.0% | 0.0% | 0.0% | 0.0% | 0.0% | 0.0% | 0.0% | 0.0% |
|  | k\_\_Bacteria;p\_\_Bacteroidetes;c\_\_Bacteroidia;o\_\_Bacteroidales;Other;Other | 0 | 0.0% | 0.1% | 0.0% | 0.0% | 0.0% | 0.0% | 0.0% | 0.0% | 0.0% | 0.0% | 0.0% |
|  | k\_\_Bacteria;p\_\_Bacteroidetes;c\_\_Bacteroidia;o\_\_Bacteroidales;f\_\_Porphyromonadaceae;g\_\_Porphyromonas | 0 | 0.2% | 1.3% | 0.0% | 0.0% | 0.0% | 0.1% | 0.0% | 0.0% | 0.1% | 0.0% | 0.0% |
|  | k\_\_Bacteria;p\_\_Bacteroidetes;c\_\_Bacteroidia;o\_\_Bacteroidales;f\_\_Prevotellaceae;g\_\_Prevotella | 0 | 0.1% | 0.1% | 0.2% | 0.0% | 0.1% | 0.0% | 0.1% | 0.1% | 0.2% | 0.1% | 0.1% |
|  | k\_\_Bacteria;p\_\_Bacteroidetes;c\_\_Bacteroidia;o\_\_Bacteroidales;f\_\_[Paraprevotellaceae];Other | 0 | 0.0% | 0.0% | 0.0% | 0.0% | 0.0% | 0.0% | 0.0% | 0.0% | 0.0% | 0.0% | 0.0% |
|  | k\_\_Bacteria;p\_\_Bacteroidetes;c\_\_Bacteroidia;o\_\_Bacteroidales;f\_\_[Paraprevotellaceae];g\_\_[Prevotella] | 0 | 0.0% | 0.0% | 0.0% | 0.0% | 0.0% | 0.0% | 0.0% | 0.0% | 0.0% | 0.0% | 0.0% |
|  | k\_\_Bacteria;p\_\_Bacteroidetes;c\_\_Cytophagia;o\_\_Cytophagales;Other;Other | 0 | 0.0% | 0.0% | 0.0% | 0.0% | 0.0% | 0.0% | 0.0% | 0.0% | 0.0% | 0.0% | 0.0% |
|  | k\_\_Bacteria;p\_\_Bacteroidetes;c\_\_Cytophagia;o\_\_Cytophagales;f\_\_Cytophagaceae;g\_\_Dyadobacter | 0 | 0.0% | 0.0% | 0.0% | 0.0% | 0.0% | 0.0% | 0.0% | 0.0% | 0.0% | 0.0% | 0.0% |
|  | k\_\_Bacteria;p\_\_Bacteroidetes;c\_\_Cytophagia;o\_\_Cytophagales;f\_\_Cytophagaceae;g\_\_Spirosoma | 0 | 0.0% | 0.0% | 0.0% | 0.0% | 0.0% | 0.0% | 0.0% | 0.0% | 0.0% | 0.0% | 0.0% |
|  | k\_\_Bacteria;p\_\_Bacteroidetes;c\_\_Flavobacteriia;o\_\_Flavobacteriales;f\_\_[Weeksellaceae];g\_\_Cloacibacterium | 0 | 0.0% | 0.0% | 0.0% | 0.0% | 0.0% | 0.0% | 0.0% | 0.0% | 0.1% | 0.0% | 0.0% |
|  | k\_\_Bacteria;p\_\_Cyanobacteria;Other;Other;Other;Other | 0 | 0.0% | 0.0% | 0.0% | 0.0% | 0.0% | 0.0% | 0.0% | 0.0% | 0.0% | 0.0% | 0.0% |
|  | k\_\_Bacteria;p\_\_Cyanobacteria;c\_\_4C0d-2;o\_\_YS2;f\_\_;g\_\_ | 0 | 0.0% | 0.0% | 0.0% | 0.0% | 0.0% | 0.0% | 0.0% | 0.0% | 0.0% | 0.0% | 0.0% |
|  | k\_\_Bacteria;p\_\_Cyanobacteria;c\_\_Synechococcophycideae;o\_\_Synechococcales;f\_\_Synechococcaceae;g\_\_Synechococcus | 0 | 0.0% | 0.0% | 0.0% | 0.0% | 0.0% | 0.0% | 0.0% | 0.0% | 0.0% | 0.0% | 0.0% |
|  | k\_\_Bacteria;p\_\_Firmicutes;Other;Other;Other;Other | 0 | 0.0% | 0.0% | 0.0% | 0.0% | 0.0% | 0.0% | 0.0% | 0.1% | 0.0% | 0.0% | 0.0% |
|  | k\_\_Bacteria;p\_\_Firmicutes;c\_\_Bacilli;Other;Other;Other | 0 | 1.2% | 1.4% | 2.3% | 1.1% | 1.1% | 1.8% | 0.9% | 0.7% | 0.8% | 0.6% | 1.5% |
|  | k\_\_Bacteria;p\_\_Firmicutes;c\_\_Bacilli;o\_\_Bacillales;Other;Other | 0 | 0.0% | 0.0% | 0.0% | 0.0% | 0.0% | 0.0% | 0.0% | 0.0% | 0.0% | 0.0% | 0.0% |
|  | k\_\_Bacteria;p\_\_Firmicutes;c\_\_Bacilli;o\_\_Bacillales;f\_\_Bacillaceae;g\_\_Anoxybacillus | 0 | 0.0% | 0.0% | 0.0% | 0.0% | 0.0% | 0.0% | 0.0% | 0.0% | 0.0% | 0.0% | 0.0% |
|  | k\_\_Bacteria;p\_\_Firmicutes;c\_\_Bacilli;o\_\_Bacillales;f\_\_Paenibacillaceae;g\_\_Paenibacillus | 0 | 0.0% | 0.0% | 0.0% | 0.0% | 0.0% | 0.0% | 0.0% | 0.0% | 0.0% | 0.0% | 0.0% |
|  | k\_\_Bacteria;p\_\_Firmicutes;c\_\_Bacilli;o\_\_Bacillales;f\_\_Planococcaceae;g\_\_Planomicrobium | 0 | 0.0% | 0.0% | 0.0% | 0.0% | 0.0% | 0.0% | 0.0% | 0.0% | 0.0% | 0.0% | 0.0% |
|  | k\_\_Bacteria;p\_\_Firmicutes;c\_\_Bacilli;o\_\_Bacillales;f\_\_Staphylococcaceae;g\_\_Staphylococcus | 1 | 7.4% | 12.0% | 18.6% | 6.8% | 4.0% | 10.3% | 3.0% | 2.0% | 3.0% | 3.8% | 10.5% |
|  | k\_\_Bacteria;p\_\_Firmicutes;c\_\_Bacilli;o\_\_Gemellales;f\_\_;g\_\_ | 0 | 0.1% | 0.1% | 0.1% | 0.1% | 0.1% | 0.1% | 0.0% | 0.0% | 0.0% | 0.0% | 0.3% |
|  | k\_\_Bacteria;p\_\_Firmicutes;c\_\_Bacilli;o\_\_Gemellales;f\_\_Gemellaceae;Other | 0 | 0.0% | 0.0% | 0.0% | 0.0% | 0.0% | 0.0% | 0.0% | 0.0% | 0.0% | 0.0% | 0.0% |
|  | k\_\_Bacteria;p\_\_Firmicutes;c\_\_Bacilli;o\_\_Gemellales;f\_\_Gemellaceae;g\_\_ | 0 | 0.0% | 0.1% | 0.0% | 0.0% | 0.0% | 0.0% | 0.0% | 0.0% | 0.1% | 0.0% | 0.0% |
|  | k\_\_Bacteria;p\_\_Firmicutes;c\_\_Bacilli;o\_\_Lactobacillales;Other;Other | 0 | 0.0% | 0.0% | 0.0% | 0.0% | 0.0% | 0.0% | 0.0% | 0.0% | 0.0% | 0.0% | 0.0% |
|  | k\_\_Bacteria;p\_\_Firmicutes;c\_\_Bacilli;o\_\_Lactobacillales;f\_\_;g\_\_ | 0 | 0.0% | 0.0% | 0.0% | 0.0% | 0.0% | 0.0% | 0.0% | 0.0% | 0.0% | 0.0% | 0.0% |
|  | k\_\_Bacteria;p\_\_Firmicutes;c\_\_Bacilli;o\_\_Lactobacillales;f\_\_Aerococcaceae;Other | 0 | 0.0% | 0.0% | 0.1% | 0.0% | 0.0% | 0.0% | 0.0% | 0.0% | 0.0% | 0.0% | 0.0% |
|  | k\_\_Bacteria;p\_\_Firmicutes;c\_\_Bacilli;o\_\_Lactobacillales;f\_\_Aerococcaceae;g\_\_ | 0 | 0.0% | 0.0% | 0.0% | 0.0% | 0.0% | 0.0% | 0.0% | 0.0% | 0.0% | 0.0% | 0.0% |
|  | k\_\_Bacteria;p\_\_Firmicutes;c\_\_Bacilli;o\_\_Lactobacillales;f\_\_Aerococcaceae;g\_\_Abiotrophia | 0 | 0.0% | 0.0% | 0.0% | 0.0% | 0.0% | 0.0% | 0.0% | 0.0% | 0.0% | 0.0% | 0.0% |
|  | k\_\_Bacteria;p\_\_Firmicutes;c\_\_Bacilli;o\_\_Lactobacillales;f\_\_Aerococcaceae;g\_\_Aerococcus | 0 | 0.0% | 0.0% | 0.0% | 0.0% | 0.0% | 0.0% | 0.0% | 0.0% | 0.0% | 0.0% | 0.0% |
|  | k\_\_Bacteria;p\_\_Firmicutes;c\_\_Bacilli;o\_\_Lactobacillales;f\_\_Aerococcaceae;g\_\_Alloiococcus | 3 | 30.8% | 9.2% | 12.4% | 41.2% | 37.6% | 29.0% | 32.9% | 44.8% | 27.7% | 44.7% | 28.7% |
|  | k\_\_Bacteria;p\_\_Firmicutes;c\_\_Bacilli;o\_\_Lactobacillales;f\_\_Aerococcaceae;g\_\_Facklamia | 0 | 0.0% | 0.0% | 0.0% | 0.0% | 0.0% | 0.0% | 0.0% | 0.0% | 0.0% | 0.0% | 0.0% |
|  | k\_\_Bacteria;p\_\_Firmicutes;c\_\_Bacilli;o\_\_Lactobacillales;f\_\_Carnobacteriaceae;g\_\_Granulicatella | 0 | 0.2% | 0.8% | 0.0% | 0.4% | 0.1% | 0.1% | 0.1% | 0.0% | 0.1% | 0.4% | 0.1% |
|  | k\_\_Bacteria;p\_\_Firmicutes;c\_\_Bacilli;o\_\_Lactobacillales;f\_\_Enterococcaceae;g\_\_Enterococcus | 0 | 0.0% | 0.0% | 0.0% | 0.0% | 0.0% | 0.0% | 0.0% | 0.0% | 0.0% | 0.0% | 0.0% |
|  | k\_\_Bacteria;p\_\_Firmicutes;c\_\_Bacilli;o\_\_Lactobacillales;f\_\_Lactobacillaceae;g\_\_Lactobacillus | 0 | 0.1% | 0.0% | 0.1% | 0.1% | 0.1% | 0.1% | 0.1% | 0.1% | 0.0% | 0.0% | 0.1% |
|  | k\_\_Bacteria;p\_\_Firmicutes;c\_\_Bacilli;o\_\_Lactobacillales;f\_\_Streptococcaceae;Other | 0 | 0.2% | 0.7% | 0.1% | 0.1% | 0.2% | 0.3% | 0.1% | 0.0% | 0.1% | 0.2% | 0.2% |
|  | k\_\_Bacteria;p\_\_Firmicutes;c\_\_Bacilli;o\_\_Lactobacillales;f\_\_Streptococcaceae;g\_\_ | 0 | 0.0% | 0.1% | 0.0% | 0.0% | 0.1% | 0.0% | 0.0% | 0.0% | 0.0% | 0.0% | 0.0% |
|  | k\_\_Bacteria;p\_\_Firmicutes;c\_\_Bacilli;o\_\_Lactobacillales;f\_\_Streptococcaceae;g\_\_Lactococcus | 0 | 0.8% | 0.0% | 7.5% | 0.0% | 0.0% | 0.0% | 0.0% | 0.0% | 0.0% | 0.0% | 0.0% |
|  | k\_\_Bacteria;p\_\_Firmicutes;c\_\_Bacilli;o\_\_Lactobacillales;f\_\_Streptococcaceae;g\_\_Streptococcus | 0 | 3.6% | 20.3% | 0.2% | 1.7% | 2.5% | 2.4% | 0.6% | 0.2% | 1.2% | 5.2% | 1.6% |
|  | k\_\_Bacteria;p\_\_Firmicutes;c\_\_Bacilli;o\_\_Turicibacterales;f\_\_Turicibacteraceae;g\_\_Turicibacter | 0 | 0.0% | 0.0% | 0.0% | 0.0% | 0.0% | 0.0% | 0.0% | 0.1% | 0.0% | 0.0% | 0.0% |
|  | k\_\_Bacteria;p\_\_Firmicutes;c\_\_Clostridia;o\_\_Clostridiales;Other;Other | 0 | 0.1% | 0.0% | 0.1% | 0.0% | 0.1% | 0.1% | 0.1% | 0.3% | 0.6% | 0.1% | 0.0% |
|  | k\_\_Bacteria;p\_\_Firmicutes;c\_\_Clostridia;o\_\_Clostridiales;f\_\_;g\_\_ | 0 | 0.0% | 0.0% | 0.0% | 0.0% | 0.0% | 0.0% | 0.1% | 0.1% | 0.1% | 0.0% | 0.0% |
|  | k\_\_Bacteria;p\_\_Firmicutes;c\_\_Clostridia;o\_\_Clostridiales;f\_\_Christensenellaceae;g\_\_ | 0 | 0.0% | 0.0% | 0.0% | 0.0% | 0.0% | 0.0% | 0.0% | 0.0% | 0.1% | 0.0% | 0.0% |
|  | k\_\_Bacteria;p\_\_Firmicutes;c\_\_Clostridia;o\_\_Clostridiales;f\_\_Clostridiaceae;g\_\_02d06 | 0 | 0.0% | 0.0% | 0.0% | 0.0% | 0.0% | 0.0% | 0.0% | 0.0% | 0.0% | 0.0% | 0.0% |
|  | k\_\_Bacteria;p\_\_Firmicutes;c\_\_Clostridia;o\_\_Clostridiales;f\_\_Clostridiaceae;g\_\_Clostridium | 0 | 0.0% | 0.0% | 0.0% | 0.0% | 0.0% | 0.0% | 0.0% | 0.0% | 0.0% | 0.0% | 0.0% |
|  | k\_\_Bacteria;p\_\_Firmicutes;c\_\_Clostridia;o\_\_Clostridiales;f\_\_Clostridiaceae;g\_\_SMB53 | 0 | 0.0% | 0.0% | 0.0% | 0.0% | 0.0% | 0.0% | 0.2% | 0.1% | 0.0% | 0.0% | 0.0% |
|  | k\_\_Bacteria;p\_\_Firmicutes;c\_\_Clostridia;o\_\_Clostridiales;f\_\_Lachnospiraceae;Other | 0 | 0.1% | 0.0% | 0.0% | 0.0% | 0.1% | 0.0% | 0.1% | 0.1% | 0.2% | 0.0% | 0.0% |
|  | k\_\_Bacteria;p\_\_Firmicutes;c\_\_Clostridia;o\_\_Clostridiales;f\_\_Lachnospiraceae;g\_\_ | 0 | 0.0% | 0.0% | 0.1% | 0.0% | 0.0% | 0.0% | 0.0% | 0.0% | 0.0% | 0.0% | 0.0% |
|  | k\_\_Bacteria;p\_\_Firmicutes;c\_\_Clostridia;o\_\_Clostridiales;f\_\_Lachnospiraceae;g\_\_Blautia | 0 | 0.0% | 0.0% | 0.0% | 0.1% | 0.2% | 0.0% | 0.1% | 0.0% | 0.1% | 0.0% | 0.0% |
|  | k\_\_Bacteria;p\_\_Firmicutes;c\_\_Clostridia;o\_\_Clostridiales;f\_\_Lachnospiraceae;g\_\_Coprococcus | 0 | 0.0% | 0.0% | 0.0% | 0.0% | 0.0% | 0.0% | 0.0% | 0.1% | 0.1% | 0.0% | 0.0% |
|  | k\_\_Bacteria;p\_\_Firmicutes;c\_\_Clostridia;o\_\_Clostridiales;f\_\_Lachnospiraceae;g\_\_Lachnobacterium | 0 | 0.0% | 0.0% | 0.0% | 0.0% | 0.0% | 0.0% | 0.0% | 0.0% | 0.0% | 0.0% | 0.0% |
|  | k\_\_Bacteria;p\_\_Firmicutes;c\_\_Clostridia;o\_\_Clostridiales;f\_\_Lachnospiraceae;g\_\_Oribacterium | 0 | 0.0% | 0.0% | 0.0% | 0.0% | 0.0% | 0.0% | 0.0% | 0.0% | 0.0% | 0.0% | 0.0% |
|  | k\_\_Bacteria;p\_\_Firmicutes;c\_\_Clostridia;o\_\_Clostridiales;f\_\_Peptostreptococcaceae;g\_\_Peptostreptococcus | 0 | 0.0% | 0.0% | 0.0% | 0.0% | 0.0% | 0.0% | 0.0% | 0.0% | 0.0% | 0.0% | 0.0% |
|  | k\_\_Bacteria;p\_\_Firmicutes;c\_\_Clostridia;o\_\_Clostridiales;f\_\_Ruminococcaceae;Other | 0 | 0.0% | 0.0% | 0.0% | 0.0% | 0.1% | 0.0% | 0.0% | 0.0% | 0.0% | 0.0% | 0.0% |
|  | k\_\_Bacteria;p\_\_Firmicutes;c\_\_Clostridia;o\_\_Clostridiales;f\_\_Ruminococcaceae;g\_\_ | 0 | 0.1% | 0.0% | 0.0% | 0.1% | 0.1% | 0.0% | 0.1% | 0.1% | 0.2% | 0.0% | 0.0% |
|  | k\_\_Bacteria;p\_\_Firmicutes;c\_\_Clostridia;o\_\_Clostridiales;f\_\_Ruminococcaceae;g\_\_Faecalibacterium | 0 | 0.0% | 0.0% | 0.0% | 0.0% | 0.1% | 0.0% | 0.0% | 0.0% | 0.1% | 0.0% | 0.0% |
|  | k\_\_Bacteria;p\_\_Firmicutes;c\_\_Clostridia;o\_\_Clostridiales;f\_\_Ruminococcaceae;g\_\_Oscillospira | 0 | 0.0% | 0.0% | 0.0% | 0.0% | 0.0% | 0.0% | 0.0% | 0.0% | 0.1% | 0.0% | 0.0% |
|  | k\_\_Bacteria;p\_\_Firmicutes;c\_\_Clostridia;o\_\_Clostridiales;f\_\_Ruminococcaceae;g\_\_Ruminococcus | 0 | 0.0% | 0.0% | 0.0% | 0.0% | 0.0% | 0.0% | 0.1% | 0.0% | 0.0% | 0.0% | 0.0% |
|  | k\_\_Bacteria;p\_\_Firmicutes;c\_\_Clostridia;o\_\_Clostridiales;f\_\_Veillonellaceae;Other | 0 | 0.0% | 0.0% | 0.0% | 0.0% | 0.0% | 0.0% | 0.0% | 0.0% | 0.0% | 0.0% | 0.0% |
|  | k\_\_Bacteria;p\_\_Firmicutes;c\_\_Clostridia;o\_\_Clostridiales;f\_\_Veillonellaceae;g\_\_Dialister | 0 | 0.0% | 0.0% | 0.0% | 0.0% | 0.0% | 0.0% | 0.0% | 0.0% | 0.0% | 0.0% | 0.0% |
|  | k\_\_Bacteria;p\_\_Firmicutes;c\_\_Clostridia;o\_\_Clostridiales;f\_\_Veillonellaceae;g\_\_Megasphaera | 0 | 0.0% | 0.0% | 0.0% | 0.0% | 0.0% | 0.0% | 0.0% | 0.1% | 0.0% | 0.0% | 0.0% |
|  | k\_\_Bacteria;p\_\_Firmicutes;c\_\_Clostridia;o\_\_Clostridiales;f\_\_Veillonellaceae;g\_\_Veillonella | 0 | 0.1% | 0.9% | 0.0% | 0.1% | 0.1% | 0.1% | 0.0% | 0.0% | 0.2% | 0.0% | 0.0% |
|  | k\_\_Bacteria;p\_\_Firmicutes;c\_\_Clostridia;o\_\_Clostridiales;f\_\_[Tissierellaceae];Other | 0 | 0.0% | 0.0% | 0.0% | 0.0% | 0.0% | 0.0% | 0.0% | 0.0% | 0.0% | 0.0% | 0.0% |
|  | k\_\_Bacteria;p\_\_Firmicutes;c\_\_Clostridia;o\_\_Clostridiales;f\_\_[Tissierellaceae];g\_\_Anaerococcus | 0 | 0.5% | 0.4% | 0.5% | 0.0% | 0.3% | 0.3% | 0.2% | 0.1% | 0.1% | 0.1% | 2.8% |
|  | k\_\_Bacteria;p\_\_Firmicutes;c\_\_Clostridia;o\_\_Clostridiales;f\_\_[Tissierellaceae];g\_\_Finegoldia | 0 | 1.2% | 1.0% | 1.5% | 0.7% | 0.7% | 2.6% | 1.6% | 1.1% | 0.2% | 0.4% | 1.9% |
|  | k\_\_Bacteria;p\_\_Firmicutes;c\_\_Clostridia;o\_\_Clostridiales;f\_\_[Tissierellaceae];g\_\_Parvimonas | 0 | 0.0% | 0.0% | 0.0% | 0.0% | 0.0% | 0.0% | 0.0% | 0.0% | 0.0% | 0.0% | 0.0% |
|  | k\_\_Bacteria;p\_\_Firmicutes;c\_\_Clostridia;o\_\_Clostridiales;f\_\_[Tissierellaceae];g\_\_Peptoniphilus | 0 | 0.5% | 0.5% | 0.8% | 0.4% | 0.3% | 1.4% | 0.3% | 0.2% | 0.1% | 0.2% | 1.1% |
|  | k\_\_Bacteria;p\_\_Firmicutes;c\_\_Erysipelotrichi;o\_\_Erysipelotrichales;f\_\_Erysipelotrichaceae;Other | 0 | 0.0% | 0.0% | 0.0% | 0.0% | 0.0% | 0.0% | 0.0% | 0.0% | 0.0% | 0.0% | 0.0% |
|  | k\_\_Bacteria;p\_\_Firmicutes;c\_\_Erysipelotrichi;o\_\_Erysipelotrichales;f\_\_Erysipelotrichaceae;g\_\_ | 0 | 0.0% | 0.0% | 0.0% | 0.0% | 0.0% | 0.0% | 0.0% | 0.0% | 0.0% | 0.0% | 0.0% |
|  | k\_\_Bacteria;p\_\_Firmicutes;c\_\_Erysipelotrichi;o\_\_Erysipelotrichales;f\_\_Erysipelotrichaceae;g\_\_Bulleidia | 0 | 0.0% | 0.0% | 0.0% | 0.0% | 0.0% | 0.0% | 0.0% | 0.0% | 0.0% | 0.0% | 0.0% |
|  | k\_\_Bacteria;p\_\_Firmicutes;c\_\_Erysipelotrichi;o\_\_Erysipelotrichales;f\_\_Erysipelotrichaceae;g\_\_RFN20 | 0 | 0.0% | 0.0% | 0.0% | 0.0% | 0.0% | 0.0% | 0.0% | 0.0% | 0.0% | 0.0% | 0.0% |
|  | k\_\_Bacteria;p\_\_Firmicutes;c\_\_Erysipelotrichi;o\_\_Erysipelotrichales;f\_\_Erysipelotrichaceae;g\_\_[Eubacterium] | 0 | 0.0% | 0.0% | 0.0% | 0.0% | 0.0% | 0.0% | 0.0% | 0.0% | 0.0% | 0.0% | 0.0% |
|  | k\_\_Bacteria;p\_\_Fusobacteria;c\_\_Fusobacteriia;o\_\_Fusobacteriales;f\_\_Fusobacteriaceae;g\_\_Fusobacterium | 0 | 0.1% | 0.4% | 0.0% | 0.0% | 0.0% | 0.0% | 0.0% | 0.0% | 0.0% | 0.0% | 0.1% |
|  | k\_\_Bacteria;p\_\_Fusobacteria;c\_\_Fusobacteriia;o\_\_Fusobacteriales;f\_\_Leptotrichiaceae;g\_\_ | 0 | 0.0% | 0.1% | 0.0% | 0.0% | 0.0% | 0.0% | 0.0% | 0.0% | 0.0% | 0.0% | 0.0% |
|  | k\_\_Bacteria;p\_\_Fusobacteria;c\_\_Fusobacteriia;o\_\_Fusobacteriales;f\_\_Leptotrichiaceae;g\_\_Leptotrichia | 0 | 0.0% | 0.1% | 0.0% | 0.0% | 0.0% | 0.0% | 0.0% | 0.0% | 0.0% | 0.0% | 0.0% |
|  | k\_\_Bacteria;p\_\_Lentisphaerae;c\_\_[Lentisphaeria];o\_\_Victivallales;f\_\_Victivallaceae;Other | 0 | 0.0% | 0.0% | 0.0% | 0.0% | 0.0% | 0.0% | 0.0% | 0.0% | 0.0% | 0.0% | 0.0% |
|  | k\_\_Bacteria;p\_\_Lentisphaerae;c\_\_[Lentisphaeria];o\_\_Victivallales;f\_\_Victivallaceae;g\_\_ | 0 | 0.0% | 0.0% | 0.0% | 0.0% | 0.0% | 0.0% | 0.0% | 0.0% | 0.0% | 0.0% | 0.0% |
|  | k\_\_Bacteria;p\_\_Proteobacteria;Other;Other;Other;Other | 0 | 0.1% | 0.0% | 0.1% | 0.1% | 0.0% | 0.0% | 0.1% | 0.0% | 0.1% | 0.0% | 0.0% |
|  | k\_\_Bacteria;p\_\_Proteobacteria;c\_\_Alphaproteobacteria;Other;Other;Other | 0 | 0.4% | 0.1% | 0.3% | 0.3% | 0.3% | 0.4% | 0.8% | 0.6% | 0.7% | 0.4% | 0.3% |
|  | k\_\_Bacteria;p\_\_Proteobacteria;c\_\_Alphaproteobacteria;o\_\_;f\_\_;g\_\_ | 0 | 0.0% | 0.0% | 0.0% | 0.0% | 0.0% | 0.0% | 0.0% | 0.0% | 0.0% | 0.0% | 0.0% |
|  | k\_\_Bacteria;p\_\_Proteobacteria;c\_\_Alphaproteobacteria;o\_\_BD7-3;f\_\_;g\_\_ | 0 | 3.1% | 0.7% | 1.9% | 1.8% | 2.5% | 2.3% | 6.3% | 4.3% | 5.6% | 2.3% | 3.0% |
|  | k\_\_Bacteria;p\_\_Proteobacteria;c\_\_Alphaproteobacteria;o\_\_Caulobacterales;f\_\_Caulobacteraceae;g\_\_Mycoplana | 0 | 0.0% | 0.0% | 0.0% | 0.0% | 0.1% | 0.0% | 0.0% | 0.0% | 0.0% | 0.0% | 0.0% |
|  | k\_\_Bacteria;p\_\_Proteobacteria;c\_\_Alphaproteobacteria;o\_\_RF32;f\_\_;g\_\_ | 0 | 0.0% | 0.0% | 0.0% | 0.0% | 0.0% | 0.0% | 0.0% | 0.0% | 0.0% | 0.0% | 0.0% |
|  | k\_\_Bacteria;p\_\_Proteobacteria;c\_\_Alphaproteobacteria;o\_\_Rhizobiales;Other;Other | 0 | 0.0% | 0.0% | 0.0% | 0.0% | 0.0% | 0.0% | 0.0% | 0.0% | 0.0% | 0.0% | 0.0% |
|  | k\_\_Bacteria;p\_\_Proteobacteria;c\_\_Alphaproteobacteria;o\_\_Rhizobiales;f\_\_Beijerinckiaceae;Other | 0 | 0.0% | 0.0% | 0.0% | 0.0% | 0.0% | 0.0% | 0.0% | 0.0% | 0.0% | 0.0% | 0.0% |
|  | k\_\_Bacteria;p\_\_Proteobacteria;c\_\_Alphaproteobacteria;o\_\_Rhizobiales;f\_\_Bradyrhizobiaceae;g\_\_Balneimonas | 0 | 0.0% | 0.0% | 0.0% | 0.0% | 0.0% | 0.0% | 0.0% | 0.0% | 0.0% | 0.0% | 0.0% |
|  | k\_\_Bacteria;p\_\_Proteobacteria;c\_\_Alphaproteobacteria;o\_\_Rhizobiales;f\_\_Bradyrhizobiaceae;g\_\_Bradyrhizobium | 0 | 0.0% | 0.0% | 0.0% | 0.1% | 0.0% | 0.0% | 0.0% | 0.0% | 0.0% | 0.0% | 0.0% |
|  | k\_\_Bacteria;p\_\_Proteobacteria;c\_\_Alphaproteobacteria;o\_\_Rhizobiales;f\_\_Hyphomicrobiaceae;g\_\_Devosia | 0 | 0.0% | 0.0% | 0.0% | 0.0% | 0.0% | 0.0% | 0.0% | 0.0% | 0.0% | 0.0% | 0.0% |
|  | k\_\_Bacteria;p\_\_Proteobacteria;c\_\_Alphaproteobacteria;o\_\_Rhizobiales;f\_\_Hyphomicrobiaceae;g\_\_Hyphomicrobium | 0 | 0.0% | 0.0% | 0.0% | 0.0% | 0.0% | 0.0% | 0.0% | 0.0% | 0.0% | 0.0% | 0.0% |
|  | k\_\_Bacteria;p\_\_Proteobacteria;c\_\_Alphaproteobacteria;o\_\_Rhizobiales;f\_\_Methylobacteriaceae;g\_\_ | 0 | 0.0% | 0.0% | 0.0% | 0.0% | 0.0% | 0.0% | 0.1% | 0.0% | 0.0% | 0.0% | 0.0% |
|  | k\_\_Bacteria;p\_\_Proteobacteria;c\_\_Alphaproteobacteria;o\_\_Rhizobiales;f\_\_Methylobacteriaceae;g\_\_Methylobacterium | 0 | 0.0% | 0.0% | 0.0% | 0.0% | 0.0% | 0.0% | 0.0% | 0.0% | 0.0% | 0.0% | 0.0% |
|  | k\_\_Bacteria;p\_\_Proteobacteria;c\_\_Alphaproteobacteria;o\_\_Rhizobiales;f\_\_Methylocystaceae;g\_\_ | 0 | 0.0% | 0.0% | 0.0% | 0.0% | 0.1% | 0.0% | 0.1% | 0.0% | 0.0% | 0.0% | 0.0% |
|  | k\_\_Bacteria;p\_\_Proteobacteria;c\_\_Alphaproteobacteria;o\_\_Rhizobiales;f\_\_Rhizobiaceae;Other | 0 | 0.0% | 0.0% | 0.0% | 0.0% | 0.0% | 0.0% | 0.0% | 0.0% | 0.0% | 0.0% | 0.0% |
|  | k\_\_Bacteria;p\_\_Proteobacteria;c\_\_Alphaproteobacteria;o\_\_Rhizobiales;f\_\_Rhizobiaceae;g\_\_Agrobacterium | 0 | 0.0% | 0.0% | 0.0% | 0.0% | 0.0% | 0.0% | 0.0% | 0.0% | 0.1% | 0.0% | 0.0% |
|  | k\_\_Bacteria;p\_\_Proteobacteria;c\_\_Alphaproteobacteria;o\_\_Rhodospirillales;f\_\_;g\_\_ | 0 | 0.0% | 0.0% | 0.0% | 0.0% | 0.0% | 0.0% | 0.0% | 0.0% | 0.0% | 0.0% | 0.0% |
|  | k\_\_Bacteria;p\_\_Proteobacteria;c\_\_Alphaproteobacteria;o\_\_Rhodospirillales;f\_\_Acetobacteraceae;Other | 0 | 0.0% | 0.0% | 0.0% | 0.0% | 0.0% | 0.0% | 0.0% | 0.0% | 0.0% | 0.0% | 0.0% |
|  | k\_\_Bacteria;p\_\_Proteobacteria;c\_\_Alphaproteobacteria;o\_\_Rhodospirillales;f\_\_Acetobacteraceae;g\_\_ | 0 | 0.0% | 0.0% | 0.0% | 0.0% | 0.0% | 0.0% | 0.0% | 0.0% | 0.0% | 0.0% | 0.0% |
|  | k\_\_Bacteria;p\_\_Proteobacteria;c\_\_Alphaproteobacteria;o\_\_Rhodospirillales;f\_\_Acetobacteraceae;g\_\_Acetobacter | 0 | 0.0% | 0.0% | 0.0% | 0.0% | 0.0% | 0.0% | 0.0% | 0.0% | 0.0% | 0.0% | 0.0% |
|  | k\_\_Bacteria;p\_\_Proteobacteria;c\_\_Alphaproteobacteria;o\_\_Rickettsiales;Other;Other | 0 | 0.0% | 0.0% | 0.0% | 0.0% | 0.0% | 0.0% | 0.0% | 0.0% | 0.1% | 0.0% | 0.0% |
|  | k\_\_Bacteria;p\_\_Proteobacteria;c\_\_Alphaproteobacteria;o\_\_Rickettsiales;f\_\_;g\_\_ | 0 | 0.0% | 0.0% | 0.0% | 0.0% | 0.0% | 0.0% | 0.0% | 0.0% | 0.0% | 0.0% | 0.0% |
|  | k\_\_Bacteria;p\_\_Proteobacteria;c\_\_Alphaproteobacteria;o\_\_Rickettsiales;f\_\_Rickettsiaceae;g\_\_ | 0 | 0.3% | 0.0% | 0.2% | 0.1% | 0.3% | 0.1% | 0.3% | 0.4% | 1.1% | 0.0% | 0.1% |
|  | k\_\_Bacteria;p\_\_Proteobacteria;c\_\_Alphaproteobacteria;o\_\_Rickettsiales;f\_\_mitochondria;Other | 0 | 0.0% | 0.0% | 0.0% | 0.0% | 0.1% | 0.1% | 0.2% | 0.0% | 0.0% | 0.0% | 0.0% |
|  | k\_\_Bacteria;p\_\_Proteobacteria;c\_\_Alphaproteobacteria;o\_\_Rickettsiales;f\_\_mitochondria;g\_\_Abies | 0 | 0.0% | 0.0% | 0.0% | 0.0% | 0.0% | 0.0% | 0.0% | 0.0% | 0.0% | 0.0% | 0.0% |
|  | k\_\_Bacteria;p\_\_Proteobacteria;c\_\_Alphaproteobacteria;o\_\_Rickettsiales;f\_\_mitochondria;g\_\_Aristolochia | 0 | 0.0% | 0.0% | 0.0% | 0.0% | 0.0% | 0.0% | 0.0% | 0.0% | 0.0% | 0.0% | 0.0% |
|  | k\_\_Bacteria;p\_\_Proteobacteria;c\_\_Alphaproteobacteria;o\_\_Rickettsiales;f\_\_mitochondria;g\_\_Trebouxia | 0 | 0.0% | 0.0% | 0.0% | 0.0% | 0.0% | 0.0% | 0.3% | 0.0% | 0.0% | 0.0% | 0.0% |
|  | k\_\_Bacteria;p\_\_Proteobacteria;c\_\_Alphaproteobacteria;o\_\_Sphingomonadales;f\_\_Erythrobacteraceae;g\_\_ | 0 | 0.1% | 0.0% | 0.1% | 0.0% | 0.2% | 0.1% | 0.2% | 0.0% | 0.0% | 0.0% | 0.0% |
|  | k\_\_Bacteria;p\_\_Proteobacteria;c\_\_Alphaproteobacteria;o\_\_Sphingomonadales;f\_\_Erythrobacteraceae;g\_\_Altererythrobacter | 0 | 0.0% | 0.0% | 0.0% | 0.0% | 0.0% | 0.0% | 0.0% | 0.0% | 0.0% | 0.0% | 0.0% |
|  | k\_\_Bacteria;p\_\_Proteobacteria;c\_\_Alphaproteobacteria;o\_\_Sphingomonadales;f\_\_Sphingomonadaceae;Other | 0 | 0.1% | 0.1% | 0.0% | 0.0% | 0.1% | 0.0% | 0.2% | 0.0% | 0.2% | 0.0% | 0.1% |
|  | k\_\_Bacteria;p\_\_Proteobacteria;c\_\_Alphaproteobacteria;o\_\_Sphingomonadales;f\_\_Sphingomonadaceae;g\_\_Kaistobacter | 0 | 0.0% | 0.0% | 0.0% | 0.0% | 0.0% | 0.0% | 0.0% | 0.0% | 0.0% | 0.0% | 0.0% |
|  | k\_\_Bacteria;p\_\_Proteobacteria;c\_\_Alphaproteobacteria;o\_\_Sphingomonadales;f\_\_Sphingomonadaceae;g\_\_Sphingobium | 0 | 0.0% | 0.0% | 0.0% | 0.1% | 0.0% | 0.0% | 0.1% | 0.0% | 0.0% | 0.1% | 0.0% |
|  | k\_\_Bacteria;p\_\_Proteobacteria;c\_\_Alphaproteobacteria;o\_\_Sphingomonadales;f\_\_Sphingomonadaceae;g\_\_Sphingomonas | 0 | 0.0% | 0.0% | 0.0% | 0.0% | 0.1% | 0.1% | 0.0% | 0.0% | 0.0% | 0.0% | 0.0% |
|  | k\_\_Bacteria;p\_\_Proteobacteria;c\_\_Betaproteobacteria;Other;Other;Other | 0 | 0.0% | 0.0% | 0.0% | 0.0% | 0.0% | 0.0% | 0.0% | 0.0% | 0.0% | 0.0% | 0.0% |
|  | k\_\_Bacteria;p\_\_Proteobacteria;c\_\_Betaproteobacteria;o\_\_Burkholderiales;Other;Other | 0 | 0.0% | 0.0% | 0.0% | 0.0% | 0.0% | 0.0% | 0.0% | 0.0% | 0.0% | 0.0% | 0.0% |
|  | k\_\_Bacteria;p\_\_Proteobacteria;c\_\_Betaproteobacteria;o\_\_Burkholderiales;f\_\_Burkholderiaceae;g\_\_Burkholderia | 0 | 0.0% | 0.0% | 0.0% | 0.0% | 0.0% | 0.0% | 0.0% | 0.0% | 0.0% | 0.0% | 0.0% |
|  | k\_\_Bacteria;p\_\_Proteobacteria;c\_\_Betaproteobacteria;o\_\_Burkholderiales;f\_\_Burkholderiaceae;g\_\_Lautropia | 0 | 0.0% | 0.0% | 0.0% | 0.0% | 0.0% | 0.0% | 0.0% | 0.0% | 0.0% | 0.0% | 0.0% |
|  | k\_\_Bacteria;p\_\_Proteobacteria;c\_\_Betaproteobacteria;o\_\_Burkholderiales;f\_\_Comamonadaceae;Other | 0 | 0.3% | 0.3% | 0.2% | 0.2% | 0.3% | 0.1% | 0.5% | 0.1% | 0.8% | 0.5% | 0.1% |
|  | k\_\_Bacteria;p\_\_Proteobacteria;c\_\_Betaproteobacteria;o\_\_Burkholderiales;f\_\_Comamonadaceae;g\_\_Acidovorax | 0 | 0.0% | 0.0% | 0.0% | 0.0% | 0.0% | 0.0% | 0.0% | 0.0% | 0.0% | 0.0% | 0.0% |
|  | k\_\_Bacteria;p\_\_Proteobacteria;c\_\_Betaproteobacteria;o\_\_Burkholderiales;f\_\_Comamonadaceae;g\_\_Aquabacterium | 0 | 0.1% | 0.0% | 0.0% | 0.0% | 0.0% | 0.0% | 0.2% | 0.0% | 0.2% | 0.0% | 0.0% |
|  | k\_\_Bacteria;p\_\_Proteobacteria;c\_\_Betaproteobacteria;o\_\_Burkholderiales;f\_\_Comamonadaceae;g\_\_Comamonas | 0 | 0.0% | 0.0% | 0.0% | 0.0% | 0.0% | 0.0% | 0.0% | 0.0% | 0.0% | 0.0% | 0.0% |
|  | k\_\_Bacteria;p\_\_Proteobacteria;c\_\_Betaproteobacteria;o\_\_Burkholderiales;f\_\_Comamonadaceae;g\_\_Delftia | 0 | 0.0% | 0.0% | 0.0% | 0.0% | 0.0% | 0.0% | 0.0% | 0.0% | 0.0% | 0.0% | 0.0% |
|  | k\_\_Bacteria;p\_\_Proteobacteria;c\_\_Betaproteobacteria;o\_\_Burkholderiales;f\_\_Comamonadaceae;g\_\_Diaphorobacter | 0 | 0.1% | 0.3% | 0.0% | 0.0% | 0.1% | 0.0% | 0.2% | 0.0% | 0.3% | 0.1% | 0.0% |
|  | k\_\_Bacteria;p\_\_Proteobacteria;c\_\_Betaproteobacteria;o\_\_Burkholderiales;f\_\_Comamonadaceae;g\_\_Variovorax | 0 | 0.0% | 0.0% | 0.0% | 0.0% | 0.1% | 0.0% | 0.0% | 0.0% | 0.0% | 0.0% | 0.0% |
|  | k\_\_Bacteria;p\_\_Proteobacteria;c\_\_Betaproteobacteria;o\_\_Burkholderiales;f\_\_Oxalobacteraceae;Other | 0 | 0.0% | 0.0% | 0.0% | 0.0% | 0.0% | 0.0% | 0.0% | 0.0% | 0.0% | 0.0% | 0.0% |
|  | k\_\_Bacteria;p\_\_Proteobacteria;c\_\_Betaproteobacteria;o\_\_Burkholderiales;f\_\_Oxalobacteraceae;g\_\_ | 0 | 0.0% | 0.0% | 0.0% | 0.0% | 0.0% | 0.0% | 0.0% | 0.0% | 0.0% | 0.0% | 0.0% |
|  | k\_\_Bacteria;p\_\_Proteobacteria;c\_\_Betaproteobacteria;o\_\_Burkholderiales;f\_\_Oxalobacteraceae;g\_\_Cupriavidus | 0 | 0.0% | 0.0% | 0.0% | 0.0% | 0.0% | 0.0% | 0.0% | 0.0% | 0.0% | 0.0% | 0.0% |
|  | k\_\_Bacteria;p\_\_Proteobacteria;c\_\_Betaproteobacteria;o\_\_Burkholderiales;f\_\_Oxalobacteraceae;g\_\_Herbaspirillum | 0 | 0.0% | 0.0% | 0.0% | 0.0% | 0.0% | 0.0% | 0.0% | 0.0% | 0.0% | 0.0% | 0.0% |
|  | k\_\_Bacteria;p\_\_Proteobacteria;c\_\_Betaproteobacteria;o\_\_Burkholderiales;f\_\_Oxalobacteraceae;g\_\_Janthinobacterium | 0 | 0.0% | 0.0% | 0.0% | 0.0% | 0.0% | 0.0% | 0.0% | 0.0% | 0.0% | 0.0% | 0.0% |
|  | k\_\_Bacteria;p\_\_Proteobacteria;c\_\_Betaproteobacteria;o\_\_Neisseriales;f\_\_Neisseriaceae;Other | 0 | 0.0% | 0.1% | 0.0% | 0.0% | 0.0% | 0.0% | 0.0% | 0.0% | 0.0% | 0.0% | 0.0% |
|  | k\_\_Bacteria;p\_\_Proteobacteria;c\_\_Betaproteobacteria;o\_\_Neisseriales;f\_\_Neisseriaceae;g\_\_ | 0 | 0.0% | 0.1% | 0.1% | 0.0% | 0.0% | 0.1% | 0.0% | 0.0% | 0.0% | 0.0% | 0.0% |
|  | k\_\_Bacteria;p\_\_Proteobacteria;c\_\_Betaproteobacteria;o\_\_Neisseriales;f\_\_Neisseriaceae;g\_\_Kingella | 0 | 0.0% | 0.0% | 0.0% | 0.0% | 0.0% | 0.0% | 0.0% | 0.0% | 0.0% | 0.0% | 0.0% |
|  | k\_\_Bacteria;p\_\_Proteobacteria;c\_\_Betaproteobacteria;o\_\_Neisseriales;f\_\_Neisseriaceae;g\_\_Neisseria | 0 | 0.3% | 0.7% | 0.0% | 0.0% | 0.1% | 0.0% | 0.1% | 1.1% | 0.7% | 0.2% | 0.3% |
|  | k\_\_Bacteria;p\_\_Proteobacteria;c\_\_Betaproteobacteria;o\_\_Rhodocyclales;f\_\_Rhodocyclaceae;Other | 0 | 0.0% | 0.0% | 0.0% | 0.0% | 0.1% | 0.0% | 0.0% | 0.0% | 0.0% | 0.0% | 0.0% |
|  | k\_\_Bacteria;p\_\_Proteobacteria;c\_\_Betaproteobacteria;o\_\_Rhodocyclales;f\_\_Rhodocyclaceae;g\_\_Dechloromonas | 0 | 0.0% | 0.1% | 0.0% | 0.0% | 0.0% | 0.0% | 0.1% | 0.0% | 0.1% | 0.0% | 0.0% |
|  | k\_\_Bacteria;p\_\_Proteobacteria;c\_\_Betaproteobacteria;o\_\_Rhodocyclales;f\_\_Rhodocyclaceae;g\_\_Zoogloea | 0 | 0.0% | 0.0% | 0.0% | 0.0% | 0.0% | 0.0% | 0.0% | 0.0% | 0.0% | 0.0% | 0.0% |
|  | k\_\_Bacteria;p\_\_Proteobacteria;c\_\_Deltaproteobacteria;o\_\_Bdellovibrionales;f\_\_Bdellovibrionaceae;g\_\_Bdellovibrio | 0 | 0.0% | 0.0% | 0.0% | 0.0% | 0.0% | 0.0% | 0.0% | 0.0% | 0.0% | 0.0% | 0.0% |
|  | k\_\_Bacteria;p\_\_Proteobacteria;c\_\_Epsilonproteobacteria;o\_\_Campylobacterales;f\_\_Campylobacteraceae;g\_\_Campylobacter | 0 | 0.0% | 0.0% | 0.1% | 0.0% | 0.0% | 0.0% | 0.0% | 0.0% | 0.0% | 0.0% | 0.0% |
|  | k\_\_Bacteria;p\_\_Proteobacteria;c\_\_Epsilonproteobacteria;o\_\_Campylobacterales;f\_\_Helicobacteraceae;Other | 0 | 0.0% | 0.0% | 0.0% | 0.0% | 0.0% | 0.0% | 0.0% | 0.0% | 0.0% | 0.0% | 0.0% |
|  | k\_\_Bacteria;p\_\_Proteobacteria;c\_\_Gammaproteobacteria;Other;Other;Other | 0 | 0.0% | 0.0% | 0.0% | 0.0% | 0.0% | 0.0% | 0.0% | 0.0% | 0.0% | 0.0% | 0.0% |
|  | k\_\_Bacteria;p\_\_Proteobacteria;c\_\_Gammaproteobacteria;o\_\_Aeromonadales;f\_\_Aeromonadaceae;Other | 0 | 0.0% | 0.0% | 0.0% | 0.0% | 0.0% | 0.0% | 0.0% | 0.0% | 0.0% | 0.0% | 0.0% |
|  | k\_\_Bacteria;p\_\_Proteobacteria;c\_\_Gammaproteobacteria;o\_\_Aeromonadales;f\_\_Succinivibrionaceae;Other | 0 | 0.0% | 0.0% | 0.0% | 0.0% | 0.0% | 0.0% | 0.0% | 0.0% | 0.0% | 0.0% | 0.0% |
|  | k\_\_Bacteria;p\_\_Proteobacteria;c\_\_Gammaproteobacteria;o\_\_Aeromonadales;f\_\_Succinivibrionaceae;g\_\_Succinivibrio | 0 | 0.0% | 0.0% | 0.0% | 0.0% | 0.0% | 0.0% | 0.0% | 0.0% | 0.0% | 0.0% | 0.0% |
|  | k\_\_Bacteria;p\_\_Proteobacteria;c\_\_Gammaproteobacteria;o\_\_Alteromonadales;f\_\_Shewanellaceae;g\_\_Shewanella | 0 | 0.0% | 0.0% | 0.0% | 0.0% | 0.0% | 0.0% | 0.0% | 0.0% | 0.0% | 0.0% | 0.0% |
|  | k\_\_Bacteria;p\_\_Proteobacteria;c\_\_Gammaproteobacteria;o\_\_Enterobacteriales;f\_\_Enterobacteriaceae;Other | 0 | 1.7% | 0.3% | 7.3% | 1.5% | 2.6% | 1.6% | 0.5% | 0.9% | 1.1% | 0.2% | 0.7% |
|  | k\_\_Bacteria;p\_\_Proteobacteria;c\_\_Gammaproteobacteria;o\_\_Enterobacteriales;f\_\_Enterobacteriaceae;g\_\_Serratia | 0 | 0.0% | 0.0% | 0.2% | 0.0% | 0.1% | 0.0% | 0.0% | 0.1% | 0.0% | 0.0% | 0.0% |
|  | k\_\_Bacteria;p\_\_Proteobacteria;c\_\_Gammaproteobacteria;o\_\_Enterobacteriales;f\_\_Enterobacteriaceae;g\_\_Trabulsiella | 0 | 0.0% | 0.0% | 0.0% | 0.0% | 0.0% | 0.0% | 0.1% | 0.0% | 0.1% | 0.1% | 0.1% |
|  | k\_\_Bacteria;p\_\_Proteobacteria;c\_\_Gammaproteobacteria;o\_\_Pasteurellales;f\_\_Pasteurellaceae;Other | 0 | 0.2% | 0.6% | 0.3% | 0.0% | 0.1% | 0.1% | 0.1% | 0.1% | 0.1% | 0.1% | 0.3% |
|  | k\_\_Bacteria;p\_\_Proteobacteria;c\_\_Gammaproteobacteria;o\_\_Pasteurellales;f\_\_Pasteurellaceae;g\_\_Actinobacillus | 0 | 0.0% | 0.0% | 0.0% | 0.1% | 0.0% | 0.0% | 0.0% | 0.0% | 0.0% | 0.0% | 0.0% |
|  | k\_\_Bacteria;p\_\_Proteobacteria;c\_\_Gammaproteobacteria;o\_\_Pasteurellales;f\_\_Pasteurellaceae;g\_\_Haemophilus | 1 | 5.0% | 10.4% | 14.8% | 0.5% | 0.2% | 3.6% | 1.7% | 4.4% | 4.3% | 0.5% | 10.3% |
|  | k\_\_Bacteria;p\_\_Proteobacteria;c\_\_Gammaproteobacteria;o\_\_Pseudomonadales;f\_\_Moraxellaceae;Other | 0 | 0.0% | 0.0% | 0.0% | 0.0% | 0.0% | 0.0% | 0.0% | 0.0% | 0.0% | 0.0% | 0.0% |
|  | k\_\_Bacteria;p\_\_Proteobacteria;c\_\_Gammaproteobacteria;o\_\_Pseudomonadales;f\_\_Moraxellaceae;g\_\_Acinetobacter | 0 | 0.3% | 0.1% | 0.3% | 0.1% | 0.3% | 0.2% | 0.4% | 0.3% | 0.8% | 0.1% | 0.2% |
|  | k\_\_Bacteria;p\_\_Proteobacteria;c\_\_Gammaproteobacteria;o\_\_Pseudomonadales;f\_\_Moraxellaceae;g\_\_Enhydrobacter | 0 | 0.0% | 0.0% | 0.0% | 0.0% | 0.0% | 0.0% | 0.1% | 0.0% | 0.0% | 0.0% | 0.0% |
|  | k\_\_Bacteria;p\_\_Proteobacteria;c\_\_Gammaproteobacteria;o\_\_Pseudomonadales;f\_\_Moraxellaceae;g\_\_Moraxella | 0 | 0.7% | 1.3% | 3.1% | 0.8% | 0.2% | 0.3% | 0.5% | 0.2% | 0.5% | 0.3% | 0.1% |
|  | k\_\_Bacteria;p\_\_Proteobacteria;c\_\_Gammaproteobacteria;o\_\_Pseudomonadales;f\_\_Moraxellaceae;g\_\_Psychrobacter | 0 | 0.0% | 0.0% | 0.0% | 0.0% | 0.0% | 0.0% | 0.0% | 0.0% | 0.0% | 0.0% | 0.0% |
|  | k\_\_Bacteria;p\_\_Proteobacteria;c\_\_Gammaproteobacteria;o\_\_Pseudomonadales;f\_\_Pseudomonadaceae;g\_\_Pseudomonas | 0 | 0.0% | 0.0% | 0.0% | 0.0% | 0.1% | 0.1% | 0.1% | 0.0% | 0.0% | 0.0% | 0.0% |
|  | k\_\_Bacteria;p\_\_Proteobacteria;c\_\_Gammaproteobacteria;o\_\_Vibrionales;Other;Other | 0 | 0.0% | 0.0% | 0.0% | 0.0% | 0.0% | 0.0% | 0.0% | 0.0% | 0.0% | 0.0% | 0.0% |
|  | k\_\_Bacteria;p\_\_Proteobacteria;c\_\_Gammaproteobacteria;o\_\_Vibrionales;f\_\_Vibrionaceae;g\_\_Vibrio | 0 | 0.3% | 0.0% | 0.1% | 0.0% | 0.0% | 0.0% | 0.1% | 0.0% | 2.8% | 0.0% | 0.0% |
|  | k\_\_Bacteria;p\_\_Proteobacteria;c\_\_Gammaproteobacteria;o\_\_Xanthomonadales;f\_\_Xanthomonadaceae;g\_\_Stenotrophomonas | 0 | 0.0% | 0.0% | 0.0% | 0.1% | 0.2% | 0.0% | 0.0% | 0.0% | 0.0% | 0.0% | 0.0% |
|  | k\_\_Bacteria;p\_\_Spirochaetes;c\_\_[Brachyspirae];o\_\_[Brachyspirales];f\_\_Brachyspiraceae;g\_\_Brachyspira | 0 | 0.0% | 0.0% | 0.0% | 0.0% | 0.0% | 0.0% | 0.0% | 0.0% | 0.0% | 0.0% | 0.0% |
|  | k\_\_Bacteria;p\_\_Tenericutes;c\_\_Mollicutes;o\_\_Mycoplasmatales;f\_\_Mycoplasmataceae;g\_\_Mycoplasma | 0 | 0.0% | 0.0% | 0.0% | 0.0% | 0.0% | 0.0% | 0.0% | 0.0% | 0.0% | 0.0% | 0.0% |
|  | k\_\_Bacteria;p\_\_Tenericutes;c\_\_Mollicutes;o\_\_Mycoplasmatales;f\_\_Mycoplasmataceae;g\_\_Ureaplasma | 0 | 0.0% | 0.0% | 0.0% | 0.0% | 0.0% | 0.0% | 0.0% | 0.0% | 0.0% | 0.0% | 0.0% |
|  | k\_\_Bacteria;p\_\_Tenericutes;c\_\_Mollicutes;o\_\_RF39;f\_\_;g\_\_ | 0 | 0.0% | 0.0% | 0.0% | 0.0% | 0.0% | 0.0% | 0.0% | 0.0% | 0.1% | 0.0% | 0.0% |
|  | k\_\_Bacteria;p\_\_Verrucomicrobia;c\_\_Verruco-5;o\_\_WCHB1-41;f\_\_RFP12;g\_\_ | 0 | 0.1% | 0.0% | 0.0% | 0.0% | 0.0% | 0.0% | 0.0% | 0.5% | 0.6% | 0.1% | 0.0% |
|  | k\_\_Bacteria;p\_\_Verrucomicrobia;c\_\_Verrucomicrobiae;o\_\_Verrucomicrobiales;f\_\_Verrucomicrobiaceae;Other | 0 | 0.0% | 0.0% | 0.0% | 0.0% | 0.0% | 0.0% | 0.0% | 0.0% | 0.0% | 0.0% | 0.0% |
|  | k\_\_Bacteria;p\_\_Verrucomicrobia;c\_\_Verrucomicrobiae;o\_\_Verrucomicrobiales;f\_\_Verrucomicrobiaceae;g\_\_Akkermansia | 0 | 0.0% | 0.0% | 0.0% | 0.0% | 0.0% | 0.0% | 0.0% | 0.0% | 0.0% | 0.0% | 0.0% |
|  | k\_\_Bacteria;p\_\_WPS-2;c\_\_;o\_\_;f\_\_;g\_\_ | 0 | 0.0% | 0.0% | 0.0% | 0.0% | 0.0% | 0.0% | 0.0% | 0.1% | 0.1% | 0.0% | 0.0% |
|  | k\_\_Bacteria;p\_\_[Thermi];c\_\_Deinococci;o\_\_Deinococcales;f\_\_Deinococcaceae;g\_\_Deinococcus | 0 | 0.0% | 0.0% | 0.0% | 0.0% | 0.0% | 0.0% | 0.0% | 0.0% | 0.0% | 0.0% | 0.0% |
