# Supplementary material for: Characterization of the nasopharyngeal microbiota in health and during rhinovirus challenge
Source: Microbiome. 2014 Jun 25;2:22. doi: 10.1186/2049-2618-2-22 (PMC4098959; doi:10.1186/2049-2618-2-22)
Supplement: Additional file 4 — Qiime taxonomic composition results (compressed). Taxa summary plots organized by time point. Bar and area charts describing the taxonomic composition of all samples analyzed at all taxonomic ranks from genus to phylum, organized by time point. [file 2049-2618-2-22-S4.zip › 8440692981118328_add4.tgz!/Tax_composition_less1_byTime/taxa_summary_plots/charts/2YiukBwgtUWL5YUsrHdXdUub91KWPb.pdf]

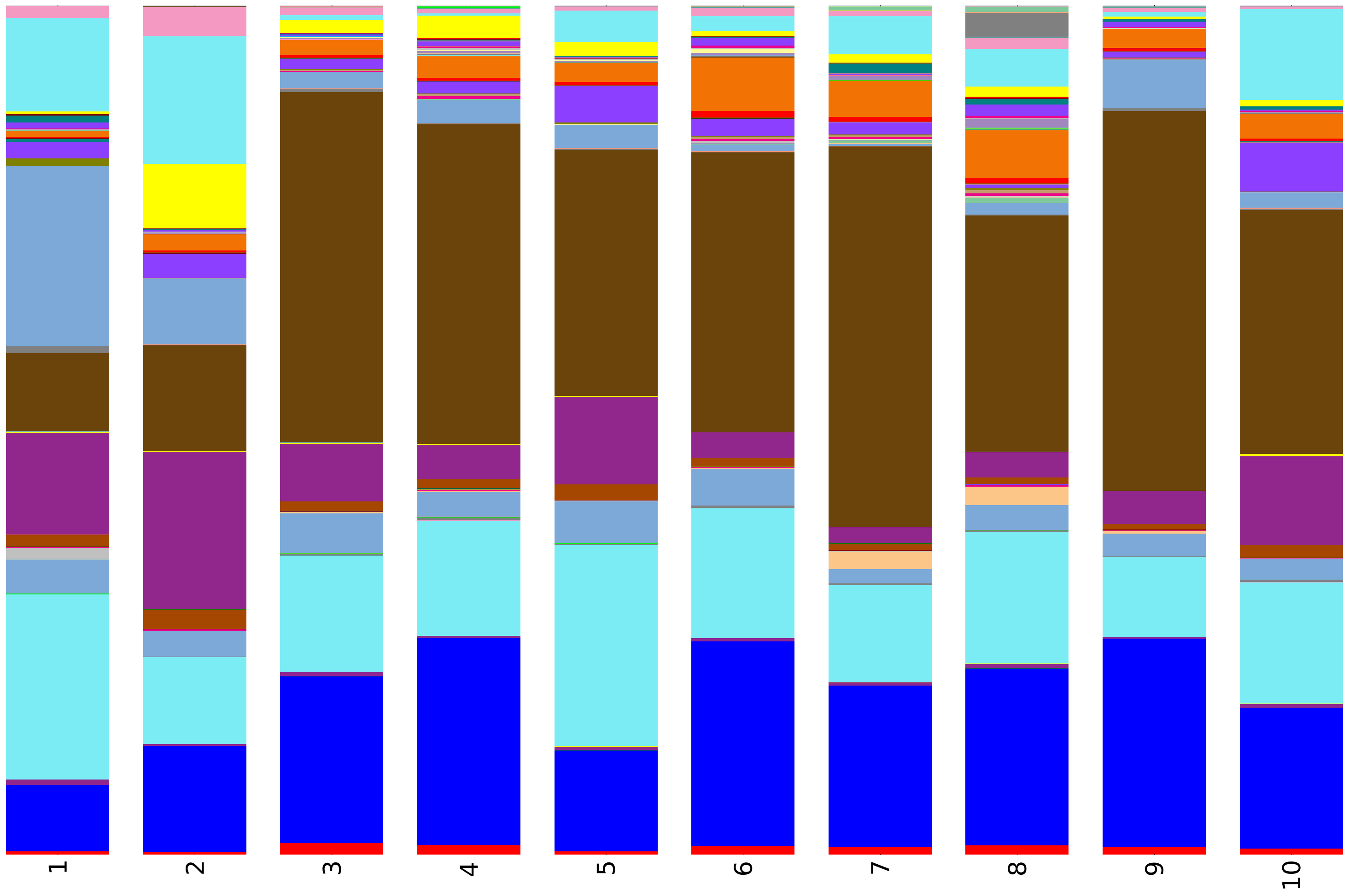
1
2
3
4
5
6
7
8
9
10
