# Supplementary material for: Characterization of the nasopharyngeal microbiota in health and during rhinovirus challenge
Source: Microbiome. 2014 Jun 25;2:22. doi: 10.1186/2049-2618-2-22 (PMC4098959; doi:10.1186/2049-2618-2-22)
Supplement: Additional file 4 — Qiime taxonomic composition results (compressed). Taxa summary plots organized by time point. Bar and area charts describing the taxonomic composition of all samples analyzed at all taxonomic ranks from genus to phylum, organized by time point. [file 2049-2618-2-22-S4.zip › 8440692981118328_add4.tgz!/Tax_composition_less1_byTime/taxa_summary_plots/charts/BotPJH9zxzEwtQcRXMKiZSy9Nh6tjz_legend.pdf]

- Unclassified;Other;Other;Other
- k__Bacteria;Other;Other;Other
- k__Bacteria;p__Actinobacteria;Other;Other
- k__Bacteria;p__Actinobacteria;c__Actinobacteria;Other
- k__Bacteria;p__Actinobacteria;c__Actinobacteria;o__Actinomycetales
- k__Bacteria;p__Actinobacteria;c__Actinobacteria;o__Bifidobacteriales
- k__Bacteria;p__Actinobacteria;c__Coriobacteriia;o__Coriobacteriales
- k__Bacteria;p__Bacteroidetes;Other;Other
- k__Bacteria;p__Bacteroidetes;c__Bacteroidia;o__Bacteroidales
- k__Bacteria;p__Bacteroidetes;c__Cytophagia;o__Cytophagales
- k__Bacteria;p__Bacteroidetes;c__Flavobacteriia;o__Flavobacteriales
- k__Bacteria;p__Cyanobacteria;Other;Other
- k__Bacteria;p__Cyanobacteria;c__4C0d-2;o__YS2
- k__Bacteria;p__Cyanobacteria;c__Synechococcophycideae;o__Synechococcales
- k__Bacteria;p__Firmicutes;Other;Other
- k__Bacteria;p__Firmicutes;c__Bacilli;Other
- k__Bacteria;p__Firmicutes;c__Bacilli;o__Bacillales
- k__Bacteria;p__Firmicutes;c__Bacilli;o__Gemellales
- k__Bacteria;p__Firmicutes;c__Bacilli;o__Lactobacillales
- k__Bacteria;p__Firmicutes;c__Bacilli;o__Turicibacterales
- k__Bacteria;p__Firmicutes;c__Clostridia;o__Clostridiales
- k__Bacteria;p__Firmicutes;c__Erysipelotrichi;o__Erysipelotrichales
- k__Bacteria;p__Fusobacteria;c__Fusobacteriia;o__Fusobacteriales
- k__Bacteria;p__Lentisphaerae;c__[Lentisphaeria];o__Victivallales
- k__Bacteria;p__Proteobacteria;Other;Other
- k__Bacteria;p__Proteobacteria;c__Alphaproteobacteria;Other
- k__Bacteria;p__Proteobacteria;c__Alphaproteobacteria;o__
- k__Bacteria;p__Proteobacteria;c__Alphaproteobacteria;o__BD7-3
- k__Bacteria;p__Proteobacteria;c__Alphaproteobacteria;o__Caulobacterales
- k__Bacteria;p__Proteobacteria;c__Alphaproteobacteria;o__RF32
- k__Bacteria;p__Proteobacteria;c__Alphaproteobacteria;o__Rhizobiales
- k__Bacteria;p__Proteobacteria;c__Alphaproteobacteria;o__Rhodospirillales
- k__Bacteria;p__Proteobacteria;c__Alphaproteobacteria;o__Rickettsiales
- k__Bacteria;p__Proteobacteria;c__Alphaproteobacteria;o__Sphingomonadales
- k__Bacteria;p__Proteobacteria;c__Betaproteobacteria;Other
- k__Bacteria;p__Proteobacteria;c__Betaproteobacteria;o__Burkholderiales
- k__Bacteria;p__Proteobacteria;c__Betaproteobacteria;o__Neisseriales
- k__Bacteria;p__Proteobacteria;c__Betaproteobacteria;o__Rhodocyclales
- k__Bacteria;p__Proteobacteria;c__Deltaproteobacteria;o__Bdellovibrionales
- k__Bacteria;p__Proteobacteria;c__Epsilonproteobacteria;o__Campylobacterales
- k__Bacteria;p__Proteobacteria;c__Gammaproteobacteria;Other
- k__Bacteria;p__Proteobacteria;c__Gammaproteobacteria;o__Aeromonadales
- k__Bacteria;p__Proteobacteria;c__Gammaproteobacteria;o__Alteromonadales
- k__Bacteria;p__Proteobacteria;c__Gammaproteobacteria;o__Enterobacteriales
- k__Bacteria;p__Proteobacteria;c__Gammaproteobacteria;o__Pasteurellales
- k__Bacteria;p__Proteobacteria;c__Gammaproteobacteria;o__Pseudomonadales
- k__Bacteria;p__Proteobacteria;c__Gammaproteobacteria;o__Vibrionales
- k__Bacteria;p__Proteobacteria;c__Gammaproteobacteria;o__Xanthomonadales
- k__Bacteria;p__Spirochaetes;c__[Brachyspirae];o__[Brachyspirales]
- k__Bacteria;p__Tenericutes;c__Mollicutes;o__Mycoplasmatales
- k__Bacteria;p__Tenericutes;c__Mollicutes;o__RF39
- k__Bacteria;p__Verrucomicrobia;c__Verruco-5;o__WCHB1-41
- k__Bacteria;p__Verrucomicrobia;c__Verrucomicrobiae;o__Verrucomicrobiales
- k__Bacteria;p__WPS-2;c__;o__
- k__Bacteria;p__[Thermi];c__Deinococci;o__Deinococcales
