# Supplementary material for: Characterization of the nasopharyngeal microbiota in health and during rhinovirus challenge
Source: Microbiome. 2014 Jun 25;2:22. doi: 10.1186/2049-2618-2-22 (PMC4098959; doi:10.1186/2049-2618-2-22)
Supplement: Additional file 4 — Qiime taxonomic composition results (compressed). Taxa summary plots organized by time point. Bar and area charts describing the taxonomic composition of all samples analyzed at all taxonomic ranks from genus to phylum, organized by time point. [file 2049-2618-2-22-S4.zip › 8440692981118328_add4.tgz!/Tax_composition_less1_byTime/taxa_summary_plots/charts/ZLnha6xTJunAS17PA4hfWWpxK6zFOH_legend.pdf]

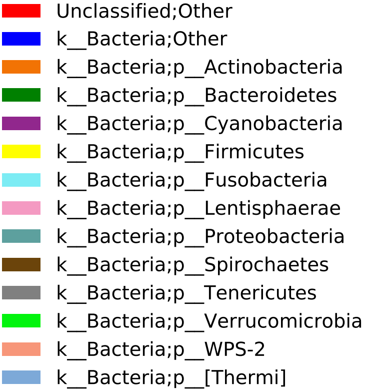
Unclassified;Other
k__Bacteria;Other
k__Bacteria;p__Actinobacteria
k__Bacteria;p__Bacteroidetes
k__Bacteria;p__Cyanobacteria
k__Bacteria;p__Firmicutes
k__Bacteria;p__Fusobacteria
k__Bacteria;p__Lentisphaerae
k__Bacteria;p__Proteobacteria
k__Bacteria;p__Spirochaetes
k__Bacteria;p__Tenericutes
k__Bacteria;p__Verrucomicrobia
k__Bacteria;p__WPS-2
k__Bacteria;p__[Thermi]
