# Supplementary material for: Characterization of the nasopharyngeal microbiota in health and during rhinovirus challenge
Source: Microbiome. 2014 Jun 25;2:22. doi: 10.1186/2049-2618-2-22 (PMC4098959; doi:10.1186/2049-2618-2-22)
Supplement: Additional file 7 — Compressed file. Alpha diversity_150. Alpha diversity analyses including rarefaction plots. Plots go to minimum sequences/sample (approximately 150). [file 2049-2618-2-22-S7.zip › 8440692981118328_add7.tgz!/alpha_div_less1/alpha_rarefaction_plots/rarefaction_plots.html]

 
 
 
   
   Rarefaction Curves 
 
td.data{font-size:10px;border-spacing:0px 10px;text-align:center;}
td.headers{font-size:12px;font-weight:bold;text-align:center;}
table{border-spacing:0px;}
.removed{display:none;}
.expands{cursor:pointer; cursor:hand;}
.child1 td:first-child{padding-left: 3px;}
 
 

function show_hide_category(checkobject){
    var imagetype=document.getElementById('imagetype').value;
    img=document.getElementById(checkobject.name.replace('_raw'+imagetype,'_ave'+imagetype))
    if (checkobject.checked==false){
        img.style.display='none';
    }else{
        img.style.display='';
    }
}

function reset_tree(){
    var category=document.getElementById('category').value;
    var metric=document.getElementById('metric').value;
    var old_all_categories=document.getElementById('all_categories');
    var imagetype=document.getElementById('imagetype').value;
    cat_list=old_all_categories.value.split('$#!')
    if (metric!='' && category != ''){
    for (var i=1, il=cat_list.length; i 

 
 
 
 
 
 
 
 
  
  Select a Metric:  
 
 
   
 chao1 
 observed_species 
 shannon 
 
 
    Select a Category:  
 
 
   
 BarcodeSequence 
 Description 
 Innoculation 
 LibCodes 
 SampleID 
 Subject 
 TimePoint 
 
 
 
 
 
  

 
      Show Categories: 
     
           
         All 
         None 
         Invert 
     
      
 Legend   
  ▶     ■    ACATACGCGTAG   
  ∟    ◆   BW.03.5   
  ∟    ◆   BW.08.6   
  ▶     ■    ACGACTACAGAG   
  ∟    ◆   BW.02.9   
  ∟    ◆   BW.07.10   
  ▶     ■    ACGAGTGCGTAG   
  ∟    ◆   BW.01.1   
  ∟    ◆   BW.06.1   
  ▶     ■    ACGCGAGTATAG   
  ∟    ◆   BW.03.6   
  ∟    ◆   BW.08.7   
  ▶     ■    ACGCGATCGAAG   
  ∟    ◆   BW.05.7   
  ∟    ◆   BW.10.8   
  ▶     ■    ACGCTCGACAAG   
  ∟    ◆   BW.01.2   
  ∟    ◆   BW.06.2   
  ▶     ■    ACTACTATGTAG   
  ∟    ◆   BW.03.7   
  ∟    ◆   BW.08.8   
  ▶     ■    ACTAGCAGTAAG   
  ∟    ◆   BW.05.8   
  ∟    ◆   BW.10.9   
  ▶     ■    ACTGTACAGTAG   
  ∟    ◆   BW.03.8   
  ∟    ◆   BW.08.9   
  ▶     ■    AGACGCACTCAG   
  ∟    ◆   BW.01.3   
  ∟    ◆   BW.06.4   
  ▶     ■    AGACTATACTAG   
  ∟    ◆   BW.03.9   
  ∟    ◆   BW.08.10   
  ▶     ■    AGCACTGTAGAG   
  ∟    ◆   BW.01.4   
  ∟    ◆   BW.06.5   
  ▶     ■    AGCGTCGTCTAG   
  ∟    ◆   BW.03.10   
  ∟    ◆   BW.09.1   
  ▶     ■    AGCTCACGTAAG   
  ∟    ◆   BW.05.9   
  ∟    ◆   BW.10.10   
  ▶     ■    AGTACGCTATAG   
  ∟    ◆   BW.04.1   
  ∟    ◆   BW.09.2   
  ▶     ■    AGTATACATAAG   
  ∟    ◆   BW.05.10   
  ▶     ■    ATACGACGTAAG   
  ∟    ◆   BW.02.4   
  ∟    ◆   BW.07.5   
  ▶     ■    ATAGAGTACTAG   
  ∟    ◆   BW.04.2   
  ∟    ◆   BW.09.3   
  ▶     ■    ATATCGCGAGAG   
  ∟    ◆   BW.01.6   
  ∟    ◆   BW.06.7   
  ▶     ■    ATCAGACACGAG   
  ∟    ◆   BW.01.5   
  ∟    ◆   BW.06.6   
  ▶     ■    CACGCTACGTAG   
  ∟    ◆   BW.04.3   
  ∟    ◆   BW.09.4   
  ▶     ■    CAGTAGACGTAG   
  ∟    ◆   BW.04.4   
  ∟    ◆   BW.09.5   
  ▶     ■    CATAGTAGTGAG   
  ∟    ◆   BW.02.2   
  ∟    ◆   BW.07.3   
  ▶     ■    CGACGTGACTAG   
  ∟    ◆   BW.04.5   
  ∟    ◆   BW.09.6   
  ▶     ■    CGAGAGATACAG   
  ∟    ◆   BW.02.3   
  ∟    ◆   BW.07.4   
  ▶     ■    CGTAGACTAGAG   
  ∟    ◆   BW.02.10   
  ∟    ◆   BW.08.1   
  ▶     ■    CGTCTAGTACAG   
  ∟    ◆   BW.02.6   
  ∟    ◆   BW.07.7   
  ▶     ■    CGTGTCTCTAAG   
  ∟    ◆   BW.01.7   
  ∟    ◆   BW.06.8   
  ▶     ■    CTCGCGTGTCAG   
  ∟    ◆   BW.01.8   
  ∟    ◆   BW.06.9   
  ▶     ■    TACACACACTAG   
  ∟    ◆   BW.04.6   
  ∟    ◆   BW.09.7   
  ▶     ■    TACACGTGATAG   
  ∟    ◆   BW.04.7   
  ∟    ◆   BW.09.8   
  ▶     ■    TACAGATCGTAG   
  ∟    ◆   BW.04.8   
  ∟    ◆   BW.09.9   
  ▶     ■    TACGAGTATGAG   
  ∟    ◆   BW.03.1   
  ∟    ◆   BW.08.2   
  ▶     ■    TACGCTGTCTAG   
  ∟    ◆   BW.04.9   
  ∟    ◆   BW.09.10   
  ▶     ■    TACTCTCGTGAG   
  ∟    ◆   BW.08.3   
  ▶     ■    TAGAGACGAGAG   
  ∟    ◆   BW.03.3   
  ∟    ◆   BW.08.4   
  ▶     ■    TAGTATCAGCAG   
  ∟    ◆   BW.01.9   
  ∟    ◆   BW.06.10   
  ▶     ■    TAGTGTAGATAG   
  ∟    ◆   BW.04.10   
  ∟    ◆   BW.10.1   
  ▶     ■    TCACGTACTAAG   
  ∟    ◆   BW.02.5   
  ∟    ◆   BW.07.6   
  ▶     ■    TCGATCACGTAG   
  ∟    ◆   BW.05.1   
  ∟    ◆   BW.10.2   
  ▶     ■    TCGCACTAGTAG   
  ∟    ◆   BW.05.2   
  ▶     ■    TCGTCGCTCGAG   
  ∟    ◆   BW.03.4   
  ∟    ◆   BW.08.5   
  ▶     ■    TCTACGTAGCAG   
  ∟    ◆   BW.02.7   
  ∟    ◆   BW.07.8   
  ▶     ■    TCTAGCGACTAG   
  ∟    ◆   BW.05.3   
  ∟    ◆   BW.10.4   
  ▶     ■    TCTATACTATAG   
  ∟    ◆   BW.05.4   
  ∟    ◆   BW.10.5   
  ▶     ■    TCTCTATGCGAG   
  ∟    ◆   BW.01.10   
  ∟    ◆   BW.07.1   
  ▶     ■    TGACGTATGTAG   
  ∟    ◆   BW.05.5   
  ∟    ◆   BW.10.6   
  ▶     ■    TGATACGTCTAG   
  ∟    ◆   BW.02.1   
  ∟    ◆   BW.07.2   
  ▶     ■    TGTACTACTCAG   
  ∟    ◆   BW.02.8   
  ∟    ◆   BW.07.9   
  ▶     ■    TGTGAGTAGTAG   
  ∟    ◆   BW.05.6   
  ▶     ■    BW-01.1.Lib_L_27F-001   
  ∟    ◆   BW.01.1   
  ▶     ■    BW-01.10.Lib_L_27F-010   
  ∟    ◆   BW.01.10   
  ▶     ■    BW-01.2.Lib_L_27F-002   
  ∟    ◆   BW.01.2   
  ▶     ■    BW-01.3.Lib_L_27F-003   
  ∟    ◆   BW.01.3   
  ▶     ■    BW-01.4.Lib_L_27F-004   
  ∟    ◆   BW.01.4   
  ▶     ■    BW-01.5.Lib_L_27F-005   
  ∟    ◆   BW.01.5   
  ▶     ■    BW-01.6.Lib_L_27F-006   
  ∟    ◆   BW.01.6   
  ▶     ■    BW-01.7.Lib_L_27F-007   
  ∟    ◆   BW.01.7   
  ▶     ■    BW-01.8.Lib_L_27F-008   
  ∟    ◆   BW.01.8   
  ▶     ■    BW-01.9.Lib_L_27F-009   
  ∟    ◆   BW.01.9   
  ▶     ■    BW-02.1.Lib_L_27F-011   
  ∟    ◆   BW.02.1   
  ▶     ■    BW-02.10.Lib_L_27F-021   
  ∟    ◆   BW.02.10   
  ▶     ■    BW-02.2.Lib_L_27F-013   
  ∟    ◆   BW.02.2   
  ▶     ■    BW-02.3.Lib_L_27F-014   
  ∟    ◆   BW.02.3   
  ▶     ■    BW-02.4.Lib_L_27F-015   
  ∟    ◆   BW.02.4   
  ▶     ■    BW-02.5.Lib_L_27F-016   
  ∟    ◆   BW.02.5   
  ▶     ■    BW-02.6.Lib_L_27F-017   
  ∟    ◆   BW.02.6   
  ▶     ■    BW-02.7.Lib_L_27F-018   
  ∟    ◆   BW.02.7   
  ▶     ■    BW-02.8.Lib_L_27F-019   
  ∟    ◆   BW.02.8   
  ▶     ■    BW-02.9.Lib_L_27F-020   
  ∟    ◆   BW.02.9   
  ▶     ■    BW-03.1.Lib_L_27F-022   
  ∟    ◆   BW.03.1   
  ▶     ■    BW-03.10.Lib_L_27F-031   
  ∟    ◆   BW.03.10   
  ▶     ■    BW-03.3.Lib_L_27F-024   
  ∟    ◆   BW.03.3   
  ▶     ■    BW-03.4.Lib_L_27F-025   
  ∟    ◆   BW.03.4   
  ▶     ■    BW-03.5.Lib_L_27F-026   
  ∟    ◆   BW.03.5   
  ▶     ■    BW-03.6.Lib_L_27F-027   
  ∟    ◆   BW.03.6   
  ▶     ■    BW-03.7.Lib_L_27F-028   
  ∟    ◆   BW.03.7   
  ▶     ■    BW-03.8.Lib_L_27F-029   
  ∟    ◆   BW.03.8   
  ▶     ■    BW-03.9.Lib_L_27F-030   
  ∟    ◆   BW.03.9   
  ▶     ■    BW-04.1.Lib_L_27F-032   
  ∟    ◆   BW.04.1   
  ▶     ■    BW-04.10.Lib_L_27F-041   
  ∟    ◆   BW.04.10   
  ▶     ■    BW-04.2.Lib_L_27F-033   
  ∟    ◆   BW.04.2   
  ▶     ■    BW-04.3.Lib_L_27F-034   
  ∟    ◆   BW.04.3   
  ▶     ■    BW-04.4.Lib_L_27F-035   
  ∟    ◆   BW.04.4   
  ▶     ■    BW-04.5.Lib_L_27F-036   
  ∟    ◆   BW.04.5   
  ▶     ■    BW-04.6.Lib_L_27F-037   
  ∟    ◆   BW.04.6   
  ▶     ■    BW-04.7.Lib_L_27F-038   
  ∟    ◆   BW.04.7   
  ▶     ■    BW-04.8.Lib_L_27F-039   
  ∟    ◆   BW.04.8   
  ▶     ■    BW-04.9.Lib_L_27F-040   
  ∟    ◆   BW.04.9   
  ▶     ■    BW-05.1.Lib_L_27F-042   
  ∟    ◆   BW.05.1   
  ▶     ■    BW-05.10.Lib_L_27F-052   
  ∟    ◆   BW.05.10   
  ▶     ■    BW-05.2.Lib_L_27F-043   
  ∟    ◆   BW.05.2   
  ▶     ■    BW-05.3.Lib_L_27F-044   
  ∟    ◆   BW.05.3   
  ▶     ■    BW-05.4.Lib_L_27F-045   
  ∟    ◆   BW.05.4   
  ▶     ■    BW-05.5.Lib_L_27F-046   
  ∟    ◆   BW.05.5   
  ▶     ■    BW-05.6.Lib_L_27F-047   
  ∟    ◆   BW.05.6   
  ▶     ■    BW-05.7.Lib_L_27F-049   
  ∟    ◆   BW.05.7   
  ▶     ■    BW-05.8.Lib_L_27F-050   
  ∟    ◆   BW.05.8   
  ▶     ■    BW-05.9.Lib_L_27F-051   
  ∟    ◆   BW.05.9   
  ▶     ■    BW-06.1.Lib_L_27F-001   
  ∟    ◆   BW.06.1   
  ▶     ■    BW-06.10.Lib_L_27F-009   
  ∟    ◆   BW.06.10   
  ▶     ■    BW-06.2.Lib_L_27F-002   
  ∟    ◆   BW.06.2   
  ▶     ■    BW-06.4.Lib_L_27F-003   
  ∟    ◆   BW.06.4   
  ▶     ■    BW-06.5.Lib_L_27F-004   
  ∟    ◆   BW.06.5   
  ▶     ■    BW-06.6.Lib_L_27F-005   
  ∟    ◆   BW.06.6   
  ▶     ■    BW-06.7.Lib_L_27F-006   
  ∟    ◆   BW.06.7   
  ▶     ■    BW-06.8.Lib_L_27F-007   
  ∟    ◆   BW.06.8   
  ▶     ■    BW-06.9.Lib_L_27F-008   
  ∟    ◆   BW.06.9   
  ▶     ■    BW-07.1.Lib_L_27F-010   
  ∟    ◆   BW.07.1   
  ▶     ■    BW-07.10.Lib_L_27F-020   
  ∟    ◆   BW.07.10   
  ▶     ■    BW-07.2.Lib_L_27F-011   
  ∟    ◆   BW.07.2   
  ▶     ■    BW-07.3.Lib_L_27F-013   
  ∟    ◆   BW.07.3   
  ▶     ■    BW-07.4.Lib_L_27F-014   
  ∟    ◆   BW.07.4   
  ▶     ■    BW-07.5.Lib_L_27F-015   
  ∟    ◆   BW.07.5   
  ▶     ■    BW-07.6.Lib_L_27F-016   
  ∟    ◆   BW.07.6   
  ▶     ■    BW-07.7.Lib_L_27F-017   
  ∟    ◆   BW.07.7   
  ▶     ■    BW-07.8.Lib_L_27F-018   
  ∟    ◆   BW.07.8   
  ▶     ■    BW-07.9.Lib_L_27F-019   
  ∟    ◆   BW.07.9   
  ▶     ■    BW-08.1.Lib_L_27F-021   
  ∟    ◆   BW.08.1   
  ▶     ■    BW-08.10.Lib_L_27F-030   
  ∟    ◆   BW.08.10   
  ▶     ■    BW-08.2.Lib_L_27F-022   
  ∟    ◆   BW.08.2   
  ▶     ■    BW-08.3.Lib_L_27F-023   
  ∟    ◆   BW.08.3   
  ▶     ■    BW-08.4.Lib_L_27F-024   
  ∟    ◆   BW.08.4   
  ▶     ■    BW-08.5.Lib_L_27F-025   
  ∟    ◆   BW.08.5   
  ▶     ■    BW-08.6.Lib_L_27F-026   
  ∟    ◆   BW.08.6   
  ▶     ■    BW-08.7.Lib_L_27F-027   
  ∟    ◆   BW.08.7   
  ▶     ■    BW-08.8.Lib_L_27F-028   
  ∟    ◆   BW.08.8   
  ▶     ■    BW-08.9.Lib_L_27F-029   
  ∟    ◆   BW.08.9   
  ▶     ■    BW-09.1.Lib_L_27F-031   
  ∟    ◆   BW.09.1   
  ▶     ■    BW-09.10.Lib_L_27F-040   
  ∟    ◆   BW.09.10   
  ▶     ■    BW-09.2.Lib_L_27F-032   
  ∟    ◆   BW.09.2   
  ▶     ■    BW-09.3.Lib_L_27F-033   
  ∟    ◆   BW.09.3   
  ▶     ■    BW-09.4.Lib_L_27F-034   
  ∟    ◆   BW.09.4   
  ▶     ■    BW-09.5.Lib_L_27F-035   
  ∟    ◆   BW.09.5   
  ▶     ■    BW-09.6.Lib_L_27F-036   
  ∟    ◆   BW.09.6   
  ▶     ■    BW-09.7.Lib_L_27F-037   
  ∟    ◆   BW.09.7   
  ▶     ■    BW-09.8.Lib_L_27F-038   
  ∟    ◆   BW.09.8   
  ▶     ■    BW-09.9.Lib_L_27F-039   
  ∟    ◆   BW.09.9   
  ▶     ■    BW-10.1.Lib_L_27F-041   
  ∟    ◆   BW.10.1   
  ▶     ■    BW-10.10.Lib_L_27F-051   
  ∟    ◆   BW.10.10   
  ▶     ■    BW-10.2.Lib_L_27F-042   
  ∟    ◆   BW.10.2   
  ▶     ■    BW-10.4.Lib_L_27F-044   
  ∟    ◆   BW.10.4   
  ▶     ■    BW-10.5.Lib_L_27F-045   
  ∟    ◆   BW.10.5   
  ▶     ■    BW-10.6.Lib_L_27F-046   
  ∟    ◆   BW.10.6   
  ▶     ■    BW-10.8.Lib_L_27F-049   
  ∟    ◆   BW.10.8   
  ▶     ■    BW-10.9.Lib_L_27F-050   
  ∟    ◆   BW.10.9   
  ▶     ■    After   
  ∟    ◆   BW.01.10   
  ∟    ◆   BW.01.9   
  ∟    ◆   BW.02.10   
  ∟    ◆   BW.02.9   
  ∟    ◆   BW.03.10   
  ∟    ◆   BW.03.9   
  ∟    ◆   BW.04.10   
  ∟    ◆   BW.04.9   
  ∟    ◆   BW.05.10   
  ∟    ◆   BW.05.9   
  ∟    ◆   BW.06.10   
  ∟    ◆   BW.06.9   
  ∟    ◆   BW.07.10   
  ∟    ◆   BW.07.9   
  ∟    ◆   BW.08.10   
  ∟    ◆   BW.08.9   
  ∟    ◆   BW.09.10   
  ∟    ◆   BW.09.9   
  ∟    ◆   BW.10.10   
  ∟    ◆   BW.10.9   
  ▶     ■    Before   
  ∟    ◆   BW.01.1   
  ∟    ◆   BW.01.2   
  ∟    ◆   BW.01.3   
  ∟    ◆   BW.02.1   
  ∟    ◆   BW.02.2   
  ∟    ◆   BW.02.3   
  ∟    ◆   BW.03.1   
  ∟    ◆   BW.03.3   
  ∟    ◆   BW.04.1   
  ∟    ◆   BW.04.2   
  ∟    ◆   BW.04.3   
  ∟    ◆   BW.05.1   
  ∟    ◆   BW.05.2   
  ∟    ◆   BW.05.3   
  ∟    ◆   BW.06.1   
  ∟    ◆   BW.06.2   
  ∟    ◆   BW.07.1   
  ∟    ◆   BW.07.2   
  ∟    ◆   BW.07.3   
  ∟    ◆   BW.08.1   
  ∟    ◆   BW.08.2   
  ∟    ◆   BW.08.3   
  ∟    ◆   BW.09.1   
  ∟    ◆   BW.09.2   
  ∟    ◆   BW.09.3   
  ∟    ◆   BW.10.1   
  ∟    ◆   BW.10.2   
  ▶     ■    During   
  ∟    ◆   BW.01.4   
  ∟    ◆   BW.01.5   
  ∟    ◆   BW.01.6   
  ∟    ◆   BW.01.7   
  ∟    ◆   BW.01.8   
  ∟    ◆   BW.02.4   
  ∟    ◆   BW.02.5   
  ∟    ◆   BW.02.6   
  ∟    ◆   BW.02.7   
  ∟    ◆   BW.02.8   
  ∟    ◆   BW.03.4   
  ∟    ◆   BW.03.5   
  ∟    ◆   BW.03.6   
  ∟    ◆   BW.03.7   
  ∟    ◆   BW.03.8   
  ∟    ◆   BW.04.4   
  ∟    ◆   BW.04.5   
  ∟    ◆   BW.04.6   
  ∟    ◆   BW.04.7   
  ∟    ◆   BW.04.8   
  ∟    ◆   BW.05.4   
  ∟    ◆   BW.05.5   
  ∟    ◆   BW.05.6   
  ∟    ◆   BW.05.7   
  ∟    ◆   BW.05.8   
  ∟    ◆   BW.06.4   
  ∟    ◆   BW.06.5   
  ∟    ◆   BW.06.6   
  ∟    ◆   BW.06.7   
  ∟    ◆   BW.06.8   
  ∟    ◆   BW.07.4   
  ∟    ◆   BW.07.5   
  ∟    ◆   BW.07.6   
  ∟    ◆   BW.07.7   
  ∟    ◆   BW.07.8   
  ∟    ◆   BW.08.4   
  ∟    ◆   BW.08.5   
  ∟    ◆   BW.08.6   
  ∟    ◆   BW.08.7   
  ∟    ◆   BW.08.8   
  ∟    ◆   BW.09.4   
  ∟    ◆   BW.09.5   
  ∟    ◆   BW.09.6   
  ∟    ◆   BW.09.7   
  ∟    ◆   BW.09.8   
  ∟    ◆   BW.10.4   
  ∟    ◆   BW.10.5   
  ∟    ◆   BW.10.6   
  ∟    ◆   BW.10.8   
  ▶     ■    Lib_L_27F-001   
  ∟    ◆   BW.01.1   
  ∟    ◆   BW.06.1   
  ▶     ■    Lib_L_27F-002   
  ∟    ◆   BW.01.2   
  ∟    ◆   BW.06.2   
  ▶     ■    Lib_L_27F-003   
  ∟    ◆   BW.01.3   
  ∟    ◆   BW.06.4   
  ▶     ■    Lib_L_27F-004   
  ∟    ◆   BW.01.4   
  ∟    ◆   BW.06.5   
  ▶     ■    Lib_L_27F-005   
  ∟    ◆   BW.01.5   
  ∟    ◆   BW.06.6   
  ▶     ■    Lib_L_27F-006   
  ∟    ◆   BW.01.6   
  ∟    ◆   BW.06.7   
  ▶     ■    Lib_L_27F-007   
  ∟    ◆   BW.01.7   
  ∟    ◆   BW.06.8   
  ▶     ■    Lib_L_27F-008   
  ∟    ◆   BW.01.8   
  ∟    ◆   BW.06.9   
  ▶     ■    Lib_L_27F-009   
  ∟    ◆   BW.01.9   
  ∟    ◆   BW.06.10   
  ▶     ■    Lib_L_27F-010   
  ∟    ◆   BW.01.10   
  ∟    ◆   BW.07.1   
  ▶     ■    Lib_L_27F-011   
  ∟    ◆   BW.02.1   
  ∟    ◆   BW.07.2   
  ▶     ■    Lib_L_27F-013   
  ∟    ◆   BW.02.2   
  ∟    ◆   BW.07.3   
  ▶     ■    Lib_L_27F-014   
  ∟    ◆   BW.02.3   
  ∟    ◆   BW.07.4   
  ▶     ■    Lib_L_27F-015   
  ∟    ◆   BW.02.4   
  ∟    ◆   BW.07.5   
  ▶     ■    Lib_L_27F-016   
  ∟    ◆   BW.02.5   
  ∟    ◆   BW.07.6   
  ▶     ■    Lib_L_27F-017   
  ∟    ◆   BW.02.6   
  ∟    ◆   BW.07.7   
  ▶     ■    Lib_L_27F-018   
  ∟    ◆   BW.02.7   
  ∟    ◆   BW.07.8   
  ▶     ■    Lib_L_27F-019   
  ∟    ◆   BW.02.8   
  ∟    ◆   BW.07.9   
  ▶     ■    Lib_L_27F-020   
  ∟    ◆   BW.02.9   
  ∟    ◆   BW.07.10   
  ▶     ■    Lib_L_27F-021   
  ∟    ◆   BW.02.10   
  ∟    ◆   BW.08.1   
  ▶     ■    Lib_L_27F-022   
  ∟    ◆   BW.03.1   
  ∟    ◆   BW.08.2   
  ▶     ■    Lib_L_27F-023   
  ∟    ◆   BW.08.3   
  ▶     ■    Lib_L_27F-024   
  ∟    ◆   BW.03.3   
  ∟    ◆   BW.08.4   
  ▶     ■    Lib_L_27F-025   
  ∟    ◆   BW.03.4   
  ∟    ◆   BW.08.5   
  ▶     ■    Lib_L_27F-026   
  ∟    ◆   BW.03.5   
  ∟    ◆   BW.08.6   
  ▶     ■    Lib_L_27F-027   
  ∟    ◆   BW.03.6   
  ∟    ◆   BW.08.7   
  ▶     ■    Lib_L_27F-028   
  ∟    ◆   BW.03.7   
  ∟    ◆   BW.08.8   
  ▶     ■    Lib_L_27F-029   
  ∟    ◆   BW.03.8   
  ∟    ◆   BW.08.9   
  ▶     ■    Lib_L_27F-030   
  ∟    ◆   BW.03.9   
  ∟    ◆   BW.08.10   
  ▶     ■    Lib_L_27F-031   
  ∟    ◆   BW.03.10   
  ∟    ◆   BW.09.1   
  ▶     ■    Lib_L_27F-032   
  ∟    ◆   BW.04.1   
  ∟    ◆   BW.09.2   
  ▶     ■    Lib_L_27F-033   
  ∟    ◆   BW.04.2   
  ∟    ◆   BW.09.3   
  ▶     ■    Lib_L_27F-034   
  ∟    ◆   BW.04.3   
  ∟    ◆   BW.09.4   
  ▶     ■    Lib_L_27F-035   
  ∟    ◆   BW.04.4   
  ∟    ◆   BW.09.5   
  ▶     ■    Lib_L_27F-036   
  ∟    ◆   BW.04.5   
  ∟    ◆   BW.09.6   
  ▶     ■    Lib_L_27F-037   
  ∟    ◆   BW.04.6   
  ∟    ◆   BW.09.7   
  ▶     ■    Lib_L_27F-038   
  ∟    ◆   BW.04.7   
  ∟    ◆   BW.09.8   
  ▶     ■    Lib_L_27F-039   
  ∟    ◆   BW.04.8   
  ∟    ◆   BW.09.9   
  ▶     ■    Lib_L_27F-040   
  ∟    ◆   BW.04.9   
  ∟    ◆   BW.09.10   
  ▶     ■    Lib_L_27F-041   
  ∟    ◆   BW.04.10   
  ∟    ◆   BW.10.1   
  ▶     ■    Lib_L_27F-042   
  ∟    ◆   BW.05.1   
  ∟    ◆   BW.10.2   
  ▶     ■    Lib_L_27F-043   
  ∟    ◆   BW.05.2   
  ▶     ■    Lib_L_27F-044   
  ∟    ◆   BW.05.3   
  ∟    ◆   BW.10.4   
  ▶     ■    Lib_L_27F-045   
  ∟    ◆   BW.05.4   
  ∟    ◆   BW.10.5   
  ▶     ■    Lib_L_27F-046   
  ∟    ◆   BW.05.5   
  ∟    ◆   BW.10.6   
  ▶     ■    Lib_L_27F-047   
  ∟    ◆   BW.05.6   
  ▶     ■    Lib_L_27F-049   
  ∟    ◆   BW.05.7   
  ∟    ◆   BW.10.8   
  ▶     ■    Lib_L_27F-050   
  ∟    ◆   BW.05.8   
  ∟    ◆   BW.10.9   
  ▶     ■    Lib_L_27F-051   
  ∟    ◆   BW.05.9   
  ∟    ◆   BW.10.10   
  ▶     ■    Lib_L_27F-052   
  ∟    ◆   BW.05.10   
  ▶     ■    BW.01.1   
  ∟    ◆   BW.01.1   
  ▶     ■    BW.01.10   
  ∟    ◆   BW.01.10   
  ▶     ■    BW.01.2   
  ∟    ◆   BW.01.2   
  ▶     ■    BW.01.3   
  ∟    ◆   BW.01.3   
  ▶     ■    BW.01.4   
  ∟    ◆   BW.01.4   
  ▶     ■    BW.01.5   
  ∟    ◆   BW.01.5   
  ▶     ■    BW.01.6   
  ∟    ◆   BW.01.6   
  ▶     ■    BW.01.7   
  ∟    ◆   BW.01.7   
  ▶     ■    BW.01.8   
  ∟    ◆   BW.01.8   
  ▶     ■    BW.01.9   
  ∟    ◆   BW.01.9   
  ▶     ■    BW.02.1   
  ∟    ◆   BW.02.1   
  ▶     ■    BW.02.10   
  ∟    ◆   BW.02.10   
  ▶     ■    BW.02.2   
  ∟    ◆   BW.02.2   
  ▶     ■    BW.02.3   
  ∟    ◆   BW.02.3   
  ▶     ■    BW.02.4   
  ∟    ◆   BW.02.4   
  ▶     ■    BW.02.5   
  ∟    ◆   BW.02.5   
  ▶     ■    BW.02.6   
  ∟    ◆   BW.02.6   
  ▶     ■    BW.02.7   
  ∟    ◆   BW.02.7   
  ▶     ■    BW.02.8   
  ∟    ◆   BW.02.8   
  ▶     ■    BW.02.9   
  ∟    ◆   BW.02.9   
  ▶     ■    BW.03.1   
  ∟    ◆   BW.03.1   
  ▶     ■    BW.03.10   
  ∟    ◆   BW.03.10   
  ▶     ■    BW.03.3   
  ∟    ◆   BW.03.3   
  ▶     ■    BW.03.4   
  ∟    ◆   BW.03.4   
  ▶     ■    BW.03.5   
  ∟    ◆   BW.03.5   
  ▶     ■    BW.03.6   
  ∟    ◆   BW.03.6   
  ▶     ■    BW.03.7   
  ∟    ◆   BW.03.7   
  ▶     ■    BW.03.8   
  ∟    ◆   BW.03.8   
  ▶     ■    BW.03.9   
  ∟    ◆   BW.03.9   
  ▶     ■    BW.04.1   
  ∟    ◆   BW.04.1   
  ▶     ■    BW.04.10   
  ∟    ◆   BW.04.10   
  ▶     ■    BW.04.2   
  ∟    ◆   BW.04.2   
  ▶     ■    BW.04.3   
  ∟    ◆   BW.04.3   
  ▶     ■    BW.04.4   
  ∟    ◆   BW.04.4   
  ▶     ■    BW.04.5   
  ∟    ◆   BW.04.5   
  ▶     ■    BW.04.6   
  ∟    ◆   BW.04.6   
  ▶     ■    BW.04.7   
  ∟    ◆   BW.04.7   
  ▶     ■    BW.04.8   
  ∟    ◆   BW.04.8   
  ▶     ■    BW.04.9   
  ∟    ◆   BW.04.9   
  ▶     ■    BW.05.1   
  ∟    ◆   BW.05.1   
  ▶     ■    BW.05.10   
  ∟    ◆   BW.05.10   
  ▶     ■    BW.05.2   
  ∟    ◆   BW.05.2   
  ▶     ■    BW.05.3   
  ∟    ◆   BW.05.3   
  ▶     ■    BW.05.4   
  ∟    ◆   BW.05.4   
  ▶     ■    BW.05.5   
  ∟    ◆   BW.05.5   
  ▶     ■    BW.05.6   
  ∟    ◆   BW.05.6   
  ▶     ■    BW.05.7   
  ∟    ◆   BW.05.7   
  ▶     ■    BW.05.8   
  ∟    ◆   BW.05.8   
  ▶     ■    BW.05.9   
  ∟    ◆   BW.05.9   
  ▶     ■    BW.06.1   
  ∟    ◆   BW.06.1   
  ▶     ■    BW.06.10   
  ∟    ◆   BW.06.10   
  ▶     ■    BW.06.2   
  ∟    ◆   BW.06.2   
  ▶     ■    BW.06.4   
  ∟    ◆   BW.06.4   
  ▶     ■    BW.06.5   
  ∟    ◆   BW.06.5   
  ▶     ■    BW.06.6   
  ∟    ◆   BW.06.6   
  ▶     ■    BW.06.7   
  ∟    ◆   BW.06.7   
  ▶     ■    BW.06.8   
  ∟    ◆   BW.06.8   
  ▶     ■    BW.06.9   
  ∟    ◆   BW.06.9   
  ▶     ■    BW.07.1   
  ∟    ◆   BW.07.1   
  ▶     ■    BW.07.10   
  ∟    ◆   BW.07.10   
  ▶     ■    BW.07.2   
  ∟    ◆   BW.07.2   
  ▶     ■    BW.07.3   
  ∟    ◆   BW.07.3   
  ▶     ■    BW.07.4   
  ∟    ◆   BW.07.4   
  ▶     ■    BW.07.5   
  ∟    ◆   BW.07.5   
  ▶     ■    BW.07.6   
  ∟    ◆   BW.07.6   
  ▶     ■    BW.07.7   
  ∟    ◆   BW.07.7   
  ▶     ■    BW.07.8   
  ∟    ◆   BW.07.8   
  ▶     ■    BW.07.9   
  ∟    ◆   BW.07.9   
  ▶     ■    BW.08.1   
  ∟    ◆   BW.08.1   
  ▶     ■    BW.08.10   
  ∟    ◆   BW.08.10   
  ▶     ■    BW.08.2   
  ∟    ◆   BW.08.2   
  ▶     ■    BW.08.3   
  ∟    ◆   BW.08.3   
  ▶     ■    BW.08.4   
  ∟    ◆   BW.08.4   
  ▶     ■    BW.08.5   
  ∟    ◆   BW.08.5   
  ▶     ■    BW.08.6   
  ∟    ◆   BW.08.6   
  ▶     ■    BW.08.7   
  ∟    ◆   BW.08.7   
  ▶     ■    BW.08.8   
  ∟    ◆   BW.08.8   
  ▶     ■    BW.08.9   
  ∟    ◆   BW.08.9   
  ▶     ■    BW.09.1   
  ∟    ◆   BW.09.1   
  ▶     ■    BW.09.10   
  ∟    ◆   BW.09.10   
  ▶     ■    BW.09.2   
  ∟    ◆   BW.09.2   
  ▶     ■    BW.09.3   
  ∟    ◆   BW.09.3   
  ▶     ■    BW.09.4   
  ∟    ◆   BW.09.4   
  ▶     ■    BW.09.5   
  ∟    ◆   BW.09.5   
  ▶     ■    BW.09.6   
  ∟    ◆   BW.09.6   
  ▶     ■    BW.09.7   
  ∟    ◆   BW.09.7   
  ▶     ■    BW.09.8   
  ∟    ◆   BW.09.8   
  ▶     ■    BW.09.9   
  ∟    ◆   BW.09.9   
  ▶     ■    BW.10.1   
  ∟    ◆   BW.10.1   
  ▶     ■    BW.10.10   
  ∟    ◆   BW.10.10   
  ▶     ■    BW.10.2   
  ∟    ◆   BW.10.2   
  ▶     ■    BW.10.4   
  ∟    ◆   BW.10.4   
  ▶     ■    BW.10.5   
  ∟    ◆   BW.10.5   
  ▶     ■    BW.10.6   
  ∟    ◆   BW.10.6   
  ▶     ■    BW.10.8   
  ∟    ◆   BW.10.8   
  ▶     ■    BW.10.9   
  ∟    ◆   BW.10.9   
  ▶     ■    01   
  ∟    ◆   BW.01.1   
  ∟    ◆   BW.01.10   
  ∟    ◆   BW.01.2   
  ∟    ◆   BW.01.3   
  ∟    ◆   BW.01.4   
  ∟    ◆   BW.01.5   
  ∟    ◆   BW.01.6   
  ∟    ◆   BW.01.7   
  ∟    ◆   BW.01.8   
  ∟    ◆   BW.01.9   
  ▶     ■    02   
  ∟    ◆   BW.02.1   
  ∟    ◆   BW.02.10   
  ∟    ◆   BW.02.2   
  ∟    ◆   BW.02.3   
  ∟    ◆   BW.02.4   
  ∟    ◆   BW.02.5   
  ∟    ◆   BW.02.6   
  ∟    ◆   BW.02.7   
  ∟    ◆   BW.02.8   
  ∟    ◆   BW.02.9   
  ▶     ■    03   
  ∟    ◆   BW.03.1   
  ∟    ◆   BW.03.10   
  ∟    ◆   BW.03.3   
  ∟    ◆   BW.03.4   
  ∟    ◆   BW.03.5   
  ∟    ◆   BW.03.6   
  ∟    ◆   BW.03.7   
  ∟    ◆   BW.03.8   
  ∟    ◆   BW.03.9   
  ▶     ■    04   
  ∟    ◆   BW.04.1   
  ∟    ◆   BW.04.10   
  ∟    ◆   BW.04.2   
  ∟    ◆   BW.04.3   
  ∟    ◆   BW.04.4   
  ∟    ◆   BW.04.5   
  ∟    ◆   BW.04.6   
  ∟    ◆   BW.04.7   
  ∟    ◆   BW.04.8   
  ∟    ◆   BW.04.9   
  ▶     ■    05   
  ∟    ◆   BW.05.1   
  ∟    ◆   BW.05.10   
  ∟    ◆   BW.05.2   
  ∟    ◆   BW.05.3   
  ∟    ◆   BW.05.4   
  ∟    ◆   BW.05.5   
  ∟    ◆   BW.05.6   
  ∟    ◆   BW.05.7   
  ∟    ◆   BW.05.8   
  ∟    ◆   BW.05.9   
  ▶     ■    06   
  ∟    ◆   BW.06.1   
  ∟    ◆   BW.06.10   
  ∟    ◆   BW.06.2   
  ∟    ◆   BW.06.4   
  ∟    ◆   BW.06.5   
  ∟    ◆   BW.06.6   
  ∟    ◆   BW.06.7   
  ∟    ◆   BW.06.8   
  ∟    ◆   BW.06.9   
  ▶     ■    07   
  ∟    ◆   BW.07.1   
  ∟    ◆   BW.07.10   
  ∟    ◆   BW.07.2   
  ∟    ◆   BW.07.3   
  ∟    ◆   BW.07.4   
  ∟    ◆   BW.07.5   
  ∟    ◆   BW.07.6   
  ∟    ◆   BW.07.7   
  ∟    ◆   BW.07.8   
  ∟    ◆   BW.07.9   
  ▶     ■    08   
  ∟    ◆   BW.08.1   
  ∟    ◆   BW.08.10   
  ∟    ◆   BW.08.2   
  ∟    ◆   BW.08.3   
  ∟    ◆   BW.08.4   
  ∟    ◆   BW.08.5   
  ∟    ◆   BW.08.6   
  ∟    ◆   BW.08.7   
  ∟    ◆   BW.08.8   
  ∟    ◆   BW.08.9   
  ▶     ■    09   
  ∟    ◆   BW.09.1   
  ∟    ◆   BW.09.10   
  ∟    ◆   BW.09.2   
  ∟    ◆   BW.09.3   
  ∟    ◆   BW.09.4   
  ∟    ◆   BW.09.5   
  ∟    ◆   BW.09.6   
  ∟    ◆   BW.09.7   
  ∟    ◆   BW.09.8   
  ∟    ◆   BW.09.9   
  ▶     ■    10   
  ∟    ◆   BW.10.1   
  ∟    ◆   BW.10.10   
  ∟    ◆   BW.10.2   
  ∟    ◆   BW.10.4   
  ∟    ◆   BW.10.5   
  ∟    ◆   BW.10.6   
  ∟    ◆   BW.10.8   
  ∟    ◆   BW.10.9   
  ▶     ■    1   
  ∟    ◆   BW.01.1   
  ∟    ◆   BW.02.1   
  ∟    ◆   BW.03.1   
  ∟    ◆   BW.04.1   
  ∟    ◆   BW.05.1   
  ∟    ◆   BW.06.1   
  ∟    ◆   BW.07.1   
  ∟    ◆   BW.08.1   
  ∟    ◆   BW.09.1   
  ∟    ◆   BW.10.1   
  ▶     ■    2   
  ∟    ◆   BW.01.2   
  ∟    ◆   BW.02.2   
  ∟    ◆   BW.04.2   
  ∟    ◆   BW.05.2   
  ∟    ◆   BW.06.2   
  ∟    ◆   BW.07.2   
  ∟    ◆   BW.08.2   
  ∟    ◆   BW.09.2   
  ∟    ◆   BW.10.2   
  ▶     ■    3   
  ∟    ◆   BW.01.3   
  ∟    ◆   BW.02.3   
  ∟    ◆   BW.03.3   
  ∟    ◆   BW.04.3   
  ∟    ◆   BW.05.3   
  ∟    ◆   BW.07.3   
  ∟    ◆   BW.08.3   
  ∟    ◆   BW.09.3   
  ▶     ■    4   
  ∟    ◆   BW.01.4   
  ∟    ◆   BW.02.4   
  ∟    ◆   BW.03.4   
  ∟    ◆   BW.04.4   
  ∟    ◆   BW.05.4   
  ∟    ◆   BW.06.4   
  ∟    ◆   BW.07.4   
  ∟    ◆   BW.08.4   
  ∟    ◆   BW.09.4   
  ∟    ◆   BW.10.4   
  ▶     ■    5   
  ∟    ◆   BW.01.5   
  ∟    ◆   BW.02.5   
  ∟    ◆   BW.03.5   
  ∟    ◆   BW.04.5   
  ∟    ◆   BW.05.5   
  ∟    ◆   BW.06.5   
  ∟    ◆   BW.07.5   
  ∟    ◆   BW.08.5   
  ∟    ◆   BW.09.5   
  ∟    ◆   BW.10.5   
  ▶     ■    6   
  ∟    ◆   BW.01.6   
  ∟    ◆   BW.02.6   
  ∟    ◆   BW.03.6   
  ∟    ◆   BW.04.6   
  ∟    ◆   BW.05.6   
  ∟    ◆   BW.06.6   
  ∟    ◆   BW.07.6   
  ∟    ◆   BW.08.6   
  ∟    ◆   BW.09.6   
  ∟    ◆   BW.10.6   
  ▶     ■    7   
  ∟    ◆   BW.01.7   
  ∟    ◆   BW.02.7   
  ∟    ◆   BW.03.7   
  ∟    ◆   BW.04.7   
  ∟    ◆   BW.05.7   
  ∟    ◆   BW.06.7   
  ∟    ◆   BW.07.7   
  ∟    ◆   BW.08.7   
  ∟    ◆   BW.09.7   
  ▶     ■    8   
  ∟    ◆   BW.01.8   
  ∟    ◆   BW.02.8   
  ∟    ◆   BW.03.8   
  ∟    ◆   BW.04.8   
  ∟    ◆   BW.05.8   
  ∟    ◆   BW.06.8   
  ∟    ◆   BW.07.8   
  ∟    ◆   BW.08.8   
  ∟    ◆   BW.09.8   
  ∟    ◆   BW.10.8   
  ▶     ■    9   
  ∟    ◆   BW.01.9   
  ∟    ◆   BW.02.9   
  ∟    ◆   BW.03.9   
  ∟    ◆   BW.04.9   
  ∟    ◆   BW.05.9   
  ∟    ◆   BW.06.9   
  ∟    ◆   BW.07.9   
  ∟    ◆   BW.08.9   
  ∟    ◆   BW.09.9   
  ∟    ◆   BW.10.9   
  ▶     ■    10   
  ∟    ◆   BW.01.10   
  ∟    ◆   BW.02.10   
  ∟    ◆   BW.03.10   
  ∟    ◆   BW.04.10   
  ∟    ◆   BW.05.10   
  ∟    ◆   BW.06.10   
  ∟    ◆   BW.07.10   
  ∟    ◆   BW.08.10   
  ∟    ◆   BW.09.10   
  ∟    ◆   BW.10.10   
  ▶     ■    ACATACGCGTAG   
  ∟    ◆   BW.03.5   
  ∟    ◆   BW.08.6   
  ▶     ■    ACGACTACAGAG   
  ∟    ◆   BW.02.9   
  ∟    ◆   BW.07.10   
  ▶     ■    ACGAGTGCGTAG   
  ∟    ◆   BW.01.1   
  ∟    ◆   BW.06.1   
  ▶     ■    ACGCGAGTATAG   
  ∟    ◆   BW.03.6   
  ∟    ◆   BW.08.7   
  ▶     ■    ACGCGATCGAAG   
  ∟    ◆   BW.05.7   
  ∟    ◆   BW.10.8   
  ▶     ■    ACGCTCGACAAG   
  ∟    ◆   BW.01.2   
  ∟    ◆   BW.06.2   
  ▶     ■    ACTACTATGTAG   
  ∟    ◆   BW.03.7   
  ∟    ◆   BW.08.8   
  ▶     ■    ACTAGCAGTAAG   
  ∟    ◆   BW.05.8   
  ∟    ◆   BW.10.9   
  ▶     ■    ACTGTACAGTAG   
  ∟    ◆   BW.03.8   
  ∟    ◆   BW.08.9   
  ▶     ■    AGACGCACTCAG   
  ∟    ◆   BW.01.3   
  ∟    ◆   BW.06.4   
  ▶     ■    AGACTATACTAG   
  ∟    ◆   BW.03.9   
  ∟    ◆   BW.08.10   
  ▶     ■    AGCACTGTAGAG   
  ∟    ◆   BW.01.4   
  ∟    ◆   BW.06.5   
  ▶     ■    AGCGTCGTCTAG   
  ∟    ◆   BW.03.10   
  ∟    ◆   BW.09.1   
  ▶     ■    AGCTCACGTAAG   
  ∟    ◆   BW.05.9   
  ∟    ◆   BW.10.10   
  ▶     ■    AGTACGCTATAG   
  ∟    ◆   BW.04.1   
  ∟    ◆   BW.09.2   
  ▶     ■    AGTATACATAAG   
  ∟    ◆   BW.05.10   
  ▶     ■    ATACGACGTAAG   
  ∟    ◆   BW.02.4   
  ∟    ◆   BW.07.5   
  ▶     ■    ATAGAGTACTAG   
  ∟    ◆   BW.04.2   
  ∟    ◆   BW.09.3   
  ▶     ■    ATATCGCGAGAG   
  ∟    ◆   BW.01.6   
  ∟    ◆   BW.06.7   
  ▶     ■    ATCAGACACGAG   
  ∟    ◆   BW.01.5   
  ∟    ◆   BW.06.6   
  ▶     ■    CACGCTACGTAG   
  ∟    ◆   BW.04.3   
  ∟    ◆   BW.09.4   
  ▶     ■    CAGTAGACGTAG   
  ∟    ◆   BW.04.4   
  ∟    ◆   BW.09.5   
  ▶     ■    CATAGTAGTGAG   
  ∟    ◆   BW.02.2   
  ∟    ◆   BW.07.3   
  ▶     ■    CGACGTGACTAG   
  ∟    ◆   BW.04.5   
  ∟    ◆   BW.09.6   
  ▶     ■    CGAGAGATACAG   
  ∟    ◆   BW.02.3   
  ∟    ◆   BW.07.4   
  ▶     ■    CGTAGACTAGAG   
  ∟    ◆   BW.02.10   
  ∟    ◆   BW.08.1   
  ▶     ■    CGTCTAGTACAG   
  ∟    ◆   BW.02.6   
  ∟    ◆   BW.07.7   
  ▶     ■    CGTGTCTCTAAG   
  ∟    ◆   BW.01.7   
  ∟    ◆   BW.06.8   
  ▶     ■    CTCGCGTGTCAG   
  ∟    ◆   BW.01.8   
  ∟    ◆   BW.06.9   
  ▶     ■    TACACACACTAG   
  ∟    ◆   BW.04.6   
  ∟    ◆   BW.09.7   
  ▶     ■    TACACGTGATAG   
  ∟    ◆   BW.04.7   
  ∟    ◆   BW.09.8   
  ▶     ■    TACAGATCGTAG   
  ∟    ◆   BW.04.8   
  ∟    ◆   BW.09.9   
  ▶     ■    TACGAGTATGAG   
  ∟    ◆   BW.03.1   
  ∟    ◆   BW.08.2   
  ▶     ■    TACGCTGTCTAG   
  ∟    ◆   BW.04.9   
  ∟    ◆   BW.09.10   
  ▶     ■    TACTCTCGTGAG   
  ∟    ◆   BW.08.3   
  ▶     ■    TAGAGACGAGAG   
  ∟    ◆   BW.03.3   
  ∟    ◆   BW.08.4   
  ▶     ■    TAGTATCAGCAG   
  ∟    ◆   BW.01.9   
  ∟    ◆   BW.06.10   
  ▶     ■    TAGTGTAGATAG   
  ∟    ◆   BW.04.10   
  ∟    ◆   BW.10.1   
  ▶     ■    TCACGTACTAAG   
  ∟    ◆   BW.02.5   
  ∟    ◆   BW.07.6   
  ▶     ■    TCGATCACGTAG   
  ∟    ◆   BW.05.1   
  ∟    ◆   BW.10.2   
  ▶     ■    TCGCACTAGTAG   
  ∟    ◆   BW.05.2   
  ▶     ■    TCGTCGCTCGAG   
  ∟    ◆   BW.03.4   
  ∟    ◆   BW.08.5   
  ▶     ■    TCTACGTAGCAG   
  ∟    ◆   BW.02.7   
  ∟    ◆   BW.07.8   
  ▶     ■    TCTAGCGACTAG   
  ∟    ◆   BW.05.3   
  ∟    ◆   BW.10.4   
  ▶     ■    TCTATACTATAG   
  ∟    ◆   BW.05.4   
  ∟    ◆   BW.10.5   
  ▶     ■    TCTCTATGCGAG   
  ∟    ◆   BW.01.10   
  ∟    ◆   BW.07.1   
  ▶     ■    TGACGTATGTAG   
  ∟    ◆   BW.05.5   
  ∟    ◆   BW.10.6   
  ▶     ■    TGATACGTCTAG   
  ∟    ◆   BW.02.1   
  ∟    ◆   BW.07.2   
  ▶     ■    TGTACTACTCAG   
  ∟    ◆   BW.02.8   
  ∟    ◆   BW.07.9   
  ▶     ■    TGTGAGTAGTAG   
  ∟    ◆   BW.05.6   
  ▶     ■    BW-01.1.Lib_L_27F-001   
  ∟    ◆   BW.01.1   
  ▶     ■    BW-01.10.Lib_L_27F-010   
  ∟    ◆   BW.01.10   
  ▶     ■    BW-01.2.Lib_L_27F-002   
  ∟    ◆   BW.01.2   
  ▶     ■    BW-01.3.Lib_L_27F-003   
  ∟    ◆   BW.01.3   
  ▶     ■    BW-01.4.Lib_L_27F-004   
  ∟    ◆   BW.01.4   
  ▶     ■    BW-01.5.Lib_L_27F-005   
  ∟    ◆   BW.01.5   
  ▶     ■    BW-01.6.Lib_L_27F-006   
  ∟    ◆   BW.01.6   
  ▶     ■    BW-01.7.Lib_L_27F-007   
  ∟    ◆   BW.01.7   
  ▶     ■    BW-01.8.Lib_L_27F-008   
  ∟    ◆   BW.01.8   
  ▶     ■    BW-01.9.Lib_L_27F-009   
  ∟    ◆   BW.01.9   
  ▶     ■    BW-02.1.Lib_L_27F-011   
  ∟    ◆   BW.02.1   
  ▶     ■    BW-02.10.Lib_L_27F-021   
  ∟    ◆   BW.02.10   
  ▶     ■    BW-02.2.Lib_L_27F-013   
  ∟    ◆   BW.02.2   
  ▶     ■    BW-02.3.Lib_L_27F-014   
  ∟    ◆   BW.02.3   
  ▶     ■    BW-02.4.Lib_L_27F-015   
  ∟    ◆   BW.02.4   
  ▶     ■    BW-02.5.Lib_L_27F-016   
  ∟    ◆   BW.02.5   
  ▶     ■    BW-02.6.Lib_L_27F-017   
  ∟    ◆   BW.02.6   
  ▶     ■    BW-02.7.Lib_L_27F-018   
  ∟    ◆   BW.02.7   
  ▶     ■    BW-02.8.Lib_L_27F-019   
  ∟    ◆   BW.02.8   
  ▶     ■    BW-02.9.Lib_L_27F-020   
  ∟    ◆   BW.02.9   
  ▶     ■    BW-03.1.Lib_L_27F-022   
  ∟    ◆   BW.03.1   
  ▶     ■    BW-03.10.Lib_L_27F-031   
  ∟    ◆   BW.03.10   
  ▶     ■    BW-03.3.Lib_L_27F-024   
  ∟    ◆   BW.03.3   
  ▶     ■    BW-03.4.Lib_L_27F-025   
  ∟    ◆   BW.03.4   
  ▶     ■    BW-03.5.Lib_L_27F-026   
  ∟    ◆   BW.03.5   
  ▶     ■    BW-03.6.Lib_L_27F-027   
  ∟    ◆   BW.03.6   
  ▶     ■    BW-03.7.Lib_L_27F-028   
  ∟    ◆   BW.03.7   
  ▶     ■    BW-03.8.Lib_L_27F-029   
  ∟    ◆   BW.03.8   
  ▶     ■    BW-03.9.Lib_L_27F-030   
  ∟    ◆   BW.03.9   
  ▶     ■    BW-04.1.Lib_L_27F-032   
  ∟    ◆   BW.04.1   
  ▶     ■    BW-04.10.Lib_L_27F-041   
  ∟    ◆   BW.04.10   
  ▶     ■    BW-04.2.Lib_L_27F-033   
  ∟    ◆   BW.04.2   
  ▶     ■    BW-04.3.Lib_L_27F-034   
  ∟    ◆   BW.04.3   
  ▶     ■    BW-04.4.Lib_L_27F-035   
  ∟    ◆   BW.04.4   
  ▶     ■    BW-04.5.Lib_L_27F-036   
  ∟    ◆   BW.04.5   
  ▶     ■    BW-04.6.Lib_L_27F-037   
  ∟    ◆   BW.04.6   
  ▶     ■    BW-04.7.Lib_L_27F-038   
  ∟    ◆   BW.04.7   
  ▶     ■    BW-04.8.Lib_L_27F-039   
  ∟    ◆   BW.04.8   
  ▶     ■    BW-04.9.Lib_L_27F-040   
  ∟    ◆   BW.04.9   
  ▶     ■    BW-05.1.Lib_L_27F-042   
  ∟    ◆   BW.05.1   
  ▶     ■    BW-05.10.Lib_L_27F-052   
  ∟    ◆   BW.05.10   
  ▶     ■    BW-05.2.Lib_L_27F-043   
  ∟    ◆   BW.05.2   
  ▶     ■    BW-05.3.Lib_L_27F-044   
  ∟    ◆   BW.05.3   
  ▶     ■    BW-05.4.Lib_L_27F-045   
  ∟    ◆   BW.05.4   
  ▶     ■    BW-05.5.Lib_L_27F-046   
  ∟    ◆   BW.05.5   
  ▶     ■    BW-05.6.Lib_L_27F-047   
  ∟    ◆   BW.05.6   
  ▶     ■    BW-05.7.Lib_L_27F-049   
  ∟    ◆   BW.05.7   
  ▶     ■    BW-05.8.Lib_L_27F-050   
  ∟    ◆   BW.05.8   
  ▶     ■    BW-05.9.Lib_L_27F-051   
  ∟    ◆   BW.05.9   
  ▶     ■    BW-06.1.Lib_L_27F-001   
  ∟    ◆   BW.06.1   
  ▶     ■    BW-06.10.Lib_L_27F-009   
  ∟    ◆   BW.06.10   
  ▶     ■    BW-06.2.Lib_L_27F-002   
  ∟    ◆   BW.06.2   
  ▶     ■    BW-06.4.Lib_L_27F-003   
  ∟    ◆   BW.06.4   
  ▶     ■    BW-06.5.Lib_L_27F-004   
  ∟    ◆   BW.06.5   
  ▶     ■    BW-06.6.Lib_L_27F-005   
  ∟    ◆   BW.06.6   
  ▶     ■    BW-06.7.Lib_L_27F-006   
  ∟    ◆   BW.06.7   
  ▶     ■    BW-06.8.Lib_L_27F-007   
  ∟    ◆   BW.06.8   
  ▶     ■    BW-06.9.Lib_L_27F-008   
  ∟    ◆   BW.06.9   
  ▶     ■    BW-07.1.Lib_L_27F-010   
  ∟    ◆   BW.07.1   
  ▶     ■    BW-07.10.Lib_L_27F-020   
  ∟    ◆   BW.07.10   
  ▶     ■    BW-07.2.Lib_L_27F-011   
  ∟    ◆   BW.07.2   
  ▶     ■    BW-07.3.Lib_L_27F-013   
  ∟    ◆   BW.07.3   
  ▶     ■    BW-07.4.Lib_L_27F-014   
  ∟    ◆   BW.07.4   
  ▶     ■    BW-07.5.Lib_L_27F-015   
  ∟    ◆   BW.07.5   
  ▶     ■    BW-07.6.Lib_L_27F-016   
  ∟    ◆   BW.07.6   
  ▶     ■    BW-07.7.Lib_L_27F-017   
  ∟    ◆   BW.07.7   
  ▶     ■    BW-07.8.Lib_L_27F-018   
  ∟    ◆   BW.07.8   
  ▶     ■    BW-07.9.Lib_L_27F-019   
  ∟    ◆   BW.07.9   
  ▶     ■    BW-08.1.Lib_L_27F-021   
  ∟    ◆   BW.08.1   
  ▶     ■    BW-08.10.Lib_L_27F-030   
  ∟    ◆   BW.08.10   
  ▶     ■    BW-08.2.Lib_L_27F-022   
  ∟    ◆   BW.08.2   
  ▶     ■    BW-08.3.Lib_L_27F-023   
  ∟    ◆   BW.08.3   
  ▶     ■    BW-08.4.Lib_L_27F-024   
  ∟    ◆   BW.08.4   
  ▶     ■    BW-08.5.Lib_L_27F-025   
  ∟    ◆   BW.08.5   
  ▶     ■    BW-08.6.Lib_L_27F-026   
  ∟    ◆   BW.08.6   
  ▶     ■    BW-08.7.Lib_L_27F-027   
  ∟    ◆   BW.08.7   
  ▶     ■    BW-08.8.Lib_L_27F-028   
  ∟    ◆   BW.08.8   
  ▶     ■    BW-08.9.Lib_L_27F-029   
  ∟    ◆   BW.08.9   
  ▶     ■    BW-09.1.Lib_L_27F-031   
  ∟    ◆   BW.09.1   
  ▶     ■    BW-09.10.Lib_L_27F-040   
  ∟    ◆   BW.09.10   
  ▶     ■    BW-09.2.Lib_L_27F-032   
  ∟    ◆   BW.09.2   
  ▶     ■    BW-09.3.Lib_L_27F-033   
  ∟    ◆   BW.09.3   
  ▶     ■    BW-09.4.Lib_L_27F-034   
  ∟    ◆   BW.09.4   
  ▶     ■    BW-09.5.Lib_L_27F-035   
  ∟    ◆   BW.09.5   
  ▶     ■    BW-09.6.Lib_L_27F-036   
  ∟    ◆   BW.09.6   
  ▶     ■    BW-09.7.Lib_L_27F-037   
  ∟    ◆   BW.09.7   
  ▶     ■    BW-09.8.Lib_L_27F-038   
  ∟    ◆   BW.09.8   
  ▶     ■    BW-09.9.Lib_L_27F-039   
  ∟    ◆   BW.09.9   
  ▶     ■    BW-10.1.Lib_L_27F-041   
  ∟    ◆   BW.10.1   
  ▶     ■    BW-10.10.Lib_L_27F-051   
  ∟    ◆   BW.10.10   
  ▶     ■    BW-10.2.Lib_L_27F-042   
  ∟    ◆   BW.10.2   
  ▶     ■    BW-10.4.Lib_L_27F-044   
  ∟    ◆   BW.10.4   
  ▶     ■    BW-10.5.Lib_L_27F-045   
  ∟    ◆   BW.10.5   
  ▶     ■    BW-10.6.Lib_L_27F-046   
  ∟    ◆   BW.10.6   
  ▶     ■    BW-10.8.Lib_L_27F-049   
  ∟    ◆   BW.10.8   
  ▶     ■    BW-10.9.Lib_L_27F-050   
  ∟    ◆   BW.10.9   
  ▶     ■    After   
  ∟    ◆   BW.01.10   
  ∟    ◆   BW.01.9   
  ∟    ◆   BW.02.10   
  ∟    ◆   BW.02.9   
  ∟    ◆   BW.03.10   
  ∟    ◆   BW.03.9   
  ∟    ◆   BW.04.10   
  ∟    ◆   BW.04.9   
  ∟    ◆   BW.05.10   
  ∟    ◆   BW.05.9   
  ∟    ◆   BW.06.10   
  ∟    ◆   BW.06.9   
  ∟    ◆   BW.07.10   
  ∟    ◆   BW.07.9   
  ∟    ◆   BW.08.10   
  ∟    ◆   BW.08.9   
  ∟    ◆   BW.09.10   
  ∟    ◆   BW.09.9   
  ∟    ◆   BW.10.10   
  ∟    ◆   BW.10.9   
  ▶     ■    Before   
  ∟    ◆   BW.01.1   
  ∟    ◆   BW.01.2   
  ∟    ◆   BW.01.3   
  ∟    ◆   BW.02.1   
  ∟    ◆   BW.02.2   
  ∟    ◆   BW.02.3   
  ∟    ◆   BW.03.1   
  ∟    ◆   BW.03.3   
  ∟    ◆   BW.04.1   
  ∟    ◆   BW.04.2   
  ∟    ◆   BW.04.3   
  ∟    ◆   BW.05.1   
  ∟    ◆   BW.05.2   
  ∟    ◆   BW.05.3   
  ∟    ◆   BW.06.1   
  ∟    ◆   BW.06.2   
  ∟    ◆   BW.07.1   
  ∟    ◆   BW.07.2   
  ∟    ◆   BW.07.3   
  ∟    ◆   BW.08.1   
  ∟    ◆   BW.08.2   
  ∟    ◆   BW.08.3   
  ∟    ◆   BW.09.1   
  ∟    ◆   BW.09.2   
  ∟    ◆   BW.09.3   
  ∟    ◆   BW.10.1   
  ∟    ◆   BW.10.2   
  ▶     ■    During   
  ∟    ◆   BW.01.4   
  ∟    ◆   BW.01.5   
  ∟    ◆   BW.01.6   
  ∟    ◆   BW.01.7   
  ∟    ◆   BW.01.8   
  ∟    ◆   BW.02.4   
  ∟    ◆   BW.02.5   
  ∟    ◆   BW.02.6   
  ∟    ◆   BW.02.7   
  ∟    ◆   BW.02.8   
  ∟    ◆   BW.03.4   
  ∟    ◆   BW.03.5   
  ∟    ◆   BW.03.6   
  ∟    ◆   BW.03.7   
  ∟    ◆   BW.03.8   
  ∟    ◆   BW.04.4   
  ∟    ◆   BW.04.5   
  ∟    ◆   BW.04.6   
  ∟    ◆   BW.04.7   
  ∟    ◆   BW.04.8   
  ∟    ◆   BW.05.4   
  ∟    ◆   BW.05.5   
  ∟    ◆   BW.05.6   
  ∟    ◆   BW.05.7   
  ∟    ◆   BW.05.8   
  ∟    ◆   BW.06.4   
  ∟    ◆   BW.06.5   
  ∟    ◆   BW.06.6   
  ∟    ◆   BW.06.7   
  ∟    ◆   BW.06.8   
  ∟    ◆   BW.07.4   
  ∟    ◆   BW.07.5   
  ∟    ◆   BW.07.6   
  ∟    ◆   BW.07.7   
  ∟    ◆   BW.07.8   
  ∟    ◆   BW.08.4   
  ∟    ◆   BW.08.5   
  ∟    ◆   BW.08.6   
  ∟    ◆   BW.08.7   
  ∟    ◆   BW.08.8   
  ∟    ◆   BW.09.4   
  ∟    ◆   BW.09.5   
  ∟    ◆   BW.09.6   
  ∟    ◆   BW.09.7   
  ∟    ◆   BW.09.8   
  ∟    ◆   BW.10.4   
  ∟    ◆   BW.10.5   
  ∟    ◆   BW.10.6   
  ∟    ◆   BW.10.8   
  ▶     ■    Lib_L_27F-001   
  ∟    ◆   BW.01.1   
  ∟    ◆   BW.06.1   
  ▶     ■    Lib_L_27F-002   
  ∟    ◆   BW.01.2   
  ∟    ◆   BW.06.2   
  ▶     ■    Lib_L_27F-003   
  ∟    ◆   BW.01.3   
  ∟    ◆   BW.06.4   
  ▶     ■    Lib_L_27F-004   
  ∟    ◆   BW.01.4   
  ∟    ◆   BW.06.5   
  ▶     ■    Lib_L_27F-005   
  ∟    ◆   BW.01.5   
  ∟    ◆   BW.06.6   
  ▶     ■    Lib_L_27F-006   
  ∟    ◆   BW.01.6   
  ∟    ◆   BW.06.7   
  ▶     ■    Lib_L_27F-007   
  ∟    ◆   BW.01.7   
  ∟    ◆   BW.06.8   
  ▶     ■    Lib_L_27F-008   
  ∟    ◆   BW.01.8   
  ∟    ◆   BW.06.9   
  ▶     ■    Lib_L_27F-009   
  ∟    ◆   BW.01.9   
  ∟    ◆   BW.06.10   
  ▶     ■    Lib_L_27F-010   
  ∟    ◆   BW.01.10   
  ∟    ◆   BW.07.1   
  ▶     ■    Lib_L_27F-011   
  ∟    ◆   BW.02.1   
  ∟    ◆   BW.07.2   
  ▶     ■    Lib_L_27F-013   
  ∟    ◆   BW.02.2   
  ∟    ◆   BW.07.3   
  ▶     ■    Lib_L_27F-014   
  ∟    ◆   BW.02.3   
  ∟    ◆   BW.07.4   
  ▶     ■    Lib_L_27F-015   
  ∟    ◆   BW.02.4   
  ∟    ◆   BW.07.5   
  ▶     ■    Lib_L_27F-016   
  ∟    ◆   BW.02.5   
  ∟    ◆   BW.07.6   
  ▶     ■    Lib_L_27F-017   
  ∟    ◆   BW.02.6   
  ∟    ◆   BW.07.7   
  ▶     ■    Lib_L_27F-018   
  ∟    ◆   BW.02.7   
  ∟    ◆   BW.07.8   
  ▶     ■    Lib_L_27F-019   
  ∟    ◆   BW.02.8   
  ∟    ◆   BW.07.9   
  ▶     ■    Lib_L_27F-020   
  ∟    ◆   BW.02.9   
  ∟    ◆   BW.07.10   
  ▶     ■    Lib_L_27F-021   
  ∟    ◆   BW.02.10   
  ∟    ◆   BW.08.1   
  ▶     ■    Lib_L_27F-022   
  ∟    ◆   BW.03.1   
  ∟    ◆   BW.08.2   
  ▶     ■    Lib_L_27F-023   
  ∟    ◆   BW.08.3   
  ▶     ■    Lib_L_27F-024   
  ∟    ◆   BW.03.3   
  ∟    ◆   BW.08.4   
  ▶     ■    Lib_L_27F-025   
  ∟    ◆   BW.03.4   
  ∟    ◆   BW.08.5   
  ▶     ■    Lib_L_27F-026   
  ∟    ◆   BW.03.5   
  ∟    ◆   BW.08.6   
  ▶     ■    Lib_L_27F-027   
  ∟    ◆   BW.03.6   
  ∟    ◆   BW.08.7   
  ▶     ■    Lib_L_27F-028   
  ∟    ◆   BW.03.7   
  ∟    ◆   BW.08.8   
  ▶     ■    Lib_L_27F-029   
  ∟    ◆   BW.03.8   
  ∟    ◆   BW.08.9   
  ▶     ■    Lib_L_27F-030   
  ∟    ◆   BW.03.9   
  ∟    ◆   BW.08.10   
  ▶     ■    Lib_L_27F-031   
  ∟    ◆   BW.03.10   
  ∟    ◆   BW.09.1   
  ▶     ■    Lib_L_27F-032   
  ∟    ◆   BW.04.1   
  ∟    ◆   BW.09.2   
  ▶     ■    Lib_L_27F-033   
  ∟    ◆   BW.04.2   
  ∟    ◆   BW.09.3   
  ▶     ■    Lib_L_27F-034   
  ∟    ◆   BW.04.3   
  ∟    ◆   BW.09.4   
  ▶     ■    Lib_L_27F-035   
  ∟    ◆   BW.04.4   
  ∟    ◆   BW.09.5   
  ▶     ■    Lib_L_27F-036   
  ∟    ◆   BW.04.5   
  ∟    ◆   BW.09.6   
  ▶     ■    Lib_L_27F-037   
  ∟    ◆   BW.04.6   
  ∟    ◆   BW.09.7   
  ▶     ■    Lib_L_27F-038   
  ∟    ◆   BW.04.7   
  ∟    ◆   BW.09.8   
  ▶     ■    Lib_L_27F-039   
  ∟    ◆   BW.04.8   
  ∟    ◆   BW.09.9   
  ▶     ■    Lib_L_27F-040   
  ∟    ◆   BW.04.9   
  ∟    ◆   BW.09.10   
  ▶     ■    Lib_L_27F-041   
  ∟    ◆   BW.04.10   
  ∟    ◆   BW.10.1   
  ▶     ■    Lib_L_27F-042   
  ∟    ◆   BW.05.1   
  ∟    ◆   BW.10.2   
  ▶     ■    Lib_L_27F-043   
  ∟    ◆   BW.05.2   
  ▶     ■    Lib_L_27F-044   
  ∟    ◆   BW.05.3   
  ∟    ◆   BW.10.4   
  ▶     ■    Lib_L_27F-045   
  ∟    ◆   BW.05.4   
  ∟    ◆   BW.10.5   
  ▶     ■    Lib_L_27F-046   
  ∟    ◆   BW.05.5   
  ∟    ◆   BW.10.6   
  ▶     ■    Lib_L_27F-047   
  ∟    ◆   BW.05.6   
  ▶     ■    Lib_L_27F-049   
  ∟    ◆   BW.05.7   
  ∟    ◆   BW.10.8   
  ▶     ■    Lib_L_27F-050   
  ∟    ◆   BW.05.8   
  ∟    ◆   BW.10.9   
  ▶     ■    Lib_L_27F-051   
  ∟    ◆   BW.05.9   
  ∟    ◆   BW.10.10   
  ▶     ■    Lib_L_27F-052   
  ∟    ◆   BW.05.10   
  ▶     ■    BW.01.1   
  ∟    ◆   BW.01.1   
  ▶     ■    BW.01.10   
  ∟    ◆   BW.01.10   
  ▶     ■    BW.01.2   
  ∟    ◆   BW.01.2   
  ▶     ■    BW.01.3   
  ∟    ◆   BW.01.3   
  ▶     ■    BW.01.4   
  ∟    ◆   BW.01.4   
  ▶     ■    BW.01.5   
  ∟    ◆   BW.01.5   
  ▶     ■    BW.01.6   
  ∟    ◆   BW.01.6   
  ▶     ■    BW.01.7   
  ∟    ◆   BW.01.7   
  ▶     ■    BW.01.8   
  ∟    ◆   BW.01.8   
  ▶     ■    BW.01.9   
  ∟    ◆   BW.01.9   
  ▶     ■    BW.02.1   
  ∟    ◆   BW.02.1   
  ▶     ■    BW.02.10   
  ∟    ◆   BW.02.10   
  ▶     ■    BW.02.2   
  ∟    ◆   BW.02.2   
  ▶     ■    BW.02.3   
  ∟    ◆   BW.02.3   
  ▶     ■    BW.02.4   
  ∟    ◆   BW.02.4   
  ▶     ■    BW.02.5   
  ∟    ◆   BW.02.5   
  ▶     ■    BW.02.6   
  ∟    ◆   BW.02.6   
  ▶     ■    BW.02.7   
  ∟    ◆   BW.02.7   
  ▶     ■    BW.02.8   
  ∟    ◆   BW.02.8   
  ▶     ■    BW.02.9   
  ∟    ◆   BW.02.9   
  ▶     ■    BW.03.1   
  ∟    ◆   BW.03.1   
  ▶     ■    BW.03.10   
  ∟    ◆   BW.03.10   
  ▶     ■    BW.03.3   
  ∟    ◆   BW.03.3   
  ▶     ■    BW.03.4   
  ∟    ◆   BW.03.4   
  ▶     ■    BW.03.5   
  ∟    ◆   BW.03.5   
  ▶     ■    BW.03.6   
  ∟    ◆   BW.03.6   
  ▶     ■    BW.03.7   
  ∟    ◆   BW.03.7   
  ▶     ■    BW.03.8   
  ∟    ◆   BW.03.8   
  ▶     ■    BW.03.9   
  ∟    ◆   BW.03.9   
  ▶     ■    BW.04.1   
  ∟    ◆   BW.04.1   
  ▶     ■    BW.04.10   
  ∟    ◆   BW.04.10   
  ▶     ■    BW.04.2   
  ∟    ◆   BW.04.2   
  ▶     ■    BW.04.3   
  ∟    ◆   BW.04.3   
  ▶     ■    BW.04.4   
  ∟    ◆   BW.04.4   
  ▶     ■    BW.04.5   
  ∟    ◆   BW.04.5   
  ▶     ■    BW.04.6   
  ∟    ◆   BW.04.6   
  ▶     ■    BW.04.7   
  ∟    ◆   BW.04.7   
  ▶     ■    BW.04.8   
  ∟    ◆   BW.04.8   
  ▶     ■    BW.04.9   
  ∟    ◆   BW.04.9   
  ▶     ■    BW.05.1   
  ∟    ◆   BW.05.1   
  ▶     ■    BW.05.10   
  ∟    ◆   BW.05.10   
  ▶     ■    BW.05.2   
  ∟    ◆   BW.05.2   
  ▶     ■    BW.05.3   
  ∟    ◆   BW.05.3   
  ▶     ■    BW.05.4   
  ∟    ◆   BW.05.4   
  ▶     ■    BW.05.5   
  ∟    ◆   BW.05.5   
  ▶     ■    BW.05.6   
  ∟    ◆   BW.05.6   
  ▶     ■    BW.05.7   
  ∟    ◆   BW.05.7   
  ▶     ■    BW.05.8   
  ∟    ◆   BW.05.8   
  ▶     ■    BW.05.9   
  ∟    ◆   BW.05.9   
  ▶     ■    BW.06.1   
  ∟    ◆   BW.06.1   
  ▶     ■    BW.06.10   
  ∟    ◆   BW.06.10   
  ▶     ■    BW.06.2   
  ∟    ◆   BW.06.2   
  ▶     ■    BW.06.4   
  ∟    ◆   BW.06.4   
  ▶     ■    BW.06.5   
  ∟    ◆   BW.06.5   
  ▶     ■    BW.06.6   
  ∟    ◆   BW.06.6   
  ▶     ■    BW.06.7   
  ∟    ◆   BW.06.7   
  ▶     ■    BW.06.8   
  ∟    ◆   BW.06.8   
  ▶     ■    BW.06.9   
  ∟    ◆   BW.06.9   
  ▶     ■    BW.07.1   
  ∟    ◆   BW.07.1   
  ▶     ■    BW.07.10   
  ∟    ◆   BW.07.10   
  ▶     ■    BW.07.2   
  ∟    ◆   BW.07.2   
  ▶     ■    BW.07.3   
  ∟    ◆   BW.07.3   
  ▶     ■    BW.07.4   
  ∟    ◆   BW.07.4   
  ▶     ■    BW.07.5   
  ∟    ◆   BW.07.5   
  ▶     ■    BW.07.6   
  ∟    ◆   BW.07.6   
  ▶     ■    BW.07.7   
  ∟    ◆   BW.07.7   
  ▶     ■    BW.07.8   
  ∟    ◆   BW.07.8   
  ▶     ■    BW.07.9   
  ∟    ◆   BW.07.9   
  ▶     ■    BW.08.1   
  ∟    ◆   BW.08.1   
  ▶     ■    BW.08.10   
  ∟    ◆   BW.08.10   
  ▶     ■    BW.08.2   
  ∟    ◆   BW.08.2   
  ▶     ■    BW.08.3   
  ∟    ◆   BW.08.3   
  ▶     ■    BW.08.4   
  ∟    ◆   BW.08.4   
  ▶     ■    BW.08.5   
  ∟    ◆   BW.08.5   
  ▶     ■    BW.08.6   
  ∟    ◆   BW.08.6   
  ▶     ■    BW.08.7   
  ∟    ◆   BW.08.7   
  ▶     ■    BW.08.8   
  ∟    ◆   BW.08.8   
  ▶     ■    BW.08.9   
  ∟    ◆   BW.08.9   
  ▶     ■    BW.09.1   
  ∟    ◆   BW.09.1   
  ▶     ■    BW.09.10   
  ∟    ◆   BW.09.10   
  ▶     ■    BW.09.2   
  ∟    ◆   BW.09.2   
  ▶     ■    BW.09.3   
  ∟    ◆   BW.09.3   
  ▶     ■    BW.09.4   
  ∟    ◆   BW.09.4   
  ▶     ■    BW.09.5   
  ∟    ◆   BW.09.5   
  ▶     ■    BW.09.6   
  ∟    ◆   BW.09.6   
  ▶     ■    BW.09.7   
  ∟    ◆   BW.09.7   
  ▶     ■    BW.09.8   
  ∟    ◆   BW.09.8   
  ▶     ■    BW.09.9   
  ∟    ◆   BW.09.9   
  ▶     ■    BW.10.1   
  ∟    ◆   BW.10.1   
  ▶     ■    BW.10.10   
  ∟    ◆   BW.10.10   
  ▶     ■    BW.10.2   
  ∟    ◆   BW.10.2   
  ▶     ■    BW.10.4   
  ∟    ◆   BW.10.4   
  ▶     ■    BW.10.5   
  ∟    ◆   BW.10.5   
  ▶     ■    BW.10.6   
  ∟    ◆   BW.10.6   
  ▶     ■    BW.10.8   
  ∟    ◆   BW.10.8   
  ▶     ■    BW.10.9   
  ∟    ◆   BW.10.9   
  ▶     ■    01   
  ∟    ◆   BW.01.1   
  ∟    ◆   BW.01.10   
  ∟    ◆   BW.01.2   
  ∟    ◆   BW.01.3   
  ∟    ◆   BW.01.4   
  ∟    ◆   BW.01.5   
  ∟    ◆   BW.01.6   
  ∟    ◆   BW.01.7   
  ∟    ◆   BW.01.8   
  ∟    ◆   BW.01.9   
  ▶     ■    02   
  ∟    ◆   BW.02.1   
  ∟    ◆   BW.02.10   
  ∟    ◆   BW.02.2   
  ∟    ◆   BW.02.3   
  ∟    ◆   BW.02.4   
  ∟    ◆   BW.02.5   
  ∟    ◆   BW.02.6   
  ∟    ◆   BW.02.7   
  ∟    ◆   BW.02.8   
  ∟    ◆   BW.02.9   
  ▶     ■    03   
  ∟    ◆   BW.03.1   
  ∟    ◆   BW.03.10   
  ∟    ◆   BW.03.3   
  ∟    ◆   BW.03.4   
  ∟    ◆   BW.03.5   
  ∟    ◆   BW.03.6   
  ∟    ◆   BW.03.7   
  ∟    ◆   BW.03.8   
  ∟    ◆   BW.03.9   
  ▶     ■    04   
  ∟    ◆   BW.04.1   
  ∟    ◆   BW.04.10   
  ∟    ◆   BW.04.2   
  ∟    ◆   BW.04.3   
  ∟    ◆   BW.04.4   
  ∟    ◆   BW.04.5   
  ∟    ◆   BW.04.6   
  ∟    ◆   BW.04.7   
  ∟    ◆   BW.04.8   
  ∟    ◆   BW.04.9   
  ▶     ■    05   
  ∟    ◆   BW.05.1   
  ∟    ◆   BW.05.10   
  ∟    ◆   BW.05.2   
  ∟    ◆   BW.05.3   
  ∟    ◆   BW.05.4   
  ∟    ◆   BW.05.5   
  ∟    ◆   BW.05.6   
  ∟    ◆   BW.05.7   
  ∟    ◆   BW.05.8   
  ∟    ◆   BW.05.9   
  ▶     ■    06   
  ∟    ◆   BW.06.1   
  ∟    ◆   BW.06.10   
  ∟    ◆   BW.06.2   
  ∟    ◆   BW.06.4   
  ∟    ◆   BW.06.5   
  ∟    ◆   BW.06.6   
  ∟    ◆   BW.06.7   
  ∟    ◆   BW.06.8   
  ∟    ◆   BW.06.9   
  ▶     ■    07   
  ∟    ◆   BW.07.1   
  ∟    ◆   BW.07.10   
  ∟    ◆   BW.07.2   
  ∟    ◆   BW.07.3   
  ∟    ◆   BW.07.4   
  ∟    ◆   BW.07.5   
  ∟    ◆   BW.07.6   
  ∟    ◆   BW.07.7   
  ∟    ◆   BW.07.8   
  ∟    ◆   BW.07.9   
  ▶     ■    08   
  ∟    ◆   BW.08.1   
  ∟    ◆   BW.08.10   
  ∟    ◆   BW.08.2   
  ∟    ◆   BW.08.3   
  ∟    ◆   BW.08.4   
  ∟    ◆   BW.08.5   
  ∟    ◆   BW.08.6   
  ∟    ◆   BW.08.7   
  ∟    ◆   BW.08.8   
  ∟    ◆   BW.08.9   
  ▶     ■    09   
  ∟    ◆   BW.09.1   
  ∟    ◆   BW.09.10   
  ∟    ◆   BW.09.2   
  ∟    ◆   BW.09.3   
  ∟    ◆   BW.09.4   
  ∟    ◆   BW.09.5   
  ∟    ◆   BW.09.6   
  ∟    ◆   BW.09.7   
  ∟    ◆   BW.09.8   
  ∟    ◆   BW.09.9   
  ▶     ■    10   
  ∟    ◆   BW.10.1   
  ∟    ◆   BW.10.10   
  ∟    ◆   BW.10.2   
  ∟    ◆   BW.10.4   
  ∟    ◆   BW.10.5   
  ∟    ◆   BW.10.6   
  ∟    ◆   BW.10.8   
  ∟    ◆   BW.10.9   
  ▶     ■    1   
  ∟    ◆   BW.01.1   
  ∟    ◆   BW.02.1   
  ∟    ◆   BW.03.1   
  ∟    ◆   BW.04.1   
  ∟    ◆   BW.05.1   
  ∟    ◆   BW.06.1   
  ∟    ◆   BW.07.1   
  ∟    ◆   BW.08.1   
  ∟    ◆   BW.09.1   
  ∟    ◆   BW.10.1   
  ▶     ■    2   
  ∟    ◆   BW.01.2   
  ∟    ◆   BW.02.2   
  ∟    ◆   BW.04.2   
  ∟    ◆   BW.05.2   
  ∟    ◆   BW.06.2   
  ∟    ◆   BW.07.2   
  ∟    ◆   BW.08.2   
  ∟    ◆   BW.09.2   
  ∟    ◆   BW.10.2   
  ▶     ■    3   
  ∟    ◆   BW.01.3   
  ∟    ◆   BW.02.3   
  ∟    ◆   BW.03.3   
  ∟    ◆   BW.04.3   
  ∟    ◆   BW.05.3   
  ∟    ◆   BW.07.3   
  ∟    ◆   BW.08.3   
  ∟    ◆   BW.09.3   
  ▶     ■    4   
  ∟    ◆   BW.01.4   
  ∟    ◆   BW.02.4   
  ∟    ◆   BW.03.4   
  ∟    ◆   BW.04.4   
  ∟    ◆   BW.05.4   
  ∟    ◆   BW.06.4   
  ∟    ◆   BW.07.4   
  ∟    ◆   BW.08.4   
  ∟    ◆   BW.09.4   
  ∟    ◆   BW.10.4   
  ▶     ■    5   
  ∟    ◆   BW.01.5   
  ∟    ◆   BW.02.5   
  ∟    ◆   BW.03.5   
  ∟    ◆   BW.04.5   
  ∟    ◆   BW.05.5   
  ∟    ◆   BW.06.5   
  ∟    ◆   BW.07.5   
  ∟    ◆   BW.08.5   
  ∟    ◆   BW.09.5   
  ∟    ◆   BW.10.5   
  ▶     ■    6   
  ∟    ◆   BW.01.6   
  ∟    ◆   BW.02.6   
  ∟    ◆   BW.03.6   
  ∟    ◆   BW.04.6   
  ∟    ◆   BW.05.6   
  ∟    ◆   BW.06.6   
  ∟    ◆   BW.07.6   
  ∟    ◆   BW.08.6   
  ∟    ◆   BW.09.6   
  ∟    ◆   BW.10.6   
  ▶     ■    7   
  ∟    ◆   BW.01.7   
  ∟    ◆   BW.02.7   
  ∟    ◆   BW.03.7   
  ∟    ◆   BW.04.7   
  ∟    ◆   BW.05.7   
  ∟    ◆   BW.06.7   
  ∟    ◆   BW.07.7   
  ∟    ◆   BW.08.7   
  ∟    ◆   BW.09.7   
  ▶     ■    8   
  ∟    ◆   BW.01.8   
  ∟    ◆   BW.02.8   
  ∟    ◆   BW.03.8   
  ∟    ◆   BW.04.8   
  ∟    ◆   BW.05.8   
  ∟    ◆   BW.06.8   
  ∟    ◆   BW.07.8   
  ∟    ◆   BW.08.8   
  ∟    ◆   BW.09.8   
  ∟    ◆   BW.10.8   
  ▶     ■    9   
  ∟    ◆   BW.01.9   
  ∟    ◆   BW.02.9   
  ∟    ◆   BW.03.9   
  ∟    ◆   BW.04.9   
  ∟    ◆   BW.05.9   
  ∟    ◆   BW.06.9   
  ∟    ◆   BW.07.9   
  ∟    ◆   BW.08.9   
  ∟    ◆   BW.09.9   
  ∟    ◆   BW.10.9   
  ▶     ■    10   
  ∟    ◆   BW.01.10   
  ∟    ◆   BW.02.10   
  ∟    ◆   BW.03.10   
  ∟    ◆   BW.04.10   
  ∟    ◆   BW.05.10   
  ∟    ◆   BW.06.10   
  ∟    ◆   BW.07.10   
  ∟    ◆   BW.08.10   
  ∟    ◆   BW.09.10   
  ∟    ◆   BW.10.10   
  ▶     ■    ACATACGCGTAG   
  ∟    ◆   BW.03.5   
  ∟    ◆   BW.08.6   
  ▶     ■    ACGACTACAGAG   
  ∟    ◆   BW.02.9   
  ∟    ◆   BW.07.10   
  ▶     ■    ACGAGTGCGTAG   
  ∟    ◆   BW.01.1   
  ∟    ◆   BW.06.1   
  ▶     ■    ACGCGAGTATAG   
  ∟    ◆   BW.03.6   
  ∟    ◆   BW.08.7   
  ▶     ■    ACGCGATCGAAG   
  ∟    ◆   BW.05.7   
  ∟    ◆   BW.10.8   
  ▶     ■    ACGCTCGACAAG   
  ∟    ◆   BW.01.2   
  ∟    ◆   BW.06.2   
  ▶     ■    ACTACTATGTAG   
  ∟    ◆   BW.03.7   
  ∟    ◆   BW.08.8   
  ▶     ■    ACTAGCAGTAAG   
  ∟    ◆   BW.05.8   
  ∟    ◆   BW.10.9   
  ▶     ■    ACTGTACAGTAG   
  ∟    ◆   BW.03.8   
  ∟    ◆   BW.08.9   
  ▶     ■    AGACGCACTCAG   
  ∟    ◆   BW.01.3   
  ∟    ◆   BW.06.4   
  ▶     ■    AGACTATACTAG   
  ∟    ◆   BW.03.9   
  ∟    ◆   BW.08.10   
  ▶     ■    AGCACTGTAGAG   
  ∟    ◆   BW.01.4   
  ∟    ◆   BW.06.5   
  ▶     ■    AGCGTCGTCTAG   
  ∟    ◆   BW.03.10   
  ∟    ◆   BW.09.1   
  ▶     ■    AGCTCACGTAAG   
  ∟    ◆   BW.05.9   
  ∟    ◆   BW.10.10   
  ▶     ■    AGTACGCTATAG   
  ∟    ◆   BW.04.1   
  ∟    ◆   BW.09.2   
  ▶     ■    AGTATACATAAG   
  ∟    ◆   BW.05.10   
  ▶     ■    ATACGACGTAAG   
  ∟    ◆   BW.02.4   
  ∟    ◆   BW.07.5   
  ▶     ■    ATAGAGTACTAG   
  ∟    ◆   BW.04.2   
  ∟    ◆   BW.09.3   
  ▶     ■    ATATCGCGAGAG   
  ∟    ◆   BW.01.6   
  ∟    ◆   BW.06.7   
  ▶     ■    ATCAGACACGAG   
  ∟    ◆   BW.01.5   
  ∟    ◆   BW.06.6   
  ▶     ■    CACGCTACGTAG   
  ∟    ◆   BW.04.3   
  ∟    ◆   BW.09.4   
  ▶     ■    CAGTAGACGTAG   
  ∟    ◆   BW.04.4   
  ∟    ◆   BW.09.5   
  ▶     ■    CATAGTAGTGAG   
  ∟    ◆   BW.02.2   
  ∟    ◆   BW.07.3   
  ▶     ■    CGACGTGACTAG   
  ∟    ◆   BW.04.5   
  ∟    ◆   BW.09.6   
  ▶     ■    CGAGAGATACAG   
  ∟    ◆   BW.02.3   
  ∟    ◆   BW.07.4   
  ▶     ■    CGTAGACTAGAG   
  ∟    ◆   BW.02.10   
  ∟    ◆   BW.08.1   
  ▶     ■    CGTCTAGTACAG   
  ∟    ◆   BW.02.6   
  ∟    ◆   BW.07.7   
  ▶     ■    CGTGTCTCTAAG   
  ∟    ◆   BW.01.7   
  ∟    ◆   BW.06.8   
  ▶     ■    CTCGCGTGTCAG   
  ∟    ◆   BW.01.8   
  ∟    ◆   BW.06.9   
  ▶     ■    TACACACACTAG   
  ∟    ◆   BW.04.6   
  ∟    ◆   BW.09.7   
  ▶     ■    TACACGTGATAG   
  ∟    ◆   BW.04.7   
  ∟    ◆   BW.09.8   
  ▶     ■    TACAGATCGTAG   
  ∟    ◆   BW.04.8   
  ∟    ◆   BW.09.9   
  ▶     ■    TACGAGTATGAG   
  ∟    ◆   BW.03.1   
  ∟    ◆   BW.08.2   
  ▶     ■    TACGCTGTCTAG   
  ∟    ◆   BW.04.9   
  ∟    ◆   BW.09.10   
  ▶     ■    TACTCTCGTGAG   
  ∟    ◆   BW.08.3   
  ▶     ■    TAGAGACGAGAG   
  ∟    ◆   BW.03.3   
  ∟    ◆   BW.08.4   
  ▶     ■    TAGTATCAGCAG   
  ∟    ◆   BW.01.9   
  ∟    ◆   BW.06.10   
  ▶     ■    TAGTGTAGATAG   
  ∟    ◆   BW.04.10   
  ∟    ◆   BW.10.1   
  ▶     ■    TCACGTACTAAG   
  ∟    ◆   BW.02.5   
  ∟    ◆   BW.07.6   
  ▶     ■    TCGATCACGTAG   
  ∟    ◆   BW.05.1   
  ∟    ◆   BW.10.2   
  ▶     ■    TCGCACTAGTAG   
  ∟    ◆   BW.05.2   
  ▶     ■    TCGTCGCTCGAG   
  ∟    ◆   BW.03.4   
  ∟    ◆   BW.08.5   
  ▶     ■    TCTACGTAGCAG   
  ∟    ◆   BW.02.7   
  ∟    ◆   BW.07.8   
  ▶     ■    TCTAGCGACTAG   
  ∟    ◆   BW.05.3   
  ∟    ◆   BW.10.4   
  ▶     ■    TCTATACTATAG   
  ∟    ◆   BW.05.4   
  ∟    ◆   BW.10.5   
  ▶     ■    TCTCTATGCGAG   
  ∟    ◆   BW.01.10   
  ∟    ◆   BW.07.1   
  ▶     ■    TGACGTATGTAG   
  ∟    ◆   BW.05.5   
  ∟    ◆   BW.10.6   
  ▶     ■    TGATACGTCTAG   
  ∟    ◆   BW.02.1   
  ∟    ◆   BW.07.2   
  ▶     ■    TGTACTACTCAG   
  ∟    ◆   BW.02.8   
  ∟    ◆   BW.07.9   
  ▶     ■    TGTGAGTAGTAG   
  ∟    ◆   BW.05.6   
  ▶     ■    BW-01.1.Lib_L_27F-001   
  ∟    ◆   BW.01.1   
  ▶     ■    BW-01.10.Lib_L_27F-010   
  ∟    ◆   BW.01.10   
  ▶     ■    BW-01.2.Lib_L_27F-002   
  ∟    ◆   BW.01.2   
  ▶     ■    BW-01.3.Lib_L_27F-003   
  ∟    ◆   BW.01.3   
  ▶     ■    BW-01.4.Lib_L_27F-004   
  ∟    ◆   BW.01.4   
  ▶     ■    BW-01.5.Lib_L_27F-005   
  ∟    ◆   BW.01.5   
  ▶     ■    BW-01.6.Lib_L_27F-006   
  ∟    ◆   BW.01.6   
  ▶     ■    BW-01.7.Lib_L_27F-007   
  ∟    ◆   BW.01.7   
  ▶     ■    BW-01.8.Lib_L_27F-008   
  ∟    ◆   BW.01.8   
  ▶     ■    BW-01.9.Lib_L_27F-009   
  ∟    ◆   BW.01.9   
  ▶     ■    BW-02.1.Lib_L_27F-011   
  ∟    ◆   BW.02.1   
  ▶     ■    BW-02.10.Lib_L_27F-021   
  ∟    ◆   BW.02.10   
  ▶     ■    BW-02.2.Lib_L_27F-013   
  ∟    ◆   BW.02.2   
  ▶     ■    BW-02.3.Lib_L_27F-014   
  ∟    ◆   BW.02.3   
  ▶     ■    BW-02.4.Lib_L_27F-015   
  ∟    ◆   BW.02.4   
  ▶     ■    BW-02.5.Lib_L_27F-016   
  ∟    ◆   BW.02.5   
  ▶     ■    BW-02.6.Lib_L_27F-017   
  ∟    ◆   BW.02.6   
  ▶     ■    BW-02.7.Lib_L_27F-018   
  ∟    ◆   BW.02.7   
  ▶     ■    BW-02.8.Lib_L_27F-019   
  ∟    ◆   BW.02.8   
  ▶     ■    BW-02.9.Lib_L_27F-020   
  ∟    ◆   BW.02.9   
  ▶     ■    BW-03.1.Lib_L_27F-022   
  ∟    ◆   BW.03.1   
  ▶     ■    BW-03.10.Lib_L_27F-031   
  ∟    ◆   BW.03.10   
  ▶     ■    BW-03.3.Lib_L_27F-024   
  ∟    ◆   BW.03.3   
  ▶     ■    BW-03.4.Lib_L_27F-025   
  ∟    ◆   BW.03.4   
  ▶     ■    BW-03.5.Lib_L_27F-026   
  ∟    ◆   BW.03.5   
  ▶     ■    BW-03.6.Lib_L_27F-027   
  ∟    ◆   BW.03.6   
  ▶     ■    BW-03.7.Lib_L_27F-028   
  ∟    ◆   BW.03.7   
  ▶     ■    BW-03.8.Lib_L_27F-029   
  ∟    ◆   BW.03.8   
  ▶     ■    BW-03.9.Lib_L_27F-030   
  ∟    ◆   BW.03.9   
  ▶     ■    BW-04.1.Lib_L_27F-032   
  ∟    ◆   BW.04.1   
  ▶     ■    BW-04.10.Lib_L_27F-041   
  ∟    ◆   BW.04.10   
  ▶     ■    BW-04.2.Lib_L_27F-033   
  ∟    ◆   BW.04.2   
  ▶     ■    BW-04.3.Lib_L_27F-034   
  ∟    ◆   BW.04.3   
  ▶     ■    BW-04.4.Lib_L_27F-035   
  ∟    ◆   BW.04.4   
  ▶     ■    BW-04.5.Lib_L_27F-036   
  ∟    ◆   BW.04.5   
  ▶     ■    BW-04.6.Lib_L_27F-037   
  ∟    ◆   BW.04.6   
  ▶     ■    BW-04.7.Lib_L_27F-038   
  ∟    ◆   BW.04.7   
  ▶     ■    BW-04.8.Lib_L_27F-039   
  ∟    ◆   BW.04.8   
  ▶     ■    BW-04.9.Lib_L_27F-040   
  ∟    ◆   BW.04.9   
  ▶     ■    BW-05.1.Lib_L_27F-042   
  ∟    ◆   BW.05.1   
  ▶     ■    BW-05.10.Lib_L_27F-052   
  ∟    ◆   BW.05.10   
  ▶     ■    BW-05.2.Lib_L_27F-043   
  ∟    ◆   BW.05.2   
  ▶     ■    BW-05.3.Lib_L_27F-044   
  ∟    ◆   BW.05.3   
  ▶     ■    BW-05.4.Lib_L_27F-045   
  ∟    ◆   BW.05.4   
  ▶     ■    BW-05.5.Lib_L_27F-046   
  ∟    ◆   BW.05.5   
  ▶     ■    BW-05.6.Lib_L_27F-047   
  ∟    ◆   BW.05.6   
  ▶     ■    BW-05.7.Lib_L_27F-049   
  ∟    ◆   BW.05.7   
  ▶     ■    BW-05.8.Lib_L_27F-050   
  ∟    ◆   BW.05.8   
  ▶     ■    BW-05.9.Lib_L_27F-051   
  ∟    ◆   BW.05.9   
  ▶     ■    BW-06.1.Lib_L_27F-001   
  ∟    ◆   BW.06.1   
  ▶     ■    BW-06.10.Lib_L_27F-009   
  ∟    ◆   BW.06.10   
  ▶     ■    BW-06.2.Lib_L_27F-002   
  ∟    ◆   BW.06.2   
  ▶     ■    BW-06.4.Lib_L_27F-003   
  ∟    ◆   BW.06.4   
  ▶     ■    BW-06.5.Lib_L_27F-004   
  ∟    ◆   BW.06.5   
  ▶     ■    BW-06.6.Lib_L_27F-005   
  ∟    ◆   BW.06.6   
  ▶     ■    BW-06.7.Lib_L_27F-006   
  ∟    ◆   BW.06.7   
  ▶     ■    BW-06.8.Lib_L_27F-007   
  ∟    ◆   BW.06.8   
  ▶     ■    BW-06.9.Lib_L_27F-008   
  ∟    ◆   BW.06.9   
  ▶     ■    BW-07.1.Lib_L_27F-010   
  ∟    ◆   BW.07.1   
  ▶     ■    BW-07.10.Lib_L_27F-020   
  ∟    ◆   BW.07.10   
  ▶     ■    BW-07.2.Lib_L_27F-011   
  ∟    ◆   BW.07.2   
  ▶     ■    BW-07.3.Lib_L_27F-013   
  ∟    ◆   BW.07.3   
  ▶     ■    BW-07.4.Lib_L_27F-014   
  ∟    ◆   BW.07.4   
  ▶     ■    BW-07.5.Lib_L_27F-015   
  ∟    ◆   BW.07.5   
  ▶     ■    BW-07.6.Lib_L_27F-016   
  ∟    ◆   BW.07.6   
  ▶     ■    BW-07.7.Lib_L_27F-017   
  ∟    ◆   BW.07.7   
  ▶     ■    BW-07.8.Lib_L_27F-018   
  ∟    ◆   BW.07.8   
  ▶     ■    BW-07.9.Lib_L_27F-019   
  ∟    ◆   BW.07.9   
  ▶     ■    BW-08.1.Lib_L_27F-021   
  ∟    ◆   BW.08.1   
  ▶     ■    BW-08.10.Lib_L_27F-030   
  ∟    ◆   BW.08.10   
  ▶     ■    BW-08.2.Lib_L_27F-022   
  ∟    ◆   BW.08.2   
  ▶     ■    BW-08.3.Lib_L_27F-023   
  ∟    ◆   BW.08.3   
  ▶     ■    BW-08.4.Lib_L_27F-024   
  ∟    ◆   BW.08.4   
  ▶     ■    BW-08.5.Lib_L_27F-025   
  ∟    ◆   BW.08.5   
  ▶     ■    BW-08.6.Lib_L_27F-026   
  ∟    ◆   BW.08.6   
  ▶     ■    BW-08.7.Lib_L_27F-027   
  ∟    ◆   BW.08.7   
  ▶     ■    BW-08.8.Lib_L_27F-028   
  ∟    ◆   BW.08.8   
  ▶     ■    BW-08.9.Lib_L_27F-029   
  ∟    ◆   BW.08.9   
  ▶     ■    BW-09.1.Lib_L_27F-031   
  ∟    ◆   BW.09.1   
  ▶     ■    BW-09.10.Lib_L_27F-040   
  ∟    ◆   BW.09.10   
  ▶     ■    BW-09.2.Lib_L_27F-032   
  ∟    ◆   BW.09.2   
  ▶     ■    BW-09.3.Lib_L_27F-033   
  ∟    ◆   BW.09.3   
  ▶     ■    BW-09.4.Lib_L_27F-034   
  ∟    ◆   BW.09.4   
  ▶     ■    BW-09.5.Lib_L_27F-035   
  ∟    ◆   BW.09.5   
  ▶     ■    BW-09.6.Lib_L_27F-036   
  ∟    ◆   BW.09.6   
  ▶     ■    BW-09.7.Lib_L_27F-037   
  ∟    ◆   BW.09.7   
  ▶     ■    BW-09.8.Lib_L_27F-038   
  ∟    ◆   BW.09.8   
  ▶     ■    BW-09.9.Lib_L_27F-039   
  ∟    ◆   BW.09.9   
  ▶     ■    BW-10.1.Lib_L_27F-041   
  ∟    ◆   BW.10.1   
  ▶     ■    BW-10.10.Lib_L_27F-051   
  ∟    ◆   BW.10.10   
  ▶     ■    BW-10.2.Lib_L_27F-042   
  ∟    ◆   BW.10.2   
  ▶     ■    BW-10.4.Lib_L_27F-044   
  ∟    ◆   BW.10.4   
  ▶     ■    BW-10.5.Lib_L_27F-045   
  ∟    ◆   BW.10.5   
  ▶     ■    BW-10.6.Lib_L_27F-046   
  ∟    ◆   BW.10.6   
  ▶     ■    BW-10.8.Lib_L_27F-049   
  ∟    ◆   BW.10.8   
  ▶     ■    BW-10.9.Lib_L_27F-050   
  ∟    ◆   BW.10.9   
  ▶     ■    After   
  ∟    ◆   BW.01.10   
  ∟    ◆   BW.01.9   
  ∟    ◆   BW.02.10   
  ∟    ◆   BW.02.9   
  ∟    ◆   BW.03.10   
  ∟    ◆   BW.03.9   
  ∟    ◆   BW.04.10   
  ∟    ◆   BW.04.9   
  ∟    ◆   BW.05.10   
  ∟    ◆   BW.05.9   
  ∟    ◆   BW.06.10   
  ∟    ◆   BW.06.9   
  ∟    ◆   BW.07.10   
  ∟    ◆   BW.07.9   
  ∟    ◆   BW.08.10   
  ∟    ◆   BW.08.9   
  ∟    ◆   BW.09.10   
  ∟    ◆   BW.09.9   
  ∟    ◆   BW.10.10   
  ∟    ◆   BW.10.9   
  ▶     ■    Before   
  ∟    ◆   BW.01.1   
  ∟    ◆   BW.01.2   
  ∟    ◆   BW.01.3   
  ∟    ◆   BW.02.1   
  ∟    ◆   BW.02.2   
  ∟    ◆   BW.02.3   
  ∟    ◆   BW.03.1   
  ∟    ◆   BW.03.3   
  ∟    ◆   BW.04.1   
  ∟    ◆   BW.04.2   
  ∟    ◆   BW.04.3   
  ∟    ◆   BW.05.1   
  ∟    ◆   BW.05.2   
  ∟    ◆   BW.05.3   
  ∟    ◆   BW.06.1   
  ∟    ◆   BW.06.2   
  ∟    ◆   BW.07.1   
  ∟    ◆   BW.07.2   
  ∟    ◆   BW.07.3   
  ∟    ◆   BW.08.1   
  ∟    ◆   BW.08.2   
  ∟    ◆   BW.08.3   
  ∟    ◆   BW.09.1   
  ∟    ◆   BW.09.2   
  ∟    ◆   BW.09.3   
  ∟    ◆   BW.10.1   
  ∟    ◆   BW.10.2   
  ▶     ■    During   
  ∟    ◆   BW.01.4   
  ∟    ◆   BW.01.5   
  ∟    ◆   BW.01.6   
  ∟    ◆   BW.01.7   
  ∟    ◆   BW.01.8   
  ∟    ◆   BW.02.4   
  ∟    ◆   BW.02.5   
  ∟    ◆   BW.02.6   
  ∟    ◆   BW.02.7   
  ∟    ◆   BW.02.8   
  ∟    ◆   BW.03.4   
  ∟    ◆   BW.03.5   
  ∟    ◆   BW.03.6   
  ∟    ◆   BW.03.7   
  ∟    ◆   BW.03.8   
  ∟    ◆   BW.04.4   
  ∟    ◆   BW.04.5   
  ∟    ◆   BW.04.6   
  ∟    ◆   BW.04.7   
  ∟    ◆   BW.04.8   
  ∟    ◆   BW.05.4   
  ∟    ◆   BW.05.5   
  ∟    ◆   BW.05.6   
  ∟    ◆   BW.05.7   
  ∟    ◆   BW.05.8   
  ∟    ◆   BW.06.4   
  ∟    ◆   BW.06.5   
  ∟    ◆   BW.06.6   
  ∟    ◆   BW.06.7   
  ∟    ◆   BW.06.8   
  ∟    ◆   BW.07.4   
  ∟    ◆   BW.07.5   
  ∟    ◆   BW.07.6   
  ∟    ◆   BW.07.7   
  ∟    ◆   BW.07.8   
  ∟    ◆   BW.08.4   
  ∟    ◆   BW.08.5   
  ∟    ◆   BW.08.6   
  ∟    ◆   BW.08.7   
  ∟    ◆   BW.08.8   
  ∟    ◆   BW.09.4   
  ∟    ◆   BW.09.5   
  ∟    ◆   BW.09.6   
  ∟    ◆   BW.09.7   
  ∟    ◆   BW.09.8   
  ∟    ◆   BW.10.4   
  ∟    ◆   BW.10.5   
  ∟    ◆   BW.10.6   
  ∟    ◆   BW.10.8   
  ▶     ■    Lib_L_27F-001   
  ∟    ◆   BW.01.1   
  ∟    ◆   BW.06.1   
  ▶     ■    Lib_L_27F-002   
  ∟    ◆   BW.01.2   
  ∟    ◆   BW.06.2   
  ▶     ■    Lib_L_27F-003   
  ∟    ◆   BW.01.3   
  ∟    ◆   BW.06.4   
  ▶     ■    Lib_L_27F-004   
  ∟    ◆   BW.01.4   
  ∟    ◆   BW.06.5   
  ▶     ■    Lib_L_27F-005   
  ∟    ◆   BW.01.5   
  ∟    ◆   BW.06.6   
  ▶     ■    Lib_L_27F-006   
  ∟    ◆   BW.01.6   
  ∟    ◆   BW.06.7   
  ▶     ■    Lib_L_27F-007   
  ∟    ◆   BW.01.7   
  ∟    ◆   BW.06.8   
  ▶     ■    Lib_L_27F-008   
  ∟    ◆   BW.01.8   
  ∟    ◆   BW.06.9   
  ▶     ■    Lib_L_27F-009   
  ∟    ◆   BW.01.9   
  ∟    ◆   BW.06.10   
  ▶     ■    Lib_L_27F-010   
  ∟    ◆   BW.01.10   
  ∟    ◆   BW.07.1   
  ▶     ■    Lib_L_27F-011   
  ∟    ◆   BW.02.1   
  ∟    ◆   BW.07.2   
  ▶     ■    Lib_L_27F-013   
  ∟    ◆   BW.02.2   
  ∟    ◆   BW.07.3   
  ▶     ■    Lib_L_27F-014   
  ∟    ◆   BW.02.3   
  ∟    ◆   BW.07.4   
  ▶     ■    Lib_L_27F-015   
  ∟    ◆   BW.02.4   
  ∟    ◆   BW.07.5   
  ▶     ■    Lib_L_27F-016   
  ∟    ◆   BW.02.5   
  ∟    ◆   BW.07.6   
  ▶     ■    Lib_L_27F-017   
  ∟    ◆   BW.02.6   
  ∟    ◆   BW.07.7   
  ▶     ■    Lib_L_27F-018   
  ∟    ◆   BW.02.7   
  ∟    ◆   BW.07.8   
  ▶     ■    Lib_L_27F-019   
  ∟    ◆   BW.02.8   
  ∟    ◆   BW.07.9   
  ▶     ■    Lib_L_27F-020   
  ∟    ◆   BW.02.9   
  ∟    ◆   BW.07.10   
  ▶     ■    Lib_L_27F-021   
  ∟    ◆   BW.02.10   
  ∟    ◆   BW.08.1   
  ▶     ■    Lib_L_27F-022   
  ∟    ◆   BW.03.1   
  ∟    ◆   BW.08.2   
  ▶     ■    Lib_L_27F-023   
  ∟    ◆   BW.08.3   
  ▶     ■    Lib_L_27F-024   
  ∟    ◆   BW.03.3   
  ∟    ◆   BW.08.4   
  ▶     ■    Lib_L_27F-025   
  ∟    ◆   BW.03.4   
  ∟    ◆   BW.08.5   
  ▶     ■    Lib_L_27F-026   
  ∟    ◆   BW.03.5   
  ∟    ◆   BW.08.6   
  ▶     ■    Lib_L_27F-027   
  ∟    ◆   BW.03.6   
  ∟    ◆   BW.08.7   
  ▶     ■    Lib_L_27F-028   
  ∟    ◆   BW.03.7   
  ∟    ◆   BW.08.8   
  ▶     ■    Lib_L_27F-029   
  ∟    ◆   BW.03.8   
  ∟    ◆   BW.08.9   
  ▶     ■    Lib_L_27F-030   
  ∟    ◆   BW.03.9   
  ∟    ◆   BW.08.10   
  ▶     ■    Lib_L_27F-031   
  ∟    ◆   BW.03.10   
  ∟    ◆   BW.09.1   
  ▶     ■    Lib_L_27F-032   
  ∟    ◆   BW.04.1   
  ∟    ◆   BW.09.2   
  ▶     ■    Lib_L_27F-033   
  ∟    ◆   BW.04.2   
  ∟    ◆   BW.09.3   
  ▶     ■    Lib_L_27F-034   
  ∟    ◆   BW.04.3   
  ∟    ◆   BW.09.4   
  ▶     ■    Lib_L_27F-035   
  ∟    ◆   BW.04.4   
  ∟    ◆   BW.09.5   
  ▶     ■    Lib_L_27F-036   
  ∟    ◆   BW.04.5   
  ∟    ◆   BW.09.6   
  ▶     ■    Lib_L_27F-037   
  ∟    ◆   BW.04.6   
  ∟    ◆   BW.09.7   
  ▶     ■    Lib_L_27F-038   
  ∟    ◆   BW.04.7   
  ∟    ◆   BW.09.8   
  ▶     ■    Lib_L_27F-039   
  ∟    ◆   BW.04.8   
  ∟    ◆   BW.09.9   
  ▶     ■    Lib_L_27F-040   
  ∟    ◆   BW.04.9   
  ∟    ◆   BW.09.10   
  ▶     ■    Lib_L_27F-041   
  ∟    ◆   BW.04.10   
  ∟    ◆   BW.10.1   
  ▶     ■    Lib_L_27F-042   
  ∟    ◆   BW.05.1   
  ∟    ◆   BW.10.2   
  ▶     ■    Lib_L_27F-043   
  ∟    ◆   BW.05.2   
  ▶     ■    Lib_L_27F-044   
  ∟    ◆   BW.05.3   
  ∟    ◆   BW.10.4   
  ▶     ■    Lib_L_27F-045   
  ∟    ◆   BW.05.4   
  ∟    ◆   BW.10.5   
  ▶     ■    Lib_L_27F-046   
  ∟    ◆   BW.05.5   
  ∟    ◆   BW.10.6   
  ▶     ■    Lib_L_27F-047   
  ∟    ◆   BW.05.6   
  ▶     ■    Lib_L_27F-049   
  ∟    ◆   BW.05.7   
  ∟    ◆   BW.10.8   
  ▶     ■    Lib_L_27F-050   
  ∟    ◆   BW.05.8   
  ∟    ◆   BW.10.9   
  ▶     ■    Lib_L_27F-051   
  ∟    ◆   BW.05.9   
  ∟    ◆   BW.10.10   
  ▶     ■    Lib_L_27F-052   
  ∟    ◆   BW.05.10   
  ▶     ■    BW.01.1   
  ∟    ◆   BW.01.1   
  ▶     ■    BW.01.10   
  ∟    ◆   BW.01.10   
  ▶     ■    BW.01.2   
  ∟    ◆   BW.01.2   
  ▶     ■    BW.01.3   
  ∟    ◆   BW.01.3   
  ▶     ■    BW.01.4   
  ∟    ◆   BW.01.4   
  ▶     ■    BW.01.5   
  ∟    ◆   BW.01.5   
  ▶     ■    BW.01.6   
  ∟    ◆   BW.01.6   
  ▶     ■    BW.01.7   
  ∟    ◆   BW.01.7   
  ▶     ■    BW.01.8   
  ∟    ◆   BW.01.8   
  ▶     ■    BW.01.9   
  ∟    ◆   BW.01.9   
  ▶     ■    BW.02.1   
  ∟    ◆   BW.02.1   
  ▶     ■    BW.02.10   
  ∟    ◆   BW.02.10   
  ▶     ■    BW.02.2   
  ∟    ◆   BW.02.2   
  ▶     ■    BW.02.3   
  ∟    ◆   BW.02.3   
  ▶     ■    BW.02.4   
  ∟    ◆   BW.02.4   
  ▶     ■    BW.02.5   
  ∟    ◆   BW.02.5   
  ▶     ■    BW.02.6   
  ∟    ◆   BW.02.6   
  ▶     ■    BW.02.7   
  ∟    ◆   BW.02.7   
  ▶     ■    BW.02.8   
  ∟    ◆   BW.02.8   
  ▶     ■    BW.02.9   
  ∟    ◆   BW.02.9   
  ▶     ■    BW.03.1   
  ∟    ◆   BW.03.1   
  ▶     ■    BW.03.10   
  ∟    ◆   BW.03.10   
  ▶     ■    BW.03.3   
  ∟    ◆   BW.03.3   
  ▶     ■    BW.03.4   
  ∟    ◆   BW.03.4   
  ▶     ■    BW.03.5   
  ∟    ◆   BW.03.5   
  ▶     ■    BW.03.6   
  ∟    ◆   BW.03.6   
  ▶     ■    BW.03.7   
  ∟    ◆   BW.03.7   
  ▶     ■    BW.03.8   
  ∟    ◆   BW.03.8   
  ▶     ■    BW.03.9   
  ∟    ◆   BW.03.9   
  ▶     ■    BW.04.1   
  ∟    ◆   BW.04.1   
  ▶     ■    BW.04.10   
  ∟    ◆   BW.04.10   
  ▶     ■    BW.04.2   
  ∟    ◆   BW.04.2   
  ▶     ■    BW.04.3   
  ∟    ◆   BW.04.3   
  ▶     ■    BW.04.4   
  ∟    ◆   BW.04.4   
  ▶     ■    BW.04.5   
  ∟    ◆   BW.04.5   
  ▶     ■    BW.04.6   
  ∟    ◆   BW.04.6   
  ▶     ■    BW.04.7   
  ∟    ◆   BW.04.7   
  ▶     ■    BW.04.8   
  ∟    ◆   BW.04.8   
  ▶     ■    BW.04.9   
  ∟    ◆   BW.04.9   
  ▶     ■    BW.05.1   
  ∟    ◆   BW.05.1   
  ▶     ■    BW.05.10   
  ∟    ◆   BW.05.10   
  ▶     ■    BW.05.2   
  ∟    ◆   BW.05.2   
  ▶     ■    BW.05.3   
  ∟    ◆   BW.05.3   
  ▶     ■    BW.05.4   
  ∟    ◆   BW.05.4   
  ▶     ■    BW.05.5   
  ∟    ◆   BW.05.5   
  ▶     ■    BW.05.6   
  ∟    ◆   BW.05.6   
  ▶     ■    BW.05.7   
  ∟    ◆   BW.05.7   
  ▶     ■    BW.05.8   
  ∟    ◆   BW.05.8   
  ▶     ■    BW.05.9   
  ∟    ◆   BW.05.9   
  ▶     ■    BW.06.1   
  ∟    ◆   BW.06.1   
  ▶     ■    BW.06.10   
  ∟    ◆   BW.06.10   
  ▶     ■    BW.06.2   
  ∟    ◆   BW.06.2   
  ▶     ■    BW.06.4   
  ∟    ◆   BW.06.4   
  ▶     ■    BW.06.5   
  ∟    ◆   BW.06.5   
  ▶     ■    BW.06.6   
  ∟    ◆   BW.06.6   
  ▶     ■    BW.06.7   
  ∟    ◆   BW.06.7   
  ▶     ■    BW.06.8   
  ∟    ◆   BW.06.8   
  ▶     ■    BW.06.9   
  ∟    ◆   BW.06.9   
  ▶     ■    BW.07.1   
  ∟    ◆   BW.07.1   
  ▶     ■    BW.07.10   
  ∟    ◆   BW.07.10   
  ▶     ■    BW.07.2   
  ∟    ◆   BW.07.2   
  ▶     ■    BW.07.3   
  ∟    ◆   BW.07.3   
  ▶     ■    BW.07.4   
  ∟    ◆   BW.07.4   
  ▶     ■    BW.07.5   
  ∟    ◆   BW.07.5   
  ▶     ■    BW.07.6   
  ∟    ◆   BW.07.6   
  ▶     ■    BW.07.7   
  ∟    ◆   BW.07.7   
  ▶     ■    BW.07.8   
  ∟    ◆   BW.07.8   
  ▶     ■    BW.07.9   
  ∟    ◆   BW.07.9   
  ▶     ■    BW.08.1   
  ∟    ◆   BW.08.1   
  ▶     ■    BW.08.10   
  ∟    ◆   BW.08.10   
  ▶     ■    BW.08.2   
  ∟    ◆   BW.08.2   
  ▶     ■    BW.08.3   
  ∟    ◆   BW.08.3   
  ▶     ■    BW.08.4   
  ∟    ◆   BW.08.4   
  ▶     ■    BW.08.5   
  ∟    ◆   BW.08.5   
  ▶     ■    BW.08.6   
  ∟    ◆   BW.08.6   
  ▶     ■    BW.08.7   
  ∟    ◆   BW.08.7   
  ▶     ■    BW.08.8   
  ∟    ◆   BW.08.8   
  ▶     ■    BW.08.9   
  ∟    ◆   BW.08.9   
  ▶     ■    BW.09.1   
  ∟    ◆   BW.09.1   
  ▶     ■    BW.09.10   
  ∟    ◆   BW.09.10   
  ▶     ■    BW.09.2   
  ∟    ◆   BW.09.2   
  ▶     ■    BW.09.3   
  ∟    ◆   BW.09.3   
  ▶     ■    BW.09.4   
  ∟    ◆   BW.09.4   
  ▶     ■    BW.09.5   
  ∟    ◆   BW.09.5   
  ▶     ■    BW.09.6   
  ∟    ◆   BW.09.6   
  ▶     ■    BW.09.7   
  ∟    ◆   BW.09.7   
  ▶     ■    BW.09.8   
  ∟    ◆   BW.09.8   
  ▶     ■    BW.09.9   
  ∟    ◆   BW.09.9   
  ▶     ■    BW.10.1   
  ∟    ◆   BW.10.1   
  ▶     ■    BW.10.10   
  ∟    ◆   BW.10.10   
  ▶     ■    BW.10.2   
  ∟    ◆   BW.10.2   
  ▶     ■    BW.10.4   
  ∟    ◆   BW.10.4   
  ▶     ■    BW.10.5   
  ∟    ◆   BW.10.5   
  ▶     ■    BW.10.6   
  ∟    ◆   BW.10.6   
  ▶     ■    BW.10.8   
  ∟    ◆   BW.10.8   
  ▶     ■    BW.10.9   
  ∟    ◆   BW.10.9   
  ▶     ■    01   
  ∟    ◆   BW.01.1   
  ∟    ◆   BW.01.10   
  ∟    ◆   BW.01.2   
  ∟    ◆   BW.01.3   
  ∟    ◆   BW.01.4   
  ∟    ◆   BW.01.5   
  ∟    ◆   BW.01.6   
  ∟    ◆   BW.01.7   
  ∟    ◆   BW.01.8   
  ∟    ◆   BW.01.9   
  ▶     ■    02   
  ∟    ◆   BW.02.1   
  ∟    ◆   BW.02.10   
  ∟    ◆   BW.02.2   
  ∟    ◆   BW.02.3   
  ∟    ◆   BW.02.4   
  ∟    ◆   BW.02.5   
  ∟    ◆   BW.02.6   
  ∟    ◆   BW.02.7   
  ∟    ◆   BW.02.8   
  ∟    ◆   BW.02.9   
  ▶     ■    03   
  ∟    ◆   BW.03.1   
  ∟    ◆   BW.03.10   
  ∟    ◆   BW.03.3   
  ∟    ◆   BW.03.4   
  ∟    ◆   BW.03.5   
  ∟    ◆   BW.03.6   
  ∟    ◆   BW.03.7   
  ∟    ◆   BW.03.8   
  ∟    ◆   BW.03.9   
  ▶     ■    04   
  ∟    ◆   BW.04.1   
  ∟    ◆   BW.04.10   
  ∟    ◆   BW.04.2   
  ∟    ◆   BW.04.3   
  ∟    ◆   BW.04.4   
  ∟    ◆   BW.04.5   
  ∟    ◆   BW.04.6   
  ∟    ◆   BW.04.7   
  ∟    ◆   BW.04.8   
  ∟    ◆   BW.04.9   
  ▶     ■    05   
  ∟    ◆   BW.05.1   
  ∟    ◆   BW.05.10   
  ∟    ◆   BW.05.2   
  ∟    ◆   BW.05.3   
  ∟    ◆   BW.05.4   
  ∟    ◆   BW.05.5   
  ∟    ◆   BW.05.6   
  ∟    ◆   BW.05.7   
  ∟    ◆   BW.05.8   
  ∟    ◆   BW.05.9   
  ▶     ■    06   
  ∟    ◆   BW.06.1   
  ∟    ◆   BW.06.10   
  ∟    ◆   BW.06.2   
  ∟    ◆   BW.06.4   
  ∟    ◆   BW.06.5   
  ∟    ◆   BW.06.6   
  ∟    ◆   BW.06.7   
  ∟    ◆   BW.06.8   
  ∟    ◆   BW.06.9   
  ▶     ■    07   
  ∟    ◆   BW.07.1   
  ∟    ◆   BW.07.10   
  ∟    ◆   BW.07.2   
  ∟    ◆   BW.07.3   
  ∟    ◆   BW.07.4   
  ∟    ◆   BW.07.5   
  ∟    ◆   BW.07.6   
  ∟    ◆   BW.07.7   
  ∟    ◆   BW.07.8   
  ∟    ◆   BW.07.9   
  ▶     ■    08   
  ∟    ◆   BW.08.1   
  ∟    ◆   BW.08.10   
  ∟    ◆   BW.08.2   
  ∟    ◆   BW.08.3   
  ∟    ◆   BW.08.4   
  ∟    ◆   BW.08.5   
  ∟    ◆   BW.08.6   
  ∟    ◆   BW.08.7   
  ∟    ◆   BW.08.8   
  ∟    ◆   BW.08.9   
  ▶     ■    09   
  ∟    ◆   BW.09.1   
  ∟    ◆   BW.09.10   
  ∟    ◆   BW.09.2   
  ∟    ◆   BW.09.3   
  ∟    ◆   BW.09.4   
  ∟    ◆   BW.09.5   
  ∟    ◆   BW.09.6   
  ∟    ◆   BW.09.7   
  ∟    ◆   BW.09.8   
  ∟    ◆   BW.09.9   
  ▶     ■    10   
  ∟    ◆   BW.10.1   
  ∟    ◆   BW.10.10   
  ∟    ◆   BW.10.2   
  ∟    ◆   BW.10.4   
  ∟    ◆   BW.10.5   
  ∟    ◆   BW.10.6   
  ∟    ◆   BW.10.8   
  ∟    ◆   BW.10.9   
  ▶     ■    1   
  ∟    ◆   BW.01.1   
  ∟    ◆   BW.02.1   
  ∟    ◆   BW.03.1   
  ∟    ◆   BW.04.1   
  ∟    ◆   BW.05.1   
  ∟    ◆   BW.06.1   
  ∟    ◆   BW.07.1   
  ∟    ◆   BW.08.1   
  ∟    ◆   BW.09.1   
  ∟    ◆   BW.10.1   
  ▶     ■    2   
  ∟    ◆   BW.01.2   
  ∟    ◆   BW.02.2   
  ∟    ◆   BW.04.2   
  ∟    ◆   BW.05.2   
  ∟    ◆   BW.06.2   
  ∟    ◆   BW.07.2   
  ∟    ◆   BW.08.2   
  ∟    ◆   BW.09.2   
  ∟    ◆   BW.10.2   
  ▶     ■    3   
  ∟    ◆   BW.01.3   
  ∟    ◆   BW.02.3   
  ∟    ◆   BW.03.3   
  ∟    ◆   BW.04.3   
  ∟    ◆   BW.05.3   
  ∟    ◆   BW.07.3   
  ∟    ◆   BW.08.3   
  ∟    ◆   BW.09.3   
  ▶     ■    4   
  ∟    ◆   BW.01.4   
  ∟    ◆   BW.02.4   
  ∟    ◆   BW.03.4   
  ∟    ◆   BW.04.4   
  ∟    ◆   BW.05.4   
  ∟    ◆   BW.06.4   
  ∟    ◆   BW.07.4   
  ∟    ◆   BW.08.4   
  ∟    ◆   BW.09.4   
  ∟    ◆   BW.10.4   
  ▶     ■    5   
  ∟    ◆   BW.01.5   
  ∟    ◆   BW.02.5   
  ∟    ◆   BW.03.5   
  ∟    ◆   BW.04.5   
  ∟    ◆   BW.05.5   
  ∟    ◆   BW.06.5   
  ∟    ◆   BW.07.5   
  ∟    ◆   BW.08.5   
  ∟    ◆   BW.09.5   
  ∟    ◆   BW.10.5   
  ▶     ■    6   
  ∟    ◆   BW.01.6   
  ∟    ◆   BW.02.6   
  ∟    ◆   BW.03.6   
  ∟    ◆   BW.04.6   
  ∟    ◆   BW.05.6   
  ∟    ◆   BW.06.6   
  ∟    ◆   BW.07.6   
  ∟    ◆   BW.08.6   
  ∟    ◆   BW.09.6   
  ∟    ◆   BW.10.6   
  ▶     ■    7   
  ∟    ◆   BW.01.7   
  ∟    ◆   BW.02.7   
  ∟    ◆   BW.03.7   
  ∟    ◆   BW.04.7   
  ∟    ◆   BW.05.7   
  ∟    ◆   BW.06.7   
  ∟    ◆   BW.07.7   
  ∟    ◆   BW.08.7   
  ∟    ◆   BW.09.7   
  ▶     ■    8   
  ∟    ◆   BW.01.8   
  ∟    ◆   BW.02.8   
  ∟    ◆   BW.03.8   
  ∟    ◆   BW.04.8   
  ∟    ◆   BW.05.8   
  ∟    ◆   BW.06.8   
  ∟    ◆   BW.07.8   
  ∟    ◆   BW.08.8   
  ∟    ◆   BW.09.8   
  ∟    ◆   BW.10.8   
  ▶     ■    9   
  ∟    ◆   BW.01.9   
  ∟    ◆   BW.02.9   
  ∟    ◆   BW.03.9   
  ∟    ◆   BW.04.9   
  ∟    ◆   BW.05.9   
  ∟    ◆   BW.06.9   
  ∟    ◆   BW.07.9   
  ∟    ◆   BW.08.9   
  ∟    ◆   BW.09.9   
  ∟    ◆   BW.10.9   
  ▶     ■    10   
  ∟    ◆   BW.01.10   
  ∟    ◆   BW.02.10   
  ∟    ◆   BW.03.10   
  ∟    ◆   BW.04.10   
  ∟    ◆   BW.05.10   
  ∟    ◆   BW.06.10   
  ∟    ◆   BW.07.10   
  ∟    ◆   BW.08.10   
  ∟    ◆   BW.09.10   
  ∟    ◆   BW.10.10   
   
 
 
 If the lines for some categories do not extend all the way to the right end of the x-axis, that means that at least one of the samples in that category does not have that many samples. 
 
  
 
  LibCodes  Seqs/Sample 
 chao1 Ave.  chao1 Err. 
 observed_species Ave.  observed_species Err. 
 shannon Ave.  shannon Err. 
 
 
 Lib_L_27F-001  10.0 
     12.495       3.037 
      6.355       0.805 
      2.427       0.232 
 
 Lib_L_27F-001  24.0 
     24.280       6.148 
     11.100       1.900 
      2.996       0.331 
 
 Lib_L_27F-001  38.0 
     35.351       9.119 
     14.655       2.635 
      3.202       0.367 
 
 Lib_L_27F-001  52.0 
     44.425       7.777 
     17.960       3.370 
      3.356       0.409 
 
 Lib_L_27F-001  66.0 
     51.272      13.335 
     20.805       4.105 
      3.458       0.426 
 
 Lib_L_27F-001  80.0 
     62.085      22.053 
     22.910       4.590 
      3.493       0.424 
 
 Lib_L_27F-001  94.0 
     68.445      18.128 
     25.520       5.220 
      3.542       0.450 
 
 Lib_L_27F-001  108.0 
     80.245      30.150 
     28.245       5.745 
      3.613       0.447 
 
 Lib_L_27F-001  122.0 
     77.821      31.579 
     30.150       6.220 
      3.638       0.457 
 
 Lib_L_27F-001  136.0 
     86.839      34.136 
     32.575       6.875 
      3.677       0.476 
 
 Lib_L_27F-001  150.0 
     84.300      33.300 
     34.500       7.500 
      3.697       0.483 
 
 Lib_L_27F-002  10.0 
      9.555       5.910 
      4.495       1.825 
      1.556       0.778 
 
 Lib_L_27F-002  24.0 
     19.752       9.721 
      8.370       3.540 
      1.955       0.961 
 
 Lib_L_27F-002  38.0 
     25.711      10.071 
     11.440       4.500 
      2.115       0.994 
 
 Lib_L_27F-002  52.0 
     28.549       7.834 
     14.145       5.185 
      2.251       1.042 
 
 Lib_L_27F-002  66.0 
     32.177      10.147 
     16.325       6.085 
      2.314       1.105 
 
 Lib_L_27F-002  80.0 
     35.698      13.846 
     18.330       6.440 
      2.370       1.091 
 
 Lib_L_27F-002  94.0 
     38.012      15.855 
     20.100       6.850 
      2.426       1.099 
 
 Lib_L_27F-002  108.0 
     42.961      21.122 
     21.640       7.270 
      2.444       1.099 
 
 Lib_L_27F-002  122.0 
     47.745      25.712 
     23.305       8.025 
      2.473       1.127 
 
 Lib_L_27F-002  136.0 
     49.270      27.195 
     24.610       8.390 
      2.497       1.143 
 
 Lib_L_27F-002  150.0 
     52.083      30.417 
     26.000       9.000 
      2.520       1.146 
 
 Lib_L_27F-003  10.0 
     13.852       0.621 
      6.485       0.295 
      2.415       0.148 
 
 Lib_L_27F-003  24.0 
     31.606       2.256 
     12.280       0.460 
      3.045       0.229 
 
 Lib_L_27F-003  38.0 
     44.309       4.321 
     17.060       0.340 
      3.344       0.161 
 
 Lib_L_27F-003  52.0 
     62.543       4.397 
     20.940       0.410 
      3.473       0.179 
 
 Lib_L_27F-003  66.0 
     73.354       5.814 
     24.875       0.595 
      3.575       0.204 
 
 Lib_L_27F-003  80.0 
     82.456       5.539 
     28.360       1.020 
      3.678       0.171 
 
 Lib_L_27F-003  94.0 
     85.562       9.794 
     31.590       1.060 
      3.729       0.187 
 
 Lib_L_27F-003  108.0 
     94.987      23.163 
     34.685       1.785 
      3.789       0.165 
 
 Lib_L_27F-003  122.0 
     97.999      22.715 
     37.870       1.820 
      3.832       0.171 
 
 Lib_L_27F-003  136.0 
     98.283      26.005 
     40.485       2.015 
      3.863       0.177 
 
 Lib_L_27F-003  150.0 
    102.500      31.500 
     43.500       2.500 
      3.909       0.168 
 
 Lib_L_27F-004  10.0 
      7.778       5.988 
      4.170       2.560 
      1.416       1.123 
 
 Lib_L_27F-004  24.0 
     13.561      10.301 
      7.170       4.620 
      1.771       1.357 
 
 Lib_L_27F-004  38.0 
     20.921      16.221 
      9.655       6.375 
      1.927       1.479 
 
 Lib_L_27F-004  52.0 
     28.526      20.516 
     11.960       7.440 
      2.036       1.501 
 
 Lib_L_27F-004  66.0 
     35.461      25.231 
     13.725       8.545 
      2.070       1.539 
 
 Lib_L_27F-004  80.0 
     37.450      26.080 
     15.165       9.485 
      2.090       1.556 
 
 Lib_L_27F-004  94.0 
     40.667      27.107 
     17.045      10.685 
      2.145       1.608 
 
 Lib_L_27F-004  108.0 
     43.969      27.184 
     18.535      11.555 
      2.165       1.612 
 
 Lib_L_27F-004  122.0 
     47.169      26.544 
     20.115      12.525 
      2.193       1.637 
 
 Lib_L_27F-004  136.0 
     51.210      25.520 
     21.575      13.215 
      2.212       1.638 
 
 Lib_L_27F-004  150.0 
     54.583      24.583 
     23.000      14.000 
      2.229       1.651 
 
 Lib_L_27F-005  10.0 
     15.672       0.657 
      6.795       0.285 
      2.515       0.100 
 
 Lib_L_27F-005  24.0 
     31.877       0.798 
     12.475       0.065 
      3.106       0.063 
 
 Lib_L_27F-005  38.0 
     51.353       0.803 
     17.875       0.365 
      3.440       0.126 
 
 Lib_L_27F-005  52.0 
     68.829      10.135 
     21.780       0.090 
      3.577       0.078 
 
 Lib_L_27F-005  66.0 
     84.699      15.325 
     25.665       0.095 
      3.657       0.116 
 
 Lib_L_27F-005  80.0 
     98.848      18.753 
     29.470       0.140 
      3.778       0.102 
 
 Lib_L_27F-005  94.0 
    111.331      22.286 
     32.790       0.180 
      3.830       0.125 
 
 Lib_L_27F-005  108.0 
    123.505      26.798 
     36.180       0.200 
      3.882       0.132 
 
 Lib_L_27F-005  122.0 
    149.782      48.166 
     39.635       0.635 
      3.938       0.100 
 
 Lib_L_27F-005  136.0 
    152.325      46.519 
     42.780       0.800 
      3.986       0.107 
 
 Lib_L_27F-005  150.0 
    161.000      43.500 
     46.000       1.000 
      4.028       0.106 
 
 Lib_L_27F-006  10.0 
     19.078       1.969 
      7.520       0.460 
      2.740       0.129 
 
 Lib_L_27F-006  24.0 
     34.822       7.304 
     14.010       1.300 
      3.439       0.213 
 
 Lib_L_27F-006  38.0 
     49.158      14.641 
     19.150       2.200 
      3.752       0.257 
 
 Lib_L_27F-006  52.0 
     63.795      14.581 
     23.975       2.455 
      3.942       0.236 
 
 Lib_L_27F-006  66.0 
     79.329      21.069 
     28.040       3.600 
      4.075       0.290 
 
 Lib_L_27F-006  80.0 
     81.364      19.160 
     31.790       4.280 
      4.180       0.287 
 
 Lib_L_27F-006  94.0 
     86.063      23.151 
     35.160       5.100 
      4.235       0.314 
 
 Lib_L_27F-006  108.0 
     87.490      18.093 
     38.490       5.330 
      4.300       0.303 
 
 Lib_L_27F-006  122.0 
     93.829      22.778 
     41.670       6.330 
      4.355       0.320 
 
 Lib_L_27F-006  136.0 
     92.828      21.874 
     44.535       6.835 
      4.397       0.326 
 
 Lib_L_27F-006  150.0 
     94.812      23.188 
     47.500       7.500 
      4.438       0.336 
 
 Lib_L_27F-007  10.0 
     20.633       2.937 
      7.335       0.415 
      2.645       0.116 
 
 Lib_L_27F-007  24.0 
     50.670      15.392 
     14.345       1.275 
      3.319       0.205 
 
 Lib_L_27F-007  38.0 
     64.925      22.167 
     20.750       2.490 
      3.695       0.281 
 
 Lib_L_27F-007  52.0 
     80.727      29.606 
     25.980       3.450 
      3.856       0.285 
 
 Lib_L_27F-007  66.0 
     82.486      22.731 
     31.335       4.555 
      4.067       0.316 
 
 Lib_L_27F-007  80.0 
     90.762      24.615 
     35.610       5.320 
      4.126       0.314 
 
 Lib_L_27F-007  94.0 
     93.183      18.301 
     39.750       6.360 
      4.223       0.346 
 
 Lib_L_27F-007  108.0 
     92.563      18.265 
     43.615       7.675 
      4.310       0.373 
 
 Lib_L_27F-007  122.0 
     95.436      14.650 
     47.005       8.315 
      4.354       0.371 
 
 Lib_L_27F-007  136.0 
     96.542      10.443 
     50.365       9.075 
      4.402       0.390 
 
 Lib_L_27F-007  150.0 
     98.508       8.080 
     53.500       9.500 
      4.445       0.387 
 
 Lib_L_27F-008  10.0 
     17.460       6.485 
      6.620       1.360 
      2.386       0.461 
 
 Lib_L_27F-008  24.0 
     42.991      16.439 
     13.180       3.380 
      3.042       0.699 
 
 Lib_L_27F-008  38.0 
     66.436      26.178 
     19.000       4.650 
      3.371       0.729 
 
 Lib_L_27F-008  52.0 
     92.498      42.563 
     24.410       6.600 
      3.586       0.804 
 
 Lib_L_27F-008  66.0 
     96.583      40.000 
     29.060       8.060 
      3.708       0.857 
 
 Lib_L_27F-008  80.0 
    104.188      38.517 
     33.660       9.160 
      3.822       0.855 
 
 Lib_L_27F-008  94.0 
    113.201      44.445 
     38.170      10.860 
      3.928       0.895 
 
 Lib_L_27F-008  108.0 
    116.130      45.003 
     41.915      12.415 
      3.983       0.926 
 
 Lib_L_27F-008  122.0 
    124.179      50.274 
     45.990      13.590 
      4.048       0.935 
 
 Lib_L_27F-008  136.0 
    125.593      48.102 
     49.430      14.610 
      4.089       0.944 
 
 Lib_L_27F-008  150.0 
    128.396      51.968 
     53.000      16.000 
      4.135       0.964 
 
 Lib_L_27F-009  10.0 
     18.001       7.524 
      7.320       1.160 
      2.695       0.297 
 
 Lib_L_27F-009  24.0 
     35.950      10.437 
     13.980       3.280 
      3.408       0.508 
 
 Lib_L_27F-009  38.0 
     51.003      14.120 
     19.060       4.750 
      3.701       0.570 
 
 Lib_L_27F-009  52.0 
     61.406      10.036 
     23.625       6.295 
      3.887       0.645 
 
 Lib_L_27F-009  66.0 
     77.067       0.388 
     27.395       7.065 
      3.996       0.666 
 
 Lib_L_27F-009  80.0 
     95.950       5.680 
     31.275       8.115 
      4.089       0.711 
 
 Lib_L_27F-009  94.0 
    107.537      15.095 
     34.710       8.600 
      4.162       0.715 
 
 Lib_L_27F-009  108.0 
    113.787      20.741 
     37.915       8.855 
      4.221       0.723 
 
 Lib_L_27F-009  122.0 
    121.365      25.813 
     41.330       9.560 
      4.284       0.744 
 
 Lib_L_27F-009  136.0 
    112.131      17.727 
     44.020       9.710 
      4.314       0.743 
 
 Lib_L_27F-009  150.0 
    114.917      16.583 
     47.000      10.000 
      4.356       0.754 
 
 Lib_L_27F-010  10.0 
      9.104       3.542 
      5.210       1.040 
      2.031       0.325 
 
 Lib_L_27F-010  24.0 
     18.985       9.539 
      8.900       2.780 
      2.461       0.500 
 
 Lib_L_27F-010  38.0 
     25.495      11.830 
     11.725       3.585 
      2.640       0.479 
 
 Lib_L_27F-010  52.0 
     30.688      15.129 
     14.250       4.930 
      2.744       0.565 
 
 Lib_L_27F-010  66.0 
     37.897      21.561 
     16.720       6.230 
      2.843       0.595 
 
 Lib_L_27F-010  80.0 
     41.840      25.246 
     18.640       6.990 
      2.883       0.596 
 
 Lib_L_27F-010  94.0 
     47.942      30.115 
     20.705       8.245 
      2.937       0.639 
 
 Lib_L_27F-010  108.0 
     52.729      34.319 
     22.370       8.990 
      2.955       0.638 
 
 Lib_L_27F-010  122.0 
     57.440      40.157 
     24.055      10.095 
      2.999       0.657 
 
 Lib_L_27F-010  136.0 
     58.527      41.255 
     25.625      11.095 
      3.021       0.662 
 
 Lib_L_27F-010  150.0 
     60.500      43.500 
     27.000      12.000 
      3.036       0.675 
 
 Lib_L_27F-011  10.0 
      6.966       0.654 
      4.780       0.560 
      1.905       0.254 
 
 Lib_L_27F-011  24.0 
     13.325       0.955 
      7.780       0.720 
      2.300       0.287 
 
 Lib_L_27F-011  38.0 
     20.970       3.985 
     10.045       1.185 
      2.440       0.346 
 
 Lib_L_27F-011  52.0 
     30.595       7.556 
     12.330       1.440 
      2.553       0.359 
 
 Lib_L_27F-011  66.0 
     40.829      15.169 
     14.125       1.955 
      2.608       0.382 
 
 Lib_L_27F-011  80.0 
     48.182      20.119 
     15.800       2.320 
      2.640       0.393 
 
 Lib_L_27F-011  94.0 
     52.489      20.380 
     17.305       2.805 
      2.660       0.405 
 
 Lib_L_27F-011  108.0 
     60.776      17.755 
     19.215       3.185 
      2.704       0.408 
 
 Lib_L_27F-011  122.0 
     65.603      14.608 
     20.960       3.300 
      2.736       0.396 
 
 Lib_L_27F-011  136.0 
     67.854      11.805 
     22.485       3.695 
      2.760       0.399 
 
 Lib_L_27F-011  150.0 
     67.250       8.250 
     24.000       4.000 
      2.774       0.403 
 
 Lib_L_27F-013  10.0 
      5.782       0.298 
      4.215       0.395 
      1.730       0.209 
 
 Lib_L_27F-013  24.0 
     11.706       0.352 
      6.880       0.310 
      2.091       0.219 
 
 Lib_L_27F-013  38.0 
     15.501       0.633 
      8.375       0.085 
      2.151       0.196 
 
 Lib_L_27F-013  52.0 
     21.151       3.182 
     10.210       0.370 
      2.234       0.244 
 
 Lib_L_27F-013  66.0 
     26.133       3.403 
     12.110       0.210 
      2.329       0.210 
 
 Lib_L_27F-013  80.0 
     29.066       4.657 
     13.120       0.350 
      2.338       0.208 
 
 Lib_L_27F-013  94.0 
     32.040       3.527 
     14.415       0.485 
      2.352       0.217 
 
 Lib_L_27F-013  108.0 
     31.360       4.345 
     15.530       0.540 
      2.380       0.207 
 
 Lib_L_27F-013  122.0 
     33.020       1.057 
     16.835       0.775 
      2.411       0.218 
 
 Lib_L_27F-013  136.0 
     35.071       0.624 
     17.975       1.025 
      2.420       0.226 
 
 Lib_L_27F-013  150.0 
     33.100       0.100 
     19.000       1.000 
      2.436       0.222 
 
 Lib_L_27F-014  10.0 
      7.586       1.472 
      4.905       0.495 
      1.946       0.164 
 
 Lib_L_27F-014  24.0 
     17.339       4.630 
      8.175       1.035 
      2.305       0.222 
 
 Lib_L_27F-014  38.0 
     26.944      10.699 
     10.805       1.645 
      2.447       0.249 
 
 Lib_L_27F-014  52.0 
     39.826      18.344 
     13.595       2.005 
      2.592       0.238 
 
 Lib_L_27F-014  66.0 
     44.592      17.558 
     15.430       2.390 
      2.613       0.249 
 
 Lib_L_27F-014  80.0 
     51.729      22.093 
     17.985       3.155 
      2.699       0.275 
 
 Lib_L_27F-014  94.0 
     60.571      25.541 
     19.810       3.550 
      2.730       0.271 
 
 Lib_L_27F-014  108.0 
     64.120      28.214 
     21.610       4.320 
      2.756       0.281 
 
 Lib_L_27F-014  122.0 
     61.313      22.160 
     23.465       4.905 
      2.783       0.298 
 
 Lib_L_27F-014  136.0 
     64.917      25.796 
     25.270       5.560 
      2.810       0.304 
 
 Lib_L_27F-014  150.0 
     62.850      25.350 
     27.000       6.000 
      2.836       0.303 
 
 Lib_L_27F-015  10.0 
     10.153       2.322 
      5.610       1.010 
      2.146       0.374 
 
 Lib_L_27F-015  24.0 
     26.169       5.832 
      9.895       1.635 
      2.651       0.416 
 
 Lib_L_27F-015  38.0 
     37.459       6.772 
     13.510       2.180 
      2.868       0.457 
 
 Lib_L_27F-015  52.0 
     46.410      14.415 
     16.345       2.845 
      2.953       0.484 
 
 Lib_L_27F-015  66.0 
     55.341      16.577 
     19.260       3.440 
      3.035       0.504 
 
 Lib_L_27F-015  80.0 
     60.135      14.083 
     22.215       3.635 
      3.121       0.502 
 
 Lib_L_27F-015  94.0 
     66.838      14.995 
     24.410       4.140 
      3.138       0.510 
 
 Lib_L_27F-015  108.0 
     70.470       8.306 
     27.280       4.750 
      3.213       0.513 
 
 Lib_L_27F-015  122.0 
     69.456       3.628 
     29.515       5.255 
      3.240       0.536 
 
 Lib_L_27F-015  136.0 
     78.142       0.770 
     31.950       5.740 
      3.277       0.533 
 
 Lib_L_27F-015  150.0 
     78.750       6.250 
     34.000       6.000 
      3.301       0.538 
 
 Lib_L_27F-016  10.0 
      6.463       2.007 
      4.540       1.000 
      1.838       0.362 
 
 Lib_L_27F-016  24.0 
     12.821       1.911 
      7.125       1.445 
      2.141       0.446 
 
 Lib_L_27F-016  38.0 
     22.025       2.340 
      9.485       1.835 
      2.296       0.493 
 
 Lib_L_27F-016  52.0 
     36.536       7.134 
     11.880       2.150 
      2.393       0.504 
 
 Lib_L_27F-016  66.0 
     48.547      15.447 
     14.035       2.505 
      2.464       0.519 
 
 Lib_L_27F-016  80.0 
     59.859      18.851 
     16.045       2.565 
      2.513       0.512 
 
 Lib_L_27F-016  94.0 
     62.361      23.134 
     17.390       2.790 
      2.504       0.516 
 
 Lib_L_27F-016  108.0 
     73.183      30.723 
     19.585       3.145 
      2.552       0.527 
 
 Lib_L_27F-016  122.0 
     79.302      31.858 
     21.450       3.410 
      2.586       0.527 
 
 Lib_L_27F-016  136.0 
     76.243      29.419 
     23.270       3.700 
      2.612       0.533 
 
 Lib_L_27F-016  150.0 
     76.625      29.375 
     25.000       4.000 
      2.630       0.533 
 
 Lib_L_27F-017  10.0 
      4.681       0.301 
      3.335       0.555 
      1.208       0.397 
 
 Lib_L_27F-017  24.0 
     11.633       0.258 
      5.750       0.750 
      1.476       0.472 
 
 Lib_L_27F-017  38.0 
     18.587       0.699 
      7.705       0.915 
      1.571       0.508 
 
 Lib_L_27F-017  52.0 
     24.861       1.628 
      9.665       0.635 
      1.657       0.473 
 
 Lib_L_27F-017  66.0 
     29.139       0.347 
     11.260       0.850 
      1.684       0.501 
 
 Lib_L_27F-017  80.0 
     35.267       1.049 
     13.125       0.635 
      1.757       0.492 
 
 Lib_L_27F-017  94.0 
     44.065       1.097 
     14.885       0.515 
      1.787       0.484 
 
 Lib_L_27F-017  108.0 
     44.623       0.404 
     16.340       0.710 
      1.809       0.497 
 
 Lib_L_27F-017  122.0 
     46.704       0.397 
     17.690       0.770 
      1.822       0.499 
 
 Lib_L_27F-017  136.0 
     48.787       4.591 
     19.075       0.525 
      1.836       0.493 
 
 Lib_L_27F-017  150.0 
     49.375       5.625 
     20.500       0.500 
      1.857       0.489 
 
 Lib_L_27F-018  10.0 
      3.687       0.352 
      2.970       0.450 
      1.068       0.350 
 
 Lib_L_27F-018  24.0 
      7.828       0.430 
      4.720       0.330 
      1.253       0.373 
 
 Lib_L_27F-018  38.0 
     11.572       1.462 
      6.350       0.220 
      1.392       0.407 
 
 Lib_L_27F-018  52.0 
     14.664       2.880 
      7.750       0.130 
      1.452       0.384 
 
 Lib_L_27F-018  66.0 
     18.381       2.858 
      9.065       0.295 
      1.481       0.382 
 
 Lib_L_27F-018  80.0 
     20.955       1.767 
     10.320       0.600 
      1.528       0.363 
 
 Lib_L_27F-018  94.0 
     22.184       1.916 
     11.200       0.640 
      1.535       0.375 
 
 Lib_L_27F-018  108.0 
     23.674       0.866 
     12.210       0.570 
      1.549       0.398 
 
 Lib_L_27F-018  122.0 
     26.841       0.860 
     13.280       0.670 
      1.578       0.387 
 
 Lib_L_27F-018  136.0 
     26.764       3.067 
     14.080       0.880 
      1.586       0.383 
 
 Lib_L_27F-018  150.0 
     27.167       3.833 
     15.000       1.000 
      1.603       0.378 
 
 Lib_L_27F-019  10.0 
      5.418       0.978 
      3.630       0.530 
      1.306       0.298 
 
 Lib_L_27F-019  24.0 
     13.196       2.057 
      6.580       0.840 
      1.660       0.376 
 
 Lib_L_27F-019  38.0 
     17.124       3.198 
      8.580       1.060 
      1.761       0.385 
 
 Lib_L_27F-019  52.0 
     19.374       3.261 
     10.515       1.395 
      1.863       0.400 
 
 Lib_L_27F-019  66.0 
     21.689       4.629 
     12.195       1.725 
      1.939       0.406 
 
 Lib_L_27F-019  80.0 
     22.639       3.110 
     13.450       1.690 
      1.963       0.402 
 
 Lib_L_27F-019  94.0 
     24.913       3.266 
     14.500       1.670 
      1.979       0.394 
 
 Lib_L_27F-019  108.0 
     26.985       4.684 
     15.575       1.975 
      1.996       0.402 
 
 Lib_L_27F-019  122.0 
     28.677       6.825 
     16.575       2.355 
      2.023       0.415 
 
 Lib_L_27F-019  136.0 
     31.760       7.170 
     17.615       2.335 
      2.038       0.413 
 
 Lib_L_27F-019  150.0 
     32.333       7.000 
     18.500       2.500 
      2.051       0.417 
 
 Lib_L_27F-020  10.0 
      3.552       2.182 
      2.565       1.215 
      0.788       0.624 
 
 Lib_L_27F-020  24.0 
      6.214       4.504 
      3.900       2.270 
      0.922       0.760 
 
 Lib_L_27F-020  38.0 
     10.636       8.106 
      5.275       3.115 
      1.029       0.816 
 
 Lib_L_27F-020  52.0 
     12.002       8.597 
      6.160       3.530 
      1.054       0.817 
 
 Lib_L_27F-020  66.0 
     15.259      11.099 
      7.315       4.275 
      1.091       0.840 
 
 Lib_L_27F-020  80.0 
     18.866      13.421 
      8.480       4.940 
      1.129       0.865 
 
 Lib_L_27F-020  94.0 
     18.905      13.670 
      9.055       5.395 
      1.128       0.874 
 
 Lib_L_27F-020  108.0 
     21.676      15.401 
      9.960       5.850 
      1.143       0.880 
 
 Lib_L_27F-020  122.0 
     24.105      17.250 
     10.820       6.320 
      1.155       0.883 
 
 Lib_L_27F-020  136.0 
     25.255      18.709 
     11.855       7.135 
      1.182       0.909 
 
 Lib_L_27F-020  150.0 
     26.250      19.750 
     12.500       7.500 
      1.186       0.911 
 
 Lib_L_27F-021  10.0 
     12.024       5.093 
      5.650       1.450 
      2.098       0.504 
 
 Lib_L_27F-021  24.0 
     24.716       5.753 
     10.405       2.785 
      2.606       0.600 
 
 Lib_L_27F-021  38.0 
     36.671       4.321 
     14.315       3.755 
      2.832       0.668 
 
 Lib_L_27F-021  52.0 
     46.357       0.559 
     17.470       4.250 
      2.938       0.702 
 
 Lib_L_27F-021  66.0 
     52.880       1.234 
     20.675       4.905 
      3.055       0.721 
 
 Lib_L_27F-021  80.0 
     58.577       3.992 
     23.360       5.010 
      3.124       0.742 
 
 Lib_L_27F-021  94.0 
     67.617       4.522 
     26.035       4.975 
      3.184       0.743 
 
 Lib_L_27F-021  108.0 
     75.352      11.864 
     28.275       5.085 
      3.219       0.747 
 
 Lib_L_27F-021  122.0 
     78.008      14.556 
     30.650       5.130 
      3.264       0.759 
 
 Lib_L_27F-021  136.0 
     80.486      14.123 
     32.890       5.060 
      3.294       0.763 
 
 Lib_L_27F-021  150.0 
     79.750      13.500 
     35.000       5.000 
      3.324       0.763 
 
 Lib_L_27F-022  10.0 
     14.395       0.750 
      6.675       0.465 
      2.499       0.168 
 
 Lib_L_27F-022  24.0 
     26.945       2.542 
     12.535       0.615 
      3.205       0.212 
 
 Lib_L_27F-022  38.0 
     37.550       6.654 
     16.475       0.205 
      3.426       0.185 
 
 Lib_L_27F-022  52.0 
     42.538      10.243 
     20.000       0.290 
      3.590       0.180 
 
 Lib_L_27F-022  66.0 
     51.483      11.808 
     23.510       0.820 
      3.699       0.164 
 
 Lib_L_27F-022  80.0 
     57.213      14.567 
     25.970       1.500 
      3.761       0.164 
 
 Lib_L_27F-022  94.0 
     55.317      14.062 
     28.215       2.005 
      3.808       0.157 
 
 Lib_L_27F-022  108.0 
     58.084      11.662 
     30.620       2.020 
      3.855       0.171 
 
 Lib_L_27F-022  122.0 
     59.713      14.559 
     33.090       2.870 
      3.911       0.154 
 
 Lib_L_27F-022  136.0 
     59.241      14.304 
     34.985       3.395 
      3.939       0.149 
 
 Lib_L_27F-022  150.0 
     59.354      14.979 
     37.000       4.000 
      3.972       0.141 
 
 Lib_L_27F-023  10.0 
     16.553         nan 
      7.380         nan 
      2.714         nan 
 
 Lib_L_27F-023  24.0 
     30.223         nan 
     13.790         nan 
      3.473         nan 
 
 Lib_L_27F-023  38.0 
     46.002         nan 
     18.350         nan 
      3.733         nan 
 
 Lib_L_27F-023  52.0 
     58.554         nan 
     22.270         nan 
      3.885         nan 
 
 Lib_L_27F-023  66.0 
     74.299         nan 
     26.090         nan 
      4.029         nan 
 
 Lib_L_27F-023  80.0 
     84.936         nan 
     28.970         nan 
      4.081         nan 
 
 Lib_L_27F-023  94.0 
     91.991         nan 
     32.310         nan 
      4.146         nan 
 
 Lib_L_27F-023  108.0 
    104.275         nan 
     35.730         nan 
      4.229         nan 
 
 Lib_L_27F-023  122.0 
    107.468         nan 
     38.650         nan 
      4.268         nan 
 
 Lib_L_27F-023  136.0 
    109.182         nan 
     41.340         nan 
      4.304         nan 
 
 Lib_L_27F-023  150.0 
    111.667         nan 
     44.000         nan 
      4.336         nan 
 
 Lib_L_27F-024  10.0 
     10.080       7.990 
      4.455       2.665 
      1.479       1.113 
 
 Lib_L_27F-024  24.0 
     20.774      17.079 
      8.220       5.590 
      1.855       1.443 
 
 Lib_L_27F-024  38.0 
     25.132      19.092 
     11.240       7.640 
      2.014       1.543 
 
 Lib_L_27F-024  52.0 
     29.847      22.219 
     13.995       9.705 
      2.125       1.642 
 
 Lib_L_27F-024  66.0 
     31.830      21.885 
     16.310      11.140 
      2.216       1.703 
 
 Lib_L_27F-024  80.0 
     35.308      23.114 
     18.480      12.280 
      2.283       1.725 
 
 Lib_L_27F-024  94.0 
     39.299      27.360 
     20.200      13.590 
      2.303       1.755 
 
 Lib_L_27F-024  108.0 
     40.281      28.378 
     21.855      14.515 
      2.343       1.767 
 
 Lib_L_27F-024  122.0 
     42.303      30.319 
     23.470      15.590 
      2.361       1.790 
 
 Lib_L_27F-024  136.0 
     43.564      31.937 
     25.115      16.615 
      2.398       1.808 
 
 Lib_L_27F-024  150.0 
     43.083      31.583 
     26.500      17.500 
      2.415       1.823 
 
 Lib_L_27F-025  10.0 
     11.870       1.409 
      6.340       0.220 
      2.433       0.048 
 
 Lib_L_27F-025  24.0 
     26.020       1.520 
     11.205       0.435 
      3.013       0.052 
 
 Lib_L_27F-025  38.0 
     43.264       0.412 
     15.395       0.905 
      3.276       0.099 
 
 Lib_L_27F-025  52.0 
     56.639       8.710 
     18.825       0.965 
      3.403       0.104 
 
 Lib_L_27F-025  66.0 
     63.268      10.779 
     22.125       0.935 
      3.505       0.094 
 
 Lib_L_27F-025  80.0 
     72.756      14.871 
     25.385       1.215 
      3.585       0.118 
 
 Lib_L_27F-025  94.0 
     73.953      15.108 
     27.715       1.015 
      3.614       0.105 
 
 Lib_L_27F-025  108.0 
     76.861      13.631 
     30.730       0.630 
      3.693       0.098 
 
 Lib_L_27F-025  122.0 
     82.070      14.928 
     33.320       0.620 
      3.720       0.105 
 
 Lib_L_27F-025  136.0 
     83.361      14.609 
     35.785       0.105 
      3.764       0.093 
 
 Lib_L_27F-025  150.0 
     86.500      10.000 
     38.000         nan 
      3.789       0.097 
 
 Lib_L_27F-026  10.0 
      5.438       1.698 
      3.370       0.660 
      1.111       0.296 
 
 Lib_L_27F-026  24.0 
     11.168       2.986 
      5.990       1.170 
      1.383       0.308 
 
 Lib_L_27F-026  38.0 
     14.776       2.100 
      8.160       1.600 
      1.514       0.364 
 
 Lib_L_27F-026  52.0 
     18.086       3.092 
     10.155       2.255 
      1.628       0.418 
 
 Lib_L_27F-026  66.0 
     20.213       2.159 
     11.390       2.460 
      1.648       0.435 
 
 Lib_L_27F-026  80.0 
     23.200       1.711 
     12.680       2.580 
      1.673       0.439 
 
 Lib_L_27F-026  94.0 
     25.342       2.310 
     13.875       2.895 
      1.715       0.462 
 
 Lib_L_27F-026  108.0 
     30.792       2.002 
     14.970       2.930 
      1.735       0.452 
 
 Lib_L_27F-026  122.0 
     33.882       2.897 
     15.925       2.915 
      1.744       0.454 
 
 Lib_L_27F-026  136.0 
     38.670       3.628 
     17.010       2.930 
      1.757       0.458 
 
 Lib_L_27F-026  150.0 
     43.000       5.500 
     18.000       3.000 
      1.769       0.457 
 
 Lib_L_27F-027  10.0 
      6.194       1.156 
      3.700       0.400 
      1.271       0.172 
 
 Lib_L_27F-027  24.0 
     12.026       2.586 
      6.365       0.825 
      1.532       0.222 
 
 Lib_L_27F-027  38.0 
     16.382       1.734 
      8.835       1.045 
      1.706       0.230 
 
 Lib_L_27F-027  52.0 
     20.673       4.441 
     10.720       1.680 
      1.782       0.309 
 
 Lib_L_27F-027  66.0 
     24.249       5.071 
     12.345       2.115 
      1.839       0.325 
 
 Lib_L_27F-027  80.0 
     28.374       5.197 
     13.925       2.325 
      1.880       0.330 
 
 Lib_L_27F-027  94.0 
     33.066       4.007 
     15.315       2.175 
      1.918       0.287 
 
 Lib_L_27F-027  108.0 
     36.654       2.133 
     16.425       2.435 
      1.923       0.294 
 
 Lib_L_27F-027  122.0 
     39.754       0.079 
     17.590       2.790 
      1.942       0.308 
 
 Lib_L_27F-027  136.0 
     45.260       7.239 
     18.880       2.780 
      1.967       0.310 
 
 Lib_L_27F-027  150.0 
     55.300      16.700 
     20.000       3.000 
      1.979       0.318 
 
 Lib_L_27F-028  10.0 
      5.753       1.333 
      3.760       0.690 
      1.371       0.384 
 
 Lib_L_27F-028  24.0 
     13.595       4.083 
      6.535       1.215 
      1.682       0.429 
 
 Lib_L_27F-028  38.0 
     22.570       9.303 
      9.400       1.980 
      1.910       0.499 
 
 Lib_L_27F-028  52.0 
     29.946      12.190 
     11.400       2.400 
      1.957       0.475 
 
 Lib_L_27F-028  66.0 
     36.219      14.371 
     13.510       3.110 
      2.027       0.506 
 
 Lib_L_27F-028  80.0 
     38.257      14.505 
     15.480       3.700 
      2.105       0.528 
 
 Lib_L_27F-028  94.0 
     40.663      14.525 
     17.240       4.220 
      2.131       0.533 
 
 Lib_L_27F-028  108.0 
     42.081      15.067 
     18.690       4.770 
      2.147       0.548 
 
 Lib_L_27F-028  122.0 
     43.565      13.403 
     20.065       4.885 
      2.153       0.522 
 
 Lib_L_27F-028  136.0 
     42.117      12.391 
     21.515       5.545 
      2.170       0.542 
 
 Lib_L_27F-028  150.0 
     42.714      10.714 
     23.000       6.000 
      2.198       0.550 
 
 Lib_L_27F-029  10.0 
      8.501       0.448 
      4.985       0.115 
      1.909       0.100 
 
 Lib_L_27F-029  24.0 
     16.207       0.072 
      8.620       0.130 
      2.366       0.066 
 
 Lib_L_27F-029  38.0 
     22.214       0.098 
     11.075       0.225 
      2.465       0.066 
 
 Lib_L_27F-029  52.0 
     29.202       3.445 
     13.660       0.150 
      2.599       0.060 
 
 Lib_L_27F-029  66.0 
     36.419       5.793 
     15.855       0.015 
      2.666       0.069 
 
 Lib_L_27F-029  80.0 
     38.133       4.665 
     17.880       0.060 
      2.730       0.067 
 
 Lib_L_27F-029  94.0 
     38.897       4.028 
     19.295       0.095 
      2.753       0.065 
 
 Lib_L_27F-029  108.0 
     42.920       5.111 
     21.090       0.120 
      2.800       0.068 
 
 Lib_L_27F-029  122.0 
     45.406       6.046 
     22.750       0.290 
      2.834       0.065 
 
 Lib_L_27F-029  136.0 
     48.027       7.136 
     24.130       0.320 
      2.852       0.063 
 
 Lib_L_27F-029  150.0 
     48.300       7.700 
     25.500       0.500 
      2.872       0.068 
 
 Lib_L_27F-030  10.0 
      9.178       0.011 
      5.110       0.250 
      1.958       0.161 
 
 Lib_L_27F-030  24.0 
     19.156       2.159 
      8.895       0.305 
      2.372       0.176 
 
 Lib_L_27F-030  38.0 
     28.527       5.457 
     12.225       0.165 
      2.605       0.130 
 
 Lib_L_27F-030  52.0 
     36.463       8.990 
     15.145       0.535 
      2.720       0.170 
 
 Lib_L_27F-030  66.0 
     43.090       8.959 
     17.615       0.825 
      2.811       0.158 
 
 Lib_L_27F-030  80.0 
     45.056      12.331 
     19.615       1.275 
      2.849       0.183 
 
 Lib_L_27F-030  94.0 
     48.802      13.094 
     21.985       1.875 
      2.914       0.198 
 
 Lib_L_27F-030  108.0 
     50.367      11.185 
     23.835       2.065 
      2.938       0.188 
 
 Lib_L_27F-030  122.0 
     52.592       8.221 
     25.755       2.515 
      2.966       0.204 
 
 Lib_L_27F-030  136.0 
     51.097       5.961 
     27.295       2.655 
      2.986       0.200 
 
 Lib_L_27F-030  150.0 
     52.250       3.500 
     29.000       3.000 
      3.011       0.206 
 
 Lib_L_27F-031  10.0 
     11.589       5.136 
      5.465       1.115 
      2.063       0.330 
 
 Lib_L_27F-031  24.0 
     33.209      17.786 
     10.505       3.035 
      2.589       0.512 
 
 Lib_L_27F-031  38.0 
     45.556      24.088 
     14.630       4.740 
      2.793       0.593 
 
 Lib_L_27F-031  52.0 
     61.359      35.834 
     18.505       6.465 
      2.937       0.664 
 
 Lib_L_27F-031  66.0 
     64.828      35.957 
     21.735       7.815 
      3.029       0.692 
 
 Lib_L_27F-031  80.0 
     73.535      45.538 
     25.205       9.795 
      3.116       0.745 
 
 Lib_L_27F-031  94.0 
     75.405      47.170 
     27.705      10.865 
      3.143       0.743 
 
 Lib_L_27F-031  108.0 
     74.205      41.152 
     30.530      12.080 
      3.192       0.754 
 
 Lib_L_27F-031  122.0 
     77.181      43.356 
     33.405      13.835 
      3.251       0.804 
 
 Lib_L_27F-031  136.0 
     77.449      46.441 
     36.095      15.205 
      3.297       0.807 
 
 Lib_L_27F-031  150.0 
     76.765      45.598 
     38.500      16.500 
      3.325       0.818 
 
 Lib_L_27F-032  10.0 
     10.048       4.628 
      5.240       1.750 
      1.917       0.703 
 
 Lib_L_27F-032  24.0 
     20.644       9.940 
      9.425       3.245 
      2.424       0.866 
 
 Lib_L_27F-032  38.0 
     36.923      20.182 
     13.030       4.880 
      2.651       0.960 
 
 Lib_L_27F-032  52.0 
     40.201      16.786 
     15.645       5.925 
      2.725       1.017 
 
 Lib_L_27F-032  66.0 
     45.519      17.566 
     18.555       7.205 
      2.831       1.066 
 
 Lib_L_27F-032  80.0 
     48.659      15.981 
     20.725       7.785 
      2.862       1.063 
 
 Lib_L_27F-032  94.0 
     49.289      16.080 
     23.215       9.015 
      2.928       1.101 
 
 Lib_L_27F-032  108.0 
     48.649      18.247 
     25.300       9.740 
      2.970       1.117 
 
 Lib_L_27F-032  122.0 
     47.737      17.712 
     27.115      10.085 
      3.003       1.104 
 
 Lib_L_27F-032  136.0 
     46.836      18.621 
     28.915      11.065 
      3.031       1.135 
 
 Lib_L_27F-032  150.0 
     46.583      18.417 
     30.500      11.500 
      3.047       1.138 
 
 Lib_L_27F-033  10.0 
     10.481       3.356 
      5.535       1.195 
      2.109       0.425 
 
 Lib_L_27F-033  24.0 
     20.024       6.135 
      9.515       2.265 
      2.574       0.513 
 
 Lib_L_27F-033  38.0 
     33.354      11.036 
     12.825       3.035 
      2.774       0.544 
 
 Lib_L_27F-033  52.0 
     41.720      15.108 
     15.460       4.090 
      2.868       0.604 
 
 Lib_L_27F-033  66.0 
     52.423      14.431 
     18.220       4.640 
      2.972       0.619 
 
 Lib_L_27F-033  80.0 
     67.780      18.313 
     20.580       5.070 
      3.018       0.612 
 
 Lib_L_27F-033  94.0 
     81.765      27.105 
     23.085       6.125 
      3.068       0.657 
 
 Lib_L_27F-033  108.0 
     90.929      34.559 
     25.725       6.995 
      3.122       0.692 
 
 Lib_L_27F-033  122.0 
    107.679      44.983 
     28.015       7.325 
      3.154       0.683 
 
 Lib_L_27F-033  136.0 
    107.388      45.714 
     30.305       7.905 
      3.180       0.691 
 
 Lib_L_27F-033  150.0 
    103.875      45.875 
     32.500       8.500 
      3.209       0.694 
 
 Lib_L_27F-034  10.0 
     17.783       8.417 
      7.295       1.385 
      2.687       0.356 
 
 Lib_L_27F-034  24.0 
     38.971      20.213 
     13.805       4.365 
      3.375       0.636 
 
 Lib_L_27F-034  38.0 
     51.157      17.400 
     19.040       6.080 
      3.680       0.677 
 
 Lib_L_27F-034  52.0 
     57.644      18.440 
     23.275       8.155 
      3.845       0.781 
 
 Lib_L_27F-034  66.0 
     65.556      13.250 
     27.690       9.930 
      3.981       0.845 
 
 Lib_L_27F-034  80.0 
     71.361      11.124 
     31.105      10.925 
      4.065       0.865 
 
 Lib_L_27F-034  94.0 
     73.601      11.988 
     34.525      12.195 
      4.139       0.892 
 
 Lib_L_27F-034  108.0 
     75.919      11.468 
     37.670      13.340 
      4.201       0.920 
 
 Lib_L_27F-034  122.0 
     81.277       7.864 
     40.955      13.715 
      4.269       0.917 
 
 Lib_L_27F-034  136.0 
     86.080       3.912 
     43.585      14.455 
      4.305       0.936 
 
 Lib_L_27F-034  150.0 
     85.926       2.426 
     46.000      15.000 
      4.339       0.949 
 
 Lib_L_27F-035  10.0 
      7.608       2.030 
      4.455       0.735 
      1.655       0.317 
 
 Lib_L_27F-035  24.0 
     16.804       3.752 
      7.745       1.545 
      2.010       0.433 
 
 Lib_L_27F-035  38.0 
     27.511       4.806 
     10.605       1.935 
      2.188       0.435 
 
 Lib_L_27F-035  52.0 
     35.776       6.349 
     13.050       2.510 
      2.277       0.487 
 
 Lib_L_27F-035  66.0 
     41.032       2.028 
     15.395       2.805 
      2.336       0.511 
 
 Lib_L_27F-035  80.0 
     51.609       3.780 
     17.740       2.690 
      2.398       0.472 
 
 Lib_L_27F-035  94.0 
     59.809       2.921 
     19.860       2.770 
      2.433       0.475 
 
 Lib_L_27F-035  108.0 
     61.324       8.588 
     21.710       3.120 
      2.471       0.484 
 
 Lib_L_27F-035  122.0 
     70.341       8.955 
     23.780       3.360 
      2.499       0.492 
 
 Lib_L_27F-035  136.0 
     75.396       5.067 
     25.645       3.385 
      2.526       0.489 
 
 Lib_L_27F-035  150.0 
     79.250       4.250 
     27.500       3.500 
      2.548       0.492 
 
 Lib_L_27F-036  10.0 
     10.202       5.547 
      5.410       1.750 
      2.087       0.573 
 
 Lib_L_27F-036  24.0 
     21.574      11.904 
      9.370       3.790 
      2.532       0.814 
 
 Lib_L_27F-036  38.0 
     36.515      18.470 
     12.785       5.235 
      2.722       0.904 
 
 Lib_L_27F-036  52.0 
     50.417      21.372 
     16.040       6.360 
      2.871       0.920 
 
 Lib_L_27F-036  66.0 
     61.886      21.376 
     19.040       7.690 
      2.951       0.988 
 
 Lib_L_27F-036  80.0 
     71.171      14.996 
     21.700       8.770 
      3.009       1.012 
 
 Lib_L_27F-036  94.0 
     91.124       8.284 
     24.595       9.455 
      3.082       1.020 
 
 Lib_L_27F-036  108.0 
    101.315       3.010 
     27.280      10.630 
      3.127       1.052 
 
 Lib_L_27F-036  122.0 
    114.136      16.724 
     29.640      11.220 
      3.153       1.060 
 
 Lib_L_27F-036  136.0 
    131.497      31.893 
     32.195      11.805 
      3.196       1.067 
 
 Lib_L_27F-036  150.0 
    145.833      47.167 
     34.500      12.500 
      3.220       1.080 
 
 Lib_L_27F-037  10.0 
     11.393       1.317 
      5.740       0.550 
      2.162       0.231 
 
 Lib_L_27F-037  24.0 
     26.952       4.639 
     10.510       1.090 
      2.683       0.299 
 
 Lib_L_27F-037  38.0 
     41.642       7.575 
     14.625       1.615 
      2.932       0.320 
 
 Lib_L_27F-037  52.0 
     50.576       8.769 
     18.165       2.125 
      3.047       0.349 
 
 Lib_L_27F-037  66.0 
     59.223      15.981 
     21.360       2.300 
      3.161       0.326 
 
 Lib_L_27F-037  80.0 
     67.036      12.812 
     24.685       2.905 
      3.240       0.357 
 
 Lib_L_27F-037  94.0 
     68.171      16.663 
     27.145       3.265 
      3.277       0.369 
 
 Lib_L_27F-037  108.0 
     66.460      16.574 
     30.060       3.760 
      3.348       0.376 
 
 Lib_L_27F-037  122.0 
     64.673      14.499 
     32.490       4.150 
      3.378       0.379 
 
 Lib_L_27F-037  136.0 
     64.188      14.297 
     34.780       4.540 
      3.414       0.382 
 
 Lib_L_27F-037  150.0 
     63.050      14.050 
     37.000       5.000 
      3.444       0.389 
 
 Lib_L_27F-038  10.0 
      7.508       1.782 
      4.260       0.590 
      1.546       0.239 
 
 Lib_L_27F-038  24.0 
     15.016       3.933 
      7.720       1.200 
      1.958       0.266 
 
 Lib_L_27F-038  38.0 
     19.550       3.218 
     10.125       1.495 
      2.091       0.261 
 
 Lib_L_27F-038  52.0 
     24.176       5.382 
     12.075       2.165 
      2.161       0.328 
 
 Lib_L_27F-038  66.0 
     28.102       5.916 
     14.095       2.785 
      2.246       0.369 
 
 Lib_L_27F-038  80.0 
     31.723       6.835 
     15.435       2.775 
      2.247       0.338 
 
 Lib_L_27F-038  94.0 
     35.858      10.542 
     17.175       3.365 
      2.313       0.364 
 
 Lib_L_27F-038  108.0 
     44.739      17.017 
     18.625       3.605 
      2.353       0.357 
 
 Lib_L_27F-038  122.0 
     49.672      19.192 
     19.815       3.895 
      2.362       0.358 
 
 Lib_L_27F-038  136.0 
     51.341      18.874 
     21.205       4.135 
      2.380       0.363 
 
 Lib_L_27F-038  150.0 
     52.667      19.667 
     22.500       4.500 
      2.399       0.371 
 
 Lib_L_27F-039  10.0 
     12.977       7.335 
      5.945       1.805 
      2.250       0.555 
 
 Lib_L_27F-039  24.0 
     30.785      14.411 
     11.350       4.280 
      2.852       0.820 
 
 Lib_L_27F-039  38.0 
     46.419      24.784 
     15.505       6.315 
      3.065       0.936 
 
 Lib_L_27F-039  52.0 
     59.200      31.360 
     19.250       7.870 
      3.221       0.995 
 
 Lib_L_27F-039  66.0 
     69.105      32.940 
     22.575       9.175 
      3.295       1.040 
 
 Lib_L_27F-039  80.0 
     85.818      42.258 
     26.130      10.670 
      3.390       1.074 
 
 Lib_L_27F-039  94.0 
    104.033      50.297 
     29.270      12.410 
      3.438       1.123 
 
 Lib_L_27F-039  108.0 
    104.265      47.825 
     32.455      13.365 
      3.508       1.128 
 
 Lib_L_27F-039  122.0 
    109.103      50.046 
     35.150      14.580 
      3.544       1.144 
 
 Lib_L_27F-039  136.0 
    115.566      55.131 
     38.205      15.715 
      3.588       1.159 
 
 Lib_L_27F-039  150.0 
    119.500      61.500 
     41.000      17.000 
      3.622       1.176 
 
 Lib_L_27F-040  10.0 
      8.463       1.374 
      5.070       0.760 
      1.962       0.336 
 
 Lib_L_27F-040  24.0 
     19.138       3.543 
      8.610       1.570 
      2.362       0.483 
 
 Lib_L_27F-040  38.0 
     29.057       2.939 
     11.315       1.665 
      2.503       0.461 
 
 Lib_L_27F-040  52.0 
     37.229       1.849 
     14.160       2.080 
      2.618       0.501 
 
 Lib_L_27F-040  66.0 
     43.936       0.652 
     16.540       2.340 
      2.684       0.533 
 
 Lib_L_27F-040  80.0 
     48.919       0.825 
     18.875       2.355 
      2.740       0.521 
 
 Lib_L_27F-040  94.0 
     54.503       1.290 
     21.080       2.670 
      2.785       0.539 
 
 Lib_L_27F-040  108.0 
     58.644       4.603 
     22.770       2.560 
      2.797       0.535 
 
 Lib_L_27F-040  122.0 
     59.608       7.054 
     24.900       2.420 
      2.846       0.530 
 
 Lib_L_27F-040  136.0 
     66.539      12.561 
     26.745       2.525 
      2.867       0.541 
 
 Lib_L_27F-040  150.0 
     67.929      15.071 
     28.500       2.500 
      2.894       0.537 
 
 Lib_L_27F-041  10.0 
      7.819       4.494 
      4.110       1.530 
      1.432       0.661 
 
 Lib_L_27F-041  24.0 
     21.216      13.753 
      7.800       3.130 
      1.865       0.783 
 
 Lib_L_27F-041  38.0 
     32.046      21.530 
     10.570       4.330 
      1.992       0.833 
 
 Lib_L_27F-041  52.0 
     39.920      27.773 
     13.025       5.845 
      2.079       0.904 
 
 Lib_L_27F-041  66.0 
     49.806      34.503 
     15.425       7.105 
      2.152       0.940 
 
 Lib_L_27F-041  80.0 
     56.779      40.958 
     17.695       8.625 
      2.201       0.986 
 
 Lib_L_27F-041  94.0 
     55.937      38.502 
     19.720       9.680 
      2.242       0.994 
 
 Lib_L_27F-041  108.0 
     62.017      42.717 
     21.780      10.890 
      2.267       1.014 
 
 Lib_L_27F-041  122.0 
     65.166      45.600 
     23.845      12.205 
      2.309       1.035 
 
 Lib_L_27F-041  136.0 
     66.752      47.337 
     25.740      13.460 
      2.334       1.057 
 
 Lib_L_27F-041  150.0 
     69.750      49.750 
     27.500      14.500 
      2.354       1.064 
 
 Lib_L_27F-042  10.0 
      7.920       2.610 
      4.495       1.265 
      1.625       0.597 
 
 Lib_L_27F-042  24.0 
     18.982       7.444 
      8.345       2.635 
      2.078       0.810 
 
 Lib_L_27F-042  38.0 
     25.798      10.149 
     11.040       3.530 
      2.194       0.861 
 
 Lib_L_27F-042  52.0 
     29.637      11.198 
     13.555       4.075 
      2.287       0.840 
 
 Lib_L_27F-042  66.0 
     37.970      15.016 
     15.940       4.970 
      2.353       0.889 
 
 Lib_L_27F-042  80.0 
     39.438      14.371 
     18.135       5.675 
      2.429       0.905 
 
 Lib_L_27F-042  94.0 
     43.250      15.869 
     19.935       6.325 
      2.462       0.921 
 
 Lib_L_27F-042  108.0 
     47.241      15.238 
     21.750       6.940 
      2.503       0.930 
 
 Lib_L_27F-042  122.0 
     50.096      15.698 
     23.440       7.520 
      2.527       0.949 
 
 Lib_L_27F-042  136.0 
     51.327      14.133 
     25.045       7.975 
      2.556       0.953 
 
 Lib_L_27F-042  150.0 
     57.750      12.250 
     26.500       8.500 
      2.569       0.961 
 
 Lib_L_27F-043  10.0 
      7.140         nan 
      3.910         nan 
      1.344         nan 
 
 Lib_L_27F-043  24.0 
     16.217         nan 
      7.080         nan 
      1.641         nan 
 
 Lib_L_27F-043  38.0 
     17.871         nan 
      9.340         nan 
      1.768         nan 
 
 Lib_L_27F-043  52.0 
     24.664         nan 
     11.610         nan 
      1.825         nan 
 
 Lib_L_27F-043  66.0 
     27.573         nan 
     13.730         nan 
      1.903         nan 
 
 Lib_L_27F-043  80.0 
     33.177         nan 
     15.410         nan 
      1.951         nan 
 
 Lib_L_27F-043  94.0 
     42.991         nan 
     17.310         nan 
      2.025         nan 
 
 Lib_L_27F-043  108.0 
     45.825         nan 
     18.560         nan 
      2.015         nan 
 
 Lib_L_27F-043  122.0 
     54.624         nan 
     20.050         nan 
      2.041         nan 
 
 Lib_L_27F-043  136.0 
     58.700         nan 
     21.960         nan 
      2.081         nan 
 
 Lib_L_27F-043  150.0 
     58.000         nan 
     23.000         nan 
      2.084         nan 
 
 Lib_L_27F-044  10.0 
     13.291       4.907 
      6.155       1.315 
      2.302       0.423 
 
 Lib_L_27F-044  24.0 
     32.330      13.216 
     11.850       3.320 
      2.940       0.645 
 
 Lib_L_27F-044  38.0 
     46.833      15.499 
     16.225       4.585 
      3.172       0.694 
 
 Lib_L_27F-044  52.0 
     52.804      19.431 
     20.200       5.850 
      3.331       0.744 
 
 Lib_L_27F-044  66.0 
     58.577      22.858 
     24.030       7.210 
      3.465       0.772 
 
 Lib_L_27F-044  80.0 
     61.599      19.937 
     27.050       7.950 
      3.522       0.778 
 
 Lib_L_27F-044  94.0 
     62.922      17.328 
     29.925       8.645 
      3.582       0.797 
 
 Lib_L_27F-044  108.0 
     66.094      18.829 
     32.900       9.990 
      3.642       0.835 
 
 Lib_L_27F-044  122.0 
     65.938      20.092 
     35.370      10.530 
      3.682       0.832 
 
 Lib_L_27F-044  136.0 
     67.486      20.153 
     37.795      11.475 
      3.732       0.854 
 
 Lib_L_27F-044  150.0 
     68.125      20.125 
     40.000      12.000 
      3.759       0.858 
 
 Lib_L_27F-045  10.0 
     13.069       5.354 
      5.900       1.730 
      2.121       0.657 
 
 Lib_L_27F-045  24.0 
     25.611       7.846 
     10.700       3.130 
      2.594       0.849 
 
 Lib_L_27F-045  38.0 
     32.346       8.727 
     14.580       4.060 
      2.830       0.913 
 
 Lib_L_27F-045  52.0 
     42.315       9.054 
     18.325       4.645 
      3.012       0.918 
 
 Lib_L_27F-045  66.0 
     50.430      10.003 
     21.175       5.435 
      3.070       0.953 
 
 Lib_L_27F-045  80.0 
     51.181       9.774 
     24.030       5.750 
      3.165       0.959 
 
 Lib_L_27F-045  94.0 
     54.300      11.764 
     26.490       6.200 
      3.213       0.971 
 
 Lib_L_27F-045  108.0 
     57.850      13.186 
     28.440       6.760 
      3.244       0.985 
 
 Lib_L_27F-045  122.0 
     59.182      13.722 
     31.005       7.335 
      3.299       0.998 
 
 Lib_L_27F-045  136.0 
     61.469      16.909 
     33.020       7.760 
      3.323       1.011 
 
 Lib_L_27F-045  150.0 
     60.821      16.679 
     35.000       8.000 
      3.357       1.009 
 
 Lib_L_27F-046  10.0 
     11.106       4.899 
      5.785       1.155 
      2.244       0.319 
 
 Lib_L_27F-046  24.0 
     20.456       7.684 
      9.660       2.270 
      2.687       0.379 
 
 Lib_L_27F-046  38.0 
     34.123      12.026 
     13.230       3.480 
      2.929       0.472 
 
 Lib_L_27F-046  52.0 
     43.770      15.968 
     16.335       4.455 
      3.069       0.505 
 
 Lib_L_27F-046  66.0 
     48.487      14.630 
     18.515       5.525 
      3.106       0.561 
 
 Lib_L_27F-046  80.0 
     54.429      15.597 
     21.375       6.075 
      3.198       0.554 
 
 Lib_L_27F-046  94.0 
     55.592      16.937 
     23.650       6.630 
      3.232       0.554 
 
 Lib_L_27F-046  108.0 
     57.084      15.171 
     25.945       7.335 
      3.281       0.573 
 
 Lib_L_27F-046  122.0 
     57.614      16.825 
     28.085       7.855 
      3.324       0.581 
 
 Lib_L_27F-046  136.0 
     57.232      17.531 
     30.050       8.450 
      3.344       0.593 
 
 Lib_L_27F-046  150.0 
     56.250      18.083 
     32.000       9.000 
      3.373       0.600 
 
 Lib_L_27F-047  10.0 
     18.733         nan 
      7.400         nan 
      2.689         nan 
 
 Lib_L_27F-047  24.0 
     32.097         nan 
     13.530         nan 
      3.320         nan 
 
 Lib_L_27F-047  38.0 
     45.808         nan 
     19.010         nan 
      3.668         nan 
 
 Lib_L_27F-047  52.0 
     54.941         nan 
     23.180         nan 
      3.840         nan 
 
 Lib_L_27F-047  66.0 
     65.028         nan 
     27.180         nan 
      3.968         nan 
 
 Lib_L_27F-047  80.0 
     82.373         nan 
     30.320         nan 
      4.039         nan 
 
 Lib_L_27F-047  94.0 
     90.534         nan 
     33.850         nan 
      4.112         nan 
 
 Lib_L_27F-047  108.0 
    119.215         nan 
     37.260         nan 
      4.190         nan 
 
 Lib_L_27F-047  122.0 
    143.313         nan 
     40.390         nan 
      4.239         nan 
 
 Lib_L_27F-047  136.0 
    161.322         nan 
     43.040         nan 
      4.270         nan 
 
 Lib_L_27F-047  150.0 
    201.000         nan 
     46.000         nan 
      4.310         nan 
 
 Lib_L_27F-049  10.0 
     16.499       3.921 
      6.635       1.065 
      2.407       0.375 
 
 Lib_L_27F-049  24.0 
     33.668       6.516 
     12.460       2.220 
      3.021       0.514 
 
 Lib_L_27F-049  38.0 
     50.617       1.188 
     17.565       2.175 
      3.325       0.468 
 
 Lib_L_27F-049  52.0 
     63.036       9.136 
     22.065       3.095 
      3.515       0.522 
 
 Lib_L_27F-049  66.0 
     67.531       6.772 
     26.065       3.465 
      3.643       0.538 
 
 Lib_L_27F-049  80.0 
     73.036      10.861 
     29.455       3.935 
      3.695       0.570 
 
 Lib_L_27F-049  94.0 
     80.031      13.761 
     33.155       4.455 
      3.782       0.582 
 
 Lib_L_27F-049  108.0 
     82.344      18.770 
     36.440       5.090 
      3.861       0.588 
 
 Lib_L_27F-049  122.0 
     89.215      21.232 
     39.370       5.340 
      3.898       0.592 
 
 Lib_L_27F-049  136.0 
     93.868      25.325 
     42.320       5.650 
      3.954       0.592 
 
 Lib_L_27F-049  150.0 
     99.964      26.464 
     45.000       6.000 
      3.984       0.592 
 
 Lib_L_27F-050  10.0 
     13.550       2.378 
      6.185       0.705 
      2.285       0.245 
 
 Lib_L_27F-050  24.0 
     29.226       3.505 
     11.360       1.200 
      2.765       0.275 
 
 Lib_L_27F-050  38.0 
     35.989       3.736 
     15.685       2.055 
      3.025       0.355 
 
 Lib_L_27F-050  52.0 
     43.565       5.395 
     19.260       2.560 
      3.177       0.361 
 
 Lib_L_27F-050  66.0 
     47.282       3.517 
     22.405       2.775 
      3.280       0.340 
 
 Lib_L_27F-050  80.0 
     53.752       2.735 
     25.715       3.165 
      3.397       0.363 
 
 Lib_L_27F-050  94.0 
     58.791       2.518 
     28.205       3.305 
      3.415       0.377 
 
 Lib_L_27F-050  108.0 
     64.660       0.355 
     30.475       3.305 
      3.481       0.367 
 
 Lib_L_27F-050  122.0 
     73.733       1.573 
     33.025       3.755 
      3.523       0.383 
 
 Lib_L_27F-050  136.0 
     81.183       6.208 
     34.865       3.905 
      3.541       0.393 
 
 Lib_L_27F-050  150.0 
     93.083       9.917 
     37.000       4.000 
      3.572       0.394 
 
 Lib_L_27F-051  10.0 
      7.094       3.119 
      4.305       1.125 
      1.610       0.443 
 
 Lib_L_27F-051  24.0 
     17.040       9.805 
      7.505       2.675 
      2.009       0.581 
 
 Lib_L_27F-051  38.0 
     24.601      13.152 
     10.270       3.900 
      2.141       0.662 
 
 Lib_L_27F-051  52.0 
     33.561      16.141 
     12.700       4.910 
      2.256       0.709 
 
 Lib_L_27F-051  66.0 
     39.116      16.578 
     14.850       5.930 
      2.324       0.732 
 
 Lib_L_27F-051  80.0 
     40.899      13.354 
     16.450       6.300 
      2.342       0.733 
 
 Lib_L_27F-051  94.0 
     48.540      14.735 
     18.560       7.360 
      2.385       0.755 
 
 Lib_L_27F-051  108.0 
     55.290      13.240 
     20.490       7.820 
      2.418       0.755 
 
 Lib_L_27F-051  122.0 
     56.032      10.692 
     22.160       8.410 
      2.452       0.767 
 
 Lib_L_27F-051  136.0 
     58.151      10.051 
     23.900       9.120 
      2.474       0.791 
 
 Lib_L_27F-051  150.0 
     58.500       9.500 
     25.500       9.500 
      2.495       0.791 
 
 Lib_L_27F-052  10.0 
     10.095         nan 
      5.200         nan 
      1.929         nan 
 
 Lib_L_27F-052  24.0 
     18.405         nan 
      9.010         nan 
      2.294         nan 
 
 Lib_L_27F-052  38.0 
     24.971         nan 
     12.010         nan 
      2.467         nan 
 
 Lib_L_27F-052  52.0 
     31.391         nan 
     14.860         nan 
      2.640         nan 
 
 Lib_L_27F-052  66.0 
     31.737         nan 
     16.780         nan 
      2.701         nan 
 
 Lib_L_27F-052  80.0 
     38.183         nan 
     19.090         nan 
      2.775         nan 
 
 Lib_L_27F-052  94.0 
     43.259         nan 
     20.670         nan 
      2.806         nan 
 
 Lib_L_27F-052  108.0 
     48.751         nan 
     22.590         nan 
      2.846         nan 
 
 Lib_L_27F-052  122.0 
     47.196         nan 
     24.010         nan 
      2.865         nan 
 
 Lib_L_27F-052  136.0 
     49.574         nan 
     25.730         nan 
      2.897         nan 
 
 Lib_L_27F-052  150.0 
     48.000         nan 
     27.000         nan 
      2.915         nan 
 
  Innoculation  Seqs/Sample 
 chao1 Ave.  chao1 Err. 
 observed_species Ave.  observed_species Err. 
 shannon Ave.  shannon Err. 
 
 
 After  10.0 
      9.207       4.676 
      4.915       1.374 
      1.840       0.548 
 
 After  24.0 
     20.573       9.577 
      8.784       3.001 
      2.267       0.713 
 
 After  38.0 
     29.696      13.596 
     11.854       4.155 
      2.436       0.766 
 
 After  52.0 
     37.094      16.355 
     14.668       5.271 
      2.557       0.815 
 
 After  66.0 
     44.321      19.820 
     17.159       6.150 
      2.635       0.840 
 
 After  80.0 
     50.621      24.453 
     19.489       7.029 
      2.692       0.860 
 
 After  94.0 
     55.726      26.868 
     21.616       7.914 
      2.733       0.882 
 
 After  108.0 
     61.084      28.886 
     23.624       8.525 
      2.767       0.889 
 
 After  122.0 
     64.624      31.279 
     25.643       9.342 
      2.804       0.904 
 
 After  136.0 
     66.027      29.843 
     27.502       9.940 
      2.830       0.910 
 
 After  150.0 
     67.648      31.514 
     29.250      10.601 
      2.853       0.919 
 
 Before  10.0 
     10.395       5.545 
      5.289       1.711 
      1.964       0.681 
 
 Before  24.0 
     21.831      12.600 
      9.540       3.604 
      2.453       0.876 
 
 Before  38.0 
     31.280      16.798 
     12.771       5.012 
      2.634       0.944 
 
 Before  52.0 
     38.743      20.695 
     15.625       6.267 
      2.749       0.997 
 
 Before  66.0 
     45.118      22.424 
     18.320       7.493 
      2.840       1.036 
 
 Before  80.0 
     51.389      26.631 
     20.571       8.430 
      2.894       1.049 
 
 Before  94.0 
     55.634      28.471 
     22.733       9.426 
      2.937       1.070 
 
 Before  108.0 
     59.180      30.871 
     24.840      10.339 
      2.978       1.087 
 
 Before  122.0 
     61.759      33.505 
     26.850      11.211 
      3.015       1.101 
 
 Before  136.0 
     63.497      34.414 
     28.715      12.039 
      3.043       1.113 
 
 Before  150.0 
     63.368      33.819 
     30.444      12.810 
      3.065       1.123 
 
 During  10.0 
     11.167       6.011 
      5.400       1.741 
      1.991       0.672 
 
 During  24.0 
     23.878      13.606 
      9.743       3.607 
      2.459       0.859 
 
 During  38.0 
     35.130      19.451 
     13.457       5.181 
      2.686       0.943 
 
 During  52.0 
     45.505      25.704 
     16.664       6.535 
      2.813       0.984 
 
 During  66.0 
     52.560      27.441 
     19.537       7.851 
      2.896       1.028 
 
 During  80.0 
     58.870      29.840 
     22.241       8.946 
      2.966       1.048 
 
 During  94.0 
     64.418      33.044 
     24.681      10.064 
      3.012       1.068 
 
 During  108.0 
     68.854      34.830 
     27.070      11.169 
      3.060       1.092 
 
 During  122.0 
     74.277      39.717 
     29.328      12.147 
      3.093       1.106 
 
 During  136.0 
     77.625      41.620 
     31.468      13.034 
      3.124       1.119 
 
 During  150.0 
     81.574      46.037 
     33.551      13.931 
      3.152       1.129 
 
  BarcodeSequence  Seqs/Sample 
 chao1 Ave.  chao1 Err. 
 observed_species Ave.  observed_species Err. 
 shannon Ave.  shannon Err. 
 
 
 ACATACGCGTAG  10.0 
      5.438       1.698 
      3.370       0.660 
      1.111       0.296 
 
 ACATACGCGTAG  24.0 
     11.168       2.986 
      5.990       1.170 
      1.383       0.308 
 
 ACATACGCGTAG  38.0 
     14.776       2.100 
      8.160       1.600 
      1.514       0.364 
 
 ACATACGCGTAG  52.0 
     18.086       3.092 
     10.155       2.255 
      1.628       0.418 
 
 ACATACGCGTAG  66.0 
     20.213       2.159 
     11.390       2.460 
      1.648       0.435 
 
 ACATACGCGTAG  80.0 
     23.200       1.711 
     12.680       2.580 
      1.673       0.439 
 
 ACATACGCGTAG  94.0 
     25.342       2.310 
     13.875       2.895 
      1.715       0.462 
 
 ACATACGCGTAG  108.0 
     30.792       2.002 
     14.970       2.930 
      1.735       0.452 
 
 ACATACGCGTAG  122.0 
     33.882       2.897 
     15.925       2.915 
      1.744       0.454 
 
 ACATACGCGTAG  136.0 
     38.670       3.628 
     17.010       2.930 
      1.757       0.458 
 
 ACATACGCGTAG  150.0 
     43.000       5.500 
     18.000       3.000 
      1.769       0.457 
 
 ACGACTACAGAG  10.0 
      3.552       2.182 
      2.565       1.215 
      0.788       0.624 
 
 ACGACTACAGAG  24.0 
      6.214       4.504 
      3.900       2.270 
      0.922       0.760 
 
 ACGACTACAGAG  38.0 
     10.636       8.106 
      5.275       3.115 
      1.029       0.816 
 
 ACGACTACAGAG  52.0 
     12.002       8.597 
      6.160       3.530 
      1.054       0.817 
 
 ACGACTACAGAG  66.0 
     15.259      11.099 
      7.315       4.275 
      1.091       0.840 
 
 ACGACTACAGAG  80.0 
     18.866      13.421 
      8.480       4.940 
      1.129       0.865 
 
 ACGACTACAGAG  94.0 
     18.905      13.670 
      9.055       5.395 
      1.128       0.874 
 
 ACGACTACAGAG  108.0 
     21.676      15.401 
      9.960       5.850 
      1.143       0.880 
 
 ACGACTACAGAG  122.0 
     24.105      17.250 
     10.820       6.320 
      1.155       0.883 
 
 ACGACTACAGAG  136.0 
     25.255      18.709 
     11.855       7.135 
      1.182       0.909 
 
 ACGACTACAGAG  150.0 
     26.250      19.750 
     12.500       7.500 
      1.186       0.911 
 
 ACGAGTGCGTAG  10.0 
     12.495       3.037 
      6.355       0.805 
      2.427       0.232 
 
 ACGAGTGCGTAG  24.0 
     24.280       6.148 
     11.100       1.900 
      2.996       0.331 
 
 ACGAGTGCGTAG  38.0 
     35.351       9.119 
     14.655       2.635 
      3.202       0.367 
 
 ACGAGTGCGTAG  52.0 
     44.425       7.777 
     17.960       3.370 
      3.356       0.409 
 
 ACGAGTGCGTAG  66.0 
     51.272      13.335 
     20.805       4.105 
      3.458       0.426 
 
 ACGAGTGCGTAG  80.0 
     62.085      22.053 
     22.910       4.590 
      3.493       0.424 
 
 ACGAGTGCGTAG  94.0 
     68.445      18.128 
     25.520       5.220 
      3.542       0.450 
 
 ACGAGTGCGTAG  108.0 
     80.245      30.150 
     28.245       5.745 
      3.613       0.447 
 
 ACGAGTGCGTAG  122.0 
     77.821      31.579 
     30.150       6.220 
      3.638       0.457 
 
 ACGAGTGCGTAG  136.0 
     86.839      34.136 
     32.575       6.875 
      3.677       0.476 
 
 ACGAGTGCGTAG  150.0 
     84.300      33.300 
     34.500       7.500 
      3.697       0.483 
 
 ACGCGAGTATAG  10.0 
      6.194       1.156 
      3.700       0.400 
      1.271       0.172 
 
 ACGCGAGTATAG  24.0 
     12.026       2.586 
      6.365       0.825 
      1.532       0.222 
 
 ACGCGAGTATAG  38.0 
     16.382       1.734 
      8.835       1.045 
      1.706       0.230 
 
 ACGCGAGTATAG  52.0 
     20.673       4.441 
     10.720       1.680 
      1.782       0.309 
 
 ACGCGAGTATAG  66.0 
     24.249       5.071 
     12.345       2.115 
      1.839       0.325 
 
 ACGCGAGTATAG  80.0 
     28.374       5.197 
     13.925       2.325 
      1.880       0.330 
 
 ACGCGAGTATAG  94.0 
     33.066       4.007 
     15.315       2.175 
      1.918       0.287 
 
 ACGCGAGTATAG  108.0 
     36.654       2.133 
     16.425       2.435 
      1.923       0.294 
 
 ACGCGAGTATAG  122.0 
     39.754       0.079 
     17.590       2.790 
      1.942       0.308 
 
 ACGCGAGTATAG  136.0 
     45.260       7.239 
     18.880       2.780 
      1.967       0.310 
 
 ACGCGAGTATAG  150.0 
     55.300      16.700 
     20.000       3.000 
      1.979       0.318 
 
 ACGCGATCGAAG  10.0 
     16.499       3.921 
      6.635       1.065 
      2.407       0.375 
 
 ACGCGATCGAAG  24.0 
     33.668       6.516 
     12.460       2.220 
      3.021       0.514 
 
 ACGCGATCGAAG  38.0 
     50.617       1.188 
     17.565       2.175 
      3.325       0.468 
 
 ACGCGATCGAAG  52.0 
     63.036       9.136 
     22.065       3.095 
      3.515       0.522 
 
 ACGCGATCGAAG  66.0 
     67.531       6.772 
     26.065       3.465 
      3.643       0.538 
 
 ACGCGATCGAAG  80.0 
     73.036      10.861 
     29.455       3.935 
      3.695       0.570 
 
 ACGCGATCGAAG  94.0 
     80.031      13.761 
     33.155       4.455 
      3.782       0.582 
 
 ACGCGATCGAAG  108.0 
     82.344      18.770 
     36.440       5.090 
      3.861       0.588 
 
 ACGCGATCGAAG  122.0 
     89.215      21.232 
     39.370       5.340 
      3.898       0.592 
 
 ACGCGATCGAAG  136.0 
     93.868      25.325 
     42.320       5.650 
      3.954       0.592 
 
 ACGCGATCGAAG  150.0 
     99.964      26.464 
     45.000       6.000 
      3.984       0.592 
 
 ACGCTCGACAAG  10.0 
      9.555       5.910 
      4.495       1.825 
      1.556       0.778 
 
 ACGCTCGACAAG  24.0 
     19.752       9.721 
      8.370       3.540 
      1.955       0.961 
 
 ACGCTCGACAAG  38.0 
     25.711      10.071 
     11.440       4.500 
      2.115       0.994 
 
 ACGCTCGACAAG  52.0 
     28.549       7.834 
     14.145       5.185 
      2.251       1.042 
 
 ACGCTCGACAAG  66.0 
     32.177      10.147 
     16.325       6.085 
      2.314       1.105 
 
 ACGCTCGACAAG  80.0 
     35.698      13.846 
     18.330       6.440 
      2.370       1.091 
 
 ACGCTCGACAAG  94.0 
     38.012      15.855 
     20.100       6.850 
      2.426       1.099 
 
 ACGCTCGACAAG  108.0 
     42.961      21.122 
     21.640       7.270 
      2.444       1.099 
 
 ACGCTCGACAAG  122.0 
     47.745      25.712 
     23.305       8.025 
      2.473       1.127 
 
 ACGCTCGACAAG  136.0 
     49.270      27.195 
     24.610       8.390 
      2.497       1.143 
 
 ACGCTCGACAAG  150.0 
     52.083      30.417 
     26.000       9.000 
      2.520       1.146 
 
 ACTACTATGTAG  10.0 
      5.753       1.333 
      3.760       0.690 
      1.371       0.384 
 
 ACTACTATGTAG  24.0 
     13.595       4.083 
      6.535       1.215 
      1.682       0.429 
 
 ACTACTATGTAG  38.0 
     22.570       9.303 
      9.400       1.980 
      1.910       0.499 
 
 ACTACTATGTAG  52.0 
     29.946      12.190 
     11.400       2.400 
      1.957       0.475 
 
 ACTACTATGTAG  66.0 
     36.219      14.371 
     13.510       3.110 
      2.027       0.506 
 
 ACTACTATGTAG  80.0 
     38.257      14.505 
     15.480       3.700 
      2.105       0.528 
 
 ACTACTATGTAG  94.0 
     40.663      14.525 
     17.240       4.220 
      2.131       0.533 
 
 ACTACTATGTAG  108.0 
     42.081      15.067 
     18.690       4.770 
      2.147       0.548 
 
 ACTACTATGTAG  122.0 
     43.565      13.403 
     20.065       4.885 
      2.153       0.522 
 
 ACTACTATGTAG  136.0 
     42.117      12.391 
     21.515       5.545 
      2.170       0.542 
 
 ACTACTATGTAG  150.0 
     42.714      10.714 
     23.000       6.000 
      2.198       0.550 
 
 ACTAGCAGTAAG  10.0 
     13.550       2.378 
      6.185       0.705 
      2.285       0.245 
 
 ACTAGCAGTAAG  24.0 
     29.226       3.505 
     11.360       1.200 
      2.765       0.275 
 
 ACTAGCAGTAAG  38.0 
     35.989       3.736 
     15.685       2.055 
      3.025       0.355 
 
 ACTAGCAGTAAG  52.0 
     43.565       5.395 
     19.260       2.560 
      3.177       0.361 
 
 ACTAGCAGTAAG  66.0 
     47.282       3.517 
     22.405       2.775 
      3.280       0.340 
 
 ACTAGCAGTAAG  80.0 
     53.752       2.735 
     25.715       3.165 
      3.397       0.363 
 
 ACTAGCAGTAAG  94.0 
     58.791       2.518 
     28.205       3.305 
      3.415       0.377 
 
 ACTAGCAGTAAG  108.0 
     64.660       0.355 
     30.475       3.305 
      3.481       0.367 
 
 ACTAGCAGTAAG  122.0 
     73.733       1.573 
     33.025       3.755 
      3.523       0.383 
 
 ACTAGCAGTAAG  136.0 
     81.183       6.208 
     34.865       3.905 
      3.541       0.393 
 
 ACTAGCAGTAAG  150.0 
     93.083       9.917 
     37.000       4.000 
      3.572       0.394 
 
 ACTGTACAGTAG  10.0 
      8.501       0.448 
      4.985       0.115 
      1.909       0.100 
 
 ACTGTACAGTAG  24.0 
     16.207       0.072 
      8.620       0.130 
      2.366       0.066 
 
 ACTGTACAGTAG  38.0 
     22.214       0.098 
     11.075       0.225 
      2.465       0.066 
 
 ACTGTACAGTAG  52.0 
     29.202       3.445 
     13.660       0.150 
      2.599       0.060 
 
 ACTGTACAGTAG  66.0 
     36.419       5.793 
     15.855       0.015 
      2.666       0.069 
 
 ACTGTACAGTAG  80.0 
     38.133       4.665 
     17.880       0.060 
      2.730       0.067 
 
 ACTGTACAGTAG  94.0 
     38.897       4.028 
     19.295       0.095 
      2.753       0.065 
 
 ACTGTACAGTAG  108.0 
     42.920       5.111 
     21.090       0.120 
      2.800       0.068 
 
 ACTGTACAGTAG  122.0 
     45.406       6.046 
     22.750       0.290 
      2.834       0.065 
 
 ACTGTACAGTAG  136.0 
     48.027       7.136 
     24.130       0.320 
      2.852       0.063 
 
 ACTGTACAGTAG  150.0 
     48.300       7.700 
     25.500       0.500 
      2.872       0.068 
 
 AGACGCACTCAG  10.0 
     13.852       0.621 
      6.485       0.295 
      2.415       0.148 
 
 AGACGCACTCAG  24.0 
     31.606       2.256 
     12.280       0.460 
      3.045       0.229 
 
 AGACGCACTCAG  38.0 
     44.309       4.321 
     17.060       0.340 
      3.344       0.161 
 
 AGACGCACTCAG  52.0 
     62.543       4.397 
     20.940       0.410 
      3.473       0.179 
 
 AGACGCACTCAG  66.0 
     73.354       5.814 
     24.875       0.595 
      3.575       0.204 
 
 AGACGCACTCAG  80.0 
     82.456       5.539 
     28.360       1.020 
      3.678       0.171 
 
 AGACGCACTCAG  94.0 
     85.562       9.794 
     31.590       1.060 
      3.729       0.187 
 
 AGACGCACTCAG  108.0 
     94.987      23.163 
     34.685       1.785 
      3.789       0.165 
 
 AGACGCACTCAG  122.0 
     97.999      22.715 
     37.870       1.820 
      3.832       0.171 
 
 AGACGCACTCAG  136.0 
     98.283      26.005 
     40.485       2.015 
      3.863       0.177 
 
 AGACGCACTCAG  150.0 
    102.500      31.500 
     43.500       2.500 
      3.909       0.168 
 
 AGACTATACTAG  10.0 
      9.178       0.011 
      5.110       0.250 
      1.958       0.161 
 
 AGACTATACTAG  24.0 
     19.156       2.159 
      8.895       0.305 
      2.372       0.176 
 
 AGACTATACTAG  38.0 
     28.527       5.457 
     12.225       0.165 
      2.605       0.130 
 
 AGACTATACTAG  52.0 
     36.463       8.990 
     15.145       0.535 
      2.720       0.170 
 
 AGACTATACTAG  66.0 
     43.090       8.959 
     17.615       0.825 
      2.811       0.158 
 
 AGACTATACTAG  80.0 
     45.056      12.331 
     19.615       1.275 
      2.849       0.183 
 
 AGACTATACTAG  94.0 
     48.802      13.094 
     21.985       1.875 
      2.914       0.198 
 
 AGACTATACTAG  108.0 
     50.367      11.185 
     23.835       2.065 
      2.938       0.188 
 
 AGACTATACTAG  122.0 
     52.592       8.221 
     25.755       2.515 
      2.966       0.204 
 
 AGACTATACTAG  136.0 
     51.097       5.961 
     27.295       2.655 
      2.986       0.200 
 
 AGACTATACTAG  150.0 
     52.250       3.500 
     29.000       3.000 
      3.011       0.206 
 
 AGCACTGTAGAG  10.0 
      7.778       5.988 
      4.170       2.560 
      1.416       1.123 
 
 AGCACTGTAGAG  24.0 
     13.561      10.301 
      7.170       4.620 
      1.771       1.357 
 
 AGCACTGTAGAG  38.0 
     20.921      16.221 
      9.655       6.375 
      1.927       1.479 
 
 AGCACTGTAGAG  52.0 
     28.526      20.516 
     11.960       7.440 
      2.036       1.501 
 
 AGCACTGTAGAG  66.0 
     35.461      25.231 
     13.725       8.545 
      2.070       1.539 
 
 AGCACTGTAGAG  80.0 
     37.450      26.080 
     15.165       9.485 
      2.090       1.556 
 
 AGCACTGTAGAG  94.0 
     40.667      27.107 
     17.045      10.685 
      2.145       1.608 
 
 AGCACTGTAGAG  108.0 
     43.969      27.184 
     18.535      11.555 
      2.165       1.612 
 
 AGCACTGTAGAG  122.0 
     47.169      26.544 
     20.115      12.525 
      2.193       1.637 
 
 AGCACTGTAGAG  136.0 
     51.210      25.520 
     21.575      13.215 
      2.212       1.638 
 
 AGCACTGTAGAG  150.0 
     54.583      24.583 
     23.000      14.000 
      2.229       1.651 
 
 AGCGTCGTCTAG  10.0 
     11.589       5.136 
      5.465       1.115 
      2.063       0.330 
 
 AGCGTCGTCTAG  24.0 
     33.209      17.786 
     10.505       3.035 
      2.589       0.512 
 
 AGCGTCGTCTAG  38.0 
     45.556      24.088 
     14.630       4.740 
      2.793       0.593 
 
 AGCGTCGTCTAG  52.0 
     61.359      35.834 
     18.505       6.465 
      2.937       0.664 
 
 AGCGTCGTCTAG  66.0 
     64.828      35.957 
     21.735       7.815 
      3.029       0.692 
 
 AGCGTCGTCTAG  80.0 
     73.535      45.538 
     25.205       9.795 
      3.116       0.745 
 
 AGCGTCGTCTAG  94.0 
     75.405      47.170 
     27.705      10.865 
      3.143       0.743 
 
 AGCGTCGTCTAG  108.0 
     74.205      41.152 
     30.530      12.080 
      3.192       0.754 
 
 AGCGTCGTCTAG  122.0 
     77.181      43.356 
     33.405      13.835 
      3.251       0.804 
 
 AGCGTCGTCTAG  136.0 
     77.449      46.441 
     36.095      15.205 
      3.297       0.807 
 
 AGCGTCGTCTAG  150.0 
     76.765      45.598 
     38.500      16.500 
      3.325       0.818 
 
 AGCTCACGTAAG  10.0 
      7.094       3.119 
      4.305       1.125 
      1.610       0.443 
 
 AGCTCACGTAAG  24.0 
     17.040       9.805 
      7.505       2.675 
      2.009       0.581 
 
 AGCTCACGTAAG  38.0 
     24.601      13.152 
     10.270       3.900 
      2.141       0.662 
 
 AGCTCACGTAAG  52.0 
     33.561      16.141 
     12.700       4.910 
      2.256       0.709 
 
 AGCTCACGTAAG  66.0 
     39.116      16.578 
     14.850       5.930 
      2.324       0.732 
 
 AGCTCACGTAAG  80.0 
     40.899      13.354 
     16.450       6.300 
      2.342       0.733 
 
 AGCTCACGTAAG  94.0 
     48.540      14.735 
     18.560       7.360 
      2.385       0.755 
 
 AGCTCACGTAAG  108.0 
     55.290      13.240 
     20.490       7.820 
      2.418       0.755 
 
 AGCTCACGTAAG  122.0 
     56.032      10.692 
     22.160       8.410 
      2.452       0.767 
 
 AGCTCACGTAAG  136.0 
     58.151      10.051 
     23.900       9.120 
      2.474       0.791 
 
 AGCTCACGTAAG  150.0 
     58.500       9.500 
     25.500       9.500 
      2.495       0.791 
 
 AGTACGCTATAG  10.0 
     10.048       4.628 
      5.240       1.750 
      1.917       0.703 
 
 AGTACGCTATAG  24.0 
     20.644       9.940 
      9.425       3.245 
      2.424       0.866 
 
 AGTACGCTATAG  38.0 
     36.923      20.182 
     13.030       4.880 
      2.651       0.960 
 
 AGTACGCTATAG  52.0 
     40.201      16.786 
     15.645       5.925 
      2.725       1.017 
 
 AGTACGCTATAG  66.0 
     45.519      17.566 
     18.555       7.205 
      2.831       1.066 
 
 AGTACGCTATAG  80.0 
     48.659      15.981 
     20.725       7.785 
      2.862       1.063 
 
 AGTACGCTATAG  94.0 
     49.289      16.080 
     23.215       9.015 
      2.928       1.101 
 
 AGTACGCTATAG  108.0 
     48.649      18.247 
     25.300       9.740 
      2.970       1.117 
 
 AGTACGCTATAG  122.0 
     47.737      17.712 
     27.115      10.085 
      3.003       1.104 
 
 AGTACGCTATAG  136.0 
     46.836      18.621 
     28.915      11.065 
      3.031       1.135 
 
 AGTACGCTATAG  150.0 
     46.583      18.417 
     30.500      11.500 
      3.047       1.138 
 
 AGTATACATAAG  10.0 
     10.095         nan 
      5.200         nan 
      1.929         nan 
 
 AGTATACATAAG  24.0 
     18.405         nan 
      9.010         nan 
      2.294         nan 
 
 AGTATACATAAG  38.0 
     24.971         nan 
     12.010         nan 
      2.467         nan 
 
 AGTATACATAAG  52.0 
     31.391         nan 
     14.860         nan 
      2.640         nan 
 
 AGTATACATAAG  66.0 
     31.737         nan 
     16.780         nan 
      2.701         nan 
 
 AGTATACATAAG  80.0 
     38.183         nan 
     19.090         nan 
      2.775         nan 
 
 AGTATACATAAG  94.0 
     43.259         nan 
     20.670         nan 
      2.806         nan 
 
 AGTATACATAAG  108.0 
     48.751         nan 
     22.590         nan 
      2.846         nan 
 
 AGTATACATAAG  122.0 
     47.196         nan 
     24.010         nan 
      2.865         nan 
 
 AGTATACATAAG  136.0 
     49.574         nan 
     25.730         nan 
      2.897         nan 
 
 AGTATACATAAG  150.0 
     48.000         nan 
     27.000         nan 
      2.915         nan 
 
 ATACGACGTAAG  10.0 
     10.153       2.322 
      5.610       1.010 
      2.146       0.374 
 
 ATACGACGTAAG  24.0 
     26.169       5.832 
      9.895       1.635 
      2.651       0.416 
 
 ATACGACGTAAG  38.0 
     37.459       6.772 
     13.510       2.180 
      2.868       0.457 
 
 ATACGACGTAAG  52.0 
     46.410      14.415 
     16.345       2.845 
      2.953       0.484 
 
 ATACGACGTAAG  66.0 
     55.341      16.577 
     19.260       3.440 
      3.035       0.504 
 
 ATACGACGTAAG  80.0 
     60.135      14.083 
     22.215       3.635 
      3.121       0.502 
 
 ATACGACGTAAG  94.0 
     66.838      14.995 
     24.410       4.140 
      3.138       0.510 
 
 ATACGACGTAAG  108.0 
     70.470       8.306 
     27.280       4.750 
      3.213       0.513 
 
 ATACGACGTAAG  122.0 
     69.456       3.628 
     29.515       5.255 
      3.240       0.536 
 
 ATACGACGTAAG  136.0 
     78.142       0.770 
     31.950       5.740 
      3.277       0.533 
 
 ATACGACGTAAG  150.0 
     78.750       6.250 
     34.000       6.000 
      3.301       0.538 
 
 ATAGAGTACTAG  10.0 
     10.481       3.356 
      5.535       1.195 
      2.109       0.425 
 
 ATAGAGTACTAG  24.0 
     20.024       6.135 
      9.515       2.265 
      2.574       0.513 
 
 ATAGAGTACTAG  38.0 
     33.354      11.036 
     12.825       3.035 
      2.774       0.544 
 
 ATAGAGTACTAG  52.0 
     41.720      15.108 
     15.460       4.090 
      2.868       0.604 
 
 ATAGAGTACTAG  66.0 
     52.423      14.431 
     18.220       4.640 
      2.972       0.619 
 
 ATAGAGTACTAG  80.0 
     67.780      18.313 
     20.580       5.070 
      3.018       0.612 
 
 ATAGAGTACTAG  94.0 
     81.765      27.105 
     23.085       6.125 
      3.068       0.657 
 
 ATAGAGTACTAG  108.0 
     90.929      34.559 
     25.725       6.995 
      3.122       0.692 
 
 ATAGAGTACTAG  122.0 
    107.679      44.983 
     28.015       7.325 
      3.154       0.683 
 
 ATAGAGTACTAG  136.0 
    107.388      45.714 
     30.305       7.905 
      3.180       0.691 
 
 ATAGAGTACTAG  150.0 
    103.875      45.875 
     32.500       8.500 
      3.209       0.694 
 
 ATATCGCGAGAG  10.0 
     19.078       1.969 
      7.520       0.460 
      2.740       0.129 
 
 ATATCGCGAGAG  24.0 
     34.822       7.304 
     14.010       1.300 
      3.439       0.213 
 
 ATATCGCGAGAG  38.0 
     49.158      14.641 
     19.150       2.200 
      3.752       0.257 
 
 ATATCGCGAGAG  52.0 
     63.795      14.581 
     23.975       2.455 
      3.942       0.236 
 
 ATATCGCGAGAG  66.0 
     79.329      21.069 
     28.040       3.600 
      4.075       0.290 
 
 ATATCGCGAGAG  80.0 
     81.364      19.160 
     31.790       4.280 
      4.180       0.287 
 
 ATATCGCGAGAG  94.0 
     86.063      23.151 
     35.160       5.100 
      4.235       0.314 
 
 ATATCGCGAGAG  108.0 
     87.490      18.093 
     38.490       5.330 
      4.300       0.303 
 
 ATATCGCGAGAG  122.0 
     93.829      22.778 
     41.670       6.330 
      4.355       0.320 
 
 ATATCGCGAGAG  136.0 
     92.828      21.874 
     44.535       6.835 
      4.397       0.326 
 
 ATATCGCGAGAG  150.0 
     94.812      23.188 
     47.500       7.500 
      4.438       0.336 
 
 ATCAGACACGAG  10.0 
     15.672       0.657 
      6.795       0.285 
      2.515       0.100 
 
 ATCAGACACGAG  24.0 
     31.877       0.798 
     12.475       0.065 
      3.106       0.063 
 
 ATCAGACACGAG  38.0 
     51.353       0.803 
     17.875       0.365 
      3.440       0.126 
 
 ATCAGACACGAG  52.0 
     68.829      10.135 
     21.780       0.090 
      3.577       0.078 
 
 ATCAGACACGAG  66.0 
     84.699      15.325 
     25.665       0.095 
      3.657       0.116 
 
 ATCAGACACGAG  80.0 
     98.848      18.753 
     29.470       0.140 
      3.778       0.102 
 
 ATCAGACACGAG  94.0 
    111.331      22.286 
     32.790       0.180 
      3.830       0.125 
 
 ATCAGACACGAG  108.0 
    123.505      26.798 
     36.180       0.200 
      3.882       0.132 
 
 ATCAGACACGAG  122.0 
    149.782      48.166 
     39.635       0.635 
      3.938       0.100 
 
 ATCAGACACGAG  136.0 
    152.325      46.519 
     42.780       0.800 
      3.986       0.107 
 
 ATCAGACACGAG  150.0 
    161.000      43.500 
     46.000       1.000 
      4.028       0.106 
 
 CACGCTACGTAG  10.0 
     17.783       8.417 
      7.295       1.385 
      2.687       0.356 
 
 CACGCTACGTAG  24.0 
     38.971      20.213 
     13.805       4.365 
      3.375       0.636 
 
 CACGCTACGTAG  38.0 
     51.157      17.400 
     19.040       6.080 
      3.680       0.677 
 
 CACGCTACGTAG  52.0 
     57.644      18.440 
     23.275       8.155 
      3.845       0.781 
 
 CACGCTACGTAG  66.0 
     65.556      13.250 
     27.690       9.930 
      3.981       0.845 
 
 CACGCTACGTAG  80.0 
     71.361      11.124 
     31.105      10.925 
      4.065       0.865 
 
 CACGCTACGTAG  94.0 
     73.601      11.988 
     34.525      12.195 
      4.139       0.892 
 
 CACGCTACGTAG  108.0 
     75.919      11.468 
     37.670      13.340 
      4.201       0.920 
 
 CACGCTACGTAG  122.0 
     81.277       7.864 
     40.955      13.715 
      4.269       0.917 
 
 CACGCTACGTAG  136.0 
     86.080       3.912 
     43.585      14.455 
      4.305       0.936 
 
 CACGCTACGTAG  150.0 
     85.926       2.426 
     46.000      15.000 
      4.339       0.949 
 
 CAGTAGACGTAG  10.0 
      7.608       2.030 
      4.455       0.735 
      1.655       0.317 
 
 CAGTAGACGTAG  24.0 
     16.804       3.752 
      7.745       1.545 
      2.010       0.433 
 
 CAGTAGACGTAG  38.0 
     27.511       4.806 
     10.605       1.935 
      2.188       0.435 
 
 CAGTAGACGTAG  52.0 
     35.776       6.349 
     13.050       2.510 
      2.277       0.487 
 
 CAGTAGACGTAG  66.0 
     41.032       2.028 
     15.395       2.805 
      2.336       0.511 
 
 CAGTAGACGTAG  80.0 
     51.609       3.780 
     17.740       2.690 
      2.398       0.472 
 
 CAGTAGACGTAG  94.0 
     59.809       2.921 
     19.860       2.770 
      2.433       0.475 
 
 CAGTAGACGTAG  108.0 
     61.324       8.588 
     21.710       3.120 
      2.471       0.484 
 
 CAGTAGACGTAG  122.0 
     70.341       8.955 
     23.780       3.360 
      2.499       0.492 
 
 CAGTAGACGTAG  136.0 
     75.396       5.067 
     25.645       3.385 
      2.526       0.489 
 
 CAGTAGACGTAG  150.0 
     79.250       4.250 
     27.500       3.500 
      2.548       0.492 
 
 CATAGTAGTGAG  10.0 
      5.782       0.298 
      4.215       0.395 
      1.730       0.209 
 
 CATAGTAGTGAG  24.0 
     11.706       0.352 
      6.880       0.310 
      2.091       0.219 
 
 CATAGTAGTGAG  38.0 
     15.501       0.633 
      8.375       0.085 
      2.151       0.196 
 
 CATAGTAGTGAG  52.0 
     21.151       3.182 
     10.210       0.370 
      2.234       0.244 
 
 CATAGTAGTGAG  66.0 
     26.133       3.403 
     12.110       0.210 
      2.329       0.210 
 
 CATAGTAGTGAG  80.0 
     29.066       4.657 
     13.120       0.350 
      2.338       0.208 
 
 CATAGTAGTGAG  94.0 
     32.040       3.527 
     14.415       0.485 
      2.352       0.217 
 
 CATAGTAGTGAG  108.0 
     31.360       4.345 
     15.530       0.540 
      2.380       0.207 
 
 CATAGTAGTGAG  122.0 
     33.020       1.057 
     16.835       0.775 
      2.411       0.218 
 
 CATAGTAGTGAG  136.0 
     35.071       0.624 
     17.975       1.025 
      2.420       0.226 
 
 CATAGTAGTGAG  150.0 
     33.100       0.100 
     19.000       1.000 
      2.436       0.222 
 
 CGACGTGACTAG  10.0 
     10.202       5.547 
      5.410       1.750 
      2.087       0.573 
 
 CGACGTGACTAG  24.0 
     21.574      11.904 
      9.370       3.790 
      2.532       0.814 
 
 CGACGTGACTAG  38.0 
     36.515      18.470 
     12.785       5.235 
      2.722       0.904 
 
 CGACGTGACTAG  52.0 
     50.417      21.372 
     16.040       6.360 
      2.871       0.920 
 
 CGACGTGACTAG  66.0 
     61.886      21.376 
     19.040       7.690 
      2.951       0.988 
 
 CGACGTGACTAG  80.0 
     71.171      14.996 
     21.700       8.770 
      3.009       1.012 
 
 CGACGTGACTAG  94.0 
     91.124       8.284 
     24.595       9.455 
      3.082       1.020 
 
 CGACGTGACTAG  108.0 
    101.315       3.010 
     27.280      10.630 
      3.127       1.052 
 
 CGACGTGACTAG  122.0 
    114.136      16.724 
     29.640      11.220 
      3.153       1.060 
 
 CGACGTGACTAG  136.0 
    131.497      31.893 
     32.195      11.805 
      3.196       1.067 
 
 CGACGTGACTAG  150.0 
    145.833      47.167 
     34.500      12.500 
      3.220       1.080 
 
 CGAGAGATACAG  10.0 
      7.586       1.472 
      4.905       0.495 
      1.946       0.164 
 
 CGAGAGATACAG  24.0 
     17.339       4.630 
      8.175       1.035 
      2.305       0.222 
 
 CGAGAGATACAG  38.0 
     26.944      10.699 
     10.805       1.645 
      2.447       0.249 
 
 CGAGAGATACAG  52.0 
     39.826      18.344 
     13.595       2.005 
      2.592       0.238 
 
 CGAGAGATACAG  66.0 
     44.592      17.558 
     15.430       2.390 
      2.613       0.249 
 
 CGAGAGATACAG  80.0 
     51.729      22.093 
     17.985       3.155 
      2.699       0.275 
 
 CGAGAGATACAG  94.0 
     60.571      25.541 
     19.810       3.550 
      2.730       0.271 
 
 CGAGAGATACAG  108.0 
     64.120      28.214 
     21.610       4.320 
      2.756       0.281 
 
 CGAGAGATACAG  122.0 
     61.313      22.160 
     23.465       4.905 
      2.783       0.298 
 
 CGAGAGATACAG  136.0 
     64.917      25.796 
     25.270       5.560 
      2.810       0.304 
 
 CGAGAGATACAG  150.0 
     62.850      25.350 
     27.000       6.000 
      2.836       0.303 
 
 CGTAGACTAGAG  10.0 
     12.024       5.093 
      5.650       1.450 
      2.098       0.504 
 
 CGTAGACTAGAG  24.0 
     24.716       5.753 
     10.405       2.785 
      2.606       0.600 
 
 CGTAGACTAGAG  38.0 
     36.671       4.321 
     14.315       3.755 
      2.832       0.668 
 
 CGTAGACTAGAG  52.0 
     46.357       0.559 
     17.470       4.250 
      2.938       0.702 
 
 CGTAGACTAGAG  66.0 
     52.880       1.234 
     20.675       4.905 
      3.055       0.721 
 
 CGTAGACTAGAG  80.0 
     58.577       3.992 
     23.360       5.010 
      3.124       0.742 
 
 CGTAGACTAGAG  94.0 
     67.617       4.522 
     26.035       4.975 
      3.184       0.743 
 
 CGTAGACTAGAG  108.0 
     75.352      11.864 
     28.275       5.085 
      3.219       0.747 
 
 CGTAGACTAGAG  122.0 
     78.008      14.556 
     30.650       5.130 
      3.264       0.759 
 
 CGTAGACTAGAG  136.0 
     80.486      14.123 
     32.890       5.060 
      3.294       0.763 
 
 CGTAGACTAGAG  150.0 
     79.750      13.500 
     35.000       5.000 
      3.324       0.763 
 
 CGTCTAGTACAG  10.0 
      4.681       0.301 
      3.335       0.555 
      1.208       0.397 
 
 CGTCTAGTACAG  24.0 
     11.633       0.258 
      5.750       0.750 
      1.476       0.472 
 
 CGTCTAGTACAG  38.0 
     18.587       0.699 
      7.705       0.915 
      1.571       0.508 
 
 CGTCTAGTACAG  52.0 
     24.861       1.628 
      9.665       0.635 
      1.657       0.473 
 
 CGTCTAGTACAG  66.0 
     29.139       0.347 
     11.260       0.850 
      1.684       0.501 
 
 CGTCTAGTACAG  80.0 
     35.267       1.049 
     13.125       0.635 
      1.757       0.492 
 
 CGTCTAGTACAG  94.0 
     44.065       1.097 
     14.885       0.515 
      1.787       0.484 
 
 CGTCTAGTACAG  108.0 
     44.623       0.404 
     16.340       0.710 
      1.809       0.497 
 
 CGTCTAGTACAG  122.0 
     46.704       0.397 
     17.690       0.770 
      1.822       0.499 
 
 CGTCTAGTACAG  136.0 
     48.787       4.591 
     19.075       0.525 
      1.836       0.493 
 
 CGTCTAGTACAG  150.0 
     49.375       5.625 
     20.500       0.500 
      1.857       0.489 
 
 CGTGTCTCTAAG  10.0 
     20.633       2.937 
      7.335       0.415 
      2.645       0.116 
 
 CGTGTCTCTAAG  24.0 
     50.670      15.392 
     14.345       1.275 
      3.319       0.205 
 
 CGTGTCTCTAAG  38.0 
     64.925      22.167 
     20.750       2.490 
      3.695       0.281 
 
 CGTGTCTCTAAG  52.0 
     80.727      29.606 
     25.980       3.450 
      3.856       0.285 
 
 CGTGTCTCTAAG  66.0 
     82.486      22.731 
     31.335       4.555 
      4.067       0.316 
 
 CGTGTCTCTAAG  80.0 
     90.762      24.615 
     35.610       5.320 
      4.126       0.314 
 
 CGTGTCTCTAAG  94.0 
     93.183      18.301 
     39.750       6.360 
      4.223       0.346 
 
 CGTGTCTCTAAG  108.0 
     92.563      18.265 
     43.615       7.675 
      4.310       0.373 
 
 CGTGTCTCTAAG  122.0 
     95.436      14.650 
     47.005       8.315 
      4.354       0.371 
 
 CGTGTCTCTAAG  136.0 
     96.542      10.443 
     50.365       9.075 
      4.402       0.390 
 
 CGTGTCTCTAAG  150.0 
     98.508       8.080 
     53.500       9.500 
      4.445       0.387 
 
 CTCGCGTGTCAG  10.0 
     17.460       6.485 
      6.620       1.360 
      2.386       0.461 
 
 CTCGCGTGTCAG  24.0 
     42.991      16.439 
     13.180       3.380 
      3.042       0.699 
 
 CTCGCGTGTCAG  38.0 
     66.436      26.178 
     19.000       4.650 
      3.371       0.729 
 
 CTCGCGTGTCAG  52.0 
     92.498      42.563 
     24.410       6.600 
      3.586       0.804 
 
 CTCGCGTGTCAG  66.0 
     96.583      40.000 
     29.060       8.060 
      3.708       0.857 
 
 CTCGCGTGTCAG  80.0 
    104.188      38.517 
     33.660       9.160 
      3.822       0.855 
 
 CTCGCGTGTCAG  94.0 
    113.201      44.445 
     38.170      10.860 
      3.928       0.895 
 
 CTCGCGTGTCAG  108.0 
    116.130      45.003 
     41.915      12.415 
      3.983       0.926 
 
 CTCGCGTGTCAG  122.0 
    124.179      50.274 
     45.990      13.590 
      4.048       0.935 
 
 CTCGCGTGTCAG  136.0 
    125.593      48.102 
     49.430      14.610 
      4.089       0.944 
 
 CTCGCGTGTCAG  150.0 
    128.396      51.968 
     53.000      16.000 
      4.135       0.964 
 
 TACACACACTAG  10.0 
     11.393       1.317 
      5.740       0.550 
      2.162       0.231 
 
 TACACACACTAG  24.0 
     26.952       4.639 
     10.510       1.090 
      2.683       0.299 
 
 TACACACACTAG  38.0 
     41.642       7.575 
     14.625       1.615 
      2.932       0.320 
 
 TACACACACTAG  52.0 
     50.576       8.769 
     18.165       2.125 
      3.047       0.349 
 
 TACACACACTAG  66.0 
     59.223      15.981 
     21.360       2.300 
      3.161       0.326 
 
 TACACACACTAG  80.0 
     67.036      12.812 
     24.685       2.905 
      3.240       0.357 
 
 TACACACACTAG  94.0 
     68.171      16.663 
     27.145       3.265 
      3.277       0.369 
 
 TACACACACTAG  108.0 
     66.460      16.574 
     30.060       3.760 
      3.348       0.376 
 
 TACACACACTAG  122.0 
     64.673      14.499 
     32.490       4.150 
      3.378       0.379 
 
 TACACACACTAG  136.0 
     64.188      14.297 
     34.780       4.540 
      3.414       0.382 
 
 TACACACACTAG  150.0 
     63.050      14.050 
     37.000       5.000 
      3.444       0.389 
 
 TACACGTGATAG  10.0 
      7.508       1.782 
      4.260       0.590 
      1.546       0.239 
 
 TACACGTGATAG  24.0 
     15.016       3.933 
      7.720       1.200 
      1.958       0.266 
 
 TACACGTGATAG  38.0 
     19.550       3.218 
     10.125       1.495 
      2.091       0.261 
 
 TACACGTGATAG  52.0 
     24.176       5.382 
     12.075       2.165 
      2.161       0.328 
 
 TACACGTGATAG  66.0 
     28.102       5.916 
     14.095       2.785 
      2.246       0.369 
 
 TACACGTGATAG  80.0 
     31.723       6.835 
     15.435       2.775 
      2.247       0.338 
 
 TACACGTGATAG  94.0 
     35.858      10.542 
     17.175       3.365 
      2.313       0.364 
 
 TACACGTGATAG  108.0 
     44.739      17.017 
     18.625       3.605 
      2.353       0.357 
 
 TACACGTGATAG  122.0 
     49.672      19.192 
     19.815       3.895 
      2.362       0.358 
 
 TACACGTGATAG  136.0 
     51.341      18.874 
     21.205       4.135 
      2.380       0.363 
 
 TACACGTGATAG  150.0 
     52.667      19.667 
     22.500       4.500 
      2.399       0.371 
 
 TACAGATCGTAG  10.0 
     12.977       7.335 
      5.945       1.805 
      2.250       0.555 
 
 TACAGATCGTAG  24.0 
     30.785      14.411 
     11.350       4.280 
      2.852       0.820 
 
 TACAGATCGTAG  38.0 
     46.419      24.784 
     15.505       6.315 
      3.065       0.936 
 
 TACAGATCGTAG  52.0 
     59.200      31.360 
     19.250       7.870 
      3.221       0.995 
 
 TACAGATCGTAG  66.0 
     69.105      32.940 
     22.575       9.175 
      3.295       1.040 
 
 TACAGATCGTAG  80.0 
     85.818      42.258 
     26.130      10.670 
      3.390       1.074 
 
 TACAGATCGTAG  94.0 
    104.033      50.297 
     29.270      12.410 
      3.438       1.123 
 
 TACAGATCGTAG  108.0 
    104.265      47.825 
     32.455      13.365 
      3.508       1.128 
 
 TACAGATCGTAG  122.0 
    109.103      50.046 
     35.150      14.580 
      3.544       1.144 
 
 TACAGATCGTAG  136.0 
    115.566      55.131 
     38.205      15.715 
      3.588       1.159 
 
 TACAGATCGTAG  150.0 
    119.500      61.500 
     41.000      17.000 
      3.622       1.176 
 
 TACGAGTATGAG  10.0 
     14.395       0.750 
      6.675       0.465 
      2.499       0.168 
 
 TACGAGTATGAG  24.0 
     26.945       2.542 
     12.535       0.615 
      3.205       0.212 
 
 TACGAGTATGAG  38.0 
     37.550       6.654 
     16.475       0.205 
      3.426       0.185 
 
 TACGAGTATGAG  52.0 
     42.538      10.243 
     20.000       0.290 
      3.590       0.180 
 
 TACGAGTATGAG  66.0 
     51.483      11.808 
     23.510       0.820 
      3.699       0.164 
 
 TACGAGTATGAG  80.0 
     57.213      14.567 
     25.970       1.500 
      3.761       0.164 
 
 TACGAGTATGAG  94.0 
     55.317      14.062 
     28.215       2.005 
      3.808       0.157 
 
 TACGAGTATGAG  108.0 
     58.084      11.662 
     30.620       2.020 
      3.855       0.171 
 
 TACGAGTATGAG  122.0 
     59.713      14.559 
     33.090       2.870 
      3.911       0.154 
 
 TACGAGTATGAG  136.0 
     59.241      14.304 
     34.985       3.395 
      3.939       0.149 
 
 TACGAGTATGAG  150.0 
     59.354      14.979 
     37.000       4.000 
      3.972       0.141 
 
 TACGCTGTCTAG  10.0 
      8.463       1.374 
      5.070       0.760 
      1.962       0.336 
 
 TACGCTGTCTAG  24.0 
     19.138       3.543 
      8.610       1.570 
      2.362       0.483 
 
 TACGCTGTCTAG  38.0 
     29.057       2.939 
     11.315       1.665 
      2.503       0.461 
 
 TACGCTGTCTAG  52.0 
     37.229       1.849 
     14.160       2.080 
      2.618       0.501 
 
 TACGCTGTCTAG  66.0 
     43.936       0.652 
     16.540       2.340 
      2.684       0.533 
 
 TACGCTGTCTAG  80.0 
     48.919       0.825 
     18.875       2.355 
      2.740       0.521 
 
 TACGCTGTCTAG  94.0 
     54.503       1.290 
     21.080       2.670 
      2.785       0.539 
 
 TACGCTGTCTAG  108.0 
     58.644       4.603 
     22.770       2.560 
      2.797       0.535 
 
 TACGCTGTCTAG  122.0 
     59.608       7.054 
     24.900       2.420 
      2.846       0.530 
 
 TACGCTGTCTAG  136.0 
     66.539      12.561 
     26.745       2.525 
      2.867       0.541 
 
 TACGCTGTCTAG  150.0 
     67.929      15.071 
     28.500       2.500 
      2.894       0.537 
 
 TACTCTCGTGAG  10.0 
     16.553         nan 
      7.380         nan 
      2.714         nan 
 
 TACTCTCGTGAG  24.0 
     30.223         nan 
     13.790         nan 
      3.473         nan 
 
 TACTCTCGTGAG  38.0 
     46.002         nan 
     18.350         nan 
      3.733         nan 
 
 TACTCTCGTGAG  52.0 
     58.554         nan 
     22.270         nan 
      3.885         nan 
 
 TACTCTCGTGAG  66.0 
     74.299         nan 
     26.090         nan 
      4.029         nan 
 
 TACTCTCGTGAG  80.0 
     84.936         nan 
     28.970         nan 
      4.081         nan 
 
 TACTCTCGTGAG  94.0 
     91.991         nan 
     32.310         nan 
      4.146         nan 
 
 TACTCTCGTGAG  108.0 
    104.275         nan 
     35.730         nan 
      4.229         nan 
 
 TACTCTCGTGAG  122.0 
    107.468         nan 
     38.650         nan 
      4.268         nan 
 
 TACTCTCGTGAG  136.0 
    109.182         nan 
     41.340         nan 
      4.304         nan 
 
 TACTCTCGTGAG  150.0 
    111.667         nan 
     44.000         nan 
      4.336         nan 
 
 TAGAGACGAGAG  10.0 
     10.080       7.990 
      4.455       2.665 
      1.479       1.113 
 
 TAGAGACGAGAG  24.0 
     20.774      17.079 
      8.220       5.590 
      1.855       1.443 
 
 TAGAGACGAGAG  38.0 
     25.132      19.092 
     11.240       7.640 
      2.014       1.543 
 
 TAGAGACGAGAG  52.0 
     29.847      22.219 
     13.995       9.705 
      2.125       1.642 
 
 TAGAGACGAGAG  66.0 
     31.830      21.885 
     16.310      11.140 
      2.216       1.703 
 
 TAGAGACGAGAG  80.0 
     35.308      23.114 
     18.480      12.280 
      2.283       1.725 
 
 TAGAGACGAGAG  94.0 
     39.299      27.360 
     20.200      13.590 
      2.303       1.755 
 
 TAGAGACGAGAG  108.0 
     40.281      28.378 
     21.855      14.515 
      2.343       1.767 
 
 TAGAGACGAGAG  122.0 
     42.303      30.319 
     23.470      15.590 
      2.361       1.790 
 
 TAGAGACGAGAG  136.0 
     43.564      31.937 
     25.115      16.615 
      2.398       1.808 
 
 TAGAGACGAGAG  150.0 
     43.083      31.583 
     26.500      17.500 
      2.415       1.823 
 
 TAGTATCAGCAG  10.0 
     18.001       7.524 
      7.320       1.160 
      2.695       0.297 
 
 TAGTATCAGCAG  24.0 
     35.950      10.437 
     13.980       3.280 
      3.408       0.508 
 
 TAGTATCAGCAG  38.0 
     51.003      14.120 
     19.060       4.750 
      3.701       0.570 
 
 TAGTATCAGCAG  52.0 
     61.406      10.036 
     23.625       6.295 
      3.887       0.645 
 
 TAGTATCAGCAG  66.0 
     77.067       0.388 
     27.395       7.065 
      3.996       0.666 
 
 TAGTATCAGCAG  80.0 
     95.950       5.680 
     31.275       8.115 
      4.089       0.711 
 
 TAGTATCAGCAG  94.0 
    107.537      15.095 
     34.710       8.600 
      4.162       0.715 
 
 TAGTATCAGCAG  108.0 
    113.787      20.741 
     37.915       8.855 
      4.221       0.723 
 
 TAGTATCAGCAG  122.0 
    121.365      25.813 
     41.330       9.560 
      4.284       0.744 
 
 TAGTATCAGCAG  136.0 
    112.131      17.727 
     44.020       9.710 
      4.314       0.743 
 
 TAGTATCAGCAG  150.0 
    114.917      16.583 
     47.000      10.000 
      4.356       0.754 
 
 TAGTGTAGATAG  10.0 
      7.819       4.494 
      4.110       1.530 
      1.432       0.661 
 
 TAGTGTAGATAG  24.0 
     21.216      13.753 
      7.800       3.130 
      1.865       0.783 
 
 TAGTGTAGATAG  38.0 
     32.046      21.530 
     10.570       4.330 
      1.992       0.833 
 
 TAGTGTAGATAG  52.0 
     39.920      27.773 
     13.025       5.845 
      2.079       0.904 
 
 TAGTGTAGATAG  66.0 
     49.806      34.503 
     15.425       7.105 
      2.152       0.940 
 
 TAGTGTAGATAG  80.0 
     56.779      40.958 
     17.695       8.625 
      2.201       0.986 
 
 TAGTGTAGATAG  94.0 
     55.937      38.502 
     19.720       9.680 
      2.242       0.994 
 
 TAGTGTAGATAG  108.0 
     62.017      42.717 
     21.780      10.890 
      2.267       1.014 
 
 TAGTGTAGATAG  122.0 
     65.166      45.600 
     23.845      12.205 
      2.309       1.035 
 
 TAGTGTAGATAG  136.0 
     66.752      47.337 
     25.740      13.460 
      2.334       1.057 
 
 TAGTGTAGATAG  150.0 
     69.750      49.750 
     27.500      14.500 
      2.354       1.064 
 
 TCACGTACTAAG  10.0 
      6.463       2.007 
      4.540       1.000 
      1.838       0.362 
 
 TCACGTACTAAG  24.0 
     12.821       1.911 
      7.125       1.445 
      2.141       0.446 
 
 TCACGTACTAAG  38.0 
     22.025       2.340 
      9.485       1.835 
      2.296       0.493 
 
 TCACGTACTAAG  52.0 
     36.536       7.134 
     11.880       2.150 
      2.393       0.504 
 
 TCACGTACTAAG  66.0 
     48.547      15.447 
     14.035       2.505 
      2.464       0.519 
 
 TCACGTACTAAG  80.0 
     59.859      18.851 
     16.045       2.565 
      2.513       0.512 
 
 TCACGTACTAAG  94.0 
     62.361      23.134 
     17.390       2.790 
      2.504       0.516 
 
 TCACGTACTAAG  108.0 
     73.183      30.723 
     19.585       3.145 
      2.552       0.527 
 
 TCACGTACTAAG  122.0 
     79.302      31.858 
     21.450       3.410 
      2.586       0.527 
 
 TCACGTACTAAG  136.0 
     76.243      29.419 
     23.270       3.700 
      2.612       0.533 
 
 TCACGTACTAAG  150.0 
     76.625      29.375 
     25.000       4.000 
      2.630       0.533 
 
 TCGATCACGTAG  10.0 
      7.920       2.610 
      4.495       1.265 
      1.625       0.597 
 
 TCGATCACGTAG  24.0 
     18.982       7.444 
      8.345       2.635 
      2.078       0.810 
 
 TCGATCACGTAG  38.0 
     25.798      10.149 
     11.040       3.530 
      2.194       0.861 
 
 TCGATCACGTAG  52.0 
     29.637      11.198 
     13.555       4.075 
      2.287       0.840 
 
 TCGATCACGTAG  66.0 
     37.970      15.016 
     15.940       4.970 
      2.353       0.889 
 
 TCGATCACGTAG  80.0 
     39.438      14.371 
     18.135       5.675 
      2.429       0.905 
 
 TCGATCACGTAG  94.0 
     43.250      15.869 
     19.935       6.325 
      2.462       0.921 
 
 TCGATCACGTAG  108.0 
     47.241      15.238 
     21.750       6.940 
      2.503       0.930 
 
 TCGATCACGTAG  122.0 
     50.096      15.698 
     23.440       7.520 
      2.527       0.949 
 
 TCGATCACGTAG  136.0 
     51.327      14.133 
     25.045       7.975 
      2.556       0.953 
 
 TCGATCACGTAG  150.0 
     57.750      12.250 
     26.500       8.500 
      2.569       0.961 
 
 TCGCACTAGTAG  10.0 
      7.140         nan 
      3.910         nan 
      1.344         nan 
 
 TCGCACTAGTAG  24.0 
     16.217         nan 
      7.080         nan 
      1.641         nan 
 
 TCGCACTAGTAG  38.0 
     17.871         nan 
      9.340         nan 
      1.768         nan 
 
 TCGCACTAGTAG  52.0 
     24.664         nan 
     11.610         nan 
      1.825         nan 
 
 TCGCACTAGTAG  66.0 
     27.573         nan 
     13.730         nan 
      1.903         nan 
 
 TCGCACTAGTAG  80.0 
     33.177         nan 
     15.410         nan 
      1.951         nan 
 
 TCGCACTAGTAG  94.0 
     42.991         nan 
     17.310         nan 
      2.025         nan 
 
 TCGCACTAGTAG  108.0 
     45.825         nan 
     18.560         nan 
      2.015         nan 
 
 TCGCACTAGTAG  122.0 
     54.624         nan 
     20.050         nan 
      2.041         nan 
 
 TCGCACTAGTAG  136.0 
     58.700         nan 
     21.960         nan 
      2.081         nan 
 
 TCGCACTAGTAG  150.0 
     58.000         nan 
     23.000         nan 
      2.084         nan 
 
 TCGTCGCTCGAG  10.0 
     11.870       1.409 
      6.340       0.220 
      2.433       0.048 
 
 TCGTCGCTCGAG  24.0 
     26.020       1.520 
     11.205       0.435 
      3.013       0.052 
 
 TCGTCGCTCGAG  38.0 
     43.264       0.412 
     15.395       0.905 
      3.276       0.099 
 
 TCGTCGCTCGAG  52.0 
     56.639       8.710 
     18.825       0.965 
      3.403       0.104 
 
 TCGTCGCTCGAG  66.0 
     63.268      10.779 
     22.125       0.935 
      3.505       0.094 
 
 TCGTCGCTCGAG  80.0 
     72.756      14.871 
     25.385       1.215 
      3.585       0.118 
 
 TCGTCGCTCGAG  94.0 
     73.953      15.108 
     27.715       1.015 
      3.614       0.105 
 
 TCGTCGCTCGAG  108.0 
     76.861      13.631 
     30.730       0.630 
      3.693       0.098 
 
 TCGTCGCTCGAG  122.0 
     82.070      14.928 
     33.320       0.620 
      3.720       0.105 
 
 TCGTCGCTCGAG  136.0 
     83.361      14.609 
     35.785       0.105 
      3.764       0.093 
 
 TCGTCGCTCGAG  150.0 
     86.500      10.000 
     38.000         nan 
      3.789       0.097 
 
 TCTACGTAGCAG  10.0 
      3.687       0.352 
      2.970       0.450 
      1.068       0.350 
 
 TCTACGTAGCAG  24.0 
      7.828       0.430 
      4.720       0.330 
      1.253       0.373 
 
 TCTACGTAGCAG  38.0 
     11.572       1.462 
      6.350       0.220 
      1.392       0.407 
 
 TCTACGTAGCAG  52.0 
     14.664       2.880 
      7.750       0.130 
      1.452       0.384 
 
 TCTACGTAGCAG  66.0 
     18.381       2.858 
      9.065       0.295 
      1.481       0.382 
 
 TCTACGTAGCAG  80.0 
     20.955       1.767 
     10.320       0.600 
      1.528       0.363 
 
 TCTACGTAGCAG  94.0 
     22.184       1.916 
     11.200       0.640 
      1.535       0.375 
 
 TCTACGTAGCAG  108.0 
     23.674       0.866 
     12.210       0.570 
      1.549       0.398 
 
 TCTACGTAGCAG  122.0 
     26.841       0.860 
     13.280       0.670 
      1.578       0.387 
 
 TCTACGTAGCAG  136.0 
     26.764       3.067 
     14.080       0.880 
      1.586       0.383 
 
 TCTACGTAGCAG  150.0 
     27.167       3.833 
     15.000       1.000 
      1.603       0.378 
 
 TCTAGCGACTAG  10.0 
     13.291       4.907 
      6.155       1.315 
      2.302       0.423 
 
 TCTAGCGACTAG  24.0 
     32.330      13.216 
     11.850       3.320 
      2.940       0.645 
 
 TCTAGCGACTAG  38.0 
     46.833      15.499 
     16.225       4.585 
      3.172       0.694 
 
 TCTAGCGACTAG  52.0 
     52.804      19.431 
     20.200       5.850 
      3.331       0.744 
 
 TCTAGCGACTAG  66.0 
     58.577      22.858 
     24.030       7.210 
      3.465       0.772 
 
 TCTAGCGACTAG  80.0 
     61.599      19.937 
     27.050       7.950 
      3.522       0.778 
 
 TCTAGCGACTAG  94.0 
     62.922      17.328 
     29.925       8.645 
      3.582       0.797 
 
 TCTAGCGACTAG  108.0 
     66.094      18.829 
     32.900       9.990 
      3.642       0.835 
 
 TCTAGCGACTAG  122.0 
     65.938      20.092 
     35.370      10.530 
      3.682       0.832 
 
 TCTAGCGACTAG  136.0 
     67.486      20.153 
     37.795      11.475 
      3.732       0.854 
 
 TCTAGCGACTAG  150.0 
     68.125      20.125 
     40.000      12.000 
      3.759       0.858 
 
 TCTATACTATAG  10.0 
     13.069       5.354 
      5.900       1.730 
      2.121       0.657 
 
 TCTATACTATAG  24.0 
     25.611       7.846 
     10.700       3.130 
      2.594       0.849 
 
 TCTATACTATAG  38.0 
     32.346       8.727 
     14.580       4.060 
      2.830       0.913 
 
 TCTATACTATAG  52.0 
     42.315       9.054 
     18.325       4.645 
      3.012       0.918 
 
 TCTATACTATAG  66.0 
     50.430      10.003 
     21.175       5.435 
      3.070       0.953 
 
 TCTATACTATAG  80.0 
     51.181       9.774 
     24.030       5.750 
      3.165       0.959 
 
 TCTATACTATAG  94.0 
     54.300      11.764 
     26.490       6.200 
      3.213       0.971 
 
 TCTATACTATAG  108.0 
     57.850      13.186 
     28.440       6.760 
      3.244       0.985 
 
 TCTATACTATAG  122.0 
     59.182      13.722 
     31.005       7.335 
      3.299       0.998 
 
 TCTATACTATAG  136.0 
     61.469      16.909 
     33.020       7.760 
      3.323       1.011 
 
 TCTATACTATAG  150.0 
     60.821      16.679 
     35.000       8.000 
      3.357       1.009 
 
 TCTCTATGCGAG  10.0 
      9.104       3.542 
      5.210       1.040 
      2.031       0.325 
 
 TCTCTATGCGAG  24.0 
     18.985       9.539 
      8.900       2.780 
      2.461       0.500 
 
 TCTCTATGCGAG  38.0 
     25.495      11.830 
     11.725       3.585 
      2.640       0.479 
 
 TCTCTATGCGAG  52.0 
     30.688      15.129 
     14.250       4.930 
      2.744       0.565 
 
 TCTCTATGCGAG  66.0 
     37.897      21.561 
     16.720       6.230 
      2.843       0.595 
 
 TCTCTATGCGAG  80.0 
     41.840      25.246 
     18.640       6.990 
      2.883       0.596 
 
 TCTCTATGCGAG  94.0 
     47.942      30.115 
     20.705       8.245 
      2.937       0.639 
 
 TCTCTATGCGAG  108.0 
     52.729      34.319 
     22.370       8.990 
      2.955       0.638 
 
 TCTCTATGCGAG  122.0 
     57.440      40.157 
     24.055      10.095 
      2.999       0.657 
 
 TCTCTATGCGAG  136.0 
     58.527      41.255 
     25.625      11.095 
      3.021       0.662 
 
 TCTCTATGCGAG  150.0 
     60.500      43.500 
     27.000      12.000 
      3.036       0.675 
 
 TGACGTATGTAG  10.0 
     11.106       4.899 
      5.785       1.155 
      2.244       0.319 
 
 TGACGTATGTAG  24.0 
     20.456       7.684 
      9.660       2.270 
      2.687       0.379 
 
 TGACGTATGTAG  38.0 
     34.123      12.026 
     13.230       3.480 
      2.929       0.472 
 
 TGACGTATGTAG  52.0 
     43.770      15.968 
     16.335       4.455 
      3.069       0.505 
 
 TGACGTATGTAG  66.0 
     48.487      14.630 
     18.515       5.525 
      3.106       0.561 
 
 TGACGTATGTAG  80.0 
     54.429      15.597 
     21.375       6.075 
      3.198       0.554 
 
 TGACGTATGTAG  94.0 
     55.592      16.937 
     23.650       6.630 
      3.232       0.554 
 
 TGACGTATGTAG  108.0 
     57.084      15.171 
     25.945       7.335 
      3.281       0.573 
 
 TGACGTATGTAG  122.0 
     57.614      16.825 
     28.085       7.855 
      3.324       0.581 
 
 TGACGTATGTAG  136.0 
     57.232      17.531 
     30.050       8.450 
      3.344       0.593 
 
 TGACGTATGTAG  150.0 
     56.250      18.083 
     32.000       9.000 
      3.373       0.600 
 
 TGATACGTCTAG  10.0 
      6.966       0.654 
      4.780       0.560 
      1.905       0.254 
 
 TGATACGTCTAG  24.0 
     13.325       0.955 
      7.780       0.720 
      2.300       0.287 
 
 TGATACGTCTAG  38.0 
     20.970       3.985 
     10.045       1.185 
      2.440       0.346 
 
 TGATACGTCTAG  52.0 
     30.595       7.556 
     12.330       1.440 
      2.553       0.359 
 
 TGATACGTCTAG  66.0 
     40.829      15.169 
     14.125       1.955 
      2.608       0.382 
 
 TGATACGTCTAG  80.0 
     48.182      20.119 
     15.800       2.320 
      2.640       0.393 
 
 TGATACGTCTAG  94.0 
     52.489      20.380 
     17.305       2.805 
      2.660       0.405 
 
 TGATACGTCTAG  108.0 
     60.776      17.755 
     19.215       3.185 
      2.704       0.408 
 
 TGATACGTCTAG  122.0 
     65.603      14.608 
     20.960       3.300 
      2.736       0.396 
 
 TGATACGTCTAG  136.0 
     67.854      11.805 
     22.485       3.695 
      2.760       0.399 
 
 TGATACGTCTAG  150.0 
     67.250       8.250 
     24.000       4.000 
      2.774       0.403 
 
 TGTACTACTCAG  10.0 
      5.418       0.978 
      3.630       0.530 
      1.306       0.298 
 
 TGTACTACTCAG  24.0 
     13.196       2.057 
      6.580       0.840 
      1.660       0.376 
 
 TGTACTACTCAG  38.0 
     17.124       3.198 
      8.580       1.060 
      1.761       0.385 
 
 TGTACTACTCAG  52.0 
     19.374       3.261 
     10.515       1.395 
      1.863       0.400 
 
 TGTACTACTCAG  66.0 
     21.689       4.629 
     12.195       1.725 
      1.939       0.406 
 
 TGTACTACTCAG  80.0 
     22.639       3.110 
     13.450       1.690 
      1.963       0.402 
 
 TGTACTACTCAG  94.0 
     24.913       3.266 
     14.500       1.670 
      1.979       0.394 
 
 TGTACTACTCAG  108.0 
     26.985       4.684 
     15.575       1.975 
      1.996       0.402 
 
 TGTACTACTCAG  122.0 
     28.677       6.825 
     16.575       2.355 
      2.023       0.415 
 
 TGTACTACTCAG  136.0 
     31.760       7.170 
     17.615       2.335 
      2.038       0.413 
 
 TGTACTACTCAG  150.0 
     32.333       7.000 
     18.500       2.500 
      2.051       0.417 
 
 TGTGAGTAGTAG  10.0 
     18.733         nan 
      7.400         nan 
      2.689         nan 
 
 TGTGAGTAGTAG  24.0 
     32.097         nan 
     13.530         nan 
      3.320         nan 
 
 TGTGAGTAGTAG  38.0 
     45.808         nan 
     19.010         nan 
      3.668         nan 
 
 TGTGAGTAGTAG  52.0 
     54.941         nan 
     23.180         nan 
      3.840         nan 
 
 TGTGAGTAGTAG  66.0 
     65.028         nan 
     27.180         nan 
      3.968         nan 
 
 TGTGAGTAGTAG  80.0 
     82.373         nan 
     30.320         nan 
      4.039         nan 
 
 TGTGAGTAGTAG  94.0 
     90.534         nan 
     33.850         nan 
      4.112         nan 
 
 TGTGAGTAGTAG  108.0 
    119.215         nan 
     37.260         nan 
      4.190         nan 
 
 TGTGAGTAGTAG  122.0 
    143.313         nan 
     40.390         nan 
      4.239         nan 
 
 TGTGAGTAGTAG  136.0 
    161.322         nan 
     43.040         nan 
      4.270         nan 
 
 TGTGAGTAGTAG  150.0 
    201.000         nan 
     46.000         nan 
      4.310         nan 
 
  SampleID  Seqs/Sample 
 chao1 Ave.  chao1 Err. 
 observed_species Ave.  observed_species Err. 
 shannon Ave.  shannon Err. 
 
 
 BW.01.1  10.0 
      9.458         nan 
      5.550         nan 
      2.195         nan 
 
 BW.01.1  24.0 
     18.133         nan 
      9.200         nan 
      2.665         nan 
 
 BW.01.1  38.0 
     26.232         nan 
     12.020         nan 
      2.835         nan 
 
 BW.01.1  52.0 
     36.648         nan 
     14.590         nan 
      2.947         nan 
 
 BW.01.1  66.0 
     37.937         nan 
     16.700         nan 
      3.032         nan 
 
 BW.01.1  80.0 
     40.032         nan 
     18.320         nan 
      3.069         nan 
 
 BW.01.1  94.0 
     50.318         nan 
     20.300         nan 
      3.092         nan 
 
 BW.01.1  108.0 
     50.094         nan 
     22.500         nan 
      3.165         nan 
 
 BW.01.1  122.0 
     46.242         nan 
     23.930         nan 
      3.181         nan 
 
 BW.01.1  136.0 
     52.703         nan 
     25.700         nan 
      3.202         nan 
 
 BW.01.1  150.0 
     51.000         nan 
     27.000         nan 
      3.214         nan 
 
 BW.01.10  10.0 
     12.647         nan 
      6.250         nan 
      2.356         nan 
 
 BW.01.10  24.0 
     28.524         nan 
     11.680         nan 
      2.961         nan 
 
 BW.01.10  38.0 
     37.325         nan 
     15.310         nan 
      3.120         nan 
 
 BW.01.10  52.0 
     45.816         nan 
     19.180         nan 
      3.309         nan 
 
 BW.01.10  66.0 
     59.458         nan 
     22.950         nan 
      3.438         nan 
 
 BW.01.10  80.0 
     67.087         nan 
     25.630         nan 
      3.479         nan 
 
 BW.01.10  94.0 
     78.057         nan 
     28.950         nan 
      3.576         nan 
 
 BW.01.10  108.0 
     87.047         nan 
     31.360         nan 
      3.593         nan 
 
 BW.01.10  122.0 
     97.598         nan 
     34.150         nan 
      3.656         nan 
 
 BW.01.10  136.0 
     99.782         nan 
     36.720         nan 
      3.684         nan 
 
 BW.01.10  150.0 
    104.000         nan 
     39.000         nan 
      3.711         nan 
 
 BW.01.2  10.0 
      3.645         nan 
      2.670         nan 
      0.778         nan 
 
 BW.01.2  24.0 
     10.032         nan 
      4.830         nan 
      0.994         nan 
 
 BW.01.2  38.0 
     15.640         nan 
      6.940         nan 
      1.121         nan 
 
 BW.01.2  52.0 
     20.715         nan 
      8.960         nan 
      1.208         nan 
 
 BW.01.2  66.0 
     22.030         nan 
     10.240         nan 
      1.209         nan 
 
 BW.01.2  80.0 
     21.851         nan 
     11.890         nan 
      1.280         nan 
 
 BW.01.2  94.0 
     22.156         nan 
     13.250         nan 
      1.326         nan 
 
 BW.01.2  108.0 
     21.839         nan 
     14.370         nan 
      1.344         nan 
 
 BW.01.2  122.0 
     22.033         nan 
     15.280         nan 
      1.346         nan 
 
 BW.01.2  136.0 
     22.075         nan 
     16.220         nan 
      1.354         nan 
 
 BW.01.2  150.0 
     21.667         nan 
     17.000         nan 
      1.374         nan 
 
 BW.01.3  10.0 
     13.232         nan 
      6.780         nan 
      2.563         nan 
 
 BW.01.3  24.0 
     29.350         nan 
     12.740         nan 
      3.274         nan 
 
 BW.01.3  38.0 
     39.987         nan 
     16.720         nan 
      3.504         nan 
 
 BW.01.3  52.0 
     58.146         nan 
     20.530         nan 
      3.652         nan 
 
 BW.01.3  66.0 
     67.540         nan 
     24.280         nan 
      3.779         nan 
 
 BW.01.3  80.0 
     76.917         nan 
     27.340         nan 
      3.849         nan 
 
 BW.01.3  94.0 
     75.768         nan 
     30.530         nan 
      3.917         nan 
 
 BW.01.3  108.0 
     71.824         nan 
     32.900         nan 
      3.954         nan 
 
 BW.01.3  122.0 
     75.283         nan 
     36.050         nan 
      4.003         nan 
 
 BW.01.3  136.0 
     72.278         nan 
     38.470         nan 
      4.040         nan 
 
 BW.01.3  150.0 
     71.000         nan 
     41.000         nan 
      4.077         nan 
 
 BW.01.4  10.0 
      1.790         nan 
      1.610         nan 
      0.294         nan 
 
 BW.01.4  24.0 
      3.260         nan 
      2.550         nan 
      0.414         nan 
 
 BW.01.4  38.0 
      4.700         nan 
      3.280         nan 
      0.448         nan 
 
 BW.01.4  52.0 
      8.010         nan 
      4.520         nan 
      0.535         nan 
 
 BW.01.4  66.0 
     10.230         nan 
      5.180         nan 
      0.531         nan 
 
 BW.01.4  80.0 
     11.370         nan 
      5.680         nan 
      0.534         nan 
 
 BW.01.4  94.0 
     13.560         nan 
      6.360         nan 
      0.537         nan 
 
 BW.01.4  108.0 
     16.785         nan 
      6.980         nan 
      0.553         nan 
 
 BW.01.4  122.0 
     20.625         nan 
      7.590         nan 
      0.555         nan 
 
 BW.01.4  136.0 
     25.690         nan 
      8.360         nan 
      0.573         nan 
 
 BW.01.4  150.0 
     30.000         nan 
      9.000         nan 
      0.579         nan 
 
 BW.01.5  10.0 
     16.330         nan 
      7.080         nan 
      2.615         nan 
 
 BW.01.5  24.0 
     31.078         nan 
     12.540         nan 
      3.169         nan 
 
 BW.01.5  38.0 
     52.156         nan 
     18.240         nan 
      3.566         nan 
 
 BW.01.5  52.0 
     58.694         nan 
     21.690         nan 
      3.655         nan 
 
 BW.01.5  66.0 
     69.374         nan 
     25.760         nan 
      3.774         nan 
 
 BW.01.5  80.0 
     80.096         nan 
     29.330         nan 
      3.880         nan 
 
 BW.01.5  94.0 
     89.044         nan 
     32.970         nan 
      3.955         nan 
 
 BW.01.5  108.0 
     96.707         nan 
     36.380         nan 
      4.015         nan 
 
 BW.01.5  122.0 
    101.616         nan 
     39.000         nan 
      4.038         nan 
 
 BW.01.5  136.0 
    105.807         nan 
     41.980         nan 
      4.093         nan 
 
 BW.01.5  150.0 
    117.500         nan 
     45.000         nan 
      4.133         nan 
 
 BW.01.6  10.0 
     21.047         nan 
      7.980         nan 
      2.869         nan 
 
 BW.01.6  24.0 
     42.126         nan 
     15.310         nan 
      3.653         nan 
 
 BW.01.6  38.0 
     63.799         nan 
     21.350         nan 
      4.009         nan 
 
 BW.01.6  52.0 
     78.377         nan 
     26.430         nan 
      4.178         nan 
 
 BW.01.6  66.0 
    100.398         nan 
     31.640         nan 
      4.365         nan 
 
 BW.01.6  80.0 
    100.524         nan 
     36.070         nan 
      4.467         nan 
 
 BW.01.6  94.0 
    109.214         nan 
     40.260         nan 
      4.548         nan 
 
 BW.01.6  108.0 
    105.583         nan 
     43.820         nan 
      4.603         nan 
 
 BW.01.6  122.0 
    116.607         nan 
     48.000         nan 
      4.675         nan 
 
 BW.01.6  136.0 
    114.702         nan 
     51.370         nan 
      4.724         nan 
 
 BW.01.6  150.0 
    118.000         nan 
     55.000         nan 
      4.773         nan 
 
 BW.01.7  10.0 
     23.570         nan 
      7.750         nan 
      2.761         nan 
 
 BW.01.7  24.0 
     66.062         nan 
     15.620         nan 
      3.524         nan 
 
 BW.01.7  38.0 
     87.092         nan 
     23.240         nan 
      3.976         nan 
 
 BW.01.7  52.0 
    110.333         nan 
     29.430         nan 
      4.141         nan 
 
 BW.01.7  66.0 
    105.217         nan 
     35.890         nan 
      4.383         nan 
 
 BW.01.7  80.0 
    115.377         nan 
     40.930         nan 
      4.440         nan 
 
 BW.01.7  94.0 
    111.484         nan 
     46.110         nan 
      4.569         nan 
 
 BW.01.7  108.0 
    110.828         nan 
     51.290         nan 
      4.683         nan 
 
 BW.01.7  122.0 
    110.086         nan 
     55.320         nan 
      4.725         nan 
 
 BW.01.7  136.0 
    106.985         nan 
     59.440         nan 
      4.792         nan 
 
 BW.01.7  150.0 
    106.588         nan 
     63.000         nan 
      4.832         nan 
 
 BW.01.8  10.0 
     23.945         nan 
      7.980         nan 
      2.847         nan 
 
 BW.01.8  24.0 
     59.431         nan 
     16.560         nan 
      3.740         nan 
 
 BW.01.8  38.0 
     92.615         nan 
     23.650         nan 
      4.100         nan 
 
 BW.01.8  52.0 
    135.062         nan 
     31.010         nan 
      4.390         nan 
 
 BW.01.8  66.0 
    136.582         nan 
     37.120         nan 
      4.564         nan 
 
 BW.01.8  80.0 
    142.705         nan 
     42.820         nan 
      4.677         nan 
 
 BW.01.8  94.0 
    157.646         nan 
     49.030         nan 
      4.823         nan 
 
 BW.01.8  108.0 
    161.133         nan 
     54.330         nan 
      4.909         nan 
 
 BW.01.8  122.0 
    174.453         nan 
     59.580         nan 
      4.984         nan 
 
 BW.01.8  136.0 
    173.695         nan 
     64.040         nan 
      5.034         nan 
 
 BW.01.8  150.0 
    180.364         nan 
     69.000         nan 
      5.099         nan 
 
 BW.01.9  10.0 
     25.525         nan 
      8.480         nan 
      2.991         nan 
 
 BW.01.9  24.0 
     46.387         nan 
     17.260         nan 
      3.916         nan 
 
 BW.01.9  38.0 
     65.123         nan 
     23.810         nan 
      4.271         nan 
 
 BW.01.9  52.0 
     71.441         nan 
     29.920         nan 
      4.532         nan 
 
 BW.01.9  66.0 
     76.679         nan 
     34.460         nan 
      4.662         nan 
 
 BW.01.9  80.0 
     90.270         nan 
     39.390         nan 
      4.801         nan 
 
 BW.01.9  94.0 
     92.441         nan 
     43.310         nan 
      4.877         nan 
 
 BW.01.9  108.0 
     93.047         nan 
     46.770         nan 
      4.944         nan 
 
 BW.01.9  122.0 
     95.552         nan 
     50.890         nan 
      5.028         nan 
 
 BW.01.9  136.0 
     94.404         nan 
     53.730         nan 
      5.057         nan 
 
 BW.01.9  150.0 
     98.333         nan 
     57.000         nan 
      5.110         nan 
 
 BW.02.1  10.0 
      6.312         nan 
      4.220         nan 
      1.651         nan 
 
 BW.02.1  24.0 
     12.370         nan 
      7.060         nan 
      2.014         nan 
 
 BW.02.1  38.0 
     16.984         nan 
      8.860         nan 
      2.093         nan 
 
 BW.02.1  52.0 
     23.038         nan 
     10.890         nan 
      2.194         nan 
 
 BW.02.1  66.0 
     25.660         nan 
     12.170         nan 
      2.226         nan 
 
 BW.02.1  80.0 
     28.064         nan 
     13.480         nan 
      2.247         nan 
 
 BW.02.1  94.0 
     32.109         nan 
     14.500         nan 
      2.254         nan 
 
 BW.02.1  108.0 
     43.021         nan 
     16.030         nan 
      2.296         nan 
 
 BW.02.1  122.0 
     50.995         nan 
     17.660         nan 
      2.340         nan 
 
 BW.02.1  136.0 
     56.048         nan 
     18.790         nan 
      2.361         nan 
 
 BW.02.1  150.0 
     59.000         nan 
     20.000         nan 
      2.372         nan 
 
 BW.02.10  10.0 
      6.932         nan 
      4.200         nan 
      1.594         nan 
 
 BW.02.10  24.0 
     18.963         nan 
      7.620         nan 
      2.006         nan 
 
 BW.02.10  38.0 
     32.350         nan 
     10.560         nan 
      2.165         nan 
 
 BW.02.10  52.0 
     45.798         nan 
     13.220         nan 
      2.235         nan 
 
 BW.02.10  66.0 
     51.646         nan 
     15.770         nan 
      2.333         nan 
 
 BW.02.10  80.0 
     62.569         nan 
     18.350         nan 
      2.382         nan 
 
 BW.02.10  94.0 
     72.139         nan 
     21.060         nan 
      2.441         nan 
 
 BW.02.10  108.0 
     87.216         nan 
     23.190         nan 
      2.471         nan 
 
 BW.02.10  122.0 
     92.563         nan 
     25.520         nan 
      2.505         nan 
 
 BW.02.10  136.0 
     94.609         nan 
     27.830         nan 
      2.531         nan 
 
 BW.02.10  150.0 
     93.250         nan 
     30.000         nan 
      2.561         nan 
 
 BW.02.2  10.0 
      6.080         nan 
      4.610         nan 
      1.939         nan 
 
 BW.02.2  24.0 
     12.058         nan 
      7.190         nan 
      2.311         nan 
 
 BW.02.2  38.0 
     16.133         nan 
      8.460         nan 
      2.346         nan 
 
 BW.02.2  52.0 
     24.332         nan 
     10.580         nan 
      2.478         nan 
 
 BW.02.2  66.0 
     29.536         nan 
     12.320         nan 
      2.539         nan 
 
 BW.02.2  80.0 
     33.723         nan 
     13.470         nan 
      2.546         nan 
 
 BW.02.2  94.0 
     35.568         nan 
     14.900         nan 
      2.569         nan 
 
 BW.02.2  108.0 
     35.705         nan 
     16.070         nan 
      2.587         nan 
 
 BW.02.2  122.0 
     34.077         nan 
     17.610         nan 
      2.630         nan 
 
 BW.02.2  136.0 
     35.694         nan 
     19.000         nan 
      2.646         nan 
 
 BW.02.2  150.0 
     33.200         nan 
     20.000         nan 
      2.658         nan 
 
 BW.02.3  10.0 
      9.058         nan 
      5.400         nan 
      2.110         nan 
 
 BW.02.3  24.0 
     21.968         nan 
      9.210         nan 
      2.527         nan 
 
 BW.02.3  38.0 
     37.643         nan 
     12.450         nan 
      2.696         nan 
 
 BW.02.3  52.0 
     58.170         nan 
     15.600         nan 
      2.831         nan 
 
 BW.02.3  66.0 
     62.150         nan 
     17.820         nan 
      2.862         nan 
 
 BW.02.3  80.0 
     73.822         nan 
     21.140         nan 
      2.974         nan 
 
 BW.02.3  94.0 
     86.112         nan 
     23.360         nan 
      3.001         nan 
 
 BW.02.3  108.0 
     92.334         nan 
     25.930         nan 
      3.038         nan 
 
 BW.02.3  122.0 
     83.474         nan 
     28.370         nan 
      3.081         nan 
 
 BW.02.3  136.0 
     90.713         nan 
     30.830         nan 
      3.114         nan 
 
 BW.02.3  150.0 
     88.200         nan 
     33.000         nan 
      3.139         nan 
 
 BW.02.4  10.0 
     12.475         nan 
      6.620         nan 
      2.520         nan 
 
 BW.02.4  24.0 
     32.001         nan 
     11.530         nan 
      3.068         nan 
 
 BW.02.4  38.0 
     44.231         nan 
     15.690         nan 
      3.325         nan 
 
 BW.02.4  52.0 
     60.826         nan 
     19.190         nan 
      3.436         nan 
 
 BW.02.4  66.0 
     71.918         nan 
     22.700         nan 
      3.539         nan 
 
 BW.02.4  80.0 
     74.218         nan 
     25.850         nan 
      3.623         nan 
 
 BW.02.4  94.0 
     81.833         nan 
     28.550         nan 
      3.647         nan 
 
 BW.02.4  108.0 
     78.776         nan 
     32.030         nan 
      3.726         nan 
 
 BW.02.4  122.0 
     73.084         nan 
     34.770         nan 
      3.775         nan 
 
 BW.02.4  136.0 
     77.372         nan 
     37.690         nan 
      3.810         nan 
 
 BW.02.4  150.0 
     72.500         nan 
     40.000         nan 
      3.840         nan 
 
 BW.02.5  10.0 
      8.470         nan 
      5.540         nan 
      2.200         nan 
 
 BW.02.5  24.0 
     14.732         nan 
      8.570         nan 
      2.587         nan 
 
 BW.02.5  38.0 
     24.365         nan 
     11.320         nan 
      2.789         nan 
 
 BW.02.5  52.0 
     43.670         nan 
     14.030         nan 
      2.898         nan 
 
 BW.02.5  66.0 
     63.993         nan 
     16.540         nan 
      2.983         nan 
 
 BW.02.5  80.0 
     78.710         nan 
     18.610         nan 
      3.025         nan 
 
 BW.02.5  94.0 
     85.495         nan 
     20.180         nan 
      3.020         nan 
 
 BW.02.5  108.0 
    103.907         nan 
     22.730         nan 
      3.079         nan 
 
 BW.02.5  122.0 
    111.160         nan 
     24.860         nan 
      3.113         nan 
 
 BW.02.5  136.0 
    105.662         nan 
     26.970         nan 
      3.145         nan 
 
 BW.02.5  150.0 
    106.000         nan 
     29.000         nan 
      3.164         nan 
 
 BW.02.6  10.0 
      4.380         nan 
      2.780         nan 
      0.812         nan 
 
 BW.02.6  24.0 
     11.375         nan 
      5.000         nan 
      1.004         nan 
 
 BW.02.6  38.0 
     17.888         nan 
      6.790         nan 
      1.063         nan 
 
 BW.02.6  52.0 
     26.489         nan 
      9.030         nan 
      1.185         nan 
 
 BW.02.6  66.0 
     28.792         nan 
     10.410         nan 
      1.183         nan 
 
 BW.02.6  80.0 
     34.219         nan 
     12.490         nan 
      1.264         nan 
 
 BW.02.6  94.0 
     45.162         nan 
     14.370         nan 
      1.303         nan 
 
 BW.02.6  108.0 
     45.027         nan 
     15.630         nan 
      1.313         nan 
 
 BW.02.6  122.0 
     46.306         nan 
     16.920         nan 
      1.323         nan 
 
 BW.02.6  136.0 
     53.378         nan 
     18.550         nan 
      1.343         nan 
 
 BW.02.6  150.0 
     55.000         nan 
     20.000         nan 
      1.368         nan 
 
 BW.02.7  10.0 
      3.335         nan 
      2.520         nan 
      0.717         nan 
 
 BW.02.7  24.0 
      8.258         nan 
      4.390         nan 
      0.880         nan 
 
 BW.02.7  38.0 
     13.035         nan 
      6.130         nan 
      0.985         nan 
 
 BW.02.7  52.0 
     17.544         nan 
      7.880         nan 
      1.068         nan 
 
 BW.02.7  66.0 
     21.239         nan 
      9.360         nan 
      1.099         nan 
 
 BW.02.7  80.0 
     22.722         nan 
     10.920         nan 
      1.165         nan 
 
 BW.02.7  94.0 
     24.101         nan 
     11.840         nan 
      1.160         nan 
 
 BW.02.7  108.0 
     24.541         nan 
     12.780         nan 
      1.150         nan 
 
 BW.02.7  122.0 
     27.701         nan 
     13.950         nan 
      1.191         nan 
 
 BW.02.7  136.0 
     29.831         nan 
     14.960         nan 
      1.204         nan 
 
 BW.02.7  150.0 
     31.000         nan 
     16.000         nan 
      1.225         nan 
 
 BW.02.8  10.0 
      4.440         nan 
      3.100         nan 
      1.007         nan 
 
 BW.02.8  24.0 
     11.139         nan 
      5.740         nan 
      1.284         nan 
 
 BW.02.8  38.0 
     13.926         nan 
      7.520         nan 
      1.376         nan 
 
 BW.02.8  52.0 
     16.113         nan 
      9.120         nan 
      1.463         nan 
 
 BW.02.8  66.0 
     17.060         nan 
     10.470         nan 
      1.533         nan 
 
 BW.02.8  80.0 
     19.529         nan 
     11.760         nan 
      1.560         nan 
 
 BW.02.8  94.0 
     21.647         nan 
     12.830         nan 
      1.585         nan 
 
 BW.02.8  108.0 
     22.301         nan 
     13.600         nan 
      1.593         nan 
 
 BW.02.8  122.0 
     21.852         nan 
     14.220         nan 
      1.608         nan 
 
 BW.02.8  136.0 
     24.590         nan 
     15.280         nan 
      1.625         nan 
 
 BW.02.8  150.0 
     25.333         nan 
     16.000         nan 
      1.634         nan 
 
 BW.02.9  10.0 
      1.370         nan 
      1.350         nan 
      0.164         nan 
 
 BW.02.9  24.0 
      1.710         nan 
      1.630         nan 
      0.162         nan 
 
 BW.02.9  38.0 
      2.530         nan 
      2.160         nan 
      0.213         nan 
 
 BW.02.9  52.0 
      3.405         nan 
      2.630         nan 
      0.238         nan 
 
 BW.02.9  66.0 
      4.160         nan 
      3.040         nan 
      0.251         nan 
 
 BW.02.9  80.0 
      5.445         nan 
      3.540         nan 
      0.264         nan 
 
 BW.02.9  94.0 
      5.235         nan 
      3.660         nan 
      0.253         nan 
 
 BW.02.9  108.0 
      6.275         nan 
      4.110         nan 
      0.262         nan 
 
 BW.02.9  122.0 
      6.855         nan 
      4.500         nan 
      0.272         nan 
 
 BW.02.9  136.0 
      6.545         nan 
      4.720         nan 
      0.273         nan 
 
 BW.02.9  150.0 
      6.500         nan 
      5.000         nan 
      0.275         nan 
 
 BW.03.1  10.0 
     13.645         nan 
      6.210         nan 
      2.331         nan 
 
 BW.03.1  24.0 
     29.487         nan 
     11.920         nan 
      2.994         nan 
 
 BW.03.1  38.0 
     44.204         nan 
     16.270         nan 
      3.241         nan 
 
 BW.03.1  52.0 
     52.781         nan 
     20.290         nan 
      3.409         nan 
 
 BW.03.1  66.0 
     63.291         nan 
     24.330         nan 
      3.535         nan 
 
 BW.03.1  80.0 
     71.780         nan 
     27.470         nan 
      3.596         nan 
 
 BW.03.1  94.0 
     69.379         nan 
     30.220         nan 
      3.651         nan 
 
 BW.03.1  108.0 
     69.746         nan 
     32.640         nan 
      3.684         nan 
 
 BW.03.1  122.0 
     74.272         nan 
     35.960         nan 
      3.757         nan 
 
 BW.03.1  136.0 
     73.545         nan 
     38.380         nan 
      3.790         nan 
 
 BW.03.1  150.0 
     74.333         nan 
     41.000         nan 
      3.830         nan 
 
 BW.03.10  10.0 
      6.453         nan 
      4.350         nan 
      1.732         nan 
 
 BW.03.10  24.0 
     15.423         nan 
      7.470         nan 
      2.076         nan 
 
 BW.03.10  38.0 
     21.468         nan 
      9.890         nan 
      2.200         nan 
 
 BW.03.10  52.0 
     25.525         nan 
     12.040         nan 
      2.274         nan 
 
 BW.03.10  66.0 
     28.871         nan 
     13.920         nan 
      2.338         nan 
 
 BW.03.10  80.0 
     27.998         nan 
     15.410         nan 
      2.370         nan 
 
 BW.03.10  94.0 
     28.234         nan 
     16.840         nan 
      2.400         nan 
 
 BW.03.10  108.0 
     33.053         nan 
     18.450         nan 
      2.438         nan 
 
 BW.03.10  122.0 
     33.825         nan 
     19.570         nan 
      2.447         nan 
 
 BW.03.10  136.0 
     31.008         nan 
     20.890         nan 
      2.490         nan 
 
 BW.03.10  150.0 
     31.167         nan 
     22.000         nan 
      2.507         nan 
 
 BW.03.3  10.0 
      2.090         nan 
      1.790         nan 
      0.366         nan 
 
 BW.03.3  24.0 
      3.695         nan 
      2.630         nan 
      0.412         nan 
 
 BW.03.3  38.0 
      6.040         nan 
      3.600         nan 
      0.471         nan 
 
 BW.03.3  52.0 
      7.628         nan 
      4.290         nan 
      0.483         nan 
 
 BW.03.3  66.0 
      9.945         nan 
      5.170         nan 
      0.513         nan 
 
 BW.03.3  80.0 
     12.193         nan 
      6.200         nan 
      0.559         nan 
 
 BW.03.3  94.0 
     11.938         nan 
      6.610         nan 
      0.548         nan 
 
 BW.03.3  108.0 
     11.902         nan 
      7.340         nan 
      0.576         nan 
 
 BW.03.3  122.0 
     11.984         nan 
      7.880         nan 
      0.571         nan 
 
 BW.03.3  136.0 
     11.627         nan 
      8.500         nan 
      0.590         nan 
 
 BW.03.3  150.0 
     11.500         nan 
      9.000         nan 
      0.592         nan 
 
 BW.03.4  10.0 
     13.279         nan 
      6.560         nan 
      2.481         nan 
 
 BW.03.4  24.0 
     27.540         nan 
     11.640         nan 
      3.064         nan 
 
 BW.03.4  38.0 
     42.852         nan 
     16.300         nan 
      3.374         nan 
 
 BW.03.4  52.0 
     47.929         nan 
     19.790         nan 
      3.507         nan 
 
 BW.03.4  66.0 
     52.488         nan 
     23.060         nan 
      3.599         nan 
 
 BW.03.4  80.0 
     57.886         nan 
     26.600         nan 
      3.703         nan 
 
 BW.03.4  94.0 
     58.845         nan 
     28.730         nan 
      3.719         nan 
 
 BW.03.4  108.0 
     63.230         nan 
     31.360         nan 
      3.791         nan 
 
 BW.03.4  122.0 
     67.141         nan 
     33.940         nan 
      3.825         nan 
 
 BW.03.4  136.0 
     68.751         nan 
     35.890         nan 
      3.857         nan 
 
 BW.03.4  150.0 
     76.500         nan 
     38.000         nan 
      3.886         nan 
 
 BW.03.5  10.0 
      7.135         nan 
      4.030         nan 
      1.407         nan 
 
 BW.03.5  24.0 
     14.153         nan 
      7.160         nan 
      1.691         nan 
 
 BW.03.5  38.0 
     16.875         nan 
      9.760         nan 
      1.878         nan 
 
 BW.03.5  52.0 
     21.178         nan 
     12.410         nan 
      2.046         nan 
 
 BW.03.5  66.0 
     22.372         nan 
     13.850         nan 
      2.084         nan 
 
 BW.03.5  80.0 
     24.911         nan 
     15.260         nan 
      2.112         nan 
 
 BW.03.5  94.0 
     27.652         nan 
     16.770         nan 
      2.177         nan 
 
 BW.03.5  108.0 
     32.794         nan 
     17.900         nan 
      2.186         nan 
 
 BW.03.5  122.0 
     36.779         nan 
     18.840         nan 
      2.197         nan 
 
 BW.03.5  136.0 
     42.298         nan 
     19.940         nan 
      2.215         nan 
 
 BW.03.5  150.0 
     48.500         nan 
     21.000         nan 
      2.226         nan 
 
 BW.03.6  10.0 
      7.350         nan 
      4.100         nan 
      1.444         nan 
 
 BW.03.6  24.0 
     14.613         nan 
      7.190         nan 
      1.753         nan 
 
 BW.03.6  38.0 
     18.117         nan 
      9.880         nan 
      1.936         nan 
 
 BW.03.6  52.0 
     25.114         nan 
     12.400         nan 
      2.091         nan 
 
 BW.03.6  66.0 
     29.320         nan 
     14.460         nan 
      2.163         nan 
 
 BW.03.6  80.0 
     33.571         nan 
     16.250         nan 
      2.210         nan 
 
 BW.03.6  94.0 
     37.073         nan 
     17.490         nan 
      2.206         nan 
 
 BW.03.6  108.0 
     38.788         nan 
     18.860         nan 
      2.217         nan 
 
 BW.03.6  122.0 
     39.675         nan 
     20.380         nan 
      2.250         nan 
 
 BW.03.6  136.0 
     38.021         nan 
     21.660         nan 
      2.276         nan 
 
 BW.03.6  150.0 
     38.600         nan 
     23.000         nan 
      2.297         nan 
 
 BW.03.7  10.0 
      7.085         nan 
      4.450         nan 
      1.755         nan 
 
 BW.03.7  24.0 
     17.678         nan 
      7.750         nan 
      2.111         nan 
 
 BW.03.7  38.0 
     31.873         nan 
     11.380         nan 
      2.409         nan 
 
 BW.03.7  52.0 
     42.136         nan 
     13.800         nan 
      2.432         nan 
 
 BW.03.7  66.0 
     50.590         nan 
     16.620         nan 
      2.533         nan 
 
 BW.03.7  80.0 
     52.762         nan 
     19.180         nan 
      2.632         nan 
 
 BW.03.7  94.0 
     55.188         nan 
     21.460         nan 
      2.664         nan 
 
 BW.03.7  108.0 
     57.148         nan 
     23.460         nan 
      2.696         nan 
 
 BW.03.7  122.0 
     56.968         nan 
     24.950         nan 
      2.674         nan 
 
 BW.03.7  136.0 
     54.508         nan 
     27.060         nan 
      2.712         nan 
 
 BW.03.7  150.0 
     53.429         nan 
     29.000         nan 
      2.748         nan 
 
 BW.03.8  10.0 
      8.053         nan 
      5.100         nan 
      2.009         nan 
 
 BW.03.8  24.0 
     16.135         nan 
      8.490         nan 
      2.432         nan 
 
 BW.03.8  38.0 
     22.312         nan 
     10.850         nan 
      2.531         nan 
 
 BW.03.8  52.0 
     32.647         nan 
     13.510         nan 
      2.659         nan 
 
 BW.03.8  66.0 
     42.212         nan 
     15.870         nan 
      2.736         nan 
 
 BW.03.8  80.0 
     42.798         nan 
     17.820         nan 
      2.797         nan 
 
 BW.03.8  94.0 
     42.926         nan 
     19.200         nan 
      2.818         nan 
 
 BW.03.8  108.0 
     48.031         nan 
     21.210         nan 
      2.868         nan 
 
 BW.03.8  122.0 
     51.452         nan 
     23.040         nan 
      2.899         nan 
 
 BW.03.8  136.0 
     55.163         nan 
     24.450         nan 
      2.915         nan 
 
 BW.03.8  150.0 
     56.000         nan 
     26.000         nan 
      2.941         nan 
 
 BW.03.9  10.0 
      9.167         nan 
      5.360         nan 
      2.119         nan 
 
 BW.03.9  24.0 
     21.315         nan 
      9.200         nan 
      2.548         nan 
 
 BW.03.9  38.0 
     33.984         nan 
     12.390         nan 
      2.734         nan 
 
 BW.03.9  52.0 
     45.453         nan 
     15.680         nan 
      2.891         nan 
 
 BW.03.9  66.0 
     52.049         nan 
     18.440         nan 
      2.970         nan 
 
 BW.03.9  80.0 
     57.387         nan 
     20.890         nan 
      3.032         nan 
 
 BW.03.9  94.0 
     61.895         nan 
     23.860         nan 
      3.113         nan 
 
 BW.03.9  108.0 
     61.553         nan 
     25.900         nan 
      3.126         nan 
 
 BW.03.9  122.0 
     60.813         nan 
     28.270         nan 
      3.170         nan 
 
 BW.03.9  136.0 
     57.058         nan 
     29.950         nan 
      3.186         nan 
 
 BW.03.9  150.0 
     55.750         nan 
     32.000         nan 
      3.216         nan 
 
 BW.04.1  10.0 
     14.675         nan 
      6.990         nan 
      2.620         nan 
 
 BW.04.1  24.0 
     30.584         nan 
     12.670         nan 
      3.290         nan 
 
 BW.04.1  38.0 
     57.105         nan 
     17.910         nan 
      3.612         nan 
 
 BW.04.1  52.0 
     56.987         nan 
     21.570         nan 
      3.742         nan 
 
 BW.04.1  66.0 
     63.085         nan 
     25.760         nan 
      3.897         nan 
 
 BW.04.1  80.0 
     64.641         nan 
     28.510         nan 
      3.925         nan 
 
 BW.04.1  94.0 
     65.369         nan 
     32.230         nan 
      4.030         nan 
 
 BW.04.1  108.0 
     66.895         nan 
     35.040         nan 
      4.087         nan 
 
 BW.04.1  122.0 
     65.450         nan 
     37.200         nan 
      4.106         nan 
 
 BW.04.1  136.0 
     65.456         nan 
     39.980         nan 
      4.166         nan 
 
 BW.04.1  150.0 
     65.000         nan 
     42.000         nan 
      4.186         nan 
 
 BW.04.10  10.0 
     12.313         nan 
      5.640         nan 
      2.094         nan 
 
 BW.04.10  24.0 
     34.969         nan 
     10.930         nan 
      2.647         nan 
 
 BW.04.10  38.0 
     53.576         nan 
     14.900         nan 
      2.825         nan 
 
 BW.04.10  52.0 
     67.693         nan 
     18.870         nan 
      2.982         nan 
 
 BW.04.10  66.0 
     84.308         nan 
     22.530         nan 
      3.092         nan 
 
 BW.04.10  80.0 
     97.737         nan 
     26.320         nan 
      3.187         nan 
 
 BW.04.10  94.0 
     94.439         nan 
     29.400         nan 
      3.237         nan 
 
 BW.04.10  108.0 
    104.735         nan 
     32.670         nan 
      3.281         nan 
 
 BW.04.10  122.0 
    110.766         nan 
     36.050         nan 
      3.344         nan 
 
 BW.04.10  136.0 
    114.089         nan 
     39.200         nan 
      3.391         nan 
 
 BW.04.10  150.0 
    119.500         nan 
     42.000         nan 
      3.418         nan 
 
 BW.04.2  10.0 
      7.125         nan 
      4.340         nan 
      1.684         nan 
 
 BW.04.2  24.0 
     13.890         nan 
      7.250         nan 
      2.062         nan 
 
 BW.04.2  38.0 
     22.318         nan 
      9.790         nan 
      2.230         nan 
 
 BW.04.2  52.0 
     26.612         nan 
     11.370         nan 
      2.265         nan 
 
 BW.04.2  66.0 
     37.992         nan 
     13.580         nan 
      2.353         nan 
 
 BW.04.2  80.0 
     49.468         nan 
     15.510         nan 
      2.406         nan 
 
 BW.04.2  94.0 
     54.660         nan 
     16.960         nan 
      2.411         nan 
 
 BW.04.2  108.0 
     56.370         nan 
     18.730         nan 
      2.430         nan 
 
 BW.04.2  122.0 
     62.696         nan 
     20.690         nan 
      2.470         nan 
 
 BW.04.2  136.0 
     61.673         nan 
     22.400         nan 
      2.489         nan 
 
 BW.04.2  150.0 
     58.000         nan 
     24.000         nan 
      2.515         nan 
 
 BW.04.3  10.0 
     26.200         nan 
      8.680         nan 
      3.042         nan 
 
 BW.04.3  24.0 
     59.184         nan 
     18.170         nan 
      4.011         nan 
 
 BW.04.3  38.0 
     68.557         nan 
     25.120         nan 
      4.357         nan 
 
 BW.04.3  52.0 
     76.084         nan 
     31.430         nan 
      4.626         nan 
 
 BW.04.3  66.0 
     78.806         nan 
     37.620         nan 
      4.825         nan 
 
 BW.04.3  80.0 
     82.485         nan 
     42.030         nan 
      4.930         nan 
 
 BW.04.3  94.0 
     85.589         nan 
     46.720         nan 
      5.031         nan 
 
 BW.04.3  108.0 
     87.387         nan 
     51.010         nan 
      5.122         nan 
 
 BW.04.3  122.0 
     89.141         nan 
     54.670         nan 
      5.186         nan 
 
 BW.04.3  136.0 
     89.992         nan 
     58.040         nan 
      5.241         nan 
 
 BW.04.3  150.0 
     88.353         nan 
     61.000         nan 
      5.288         nan 
 
 BW.04.4  10.0 
      5.578         nan 
      3.720         nan 
      1.339         nan 
 
 BW.04.4  24.0 
     13.052         nan 
      6.200         nan 
      1.577         nan 
 
 BW.04.4  38.0 
     22.705         nan 
      8.670         nan 
      1.753         nan 
 
 BW.04.4  52.0 
     29.427         nan 
     10.540         nan 
      1.790         nan 
 
 BW.04.4  66.0 
     39.005         nan 
     12.590         nan 
      1.825         nan 
 
 BW.04.4  80.0 
     47.828         nan 
     15.050         nan 
      1.926         nan 
 
 BW.04.4  94.0 
     56.888         nan 
     17.090         nan 
      1.958         nan 
 
 BW.04.4  108.0 
     52.736         nan 
     18.590         nan 
      1.987         nan 
 
 BW.04.4  122.0 
     61.386         nan 
     20.420         nan 
      2.006         nan 
 
 BW.04.4  136.0 
     70.329         nan 
     22.260         nan 
      2.037         nan 
 
 BW.04.4  150.0 
     75.000         nan 
     24.000         nan 
      2.055         nan 
 
 BW.04.5  10.0 
      4.655         nan 
      3.660         nan 
      1.514         nan 
 
 BW.04.5  24.0 
      9.670         nan 
      5.580         nan 
      1.718         nan 
 
 BW.04.5  38.0 
     18.045         nan 
      7.550         nan 
      1.819         nan 
 
 BW.04.5  52.0 
     29.045         nan 
      9.680         nan 
      1.952         nan 
 
 BW.04.5  66.0 
     40.510         nan 
     11.350         nan 
      1.963         nan 
 
 BW.04.5  80.0 
     56.175         nan 
     12.930         nan 
      1.997         nan 
 
 BW.04.5  94.0 
     82.840         nan 
     15.140         nan 
      2.061         nan 
 
 BW.04.5  108.0 
    104.325         nan 
     16.650         nan 
      2.075         nan 
 
 BW.04.5  122.0 
    130.860         nan 
     18.420         nan 
      2.094         nan 
 
 BW.04.5  136.0 
    163.390         nan 
     20.390         nan 
      2.130         nan 
 
 BW.04.5  150.0 
    193.000         nan 
     22.000         nan 
      2.140         nan 
 
 BW.04.6  10.0 
     12.710         nan 
      6.290         nan 
      2.393         nan 
 
 BW.04.6  24.0 
     31.591         nan 
     11.600         nan 
      2.983         nan 
 
 BW.04.6  38.0 
     49.217         nan 
     16.240         nan 
      3.252         nan 
 
 BW.04.6  52.0 
     59.345         nan 
     20.290         nan 
      3.395         nan 
 
 BW.04.6  66.0 
     75.205         nan 
     23.660         nan 
      3.487         nan 
 
 BW.04.6  80.0 
     79.848         nan 
     27.590         nan 
      3.596         nan 
 
 BW.04.6  94.0 
     84.833         nan 
     30.410         nan 
      3.645         nan 
 
 BW.04.6  108.0 
     83.034         nan 
     33.820         nan 
      3.724         nan 
 
 BW.04.6  122.0 
     79.172         nan 
     36.640         nan 
      3.757         nan 
 
 BW.04.6  136.0 
     78.485         nan 
     39.320         nan 
      3.796         nan 
 
 BW.04.6  150.0 
     77.100         nan 
     42.000         nan 
      3.833         nan 
 
 BW.04.7  10.0 
      5.725         nan 
      3.670         nan 
      1.307         nan 
 
 BW.04.7  24.0 
     11.083         nan 
      6.520         nan 
      1.692         nan 
 
 BW.04.7  38.0 
     16.331         nan 
      8.630         nan 
      1.830         nan 
 
 BW.04.7  52.0 
     18.794         nan 
      9.910         nan 
      1.833         nan 
 
 BW.04.7  66.0 
     22.187         nan 
     11.310         nan 
      1.877         nan 
 
 BW.04.7  80.0 
     24.889         nan 
     12.660         nan 
      1.910         nan 
 
 BW.04.7  94.0 
     25.317         nan 
     13.810         nan 
      1.949         nan 
 
 BW.04.7  108.0 
     27.722         nan 
     15.020         nan 
      1.996         nan 
 
 BW.04.7  122.0 
     30.481         nan 
     15.920         nan 
      2.004         nan 
 
 BW.04.7  136.0 
     32.467         nan 
     17.070         nan 
      2.016         nan 
 
 BW.04.7  150.0 
     33.000         nan 
     18.000         nan 
      2.029         nan 
 
 BW.04.8  10.0 
     20.312         nan 
      7.750         nan 
      2.805         nan 
 
 BW.04.8  24.0 
     45.196         nan 
     15.630         nan 
      3.672         nan 
 
 BW.04.8  38.0 
     71.203         nan 
     21.820         nan 
      4.001         nan 
 
 BW.04.8  52.0 
     90.560         nan 
     27.120         nan 
      4.216         nan 
 
 BW.04.8  66.0 
    102.045         nan 
     31.750         nan 
      4.335         nan 
 
 BW.04.8  80.0 
    128.076         nan 
     36.800         nan 
      4.465         nan 
 
 BW.04.8  94.0 
    154.330         nan 
     41.680         nan 
      4.561         nan 
 
 BW.04.8  108.0 
    152.091         nan 
     45.820         nan 
      4.636         nan 
 
 BW.04.8  122.0 
    159.149         nan 
     49.730         nan 
      4.687         nan 
 
 BW.04.8  136.0 
    170.697         nan 
     53.920         nan 
      4.747         nan 
 
 BW.04.8  150.0 
    181.000         nan 
     58.000         nan 
      4.797         nan 
 
 BW.04.9  10.0 
      7.088         nan 
      4.310         nan 
      1.626         nan 
 
 BW.04.9  24.0 
     15.595         nan 
      7.040         nan 
      1.880         nan 
 
 BW.04.9  38.0 
     26.118         nan 
      9.650         nan 
      2.042         nan 
 
 BW.04.9  52.0 
     35.380         nan 
     12.080         nan 
      2.117         nan 
 
 BW.04.9  66.0 
     44.588         nan 
     14.200         nan 
      2.152         nan 
 
 BW.04.9  80.0 
     48.093         nan 
     16.520         nan 
      2.219         nan 
 
 BW.04.9  94.0 
     55.793         nan 
     18.410         nan 
      2.246         nan 
 
 BW.04.9  108.0 
     63.247         nan 
     20.210         nan 
      2.262         nan 
 
 BW.04.9  122.0 
     66.661         nan 
     22.480         nan 
      2.316         nan 
 
 BW.04.9  136.0 
     79.100         nan 
     24.220         nan 
      2.326         nan 
 
 BW.04.9  150.0 
     83.000         nan 
     26.000         nan 
      2.357         nan 
 
 BW.05.1  10.0 
      5.310         nan 
      3.230         nan 
      1.028         nan 
 
 BW.05.1  24.0 
     11.537         nan 
      5.710         nan 
      1.268         nan 
 
 BW.05.1  38.0 
     15.649         nan 
      7.510         nan 
      1.332         nan 
 
 BW.05.1  52.0 
     18.440         nan 
      9.480         nan 
      1.447         nan 
 
 BW.05.1  66.0 
     22.954         nan 
     10.970         nan 
      1.464         nan 
 
 BW.05.1  80.0 
     25.067         nan 
     12.460         nan 
      1.524         nan 
 
 BW.05.1  94.0 
     27.380         nan 
     13.610         nan 
      1.541         nan 
 
 BW.05.1  108.0 
     32.003         nan 
     14.810         nan 
      1.573         nan 
 
 BW.05.1  122.0 
     34.398         nan 
     15.920         nan 
      1.578         nan 
 
 BW.05.1  136.0 
     37.194         nan 
     17.070         nan 
      1.604         nan 
 
 BW.05.1  150.0 
     45.500         nan 
     18.000         nan 
      1.608         nan 
 
 BW.05.10  10.0 
     10.095         nan 
      5.200         nan 
      1.929         nan 
 
 BW.05.10  24.0 
     18.405         nan 
      9.010         nan 
      2.294         nan 
 
 BW.05.10  38.0 
     24.971         nan 
     12.010         nan 
      2.467         nan 
 
 BW.05.10  52.0 
     31.391         nan 
     14.860         nan 
      2.640         nan 
 
 BW.05.10  66.0 
     31.737         nan 
     16.780         nan 
      2.701         nan 
 
 BW.05.10  80.0 
     38.183         nan 
     19.090         nan 
      2.775         nan 
 
 BW.05.10  94.0 
     43.259         nan 
     20.670         nan 
      2.806         nan 
 
 BW.05.10  108.0 
     48.751         nan 
     22.590         nan 
      2.846         nan 
 
 BW.05.10  122.0 
     47.196         nan 
     24.010         nan 
      2.865         nan 
 
 BW.05.10  136.0 
     49.574         nan 
     25.730         nan 
      2.897         nan 
 
 BW.05.10  150.0 
     48.000         nan 
     27.000         nan 
      2.915         nan 
 
 BW.05.2  10.0 
      7.140         nan 
      3.910         nan 
      1.344         nan 
 
 BW.05.2  24.0 
     16.217         nan 
      7.080         nan 
      1.641         nan 
 
 BW.05.2  38.0 
     17.871         nan 
      9.340         nan 
      1.768         nan 
 
 BW.05.2  52.0 
     24.664         nan 
     11.610         nan 
      1.825         nan 
 
 BW.05.2  66.0 
     27.573         nan 
     13.730         nan 
      1.903         nan 
 
 BW.05.2  80.0 
     33.177         nan 
     15.410         nan 
      1.951         nan 
 
 BW.05.2  94.0 
     42.991         nan 
     17.310         nan 
      2.025         nan 
 
 BW.05.2  108.0 
     45.825         nan 
     18.560         nan 
      2.015         nan 
 
 BW.05.2  122.0 
     54.624         nan 
     20.050         nan 
      2.041         nan 
 
 BW.05.2  136.0 
     58.700         nan 
     21.960         nan 
      2.081         nan 
 
 BW.05.2  150.0 
     58.000         nan 
     23.000         nan 
      2.084         nan 
 
 BW.05.3  10.0 
      8.383         nan 
      4.840         nan 
      1.879         nan 
 
 BW.05.3  24.0 
     19.114         nan 
      8.530         nan 
      2.294         nan 
 
 BW.05.3  38.0 
     31.334         nan 
     11.640         nan 
      2.478         nan 
 
 BW.05.3  52.0 
     33.374         nan 
     14.350         nan 
      2.587         nan 
 
 BW.05.3  66.0 
     35.719         nan 
     16.820         nan 
      2.693         nan 
 
 BW.05.3  80.0 
     41.662         nan 
     19.100         nan 
      2.744         nan 
 
 BW.05.3  94.0 
     45.593         nan 
     21.280         nan 
      2.785         nan 
 
 BW.05.3  108.0 
     47.265         nan 
     22.910         nan 
      2.807         nan 
 
 BW.05.3  122.0 
     45.846         nan 
     24.840         nan 
      2.850         nan 
 
 BW.05.3  136.0 
     47.333         nan 
     26.320         nan 
      2.877         nan 
 
 BW.05.3  150.0 
     48.000         nan 
     28.000         nan 
      2.901         nan 
 
 BW.05.4  10.0 
     18.423         nan 
      7.630         nan 
      2.778         nan 
 
 BW.05.4  24.0 
     33.457         nan 
     13.830         nan 
      3.443         nan 
 
 BW.05.4  38.0 
     41.073         nan 
     18.640         nan 
      3.744         nan 
 
 BW.05.4  52.0 
     51.369         nan 
     22.970         nan 
      3.930         nan 
 
 BW.05.4  66.0 
     60.433         nan 
     26.610         nan 
      4.023         nan 
 
 BW.05.4  80.0 
     60.955         nan 
     29.780         nan 
      4.124         nan 
 
 BW.05.4  94.0 
     66.064         nan 
     32.690         nan 
      4.184         nan 
 
 BW.05.4  108.0 
     71.037         nan 
     35.200         nan 
      4.228         nan 
 
 BW.05.4  122.0 
     72.904         nan 
     38.340         nan 
      4.298         nan 
 
 BW.05.4  136.0 
     78.378         nan 
     40.780         nan 
      4.334         nan 
 
 BW.05.4  150.0 
     77.500         nan 
     43.000         nan 
      4.366         nan 
 
 BW.05.5  10.0 
      6.207         nan 
      4.630         nan 
      1.925         nan 
 
 BW.05.5  24.0 
     12.772         nan 
      7.390         nan 
      2.307         nan 
 
 BW.05.5  38.0 
     22.098         nan 
      9.750         nan 
      2.456         nan 
 
 BW.05.5  52.0 
     27.802         nan 
     11.880         nan 
      2.564         nan 
 
 BW.05.5  66.0 
     33.857         nan 
     12.990         nan 
      2.545         nan 
 
 BW.05.5  80.0 
     38.832         nan 
     15.300         nan 
      2.644         nan 
 
 BW.05.5  94.0 
     38.655         nan 
     17.020         nan 
      2.678         nan 
 
 BW.05.5  108.0 
     41.913         nan 
     18.610         nan 
      2.708         nan 
 
 BW.05.5  122.0 
     40.790         nan 
     20.230         nan 
      2.742         nan 
 
 BW.05.5  136.0 
     39.702         nan 
     21.600         nan 
      2.751         nan 
 
 BW.05.5  150.0 
     38.167         nan 
     23.000         nan 
      2.773         nan 
 
 BW.05.6  10.0 
     18.733         nan 
      7.400         nan 
      2.689         nan 
 
 BW.05.6  24.0 
     32.097         nan 
     13.530         nan 
      3.320         nan 
 
 BW.05.6  38.0 
     45.808         nan 
     19.010         nan 
      3.668         nan 
 
 BW.05.6  52.0 
     54.941         nan 
     23.180         nan 
      3.840         nan 
 
 BW.05.6  66.0 
     65.028         nan 
     27.180         nan 
      3.968         nan 
 
 BW.05.6  80.0 
     82.373         nan 
     30.320         nan 
      4.039         nan 
 
 BW.05.6  94.0 
     90.534         nan 
     33.850         nan 
      4.112         nan 
 
 BW.05.6  108.0 
    119.215         nan 
     37.260         nan 
      4.190         nan 
 
 BW.05.6  122.0 
    143.313         nan 
     40.390         nan 
      4.239         nan 
 
 BW.05.6  136.0 
    161.322         nan 
     43.040         nan 
      4.270         nan 
 
 BW.05.6  150.0 
    201.000         nan 
     46.000         nan 
      4.310         nan 
 
 BW.05.7  10.0 
     20.420         nan 
      7.700         nan 
      2.782         nan 
 
 BW.05.7  24.0 
     40.184         nan 
     14.680         nan 
      3.534         nan 
 
 BW.05.7  38.0 
     51.806         nan 
     19.740         nan 
      3.793         nan 
 
 BW.05.7  52.0 
     72.172         nan 
     25.160         nan 
      4.037         nan 
 
 BW.05.7  66.0 
     74.303         nan 
     29.530         nan 
      4.181         nan 
 
 BW.05.7  80.0 
     83.897         nan 
     33.390         nan 
      4.265         nan 
 
 BW.05.7  94.0 
     93.793         nan 
     37.610         nan 
      4.364         nan 
 
 BW.05.7  108.0 
    101.114         nan 
     41.530         nan 
      4.449         nan 
 
 BW.05.7  122.0 
    110.448         nan 
     44.710         nan 
      4.489         nan 
 
 BW.05.7  136.0 
    119.193         nan 
     47.970         nan 
      4.546         nan 
 
 BW.05.7  150.0 
    126.429         nan 
     51.000         nan 
      4.576         nan 
 
 BW.05.8  10.0 
     15.928         nan 
      6.890         nan 
      2.531         nan 
 
 BW.05.8  24.0 
     32.732         nan 
     12.560         nan 
      3.040         nan 
 
 BW.05.8  38.0 
     39.724         nan 
     17.740         nan 
      3.381         nan 
 
 BW.05.8  52.0 
     48.960         nan 
     21.820         nan 
      3.538         nan 
 
 BW.05.8  66.0 
     50.799         nan 
     25.180         nan 
      3.620         nan 
 
 BW.05.8  80.0 
     56.487         nan 
     28.880         nan 
      3.760         nan 
 
 BW.05.8  94.0 
     61.309         nan 
     31.510         nan 
      3.791         nan 
 
 BW.05.8  108.0 
     65.015         nan 
     33.780         nan 
      3.848         nan 
 
 BW.05.8  122.0 
     72.160         nan 
     36.780         nan 
      3.906         nan 
 
 BW.05.8  136.0 
     74.976         nan 
     38.770         nan 
      3.934         nan 
 
 BW.05.8  150.0 
     83.167         nan 
     41.000         nan 
      3.966         nan 
 
 BW.05.9  10.0 
     10.213         nan 
      5.430         nan 
      2.053         nan 
 
 BW.05.9  24.0 
     26.845         nan 
     10.180         nan 
      2.590         nan 
 
 BW.05.9  38.0 
     37.753         nan 
     14.170         nan 
      2.803         nan 
 
 BW.05.9  52.0 
     49.701         nan 
     17.610         nan 
      2.965         nan 
 
 BW.05.9  66.0 
     55.694         nan 
     20.780         nan 
      3.055         nan 
 
 BW.05.9  80.0 
     54.253         nan 
     22.750         nan 
      3.076         nan 
 
 BW.05.9  94.0 
     63.275         nan 
     25.920         nan 
      3.140         nan 
 
 BW.05.9  108.0 
     68.530         nan 
     28.310         nan 
      3.173         nan 
 
 BW.05.9  122.0 
     66.725         nan 
     30.570         nan 
      3.219         nan 
 
 BW.05.9  136.0 
     68.202         nan 
     33.020         nan 
      3.265         nan 
 
 BW.05.9  150.0 
     68.000         nan 
     35.000         nan 
      3.286         nan 
 
 BW.06.1  10.0 
     15.532         nan 
      7.160         nan 
      2.659         nan 
 
 BW.06.1  24.0 
     30.428         nan 
     13.000         nan 
      3.328         nan 
 
 BW.06.1  38.0 
     44.470         nan 
     17.290         nan 
      3.569         nan 
 
 BW.06.1  52.0 
     52.202         nan 
     21.330         nan 
      3.765         nan 
 
 BW.06.1  66.0 
     64.608         nan 
     24.910         nan 
      3.884         nan 
 
 BW.06.1  80.0 
     84.138         nan 
     27.500         nan 
      3.917         nan 
 
 BW.06.1  94.0 
     86.573         nan 
     30.740         nan 
      3.992         nan 
 
 BW.06.1  108.0 
    110.394         nan 
     33.990         nan 
      4.060         nan 
 
 BW.06.1  122.0 
    109.400         nan 
     36.370         nan 
      4.096         nan 
 
 BW.06.1  136.0 
    120.975         nan 
     39.450         nan 
      4.153         nan 
 
 BW.06.1  150.0 
    117.600         nan 
     42.000         nan 
      4.180         nan 
 
 BW.06.10  10.0 
     10.477         nan 
      6.160         nan 
      2.398         nan 
 
 BW.06.10  24.0 
     25.513         nan 
     10.700         nan 
      2.901         nan 
 
 BW.06.10  38.0 
     36.883         nan 
     14.310         nan 
      3.130         nan 
 
 BW.06.10  52.0 
     51.370         nan 
     17.330         nan 
      3.242         nan 
 
 BW.06.10  66.0 
     77.456         nan 
     20.330         nan 
      3.330         nan 
 
 BW.06.10  80.0 
    101.629         nan 
     23.160         nan 
      3.378         nan 
 
 BW.06.10  94.0 
    122.632         nan 
     26.110         nan 
      3.447         nan 
 
 BW.06.10  108.0 
    134.528         nan 
     29.060         nan 
      3.498         nan 
 
 BW.06.10  122.0 
    147.178         nan 
     31.770         nan 
      3.539         nan 
 
 BW.06.10  136.0 
    129.858         nan 
     34.310         nan 
      3.571         nan 
 
 BW.06.10  150.0 
    131.500         nan 
     37.000         nan 
      3.601         nan 
 
 BW.06.2  10.0 
     15.465         nan 
      6.320         nan 
      2.334         nan 
 
 BW.06.2  24.0 
     29.473         nan 
     11.910         nan 
      2.916         nan 
 
 BW.06.2  38.0 
     35.781         nan 
     15.940         nan 
      3.109         nan 
 
 BW.06.2  52.0 
     36.383         nan 
     19.330         nan 
      3.293         nan 
 
 BW.06.2  66.0 
     42.324         nan 
     22.410         nan 
      3.419         nan 
 
 BW.06.2  80.0 
     49.544         nan 
     24.770         nan 
      3.461         nan 
 
 BW.06.2  94.0 
     53.867         nan 
     26.950         nan 
      3.525         nan 
 
 BW.06.2  108.0 
     64.082         nan 
     28.910         nan 
      3.543         nan 
 
 BW.06.2  122.0 
     73.457         nan 
     31.330         nan 
      3.600         nan 
 
 BW.06.2  136.0 
     76.466         nan 
     33.000         nan 
      3.640         nan 
 
 BW.06.2  150.0 
     82.500         nan 
     35.000         nan 
      3.666         nan 
 
 BW.06.4  10.0 
     14.473         nan 
      6.190         nan 
      2.267         nan 
 
 BW.06.4  24.0 
     33.862         nan 
     11.820         nan 
      2.817         nan 
 
 BW.06.4  38.0 
     48.630         nan 
     17.400         nan 
      3.183         nan 
 
 BW.06.4  52.0 
     66.940         nan 
     21.350         nan 
      3.293         nan 
 
 BW.06.4  66.0 
     79.168         nan 
     25.470         nan 
      3.371         nan 
 
 BW.06.4  80.0 
     87.994         nan 
     29.380         nan 
      3.508         nan 
 
 BW.06.4  94.0 
     95.356         nan 
     32.650         nan 
      3.542         nan 
 
 BW.06.4  108.0 
    118.150         nan 
     36.470         nan 
      3.624         nan 
 
 BW.06.4  122.0 
    120.714         nan 
     39.690         nan 
      3.661         nan 
 
 BW.06.4  136.0 
    124.288         nan 
     42.500         nan 
      3.687         nan 
 
 BW.06.4  150.0 
    134.000         nan 
     46.000         nan 
      3.741         nan 
 
 BW.06.5  10.0 
     13.767         nan 
      6.730         nan 
      2.539         nan 
 
 BW.06.5  24.0 
     23.862         nan 
     11.790         nan 
      3.128         nan 
 
 BW.06.5  38.0 
     37.142         nan 
     16.030         nan 
      3.406         nan 
 
 BW.06.5  52.0 
     49.042         nan 
     19.400         nan 
      3.537         nan 
 
 BW.06.5  66.0 
     60.692         nan 
     22.270         nan 
      3.609         nan 
 
 BW.06.5  80.0 
     63.530         nan 
     24.650         nan 
      3.645         nan 
 
 BW.06.5  94.0 
     67.773         nan 
     27.730         nan 
      3.753         nan 
 
 BW.06.5  108.0 
     71.154         nan 
     30.090         nan 
      3.776         nan 
 
 BW.06.5  122.0 
     73.713         nan 
     32.640         nan 
      3.830         nan 
 
 BW.06.5  136.0 
     76.730         nan 
     34.790         nan 
      3.850         nan 
 
 BW.06.5  150.0 
     79.167         nan 
     37.000         nan 
      3.880         nan 
 
 BW.06.6  10.0 
     15.015         nan 
      6.510         nan 
      2.415         nan 
 
 BW.06.6  24.0 
     32.675         nan 
     12.410         nan 
      3.043         nan 
 
 BW.06.6  38.0 
     50.551         nan 
     17.510         nan 
      3.314         nan 
 
 BW.06.6  52.0 
     78.965         nan 
     21.870         nan 
      3.499         nan 
 
 BW.06.6  66.0 
    100.024         nan 
     25.570         nan 
      3.541         nan 
 
 BW.06.6  80.0 
    117.601         nan 
     29.610         nan 
      3.676         nan 
 
 BW.06.6  94.0 
    133.617         nan 
     32.610         nan 
      3.705         nan 
 
 BW.06.6  108.0 
    150.303         nan 
     35.980         nan 
      3.750         nan 
 
 BW.06.6  122.0 
    197.948         nan 
     40.270         nan 
      3.838         nan 
 
 BW.06.6  136.0 
    198.844         nan 
     43.580         nan 
      3.879         nan 
 
 BW.06.6  150.0 
    204.500         nan 
     47.000         nan 
      3.922         nan 
 
 BW.06.7  10.0 
     17.109         nan 
      7.060         nan 
      2.611         nan 
 
 BW.06.7  24.0 
     27.517         nan 
     12.710         nan 
      3.226         nan 
 
 BW.06.7  38.0 
     34.517         nan 
     16.950         nan 
      3.495         nan 
 
 BW.06.7  52.0 
     49.214         nan 
     21.520         nan 
      3.706         nan 
 
 BW.06.7  66.0 
     58.259         nan 
     24.440         nan 
      3.785         nan 
 
 BW.06.7  80.0 
     62.204         nan 
     27.510         nan 
      3.892         nan 
 
 BW.06.7  94.0 
     62.912         nan 
     30.060         nan 
      3.921         nan 
 
 BW.06.7  108.0 
     69.397         nan 
     33.160         nan 
      3.998         nan 
 
 BW.06.7  122.0 
     71.051         nan 
     35.340         nan 
      4.034         nan 
 
 BW.06.7  136.0 
     70.954         nan 
     37.700         nan 
      4.071         nan 
 
 BW.06.7  150.0 
     71.625         nan 
     40.000         nan 
      4.102         nan 
 
 BW.06.8  10.0 
     17.697         nan 
      6.920         nan 
      2.529         nan 
 
 BW.06.8  24.0 
     35.278         nan 
     13.070         nan 
      3.114         nan 
 
 BW.06.8  38.0 
     42.758         nan 
     18.260         nan 
      3.414         nan 
 
 BW.06.8  52.0 
     51.121         nan 
     22.530         nan 
      3.571         nan 
 
 BW.06.8  66.0 
     59.755         nan 
     26.780         nan 
      3.751         nan 
 
 BW.06.8  80.0 
     66.147         nan 
     30.290         nan 
      3.812         nan 
 
 BW.06.8  94.0 
     74.883         nan 
     33.390         nan 
      3.877         nan 
 
 BW.06.8  108.0 
     74.298         nan 
     35.940         nan 
      3.936         nan 
 
 BW.06.8  122.0 
     80.787         nan 
     38.690         nan 
      3.983         nan 
 
 BW.06.8  136.0 
     86.099         nan 
     41.290         nan 
      4.013         nan 
 
 BW.06.8  150.0 
     90.429         nan 
     44.000         nan 
      4.058         nan 
 
 BW.06.9  10.0 
     10.975         nan 
      5.260         nan 
      1.925         nan 
 
 BW.06.9  24.0 
     26.552         nan 
      9.800         nan 
      2.343         nan 
 
 BW.06.9  38.0 
     40.258         nan 
     14.350         nan 
      2.641         nan 
 
 BW.06.9  52.0 
     49.935         nan 
     17.810         nan 
      2.782         nan 
 
 BW.06.9  66.0 
     56.583         nan 
     21.000         nan 
      2.851         nan 
 
 BW.06.9  80.0 
     65.671         nan 
     24.500         nan 
      2.967         nan 
 
 BW.06.9  94.0 
     68.757         nan 
     27.310         nan 
      3.033         nan 
 
 BW.06.9  108.0 
     71.127         nan 
     29.500         nan 
      3.057         nan 
 
 BW.06.9  122.0 
     73.906         nan 
     32.400         nan 
      3.113         nan 
 
 BW.06.9  136.0 
     77.492         nan 
     34.820         nan 
      3.145         nan 
 
 BW.06.9  150.0 
     76.429         nan 
     37.000         nan 
      3.171         nan 
 
 BW.07.1  10.0 
      5.562         nan 
      4.170         nan 
      1.706         nan 
 
 BW.07.1  24.0 
      9.446         nan 
      6.120         nan 
      1.962         nan 
 
 BW.07.1  38.0 
     13.665         nan 
      8.140         nan 
      2.161         nan 
 
 BW.07.1  52.0 
     15.559         nan 
      9.320         nan 
      2.179         nan 
 
 BW.07.1  66.0 
     16.335         nan 
     10.490         nan 
      2.248         nan 
 
 BW.07.1  80.0 
     16.594         nan 
     11.650         nan 
      2.287         nan 
 
 BW.07.1  94.0 
     17.826         nan 
     12.460         nan 
      2.298         nan 
 
 BW.07.1  108.0 
     18.410         nan 
     13.380         nan 
      2.317         nan 
 
 BW.07.1  122.0 
     17.283         nan 
     13.960         nan 
      2.341         nan 
 
 BW.07.1  136.0 
     17.272         nan 
     14.530         nan 
      2.359         nan 
 
 BW.07.1  150.0 
     17.000         nan 
     15.000         nan 
      2.361         nan 
 
 BW.07.10  10.0 
      5.733         nan 
      3.780         nan 
      1.411         nan 
 
 BW.07.10  24.0 
     10.718         nan 
      6.170         nan 
      1.682         nan 
 
 BW.07.10  38.0 
     18.742         nan 
      8.390         nan 
      1.845         nan 
 
 BW.07.10  52.0 
     20.599         nan 
      9.690         nan 
      1.871         nan 
 
 BW.07.10  66.0 
     26.358         nan 
     11.590         nan 
      1.931         nan 
 
 BW.07.10  80.0 
     32.287         nan 
     13.420         nan 
      1.994         nan 
 
 BW.07.10  94.0 
     32.575         nan 
     14.450         nan 
      2.002         nan 
 
 BW.07.10  108.0 
     37.077         nan 
     15.810         nan 
      2.023         nan 
 
 BW.07.10  122.0 
     41.355         nan 
     17.140         nan 
      2.037         nan 
 
 BW.07.10  136.0 
     43.964         nan 
     18.990         nan 
      2.091         nan 
 
 BW.07.10  150.0 
     46.000         nan 
     20.000         nan 
      2.097         nan 
 
 BW.07.2  10.0 
      7.620         nan 
      5.340         nan 
      2.159         nan 
 
 BW.07.2  24.0 
     14.279         nan 
      8.500         nan 
      2.587         nan 
 
 BW.07.2  38.0 
     24.955         nan 
     11.230         nan 
      2.786         nan 
 
 BW.07.2  52.0 
     38.151         nan 
     13.770         nan 
      2.911         nan 
 
 BW.07.2  66.0 
     55.998         nan 
     16.080         nan 
      2.989         nan 
 
 BW.07.2  80.0 
     68.301         nan 
     18.120         nan 
      3.033         nan 
 
 BW.07.2  94.0 
     72.869         nan 
     20.110         nan 
      3.065         nan 
 
 BW.07.2  108.0 
     78.530         nan 
     22.400         nan 
      3.112         nan 
 
 BW.07.2  122.0 
     80.211         nan 
     24.260         nan 
      3.132         nan 
 
 BW.07.2  136.0 
     79.659         nan 
     26.180         nan 
      3.159         nan 
 
 BW.07.2  150.0 
     75.500         nan 
     28.000         nan 
      3.177         nan 
 
 BW.07.3  10.0 
      5.483         nan 
      3.820         nan 
      1.521         nan 
 
 BW.07.3  24.0 
     11.353         nan 
      6.570         nan 
      1.872         nan 
 
 BW.07.3  38.0 
     14.868         nan 
      8.290         nan 
      1.955         nan 
 
 BW.07.3  52.0 
     17.969         nan 
      9.840         nan 
      1.991         nan 
 
 BW.07.3  66.0 
     22.730         nan 
     11.900         nan 
      2.119         nan 
 
 BW.07.3  80.0 
     24.409         nan 
     12.770         nan 
      2.130         nan 
 
 BW.07.3  94.0 
     28.512         nan 
     13.930         nan 
      2.135         nan 
 
 BW.07.3  108.0 
     27.015         nan 
     14.990         nan 
      2.173         nan 
 
 BW.07.3  122.0 
     31.962         nan 
     16.060         nan 
      2.193         nan 
 
 BW.07.3  136.0 
     34.447         nan 
     16.950         nan 
      2.194         nan 
 
 BW.07.3  150.0 
     33.000         nan 
     18.000         nan 
      2.214         nan 
 
 BW.07.4  10.0 
      6.113         nan 
      4.410         nan 
      1.781         nan 
 
 BW.07.4  24.0 
     12.709         nan 
      7.140         nan 
      2.083         nan 
 
 BW.07.4  38.0 
     16.245         nan 
      9.160         nan 
      2.198         nan 
 
 BW.07.4  52.0 
     21.481         nan 
     11.590         nan 
      2.354         nan 
 
 BW.07.4  66.0 
     27.034         nan 
     13.040         nan 
      2.365         nan 
 
 BW.07.4  80.0 
     29.637         nan 
     14.830         nan 
      2.425         nan 
 
 BW.07.4  94.0 
     35.030         nan 
     16.260         nan 
      2.459         nan 
 
 BW.07.4  108.0 
     35.905         nan 
     17.290         nan 
      2.475         nan 
 
 BW.07.4  122.0 
     39.153         nan 
     18.560         nan 
      2.486         nan 
 
 BW.07.4  136.0 
     39.120         nan 
     19.710         nan 
      2.505         nan 
 
 BW.07.4  150.0 
     37.500         nan 
     21.000         nan 
      2.533         nan 
 
 BW.07.5  10.0 
      7.832         nan 
      4.600         nan 
      1.772         nan 
 
 BW.07.5  24.0 
     20.337         nan 
      8.260         nan 
      2.235         nan 
 
 BW.07.5  38.0 
     30.687         nan 
     11.330         nan 
      2.412         nan 
 
 BW.07.5  52.0 
     31.995         nan 
     13.500         nan 
      2.469         nan 
 
 BW.07.5  66.0 
     38.764         nan 
     15.820         nan 
      2.530         nan 
 
 BW.07.5  80.0 
     46.051         nan 
     18.580         nan 
      2.619         nan 
 
 BW.07.5  94.0 
     51.843         nan 
     20.270         nan 
      2.628         nan 
 
 BW.07.5  108.0 
     62.164         nan 
     22.530         nan 
      2.700         nan 
 
 BW.07.5  122.0 
     65.828         nan 
     24.260         nan 
      2.704         nan 
 
 BW.07.5  136.0 
     78.912         nan 
     26.210         nan 
      2.744         nan 
 
 BW.07.5  150.0 
     85.000         nan 
     28.000         nan 
      2.763         nan 
 
 BW.07.6  10.0 
      4.455         nan 
      3.540         nan 
      1.475         nan 
 
 BW.07.6  24.0 
     10.910         nan 
      5.680         nan 
      1.695         nan 
 
 BW.07.6  38.0 
     19.685         nan 
      7.650         nan 
      1.803         nan 
 
 BW.07.6  52.0 
     29.402         nan 
      9.730         nan 
      1.889         nan 
 
 BW.07.6  66.0 
     33.100         nan 
     11.530         nan 
      1.944         nan 
 
 BW.07.6  80.0 
     41.008         nan 
     13.480         nan 
      2.000         nan 
 
 BW.07.6  94.0 
     39.227         nan 
     14.600         nan 
      1.988         nan 
 
 BW.07.6  108.0 
     42.460         nan 
     16.440         nan 
      2.025         nan 
 
 BW.07.6  122.0 
     47.444         nan 
     18.040         nan 
      2.060         nan 
 
 BW.07.6  136.0 
     46.824         nan 
     19.570         nan 
      2.078         nan 
 
 BW.07.6  150.0 
     47.250         nan 
     21.000         nan 
      2.097         nan 
 
 BW.07.7  10.0 
      4.982         nan 
      3.890         nan 
      1.605         nan 
 
 BW.07.7  24.0 
     11.891         nan 
      6.500         nan 
      1.948         nan 
 
 BW.07.7  38.0 
     19.286         nan 
      8.620         nan 
      2.079         nan 
 
 BW.07.7  52.0 
     23.233         nan 
     10.300         nan 
      2.130         nan 
 
 BW.07.7  66.0 
     29.485         nan 
     12.110         nan 
      2.184         nan 
 
 BW.07.7  80.0 
     36.316         nan 
     13.760         nan 
      2.249         nan 
 
 BW.07.7  94.0 
     42.967         nan 
     15.400         nan 
      2.272         nan 
 
 BW.07.7  108.0 
     44.218         nan 
     17.050         nan 
      2.306         nan 
 
 BW.07.7  122.0 
     47.101         nan 
     18.460         nan 
      2.321         nan 
 
 BW.07.7  136.0 
     44.197         nan 
     19.600         nan 
      2.328         nan 
 
 BW.07.7  150.0 
     43.750         nan 
     21.000         nan 
      2.347         nan 
 
 BW.07.8  10.0 
      4.038         nan 
      3.420         nan 
      1.418         nan 
 
 BW.07.8  24.0 
      7.398         nan 
      5.050         nan 
      1.626         nan 
 
 BW.07.8  38.0 
     10.110         nan 
      6.570         nan 
      1.800         nan 
 
 BW.07.8  52.0 
     11.784         nan 
      7.620         nan 
      1.836         nan 
 
 BW.07.8  66.0 
     15.523         nan 
      8.770         nan 
      1.863         nan 
 
 BW.07.8  80.0 
     19.188         nan 
      9.720         nan 
      1.891         nan 
 
 BW.07.8  94.0 
     20.268         nan 
     10.560         nan 
      1.910         nan 
 
 BW.07.8  108.0 
     22.808         nan 
     11.640         nan 
      1.947         nan 
 
 BW.07.8  122.0 
     25.982         nan 
     12.610         nan 
      1.965         nan 
 
 BW.07.8  136.0 
     23.697         nan 
     13.200         nan 
      1.969         nan 
 
 BW.07.8  150.0 
     23.333         nan 
     14.000         nan 
      1.981         nan 
 
 BW.07.9  10.0 
      6.395         nan 
      4.160         nan 
      1.604         nan 
 
 BW.07.9  24.0 
     15.252         nan 
      7.420         nan 
      2.036         nan 
 
 BW.07.9  38.0 
     20.322         nan 
      9.640         nan 
      2.145         nan 
 
 BW.07.9  52.0 
     22.636         nan 
     11.910         nan 
      2.263         nan 
 
 BW.07.9  66.0 
     26.317         nan 
     13.920         nan 
      2.346         nan 
 
 BW.07.9  80.0 
     25.749         nan 
     15.140         nan 
      2.365         nan 
 
 BW.07.9  94.0 
     28.179         nan 
     16.170         nan 
      2.372         nan 
 
 BW.07.9  108.0 
     31.669         nan 
     17.550         nan 
      2.398         nan 
 
 BW.07.9  122.0 
     35.502         nan 
     18.930         nan 
      2.438         nan 
 
 BW.07.9  136.0 
     38.931         nan 
     19.950         nan 
      2.450         nan 
 
 BW.07.9  150.0 
     39.333         nan 
     21.000         nan 
      2.468         nan 
 
 BW.08.1  10.0 
     17.117         nan 
      7.100         nan 
      2.602         nan 
 
 BW.08.1  24.0 
     30.468         nan 
     13.190         nan 
      3.206         nan 
 
 BW.08.1  38.0 
     40.992         nan 
     18.070         nan 
      3.500         nan 
 
 BW.08.1  52.0 
     46.916         nan 
     21.720         nan 
      3.640         nan 
 
 BW.08.1  66.0 
     54.114         nan 
     25.580         nan 
      3.776         nan 
 
 BW.08.1  80.0 
     54.584         nan 
     28.370         nan 
      3.866         nan 
 
 BW.08.1  94.0 
     63.095         nan 
     31.010         nan 
      3.927         nan 
 
 BW.08.1  108.0 
     63.488         nan 
     33.360         nan 
      3.966         nan 
 
 BW.08.1  122.0 
     63.452         nan 
     35.780         nan 
      4.023         nan 
 
 BW.08.1  136.0 
     66.363         nan 
     37.950         nan 
      4.056         nan 
 
 BW.08.1  150.0 
     66.250         nan 
     40.000         nan 
      4.087         nan 
 
 BW.08.10  10.0 
      9.188         nan 
      4.860         nan 
      1.798         nan 
 
 BW.08.10  24.0 
     16.998         nan 
      8.590         nan 
      2.195         nan 
 
 BW.08.10  38.0 
     23.071         nan 
     12.060         nan 
      2.475         nan 
 
 BW.08.10  52.0 
     27.473         nan 
     14.610         nan 
      2.550         nan 
 
 BW.08.10  66.0 
     34.130         nan 
     16.790         nan 
      2.653         nan 
 
 BW.08.10  80.0 
     32.725         nan 
     18.340         nan 
      2.667         nan 
 
 BW.08.10  94.0 
     35.708         nan 
     20.110         nan 
      2.716         nan 
 
 BW.08.10  108.0 
     39.182         nan 
     21.770         nan 
      2.751         nan 
 
 BW.08.10  122.0 
     44.372         nan 
     23.240         nan 
      2.762         nan 
 
 BW.08.10  136.0 
     45.137         nan 
     24.640         nan 
      2.787         nan 
 
 BW.08.10  150.0 
     48.750         nan 
     26.000         nan 
      2.805         nan 
 
 BW.08.2  10.0 
     15.145         nan 
      7.140         nan 
      2.667         nan 
 
 BW.08.2  24.0 
     24.404         nan 
     13.150         nan 
      3.417         nan 
 
 BW.08.2  38.0 
     30.896         nan 
     16.680         nan 
      3.611         nan 
 
 BW.08.2  52.0 
     32.296         nan 
     19.710         nan 
      3.770         nan 
 
 BW.08.2  66.0 
     39.675         nan 
     22.690         nan 
      3.864         nan 
 
 BW.08.2  80.0 
     42.645         nan 
     24.470         nan 
      3.925         nan 
 
 BW.08.2  94.0 
     41.256         nan 
     26.210         nan 
      3.966         nan 
 
 BW.08.2  108.0 
     46.421         nan 
     28.600         nan 
      4.026         nan 
 
 BW.08.2  122.0 
     45.155         nan 
     30.220         nan 
      4.065         nan 
 
 BW.08.2  136.0 
     44.938         nan 
     31.590         nan 
      4.088         nan 
 
 BW.08.2  150.0 
     44.375         nan 
     33.000         nan 
      4.113         nan 
 
 BW.08.3  10.0 
     16.553         nan 
      7.380         nan 
      2.714         nan 
 
 BW.08.3  24.0 
     30.223         nan 
     13.790         nan 
      3.473         nan 
 
 BW.08.3  38.0 
     46.002         nan 
     18.350         nan 
      3.733         nan 
 
 BW.08.3  52.0 
     58.554         nan 
     22.270         nan 
      3.885         nan 
 
 BW.08.3  66.0 
     74.299         nan 
     26.090         nan 
      4.029         nan 
 
 BW.08.3  80.0 
     84.936         nan 
     28.970         nan 
      4.081         nan 
 
 BW.08.3  94.0 
     91.991         nan 
     32.310         nan 
      4.146         nan 
 
 BW.08.3  108.0 
    104.275         nan 
     35.730         nan 
      4.229         nan 
 
 BW.08.3  122.0 
    107.468         nan 
     38.650         nan 
      4.268         nan 
 
 BW.08.3  136.0 
    109.182         nan 
     41.340         nan 
      4.304         nan 
 
 BW.08.3  150.0 
    111.667         nan 
     44.000         nan 
      4.336         nan 
 
 BW.08.4  10.0 
     18.070         nan 
      7.120         nan 
      2.592         nan 
 
 BW.08.4  24.0 
     37.853         nan 
     13.810         nan 
      3.298         nan 
 
 BW.08.4  38.0 
     44.224         nan 
     18.880         nan 
      3.557         nan 
 
 BW.08.4  52.0 
     52.066         nan 
     23.700         nan 
      3.767         nan 
 
 BW.08.4  66.0 
     53.714         nan 
     27.450         nan 
      3.918         nan 
 
 BW.08.4  80.0 
     58.422         nan 
     30.760         nan 
      4.008         nan 
 
 BW.08.4  94.0 
     66.659         nan 
     33.790         nan 
      4.058         nan 
 
 BW.08.4  108.0 
     68.659         nan 
     36.370         nan 
      4.110         nan 
 
 BW.08.4  122.0 
     72.622         nan 
     39.060         nan 
      4.151         nan 
 
 BW.08.4  136.0 
     75.502         nan 
     41.730         nan 
      4.206         nan 
 
 BW.08.4  150.0 
     74.667         nan 
     44.000         nan 
      4.238         nan 
 
 BW.08.5  10.0 
     10.461         nan 
      6.120         nan 
      2.385         nan 
 
 BW.08.5  24.0 
     24.500         nan 
     10.770         nan 
      2.961         nan 
 
 BW.08.5  38.0 
     43.676         nan 
     14.490         nan 
      3.177         nan 
 
 BW.08.5  52.0 
     65.349         nan 
     17.860         nan 
      3.299         nan 
 
 BW.08.5  66.0 
     74.047         nan 
     21.190         nan 
      3.411         nan 
 
 BW.08.5  80.0 
     87.627         nan 
     24.170         nan 
      3.468         nan 
 
 BW.08.5  94.0 
     89.062         nan 
     26.700         nan 
      3.509         nan 
 
 BW.08.5  108.0 
     90.492         nan 
     30.100         nan 
      3.595         nan 
 
 BW.08.5  122.0 
     96.998         nan 
     32.700         nan 
      3.615         nan 
 
 BW.08.5  136.0 
     97.970         nan 
     35.680         nan 
      3.671         nan 
 
 BW.08.5  150.0 
     96.500         nan 
     38.000         nan 
      3.692         nan 
 
 BW.08.6  10.0 
      3.740         nan 
      2.710         nan 
      0.814         nan 
 
 BW.08.6  24.0 
      8.182         nan 
      4.820         nan 
      1.075         nan 
 
 BW.08.6  38.0 
     12.676         nan 
      6.560         nan 
      1.149         nan 
 
 BW.08.6  52.0 
     14.993         nan 
      7.900         nan 
      1.209         nan 
 
 BW.08.6  66.0 
     18.054         nan 
      8.930         nan 
      1.213         nan 
 
 BW.08.6  80.0 
     21.488         nan 
     10.100         nan 
      1.235         nan 
 
 BW.08.6  94.0 
     23.032         nan 
     10.980         nan 
      1.253         nan 
 
 BW.08.6  108.0 
     28.791         nan 
     12.040         nan 
      1.283         nan 
 
 BW.08.6  122.0 
     30.985         nan 
     13.010         nan 
      1.290         nan 
 
 BW.08.6  136.0 
     35.042         nan 
     14.080         nan 
      1.299         nan 
 
 BW.08.6  150.0 
     37.500         nan 
     15.000         nan 
      1.312         nan 
 
 BW.08.7  10.0 
      5.038         nan 
      3.300         nan 
      1.099         nan 
 
 BW.08.7  24.0 
      9.440         nan 
      5.540         nan 
      1.310         nan 
 
 BW.08.7  38.0 
     14.648         nan 
      7.790         nan 
      1.476         nan 
 
 BW.08.7  52.0 
     16.232         nan 
      9.040         nan 
      1.472         nan 
 
 BW.08.7  66.0 
     19.179         nan 
     10.230         nan 
      1.514         nan 
 
 BW.08.7  80.0 
     23.176         nan 
     11.600         nan 
      1.551         nan 
 
 BW.08.7  94.0 
     29.059         nan 
     13.140         nan 
      1.631         nan 
 
 BW.08.7  108.0 
     34.521         nan 
     13.990         nan 
      1.629         nan 
 
 BW.08.7  122.0 
     39.833         nan 
     14.800         nan 
      1.634         nan 
 
 BW.08.7  136.0 
     52.498         nan 
     16.100         nan 
      1.657         nan 
 
 BW.08.7  150.0 
     72.000         nan 
     17.000         nan 
      1.661         nan 
 
 BW.08.8  10.0 
      4.420         nan 
      3.070         nan 
      0.988         nan 
 
 BW.08.8  24.0 
      9.512         nan 
      5.320         nan 
      1.252         nan 
 
 BW.08.8  38.0 
     13.267         nan 
      7.420         nan 
      1.411         nan 
 
 BW.08.8  52.0 
     17.756         nan 
      9.000         nan 
      1.482         nan 
 
 BW.08.8  66.0 
     21.848         nan 
     10.400         nan 
      1.521         nan 
 
 BW.08.8  80.0 
     23.753         nan 
     11.780         nan 
      1.577         nan 
 
 BW.08.8  94.0 
     26.138         nan 
     13.020         nan 
      1.598         nan 
 
 BW.08.8  108.0 
     27.015         nan 
     13.920         nan 
      1.599         nan 
 
 BW.08.8  122.0 
     30.163         nan 
     15.180         nan 
      1.631         nan 
 
 BW.08.8  136.0 
     29.725         nan 
     15.970         nan 
      1.628         nan 
 
 BW.08.8  150.0 
     32.000         nan 
     17.000         nan 
      1.648         nan 
 
 BW.08.9  10.0 
      8.948         nan 
      4.870         nan 
      1.810         nan 
 
 BW.08.9  24.0 
     16.279         nan 
      8.750         nan 
      2.300         nan 
 
 BW.08.9  38.0 
     22.116         nan 
     11.300         nan 
      2.398         nan 
 
 BW.08.9  52.0 
     25.757         nan 
     13.810         nan 
      2.539         nan 
 
 BW.08.9  66.0 
     30.626         nan 
     15.840         nan 
      2.597         nan 
 
 BW.08.9  80.0 
     33.467         nan 
     17.940         nan 
      2.663         nan 
 
 BW.08.9  94.0 
     34.869         nan 
     19.390         nan 
      2.688         nan 
 
 BW.08.9  108.0 
     37.808         nan 
     20.970         nan 
      2.732         nan 
 
 BW.08.9  122.0 
     39.360         nan 
     22.460         nan 
      2.770         nan 
 
 BW.08.9  136.0 
     40.891         nan 
     23.810         nan 
      2.790         nan 
 
 BW.08.9  150.0 
     40.600         nan 
     25.000         nan 
      2.804         nan 
 
 BW.09.1  10.0 
     16.725         nan 
      6.580         nan 
      2.393         nan 
 
 BW.09.1  24.0 
     50.995         nan 
     13.540         nan 
      3.101         nan 
 
 BW.09.1  38.0 
     69.644         nan 
     19.370         nan 
      3.386         nan 
 
 BW.09.1  52.0 
     97.193         nan 
     24.970         nan 
      3.601         nan 
 
 BW.09.1  66.0 
    100.785         nan 
     29.550         nan 
      3.721         nan 
 
 BW.09.1  80.0 
    119.073         nan 
     35.000         nan 
      3.861         nan 
 
 BW.09.1  94.0 
    122.575         nan 
     38.570         nan 
      3.886         nan 
 
 BW.09.1  108.0 
    115.358         nan 
     42.610         nan 
      3.946         nan 
 
 BW.09.1  122.0 
    120.537         nan 
     47.240         nan 
      4.055         nan 
 
 BW.09.1  136.0 
    123.891         nan 
     51.300         nan 
      4.105         nan 
 
 BW.09.1  150.0 
    122.364         nan 
     55.000         nan 
      4.143         nan 
 
 BW.09.10  10.0 
      9.837         nan 
      5.830         nan 
      2.298         nan 
 
 BW.09.10  24.0 
     22.681         nan 
     10.180         nan 
      2.845         nan 
 
 BW.09.10  38.0 
     31.996         nan 
     12.980         nan 
      2.963         nan 
 
 BW.09.10  52.0 
     39.077         nan 
     16.240         nan 
      3.119         nan 
 
 BW.09.10  66.0 
     43.285         nan 
     18.880         nan 
      3.217         nan 
 
 BW.09.10  80.0 
     49.744         nan 
     21.230         nan 
      3.261         nan 
 
 BW.09.10  94.0 
     53.213         nan 
     23.750         nan 
      3.324         nan 
 
 BW.09.10  108.0 
     54.041         nan 
     25.330         nan 
      3.332         nan 
 
 BW.09.10  122.0 
     52.554         nan 
     27.320         nan 
      3.376         nan 
 
 BW.09.10  136.0 
     53.978         nan 
     29.270         nan 
      3.409         nan 
 
 BW.09.10  150.0 
     52.857         nan 
     31.000         nan 
      3.430         nan 
 
 BW.09.2  10.0 
      5.420         nan 
      3.490         nan 
      1.214         nan 
 
 BW.09.2  24.0 
     10.704         nan 
      6.180         nan 
      1.559         nan 
 
 BW.09.2  38.0 
     16.741         nan 
      8.150         nan 
      1.691         nan 
 
 BW.09.2  52.0 
     23.415         nan 
      9.720         nan 
      1.708         nan 
 
 BW.09.2  66.0 
     27.953         nan 
     11.350         nan 
      1.765         nan 
 
 BW.09.2  80.0 
     32.678         nan 
     12.940         nan 
      1.798         nan 
 
 BW.09.2  94.0 
     33.210         nan 
     14.200         nan 
      1.827         nan 
 
 BW.09.2  108.0 
     30.402         nan 
     15.560         nan 
      1.852         nan 
 
 BW.09.2  122.0 
     30.025         nan 
     17.030         nan 
      1.899         nan 
 
 BW.09.2  136.0 
     28.215         nan 
     17.850         nan 
      1.896         nan 
 
 BW.09.2  150.0 
     28.167         nan 
     19.000         nan 
      1.909         nan 
 
 BW.09.3  10.0 
     13.837         nan 
      6.730         nan 
      2.534         nan 
 
 BW.09.3  24.0 
     26.159         nan 
     11.780         nan 
      3.087         nan 
 
 BW.09.3  38.0 
     44.390         nan 
     15.860         nan 
      3.318         nan 
 
 BW.09.3  52.0 
     56.828         nan 
     19.550         nan 
      3.472         nan 
 
 BW.09.3  66.0 
     66.855         nan 
     22.860         nan 
      3.591         nan 
 
 BW.09.3  80.0 
     86.093         nan 
     25.650         nan 
      3.630         nan 
 
 BW.09.3  94.0 
    108.871         nan 
     29.210         nan 
      3.725         nan 
 
 BW.09.3  108.0 
    125.489         nan 
     32.720         nan 
      3.814         nan 
 
 BW.09.3  122.0 
    152.663         nan 
     35.340         nan 
      3.837         nan 
 
 BW.09.3  136.0 
    153.102         nan 
     38.210         nan 
      3.871         nan 
 
 BW.09.3  150.0 
    149.750         nan 
     41.000         nan 
      3.903         nan 
 
 BW.09.4  10.0 
      9.366         nan 
      5.910         nan 
      2.331         nan 
 
 BW.09.4  24.0 
     18.758         nan 
      9.440         nan 
      2.739         nan 
 
 BW.09.4  38.0 
     33.757         nan 
     12.960         nan 
      3.003         nan 
 
 BW.09.4  52.0 
     39.204         nan 
     15.120         nan 
      3.063         nan 
 
 BW.09.4  66.0 
     52.306         nan 
     17.760         nan 
      3.136         nan 
 
 BW.09.4  80.0 
     60.237         nan 
     20.180         nan 
      3.201         nan 
 
 BW.09.4  94.0 
     61.613         nan 
     22.330         nan 
      3.247         nan 
 
 BW.09.4  108.0 
     64.451         nan 
     24.330         nan 
      3.281         nan 
 
 BW.09.4  122.0 
     73.414         nan 
     27.240         nan 
      3.352         nan 
 
 BW.09.4  136.0 
     82.167         nan 
     29.130         nan 
      3.369         nan 
 
 BW.09.4  150.0 
     83.500         nan 
     31.000         nan 
      3.390         nan 
 
 BW.09.5  10.0 
      9.638         nan 
      5.190         nan 
      1.972         nan 
 
 BW.09.5  24.0 
     20.556         nan 
      9.290         nan 
      2.443         nan 
 
 BW.09.5  38.0 
     32.317         nan 
     12.540         nan 
      2.623         nan 
 
 BW.09.5  52.0 
     42.125         nan 
     15.560         nan 
      2.764         nan 
 
 BW.09.5  66.0 
     43.060         nan 
     18.200         nan 
      2.847         nan 
 
 BW.09.5  80.0 
     55.389         nan 
     20.430         nan 
      2.869         nan 
 
 BW.09.5  94.0 
     62.730         nan 
     22.630         nan 
      2.909         nan 
 
 BW.09.5  108.0 
     69.912         nan 
     24.830         nan 
      2.955         nan 
 
 BW.09.5  122.0 
     79.295         nan 
     27.140         nan 
      2.991         nan 
 
 BW.09.5  136.0 
     80.463         nan 
     29.030         nan 
      3.015         nan 
 
 BW.09.5  150.0 
     83.500         nan 
     31.000         nan 
      3.040         nan 
 
 BW.09.6  10.0 
     15.749         nan 
      7.160         nan 
      2.659         nan 
 
 BW.09.6  24.0 
     33.478         nan 
     13.160         nan 
      3.346         nan 
 
 BW.09.6  38.0 
     54.986         nan 
     18.020         nan 
      3.626         nan 
 
 BW.09.6  52.0 
     71.788         nan 
     22.400         nan 
      3.791         nan 
 
 BW.09.6  66.0 
     83.261         nan 
     26.730         nan 
      3.939         nan 
 
 BW.09.6  80.0 
     86.167         nan 
     30.470         nan 
      4.021         nan 
 
 BW.09.6  94.0 
     99.409         nan 
     34.050         nan 
      4.102         nan 
 
 BW.09.6  108.0 
     98.305         nan 
     37.910         nan 
      4.178         nan 
 
 BW.09.6  122.0 
     97.411         nan 
     40.860         nan 
      4.213         nan 
 
 BW.09.6  136.0 
     99.605         nan 
     44.000         nan 
      4.263         nan 
 
 BW.09.6  150.0 
     98.667         nan 
     47.000         nan 
      4.300         nan 
 
 BW.09.7  10.0 
     10.075         nan 
      5.190         nan 
      1.932         nan 
 
 BW.09.7  24.0 
     22.313         nan 
      9.420         nan 
      2.384         nan 
 
 BW.09.7  38.0 
     34.067         nan 
     13.010         nan 
      2.612         nan 
 
 BW.09.7  52.0 
     41.807         nan 
     16.040         nan 
      2.698         nan 
 
 BW.09.7  66.0 
     43.242         nan 
     19.060         nan 
      2.835         nan 
 
 BW.09.7  80.0 
     54.225         nan 
     21.780         nan 
      2.883         nan 
 
 BW.09.7  94.0 
     51.508         nan 
     23.880         nan 
      2.908         nan 
 
 BW.09.7  108.0 
     49.886         nan 
     26.300         nan 
      2.972         nan 
 
 BW.09.7  122.0 
     50.173         nan 
     28.340         nan 
      2.999         nan 
 
 BW.09.7  136.0 
     49.890         nan 
     30.240         nan 
      3.033         nan 
 
 BW.09.7  150.0 
     49.000         nan 
     32.000         nan 
      3.055         nan 
 
 BW.09.8  10.0 
      9.290         nan 
      4.850         nan 
      1.785         nan 
 
 BW.09.8  24.0 
     18.949         nan 
      8.920         nan 
      2.224         nan 
 
 BW.09.8  38.0 
     22.768         nan 
     11.620         nan 
      2.352         nan 
 
 BW.09.8  52.0 
     29.558         nan 
     14.240         nan 
      2.488         nan 
 
 BW.09.8  66.0 
     34.018         nan 
     16.880         nan 
      2.614         nan 
 
 BW.09.8  80.0 
     38.558         nan 
     18.210         nan 
      2.585         nan 
 
 BW.09.8  94.0 
     46.400         nan 
     20.540         nan 
      2.677         nan 
 
 BW.09.8  108.0 
     61.756         nan 
     22.230         nan 
      2.709         nan 
 
 BW.09.8  122.0 
     68.864         nan 
     23.710         nan 
      2.720         nan 
 
 BW.09.8  136.0 
     70.215         nan 
     25.340         nan 
      2.743         nan 
 
 BW.09.8  150.0 
     72.333         nan 
     27.000         nan 
      2.770         nan 
 
 BW.09.9  10.0 
      5.642         nan 
      4.140         nan 
      1.695         nan 
 
 BW.09.9  24.0 
     16.373         nan 
      7.070         nan 
      2.031         nan 
 
 BW.09.9  38.0 
     21.635         nan 
      9.190         nan 
      2.129         nan 
 
 BW.09.9  52.0 
     27.839         nan 
     11.380         nan 
      2.226         nan 
 
 BW.09.9  66.0 
     36.165         nan 
     13.400         nan 
      2.255         nan 
 
 BW.09.9  80.0 
     43.560         nan 
     15.460         nan 
      2.316         nan 
 
 BW.09.9  94.0 
     53.736         nan 
     16.860         nan 
      2.316         nan 
 
 BW.09.9  108.0 
     56.440         nan 
     19.090         nan 
      2.380         nan 
 
 BW.09.9  122.0 
     59.057         nan 
     20.570         nan 
      2.400         nan 
 
 BW.09.9  136.0 
     60.434         nan 
     22.490         nan 
      2.429         nan 
 
 BW.09.9  150.0 
     58.000         nan 
     24.000         nan 
      2.446         nan 
 
 BW.10.1  10.0 
      3.325         nan 
      2.580         nan 
      0.771         nan 
 
 BW.10.1  24.0 
      7.463         nan 
      4.670         nan 
      1.082         nan 
 
 BW.10.1  38.0 
     10.517         nan 
      6.240         nan 
      1.159         nan 
 
 BW.10.1  52.0 
     12.146         nan 
      7.180         nan 
      1.175         nan 
 
 BW.10.1  66.0 
     15.303         nan 
      8.320         nan 
      1.211         nan 
 
 BW.10.1  80.0 
     15.821         nan 
      9.070         nan 
      1.215         nan 
 
 BW.10.1  94.0 
     17.435         nan 
     10.040         nan 
      1.248         nan 
 
 BW.10.1  108.0 
     19.300         nan 
     10.890         nan 
      1.253         nan 
 
 BW.10.1  122.0 
     19.566         nan 
     11.640         nan 
      1.274         nan 
 
 BW.10.1  136.0 
     19.415         nan 
     12.280         nan 
      1.278         nan 
 
 BW.10.1  150.0 
     20.000         nan 
     13.000         nan 
      1.290         nan 
 
 BW.10.10  10.0 
      3.975         nan 
      3.180         nan 
      1.167         nan 
 
 BW.10.10  24.0 
      7.235         nan 
      4.830         nan 
      1.429         nan 
 
 BW.10.10  38.0 
     11.448         nan 
      6.370         nan 
      1.480         nan 
 
 BW.10.10  52.0 
     17.420         nan 
      7.790         nan 
      1.547         nan 
 
 BW.10.10  66.0 
     22.538         nan 
      8.920         nan 
      1.592         nan 
 
 BW.10.10  80.0 
     27.545         nan 
     10.150         nan 
      1.609         nan 
 
 BW.10.10  94.0 
     33.805         nan 
     11.200         nan 
      1.631         nan 
 
 BW.10.10  108.0 
     42.050         nan 
     12.670         nan 
      1.663         nan 
 
 BW.10.10  122.0 
     45.340         nan 
     13.750         nan 
      1.684         nan 
 
 BW.10.10  136.0 
     48.100         nan 
     14.780         nan 
      1.684         nan 
 
 BW.10.10  150.0 
     49.000         nan 
     16.000         nan 
      1.703         nan 
 
 BW.10.2  10.0 
     10.530         nan 
      5.760         nan 
      2.222         nan 
 
 BW.10.2  24.0 
     26.426         nan 
     10.980         nan 
      2.888         nan 
 
 BW.10.2  38.0 
     35.947         nan 
     14.570         nan 
      3.055         nan 
 
 BW.10.2  52.0 
     40.835         nan 
     17.630         nan 
      3.127         nan 
 
 BW.10.2  66.0 
     52.985         nan 
     20.910         nan 
      3.242         nan 
 
 BW.10.2  80.0 
     53.809         nan 
     23.810         nan 
      3.334         nan 
 
 BW.10.2  94.0 
     59.119         nan 
     26.260         nan 
      3.383         nan 
 
 BW.10.2  108.0 
     62.480         nan 
     28.690         nan 
      3.433         nan 
 
 BW.10.2  122.0 
     65.794         nan 
     30.960         nan 
      3.476         nan 
 
 BW.10.2  136.0 
     65.461         nan 
     33.020         nan 
      3.509         nan 
 
 BW.10.2  150.0 
     70.000         nan 
     35.000         nan 
      3.530         nan 
 
 BW.10.4  10.0 
     18.198         nan 
      7.470         nan 
      2.725         nan 
 
 BW.10.4  24.0 
     45.545         nan 
     15.170         nan 
      3.585         nan 
 
 BW.10.4  38.0 
     62.332         nan 
     20.810         nan 
      3.866         nan 
 
 BW.10.4  52.0 
     72.235         nan 
     26.050         nan 
      4.075         nan 
 
 BW.10.4  66.0 
     81.436         nan 
     31.240         nan 
      4.237         nan 
 
 BW.10.4  80.0 
     81.537         nan 
     35.000         nan 
      4.300         nan 
 
 BW.10.4  94.0 
     80.250         nan 
     38.570         nan 
      4.378         nan 
 
 BW.10.4  108.0 
     84.923         nan 
     42.890         nan 
      4.478         nan 
 
 BW.10.4  122.0 
     86.030         nan 
     45.900         nan 
      4.514         nan 
 
 BW.10.4  136.0 
     87.639         nan 
     49.270         nan 
      4.586         nan 
 
 BW.10.4  150.0 
     88.250         nan 
     52.000         nan 
      4.616         nan 
 
 BW.10.5  10.0 
      7.715         nan 
      4.170         nan 
      1.463         nan 
 
 BW.10.5  24.0 
     17.764         nan 
      7.570         nan 
      1.745         nan 
 
 BW.10.5  38.0 
     23.618         nan 
     10.520         nan 
      1.917         nan 
 
 BW.10.5  52.0 
     33.261         nan 
     13.680         nan 
      2.094         nan 
 
 BW.10.5  66.0 
     40.427         nan 
     15.740         nan 
      2.116         nan 
 
 BW.10.5  80.0 
     41.407         nan 
     18.280         nan 
      2.206         nan 
 
 BW.10.5  94.0 
     42.536         nan 
     20.290         nan 
      2.242         nan 
 
 BW.10.5  108.0 
     44.664         nan 
     21.680         nan 
      2.259         nan 
 
 BW.10.5  122.0 
     45.459         nan 
     23.670         nan 
      2.301         nan 
 
 BW.10.5  136.0 
     44.560         nan 
     25.260         nan 
      2.312         nan 
 
 BW.10.5  150.0 
     44.143         nan 
     27.000         nan 
      2.348         nan 
 
 BW.10.6  10.0 
     16.005         nan 
      6.940         nan 
      2.563         nan 
 
 BW.10.6  24.0 
     28.140         nan 
     11.930         nan 
      3.066         nan 
 
 BW.10.6  38.0 
     46.149         nan 
     16.710         nan 
      3.401         nan 
 
 BW.10.6  52.0 
     59.738         nan 
     20.790         nan 
      3.574         nan 
 
 BW.10.6  66.0 
     63.117         nan 
     24.040         nan 
      3.666         nan 
 
 BW.10.6  80.0 
     70.025         nan 
     27.450         nan 
      3.752         nan 
 
 BW.10.6  94.0 
     72.530         nan 
     30.280         nan 
      3.786         nan 
 
 BW.10.6  108.0 
     72.255         nan 
     33.280         nan 
      3.854         nan 
 
 BW.10.6  122.0 
     74.439         nan 
     35.940         nan 
      3.905         nan 
 
 BW.10.6  136.0 
     74.763         nan 
     38.500         nan 
      3.937         nan 
 
 BW.10.6  150.0 
     74.333         nan 
     41.000         nan 
      3.973         nan 
 
 BW.10.8  10.0 
     12.578         nan 
      5.570         nan 
      2.031         nan 
 
 BW.10.8  24.0 
     27.152         nan 
     10.240         nan 
      2.507         nan 
 
 BW.10.8  38.0 
     49.429         nan 
     15.390         nan 
      2.857         nan 
 
 BW.10.8  52.0 
     53.899         nan 
     18.970         nan 
      2.993         nan 
 
 BW.10.8  66.0 
     60.759         nan 
     22.600         nan 
      3.105         nan 
 
 BW.10.8  80.0 
     62.174         nan 
     25.520         nan 
      3.125         nan 
 
 BW.10.8  94.0 
     66.270         nan 
     28.700         nan 
      3.200         nan 
 
 BW.10.8  108.0 
     63.573         nan 
     31.350         nan 
      3.273         nan 
 
 BW.10.8  122.0 
     67.983         nan 
     34.030         nan 
      3.306         nan 
 
 BW.10.8  136.0 
     68.543         nan 
     36.670         nan 
      3.361         nan 
 
 BW.10.8  150.0 
     73.500         nan 
     39.000         nan 
      3.392         nan 
 
 BW.10.9  10.0 
     11.172         nan 
      5.480         nan 
      2.040         nan 
 
 BW.10.9  24.0 
     25.721         nan 
     10.160         nan 
      2.490         nan 
 
 BW.10.9  38.0 
     32.253         nan 
     13.630         nan 
      2.670         nan 
 
 BW.10.9  52.0 
     38.169         nan 
     16.700         nan 
      2.816         nan 
 
 BW.10.9  66.0 
     43.764         nan 
     19.630         nan 
      2.941         nan 
 
 BW.10.9  80.0 
     51.016         nan 
     22.550         nan 
      3.033         nan 
 
 BW.10.9  94.0 
     56.273         nan 
     24.900         nan 
      3.038         nan 
 
 BW.10.9  108.0 
     64.305         nan 
     27.170         nan 
      3.113         nan 
 
 BW.10.9  122.0 
     75.305         nan 
     29.270         nan 
      3.140         nan 
 
 BW.10.9  136.0 
     87.391         nan 
     30.960         nan 
      3.148         nan 
 
 BW.10.9  150.0 
    103.000         nan 
     33.000         nan 
      3.178         nan 
 
  TimePoint  Seqs/Sample 
 chao1 Ave.  chao1 Err. 
 observed_species Ave.  observed_species Err. 
 shannon Ave.  shannon Err. 
 
 
 1  10.0 
     10.766       5.059 
      5.379       1.617 
      1.995       0.644 
 
 1  24.0 
     23.091      12.990 
      9.708       3.359 
      2.491       0.806 
 
 1  38.0 
     33.946      19.284 
     13.168       4.866 
      2.689       0.889 
 
 1  52.0 
     41.191      24.581 
     16.134       6.196 
      2.810       0.937 
 
 1  66.0 
     46.407      26.144 
     18.878       7.526 
      2.899       0.981 
 
 1  80.0 
     51.979      31.770 
     21.183       8.700 
      2.951       0.998 
 
 1  94.0 
     55.206      31.693 
     23.368       9.743 
      2.992       1.018 
 
 1  108.0 
     58.871      32.097 
     25.525      10.686 
      3.035       1.033 
 
 1  122.0 
     60.159      32.765 
     27.566      11.752 
      3.075       1.049 
 
 1  136.0 
     63.286      34.652 
     29.543      12.801 
      3.107       1.064 
 
 1  150.0 
     63.805      33.276 
     31.300      13.726 
      3.127       1.073 
 
 2  10.0 
      8.686       3.943 
      4.842       1.345 
      1.816       0.574 
 
 2  24.0 
     17.498       6.890 
      8.563       2.656 
      2.264       0.730 
 
 2  38.0 
     24.031       7.852 
     11.233       3.403 
      2.413       0.754 
 
 2  52.0 
     29.711       6.918 
     13.631       3.959 
      2.509       0.790 
 
 2  66.0 
     37.341      11.067 
     15.923       4.586 
      2.587       0.817 
 
 2  80.0 
     42.800      13.240 
     17.821       4.916 
      2.637       0.820 
 
 2  94.0 
     46.188      14.542 
     19.572       5.229 
      2.677       0.822 
 
 2  108.0 
     49.073      17.038 
     21.321       5.671 
      2.705       0.836 
 
 2  122.0 
     52.008      19.197 
     23.048       6.007 
      2.740       0.844 
 
 2  136.0 
     52.542      19.600 
     24.580       6.252 
      2.762       0.852 
 
 2  150.0 
     52.379      20.486 
     26.000       6.616 
      2.781       0.856 
 
 3  10.0 
     11.855       6.978 
      5.678       2.049 
      2.091       0.795 
 
 3  24.0 
     25.131      15.411 
     10.428       4.461 
      2.619       1.052 
 
 3  38.0 
     36.103      18.079 
     14.004       6.146 
      2.814       1.135 
 
 3  52.0 
     45.844      22.059 
     17.233       7.716 
      2.941       1.204 
 
 3  66.0 
     52.255      24.163 
     20.320       9.176 
      3.051       1.245 
 
 3  80.0 
     60.315      27.779 
     22.900      10.180 
      3.112       1.260 
 
 3  94.0 
     66.797      31.891 
     25.494      11.450 
      3.161       1.294 
 
 3  108.0 
     70.936      36.727 
     27.941      12.559 
      3.214       1.314 
 
 3  122.0 
     74.728      41.878 
     30.233      13.514 
      3.249       1.331 
 
 3  136.0 
     76.084      42.078 
     32.333      14.393 
      3.279       1.343 
 
 3  150.0 
     75.184      41.572 
     34.375      15.182 
      3.306       1.355 
 
 4  10.0 
     11.777       5.577 
      5.724       1.818 
      2.111       0.737 
 
 4  24.0 
     25.804      12.617 
     10.313       3.764 
      2.609       0.936 
 
 4  38.0 
     36.075      16.164 
     14.179       5.281 
      2.845       1.018 
 
 4  52.0 
     44.949      19.417 
     17.482       6.495 
      2.975       1.053 
 
 4  66.0 
     52.773      21.463 
     20.510       7.730 
      3.055       1.099 
 
 4  80.0 
     57.008      21.916 
     23.311       8.654 
      3.135       1.120 
 
 4  94.0 
     61.610      22.329 
     25.702       9.456 
      3.173       1.134 
 
 4  108.0 
     65.465      26.047 
     28.151      10.494 
      3.225       1.154 
 
 4  122.0 
     68.707      25.140 
     30.551      11.252 
      3.262       1.170 
 
 4  136.0 
     72.924      25.287 
     32.732      11.981 
      3.296       1.180 
 
 4  150.0 
     74.942      26.786 
     34.800      12.640 
      3.324       1.188 
 
 5  10.0 
      9.221       3.346 
      5.175       1.110 
      1.979       0.423 
 
 5  24.0 
     18.942       6.108 
      8.892       2.094 
      2.398       0.539 
 
 5  38.0 
     30.098      10.885 
     12.153       3.073 
      2.604       0.601 
 
 5  52.0 
     40.216      13.505 
     14.969       3.497 
      2.728       0.586 
 
 5  66.0 
     48.710      16.208 
     17.371       4.281 
      2.786       0.619 
 
 5  80.0 
     57.273      19.263 
     19.754       4.765 
      2.847       0.624 
 
 5  94.0 
     63.763      21.586 
     21.970       5.330 
      2.893       0.633 
 
 5  108.0 
     71.803      25.112 
     24.150       5.979 
      2.935       0.648 
 
 5  122.0 
     78.250      30.204 
     26.176       6.409 
      2.963       0.652 
 
 5  136.0 
     83.549      35.762 
     28.185       6.905 
      2.993       0.661 
 
 5  150.0 
     89.148      42.840 
     30.100       7.395 
      3.016       0.666 
 
 6  10.0 
     11.918       6.096 
      5.541       1.929 
      2.013       0.756 
 
 6  24.0 
     24.519      11.403 
     10.063       3.756 
      2.494       0.951 
 
 6  38.0 
     37.887      17.724 
     13.972       5.335 
      2.722       1.056 
 
 6  52.0 
     49.915      22.688 
     17.402       6.511 
      2.865       1.088 
 
 6  66.0 
     59.630      29.048 
     20.415       7.799 
      2.947       1.137 
 
 6  80.0 
     66.682      30.660 
     23.383       8.811 
      3.026       1.158 
 
 6  94.0 
     73.463      34.398 
     25.890       9.871 
      3.065       1.180 
 
 6  108.0 
     78.376      38.034 
     28.504      10.872 
      3.114       1.201 
 
 6  122.0 
     87.330      50.281 
     31.045      11.913 
      3.155       1.219 
 
 6  136.0 
     90.098      51.893 
     33.367      12.711 
      3.187       1.230 
 
 6  150.0 
     95.195      59.062 
     35.700      13.617 
      3.219       1.240 
 
 7  10.0 
     10.815       7.136 
      5.059       1.869 
      1.841       0.707 
 
 7  24.0 
     23.825      17.824 
      9.237       3.897 
      2.290       0.908 
 
 7  38.0 
     33.628      22.316 
     12.832       5.587 
      2.517       0.990 
 
 7  52.0 
     43.496      29.372 
     15.898       7.329 
      2.613       1.061 
 
 7  66.0 
     47.078      27.180 
     18.728       8.864 
      2.710       1.114 
 
 7  80.0 
     52.841      29.451 
     21.303      10.045 
      2.776       1.125 
 
 7  94.0 
     55.148      28.720 
     23.701      11.353 
      2.826       1.151 
 
 7  108.0 
     57.708      29.103 
     26.064      12.729 
      2.875       1.187 
 
 7  122.0 
     60.427      29.378 
     27.977      13.733 
      2.897       1.196 
 
 7  136.0 
     62.280      29.611 
     30.016      14.729 
      2.929       1.213 
 
 7  150.0 
     65.202      30.943 
     31.889      15.609 
      2.953       1.221 
 
 8  10.0 
     12.070       6.789 
      5.465       1.781 
      1.995       0.657 
 
 8  24.0 
     26.292      16.136 
     10.158       3.981 
      2.489       0.864 
 
 8  38.0 
     37.811      25.862 
     14.084       5.868 
      2.722       0.950 
 
 8  52.0 
     48.746      36.400 
     17.494       7.667 
      2.864       1.012 
 
 8  66.0 
     54.060      36.925 
     20.582       9.183 
      2.964       1.050 
 
 8  80.0 
     59.941      41.112 
     23.360      10.744 
      3.025       1.084 
 
 8  94.0 
     67.182      47.804 
     26.046      12.349 
      3.084       1.114 
 
 8  108.0 
     69.802      46.935 
     28.382      13.674 
      3.132       1.139 
 
 8  122.0 
     75.284      49.892 
     30.757      15.018 
      3.169       1.155 
 
 8  136.0 
     77.740      51.602 
     32.893      16.261 
      3.197       1.172 
 
 8  150.0 
     81.746      54.353 
     35.100      17.575 
      3.229       1.188 
 
 9  10.0 
      9.649       5.991 
      4.884       1.669 
      1.803       0.664 
 
 9  24.0 
     21.203      10.998 
      8.851       3.686 
      2.230       0.874 
 
 9  38.0 
     30.209      15.540 
     12.029       5.185 
      2.405       0.946 
 
 9  52.0 
     36.972      17.707 
     14.953       6.529 
      2.537       1.003 
 
 9  66.0 
     42.663      18.843 
     17.471       7.539 
      2.608       1.033 
 
 9  80.0 
     47.491      21.840 
     19.868       8.616 
      2.674       1.061 
 
 9  94.0 
     52.045      22.858 
     21.979       9.623 
      2.708       1.085 
 
 9  108.0 
     55.400      23.028 
     23.958      10.324 
      2.745       1.098 
 
 9  122.0 
     57.974      23.648 
     26.034      11.242 
      2.787       1.114 
 
 9  136.0 
     61.045      25.172 
     27.767      11.894 
      2.807       1.121 
 
 9  150.0 
     62.895      27.902 
     29.500      12.635 
      2.831       1.132 
 
 10  10.0 
      8.765       2.730 
      4.945       0.995 
      1.878       0.395 
 
 10  24.0 
     19.943       7.855 
      8.718       2.103 
      2.304       0.500 
 
 10  38.0 
     29.183      11.299 
     11.678       2.755 
      2.467       0.526 
 
 10  52.0 
     37.216      14.879 
     14.383       3.576 
      2.577       0.566 
 
 10  66.0 
     45.979      20.619 
     16.846       4.313 
      2.663       0.586 
 
 10  80.0 
     53.750      26.446 
     19.110       4.929 
      2.710       0.593 
 
 10  94.0 
     59.406      29.904 
     21.254       5.692 
      2.758       0.614 
 
 10  108.0 
     66.768      32.771 
     23.290       6.209 
      2.790       0.614 
 
 10  122.0 
     71.275      36.182 
     25.252       6.918 
      2.822       0.628 
 
 10  136.0 
     71.010      33.135 
     27.236       7.484 
      2.853       0.632 
 
 10  150.0 
     72.402      34.097 
     29.000       8.062 
      2.875       0.636 
 
  Description  Seqs/Sample 
 chao1 Ave.  chao1 Err. 
 observed_species Ave.  observed_species Err. 
 shannon Ave.  shannon Err. 
 
 
 BW-01.1.Lib_L_27F-001  10.0 
      9.458         nan 
      5.550         nan 
      2.195         nan 
 
 BW-01.1.Lib_L_27F-001  24.0 
     18.133         nan 
      9.200         nan 
      2.665         nan 
 
 BW-01.1.Lib_L_27F-001  38.0 
     26.232         nan 
     12.020         nan 
      2.835         nan 
 
 BW-01.1.Lib_L_27F-001  52.0 
     36.648         nan 
     14.590         nan 
      2.947         nan 
 
 BW-01.1.Lib_L_27F-001  66.0 
     37.937         nan 
     16.700         nan 
      3.032         nan 
 
 BW-01.1.Lib_L_27F-001  80.0 
     40.032         nan 
     18.320         nan 
      3.069         nan 
 
 BW-01.1.Lib_L_27F-001  94.0 
     50.318         nan 
     20.300         nan 
      3.092         nan 
 
 BW-01.1.Lib_L_27F-001  108.0 
     50.094         nan 
     22.500         nan 
      3.165         nan 
 
 BW-01.1.Lib_L_27F-001  122.0 
     46.242         nan 
     23.930         nan 
      3.181         nan 
 
 BW-01.1.Lib_L_27F-001  136.0 
     52.703         nan 
     25.700         nan 
      3.202         nan 
 
 BW-01.1.Lib_L_27F-001  150.0 
     51.000         nan 
     27.000         nan 
      3.214         nan 
 
 BW-01.10.Lib_L_27F-010  10.0 
     12.647         nan 
      6.250         nan 
      2.356         nan 
 
 BW-01.10.Lib_L_27F-010  24.0 
     28.524         nan 
     11.680         nan 
      2.961         nan 
 
 BW-01.10.Lib_L_27F-010  38.0 
     37.325         nan 
     15.310         nan 
      3.120         nan 
 
 BW-01.10.Lib_L_27F-010  52.0 
     45.816         nan 
     19.180         nan 
      3.309         nan 
 
 BW-01.10.Lib_L_27F-010  66.0 
     59.458         nan 
     22.950         nan 
      3.438         nan 
 
 BW-01.10.Lib_L_27F-010  80.0 
     67.087         nan 
     25.630         nan 
      3.479         nan 
 
 BW-01.10.Lib_L_27F-010  94.0 
     78.057         nan 
     28.950         nan 
      3.576         nan 
 
 BW-01.10.Lib_L_27F-010  108.0 
     87.047         nan 
     31.360         nan 
      3.593         nan 
 
 BW-01.10.Lib_L_27F-010  122.0 
     97.598         nan 
     34.150         nan 
      3.656         nan 
 
 BW-01.10.Lib_L_27F-010  136.0 
     99.782         nan 
     36.720         nan 
      3.684         nan 
 
 BW-01.10.Lib_L_27F-010  150.0 
    104.000         nan 
     39.000         nan 
      3.711         nan 
 
 BW-01.2.Lib_L_27F-002  10.0 
      3.645         nan 
      2.670         nan 
      0.778         nan 
 
 BW-01.2.Lib_L_27F-002  24.0 
     10.032         nan 
      4.830         nan 
      0.994         nan 
 
 BW-01.2.Lib_L_27F-002  38.0 
     15.640         nan 
      6.940         nan 
      1.121         nan 
 
 BW-01.2.Lib_L_27F-002  52.0 
     20.715         nan 
      8.960         nan 
      1.208         nan 
 
 BW-01.2.Lib_L_27F-002  66.0 
     22.030         nan 
     10.240         nan 
      1.209         nan 
 
 BW-01.2.Lib_L_27F-002  80.0 
     21.851         nan 
     11.890         nan 
      1.280         nan 
 
 BW-01.2.Lib_L_27F-002  94.0 
     22.156         nan 
     13.250         nan 
      1.326         nan 
 
 BW-01.2.Lib_L_27F-002  108.0 
     21.839         nan 
     14.370         nan 
      1.344         nan 
 
 BW-01.2.Lib_L_27F-002  122.0 
     22.033         nan 
     15.280         nan 
      1.346         nan 
 
 BW-01.2.Lib_L_27F-002  136.0 
     22.075         nan 
     16.220         nan 
      1.354         nan 
 
 BW-01.2.Lib_L_27F-002  150.0 
     21.667         nan 
     17.000         nan 
      1.374         nan 
 
 BW-01.3.Lib_L_27F-003  10.0 
     13.232         nan 
      6.780         nan 
      2.563         nan 
 
 BW-01.3.Lib_L_27F-003  24.0 
     29.350         nan 
     12.740         nan 
      3.274         nan 
 
 BW-01.3.Lib_L_27F-003  38.0 
     39.987         nan 
     16.720         nan 
      3.504         nan 
 
 BW-01.3.Lib_L_27F-003  52.0 
     58.146         nan 
     20.530         nan 
      3.652         nan 
 
 BW-01.3.Lib_L_27F-003  66.0 
     67.540         nan 
     24.280         nan 
      3.779         nan 
 
 BW-01.3.Lib_L_27F-003  80.0 
     76.917         nan 
     27.340         nan 
      3.849         nan 
 
 BW-01.3.Lib_L_27F-003  94.0 
     75.768         nan 
     30.530         nan 
      3.917         nan 
 
 BW-01.3.Lib_L_27F-003  108.0 
     71.824         nan 
     32.900         nan 
      3.954         nan 
 
 BW-01.3.Lib_L_27F-003  122.0 
     75.283         nan 
     36.050         nan 
      4.003         nan 
 
 BW-01.3.Lib_L_27F-003  136.0 
     72.278         nan 
     38.470         nan 
      4.040         nan 
 
 BW-01.3.Lib_L_27F-003  150.0 
     71.000         nan 
     41.000         nan 
      4.077         nan 
 
 BW-01.4.Lib_L_27F-004  10.0 
      1.790         nan 
      1.610         nan 
      0.294         nan 
 
 BW-01.4.Lib_L_27F-004  24.0 
      3.260         nan 
      2.550         nan 
      0.414         nan 
 
 BW-01.4.Lib_L_27F-004  38.0 
      4.700         nan 
      3.280         nan 
      0.448         nan 
 
 BW-01.4.Lib_L_27F-004  52.0 
      8.010         nan 
      4.520         nan 
      0.535         nan 
 
 BW-01.4.Lib_L_27F-004  66.0 
     10.230         nan 
      5.180         nan 
      0.531         nan 
 
 BW-01.4.Lib_L_27F-004  80.0 
     11.370         nan 
      5.680         nan 
      0.534         nan 
 
 BW-01.4.Lib_L_27F-004  94.0 
     13.560         nan 
      6.360         nan 
      0.537         nan 
 
 BW-01.4.Lib_L_27F-004  108.0 
     16.785         nan 
      6.980         nan 
      0.553         nan 
 
 BW-01.4.Lib_L_27F-004  122.0 
     20.625         nan 
      7.590         nan 
      0.555         nan 
 
 BW-01.4.Lib_L_27F-004  136.0 
     25.690         nan 
      8.360         nan 
      0.573         nan 
 
 BW-01.4.Lib_L_27F-004  150.0 
     30.000         nan 
      9.000         nan 
      0.579         nan 
 
 BW-01.5.Lib_L_27F-005  10.0 
     16.330         nan 
      7.080         nan 
      2.615         nan 
 
 BW-01.5.Lib_L_27F-005  24.0 
     31.078         nan 
     12.540         nan 
      3.169         nan 
 
 BW-01.5.Lib_L_27F-005  38.0 
     52.156         nan 
     18.240         nan 
      3.566         nan 
 
 BW-01.5.Lib_L_27F-005  52.0 
     58.694         nan 
     21.690         nan 
      3.655         nan 
 
 BW-01.5.Lib_L_27F-005  66.0 
     69.374         nan 
     25.760         nan 
      3.774         nan 
 
 BW-01.5.Lib_L_27F-005  80.0 
     80.096         nan 
     29.330         nan 
      3.880         nan 
 
 BW-01.5.Lib_L_27F-005  94.0 
     89.044         nan 
     32.970         nan 
      3.955         nan 
 
 BW-01.5.Lib_L_27F-005  108.0 
     96.707         nan 
     36.380         nan 
      4.015         nan 
 
 BW-01.5.Lib_L_27F-005  122.0 
    101.616         nan 
     39.000         nan 
      4.038         nan 
 
 BW-01.5.Lib_L_27F-005  136.0 
    105.807         nan 
     41.980         nan 
      4.093         nan 
 
 BW-01.5.Lib_L_27F-005  150.0 
    117.500         nan 
     45.000         nan 
      4.133         nan 
 
 BW-01.6.Lib_L_27F-006  10.0 
     21.047         nan 
      7.980         nan 
      2.869         nan 
 
 BW-01.6.Lib_L_27F-006  24.0 
     42.126         nan 
     15.310         nan 
      3.653         nan 
 
 BW-01.6.Lib_L_27F-006  38.0 
     63.799         nan 
     21.350         nan 
      4.009         nan 
 
 BW-01.6.Lib_L_27F-006  52.0 
     78.377         nan 
     26.430         nan 
      4.178         nan 
 
 BW-01.6.Lib_L_27F-006  66.0 
    100.398         nan 
     31.640         nan 
      4.365         nan 
 
 BW-01.6.Lib_L_27F-006  80.0 
    100.524         nan 
     36.070         nan 
      4.467         nan 
 
 BW-01.6.Lib_L_27F-006  94.0 
    109.214         nan 
     40.260         nan 
      4.548         nan 
 
 BW-01.6.Lib_L_27F-006  108.0 
    105.583         nan 
     43.820         nan 
      4.603         nan 
 
 BW-01.6.Lib_L_27F-006  122.0 
    116.607         nan 
     48.000         nan 
      4.675         nan 
 
 BW-01.6.Lib_L_27F-006  136.0 
    114.702         nan 
     51.370         nan 
      4.724         nan 
 
 BW-01.6.Lib_L_27F-006  150.0 
    118.000         nan 
     55.000         nan 
      4.773         nan 
 
 BW-01.7.Lib_L_27F-007  10.0 
     23.570         nan 
      7.750         nan 
      2.761         nan 
 
 BW-01.7.Lib_L_27F-007  24.0 
     66.062         nan 
     15.620         nan 
      3.524         nan 
 
 BW-01.7.Lib_L_27F-007  38.0 
     87.092         nan 
     23.240         nan 
      3.976         nan 
 
 BW-01.7.Lib_L_27F-007  52.0 
    110.333         nan 
     29.430         nan 
      4.141         nan 
 
 BW-01.7.Lib_L_27F-007  66.0 
    105.217         nan 
     35.890         nan 
      4.383         nan 
 
 BW-01.7.Lib_L_27F-007  80.0 
    115.377         nan 
     40.930         nan 
      4.440         nan 
 
 BW-01.7.Lib_L_27F-007  94.0 
    111.484         nan 
     46.110         nan 
      4.569         nan 
 
 BW-01.7.Lib_L_27F-007  108.0 
    110.828         nan 
     51.290         nan 
      4.683         nan 
 
 BW-01.7.Lib_L_27F-007  122.0 
    110.086         nan 
     55.320         nan 
      4.725         nan 
 
 BW-01.7.Lib_L_27F-007  136.0 
    106.985         nan 
     59.440         nan 
      4.792         nan 
 
 BW-01.7.Lib_L_27F-007  150.0 
    106.588         nan 
     63.000         nan 
      4.832         nan 
 
 BW-01.8.Lib_L_27F-008  10.0 
     23.945         nan 
      7.980         nan 
      2.847         nan 
 
 BW-01.8.Lib_L_27F-008  24.0 
     59.431         nan 
     16.560         nan 
      3.740         nan 
 
 BW-01.8.Lib_L_27F-008  38.0 
     92.615         nan 
     23.650         nan 
      4.100         nan 
 
 BW-01.8.Lib_L_27F-008  52.0 
    135.062         nan 
     31.010         nan 
      4.390         nan 
 
 BW-01.8.Lib_L_27F-008  66.0 
    136.582         nan 
     37.120         nan 
      4.564         nan 
 
 BW-01.8.Lib_L_27F-008  80.0 
    142.705         nan 
     42.820         nan 
      4.677         nan 
 
 BW-01.8.Lib_L_27F-008  94.0 
    157.646         nan 
     49.030         nan 
      4.823         nan 
 
 BW-01.8.Lib_L_27F-008  108.0 
    161.133         nan 
     54.330         nan 
      4.909         nan 
 
 BW-01.8.Lib_L_27F-008  122.0 
    174.453         nan 
     59.580         nan 
      4.984         nan 
 
 BW-01.8.Lib_L_27F-008  136.0 
    173.695         nan 
     64.040         nan 
      5.034         nan 
 
 BW-01.8.Lib_L_27F-008  150.0 
    180.364         nan 
     69.000         nan 
      5.099         nan 
 
 BW-01.9.Lib_L_27F-009  10.0 
     25.525         nan 
      8.480         nan 
      2.991         nan 
 
 BW-01.9.Lib_L_27F-009  24.0 
     46.387         nan 
     17.260         nan 
      3.916         nan 
 
 BW-01.9.Lib_L_27F-009  38.0 
     65.123         nan 
     23.810         nan 
      4.271         nan 
 
 BW-01.9.Lib_L_27F-009  52.0 
     71.441         nan 
     29.920         nan 
      4.532         nan 
 
 BW-01.9.Lib_L_27F-009  66.0 
     76.679         nan 
     34.460         nan 
      4.662         nan 
 
 BW-01.9.Lib_L_27F-009  80.0 
     90.270         nan 
     39.390         nan 
      4.801         nan 
 
 BW-01.9.Lib_L_27F-009  94.0 
     92.441         nan 
     43.310         nan 
      4.877         nan 
 
 BW-01.9.Lib_L_27F-009  108.0 
     93.047         nan 
     46.770         nan 
      4.944         nan 
 
 BW-01.9.Lib_L_27F-009  122.0 
     95.552         nan 
     50.890         nan 
      5.028         nan 
 
 BW-01.9.Lib_L_27F-009  136.0 
     94.404         nan 
     53.730         nan 
      5.057         nan 
 
 BW-01.9.Lib_L_27F-009  150.0 
     98.333         nan 
     57.000         nan 
      5.110         nan 
 
 BW-02.1.Lib_L_27F-011  10.0 
      6.312         nan 
      4.220         nan 
      1.651         nan 
 
 BW-02.1.Lib_L_27F-011  24.0 
     12.370         nan 
      7.060         nan 
      2.014         nan 
 
 BW-02.1.Lib_L_27F-011  38.0 
     16.984         nan 
      8.860         nan 
      2.093         nan 
 
 BW-02.1.Lib_L_27F-011  52.0 
     23.038         nan 
     10.890         nan 
      2.194         nan 
 
 BW-02.1.Lib_L_27F-011  66.0 
     25.660         nan 
     12.170         nan 
      2.226         nan 
 
 BW-02.1.Lib_L_27F-011  80.0 
     28.064         nan 
     13.480         nan 
      2.247         nan 
 
 BW-02.1.Lib_L_27F-011  94.0 
     32.109         nan 
     14.500         nan 
      2.254         nan 
 
 BW-02.1.Lib_L_27F-011  108.0 
     43.021         nan 
     16.030         nan 
      2.296         nan 
 
 BW-02.1.Lib_L_27F-011  122.0 
     50.995         nan 
     17.660         nan 
      2.340         nan 
 
 BW-02.1.Lib_L_27F-011  136.0 
     56.048         nan 
     18.790         nan 
      2.361         nan 
 
 BW-02.1.Lib_L_27F-011  150.0 
     59.000         nan 
     20.000         nan 
      2.372         nan 
 
 BW-02.10.Lib_L_27F-021  10.0 
      6.932         nan 
      4.200         nan 
      1.594         nan 
 
 BW-02.10.Lib_L_27F-021  24.0 
     18.963         nan 
      7.620         nan 
      2.006         nan 
 
 BW-02.10.Lib_L_27F-021  38.0 
     32.350         nan 
     10.560         nan 
      2.165         nan 
 
 BW-02.10.Lib_L_27F-021  52.0 
     45.798         nan 
     13.220         nan 
      2.235         nan 
 
 BW-02.10.Lib_L_27F-021  66.0 
     51.646         nan 
     15.770         nan 
      2.333         nan 
 
 BW-02.10.Lib_L_27F-021  80.0 
     62.569         nan 
     18.350         nan 
      2.382         nan 
 
 BW-02.10.Lib_L_27F-021  94.0 
     72.139         nan 
     21.060         nan 
      2.441         nan 
 
 BW-02.10.Lib_L_27F-021  108.0 
     87.216         nan 
     23.190         nan 
      2.471         nan 
 
 BW-02.10.Lib_L_27F-021  122.0 
     92.563         nan 
     25.520         nan 
      2.505         nan 
 
 BW-02.10.Lib_L_27F-021  136.0 
     94.609         nan 
     27.830         nan 
      2.531         nan 
 
 BW-02.10.Lib_L_27F-021  150.0 
     93.250         nan 
     30.000         nan 
      2.561         nan 
 
 BW-02.2.Lib_L_27F-013  10.0 
      6.080         nan 
      4.610         nan 
      1.939         nan 
 
 BW-02.2.Lib_L_27F-013  24.0 
     12.058         nan 
      7.190         nan 
      2.311         nan 
 
 BW-02.2.Lib_L_27F-013  38.0 
     16.133         nan 
      8.460         nan 
      2.346         nan 
 
 BW-02.2.Lib_L_27F-013  52.0 
     24.332         nan 
     10.580         nan 
      2.478         nan 
 
 BW-02.2.Lib_L_27F-013  66.0 
     29.536         nan 
     12.320         nan 
      2.539         nan 
 
 BW-02.2.Lib_L_27F-013  80.0 
     33.723         nan 
     13.470         nan 
      2.546         nan 
 
 BW-02.2.Lib_L_27F-013  94.0 
     35.568         nan 
     14.900         nan 
      2.569         nan 
 
 BW-02.2.Lib_L_27F-013  108.0 
     35.705         nan 
     16.070         nan 
      2.587         nan 
 
 BW-02.2.Lib_L_27F-013  122.0 
     34.077         nan 
     17.610         nan 
      2.630         nan 
 
 BW-02.2.Lib_L_27F-013  136.0 
     35.694         nan 
     19.000         nan 
      2.646         nan 
 
 BW-02.2.Lib_L_27F-013  150.0 
     33.200         nan 
     20.000         nan 
      2.658         nan 
 
 BW-02.3.Lib_L_27F-014  10.0 
      9.058         nan 
      5.400         nan 
      2.110         nan 
 
 BW-02.3.Lib_L_27F-014  24.0 
     21.968         nan 
      9.210         nan 
      2.527         nan 
 
 BW-02.3.Lib_L_27F-014  38.0 
     37.643         nan 
     12.450         nan 
      2.696         nan 
 
 BW-02.3.Lib_L_27F-014  52.0 
     58.170         nan 
     15.600         nan 
      2.831         nan 
 
 BW-02.3.Lib_L_27F-014  66.0 
     62.150         nan 
     17.820         nan 
      2.862         nan 
 
 BW-02.3.Lib_L_27F-014  80.0 
     73.822         nan 
     21.140         nan 
      2.974         nan 
 
 BW-02.3.Lib_L_27F-014  94.0 
     86.112         nan 
     23.360         nan 
      3.001         nan 
 
 BW-02.3.Lib_L_27F-014  108.0 
     92.334         nan 
     25.930         nan 
      3.038         nan 
 
 BW-02.3.Lib_L_27F-014  122.0 
     83.474         nan 
     28.370         nan 
      3.081         nan 
 
 BW-02.3.Lib_L_27F-014  136.0 
     90.713         nan 
     30.830         nan 
      3.114         nan 
 
 BW-02.3.Lib_L_27F-014  150.0 
     88.200         nan 
     33.000         nan 
      3.139         nan 
 
 BW-02.4.Lib_L_27F-015  10.0 
     12.475         nan 
      6.620         nan 
      2.520         nan 
 
 BW-02.4.Lib_L_27F-015  24.0 
     32.001         nan 
     11.530         nan 
      3.068         nan 
 
 BW-02.4.Lib_L_27F-015  38.0 
     44.231         nan 
     15.690         nan 
      3.325         nan 
 
 BW-02.4.Lib_L_27F-015  52.0 
     60.826         nan 
     19.190         nan 
      3.436         nan 
 
 BW-02.4.Lib_L_27F-015  66.0 
     71.918         nan 
     22.700         nan 
      3.539         nan 
 
 BW-02.4.Lib_L_27F-015  80.0 
     74.218         nan 
     25.850         nan 
      3.623         nan 
 
 BW-02.4.Lib_L_27F-015  94.0 
     81.833         nan 
     28.550         nan 
      3.647         nan 
 
 BW-02.4.Lib_L_27F-015  108.0 
     78.776         nan 
     32.030         nan 
      3.726         nan 
 
 BW-02.4.Lib_L_27F-015  122.0 
     73.084         nan 
     34.770         nan 
      3.775         nan 
 
 BW-02.4.Lib_L_27F-015  136.0 
     77.372         nan 
     37.690         nan 
      3.810         nan 
 
 BW-02.4.Lib_L_27F-015  150.0 
     72.500         nan 
     40.000         nan 
      3.840         nan 
 
 BW-02.5.Lib_L_27F-016  10.0 
      8.470         nan 
      5.540         nan 
      2.200         nan 
 
 BW-02.5.Lib_L_27F-016  24.0 
     14.732         nan 
      8.570         nan 
      2.587         nan 
 
 BW-02.5.Lib_L_27F-016  38.0 
     24.365         nan 
     11.320         nan 
      2.789         nan 
 
 BW-02.5.Lib_L_27F-016  52.0 
     43.670         nan 
     14.030         nan 
      2.898         nan 
 
 BW-02.5.Lib_L_27F-016  66.0 
     63.993         nan 
     16.540         nan 
      2.983         nan 
 
 BW-02.5.Lib_L_27F-016  80.0 
     78.710         nan 
     18.610         nan 
      3.025         nan 
 
 BW-02.5.Lib_L_27F-016  94.0 
     85.495         nan 
     20.180         nan 
      3.020         nan 
 
 BW-02.5.Lib_L_27F-016  108.0 
    103.907         nan 
     22.730         nan 
      3.079         nan 
 
 BW-02.5.Lib_L_27F-016  122.0 
    111.160         nan 
     24.860         nan 
      3.113         nan 
 
 BW-02.5.Lib_L_27F-016  136.0 
    105.662         nan 
     26.970         nan 
      3.145         nan 
 
 BW-02.5.Lib_L_27F-016  150.0 
    106.000         nan 
     29.000         nan 
      3.164         nan 
 
 BW-02.6.Lib_L_27F-017  10.0 
      4.380         nan 
      2.780         nan 
      0.812         nan 
 
 BW-02.6.Lib_L_27F-017  24.0 
     11.375         nan 
      5.000         nan 
      1.004         nan 
 
 BW-02.6.Lib_L_27F-017  38.0 
     17.888         nan 
      6.790         nan 
      1.063         nan 
 
 BW-02.6.Lib_L_27F-017  52.0 
     26.489         nan 
      9.030         nan 
      1.185         nan 
 
 BW-02.6.Lib_L_27F-017  66.0 
     28.792         nan 
     10.410         nan 
      1.183         nan 
 
 BW-02.6.Lib_L_27F-017  80.0 
     34.219         nan 
     12.490         nan 
      1.264         nan 
 
 BW-02.6.Lib_L_27F-017  94.0 
     45.162         nan 
     14.370         nan 
      1.303         nan 
 
 BW-02.6.Lib_L_27F-017  108.0 
     45.027         nan 
     15.630         nan 
      1.313         nan 
 
 BW-02.6.Lib_L_27F-017  122.0 
     46.306         nan 
     16.920         nan 
      1.323         nan 
 
 BW-02.6.Lib_L_27F-017  136.0 
     53.378         nan 
     18.550         nan 
      1.343         nan 
 
 BW-02.6.Lib_L_27F-017  150.0 
     55.000         nan 
     20.000         nan 
      1.368         nan 
 
 BW-02.7.Lib_L_27F-018  10.0 
      3.335         nan 
      2.520         nan 
      0.717         nan 
 
 BW-02.7.Lib_L_27F-018  24.0 
      8.258         nan 
      4.390         nan 
      0.880         nan 
 
 BW-02.7.Lib_L_27F-018  38.0 
     13.035         nan 
      6.130         nan 
      0.985         nan 
 
 BW-02.7.Lib_L_27F-018  52.0 
     17.544         nan 
      7.880         nan 
      1.068         nan 
 
 BW-02.7.Lib_L_27F-018  66.0 
     21.239         nan 
      9.360         nan 
      1.099         nan 
 
 BW-02.7.Lib_L_27F-018  80.0 
     22.722         nan 
     10.920         nan 
      1.165         nan 
 
 BW-02.7.Lib_L_27F-018  94.0 
     24.101         nan 
     11.840         nan 
      1.160         nan 
 
 BW-02.7.Lib_L_27F-018  108.0 
     24.541         nan 
     12.780         nan 
      1.150         nan 
 
 BW-02.7.Lib_L_27F-018  122.0 
     27.701         nan 
     13.950         nan 
      1.191         nan 
 
 BW-02.7.Lib_L_27F-018  136.0 
     29.831         nan 
     14.960         nan 
      1.204         nan 
 
 BW-02.7.Lib_L_27F-018  150.0 
     31.000         nan 
     16.000         nan 
      1.225         nan 
 
 BW-02.8.Lib_L_27F-019  10.0 
      4.440         nan 
      3.100         nan 
      1.007         nan 
 
 BW-02.8.Lib_L_27F-019  24.0 
     11.139         nan 
      5.740         nan 
      1.284         nan 
 
 BW-02.8.Lib_L_27F-019  38.0 
     13.926         nan 
      7.520         nan 
      1.376         nan 
 
 BW-02.8.Lib_L_27F-019  52.0 
     16.113         nan 
      9.120         nan 
      1.463         nan 
 
 BW-02.8.Lib_L_27F-019  66.0 
     17.060         nan 
     10.470         nan 
      1.533         nan 
 
 BW-02.8.Lib_L_27F-019  80.0 
     19.529         nan 
     11.760         nan 
      1.560         nan 
 
 BW-02.8.Lib_L_27F-019  94.0 
     21.647         nan 
     12.830         nan 
      1.585         nan 
 
 BW-02.8.Lib_L_27F-019  108.0 
     22.301         nan 
     13.600         nan 
      1.593         nan 
 
 BW-02.8.Lib_L_27F-019  122.0 
     21.852         nan 
     14.220         nan 
      1.608         nan 
 
 BW-02.8.Lib_L_27F-019  136.0 
     24.590         nan 
     15.280         nan 
      1.625         nan 
 
 BW-02.8.Lib_L_27F-019  150.0 
     25.333         nan 
     16.000         nan 
      1.634         nan 
 
 BW-02.9.Lib_L_27F-020  10.0 
      1.370         nan 
      1.350         nan 
      0.164         nan 
 
 BW-02.9.Lib_L_27F-020  24.0 
      1.710         nan 
      1.630         nan 
      0.162         nan 
 
 BW-02.9.Lib_L_27F-020  38.0 
      2.530         nan 
      2.160         nan 
      0.213         nan 
 
 BW-02.9.Lib_L_27F-020  52.0 
      3.405         nan 
      2.630         nan 
      0.238         nan 
 
 BW-02.9.Lib_L_27F-020  66.0 
      4.160         nan 
      3.040         nan 
      0.251         nan 
 
 BW-02.9.Lib_L_27F-020  80.0 
      5.445         nan 
      3.540         nan 
      0.264         nan 
 
 BW-02.9.Lib_L_27F-020  94.0 
      5.235         nan 
      3.660         nan 
      0.253         nan 
 
 BW-02.9.Lib_L_27F-020  108.0 
      6.275         nan 
      4.110         nan 
      0.262         nan 
 
 BW-02.9.Lib_L_27F-020  122.0 
      6.855         nan 
      4.500         nan 
      0.272         nan 
 
 BW-02.9.Lib_L_27F-020  136.0 
      6.545         nan 
      4.720         nan 
      0.273         nan 
 
 BW-02.9.Lib_L_27F-020  150.0 
      6.500         nan 
      5.000         nan 
      0.275         nan 
 
 BW-03.1.Lib_L_27F-022  10.0 
     13.645         nan 
      6.210         nan 
      2.331         nan 
 
 BW-03.1.Lib_L_27F-022  24.0 
     29.487         nan 
     11.920         nan 
      2.994         nan 
 
 BW-03.1.Lib_L_27F-022  38.0 
     44.204         nan 
     16.270         nan 
      3.241         nan 
 
 BW-03.1.Lib_L_27F-022  52.0 
     52.781         nan 
     20.290         nan 
      3.409         nan 
 
 BW-03.1.Lib_L_27F-022  66.0 
     63.291         nan 
     24.330         nan 
      3.535         nan 
 
 BW-03.1.Lib_L_27F-022  80.0 
     71.780         nan 
     27.470         nan 
      3.596         nan 
 
 BW-03.1.Lib_L_27F-022  94.0 
     69.379         nan 
     30.220         nan 
      3.651         nan 
 
 BW-03.1.Lib_L_27F-022  108.0 
     69.746         nan 
     32.640         nan 
      3.684         nan 
 
 BW-03.1.Lib_L_27F-022  122.0 
     74.272         nan 
     35.960         nan 
      3.757         nan 
 
 BW-03.1.Lib_L_27F-022  136.0 
     73.545         nan 
     38.380         nan 
      3.790         nan 
 
 BW-03.1.Lib_L_27F-022  150.0 
     74.333         nan 
     41.000         nan 
      3.830         nan 
 
 BW-03.10.Lib_L_27F-031  10.0 
      6.453         nan 
      4.350         nan 
      1.732         nan 
 
 BW-03.10.Lib_L_27F-031  24.0 
     15.423         nan 
      7.470         nan 
      2.076         nan 
 
 BW-03.10.Lib_L_27F-031  38.0 
     21.468         nan 
      9.890         nan 
      2.200         nan 
 
 BW-03.10.Lib_L_27F-031  52.0 
     25.525         nan 
     12.040         nan 
      2.274         nan 
 
 BW-03.10.Lib_L_27F-031  66.0 
     28.871         nan 
     13.920         nan 
      2.338         nan 
 
 BW-03.10.Lib_L_27F-031  80.0 
     27.998         nan 
     15.410         nan 
      2.370         nan 
 
 BW-03.10.Lib_L_27F-031  94.0 
     28.234         nan 
     16.840         nan 
      2.400         nan 
 
 BW-03.10.Lib_L_27F-031  108.0 
     33.053         nan 
     18.450         nan 
      2.438         nan 
 
 BW-03.10.Lib_L_27F-031  122.0 
     33.825         nan 
     19.570         nan 
      2.447         nan 
 
 BW-03.10.Lib_L_27F-031  136.0 
     31.008         nan 
     20.890         nan 
      2.490         nan 
 
 BW-03.10.Lib_L_27F-031  150.0 
     31.167         nan 
     22.000         nan 
      2.507         nan 
 
 BW-03.3.Lib_L_27F-024  10.0 
      2.090         nan 
      1.790         nan 
      0.366         nan 
 
 BW-03.3.Lib_L_27F-024  24.0 
      3.695         nan 
      2.630         nan 
      0.412         nan 
 
 BW-03.3.Lib_L_27F-024  38.0 
      6.040         nan 
      3.600         nan 
      0.471         nan 
 
 BW-03.3.Lib_L_27F-024  52.0 
      7.628         nan 
      4.290         nan 
      0.483         nan 
 
 BW-03.3.Lib_L_27F-024  66.0 
      9.945         nan 
      5.170         nan 
      0.513         nan 
 
 BW-03.3.Lib_L_27F-024  80.0 
     12.193         nan 
      6.200         nan 
      0.559         nan 
 
 BW-03.3.Lib_L_27F-024  94.0 
     11.938         nan 
      6.610         nan 
      0.548         nan 
 
 BW-03.3.Lib_L_27F-024  108.0 
     11.902         nan 
      7.340         nan 
      0.576         nan 
 
 BW-03.3.Lib_L_27F-024  122.0 
     11.984         nan 
      7.880         nan 
      0.571         nan 
 
 BW-03.3.Lib_L_27F-024  136.0 
     11.627         nan 
      8.500         nan 
      0.590         nan 
 
 BW-03.3.Lib_L_27F-024  150.0 
     11.500         nan 
      9.000         nan 
      0.592         nan 
 
 BW-03.4.Lib_L_27F-025  10.0 
     13.279         nan 
      6.560         nan 
      2.481         nan 
 
 BW-03.4.Lib_L_27F-025  24.0 
     27.540         nan 
     11.640         nan 
      3.064         nan 
 
 BW-03.4.Lib_L_27F-025  38.0 
     42.852         nan 
     16.300         nan 
      3.374         nan 
 
 BW-03.4.Lib_L_27F-025  52.0 
     47.929         nan 
     19.790         nan 
      3.507         nan 
 
 BW-03.4.Lib_L_27F-025  66.0 
     52.488         nan 
     23.060         nan 
      3.599         nan 
 
 BW-03.4.Lib_L_27F-025  80.0 
     57.886         nan 
     26.600         nan 
      3.703         nan 
 
 BW-03.4.Lib_L_27F-025  94.0 
     58.845         nan 
     28.730         nan 
      3.719         nan 
 
 BW-03.4.Lib_L_27F-025  108.0 
     63.230         nan 
     31.360         nan 
      3.791         nan 
 
 BW-03.4.Lib_L_27F-025  122.0 
     67.141         nan 
     33.940         nan 
      3.825         nan 
 
 BW-03.4.Lib_L_27F-025  136.0 
     68.751         nan 
     35.890         nan 
      3.857         nan 
 
 BW-03.4.Lib_L_27F-025  150.0 
     76.500         nan 
     38.000         nan 
      3.886         nan 
 
 BW-03.5.Lib_L_27F-026  10.0 
      7.135         nan 
      4.030         nan 
      1.407         nan 
 
 BW-03.5.Lib_L_27F-026  24.0 
     14.153         nan 
      7.160         nan 
      1.691         nan 
 
 BW-03.5.Lib_L_27F-026  38.0 
     16.875         nan 
      9.760         nan 
      1.878         nan 
 
 BW-03.5.Lib_L_27F-026  52.0 
     21.178         nan 
     12.410         nan 
      2.046         nan 
 
 BW-03.5.Lib_L_27F-026  66.0 
     22.372         nan 
     13.850         nan 
      2.084         nan 
 
 BW-03.5.Lib_L_27F-026  80.0 
     24.911         nan 
     15.260         nan 
      2.112         nan 
 
 BW-03.5.Lib_L_27F-026  94.0 
     27.652         nan 
     16.770         nan 
      2.177         nan 
 
 BW-03.5.Lib_L_27F-026  108.0 
     32.794         nan 
     17.900         nan 
      2.186         nan 
 
 BW-03.5.Lib_L_27F-026  122.0 
     36.779         nan 
     18.840         nan 
      2.197         nan 
 
 BW-03.5.Lib_L_27F-026  136.0 
     42.298         nan 
     19.940         nan 
      2.215         nan 
 
 BW-03.5.Lib_L_27F-026  150.0 
     48.500         nan 
     21.000         nan 
      2.226         nan 
 
 BW-03.6.Lib_L_27F-027  10.0 
      7.350         nan 
      4.100         nan 
      1.444         nan 
 
 BW-03.6.Lib_L_27F-027  24.0 
     14.613         nan 
      7.190         nan 
      1.753         nan 
 
 BW-03.6.Lib_L_27F-027  38.0 
     18.117         nan 
      9.880         nan 
      1.936         nan 
 
 BW-03.6.Lib_L_27F-027  52.0 
     25.114         nan 
     12.400         nan 
      2.091         nan 
 
 BW-03.6.Lib_L_27F-027  66.0 
     29.320         nan 
     14.460         nan 
      2.163         nan 
 
 BW-03.6.Lib_L_27F-027  80.0 
     33.571         nan 
     16.250         nan 
      2.210         nan 
 
 BW-03.6.Lib_L_27F-027  94.0 
     37.073         nan 
     17.490         nan 
      2.206         nan 
 
 BW-03.6.Lib_L_27F-027  108.0 
     38.788         nan 
     18.860         nan 
      2.217         nan 
 
 BW-03.6.Lib_L_27F-027  122.0 
     39.675         nan 
     20.380         nan 
      2.250         nan 
 
 BW-03.6.Lib_L_27F-027  136.0 
     38.021         nan 
     21.660         nan 
      2.276         nan 
 
 BW-03.6.Lib_L_27F-027  150.0 
     38.600         nan 
     23.000         nan 
      2.297         nan 
 
 BW-03.7.Lib_L_27F-028  10.0 
      7.085         nan 
      4.450         nan 
      1.755         nan 
 
 BW-03.7.Lib_L_27F-028  24.0 
     17.678         nan 
      7.750         nan 
      2.111         nan 
 
 BW-03.7.Lib_L_27F-028  38.0 
     31.873         nan 
     11.380         nan 
      2.409         nan 
 
 BW-03.7.Lib_L_27F-028  52.0 
     42.136         nan 
     13.800         nan 
      2.432         nan 
 
 BW-03.7.Lib_L_27F-028  66.0 
     50.590         nan 
     16.620         nan 
      2.533         nan 
 
 BW-03.7.Lib_L_27F-028  80.0 
     52.762         nan 
     19.180         nan 
      2.632         nan 
 
 BW-03.7.Lib_L_27F-028  94.0 
     55.188         nan 
     21.460         nan 
      2.664         nan 
 
 BW-03.7.Lib_L_27F-028  108.0 
     57.148         nan 
     23.460         nan 
      2.696         nan 
 
 BW-03.7.Lib_L_27F-028  122.0 
     56.968         nan 
     24.950         nan 
      2.674         nan 
 
 BW-03.7.Lib_L_27F-028  136.0 
     54.508         nan 
     27.060         nan 
      2.712         nan 
 
 BW-03.7.Lib_L_27F-028  150.0 
     53.429         nan 
     29.000         nan 
      2.748         nan 
 
 BW-03.8.Lib_L_27F-029  10.0 
      8.053         nan 
      5.100         nan 
      2.009         nan 
 
 BW-03.8.Lib_L_27F-029  24.0 
     16.135         nan 
      8.490         nan 
      2.432         nan 
 
 BW-03.8.Lib_L_27F-029  38.0 
     22.312         nan 
     10.850         nan 
      2.531         nan 
 
 BW-03.8.Lib_L_27F-029  52.0 
     32.647         nan 
     13.510         nan 
      2.659         nan 
 
 BW-03.8.Lib_L_27F-029  66.0 
     42.212         nan 
     15.870         nan 
      2.736         nan 
 
 BW-03.8.Lib_L_27F-029  80.0 
     42.798         nan 
     17.820         nan 
      2.797         nan 
 
 BW-03.8.Lib_L_27F-029  94.0 
     42.926         nan 
     19.200         nan 
      2.818         nan 
 
 BW-03.8.Lib_L_27F-029  108.0 
     48.031         nan 
     21.210         nan 
      2.868         nan 
 
 BW-03.8.Lib_L_27F-029  122.0 
     51.452         nan 
     23.040         nan 
      2.899         nan 
 
 BW-03.8.Lib_L_27F-029  136.0 
     55.163         nan 
     24.450         nan 
      2.915         nan 
 
 BW-03.8.Lib_L_27F-029  150.0 
     56.000         nan 
     26.000         nan 
      2.941         nan 
 
 BW-03.9.Lib_L_27F-030  10.0 
      9.167         nan 
      5.360         nan 
      2.119         nan 
 
 BW-03.9.Lib_L_27F-030  24.0 
     21.315         nan 
      9.200         nan 
      2.548         nan 
 
 BW-03.9.Lib_L_27F-030  38.0 
     33.984         nan 
     12.390         nan 
      2.734         nan 
 
 BW-03.9.Lib_L_27F-030  52.0 
     45.453         nan 
     15.680         nan 
      2.891         nan 
 
 BW-03.9.Lib_L_27F-030  66.0 
     52.049         nan 
     18.440         nan 
      2.970         nan 
 
 BW-03.9.Lib_L_27F-030  80.0 
     57.387         nan 
     20.890         nan 
      3.032         nan 
 
 BW-03.9.Lib_L_27F-030  94.0 
     61.895         nan 
     23.860         nan 
      3.113         nan 
 
 BW-03.9.Lib_L_27F-030  108.0 
     61.553         nan 
     25.900         nan 
      3.126         nan 
 
 BW-03.9.Lib_L_27F-030  122.0 
     60.813         nan 
     28.270         nan 
      3.170         nan 
 
 BW-03.9.Lib_L_27F-030  136.0 
     57.058         nan 
     29.950         nan 
      3.186         nan 
 
 BW-03.9.Lib_L_27F-030  150.0 
     55.750         nan 
     32.000         nan 
      3.216         nan 
 
 BW-04.1.Lib_L_27F-032  10.0 
     14.675         nan 
      6.990         nan 
      2.620         nan 
 
 BW-04.1.Lib_L_27F-032  24.0 
     30.584         nan 
     12.670         nan 
      3.290         nan 
 
 BW-04.1.Lib_L_27F-032  38.0 
     57.105         nan 
     17.910         nan 
      3.612         nan 
 
 BW-04.1.Lib_L_27F-032  52.0 
     56.987         nan 
     21.570         nan 
      3.742         nan 
 
 BW-04.1.Lib_L_27F-032  66.0 
     63.085         nan 
     25.760         nan 
      3.897         nan 
 
 BW-04.1.Lib_L_27F-032  80.0 
     64.641         nan 
     28.510         nan 
      3.925         nan 
 
 BW-04.1.Lib_L_27F-032  94.0 
     65.369         nan 
     32.230         nan 
      4.030         nan 
 
 BW-04.1.Lib_L_27F-032  108.0 
     66.895         nan 
     35.040         nan 
      4.087         nan 
 
 BW-04.1.Lib_L_27F-032  122.0 
     65.450         nan 
     37.200         nan 
      4.106         nan 
 
 BW-04.1.Lib_L_27F-032  136.0 
     65.456         nan 
     39.980         nan 
      4.166         nan 
 
 BW-04.1.Lib_L_27F-032  150.0 
     65.000         nan 
     42.000         nan 
      4.186         nan 
 
 BW-04.10.Lib_L_27F-041  10.0 
     12.313         nan 
      5.640         nan 
      2.094         nan 
 
 BW-04.10.Lib_L_27F-041  24.0 
     34.969         nan 
     10.930         nan 
      2.647         nan 
 
 BW-04.10.Lib_L_27F-041  38.0 
     53.576         nan 
     14.900         nan 
      2.825         nan 
 
 BW-04.10.Lib_L_27F-041  52.0 
     67.693         nan 
     18.870         nan 
      2.982         nan 
 
 BW-04.10.Lib_L_27F-041  66.0 
     84.308         nan 
     22.530         nan 
      3.092         nan 
 
 BW-04.10.Lib_L_27F-041  80.0 
     97.737         nan 
     26.320         nan 
      3.187         nan 
 
 BW-04.10.Lib_L_27F-041  94.0 
     94.439         nan 
     29.400         nan 
      3.237         nan 
 
 BW-04.10.Lib_L_27F-041  108.0 
    104.735         nan 
     32.670         nan 
      3.281         nan 
 
 BW-04.10.Lib_L_27F-041  122.0 
    110.766         nan 
     36.050         nan 
      3.344         nan 
 
 BW-04.10.Lib_L_27F-041  136.0 
    114.089         nan 
     39.200         nan 
      3.391         nan 
 
 BW-04.10.Lib_L_27F-041  150.0 
    119.500         nan 
     42.000         nan 
      3.418         nan 
 
 BW-04.2.Lib_L_27F-033  10.0 
      7.125         nan 
      4.340         nan 
      1.684         nan 
 
 BW-04.2.Lib_L_27F-033  24.0 
     13.890         nan 
      7.250         nan 
      2.062         nan 
 
 BW-04.2.Lib_L_27F-033  38.0 
     22.318         nan 
      9.790         nan 
      2.230         nan 
 
 BW-04.2.Lib_L_27F-033  52.0 
     26.612         nan 
     11.370         nan 
      2.265         nan 
 
 BW-04.2.Lib_L_27F-033  66.0 
     37.992         nan 
     13.580         nan 
      2.353         nan 
 
 BW-04.2.Lib_L_27F-033  80.0 
     49.468         nan 
     15.510         nan 
      2.406         nan 
 
 BW-04.2.Lib_L_27F-033  94.0 
     54.660         nan 
     16.960         nan 
      2.411         nan 
 
 BW-04.2.Lib_L_27F-033  108.0 
     56.370         nan 
     18.730         nan 
      2.430         nan 
 
 BW-04.2.Lib_L_27F-033  122.0 
     62.696         nan 
     20.690         nan 
      2.470         nan 
 
 BW-04.2.Lib_L_27F-033  136.0 
     61.673         nan 
     22.400         nan 
      2.489         nan 
 
 BW-04.2.Lib_L_27F-033  150.0 
     58.000         nan 
     24.000         nan 
      2.515         nan 
 
 BW-04.3.Lib_L_27F-034  10.0 
     26.200         nan 
      8.680         nan 
      3.042         nan 
 
 BW-04.3.Lib_L_27F-034  24.0 
     59.184         nan 
     18.170         nan 
      4.011         nan 
 
 BW-04.3.Lib_L_27F-034  38.0 
     68.557         nan 
     25.120         nan 
      4.357         nan 
 
 BW-04.3.Lib_L_27F-034  52.0 
     76.084         nan 
     31.430         nan 
      4.626         nan 
 
 BW-04.3.Lib_L_27F-034  66.0 
     78.806         nan 
     37.620         nan 
      4.825         nan 
 
 BW-04.3.Lib_L_27F-034  80.0 
     82.485         nan 
     42.030         nan 
      4.930         nan 
 
 BW-04.3.Lib_L_27F-034  94.0 
     85.589         nan 
     46.720         nan 
      5.031         nan 
 
 BW-04.3.Lib_L_27F-034  108.0 
     87.387         nan 
     51.010         nan 
      5.122         nan 
 
 BW-04.3.Lib_L_27F-034  122.0 
     89.141         nan 
     54.670         nan 
      5.186         nan 
 
 BW-04.3.Lib_L_27F-034  136.0 
     89.992         nan 
     58.040         nan 
      5.241         nan 
 
 BW-04.3.Lib_L_27F-034  150.0 
     88.353         nan 
     61.000         nan 
      5.288         nan 
 
 BW-04.4.Lib_L_27F-035  10.0 
      5.578         nan 
      3.720         nan 
      1.339         nan 
 
 BW-04.4.Lib_L_27F-035  24.0 
     13.052         nan 
      6.200         nan 
      1.577         nan 
 
 BW-04.4.Lib_L_27F-035  38.0 
     22.705         nan 
      8.670         nan 
      1.753         nan 
 
 BW-04.4.Lib_L_27F-035  52.0 
     29.427         nan 
     10.540         nan 
      1.790         nan 
 
 BW-04.4.Lib_L_27F-035  66.0 
     39.005         nan 
     12.590         nan 
      1.825         nan 
 
 BW-04.4.Lib_L_27F-035  80.0 
     47.828         nan 
     15.050         nan 
      1.926         nan 
 
 BW-04.4.Lib_L_27F-035  94.0 
     56.888         nan 
     17.090         nan 
      1.958         nan 
 
 BW-04.4.Lib_L_27F-035  108.0 
     52.736         nan 
     18.590         nan 
      1.987         nan 
 
 BW-04.4.Lib_L_27F-035  122.0 
     61.386         nan 
     20.420         nan 
      2.006         nan 
 
 BW-04.4.Lib_L_27F-035  136.0 
     70.329         nan 
     22.260         nan 
      2.037         nan 
 
 BW-04.4.Lib_L_27F-035  150.0 
     75.000         nan 
     24.000         nan 
      2.055         nan 
 
 BW-04.5.Lib_L_27F-036  10.0 
      4.655         nan 
      3.660         nan 
      1.514         nan 
 
 BW-04.5.Lib_L_27F-036  24.0 
      9.670         nan 
      5.580         nan 
      1.718         nan 
 
 BW-04.5.Lib_L_27F-036  38.0 
     18.045         nan 
      7.550         nan 
      1.819         nan 
 
 BW-04.5.Lib_L_27F-036  52.0 
     29.045         nan 
      9.680         nan 
      1.952         nan 
 
 BW-04.5.Lib_L_27F-036  66.0 
     40.510         nan 
     11.350         nan 
      1.963         nan 
 
 BW-04.5.Lib_L_27F-036  80.0 
     56.175         nan 
     12.930         nan 
      1.997         nan 
 
 BW-04.5.Lib_L_27F-036  94.0 
     82.840         nan 
     15.140         nan 
      2.061         nan 
 
 BW-04.5.Lib_L_27F-036  108.0 
    104.325         nan 
     16.650         nan 
      2.075         nan 
 
 BW-04.5.Lib_L_27F-036  122.0 
    130.860         nan 
     18.420         nan 
      2.094         nan 
 
 BW-04.5.Lib_L_27F-036  136.0 
    163.390         nan 
     20.390         nan 
      2.130         nan 
 
 BW-04.5.Lib_L_27F-036  150.0 
    193.000         nan 
     22.000         nan 
      2.140         nan 
 
 BW-04.6.Lib_L_27F-037  10.0 
     12.710         nan 
      6.290         nan 
      2.393         nan 
 
 BW-04.6.Lib_L_27F-037  24.0 
     31.591         nan 
     11.600         nan 
      2.983         nan 
 
 BW-04.6.Lib_L_27F-037  38.0 
     49.217         nan 
     16.240         nan 
      3.252         nan 
 
 BW-04.6.Lib_L_27F-037  52.0 
     59.345         nan 
     20.290         nan 
      3.395         nan 
 
 BW-04.6.Lib_L_27F-037  66.0 
     75.205         nan 
     23.660         nan 
      3.487         nan 
 
 BW-04.6.Lib_L_27F-037  80.0 
     79.848         nan 
     27.590         nan 
      3.596         nan 
 
 BW-04.6.Lib_L_27F-037  94.0 
     84.833         nan 
     30.410         nan 
      3.645         nan 
 
 BW-04.6.Lib_L_27F-037  108.0 
     83.034         nan 
     33.820         nan 
      3.724         nan 
 
 BW-04.6.Lib_L_27F-037  122.0 
     79.172         nan 
     36.640         nan 
      3.757         nan 
 
 BW-04.6.Lib_L_27F-037  136.0 
     78.485         nan 
     39.320         nan 
      3.796         nan 
 
 BW-04.6.Lib_L_27F-037  150.0 
     77.100         nan 
     42.000         nan 
      3.833         nan 
 
 BW-04.7.Lib_L_27F-038  10.0 
      5.725         nan 
      3.670         nan 
      1.307         nan 
 
 BW-04.7.Lib_L_27F-038  24.0 
     11.083         nan 
      6.520         nan 
      1.692         nan 
 
 BW-04.7.Lib_L_27F-038  38.0 
     16.331         nan 
      8.630         nan 
      1.830         nan 
 
 BW-04.7.Lib_L_27F-038  52.0 
     18.794         nan 
      9.910         nan 
      1.833         nan 
 
 BW-04.7.Lib_L_27F-038  66.0 
     22.187         nan 
     11.310         nan 
      1.877         nan 
 
 BW-04.7.Lib_L_27F-038  80.0 
     24.889         nan 
     12.660         nan 
      1.910         nan 
 
 BW-04.7.Lib_L_27F-038  94.0 
     25.317         nan 
     13.810         nan 
      1.949         nan 
 
 BW-04.7.Lib_L_27F-038  108.0 
     27.722         nan 
     15.020         nan 
      1.996         nan 
 
 BW-04.7.Lib_L_27F-038  122.0 
     30.481         nan 
     15.920         nan 
      2.004         nan 
 
 BW-04.7.Lib_L_27F-038  136.0 
     32.467         nan 
     17.070         nan 
      2.016         nan 
 
 BW-04.7.Lib_L_27F-038  150.0 
     33.000         nan 
     18.000         nan 
      2.029         nan 
 
 BW-04.8.Lib_L_27F-039  10.0 
     20.312         nan 
      7.750         nan 
      2.805         nan 
 
 BW-04.8.Lib_L_27F-039  24.0 
     45.196         nan 
     15.630         nan 
      3.672         nan 
 
 BW-04.8.Lib_L_27F-039  38.0 
     71.203         nan 
     21.820         nan 
      4.001         nan 
 
 BW-04.8.Lib_L_27F-039  52.0 
     90.560         nan 
     27.120         nan 
      4.216         nan 
 
 BW-04.8.Lib_L_27F-039  66.0 
    102.045         nan 
     31.750         nan 
      4.335         nan 
 
 BW-04.8.Lib_L_27F-039  80.0 
    128.076         nan 
     36.800         nan 
      4.465         nan 
 
 BW-04.8.Lib_L_27F-039  94.0 
    154.330         nan 
     41.680         nan 
      4.561         nan 
 
 BW-04.8.Lib_L_27F-039  108.0 
    152.091         nan 
     45.820         nan 
      4.636         nan 
 
 BW-04.8.Lib_L_27F-039  122.0 
    159.149         nan 
     49.730         nan 
      4.687         nan 
 
 BW-04.8.Lib_L_27F-039  136.0 
    170.697         nan 
     53.920         nan 
      4.747         nan 
 
 BW-04.8.Lib_L_27F-039  150.0 
    181.000         nan 
     58.000         nan 
      4.797         nan 
 
 BW-04.9.Lib_L_27F-040  10.0 
      7.088         nan 
      4.310         nan 
      1.626         nan 
 
 BW-04.9.Lib_L_27F-040  24.0 
     15.595         nan 
      7.040         nan 
      1.880         nan 
 
 BW-04.9.Lib_L_27F-040  38.0 
     26.118         nan 
      9.650         nan 
      2.042         nan 
 
 BW-04.9.Lib_L_27F-040  52.0 
     35.380         nan 
     12.080         nan 
      2.117         nan 
 
 BW-04.9.Lib_L_27F-040  66.0 
     44.588         nan 
     14.200         nan 
      2.152         nan 
 
 BW-04.9.Lib_L_27F-040  80.0 
     48.093         nan 
     16.520         nan 
      2.219         nan 
 
 BW-04.9.Lib_L_27F-040  94.0 
     55.793         nan 
     18.410         nan 
      2.246         nan 
 
 BW-04.9.Lib_L_27F-040  108.0 
     63.247         nan 
     20.210         nan 
      2.262         nan 
 
 BW-04.9.Lib_L_27F-040  122.0 
     66.661         nan 
     22.480         nan 
      2.316         nan 
 
 BW-04.9.Lib_L_27F-040  136.0 
     79.100         nan 
     24.220         nan 
      2.326         nan 
 
 BW-04.9.Lib_L_27F-040  150.0 
     83.000         nan 
     26.000         nan 
      2.357         nan 
 
 BW-05.1.Lib_L_27F-042  10.0 
      5.310         nan 
      3.230         nan 
      1.028         nan 
 
 BW-05.1.Lib_L_27F-042  24.0 
     11.537         nan 
      5.710         nan 
      1.268         nan 
 
 BW-05.1.Lib_L_27F-042  38.0 
     15.649         nan 
      7.510         nan 
      1.332         nan 
 
 BW-05.1.Lib_L_27F-042  52.0 
     18.440         nan 
      9.480         nan 
      1.447         nan 
 
 BW-05.1.Lib_L_27F-042  66.0 
     22.954         nan 
     10.970         nan 
      1.464         nan 
 
 BW-05.1.Lib_L_27F-042  80.0 
     25.067         nan 
     12.460         nan 
      1.524         nan 
 
 BW-05.1.Lib_L_27F-042  94.0 
     27.380         nan 
     13.610         nan 
      1.541         nan 
 
 BW-05.1.Lib_L_27F-042  108.0 
     32.003         nan 
     14.810         nan 
      1.573         nan 
 
 BW-05.1.Lib_L_27F-042  122.0 
     34.398         nan 
     15.920         nan 
      1.578         nan 
 
 BW-05.1.Lib_L_27F-042  136.0 
     37.194         nan 
     17.070         nan 
      1.604         nan 
 
 BW-05.1.Lib_L_27F-042  150.0 
     45.500         nan 
     18.000         nan 
      1.608         nan 
 
 BW-05.10.Lib_L_27F-052  10.0 
     10.095         nan 
      5.200         nan 
      1.929         nan 
 
 BW-05.10.Lib_L_27F-052  24.0 
     18.405         nan 
      9.010         nan 
      2.294         nan 
 
 BW-05.10.Lib_L_27F-052  38.0 
     24.971         nan 
     12.010         nan 
      2.467         nan 
 
 BW-05.10.Lib_L_27F-052  52.0 
     31.391         nan 
     14.860         nan 
      2.640         nan 
 
 BW-05.10.Lib_L_27F-052  66.0 
     31.737         nan 
     16.780         nan 
      2.701         nan 
 
 BW-05.10.Lib_L_27F-052  80.0 
     38.183         nan 
     19.090         nan 
      2.775         nan 
 
 BW-05.10.Lib_L_27F-052  94.0 
     43.259         nan 
     20.670         nan 
      2.806         nan 
 
 BW-05.10.Lib_L_27F-052  108.0 
     48.751         nan 
     22.590         nan 
      2.846         nan 
 
 BW-05.10.Lib_L_27F-052  122.0 
     47.196         nan 
     24.010         nan 
      2.865         nan 
 
 BW-05.10.Lib_L_27F-052  136.0 
     49.574         nan 
     25.730         nan 
      2.897         nan 
 
 BW-05.10.Lib_L_27F-052  150.0 
     48.000         nan 
     27.000         nan 
      2.915         nan 
 
 BW-05.2.Lib_L_27F-043  10.0 
      7.140         nan 
      3.910         nan 
      1.344         nan 
 
 BW-05.2.Lib_L_27F-043  24.0 
     16.217         nan 
      7.080         nan 
      1.641         nan 
 
 BW-05.2.Lib_L_27F-043  38.0 
     17.871         nan 
      9.340         nan 
      1.768         nan 
 
 BW-05.2.Lib_L_27F-043  52.0 
     24.664         nan 
     11.610         nan 
      1.825         nan 
 
 BW-05.2.Lib_L_27F-043  66.0 
     27.573         nan 
     13.730         nan 
      1.903         nan 
 
 BW-05.2.Lib_L_27F-043  80.0 
     33.177         nan 
     15.410         nan 
      1.951         nan 
 
 BW-05.2.Lib_L_27F-043  94.0 
     42.991         nan 
     17.310         nan 
      2.025         nan 
 
 BW-05.2.Lib_L_27F-043  108.0 
     45.825         nan 
     18.560         nan 
      2.015         nan 
 
 BW-05.2.Lib_L_27F-043  122.0 
     54.624         nan 
     20.050         nan 
      2.041         nan 
 
 BW-05.2.Lib_L_27F-043  136.0 
     58.700         nan 
     21.960         nan 
      2.081         nan 
 
 BW-05.2.Lib_L_27F-043  150.0 
     58.000         nan 
     23.000         nan 
      2.084         nan 
 
 BW-05.3.Lib_L_27F-044  10.0 
      8.383         nan 
      4.840         nan 
      1.879         nan 
 
 BW-05.3.Lib_L_27F-044  24.0 
     19.114         nan 
      8.530         nan 
      2.294         nan 
 
 BW-05.3.Lib_L_27F-044  38.0 
     31.334         nan 
     11.640         nan 
      2.478         nan 
 
 BW-05.3.Lib_L_27F-044  52.0 
     33.374         nan 
     14.350         nan 
      2.587         nan 
 
 BW-05.3.Lib_L_27F-044  66.0 
     35.719         nan 
     16.820         nan 
      2.693         nan 
 
 BW-05.3.Lib_L_27F-044  80.0 
     41.662         nan 
     19.100         nan 
      2.744         nan 
 
 BW-05.3.Lib_L_27F-044  94.0 
     45.593         nan 
     21.280         nan 
      2.785         nan 
 
 BW-05.3.Lib_L_27F-044  108.0 
     47.265         nan 
     22.910         nan 
      2.807         nan 
 
 BW-05.3.Lib_L_27F-044  122.0 
     45.846         nan 
     24.840         nan 
      2.850         nan 
 
 BW-05.3.Lib_L_27F-044  136.0 
     47.333         nan 
     26.320         nan 
      2.877         nan 
 
 BW-05.3.Lib_L_27F-044  150.0 
     48.000         nan 
     28.000         nan 
      2.901         nan 
 
 BW-05.4.Lib_L_27F-045  10.0 
     18.423         nan 
      7.630         nan 
      2.778         nan 
 
 BW-05.4.Lib_L_27F-045  24.0 
     33.457         nan 
     13.830         nan 
      3.443         nan 
 
 BW-05.4.Lib_L_27F-045  38.0 
     41.073         nan 
     18.640         nan 
      3.744         nan 
 
 BW-05.4.Lib_L_27F-045  52.0 
     51.369         nan 
     22.970         nan 
      3.930         nan 
 
 BW-05.4.Lib_L_27F-045  66.0 
     60.433         nan 
     26.610         nan 
      4.023         nan 
 
 BW-05.4.Lib_L_27F-045  80.0 
     60.955         nan 
     29.780         nan 
      4.124         nan 
 
 BW-05.4.Lib_L_27F-045  94.0 
     66.064         nan 
     32.690         nan 
      4.184         nan 
 
 BW-05.4.Lib_L_27F-045  108.0 
     71.037         nan 
     35.200         nan 
      4.228         nan 
 
 BW-05.4.Lib_L_27F-045  122.0 
     72.904         nan 
     38.340         nan 
      4.298         nan 
 
 BW-05.4.Lib_L_27F-045  136.0 
     78.378         nan 
     40.780         nan 
      4.334         nan 
 
 BW-05.4.Lib_L_27F-045  150.0 
     77.500         nan 
     43.000         nan 
      4.366         nan 
 
 BW-05.5.Lib_L_27F-046  10.0 
      6.207         nan 
      4.630         nan 
      1.925         nan 
 
 BW-05.5.Lib_L_27F-046  24.0 
     12.772         nan 
      7.390         nan 
      2.307         nan 
 
 BW-05.5.Lib_L_27F-046  38.0 
     22.098         nan 
      9.750         nan 
      2.456         nan 
 
 BW-05.5.Lib_L_27F-046  52.0 
     27.802         nan 
     11.880         nan 
      2.564         nan 
 
 BW-05.5.Lib_L_27F-046  66.0 
     33.857         nan 
     12.990         nan 
      2.545         nan 
 
 BW-05.5.Lib_L_27F-046  80.0 
     38.832         nan 
     15.300         nan 
      2.644         nan 
 
 BW-05.5.Lib_L_27F-046  94.0 
     38.655         nan 
     17.020         nan 
      2.678         nan 
 
 BW-05.5.Lib_L_27F-046  108.0 
     41.913         nan 
     18.610         nan 
      2.708         nan 
 
 BW-05.5.Lib_L_27F-046  122.0 
     40.790         nan 
     20.230         nan 
      2.742         nan 
 
 BW-05.5.Lib_L_27F-046  136.0 
     39.702         nan 
     21.600         nan 
      2.751         nan 
 
 BW-05.5.Lib_L_27F-046  150.0 
     38.167         nan 
     23.000         nan 
      2.773         nan 
 
 BW-05.6.Lib_L_27F-047  10.0 
     18.733         nan 
      7.400         nan 
      2.689         nan 
 
 BW-05.6.Lib_L_27F-047  24.0 
     32.097         nan 
     13.530         nan 
      3.320         nan 
 
 BW-05.6.Lib_L_27F-047  38.0 
     45.808         nan 
     19.010         nan 
      3.668         nan 
 
 BW-05.6.Lib_L_27F-047  52.0 
     54.941         nan 
     23.180         nan 
      3.840         nan 
 
 BW-05.6.Lib_L_27F-047  66.0 
     65.028         nan 
     27.180         nan 
      3.968         nan 
 
 BW-05.6.Lib_L_27F-047  80.0 
     82.373         nan 
     30.320         nan 
      4.039         nan 
 
 BW-05.6.Lib_L_27F-047  94.0 
     90.534         nan 
     33.850         nan 
      4.112         nan 
 
 BW-05.6.Lib_L_27F-047  108.0 
    119.215         nan 
     37.260         nan 
      4.190         nan 
 
 BW-05.6.Lib_L_27F-047  122.0 
    143.313         nan 
     40.390         nan 
      4.239         nan 
 
 BW-05.6.Lib_L_27F-047  136.0 
    161.322         nan 
     43.040         nan 
      4.270         nan 
 
 BW-05.6.Lib_L_27F-047  150.0 
    201.000         nan 
     46.000         nan 
      4.310         nan 
 
 BW-05.7.Lib_L_27F-049  10.0 
     20.420         nan 
      7.700         nan 
      2.782         nan 
 
 BW-05.7.Lib_L_27F-049  24.0 
     40.184         nan 
     14.680         nan 
      3.534         nan 
 
 BW-05.7.Lib_L_27F-049  38.0 
     51.806         nan 
     19.740         nan 
      3.793         nan 
 
 BW-05.7.Lib_L_27F-049  52.0 
     72.172         nan 
     25.160         nan 
      4.037         nan 
 
 BW-05.7.Lib_L_27F-049  66.0 
     74.303         nan 
     29.530         nan 
      4.181         nan 
 
 BW-05.7.Lib_L_27F-049  80.0 
     83.897         nan 
     33.390         nan 
      4.265         nan 
 
 BW-05.7.Lib_L_27F-049  94.0 
     93.793         nan 
     37.610         nan 
      4.364         nan 
 
 BW-05.7.Lib_L_27F-049  108.0 
    101.114         nan 
     41.530         nan 
      4.449         nan 
 
 BW-05.7.Lib_L_27F-049  122.0 
    110.448         nan 
     44.710         nan 
      4.489         nan 
 
 BW-05.7.Lib_L_27F-049  136.0 
    119.193         nan 
     47.970         nan 
      4.546         nan 
 
 BW-05.7.Lib_L_27F-049  150.0 
    126.429         nan 
     51.000         nan 
      4.576         nan 
 
 BW-05.8.Lib_L_27F-050  10.0 
     15.928         nan 
      6.890         nan 
      2.531         nan 
 
 BW-05.8.Lib_L_27F-050  24.0 
     32.732         nan 
     12.560         nan 
      3.040         nan 
 
 BW-05.8.Lib_L_27F-050  38.0 
     39.724         nan 
     17.740         nan 
      3.381         nan 
 
 BW-05.8.Lib_L_27F-050  52.0 
     48.960         nan 
     21.820         nan 
      3.538         nan 
 
 BW-05.8.Lib_L_27F-050  66.0 
     50.799         nan 
     25.180         nan 
      3.620         nan 
 
 BW-05.8.Lib_L_27F-050  80.0 
     56.487         nan 
     28.880         nan 
      3.760         nan 
 
 BW-05.8.Lib_L_27F-050  94.0 
     61.309         nan 
     31.510         nan 
      3.791         nan 
 
 BW-05.8.Lib_L_27F-050  108.0 
     65.015         nan 
     33.780         nan 
      3.848         nan 
 
 BW-05.8.Lib_L_27F-050  122.0 
     72.160         nan 
     36.780         nan 
      3.906         nan 
 
 BW-05.8.Lib_L_27F-050  136.0 
     74.976         nan 
     38.770         nan 
      3.934         nan 
 
 BW-05.8.Lib_L_27F-050  150.0 
     83.167         nan 
     41.000         nan 
      3.966         nan 
 
 BW-05.9.Lib_L_27F-051  10.0 
     10.213         nan 
      5.430         nan 
      2.053         nan 
 
 BW-05.9.Lib_L_27F-051  24.0 
     26.845         nan 
     10.180         nan 
      2.590         nan 
 
 BW-05.9.Lib_L_27F-051  38.0 
     37.753         nan 
     14.170         nan 
      2.803         nan 
 
 BW-05.9.Lib_L_27F-051  52.0 
     49.701         nan 
     17.610         nan 
      2.965         nan 
 
 BW-05.9.Lib_L_27F-051  66.0 
     55.694         nan 
     20.780         nan 
      3.055         nan 
 
 BW-05.9.Lib_L_27F-051  80.0 
     54.253         nan 
     22.750         nan 
      3.076         nan 
 
 BW-05.9.Lib_L_27F-051  94.0 
     63.275         nan 
     25.920         nan 
      3.140         nan 
 
 BW-05.9.Lib_L_27F-051  108.0 
     68.530         nan 
     28.310         nan 
      3.173         nan 
 
 BW-05.9.Lib_L_27F-051  122.0 
     66.725         nan 
     30.570         nan 
      3.219         nan 
 
 BW-05.9.Lib_L_27F-051  136.0 
     68.202         nan 
     33.020         nan 
      3.265         nan 
 
 BW-05.9.Lib_L_27F-051  150.0 
     68.000         nan 
     35.000         nan 
      3.286         nan 
 
 BW-06.1.Lib_L_27F-001  10.0 
     15.532         nan 
      7.160         nan 
      2.659         nan 
 
 BW-06.1.Lib_L_27F-001  24.0 
     30.428         nan 
     13.000         nan 
      3.328         nan 
 
 BW-06.1.Lib_L_27F-001  38.0 
     44.470         nan 
     17.290         nan 
      3.569         nan 
 
 BW-06.1.Lib_L_27F-001  52.0 
     52.202         nan 
     21.330         nan 
      3.765         nan 
 
 BW-06.1.Lib_L_27F-001  66.0 
     64.608         nan 
     24.910         nan 
      3.884         nan 
 
 BW-06.1.Lib_L_27F-001  80.0 
     84.138         nan 
     27.500         nan 
      3.917         nan 
 
 BW-06.1.Lib_L_27F-001  94.0 
     86.573         nan 
     30.740         nan 
      3.992         nan 
 
 BW-06.1.Lib_L_27F-001  108.0 
    110.394         nan 
     33.990         nan 
      4.060         nan 
 
 BW-06.1.Lib_L_27F-001  122.0 
    109.400         nan 
     36.370         nan 
      4.096         nan 
 
 BW-06.1.Lib_L_27F-001  136.0 
    120.975         nan 
     39.450         nan 
      4.153         nan 
 
 BW-06.1.Lib_L_27F-001  150.0 
    117.600         nan 
     42.000         nan 
      4.180         nan 
 
 BW-06.10.Lib_L_27F-009  10.0 
     10.477         nan 
      6.160         nan 
      2.398         nan 
 
 BW-06.10.Lib_L_27F-009  24.0 
     25.513         nan 
     10.700         nan 
      2.901         nan 
 
 BW-06.10.Lib_L_27F-009  38.0 
     36.883         nan 
     14.310         nan 
      3.130         nan 
 
 BW-06.10.Lib_L_27F-009  52.0 
     51.370         nan 
     17.330         nan 
      3.242         nan 
 
 BW-06.10.Lib_L_27F-009  66.0 
     77.456         nan 
     20.330         nan 
      3.330         nan 
 
 BW-06.10.Lib_L_27F-009  80.0 
    101.629         nan 
     23.160         nan 
      3.378         nan 
 
 BW-06.10.Lib_L_27F-009  94.0 
    122.632         nan 
     26.110         nan 
      3.447         nan 
 
 BW-06.10.Lib_L_27F-009  108.0 
    134.528         nan 
     29.060         nan 
      3.498         nan 
 
 BW-06.10.Lib_L_27F-009  122.0 
    147.178         nan 
     31.770         nan 
      3.539         nan 
 
 BW-06.10.Lib_L_27F-009  136.0 
    129.858         nan 
     34.310         nan 
      3.571         nan 
 
 BW-06.10.Lib_L_27F-009  150.0 
    131.500         nan 
     37.000         nan 
      3.601         nan 
 
 BW-06.2.Lib_L_27F-002  10.0 
     15.465         nan 
      6.320         nan 
      2.334         nan 
 
 BW-06.2.Lib_L_27F-002  24.0 
     29.473         nan 
     11.910         nan 
      2.916         nan 
 
 BW-06.2.Lib_L_27F-002  38.0 
     35.781         nan 
     15.940         nan 
      3.109         nan 
 
 BW-06.2.Lib_L_27F-002  52.0 
     36.383         nan 
     19.330         nan 
      3.293         nan 
 
 BW-06.2.Lib_L_27F-002  66.0 
     42.324         nan 
     22.410         nan 
      3.419         nan 
 
 BW-06.2.Lib_L_27F-002  80.0 
     49.544         nan 
     24.770         nan 
      3.461         nan 
 
 BW-06.2.Lib_L_27F-002  94.0 
     53.867         nan 
     26.950         nan 
      3.525         nan 
 
 BW-06.2.Lib_L_27F-002  108.0 
     64.082         nan 
     28.910         nan 
      3.543         nan 
 
 BW-06.2.Lib_L_27F-002  122.0 
     73.457         nan 
     31.330         nan 
      3.600         nan 
 
 BW-06.2.Lib_L_27F-002  136.0 
     76.466         nan 
     33.000         nan 
      3.640         nan 
 
 BW-06.2.Lib_L_27F-002  150.0 
     82.500         nan 
     35.000         nan 
      3.666         nan 
 
 BW-06.4.Lib_L_27F-003  10.0 
     14.473         nan 
      6.190         nan 
      2.267         nan 
 
 BW-06.4.Lib_L_27F-003  24.0 
     33.862         nan 
     11.820         nan 
      2.817         nan 
 
 BW-06.4.Lib_L_27F-003  38.0 
     48.630         nan 
     17.400         nan 
      3.183         nan 
 
 BW-06.4.Lib_L_27F-003  52.0 
     66.940         nan 
     21.350         nan 
      3.293         nan 
 
 BW-06.4.Lib_L_27F-003  66.0 
     79.168         nan 
     25.470         nan 
      3.371         nan 
 
 BW-06.4.Lib_L_27F-003  80.0 
     87.994         nan 
     29.380         nan 
      3.508         nan 
 
 BW-06.4.Lib_L_27F-003  94.0 
     95.356         nan 
     32.650         nan 
      3.542         nan 
 
 BW-06.4.Lib_L_27F-003  108.0 
    118.150         nan 
     36.470         nan 
      3.624         nan 
 
 BW-06.4.Lib_L_27F-003  122.0 
    120.714         nan 
     39.690         nan 
      3.661         nan 
 
 BW-06.4.Lib_L_27F-003  136.0 
    124.288         nan 
     42.500         nan 
      3.687         nan 
 
 BW-06.4.Lib_L_27F-003  150.0 
    134.000         nan 
     46.000         nan 
      3.741         nan 
 
 BW-06.5.Lib_L_27F-004  10.0 
     13.767         nan 
      6.730         nan 
      2.539         nan 
 
 BW-06.5.Lib_L_27F-004  24.0 
     23.862         nan 
     11.790         nan 
      3.128         nan 
 
 BW-06.5.Lib_L_27F-004  38.0 
     37.142         nan 
     16.030         nan 
      3.406         nan 
 
 BW-06.5.Lib_L_27F-004  52.0 
     49.042         nan 
     19.400         nan 
      3.537         nan 
 
 BW-06.5.Lib_L_27F-004  66.0 
     60.692         nan 
     22.270         nan 
      3.609         nan 
 
 BW-06.5.Lib_L_27F-004  80.0 
     63.530         nan 
     24.650         nan 
      3.645         nan 
 
 BW-06.5.Lib_L_27F-004  94.0 
     67.773         nan 
     27.730         nan 
      3.753         nan 
 
 BW-06.5.Lib_L_27F-004  108.0 
     71.154         nan 
     30.090         nan 
      3.776         nan 
 
 BW-06.5.Lib_L_27F-004  122.0 
     73.713         nan 
     32.640         nan 
      3.830         nan 
 
 BW-06.5.Lib_L_27F-004  136.0 
     76.730         nan 
     34.790         nan 
      3.850         nan 
 
 BW-06.5.Lib_L_27F-004  150.0 
     79.167         nan 
     37.000         nan 
      3.880         nan 
 
 BW-06.6.Lib_L_27F-005  10.0 
     15.015         nan 
      6.510         nan 
      2.415         nan 
 
 BW-06.6.Lib_L_27F-005  24.0 
     32.675         nan 
     12.410         nan 
      3.043         nan 
 
 BW-06.6.Lib_L_27F-005  38.0 
     50.551         nan 
     17.510         nan 
      3.314         nan 
 
 BW-06.6.Lib_L_27F-005  52.0 
     78.965         nan 
     21.870         nan 
      3.499         nan 
 
 BW-06.6.Lib_L_27F-005  66.0 
    100.024         nan 
     25.570         nan 
      3.541         nan 
 
 BW-06.6.Lib_L_27F-005  80.0 
    117.601         nan 
     29.610         nan 
      3.676         nan 
 
 BW-06.6.Lib_L_27F-005  94.0 
    133.617         nan 
     32.610         nan 
      3.705         nan 
 
 BW-06.6.Lib_L_27F-005  108.0 
    150.303         nan 
     35.980         nan 
      3.750         nan 
 
 BW-06.6.Lib_L_27F-005  122.0 
    197.948         nan 
     40.270         nan 
      3.838         nan 
 
 BW-06.6.Lib_L_27F-005  136.0 
    198.844         nan 
     43.580         nan 
      3.879         nan 
 
 BW-06.6.Lib_L_27F-005  150.0 
    204.500         nan 
     47.000         nan 
      3.922         nan 
 
 BW-06.7.Lib_L_27F-006  10.0 
     17.109         nan 
      7.060         nan 
      2.611         nan 
 
 BW-06.7.Lib_L_27F-006  24.0 
     27.517         nan 
     12.710         nan 
      3.226         nan 
 
 BW-06.7.Lib_L_27F-006  38.0 
     34.517         nan 
     16.950         nan 
      3.495         nan 
 
 BW-06.7.Lib_L_27F-006  52.0 
     49.214         nan 
     21.520         nan 
      3.706         nan 
 
 BW-06.7.Lib_L_27F-006  66.0 
     58.259         nan 
     24.440         nan 
      3.785         nan 
 
 BW-06.7.Lib_L_27F-006  80.0 
     62.204         nan 
     27.510         nan 
      3.892         nan 
 
 BW-06.7.Lib_L_27F-006  94.0 
     62.912         nan 
     30.060         nan 
      3.921         nan 
 
 BW-06.7.Lib_L_27F-006  108.0 
     69.397         nan 
     33.160         nan 
      3.998         nan 
 
 BW-06.7.Lib_L_27F-006  122.0 
     71.051         nan 
     35.340         nan 
      4.034         nan 
 
 BW-06.7.Lib_L_27F-006  136.0 
     70.954         nan 
     37.700         nan 
      4.071         nan 
 
 BW-06.7.Lib_L_27F-006  150.0 
     71.625         nan 
     40.000         nan 
      4.102         nan 
 
 BW-06.8.Lib_L_27F-007  10.0 
     17.697         nan 
      6.920         nan 
      2.529         nan 
 
 BW-06.8.Lib_L_27F-007  24.0 
     35.278         nan 
     13.070         nan 
      3.114         nan 
 
 BW-06.8.Lib_L_27F-007  38.0 
     42.758         nan 
     18.260         nan 
      3.414         nan 
 
 BW-06.8.Lib_L_27F-007  52.0 
     51.121         nan 
     22.530         nan 
      3.571         nan 
 
 BW-06.8.Lib_L_27F-007  66.0 
     59.755         nan 
     26.780         nan 
      3.751         nan 
 
 BW-06.8.Lib_L_27F-007  80.0 
     66.147         nan 
     30.290         nan 
      3.812         nan 
 
 BW-06.8.Lib_L_27F-007  94.0 
     74.883         nan 
     33.390         nan 
      3.877         nan 
 
 BW-06.8.Lib_L_27F-007  108.0 
     74.298         nan 
     35.940         nan 
      3.936         nan 
 
 BW-06.8.Lib_L_27F-007  122.0 
     80.787         nan 
     38.690         nan 
      3.983         nan 
 
 BW-06.8.Lib_L_27F-007  136.0 
     86.099         nan 
     41.290         nan 
      4.013         nan 
 
 BW-06.8.Lib_L_27F-007  150.0 
     90.429         nan 
     44.000         nan 
      4.058         nan 
 
 BW-06.9.Lib_L_27F-008  10.0 
     10.975         nan 
      5.260         nan 
      1.925         nan 
 
 BW-06.9.Lib_L_27F-008  24.0 
     26.552         nan 
      9.800         nan 
      2.343         nan 
 
 BW-06.9.Lib_L_27F-008  38.0 
     40.258         nan 
     14.350         nan 
      2.641         nan 
 
 BW-06.9.Lib_L_27F-008  52.0 
     49.935         nan 
     17.810         nan 
      2.782         nan 
 
 BW-06.9.Lib_L_27F-008  66.0 
     56.583         nan 
     21.000         nan 
      2.851         nan 
 
 BW-06.9.Lib_L_27F-008  80.0 
     65.671         nan 
     24.500         nan 
      2.967         nan 
 
 BW-06.9.Lib_L_27F-008  94.0 
     68.757         nan 
     27.310         nan 
      3.033         nan 
 
 BW-06.9.Lib_L_27F-008  108.0 
     71.127         nan 
     29.500         nan 
      3.057         nan 
 
 BW-06.9.Lib_L_27F-008  122.0 
     73.906         nan 
     32.400         nan 
      3.113         nan 
 
 BW-06.9.Lib_L_27F-008  136.0 
     77.492         nan 
     34.820         nan 
      3.145         nan 
 
 BW-06.9.Lib_L_27F-008  150.0 
     76.429         nan 
     37.000         nan 
      3.171         nan 
 
 BW-07.1.Lib_L_27F-010  10.0 
      5.562         nan 
      4.170         nan 
      1.706         nan 
 
 BW-07.1.Lib_L_27F-010  24.0 
      9.446         nan 
      6.120         nan 
      1.962         nan 
 
 BW-07.1.Lib_L_27F-010  38.0 
     13.665         nan 
      8.140         nan 
      2.161         nan 
 
 BW-07.1.Lib_L_27F-010  52.0 
     15.559         nan 
      9.320         nan 
      2.179         nan 
 
 BW-07.1.Lib_L_27F-010  66.0 
     16.335         nan 
     10.490         nan 
      2.248         nan 
 
 BW-07.1.Lib_L_27F-010  80.0 
     16.594         nan 
     11.650         nan 
      2.287         nan 
 
 BW-07.1.Lib_L_27F-010  94.0 
     17.826         nan 
     12.460         nan 
      2.298         nan 
 
 BW-07.1.Lib_L_27F-010  108.0 
     18.410         nan 
     13.380         nan 
      2.317         nan 
 
 BW-07.1.Lib_L_27F-010  122.0 
     17.283         nan 
     13.960         nan 
      2.341         nan 
 
 BW-07.1.Lib_L_27F-010  136.0 
     17.272         nan 
     14.530         nan 
      2.359         nan 
 
 BW-07.1.Lib_L_27F-010  150.0 
     17.000         nan 
     15.000         nan 
      2.361         nan 
 
 BW-07.10.Lib_L_27F-020  10.0 
      5.733         nan 
      3.780         nan 
      1.411         nan 
 
 BW-07.10.Lib_L_27F-020  24.0 
     10.718         nan 
      6.170         nan 
      1.682         nan 
 
 BW-07.10.Lib_L_27F-020  38.0 
     18.742         nan 
      8.390         nan 
      1.845         nan 
 
 BW-07.10.Lib_L_27F-020  52.0 
     20.599         nan 
      9.690         nan 
      1.871         nan 
 
 BW-07.10.Lib_L_27F-020  66.0 
     26.358         nan 
     11.590         nan 
      1.931         nan 
 
 BW-07.10.Lib_L_27F-020  80.0 
     32.287         nan 
     13.420         nan 
      1.994         nan 
 
 BW-07.10.Lib_L_27F-020  94.0 
     32.575         nan 
     14.450         nan 
      2.002         nan 
 
 BW-07.10.Lib_L_27F-020  108.0 
     37.077         nan 
     15.810         nan 
      2.023         nan 
 
 BW-07.10.Lib_L_27F-020  122.0 
     41.355         nan 
     17.140         nan 
      2.037         nan 
 
 BW-07.10.Lib_L_27F-020  136.0 
     43.964         nan 
     18.990         nan 
      2.091         nan 
 
 BW-07.10.Lib_L_27F-020  150.0 
     46.000         nan 
     20.000         nan 
      2.097         nan 
 
 BW-07.2.Lib_L_27F-011  10.0 
      7.620         nan 
      5.340         nan 
      2.159         nan 
 
 BW-07.2.Lib_L_27F-011  24.0 
     14.279         nan 
      8.500         nan 
      2.587         nan 
 
 BW-07.2.Lib_L_27F-011  38.0 
     24.955         nan 
     11.230         nan 
      2.786         nan 
 
 BW-07.2.Lib_L_27F-011  52.0 
     38.151         nan 
     13.770         nan 
      2.911         nan 
 
 BW-07.2.Lib_L_27F-011  66.0 
     55.998         nan 
     16.080         nan 
      2.989         nan 
 
 BW-07.2.Lib_L_27F-011  80.0 
     68.301         nan 
     18.120         nan 
      3.033         nan 
 
 BW-07.2.Lib_L_27F-011  94.0 
     72.869         nan 
     20.110         nan 
      3.065         nan 
 
 BW-07.2.Lib_L_27F-011  108.0 
     78.530         nan 
     22.400         nan 
      3.112         nan 
 
 BW-07.2.Lib_L_27F-011  122.0 
     80.211         nan 
     24.260         nan 
      3.132         nan 
 
 BW-07.2.Lib_L_27F-011  136.0 
     79.659         nan 
     26.180         nan 
      3.159         nan 
 
 BW-07.2.Lib_L_27F-011  150.0 
     75.500         nan 
     28.000         nan 
      3.177         nan 
 
 BW-07.3.Lib_L_27F-013  10.0 
      5.483         nan 
      3.820         nan 
      1.521         nan 
 
 BW-07.3.Lib_L_27F-013  24.0 
     11.353         nan 
      6.570         nan 
      1.872         nan 
 
 BW-07.3.Lib_L_27F-013  38.0 
     14.868         nan 
      8.290         nan 
      1.955         nan 
 
 BW-07.3.Lib_L_27F-013  52.0 
     17.969         nan 
      9.840         nan 
      1.991         nan 
 
 BW-07.3.Lib_L_27F-013  66.0 
     22.730         nan 
     11.900         nan 
      2.119         nan 
 
 BW-07.3.Lib_L_27F-013  80.0 
     24.409         nan 
     12.770         nan 
      2.130         nan 
 
 BW-07.3.Lib_L_27F-013  94.0 
     28.512         nan 
     13.930         nan 
      2.135         nan 
 
 BW-07.3.Lib_L_27F-013  108.0 
     27.015         nan 
     14.990         nan 
      2.173         nan 
 
 BW-07.3.Lib_L_27F-013  122.0 
     31.962         nan 
     16.060         nan 
      2.193         nan 
 
 BW-07.3.Lib_L_27F-013  136.0 
     34.447         nan 
     16.950         nan 
      2.194         nan 
 
 BW-07.3.Lib_L_27F-013  150.0 
     33.000         nan 
     18.000         nan 
      2.214         nan 
 
 BW-07.4.Lib_L_27F-014  10.0 
      6.113         nan 
      4.410         nan 
      1.781         nan 
 
 BW-07.4.Lib_L_27F-014  24.0 
     12.709         nan 
      7.140         nan 
      2.083         nan 
 
 BW-07.4.Lib_L_27F-014  38.0 
     16.245         nan 
      9.160         nan 
      2.198         nan 
 
 BW-07.4.Lib_L_27F-014  52.0 
     21.481         nan 
     11.590         nan 
      2.354         nan 
 
 BW-07.4.Lib_L_27F-014  66.0 
     27.034         nan 
     13.040         nan 
      2.365         nan 
 
 BW-07.4.Lib_L_27F-014  80.0 
     29.637         nan 
     14.830         nan 
      2.425         nan 
 
 BW-07.4.Lib_L_27F-014  94.0 
     35.030         nan 
     16.260         nan 
      2.459         nan 
 
 BW-07.4.Lib_L_27F-014  108.0 
     35.905         nan 
     17.290         nan 
      2.475         nan 
 
 BW-07.4.Lib_L_27F-014  122.0 
     39.153         nan 
     18.560         nan 
      2.486         nan 
 
 BW-07.4.Lib_L_27F-014  136.0 
     39.120         nan 
     19.710         nan 
      2.505         nan 
 
 BW-07.4.Lib_L_27F-014  150.0 
     37.500         nan 
     21.000         nan 
      2.533         nan 
 
 BW-07.5.Lib_L_27F-015  10.0 
      7.832         nan 
      4.600         nan 
      1.772         nan 
 
 BW-07.5.Lib_L_27F-015  24.0 
     20.337         nan 
      8.260         nan 
      2.235         nan 
 
 BW-07.5.Lib_L_27F-015  38.0 
     30.687         nan 
     11.330         nan 
      2.412         nan 
 
 BW-07.5.Lib_L_27F-015  52.0 
     31.995         nan 
     13.500         nan 
      2.469         nan 
 
 BW-07.5.Lib_L_27F-015  66.0 
     38.764         nan 
     15.820         nan 
      2.530         nan 
 
 BW-07.5.Lib_L_27F-015  80.0 
     46.051         nan 
     18.580         nan 
      2.619         nan 
 
 BW-07.5.Lib_L_27F-015  94.0 
     51.843         nan 
     20.270         nan 
      2.628         nan 
 
 BW-07.5.Lib_L_27F-015  108.0 
     62.164         nan 
     22.530         nan 
      2.700         nan 
 
 BW-07.5.Lib_L_27F-015  122.0 
     65.828         nan 
     24.260         nan 
      2.704         nan 
 
 BW-07.5.Lib_L_27F-015  136.0 
     78.912         nan 
     26.210         nan 
      2.744         nan 
 
 BW-07.5.Lib_L_27F-015  150.0 
     85.000         nan 
     28.000         nan 
      2.763         nan 
 
 BW-07.6.Lib_L_27F-016  10.0 
      4.455         nan 
      3.540         nan 
      1.475         nan 
 
 BW-07.6.Lib_L_27F-016  24.0 
     10.910         nan 
      5.680         nan 
      1.695         nan 
 
 BW-07.6.Lib_L_27F-016  38.0 
     19.685         nan 
      7.650         nan 
      1.803         nan 
 
 BW-07.6.Lib_L_27F-016  52.0 
     29.402         nan 
      9.730         nan 
      1.889         nan 
 
 BW-07.6.Lib_L_27F-016  66.0 
     33.100         nan 
     11.530         nan 
      1.944         nan 
 
 BW-07.6.Lib_L_27F-016  80.0 
     41.008         nan 
     13.480         nan 
      2.000         nan 
 
 BW-07.6.Lib_L_27F-016  94.0 
     39.227         nan 
     14.600         nan 
      1.988         nan 
 
 BW-07.6.Lib_L_27F-016  108.0 
     42.460         nan 
     16.440         nan 
      2.025         nan 
 
 BW-07.6.Lib_L_27F-016  122.0 
     47.444         nan 
     18.040         nan 
      2.060         nan 
 
 BW-07.6.Lib_L_27F-016  136.0 
     46.824         nan 
     19.570         nan 
      2.078         nan 
 
 BW-07.6.Lib_L_27F-016  150.0 
     47.250         nan 
     21.000         nan 
      2.097         nan 
 
 BW-07.7.Lib_L_27F-017  10.0 
      4.982         nan 
      3.890         nan 
      1.605         nan 
 
 BW-07.7.Lib_L_27F-017  24.0 
     11.891         nan 
      6.500         nan 
      1.948         nan 
 
 BW-07.7.Lib_L_27F-017  38.0 
     19.286         nan 
      8.620         nan 
      2.079         nan 
 
 BW-07.7.Lib_L_27F-017  52.0 
     23.233         nan 
     10.300         nan 
      2.130         nan 
 
 BW-07.7.Lib_L_27F-017  66.0 
     29.485         nan 
     12.110         nan 
      2.184         nan 
 
 BW-07.7.Lib_L_27F-017  80.0 
     36.316         nan 
     13.760         nan 
      2.249         nan 
 
 BW-07.7.Lib_L_27F-017  94.0 
     42.967         nan 
     15.400         nan 
      2.272         nan 
 
 BW-07.7.Lib_L_27F-017  108.0 
     44.218         nan 
     17.050         nan 
      2.306         nan 
 
 BW-07.7.Lib_L_27F-017  122.0 
     47.101         nan 
     18.460         nan 
      2.321         nan 
 
 BW-07.7.Lib_L_27F-017  136.0 
     44.197         nan 
     19.600         nan 
      2.328         nan 
 
 BW-07.7.Lib_L_27F-017  150.0 
     43.750         nan 
     21.000         nan 
      2.347         nan 
 
 BW-07.8.Lib_L_27F-018  10.0 
      4.038         nan 
      3.420         nan 
      1.418         nan 
 
 BW-07.8.Lib_L_27F-018  24.0 
      7.398         nan 
      5.050         nan 
      1.626         nan 
 
 BW-07.8.Lib_L_27F-018  38.0 
     10.110         nan 
      6.570         nan 
      1.800         nan 
 
 BW-07.8.Lib_L_27F-018  52.0 
     11.784         nan 
      7.620         nan 
      1.836         nan 
 
 BW-07.8.Lib_L_27F-018  66.0 
     15.523         nan 
      8.770         nan 
      1.863         nan 
 
 BW-07.8.Lib_L_27F-018  80.0 
     19.188         nan 
      9.720         nan 
      1.891         nan 
 
 BW-07.8.Lib_L_27F-018  94.0 
     20.268         nan 
     10.560         nan 
      1.910         nan 
 
 BW-07.8.Lib_L_27F-018  108.0 
     22.808         nan 
     11.640         nan 
      1.947         nan 
 
 BW-07.8.Lib_L_27F-018  122.0 
     25.982         nan 
     12.610         nan 
      1.965         nan 
 
 BW-07.8.Lib_L_27F-018  136.0 
     23.697         nan 
     13.200         nan 
      1.969         nan 
 
 BW-07.8.Lib_L_27F-018  150.0 
     23.333         nan 
     14.000         nan 
      1.981         nan 
 
 BW-07.9.Lib_L_27F-019  10.0 
      6.395         nan 
      4.160         nan 
      1.604         nan 
 
 BW-07.9.Lib_L_27F-019  24.0 
     15.252         nan 
      7.420         nan 
      2.036         nan 
 
 BW-07.9.Lib_L_27F-019  38.0 
     20.322         nan 
      9.640         nan 
      2.145         nan 
 
 BW-07.9.Lib_L_27F-019  52.0 
     22.636         nan 
     11.910         nan 
      2.263         nan 
 
 BW-07.9.Lib_L_27F-019  66.0 
     26.317         nan 
     13.920         nan 
      2.346         nan 
 
 BW-07.9.Lib_L_27F-019  80.0 
     25.749         nan 
     15.140         nan 
      2.365         nan 
 
 BW-07.9.Lib_L_27F-019  94.0 
     28.179         nan 
     16.170         nan 
      2.372         nan 
 
 BW-07.9.Lib_L_27F-019  108.0 
     31.669         nan 
     17.550         nan 
      2.398         nan 
 
 BW-07.9.Lib_L_27F-019  122.0 
     35.502         nan 
     18.930         nan 
      2.438         nan 
 
 BW-07.9.Lib_L_27F-019  136.0 
     38.931         nan 
     19.950         nan 
      2.450         nan 
 
 BW-07.9.Lib_L_27F-019  150.0 
     39.333         nan 
     21.000         nan 
      2.468         nan 
 
 BW-08.1.Lib_L_27F-021  10.0 
     17.117         nan 
      7.100         nan 
      2.602         nan 
 
 BW-08.1.Lib_L_27F-021  24.0 
     30.468         nan 
     13.190         nan 
      3.206         nan 
 
 BW-08.1.Lib_L_27F-021  38.0 
     40.992         nan 
     18.070         nan 
      3.500         nan 
 
 BW-08.1.Lib_L_27F-021  52.0 
     46.916         nan 
     21.720         nan 
      3.640         nan 
 
 BW-08.1.Lib_L_27F-021  66.0 
     54.114         nan 
     25.580         nan 
      3.776         nan 
 
 BW-08.1.Lib_L_27F-021  80.0 
     54.584         nan 
     28.370         nan 
      3.866         nan 
 
 BW-08.1.Lib_L_27F-021  94.0 
     63.095         nan 
     31.010         nan 
      3.927         nan 
 
 BW-08.1.Lib_L_27F-021  108.0 
     63.488         nan 
     33.360         nan 
      3.966         nan 
 
 BW-08.1.Lib_L_27F-021  122.0 
     63.452         nan 
     35.780         nan 
      4.023         nan 
 
 BW-08.1.Lib_L_27F-021  136.0 
     66.363         nan 
     37.950         nan 
      4.056         nan 
 
 BW-08.1.Lib_L_27F-021  150.0 
     66.250         nan 
     40.000         nan 
      4.087         nan 
 
 BW-08.10.Lib_L_27F-030  10.0 
      9.188         nan 
      4.860         nan 
      1.798         nan 
 
 BW-08.10.Lib_L_27F-030  24.0 
     16.998         nan 
      8.590         nan 
      2.195         nan 
 
 BW-08.10.Lib_L_27F-030  38.0 
     23.071         nan 
     12.060         nan 
      2.475         nan 
 
 BW-08.10.Lib_L_27F-030  52.0 
     27.473         nan 
     14.610         nan 
      2.550         nan 
 
 BW-08.10.Lib_L_27F-030  66.0 
     34.130         nan 
     16.790         nan 
      2.653         nan 
 
 BW-08.10.Lib_L_27F-030  80.0 
     32.725         nan 
     18.340         nan 
      2.667         nan 
 
 BW-08.10.Lib_L_27F-030  94.0 
     35.708         nan 
     20.110         nan 
      2.716         nan 
 
 BW-08.10.Lib_L_27F-030  108.0 
     39.182         nan 
     21.770         nan 
      2.751         nan 
 
 BW-08.10.Lib_L_27F-030  122.0 
     44.372         nan 
     23.240         nan 
      2.762         nan 
 
 BW-08.10.Lib_L_27F-030  136.0 
     45.137         nan 
     24.640         nan 
      2.787         nan 
 
 BW-08.10.Lib_L_27F-030  150.0 
     48.750         nan 
     26.000         nan 
      2.805         nan 
 
 BW-08.2.Lib_L_27F-022  10.0 
     15.145         nan 
      7.140         nan 
      2.667         nan 
 
 BW-08.2.Lib_L_27F-022  24.0 
     24.404         nan 
     13.150         nan 
      3.417         nan 
 
 BW-08.2.Lib_L_27F-022  38.0 
     30.896         nan 
     16.680         nan 
      3.611         nan 
 
 BW-08.2.Lib_L_27F-022  52.0 
     32.296         nan 
     19.710         nan 
      3.770         nan 
 
 BW-08.2.Lib_L_27F-022  66.0 
     39.675         nan 
     22.690         nan 
      3.864         nan 
 
 BW-08.2.Lib_L_27F-022  80.0 
     42.645         nan 
     24.470         nan 
      3.925         nan 
 
 BW-08.2.Lib_L_27F-022  94.0 
     41.256         nan 
     26.210         nan 
      3.966         nan 
 
 BW-08.2.Lib_L_27F-022  108.0 
     46.421         nan 
     28.600         nan 
      4.026         nan 
 
 BW-08.2.Lib_L_27F-022  122.0 
     45.155         nan 
     30.220         nan 
      4.065         nan 
 
 BW-08.2.Lib_L_27F-022  136.0 
     44.938         nan 
     31.590         nan 
      4.088         nan 
 
 BW-08.2.Lib_L_27F-022  150.0 
     44.375         nan 
     33.000         nan 
      4.113         nan 
 
 BW-08.3.Lib_L_27F-023  10.0 
     16.553         nan 
      7.380         nan 
      2.714         nan 
 
 BW-08.3.Lib_L_27F-023  24.0 
     30.223         nan 
     13.790         nan 
      3.473         nan 
 
 BW-08.3.Lib_L_27F-023  38.0 
     46.002         nan 
     18.350         nan 
      3.733         nan 
 
 BW-08.3.Lib_L_27F-023  52.0 
     58.554         nan 
     22.270         nan 
      3.885         nan 
 
 BW-08.3.Lib_L_27F-023  66.0 
     74.299         nan 
     26.090         nan 
      4.029         nan 
 
 BW-08.3.Lib_L_27F-023  80.0 
     84.936         nan 
     28.970         nan 
      4.081         nan 
 
 BW-08.3.Lib_L_27F-023  94.0 
     91.991         nan 
     32.310         nan 
      4.146         nan 
 
 BW-08.3.Lib_L_27F-023  108.0 
    104.275         nan 
     35.730         nan 
      4.229         nan 
 
 BW-08.3.Lib_L_27F-023  122.0 
    107.468         nan 
     38.650         nan 
      4.268         nan 
 
 BW-08.3.Lib_L_27F-023  136.0 
    109.182         nan 
     41.340         nan 
      4.304         nan 
 
 BW-08.3.Lib_L_27F-023  150.0 
    111.667         nan 
     44.000         nan 
      4.336         nan 
 
 BW-08.4.Lib_L_27F-024  10.0 
     18.070         nan 
      7.120         nan 
      2.592         nan 
 
 BW-08.4.Lib_L_27F-024  24.0 
     37.853         nan 
     13.810         nan 
      3.298         nan 
 
 BW-08.4.Lib_L_27F-024  38.0 
     44.224         nan 
     18.880         nan 
      3.557         nan 
 
 BW-08.4.Lib_L_27F-024  52.0 
     52.066         nan 
     23.700         nan 
      3.767         nan 
 
 BW-08.4.Lib_L_27F-024  66.0 
     53.714         nan 
     27.450         nan 
      3.918         nan 
 
 BW-08.4.Lib_L_27F-024  80.0 
     58.422         nan 
     30.760         nan 
      4.008         nan 
 
 BW-08.4.Lib_L_27F-024  94.0 
     66.659         nan 
     33.790         nan 
      4.058         nan 
 
 BW-08.4.Lib_L_27F-024  108.0 
     68.659         nan 
     36.370         nan 
      4.110         nan 
 
 BW-08.4.Lib_L_27F-024  122.0 
     72.622         nan 
     39.060         nan 
      4.151         nan 
 
 BW-08.4.Lib_L_27F-024  136.0 
     75.502         nan 
     41.730         nan 
      4.206         nan 
 
 BW-08.4.Lib_L_27F-024  150.0 
     74.667         nan 
     44.000         nan 
      4.238         nan 
 
 BW-08.5.Lib_L_27F-025  10.0 
     10.461         nan 
      6.120         nan 
      2.385         nan 
 
 BW-08.5.Lib_L_27F-025  24.0 
     24.500         nan 
     10.770         nan 
      2.961         nan 
 
 BW-08.5.Lib_L_27F-025  38.0 
     43.676         nan 
     14.490         nan 
      3.177         nan 
 
 BW-08.5.Lib_L_27F-025  52.0 
     65.349         nan 
     17.860         nan 
      3.299         nan 
 
 BW-08.5.Lib_L_27F-025  66.0 
     74.047         nan 
     21.190         nan 
      3.411         nan 
 
 BW-08.5.Lib_L_27F-025  80.0 
     87.627         nan 
     24.170         nan 
      3.468         nan 
 
 BW-08.5.Lib_L_27F-025  94.0 
     89.062         nan 
     26.700         nan 
      3.509         nan 
 
 BW-08.5.Lib_L_27F-025  108.0 
     90.492         nan 
     30.100         nan 
      3.595         nan 
 
 BW-08.5.Lib_L_27F-025  122.0 
     96.998         nan 
     32.700         nan 
      3.615         nan 
 
 BW-08.5.Lib_L_27F-025  136.0 
     97.970         nan 
     35.680         nan 
      3.671         nan 
 
 BW-08.5.Lib_L_27F-025  150.0 
     96.500         nan 
     38.000         nan 
      3.692         nan 
 
 BW-08.6.Lib_L_27F-026  10.0 
      3.740         nan 
      2.710         nan 
      0.814         nan 
 
 BW-08.6.Lib_L_27F-026  24.0 
      8.182         nan 
      4.820         nan 
      1.075         nan 
 
 BW-08.6.Lib_L_27F-026  38.0 
     12.676         nan 
      6.560         nan 
      1.149         nan 
 
 BW-08.6.Lib_L_27F-026  52.0 
     14.993         nan 
      7.900         nan 
      1.209         nan 
 
 BW-08.6.Lib_L_27F-026  66.0 
     18.054         nan 
      8.930         nan 
      1.213         nan 
 
 BW-08.6.Lib_L_27F-026  80.0 
     21.488         nan 
     10.100         nan 
      1.235         nan 
 
 BW-08.6.Lib_L_27F-026  94.0 
     23.032         nan 
     10.980         nan 
      1.253         nan 
 
 BW-08.6.Lib_L_27F-026  108.0 
     28.791         nan 
     12.040         nan 
      1.283         nan 
 
 BW-08.6.Lib_L_27F-026  122.0 
     30.985         nan 
     13.010         nan 
      1.290         nan 
 
 BW-08.6.Lib_L_27F-026  136.0 
     35.042         nan 
     14.080         nan 
      1.299         nan 
 
 BW-08.6.Lib_L_27F-026  150.0 
     37.500         nan 
     15.000         nan 
      1.312         nan 
 
 BW-08.7.Lib_L_27F-027  10.0 
      5.038         nan 
      3.300         nan 
      1.099         nan 
 
 BW-08.7.Lib_L_27F-027  24.0 
      9.440         nan 
      5.540         nan 
      1.310         nan 
 
 BW-08.7.Lib_L_27F-027  38.0 
     14.648         nan 
      7.790         nan 
      1.476         nan 
 
 BW-08.7.Lib_L_27F-027  52.0 
     16.232         nan 
      9.040         nan 
      1.472         nan 
 
 BW-08.7.Lib_L_27F-027  66.0 
     19.179         nan 
     10.230         nan 
      1.514         nan 
 
 BW-08.7.Lib_L_27F-027  80.0 
     23.176         nan 
     11.600         nan 
      1.551         nan 
 
 BW-08.7.Lib_L_27F-027  94.0 
     29.059         nan 
     13.140         nan 
      1.631         nan 
 
 BW-08.7.Lib_L_27F-027  108.0 
     34.521         nan 
     13.990         nan 
      1.629         nan 
 
 BW-08.7.Lib_L_27F-027  122.0 
     39.833         nan 
     14.800         nan 
      1.634         nan 
 
 BW-08.7.Lib_L_27F-027  136.0 
     52.498         nan 
     16.100         nan 
      1.657         nan 
 
 BW-08.7.Lib_L_27F-027  150.0 
     72.000         nan 
     17.000         nan 
      1.661         nan 
 
 BW-08.8.Lib_L_27F-028  10.0 
      4.420         nan 
      3.070         nan 
      0.988         nan 
 
 BW-08.8.Lib_L_27F-028  24.0 
      9.512         nan 
      5.320         nan 
      1.252         nan 
 
 BW-08.8.Lib_L_27F-028  38.0 
     13.267         nan 
      7.420         nan 
      1.411         nan 
 
 BW-08.8.Lib_L_27F-028  52.0 
     17.756         nan 
      9.000         nan 
      1.482         nan 
 
 BW-08.8.Lib_L_27F-028  66.0 
     21.848         nan 
     10.400         nan 
      1.521         nan 
 
 BW-08.8.Lib_L_27F-028  80.0 
     23.753         nan 
     11.780         nan 
      1.577         nan 
 
 BW-08.8.Lib_L_27F-028  94.0 
     26.138         nan 
     13.020         nan 
      1.598         nan 
 
 BW-08.8.Lib_L_27F-028  108.0 
     27.015         nan 
     13.920         nan 
      1.599         nan 
 
 BW-08.8.Lib_L_27F-028  122.0 
     30.163         nan 
     15.180         nan 
      1.631         nan 
 
 BW-08.8.Lib_L_27F-028  136.0 
     29.725         nan 
     15.970         nan 
      1.628         nan 
 
 BW-08.8.Lib_L_27F-028  150.0 
     32.000         nan 
     17.000         nan 
      1.648         nan 
 
 BW-08.9.Lib_L_27F-029  10.0 
      8.948         nan 
      4.870         nan 
      1.810         nan 
 
 BW-08.9.Lib_L_27F-029  24.0 
     16.279         nan 
      8.750         nan 
      2.300         nan 
 
 BW-08.9.Lib_L_27F-029  38.0 
     22.116         nan 
     11.300         nan 
      2.398         nan 
 
 BW-08.9.Lib_L_27F-029  52.0 
     25.757         nan 
     13.810         nan 
      2.539         nan 
 
 BW-08.9.Lib_L_27F-029  66.0 
     30.626         nan 
     15.840         nan 
      2.597         nan 
 
 BW-08.9.Lib_L_27F-029  80.0 
     33.467         nan 
     17.940         nan 
      2.663         nan 
 
 BW-08.9.Lib_L_27F-029  94.0 
     34.869         nan 
     19.390         nan 
      2.688         nan 
 
 BW-08.9.Lib_L_27F-029  108.0 
     37.808         nan 
     20.970         nan 
      2.732         nan 
 
 BW-08.9.Lib_L_27F-029  122.0 
     39.360         nan 
     22.460         nan 
      2.770         nan 
 
 BW-08.9.Lib_L_27F-029  136.0 
     40.891         nan 
     23.810         nan 
      2.790         nan 
 
 BW-08.9.Lib_L_27F-029  150.0 
     40.600         nan 
     25.000         nan 
      2.804         nan 
 
 BW-09.1.Lib_L_27F-031  10.0 
     16.725         nan 
      6.580         nan 
      2.393         nan 
 
 BW-09.1.Lib_L_27F-031  24.0 
     50.995         nan 
     13.540         nan 
      3.101         nan 
 
 BW-09.1.Lib_L_27F-031  38.0 
     69.644         nan 
     19.370         nan 
      3.386         nan 
 
 BW-09.1.Lib_L_27F-031  52.0 
     97.193         nan 
     24.970         nan 
      3.601         nan 
 
 BW-09.1.Lib_L_27F-031  66.0 
    100.785         nan 
     29.550         nan 
      3.721         nan 
 
 BW-09.1.Lib_L_27F-031  80.0 
    119.073         nan 
     35.000         nan 
      3.861         nan 
 
 BW-09.1.Lib_L_27F-031  94.0 
    122.575         nan 
     38.570         nan 
      3.886         nan 
 
 BW-09.1.Lib_L_27F-031  108.0 
    115.358         nan 
     42.610         nan 
      3.946         nan 
 
 BW-09.1.Lib_L_27F-031  122.0 
    120.537         nan 
     47.240         nan 
      4.055         nan 
 
 BW-09.1.Lib_L_27F-031  136.0 
    123.891         nan 
     51.300         nan 
      4.105         nan 
 
 BW-09.1.Lib_L_27F-031  150.0 
    122.364         nan 
     55.000         nan 
      4.143         nan 
 
 BW-09.10.Lib_L_27F-040  10.0 
      9.837         nan 
      5.830         nan 
      2.298         nan 
 
 BW-09.10.Lib_L_27F-040  24.0 
     22.681         nan 
     10.180         nan 
      2.845         nan 
 
 BW-09.10.Lib_L_27F-040  38.0 
     31.996         nan 
     12.980         nan 
      2.963         nan 
 
 BW-09.10.Lib_L_27F-040  52.0 
     39.077         nan 
     16.240         nan 
      3.119         nan 
 
 BW-09.10.Lib_L_27F-040  66.0 
     43.285         nan 
     18.880         nan 
      3.217         nan 
 
 BW-09.10.Lib_L_27F-040  80.0 
     49.744         nan 
     21.230         nan 
      3.261         nan 
 
 BW-09.10.Lib_L_27F-040  94.0 
     53.213         nan 
     23.750         nan 
      3.324         nan 
 
 BW-09.10.Lib_L_27F-040  108.0 
     54.041         nan 
     25.330         nan 
      3.332         nan 
 
 BW-09.10.Lib_L_27F-040  122.0 
     52.554         nan 
     27.320         nan 
      3.376         nan 
 
 BW-09.10.Lib_L_27F-040  136.0 
     53.978         nan 
     29.270         nan 
      3.409         nan 
 
 BW-09.10.Lib_L_27F-040  150.0 
     52.857         nan 
     31.000         nan 
      3.430         nan 
 
 BW-09.2.Lib_L_27F-032  10.0 
      5.420         nan 
      3.490         nan 
      1.214         nan 
 
 BW-09.2.Lib_L_27F-032  24.0 
     10.704         nan 
      6.180         nan 
      1.559         nan 
 
 BW-09.2.Lib_L_27F-032  38.0 
     16.741         nan 
      8.150         nan 
      1.691         nan 
 
 BW-09.2.Lib_L_27F-032  52.0 
     23.415         nan 
      9.720         nan 
      1.708         nan 
 
 BW-09.2.Lib_L_27F-032  66.0 
     27.953         nan 
     11.350         nan 
      1.765         nan 
 
 BW-09.2.Lib_L_27F-032  80.0 
     32.678         nan 
     12.940         nan 
      1.798         nan 
 
 BW-09.2.Lib_L_27F-032  94.0 
     33.210         nan 
     14.200         nan 
      1.827         nan 
 
 BW-09.2.Lib_L_27F-032  108.0 
     30.402         nan 
     15.560         nan 
      1.852         nan 
 
 BW-09.2.Lib_L_27F-032  122.0 
     30.025         nan 
     17.030         nan 
      1.899         nan 
 
 BW-09.2.Lib_L_27F-032  136.0 
     28.215         nan 
     17.850         nan 
      1.896         nan 
 
 BW-09.2.Lib_L_27F-032  150.0 
     28.167         nan 
     19.000         nan 
      1.909         nan 
 
 BW-09.3.Lib_L_27F-033  10.0 
     13.837         nan 
      6.730         nan 
      2.534         nan 
 
 BW-09.3.Lib_L_27F-033  24.0 
     26.159         nan 
     11.780         nan 
      3.087         nan 
 
 BW-09.3.Lib_L_27F-033  38.0 
     44.390         nan 
     15.860         nan 
      3.318         nan 
 
 BW-09.3.Lib_L_27F-033  52.0 
     56.828         nan 
     19.550         nan 
      3.472         nan 
 
 BW-09.3.Lib_L_27F-033  66.0 
     66.855         nan 
     22.860         nan 
      3.591         nan 
 
 BW-09.3.Lib_L_27F-033  80.0 
     86.093         nan 
     25.650         nan 
      3.630         nan 
 
 BW-09.3.Lib_L_27F-033  94.0 
    108.871         nan 
     29.210         nan 
      3.725         nan 
 
 BW-09.3.Lib_L_27F-033  108.0 
    125.489         nan 
     32.720         nan 
      3.814         nan 
 
 BW-09.3.Lib_L_27F-033  122.0 
    152.663         nan 
     35.340         nan 
      3.837         nan 
 
 BW-09.3.Lib_L_27F-033  136.0 
    153.102         nan 
     38.210         nan 
      3.871         nan 
 
 BW-09.3.Lib_L_27F-033  150.0 
    149.750         nan 
     41.000         nan 
      3.903         nan 
 
 BW-09.4.Lib_L_27F-034  10.0 
      9.366         nan 
      5.910         nan 
      2.331         nan 
 
 BW-09.4.Lib_L_27F-034  24.0 
     18.758         nan 
      9.440         nan 
      2.739         nan 
 
 BW-09.4.Lib_L_27F-034  38.0 
     33.757         nan 
     12.960         nan 
      3.003         nan 
 
 BW-09.4.Lib_L_27F-034  52.0 
     39.204         nan 
     15.120         nan 
      3.063         nan 
 
 BW-09.4.Lib_L_27F-034  66.0 
     52.306         nan 
     17.760         nan 
      3.136         nan 
 
 BW-09.4.Lib_L_27F-034  80.0 
     60.237         nan 
     20.180         nan 
      3.201         nan 
 
 BW-09.4.Lib_L_27F-034  94.0 
     61.613         nan 
     22.330         nan 
      3.247         nan 
 
 BW-09.4.Lib_L_27F-034  108.0 
     64.451         nan 
     24.330         nan 
      3.281         nan 
 
 BW-09.4.Lib_L_27F-034  122.0 
     73.414         nan 
     27.240         nan 
      3.352         nan 
 
 BW-09.4.Lib_L_27F-034  136.0 
     82.167         nan 
     29.130         nan 
      3.369         nan 
 
 BW-09.4.Lib_L_27F-034  150.0 
     83.500         nan 
     31.000         nan 
      3.390         nan 
 
 BW-09.5.Lib_L_27F-035  10.0 
      9.638         nan 
      5.190         nan 
      1.972         nan 
 
 BW-09.5.Lib_L_27F-035  24.0 
     20.556         nan 
      9.290         nan 
      2.443         nan 
 
 BW-09.5.Lib_L_27F-035  38.0 
     32.317         nan 
     12.540         nan 
      2.623         nan 
 
 BW-09.5.Lib_L_27F-035  52.0 
     42.125         nan 
     15.560         nan 
      2.764         nan 
 
 BW-09.5.Lib_L_27F-035  66.0 
     43.060         nan 
     18.200         nan 
      2.847         nan 
 
 BW-09.5.Lib_L_27F-035  80.0 
     55.389         nan 
     20.430         nan 
      2.869         nan 
 
 BW-09.5.Lib_L_27F-035  94.0 
     62.730         nan 
     22.630         nan 
      2.909         nan 
 
 BW-09.5.Lib_L_27F-035  108.0 
     69.912         nan 
     24.830         nan 
      2.955         nan 
 
 BW-09.5.Lib_L_27F-035  122.0 
     79.295         nan 
     27.140         nan 
      2.991         nan 
 
 BW-09.5.Lib_L_27F-035  136.0 
     80.463         nan 
     29.030         nan 
      3.015         nan 
 
 BW-09.5.Lib_L_27F-035  150.0 
     83.500         nan 
     31.000         nan 
      3.040         nan 
 
 BW-09.6.Lib_L_27F-036  10.0 
     15.749         nan 
      7.160         nan 
      2.659         nan 
 
 BW-09.6.Lib_L_27F-036  24.0 
     33.478         nan 
     13.160         nan 
      3.346         nan 
 
 BW-09.6.Lib_L_27F-036  38.0 
     54.986         nan 
     18.020         nan 
      3.626         nan 
 
 BW-09.6.Lib_L_27F-036  52.0 
     71.788         nan 
     22.400         nan 
      3.791         nan 
 
 BW-09.6.Lib_L_27F-036  66.0 
     83.261         nan 
     26.730         nan 
      3.939         nan 
 
 BW-09.6.Lib_L_27F-036  80.0 
     86.167         nan 
     30.470         nan 
      4.021         nan 
 
 BW-09.6.Lib_L_27F-036  94.0 
     99.409         nan 
     34.050         nan 
      4.102         nan 
 
 BW-09.6.Lib_L_27F-036  108.0 
     98.305         nan 
     37.910         nan 
      4.178         nan 
 
 BW-09.6.Lib_L_27F-036  122.0 
     97.411         nan 
     40.860         nan 
      4.213         nan 
 
 BW-09.6.Lib_L_27F-036  136.0 
     99.605         nan 
     44.000         nan 
      4.263         nan 
 
 BW-09.6.Lib_L_27F-036  150.0 
     98.667         nan 
     47.000         nan 
      4.300         nan 
 
 BW-09.7.Lib_L_27F-037  10.0 
     10.075         nan 
      5.190         nan 
      1.932         nan 
 
 BW-09.7.Lib_L_27F-037  24.0 
     22.313         nan 
      9.420         nan 
      2.384         nan 
 
 BW-09.7.Lib_L_27F-037  38.0 
     34.067         nan 
     13.010         nan 
      2.612         nan 
 
 BW-09.7.Lib_L_27F-037  52.0 
     41.807         nan 
     16.040         nan 
      2.698         nan 
 
 BW-09.7.Lib_L_27F-037  66.0 
     43.242         nan 
     19.060         nan 
      2.835         nan 
 
 BW-09.7.Lib_L_27F-037  80.0 
     54.225         nan 
     21.780         nan 
      2.883         nan 
 
 BW-09.7.Lib_L_27F-037  94.0 
     51.508         nan 
     23.880         nan 
      2.908         nan 
 
 BW-09.7.Lib_L_27F-037  108.0 
     49.886         nan 
     26.300         nan 
      2.972         nan 
 
 BW-09.7.Lib_L_27F-037  122.0 
     50.173         nan 
     28.340         nan 
      2.999         nan 
 
 BW-09.7.Lib_L_27F-037  136.0 
     49.890         nan 
     30.240         nan 
      3.033         nan 
 
 BW-09.7.Lib_L_27F-037  150.0 
     49.000         nan 
     32.000         nan 
      3.055         nan 
 
 BW-09.8.Lib_L_27F-038  10.0 
      9.290         nan 
      4.850         nan 
      1.785         nan 
 
 BW-09.8.Lib_L_27F-038  24.0 
     18.949         nan 
      8.920         nan 
      2.224         nan 
 
 BW-09.8.Lib_L_27F-038  38.0 
     22.768         nan 
     11.620         nan 
      2.352         nan 
 
 BW-09.8.Lib_L_27F-038  52.0 
     29.558         nan 
     14.240         nan 
      2.488         nan 
 
 BW-09.8.Lib_L_27F-038  66.0 
     34.018         nan 
     16.880         nan 
      2.614         nan 
 
 BW-09.8.Lib_L_27F-038  80.0 
     38.558         nan 
     18.210         nan 
      2.585         nan 
 
 BW-09.8.Lib_L_27F-038  94.0 
     46.400         nan 
     20.540         nan 
      2.677         nan 
 
 BW-09.8.Lib_L_27F-038  108.0 
     61.756         nan 
     22.230         nan 
      2.709         nan 
 
 BW-09.8.Lib_L_27F-038  122.0 
     68.864         nan 
     23.710         nan 
      2.720         nan 
 
 BW-09.8.Lib_L_27F-038  136.0 
     70.215         nan 
     25.340         nan 
      2.743         nan 
 
 BW-09.8.Lib_L_27F-038  150.0 
     72.333         nan 
     27.000         nan 
      2.770         nan 
 
 BW-09.9.Lib_L_27F-039  10.0 
      5.642         nan 
      4.140         nan 
      1.695         nan 
 
 BW-09.9.Lib_L_27F-039  24.0 
     16.373         nan 
      7.070         nan 
      2.031         nan 
 
 BW-09.9.Lib_L_27F-039  38.0 
     21.635         nan 
      9.190         nan 
      2.129         nan 
 
 BW-09.9.Lib_L_27F-039  52.0 
     27.839         nan 
     11.380         nan 
      2.226         nan 
 
 BW-09.9.Lib_L_27F-039  66.0 
     36.165         nan 
     13.400         nan 
      2.255         nan 
 
 BW-09.9.Lib_L_27F-039  80.0 
     43.560         nan 
     15.460         nan 
      2.316         nan 
 
 BW-09.9.Lib_L_27F-039  94.0 
     53.736         nan 
     16.860         nan 
      2.316         nan 
 
 BW-09.9.Lib_L_27F-039  108.0 
     56.440         nan 
     19.090         nan 
      2.380         nan 
 
 BW-09.9.Lib_L_27F-039  122.0 
     59.057         nan 
     20.570         nan 
      2.400         nan 
 
 BW-09.9.Lib_L_27F-039  136.0 
     60.434         nan 
     22.490         nan 
      2.429         nan 
 
 BW-09.9.Lib_L_27F-039  150.0 
     58.000         nan 
     24.000         nan 
      2.446         nan 
 
 BW-10.1.Lib_L_27F-041  10.0 
      3.325         nan 
      2.580         nan 
      0.771         nan 
 
 BW-10.1.Lib_L_27F-041  24.0 
      7.463         nan 
      4.670         nan 
      1.082         nan 
 
 BW-10.1.Lib_L_27F-041  38.0 
     10.517         nan 
      6.240         nan 
      1.159         nan 
 
 BW-10.1.Lib_L_27F-041  52.0 
     12.146         nan 
      7.180         nan 
      1.175         nan 
 
 BW-10.1.Lib_L_27F-041  66.0 
     15.303         nan 
      8.320         nan 
      1.211         nan 
 
 BW-10.1.Lib_L_27F-041  80.0 
     15.821         nan 
      9.070         nan 
      1.215         nan 
 
 BW-10.1.Lib_L_27F-041  94.0 
     17.435         nan 
     10.040         nan 
      1.248         nan 
 
 BW-10.1.Lib_L_27F-041  108.0 
     19.300         nan 
     10.890         nan 
      1.253         nan 
 
 BW-10.1.Lib_L_27F-041  122.0 
     19.566         nan 
     11.640         nan 
      1.274         nan 
 
 BW-10.1.Lib_L_27F-041  136.0 
     19.415         nan 
     12.280         nan 
      1.278         nan 
 
 BW-10.1.Lib_L_27F-041  150.0 
     20.000         nan 
     13.000         nan 
      1.290         nan 
 
 BW-10.10.Lib_L_27F-051  10.0 
      3.975         nan 
      3.180         nan 
      1.167         nan 
 
 BW-10.10.Lib_L_27F-051  24.0 
      7.235         nan 
      4.830         nan 
      1.429         nan 
 
 BW-10.10.Lib_L_27F-051  38.0 
     11.448         nan 
      6.370         nan 
      1.480         nan 
 
 BW-10.10.Lib_L_27F-051  52.0 
     17.420         nan 
      7.790         nan 
      1.547         nan 
 
 BW-10.10.Lib_L_27F-051  66.0 
     22.538         nan 
      8.920         nan 
      1.592         nan 
 
 BW-10.10.Lib_L_27F-051  80.0 
     27.545         nan 
     10.150         nan 
      1.609         nan 
 
 BW-10.10.Lib_L_27F-051  94.0 
     33.805         nan 
     11.200         nan 
      1.631         nan 
 
 BW-10.10.Lib_L_27F-051  108.0 
     42.050         nan 
     12.670         nan 
      1.663         nan 
 
 BW-10.10.Lib_L_27F-051  122.0 
     45.340         nan 
     13.750         nan 
      1.684         nan 
 
 BW-10.10.Lib_L_27F-051  136.0 
     48.100         nan 
     14.780         nan 
      1.684         nan 
 
 BW-10.10.Lib_L_27F-051  150.0 
     49.000         nan 
     16.000         nan 
      1.703         nan 
 
 BW-10.2.Lib_L_27F-042  10.0 
     10.530         nan 
      5.760         nan 
      2.222         nan 
 
 BW-10.2.Lib_L_27F-042  24.0 
     26.426         nan 
     10.980         nan 
      2.888         nan 
 
 BW-10.2.Lib_L_27F-042  38.0 
     35.947         nan 
     14.570         nan 
      3.055         nan 
 
 BW-10.2.Lib_L_27F-042  52.0 
     40.835         nan 
     17.630         nan 
      3.127         nan 
 
 BW-10.2.Lib_L_27F-042  66.0 
     52.985         nan 
     20.910         nan 
      3.242         nan 
 
 BW-10.2.Lib_L_27F-042  80.0 
     53.809         nan 
     23.810         nan 
      3.334         nan 
 
 BW-10.2.Lib_L_27F-042  94.0 
     59.119         nan 
     26.260         nan 
      3.383         nan 
 
 BW-10.2.Lib_L_27F-042  108.0 
     62.480         nan 
     28.690         nan 
      3.433         nan 
 
 BW-10.2.Lib_L_27F-042  122.0 
     65.794         nan 
     30.960         nan 
      3.476         nan 
 
 BW-10.2.Lib_L_27F-042  136.0 
     65.461         nan 
     33.020         nan 
      3.509         nan 
 
 BW-10.2.Lib_L_27F-042  150.0 
     70.000         nan 
     35.000         nan 
      3.530         nan 
 
 BW-10.4.Lib_L_27F-044  10.0 
     18.198         nan 
      7.470         nan 
      2.725         nan 
 
 BW-10.4.Lib_L_27F-044  24.0 
     45.545         nan 
     15.170         nan 
      3.585         nan 
 
 BW-10.4.Lib_L_27F-044  38.0 
     62.332         nan 
     20.810         nan 
      3.866         nan 
 
 BW-10.4.Lib_L_27F-044  52.0 
     72.235         nan 
     26.050         nan 
      4.075         nan 
 
 BW-10.4.Lib_L_27F-044  66.0 
     81.436         nan 
     31.240         nan 
      4.237         nan 
 
 BW-10.4.Lib_L_27F-044  80.0 
     81.537         nan 
     35.000         nan 
      4.300         nan 
 
 BW-10.4.Lib_L_27F-044  94.0 
     80.250         nan 
     38.570         nan 
      4.378         nan 
 
 BW-10.4.Lib_L_27F-044  108.0 
     84.923         nan 
     42.890         nan 
      4.478         nan 
 
 BW-10.4.Lib_L_27F-044  122.0 
     86.030         nan 
     45.900         nan 
      4.514         nan 
 
 BW-10.4.Lib_L_27F-044  136.0 
     87.639         nan 
     49.270         nan 
      4.586         nan 
 
 BW-10.4.Lib_L_27F-044  150.0 
     88.250         nan 
     52.000         nan 
      4.616         nan 
 
 BW-10.5.Lib_L_27F-045  10.0 
      7.715         nan 
      4.170         nan 
      1.463         nan 
 
 BW-10.5.Lib_L_27F-045  24.0 
     17.764         nan 
      7.570         nan 
      1.745         nan 
 
 BW-10.5.Lib_L_27F-045  38.0 
     23.618         nan 
     10.520         nan 
      1.917         nan 
 
 BW-10.5.Lib_L_27F-045  52.0 
     33.261         nan 
     13.680         nan 
      2.094         nan 
 
 BW-10.5.Lib_L_27F-045  66.0 
     40.427         nan 
     15.740         nan 
      2.116         nan 
 
 BW-10.5.Lib_L_27F-045  80.0 
     41.407         nan 
     18.280         nan 
      2.206         nan 
 
 BW-10.5.Lib_L_27F-045  94.0 
     42.536         nan 
     20.290         nan 
      2.242         nan 
 
 BW-10.5.Lib_L_27F-045  108.0 
     44.664         nan 
     21.680         nan 
      2.259         nan 
 
 BW-10.5.Lib_L_27F-045  122.0 
     45.459         nan 
     23.670         nan 
      2.301         nan 
 
 BW-10.5.Lib_L_27F-045  136.0 
     44.560         nan 
     25.260         nan 
      2.312         nan 
 
 BW-10.5.Lib_L_27F-045  150.0 
     44.143         nan 
     27.000         nan 
      2.348         nan 
 
 BW-10.6.Lib_L_27F-046  10.0 
     16.005         nan 
      6.940         nan 
      2.563         nan 
 
 BW-10.6.Lib_L_27F-046  24.0 
     28.140         nan 
     11.930         nan 
      3.066         nan 
 
 BW-10.6.Lib_L_27F-046  38.0 
     46.149         nan 
     16.710         nan 
      3.401         nan 
 
 BW-10.6.Lib_L_27F-046  52.0 
     59.738         nan 
     20.790         nan 
      3.574         nan 
 
 BW-10.6.Lib_L_27F-046  66.0 
     63.117         nan 
     24.040         nan 
      3.666         nan 
 
 BW-10.6.Lib_L_27F-046  80.0 
     70.025         nan 
     27.450         nan 
      3.752         nan 
 
 BW-10.6.Lib_L_27F-046  94.0 
     72.530         nan 
     30.280         nan 
      3.786         nan 
 
 BW-10.6.Lib_L_27F-046  108.0 
     72.255         nan 
     33.280         nan 
      3.854         nan 
 
 BW-10.6.Lib_L_27F-046  122.0 
     74.439         nan 
     35.940         nan 
      3.905         nan 
 
 BW-10.6.Lib_L_27F-046  136.0 
     74.763         nan 
     38.500         nan 
      3.937         nan 
 
 BW-10.6.Lib_L_27F-046  150.0 
     74.333         nan 
     41.000         nan 
      3.973         nan 
 
 BW-10.8.Lib_L_27F-049  10.0 
     12.578         nan 
      5.570         nan 
      2.031         nan 
 
 BW-10.8.Lib_L_27F-049  24.0 
     27.152         nan 
     10.240         nan 
      2.507         nan 
 
 BW-10.8.Lib_L_27F-049  38.0 
     49.429         nan 
     15.390         nan 
      2.857         nan 
 
 BW-10.8.Lib_L_27F-049  52.0 
     53.899         nan 
     18.970         nan 
      2.993         nan 
 
 BW-10.8.Lib_L_27F-049  66.0 
     60.759         nan 
     22.600         nan 
      3.105         nan 
 
 BW-10.8.Lib_L_27F-049  80.0 
     62.174         nan 
     25.520         nan 
      3.125         nan 
 
 BW-10.8.Lib_L_27F-049  94.0 
     66.270         nan 
     28.700         nan 
      3.200         nan 
 
 BW-10.8.Lib_L_27F-049  108.0 
     63.573         nan 
     31.350         nan 
      3.273         nan 
 
 BW-10.8.Lib_L_27F-049  122.0 
     67.983         nan 
     34.030         nan 
      3.306         nan 
 
 BW-10.8.Lib_L_27F-049  136.0 
     68.543         nan 
     36.670         nan 
      3.361         nan 
 
 BW-10.8.Lib_L_27F-049  150.0 
     73.500         nan 
     39.000         nan 
      3.392         nan 
 
 BW-10.9.Lib_L_27F-050  10.0 
     11.172         nan 
      5.480         nan 
      2.040         nan 
 
 BW-10.9.Lib_L_27F-050  24.0 
     25.721         nan 
     10.160         nan 
      2.490         nan 
 
 BW-10.9.Lib_L_27F-050  38.0 
     32.253         nan 
     13.630         nan 
      2.670         nan 
 
 BW-10.9.Lib_L_27F-050  52.0 
     38.169         nan 
     16.700         nan 
      2.816         nan 
 
 BW-10.9.Lib_L_27F-050  66.0 
     43.764         nan 
     19.630         nan 
      2.941         nan 
 
 BW-10.9.Lib_L_27F-050  80.0 
     51.016         nan 
     22.550         nan 
      3.033         nan 
 
 BW-10.9.Lib_L_27F-050  94.0 
     56.273         nan 
     24.900         nan 
      3.038         nan 
 
 BW-10.9.Lib_L_27F-050  108.0 
     64.305         nan 
     27.170         nan 
      3.113         nan 
 
 BW-10.9.Lib_L_27F-050  122.0 
     75.305         nan 
     29.270         nan 
      3.140         nan 
 
 BW-10.9.Lib_L_27F-050  136.0 
     87.391         nan 
     30.960         nan 
      3.148         nan 
 
 BW-10.9.Lib_L_27F-050  150.0 
    103.000         nan 
     33.000         nan 
      3.178         nan 
 
  Subject  Seqs/Sample 
 chao1 Ave.  chao1 Err. 
 observed_species Ave.  observed_species Err. 
 shannon Ave.  shannon Err. 
 
 
 01  10.0 
     15.119       8.033 
      6.213       2.214 
      2.227       0.882 
 
 01  24.0 
     33.438      19.294 
     11.829       4.709 
      2.831       1.128 
 
 01  38.0 
     48.467      27.660 
     16.456       6.813 
      3.095       1.240 
 
 01  52.0 
     62.324      36.851 
     20.626       8.606 
      3.255       1.287 
 
 01  66.0 
     68.545      36.933 
     24.422      10.395 
      3.374       1.352 
 
 01  80.0 
     74.623      39.097 
     27.740      12.001 
      3.447       1.380 
 
 01  94.0 
     79.969      40.955 
     31.107      13.548 
      3.522       1.412 
 
 01  108.0 
     81.489      41.236 
     34.070      14.944 
      3.576       1.434 
 
 01  122.0 
     86.010      44.578 
     36.979      16.371 
      3.619       1.458 
 
 01  136.0 
     86.812      43.153 
     39.603      17.493 
      3.655       1.471 
 
 01  150.0 
     89.845      45.164 
     42.200      18.766 
      3.690       1.486 
 
 02  10.0 
      6.285       3.019 
      4.034       1.522 
      1.471       0.723 
 
 02  24.0 
     14.458       7.849 
      6.794       2.629 
      1.784       0.868 
 
 02  38.0 
     21.909      12.053 
      8.994       3.562 
      1.905       0.918 
 
 02  52.0 
     31.939      18.141 
     11.217       4.351 
      2.002       0.939 
 
 02  66.0 
     37.615      21.834 
     13.060       5.140 
      2.055       0.962 
 
 02  80.0 
     43.302      25.180 
     14.961       5.908 
      2.105       0.973 
 
 02  94.0 
     48.940      28.437 
     16.525       6.616 
      2.123       0.980 
 
 02  108.0 
     53.910      32.192 
     18.210       7.492 
      2.152       1.001 
 
 02  122.0 
     54.807      32.239 
     19.838       8.203 
      2.184       1.012 
 
 02  136.0 
     57.444      31.834 
     21.462       8.969 
      2.205       1.021 
 
 02  150.0 
     56.998      31.197 
     22.900       9.607 
      2.223       1.026 
 
 03  10.0 
      8.251       3.331 
      4.661       1.324 
      1.738       0.596 
 
 03  24.0 
     17.782       7.262 
      8.161       2.595 
      2.120       0.758 
 
 03  38.0 
     26.414      11.972 
     11.147       3.599 
      2.308       0.814 
 
 03  52.0 
     33.377      13.957 
     13.801       4.460 
      2.421       0.847 
 
 03  66.0 
     39.015      16.343 
     16.191       5.311 
      2.497       0.870 
 
 03  80.0 
     42.365      18.070 
     18.342       6.045 
      2.557       0.884 
 
 03  94.0 
     43.681      17.956 
     20.131       6.701 
      2.588       0.898 
 
 03  108.0 
     46.249      17.598 
     21.902       7.249 
      2.620       0.906 
 
 03  122.0 
     48.101      18.277 
     23.648       8.028 
      2.643       0.923 
 
 03  136.0 
     47.998      18.266 
     25.191       8.513 
      2.670       0.926 
 
 03  150.0 
     49.531      19.271 
     26.778       9.114 
      2.694       0.936 
 
 04  10.0 
     11.638       6.780 
      5.505       1.752 
      2.042       0.605 
 
 04  24.0 
     26.481      15.849 
     10.159       4.137 
      2.553       0.848 
 
 04  38.0 
     40.518      20.482 
     14.028       5.843 
      2.772       0.929 
 
 04  52.0 
     48.993      23.155 
     17.286       7.393 
      2.892       0.998 
 
 04  66.0 
     58.773      24.311 
     20.435       8.830 
      2.981       1.050 
 
 04  80.0 
     67.924      28.284 
     23.392       9.915 
      3.056       1.067 
 
 04  94.0 
     76.006      32.562 
     26.185      11.090 
      3.113       1.093 
 
 04  108.0 
     79.854      33.093 
     28.756      12.161 
      3.160       1.116 
 
 04  122.0 
     85.576      36.137 
     31.222      12.972 
      3.197       1.127 
 
 04  136.0 
     92.568      42.149 
     33.680      13.801 
      3.234       1.142 
 
 04  150.0 
     97.295      49.567 
     35.900      14.577 
      3.262       1.152 
 
 05  10.0 
     12.085       5.429 
      5.686       1.534 
      2.094       0.574 
 
 05  24.0 
     24.336       9.472 
     10.250       3.031 
      2.573       0.725 
 
 05  38.0 
     32.809      11.689 
     13.955       4.301 
      2.789       0.807 
 
 05  52.0 
     41.281      15.850 
     17.292       5.353 
      2.937       0.847 
 
 05  66.0 
     45.810      16.779 
     20.057       6.356 
      3.015       0.878 
 
 05  80.0 
     51.489      18.957 
     22.648       7.067 
      3.090       0.893 
 
 05  94.0 
     57.285      20.848 
     25.147       7.903 
      3.143       0.906 
 
 05  108.0 
     64.067      26.176 
     27.356       8.697 
      3.184       0.927 
 
 05  122.0 
     68.840      32.376 
     29.584       9.453 
      3.223       0.941 
 
 05  136.0 
     73.457      37.137 
     31.626      10.015 
      3.256       0.946 
 
 05  150.0 
     79.376      47.355 
     33.500      10.698 
      3.279       0.957 
 
 06  10.0 
     14.501       2.320 
      6.479       0.553 
      2.409       0.209 
 
 06  24.0 
     29.462       3.707 
     11.912       1.020 
      2.979       0.273 
 
 06  38.0 
     41.221       5.434 
     16.449       1.320 
      3.251       0.264 
 
 06  52.0 
     53.908      11.467 
     20.274       1.759 
      3.410       0.282 
 
 06  66.0 
     66.541      15.769 
     23.687       2.121 
      3.505       0.294 
 
 06  80.0 
     77.607      20.612 
     26.819       2.472 
      3.584       0.282 
 
 06  94.0 
     85.152      25.840 
     29.728       2.625 
      3.644       0.280 
 
 06  108.0 
     95.937      30.848 
     32.567       3.016 
      3.693       0.292 
 
 06  122.0 
    105.350      41.316 
     35.389       3.337 
      3.744       0.288 
 
 06  136.0 
    106.856      39.310 
     37.938       3.703 
      3.779       0.292 
 
 06  150.0 
    109.750      40.436 
     40.556       4.140 
      3.814       0.294 
 
 07  10.0 
      5.821       1.168 
      4.113       0.538 
      1.645       0.214 
 
 07  24.0 
     12.430       3.390 
      6.741       1.040 
      1.973       0.275 
 
 07  38.0 
     18.856       5.535 
      8.902       1.422 
      2.118       0.291 
 
 07  52.0 
     23.281       7.519 
     10.727       1.838 
      2.189       0.315 
 
 07  66.0 
     29.164      11.137 
     12.525       2.153 
      2.252       0.319 
 
 07  80.0 
     33.954      14.424 
     14.147       2.564 
      2.299       0.323 
 
 07  94.0 
     36.930      15.389 
     15.421       2.881 
      2.313       0.330 
 
 07  108.0 
     40.026      17.392 
     16.908       3.281 
      2.347       0.336 
 
 07  122.0 
     43.182      17.574 
     18.228       3.593 
      2.368       0.334 
 
 07  136.0 
     44.702      19.393 
     19.489       4.013 
      2.388       0.338 
 
 07  150.0 
     44.767      20.058 
     20.700       4.383 
      2.404       0.340 
 
 08  10.0 
     10.868       5.240 
      5.367       1.759 
      1.947       0.715 
 
 08  24.0 
     20.786       9.748 
      9.773       3.482 
      2.449       0.908 
 
 08  38.0 
     29.157      12.984 
     13.160       4.560 
      2.649       0.958 
 
 08  52.0 
     35.739      17.615 
     15.962       5.642 
      2.761       1.008 
 
 08  66.0 
     41.968      20.128 
     18.519       6.695 
      2.850       1.052 
 
 08  80.0 
     46.282      23.318 
     20.650       7.366 
      2.904       1.067 
 
 08  94.0 
     50.087      24.460 
     22.666       8.080 
      2.949       1.074 
 
 08  108.0 
     54.065      25.390 
     24.685       8.893 
      2.992       1.096 
 
 08  122.0 
     57.041      25.952 
     26.510       9.580 
      3.021       1.107 
 
 08  136.0 
     59.725      25.592 
     28.289      10.225 
      3.049       1.120 
 
 08  150.0 
     62.431      25.255 
     29.900      10.812 
      3.070       1.127 
 
 09  10.0 
     10.558       3.618 
      5.507       1.105 
      2.081       0.421 
 
 09  24.0 
     24.097      10.618 
      9.898       2.261 
      2.576       0.523 
 
 09  38.0 
     36.230      15.339 
     13.370       3.358 
      2.770       0.575 
 
 09  52.0 
     46.883      21.522 
     16.522       4.430 
      2.893       0.616 
 
 09  66.0 
     53.093      22.251 
     19.467       5.295 
      2.992       0.640 
 
 09  80.0 
     62.572      25.348 
     22.135       6.321 
      3.042       0.662 
 
 09  94.0 
     69.326      28.386 
     24.602       7.075 
      3.092       0.676 
 
 09  108.0 
     72.604      28.947 
     27.091       7.916 
      3.142       0.687 
 
 09  122.0 
     78.399      34.565 
     29.479       8.737 
      3.184       0.697 
 
 09  136.0 
     80.196      35.218 
     31.686       9.574 
      3.213       0.710 
 
 09  150.0 
     79.814      34.583 
     33.800      10.313 
      3.239       0.717 
 
 10  10.0 
     10.437       4.951 
      5.144       1.608 
      1.873       0.638 
 
 10  24.0 
     23.181      11.663 
      9.444       3.355 
      2.349       0.806 
 
 10  38.0 
     33.962      17.187 
     13.030       4.732 
      2.551       0.887 
 
 10  52.0 
     40.963      19.202 
     16.099       5.983 
      2.675       0.932 
 
 10  66.0 
     47.541      20.387 
     18.925       7.233 
      2.764       0.972 
 
 10  80.0 
     50.417      20.323 
     21.479       8.170 
      2.822       0.993 
 
 10  94.0 
     53.527      19.649 
     23.780       9.027 
      2.863       1.005 
 
 10  108.0 
     56.694      19.155 
     26.078      10.001 
      2.916       1.032 
 
 10  122.0 
     59.990      20.245 
     28.145      10.721 
      2.950       1.039 
 
 10  136.0 
     61.984      21.882 
     30.093      11.544 
      2.977       1.059 
 
 10  150.0 
     65.278      24.709 
     32.000      12.155 
      3.004       1.064
