# Supplementary material for: Characterization of the nasopharyngeal microbiota in health and during rhinovirus challenge
Source: Microbiome. 2014 Jun 25;2:22. doi: 10.1186/2049-2618-2-22 (PMC4098959; doi:10.1186/2049-2618-2-22)
Supplement: Additional file 8 — Compressed file. Alpha diversity_1000. Alpha diversity analyses including rarefaction plots. Plots exclude samples with fewer than the mean sequences/sample (approximately 1000). [file 2049-2618-2-22-S8.zip › 8440692981118328_add8.tgz!/alpha_div_mean/alpha_rarefaction_plots/rarefaction_plots.html]

 
 
 
   
   Rarefaction Curves 
 
td.data{font-size:10px;border-spacing:0px 10px;text-align:center;}
td.headers{font-size:12px;font-weight:bold;text-align:center;}
table{border-spacing:0px;}
.removed{display:none;}
.expands{cursor:pointer; cursor:hand;}
.child1 td:first-child{padding-left: 3px;}
 
 

function show_hide_category(checkobject){
    var imagetype=document.getElementById('imagetype').value;
    img=document.getElementById(checkobject.name.replace('_raw'+imagetype,'_ave'+imagetype))
    if (checkobject.checked==false){
        img.style.display='none';
    }else{
        img.style.display='';
    }
}

function reset_tree(){
    var category=document.getElementById('category').value;
    var metric=document.getElementById('metric').value;
    var old_all_categories=document.getElementById('all_categories');
    var imagetype=document.getElementById('imagetype').value;
    cat_list=old_all_categories.value.split('$#!')
    if (metric!='' && category != ''){
    for (var i=1, il=cat_list.length; i 

 
 
 
 
 
 
 
 
  
  Select a Metric:  
 
 
   
 chao1 
 observed_species 
 shannon 
 
 
    Select a Category:  
 
 
   
 BarcodeSequence 
 Description 
 Innoculation 
 LibCodes 
 SampleID 
 Subject 
 TimePoint 
 
 
 
 
 
  

 
      Show Categories: 
     
           
         All 
         None 
         Invert 
     
      
 Legend   
  ▶     ■    ACATACGCGTAG   
  ∟    ◆   BW.03.5   
  ▶     ■    ACGACTACAGAG   
  ∟    ◆   BW.02.9   
  ▶     ■    ACGAGTGCGTAG   
  ∟    ◆   BW.01.1   
  ▶     ■    ACGCGAGTATAG   
  ∟    ◆   BW.03.6   
  ▶     ■    AGACGCACTCAG   
  ∟    ◆   BW.01.3   
  ∟    ◆   BW.06.4   
  ▶     ■    AGACTATACTAG   
  ∟    ◆   BW.03.9   
  ▶     ■    AGCACTGTAGAG   
  ∟    ◆   BW.01.4   
  ▶     ■    AGCGTCGTCTAG   
  ∟    ◆   BW.03.10   
  ▶     ■    AGCTCACGTAAG   
  ∟    ◆   BW.05.9   
  ∟    ◆   BW.10.10   
  ▶     ■    ATACGACGTAAG   
  ∟    ◆   BW.07.5   
  ▶     ■    ATAGAGTACTAG   
  ∟    ◆   BW.09.3   
  ▶     ■    ATATCGCGAGAG   
  ∟    ◆   BW.06.7   
  ▶     ■    ATCAGACACGAG   
  ∟    ◆   BW.06.6   
  ▶     ■    CACGCTACGTAG   
  ∟    ◆   BW.09.4   
  ▶     ■    CAGTAGACGTAG   
  ∟    ◆   BW.04.4   
  ▶     ■    CATAGTAGTGAG   
  ∟    ◆   BW.07.3   
  ▶     ■    CGACGTGACTAG   
  ∟    ◆   BW.04.5   
  ▶     ■    CGAGAGATACAG   
  ∟    ◆   BW.07.4   
  ▶     ■    CGTAGACTAGAG   
  ∟    ◆   BW.02.10   
  ▶     ■    CGTCTAGTACAG   
  ∟    ◆   BW.02.6   
  ▶     ■    CTCGCGTGTCAG   
  ∟    ◆   BW.06.9   
  ▶     ■    TACACACACTAG   
  ∟    ◆   BW.04.6   
  ▶     ■    TACACGTGATAG   
  ∟    ◆   BW.04.7   
  ▶     ■    TACAGATCGTAG   
  ∟    ◆   BW.09.9   
  ▶     ■    TACGCTGTCTAG   
  ∟    ◆   BW.04.9   
  ▶     ■    TAGAGACGAGAG   
  ∟    ◆   BW.03.3   
  ▶     ■    TAGTGTAGATAG   
  ∟    ◆   BW.04.10   
  ▶     ■    TCGATCACGTAG   
  ∟    ◆   BW.05.1   
  ▶     ■    TCTACGTAGCAG   
  ∟    ◆   BW.02.7   
  ∟    ◆   BW.07.8   
  ▶     ■    TGATACGTCTAG   
  ∟    ◆   BW.02.1   
  ▶     ■    TGTACTACTCAG   
  ∟    ◆   BW.02.8   
  ▶     ■    BW-01.1.Lib_L_27F-001   
  ∟    ◆   BW.01.1   
  ▶     ■    BW-01.3.Lib_L_27F-003   
  ∟    ◆   BW.01.3   
  ▶     ■    BW-01.4.Lib_L_27F-004   
  ∟    ◆   BW.01.4   
  ▶     ■    BW-02.1.Lib_L_27F-011   
  ∟    ◆   BW.02.1   
  ▶     ■    BW-02.10.Lib_L_27F-021   
  ∟    ◆   BW.02.10   
  ▶     ■    BW-02.6.Lib_L_27F-017   
  ∟    ◆   BW.02.6   
  ▶     ■    BW-02.7.Lib_L_27F-018   
  ∟    ◆   BW.02.7   
  ▶     ■    BW-02.8.Lib_L_27F-019   
  ∟    ◆   BW.02.8   
  ▶     ■    BW-02.9.Lib_L_27F-020   
  ∟    ◆   BW.02.9   
  ▶     ■    BW-03.10.Lib_L_27F-031   
  ∟    ◆   BW.03.10   
  ▶     ■    BW-03.3.Lib_L_27F-024   
  ∟    ◆   BW.03.3   
  ▶     ■    BW-03.5.Lib_L_27F-026   
  ∟    ◆   BW.03.5   
  ▶     ■    BW-03.6.Lib_L_27F-027   
  ∟    ◆   BW.03.6   
  ▶     ■    BW-03.9.Lib_L_27F-030   
  ∟    ◆   BW.03.9   
  ▶     ■    BW-04.10.Lib_L_27F-041   
  ∟    ◆   BW.04.10   
  ▶     ■    BW-04.4.Lib_L_27F-035   
  ∟    ◆   BW.04.4   
  ▶     ■    BW-04.5.Lib_L_27F-036   
  ∟    ◆   BW.04.5   
  ▶     ■    BW-04.6.Lib_L_27F-037   
  ∟    ◆   BW.04.6   
  ▶     ■    BW-04.7.Lib_L_27F-038   
  ∟    ◆   BW.04.7   
  ▶     ■    BW-04.9.Lib_L_27F-040   
  ∟    ◆   BW.04.9   
  ▶     ■    BW-05.1.Lib_L_27F-042   
  ∟    ◆   BW.05.1   
  ▶     ■    BW-05.9.Lib_L_27F-051   
  ∟    ◆   BW.05.9   
  ▶     ■    BW-06.4.Lib_L_27F-003   
  ∟    ◆   BW.06.4   
  ▶     ■    BW-06.6.Lib_L_27F-005   
  ∟    ◆   BW.06.6   
  ▶     ■    BW-06.7.Lib_L_27F-006   
  ∟    ◆   BW.06.7   
  ▶     ■    BW-06.9.Lib_L_27F-008   
  ∟    ◆   BW.06.9   
  ▶     ■    BW-07.3.Lib_L_27F-013   
  ∟    ◆   BW.07.3   
  ▶     ■    BW-07.4.Lib_L_27F-014   
  ∟    ◆   BW.07.4   
  ▶     ■    BW-07.5.Lib_L_27F-015   
  ∟    ◆   BW.07.5   
  ▶     ■    BW-07.8.Lib_L_27F-018   
  ∟    ◆   BW.07.8   
  ▶     ■    BW-09.3.Lib_L_27F-033   
  ∟    ◆   BW.09.3   
  ▶     ■    BW-09.4.Lib_L_27F-034   
  ∟    ◆   BW.09.4   
  ▶     ■    BW-09.9.Lib_L_27F-039   
  ∟    ◆   BW.09.9   
  ▶     ■    BW-10.10.Lib_L_27F-051   
  ∟    ◆   BW.10.10   
  ▶     ■    After   
  ∟    ◆   BW.02.10   
  ∟    ◆   BW.02.9   
  ∟    ◆   BW.03.10   
  ∟    ◆   BW.03.9   
  ∟    ◆   BW.04.10   
  ∟    ◆   BW.04.9   
  ∟    ◆   BW.05.9   
  ∟    ◆   BW.06.9   
  ∟    ◆   BW.09.9   
  ∟    ◆   BW.10.10   
  ▶     ■    Before   
  ∟    ◆   BW.01.1   
  ∟    ◆   BW.01.3   
  ∟    ◆   BW.02.1   
  ∟    ◆   BW.03.3   
  ∟    ◆   BW.05.1   
  ∟    ◆   BW.07.3   
  ∟    ◆   BW.09.3   
  ▶     ■    During   
  ∟    ◆   BW.01.4   
  ∟    ◆   BW.02.6   
  ∟    ◆   BW.02.7   
  ∟    ◆   BW.02.8   
  ∟    ◆   BW.03.5   
  ∟    ◆   BW.03.6   
  ∟    ◆   BW.04.4   
  ∟    ◆   BW.04.5   
  ∟    ◆   BW.04.6   
  ∟    ◆   BW.04.7   
  ∟    ◆   BW.06.4   
  ∟    ◆   BW.06.6   
  ∟    ◆   BW.06.7   
  ∟    ◆   BW.07.4   
  ∟    ◆   BW.07.5   
  ∟    ◆   BW.07.8   
  ∟    ◆   BW.09.4   
  ▶     ■    Lib_L_27F-001   
  ∟    ◆   BW.01.1   
  ▶     ■    Lib_L_27F-003   
  ∟    ◆   BW.01.3   
  ∟    ◆   BW.06.4   
  ▶     ■    Lib_L_27F-004   
  ∟    ◆   BW.01.4   
  ▶     ■    Lib_L_27F-005   
  ∟    ◆   BW.06.6   
  ▶     ■    Lib_L_27F-006   
  ∟    ◆   BW.06.7   
  ▶     ■    Lib_L_27F-008   
  ∟    ◆   BW.06.9   
  ▶     ■    Lib_L_27F-011   
  ∟    ◆   BW.02.1   
  ▶     ■    Lib_L_27F-013   
  ∟    ◆   BW.07.3   
  ▶     ■    Lib_L_27F-014   
  ∟    ◆   BW.07.4   
  ▶     ■    Lib_L_27F-015   
  ∟    ◆   BW.07.5   
  ▶     ■    Lib_L_27F-017   
  ∟    ◆   BW.02.6   
  ▶     ■    Lib_L_27F-018   
  ∟    ◆   BW.02.7   
  ∟    ◆   BW.07.8   
  ▶     ■    Lib_L_27F-019   
  ∟    ◆   BW.02.8   
  ▶     ■    Lib_L_27F-020   
  ∟    ◆   BW.02.9   
  ▶     ■    Lib_L_27F-021   
  ∟    ◆   BW.02.10   
  ▶     ■    Lib_L_27F-024   
  ∟    ◆   BW.03.3   
  ▶     ■    Lib_L_27F-026   
  ∟    ◆   BW.03.5   
  ▶     ■    Lib_L_27F-027   
  ∟    ◆   BW.03.6   
  ▶     ■    Lib_L_27F-030   
  ∟    ◆   BW.03.9   
  ▶     ■    Lib_L_27F-031   
  ∟    ◆   BW.03.10   
  ▶     ■    Lib_L_27F-033   
  ∟    ◆   BW.09.3   
  ▶     ■    Lib_L_27F-034   
  ∟    ◆   BW.09.4   
  ▶     ■    Lib_L_27F-035   
  ∟    ◆   BW.04.4   
  ▶     ■    Lib_L_27F-036   
  ∟    ◆   BW.04.5   
  ▶     ■    Lib_L_27F-037   
  ∟    ◆   BW.04.6   
  ▶     ■    Lib_L_27F-038   
  ∟    ◆   BW.04.7   
  ▶     ■    Lib_L_27F-039   
  ∟    ◆   BW.09.9   
  ▶     ■    Lib_L_27F-040   
  ∟    ◆   BW.04.9   
  ▶     ■    Lib_L_27F-041   
  ∟    ◆   BW.04.10   
  ▶     ■    Lib_L_27F-042   
  ∟    ◆   BW.05.1   
  ▶     ■    Lib_L_27F-051   
  ∟    ◆   BW.05.9   
  ∟    ◆   BW.10.10   
  ▶     ■    BW.01.1   
  ∟    ◆   BW.01.1   
  ▶     ■    BW.01.3   
  ∟    ◆   BW.01.3   
  ▶     ■    BW.01.4   
  ∟    ◆   BW.01.4   
  ▶     ■    BW.02.1   
  ∟    ◆   BW.02.1   
  ▶     ■    BW.02.10   
  ∟    ◆   BW.02.10   
  ▶     ■    BW.02.6   
  ∟    ◆   BW.02.6   
  ▶     ■    BW.02.7   
  ∟    ◆   BW.02.7   
  ▶     ■    BW.02.8   
  ∟    ◆   BW.02.8   
  ▶     ■    BW.02.9   
  ∟    ◆   BW.02.9   
  ▶     ■    BW.03.10   
  ∟    ◆   BW.03.10   
  ▶     ■    BW.03.3   
  ∟    ◆   BW.03.3   
  ▶     ■    BW.03.5   
  ∟    ◆   BW.03.5   
  ▶     ■    BW.03.6   
  ∟    ◆   BW.03.6   
  ▶     ■    BW.03.9   
  ∟    ◆   BW.03.9   
  ▶     ■    BW.04.10   
  ∟    ◆   BW.04.10   
  ▶     ■    BW.04.4   
  ∟    ◆   BW.04.4   
  ▶     ■    BW.04.5   
  ∟    ◆   BW.04.5   
  ▶     ■    BW.04.6   
  ∟    ◆   BW.04.6   
  ▶     ■    BW.04.7   
  ∟    ◆   BW.04.7   
  ▶     ■    BW.04.9   
  ∟    ◆   BW.04.9   
  ▶     ■    BW.05.1   
  ∟    ◆   BW.05.1   
  ▶     ■    BW.05.9   
  ∟    ◆   BW.05.9   
  ▶     ■    BW.06.4   
  ∟    ◆   BW.06.4   
  ▶     ■    BW.06.6   
  ∟    ◆   BW.06.6   
  ▶     ■    BW.06.7   
  ∟    ◆   BW.06.7   
  ▶     ■    BW.06.9   
  ∟    ◆   BW.06.9   
  ▶     ■    BW.07.3   
  ∟    ◆   BW.07.3   
  ▶     ■    BW.07.4   
  ∟    ◆   BW.07.4   
  ▶     ■    BW.07.5   
  ∟    ◆   BW.07.5   
  ▶     ■    BW.07.8   
  ∟    ◆   BW.07.8   
  ▶     ■    BW.09.3   
  ∟    ◆   BW.09.3   
  ▶     ■    BW.09.4   
  ∟    ◆   BW.09.4   
  ▶     ■    BW.09.9   
  ∟    ◆   BW.09.9   
  ▶     ■    BW.10.10   
  ∟    ◆   BW.10.10   
  ▶     ■    01   
  ∟    ◆   BW.01.1   
  ∟    ◆   BW.01.3   
  ∟    ◆   BW.01.4   
  ▶     ■    02   
  ∟    ◆   BW.02.1   
  ∟    ◆   BW.02.10   
  ∟    ◆   BW.02.6   
  ∟    ◆   BW.02.7   
  ∟    ◆   BW.02.8   
  ∟    ◆   BW.02.9   
  ▶     ■    03   
  ∟    ◆   BW.03.10   
  ∟    ◆   BW.03.3   
  ∟    ◆   BW.03.5   
  ∟    ◆   BW.03.6   
  ∟    ◆   BW.03.9   
  ▶     ■    04   
  ∟    ◆   BW.04.10   
  ∟    ◆   BW.04.4   
  ∟    ◆   BW.04.5   
  ∟    ◆   BW.04.6   
  ∟    ◆   BW.04.7   
  ∟    ◆   BW.04.9   
  ▶     ■    05   
  ∟    ◆   BW.05.1   
  ∟    ◆   BW.05.9   
  ▶     ■    06   
  ∟    ◆   BW.06.4   
  ∟    ◆   BW.06.6   
  ∟    ◆   BW.06.7   
  ∟    ◆   BW.06.9   
  ▶     ■    07   
  ∟    ◆   BW.07.3   
  ∟    ◆   BW.07.4   
  ∟    ◆   BW.07.5   
  ∟    ◆   BW.07.8   
  ▶     ■    09   
  ∟    ◆   BW.09.3   
  ∟    ◆   BW.09.4   
  ∟    ◆   BW.09.9   
  ▶     ■    10   
  ∟    ◆   BW.10.10   
  ▶     ■    1   
  ∟    ◆   BW.01.1   
  ∟    ◆   BW.02.1   
  ∟    ◆   BW.05.1   
  ▶     ■    3   
  ∟    ◆   BW.01.3   
  ∟    ◆   BW.03.3   
  ∟    ◆   BW.07.3   
  ∟    ◆   BW.09.3   
  ▶     ■    4   
  ∟    ◆   BW.01.4   
  ∟    ◆   BW.04.4   
  ∟    ◆   BW.06.4   
  ∟    ◆   BW.07.4   
  ∟    ◆   BW.09.4   
  ▶     ■    5   
  ∟    ◆   BW.03.5   
  ∟    ◆   BW.04.5   
  ∟    ◆   BW.07.5   
  ▶     ■    6   
  ∟    ◆   BW.02.6   
  ∟    ◆   BW.03.6   
  ∟    ◆   BW.04.6   
  ∟    ◆   BW.06.6   
  ▶     ■    7   
  ∟    ◆   BW.02.7   
  ∟    ◆   BW.04.7   
  ∟    ◆   BW.06.7   
  ▶     ■    8   
  ∟    ◆   BW.02.8   
  ∟    ◆   BW.07.8   
  ▶     ■    9   
  ∟    ◆   BW.02.9   
  ∟    ◆   BW.03.9   
  ∟    ◆   BW.04.9   
  ∟    ◆   BW.05.9   
  ∟    ◆   BW.06.9   
  ∟    ◆   BW.09.9   
  ▶     ■    10   
  ∟    ◆   BW.02.10   
  ∟    ◆   BW.03.10   
  ∟    ◆   BW.04.10   
  ∟    ◆   BW.10.10   
  ▶     ■    ACATACGCGTAG   
  ∟    ◆   BW.03.5   
  ▶     ■    ACGACTACAGAG   
  ∟    ◆   BW.02.9   
  ▶     ■    ACGAGTGCGTAG   
  ∟    ◆   BW.01.1   
  ▶     ■    ACGCGAGTATAG   
  ∟    ◆   BW.03.6   
  ▶     ■    AGACGCACTCAG   
  ∟    ◆   BW.01.3   
  ∟    ◆   BW.06.4   
  ▶     ■    AGACTATACTAG   
  ∟    ◆   BW.03.9   
  ▶     ■    AGCACTGTAGAG   
  ∟    ◆   BW.01.4   
  ▶     ■    AGCGTCGTCTAG   
  ∟    ◆   BW.03.10   
  ▶     ■    AGCTCACGTAAG   
  ∟    ◆   BW.05.9   
  ∟    ◆   BW.10.10   
  ▶     ■    ATACGACGTAAG   
  ∟    ◆   BW.07.5   
  ▶     ■    ATAGAGTACTAG   
  ∟    ◆   BW.09.3   
  ▶     ■    ATATCGCGAGAG   
  ∟    ◆   BW.06.7   
  ▶     ■    ATCAGACACGAG   
  ∟    ◆   BW.06.6   
  ▶     ■    CACGCTACGTAG   
  ∟    ◆   BW.09.4   
  ▶     ■    CAGTAGACGTAG   
  ∟    ◆   BW.04.4   
  ▶     ■    CATAGTAGTGAG   
  ∟    ◆   BW.07.3   
  ▶     ■    CGACGTGACTAG   
  ∟    ◆   BW.04.5   
  ▶     ■    CGAGAGATACAG   
  ∟    ◆   BW.07.4   
  ▶     ■    CGTAGACTAGAG   
  ∟    ◆   BW.02.10   
  ▶     ■    CGTCTAGTACAG   
  ∟    ◆   BW.02.6   
  ▶     ■    CTCGCGTGTCAG   
  ∟    ◆   BW.06.9   
  ▶     ■    TACACACACTAG   
  ∟    ◆   BW.04.6   
  ▶     ■    TACACGTGATAG   
  ∟    ◆   BW.04.7   
  ▶     ■    TACAGATCGTAG   
  ∟    ◆   BW.09.9   
  ▶     ■    TACGCTGTCTAG   
  ∟    ◆   BW.04.9   
  ▶     ■    TAGAGACGAGAG   
  ∟    ◆   BW.03.3   
  ▶     ■    TAGTGTAGATAG   
  ∟    ◆   BW.04.10   
  ▶     ■    TCGATCACGTAG   
  ∟    ◆   BW.05.1   
  ▶     ■    TCTACGTAGCAG   
  ∟    ◆   BW.02.7   
  ∟    ◆   BW.07.8   
  ▶     ■    TGATACGTCTAG   
  ∟    ◆   BW.02.1   
  ▶     ■    TGTACTACTCAG   
  ∟    ◆   BW.02.8   
  ▶     ■    BW-01.1.Lib_L_27F-001   
  ∟    ◆   BW.01.1   
  ▶     ■    BW-01.3.Lib_L_27F-003   
  ∟    ◆   BW.01.3   
  ▶     ■    BW-01.4.Lib_L_27F-004   
  ∟    ◆   BW.01.4   
  ▶     ■    BW-02.1.Lib_L_27F-011   
  ∟    ◆   BW.02.1   
  ▶     ■    BW-02.10.Lib_L_27F-021   
  ∟    ◆   BW.02.10   
  ▶     ■    BW-02.6.Lib_L_27F-017   
  ∟    ◆   BW.02.6   
  ▶     ■    BW-02.7.Lib_L_27F-018   
  ∟    ◆   BW.02.7   
  ▶     ■    BW-02.8.Lib_L_27F-019   
  ∟    ◆   BW.02.8   
  ▶     ■    BW-02.9.Lib_L_27F-020   
  ∟    ◆   BW.02.9   
  ▶     ■    BW-03.10.Lib_L_27F-031   
  ∟    ◆   BW.03.10   
  ▶     ■    BW-03.3.Lib_L_27F-024   
  ∟    ◆   BW.03.3   
  ▶     ■    BW-03.5.Lib_L_27F-026   
  ∟    ◆   BW.03.5   
  ▶     ■    BW-03.6.Lib_L_27F-027   
  ∟    ◆   BW.03.6   
  ▶     ■    BW-03.9.Lib_L_27F-030   
  ∟    ◆   BW.03.9   
  ▶     ■    BW-04.10.Lib_L_27F-041   
  ∟    ◆   BW.04.10   
  ▶     ■    BW-04.4.Lib_L_27F-035   
  ∟    ◆   BW.04.4   
  ▶     ■    BW-04.5.Lib_L_27F-036   
  ∟    ◆   BW.04.5   
  ▶     ■    BW-04.6.Lib_L_27F-037   
  ∟    ◆   BW.04.6   
  ▶     ■    BW-04.7.Lib_L_27F-038   
  ∟    ◆   BW.04.7   
  ▶     ■    BW-04.9.Lib_L_27F-040   
  ∟    ◆   BW.04.9   
  ▶     ■    BW-05.1.Lib_L_27F-042   
  ∟    ◆   BW.05.1   
  ▶     ■    BW-05.9.Lib_L_27F-051   
  ∟    ◆   BW.05.9   
  ▶     ■    BW-06.4.Lib_L_27F-003   
  ∟    ◆   BW.06.4   
  ▶     ■    BW-06.6.Lib_L_27F-005   
  ∟    ◆   BW.06.6   
  ▶     ■    BW-06.7.Lib_L_27F-006   
  ∟    ◆   BW.06.7   
  ▶     ■    BW-06.9.Lib_L_27F-008   
  ∟    ◆   BW.06.9   
  ▶     ■    BW-07.3.Lib_L_27F-013   
  ∟    ◆   BW.07.3   
  ▶     ■    BW-07.4.Lib_L_27F-014   
  ∟    ◆   BW.07.4   
  ▶     ■    BW-07.5.Lib_L_27F-015   
  ∟    ◆   BW.07.5   
  ▶     ■    BW-07.8.Lib_L_27F-018   
  ∟    ◆   BW.07.8   
  ▶     ■    BW-09.3.Lib_L_27F-033   
  ∟    ◆   BW.09.3   
  ▶     ■    BW-09.4.Lib_L_27F-034   
  ∟    ◆   BW.09.4   
  ▶     ■    BW-09.9.Lib_L_27F-039   
  ∟    ◆   BW.09.9   
  ▶     ■    BW-10.10.Lib_L_27F-051   
  ∟    ◆   BW.10.10   
  ▶     ■    After   
  ∟    ◆   BW.02.10   
  ∟    ◆   BW.02.9   
  ∟    ◆   BW.03.10   
  ∟    ◆   BW.03.9   
  ∟    ◆   BW.04.10   
  ∟    ◆   BW.04.9   
  ∟    ◆   BW.05.9   
  ∟    ◆   BW.06.9   
  ∟    ◆   BW.09.9   
  ∟    ◆   BW.10.10   
  ▶     ■    Before   
  ∟    ◆   BW.01.1   
  ∟    ◆   BW.01.3   
  ∟    ◆   BW.02.1   
  ∟    ◆   BW.03.3   
  ∟    ◆   BW.05.1   
  ∟    ◆   BW.07.3   
  ∟    ◆   BW.09.3   
  ▶     ■    During   
  ∟    ◆   BW.01.4   
  ∟    ◆   BW.02.6   
  ∟    ◆   BW.02.7   
  ∟    ◆   BW.02.8   
  ∟    ◆   BW.03.5   
  ∟    ◆   BW.03.6   
  ∟    ◆   BW.04.4   
  ∟    ◆   BW.04.5   
  ∟    ◆   BW.04.6   
  ∟    ◆   BW.04.7   
  ∟    ◆   BW.06.4   
  ∟    ◆   BW.06.6   
  ∟    ◆   BW.06.7   
  ∟    ◆   BW.07.4   
  ∟    ◆   BW.07.5   
  ∟    ◆   BW.07.8   
  ∟    ◆   BW.09.4   
  ▶     ■    Lib_L_27F-001   
  ∟    ◆   BW.01.1   
  ▶     ■    Lib_L_27F-003   
  ∟    ◆   BW.01.3   
  ∟    ◆   BW.06.4   
  ▶     ■    Lib_L_27F-004   
  ∟    ◆   BW.01.4   
  ▶     ■    Lib_L_27F-005   
  ∟    ◆   BW.06.6   
  ▶     ■    Lib_L_27F-006   
  ∟    ◆   BW.06.7   
  ▶     ■    Lib_L_27F-008   
  ∟    ◆   BW.06.9   
  ▶     ■    Lib_L_27F-011   
  ∟    ◆   BW.02.1   
  ▶     ■    Lib_L_27F-013   
  ∟    ◆   BW.07.3   
  ▶     ■    Lib_L_27F-014   
  ∟    ◆   BW.07.4   
  ▶     ■    Lib_L_27F-015   
  ∟    ◆   BW.07.5   
  ▶     ■    Lib_L_27F-017   
  ∟    ◆   BW.02.6   
  ▶     ■    Lib_L_27F-018   
  ∟    ◆   BW.02.7   
  ∟    ◆   BW.07.8   
  ▶     ■    Lib_L_27F-019   
  ∟    ◆   BW.02.8   
  ▶     ■    Lib_L_27F-020   
  ∟    ◆   BW.02.9   
  ▶     ■    Lib_L_27F-021   
  ∟    ◆   BW.02.10   
  ▶     ■    Lib_L_27F-024   
  ∟    ◆   BW.03.3   
  ▶     ■    Lib_L_27F-026   
  ∟    ◆   BW.03.5   
  ▶     ■    Lib_L_27F-027   
  ∟    ◆   BW.03.6   
  ▶     ■    Lib_L_27F-030   
  ∟    ◆   BW.03.9   
  ▶     ■    Lib_L_27F-031   
  ∟    ◆   BW.03.10   
  ▶     ■    Lib_L_27F-033   
  ∟    ◆   BW.09.3   
  ▶     ■    Lib_L_27F-034   
  ∟    ◆   BW.09.4   
  ▶     ■    Lib_L_27F-035   
  ∟    ◆   BW.04.4   
  ▶     ■    Lib_L_27F-036   
  ∟    ◆   BW.04.5   
  ▶     ■    Lib_L_27F-037   
  ∟    ◆   BW.04.6   
  ▶     ■    Lib_L_27F-038   
  ∟    ◆   BW.04.7   
  ▶     ■    Lib_L_27F-039   
  ∟    ◆   BW.09.9   
  ▶     ■    Lib_L_27F-040   
  ∟    ◆   BW.04.9   
  ▶     ■    Lib_L_27F-041   
  ∟    ◆   BW.04.10   
  ▶     ■    Lib_L_27F-042   
  ∟    ◆   BW.05.1   
  ▶     ■    Lib_L_27F-051   
  ∟    ◆   BW.05.9   
  ∟    ◆   BW.10.10   
  ▶     ■    BW.01.1   
  ∟    ◆   BW.01.1   
  ▶     ■    BW.01.3   
  ∟    ◆   BW.01.3   
  ▶     ■    BW.01.4   
  ∟    ◆   BW.01.4   
  ▶     ■    BW.02.1   
  ∟    ◆   BW.02.1   
  ▶     ■    BW.02.10   
  ∟    ◆   BW.02.10   
  ▶     ■    BW.02.6   
  ∟    ◆   BW.02.6   
  ▶     ■    BW.02.7   
  ∟    ◆   BW.02.7   
  ▶     ■    BW.02.8   
  ∟    ◆   BW.02.8   
  ▶     ■    BW.02.9   
  ∟    ◆   BW.02.9   
  ▶     ■    BW.03.10   
  ∟    ◆   BW.03.10   
  ▶     ■    BW.03.3   
  ∟    ◆   BW.03.3   
  ▶     ■    BW.03.5   
  ∟    ◆   BW.03.5   
  ▶     ■    BW.03.6   
  ∟    ◆   BW.03.6   
  ▶     ■    BW.03.9   
  ∟    ◆   BW.03.9   
  ▶     ■    BW.04.10   
  ∟    ◆   BW.04.10   
  ▶     ■    BW.04.4   
  ∟    ◆   BW.04.4   
  ▶     ■    BW.04.5   
  ∟    ◆   BW.04.5   
  ▶     ■    BW.04.6   
  ∟    ◆   BW.04.6   
  ▶     ■    BW.04.7   
  ∟    ◆   BW.04.7   
  ▶     ■    BW.04.9   
  ∟    ◆   BW.04.9   
  ▶     ■    BW.05.1   
  ∟    ◆   BW.05.1   
  ▶     ■    BW.05.9   
  ∟    ◆   BW.05.9   
  ▶     ■    BW.06.4   
  ∟    ◆   BW.06.4   
  ▶     ■    BW.06.6   
  ∟    ◆   BW.06.6   
  ▶     ■    BW.06.7   
  ∟    ◆   BW.06.7   
  ▶     ■    BW.06.9   
  ∟    ◆   BW.06.9   
  ▶     ■    BW.07.3   
  ∟    ◆   BW.07.3   
  ▶     ■    BW.07.4   
  ∟    ◆   BW.07.4   
  ▶     ■    BW.07.5   
  ∟    ◆   BW.07.5   
  ▶     ■    BW.07.8   
  ∟    ◆   BW.07.8   
  ▶     ■    BW.09.3   
  ∟    ◆   BW.09.3   
  ▶     ■    BW.09.4   
  ∟    ◆   BW.09.4   
  ▶     ■    BW.09.9   
  ∟    ◆   BW.09.9   
  ▶     ■    BW.10.10   
  ∟    ◆   BW.10.10   
  ▶     ■    01   
  ∟    ◆   BW.01.1   
  ∟    ◆   BW.01.3   
  ∟    ◆   BW.01.4   
  ▶     ■    02   
  ∟    ◆   BW.02.1   
  ∟    ◆   BW.02.10   
  ∟    ◆   BW.02.6   
  ∟    ◆   BW.02.7   
  ∟    ◆   BW.02.8   
  ∟    ◆   BW.02.9   
  ▶     ■    03   
  ∟    ◆   BW.03.10   
  ∟    ◆   BW.03.3   
  ∟    ◆   BW.03.5   
  ∟    ◆   BW.03.6   
  ∟    ◆   BW.03.9   
  ▶     ■    04   
  ∟    ◆   BW.04.10   
  ∟    ◆   BW.04.4   
  ∟    ◆   BW.04.5   
  ∟    ◆   BW.04.6   
  ∟    ◆   BW.04.7   
  ∟    ◆   BW.04.9   
  ▶     ■    05   
  ∟    ◆   BW.05.1   
  ∟    ◆   BW.05.9   
  ▶     ■    06   
  ∟    ◆   BW.06.4   
  ∟    ◆   BW.06.6   
  ∟    ◆   BW.06.7   
  ∟    ◆   BW.06.9   
  ▶     ■    07   
  ∟    ◆   BW.07.3   
  ∟    ◆   BW.07.4   
  ∟    ◆   BW.07.5   
  ∟    ◆   BW.07.8   
  ▶     ■    09   
  ∟    ◆   BW.09.3   
  ∟    ◆   BW.09.4   
  ∟    ◆   BW.09.9   
  ▶     ■    10   
  ∟    ◆   BW.10.10   
  ▶     ■    1   
  ∟    ◆   BW.01.1   
  ∟    ◆   BW.02.1   
  ∟    ◆   BW.05.1   
  ▶     ■    3   
  ∟    ◆   BW.01.3   
  ∟    ◆   BW.03.3   
  ∟    ◆   BW.07.3   
  ∟    ◆   BW.09.3   
  ▶     ■    4   
  ∟    ◆   BW.01.4   
  ∟    ◆   BW.04.4   
  ∟    ◆   BW.06.4   
  ∟    ◆   BW.07.4   
  ∟    ◆   BW.09.4   
  ▶     ■    5   
  ∟    ◆   BW.03.5   
  ∟    ◆   BW.04.5   
  ∟    ◆   BW.07.5   
  ▶     ■    6   
  ∟    ◆   BW.02.6   
  ∟    ◆   BW.03.6   
  ∟    ◆   BW.04.6   
  ∟    ◆   BW.06.6   
  ▶     ■    7   
  ∟    ◆   BW.02.7   
  ∟    ◆   BW.04.7   
  ∟    ◆   BW.06.7   
  ▶     ■    8   
  ∟    ◆   BW.02.8   
  ∟    ◆   BW.07.8   
  ▶     ■    9   
  ∟    ◆   BW.02.9   
  ∟    ◆   BW.03.9   
  ∟    ◆   BW.04.9   
  ∟    ◆   BW.05.9   
  ∟    ◆   BW.06.9   
  ∟    ◆   BW.09.9   
  ▶     ■    10   
  ∟    ◆   BW.02.10   
  ∟    ◆   BW.03.10   
  ∟    ◆   BW.04.10   
  ∟    ◆   BW.10.10   
  ▶     ■    ACATACGCGTAG   
  ∟    ◆   BW.03.5   
  ▶     ■    ACGACTACAGAG   
  ∟    ◆   BW.02.9   
  ▶     ■    ACGAGTGCGTAG   
  ∟    ◆   BW.01.1   
  ▶     ■    ACGCGAGTATAG   
  ∟    ◆   BW.03.6   
  ▶     ■    AGACGCACTCAG   
  ∟    ◆   BW.01.3   
  ∟    ◆   BW.06.4   
  ▶     ■    AGACTATACTAG   
  ∟    ◆   BW.03.9   
  ▶     ■    AGCACTGTAGAG   
  ∟    ◆   BW.01.4   
  ▶     ■    AGCGTCGTCTAG   
  ∟    ◆   BW.03.10   
  ▶     ■    AGCTCACGTAAG   
  ∟    ◆   BW.05.9   
  ∟    ◆   BW.10.10   
  ▶     ■    ATACGACGTAAG   
  ∟    ◆   BW.07.5   
  ▶     ■    ATAGAGTACTAG   
  ∟    ◆   BW.09.3   
  ▶     ■    ATATCGCGAGAG   
  ∟    ◆   BW.06.7   
  ▶     ■    ATCAGACACGAG   
  ∟    ◆   BW.06.6   
  ▶     ■    CACGCTACGTAG   
  ∟    ◆   BW.09.4   
  ▶     ■    CAGTAGACGTAG   
  ∟    ◆   BW.04.4   
  ▶     ■    CATAGTAGTGAG   
  ∟    ◆   BW.07.3   
  ▶     ■    CGACGTGACTAG   
  ∟    ◆   BW.04.5   
  ▶     ■    CGAGAGATACAG   
  ∟    ◆   BW.07.4   
  ▶     ■    CGTAGACTAGAG   
  ∟    ◆   BW.02.10   
  ▶     ■    CGTCTAGTACAG   
  ∟    ◆   BW.02.6   
  ▶     ■    CTCGCGTGTCAG   
  ∟    ◆   BW.06.9   
  ▶     ■    TACACACACTAG   
  ∟    ◆   BW.04.6   
  ▶     ■    TACACGTGATAG   
  ∟    ◆   BW.04.7   
  ▶     ■    TACAGATCGTAG   
  ∟    ◆   BW.09.9   
  ▶     ■    TACGCTGTCTAG   
  ∟    ◆   BW.04.9   
  ▶     ■    TAGAGACGAGAG   
  ∟    ◆   BW.03.3   
  ▶     ■    TAGTGTAGATAG   
  ∟    ◆   BW.04.10   
  ▶     ■    TCGATCACGTAG   
  ∟    ◆   BW.05.1   
  ▶     ■    TCTACGTAGCAG   
  ∟    ◆   BW.02.7   
  ∟    ◆   BW.07.8   
  ▶     ■    TGATACGTCTAG   
  ∟    ◆   BW.02.1   
  ▶     ■    TGTACTACTCAG   
  ∟    ◆   BW.02.8   
  ▶     ■    BW-01.1.Lib_L_27F-001   
  ∟    ◆   BW.01.1   
  ▶     ■    BW-01.3.Lib_L_27F-003   
  ∟    ◆   BW.01.3   
  ▶     ■    BW-01.4.Lib_L_27F-004   
  ∟    ◆   BW.01.4   
  ▶     ■    BW-02.1.Lib_L_27F-011   
  ∟    ◆   BW.02.1   
  ▶     ■    BW-02.10.Lib_L_27F-021   
  ∟    ◆   BW.02.10   
  ▶     ■    BW-02.6.Lib_L_27F-017   
  ∟    ◆   BW.02.6   
  ▶     ■    BW-02.7.Lib_L_27F-018   
  ∟    ◆   BW.02.7   
  ▶     ■    BW-02.8.Lib_L_27F-019   
  ∟    ◆   BW.02.8   
  ▶     ■    BW-02.9.Lib_L_27F-020   
  ∟    ◆   BW.02.9   
  ▶     ■    BW-03.10.Lib_L_27F-031   
  ∟    ◆   BW.03.10   
  ▶     ■    BW-03.3.Lib_L_27F-024   
  ∟    ◆   BW.03.3   
  ▶     ■    BW-03.5.Lib_L_27F-026   
  ∟    ◆   BW.03.5   
  ▶     ■    BW-03.6.Lib_L_27F-027   
  ∟    ◆   BW.03.6   
  ▶     ■    BW-03.9.Lib_L_27F-030   
  ∟    ◆   BW.03.9   
  ▶     ■    BW-04.10.Lib_L_27F-041   
  ∟    ◆   BW.04.10   
  ▶     ■    BW-04.4.Lib_L_27F-035   
  ∟    ◆   BW.04.4   
  ▶     ■    BW-04.5.Lib_L_27F-036   
  ∟    ◆   BW.04.5   
  ▶     ■    BW-04.6.Lib_L_27F-037   
  ∟    ◆   BW.04.6   
  ▶     ■    BW-04.7.Lib_L_27F-038   
  ∟    ◆   BW.04.7   
  ▶     ■    BW-04.9.Lib_L_27F-040   
  ∟    ◆   BW.04.9   
  ▶     ■    BW-05.1.Lib_L_27F-042   
  ∟    ◆   BW.05.1   
  ▶     ■    BW-05.9.Lib_L_27F-051   
  ∟    ◆   BW.05.9   
  ▶     ■    BW-06.4.Lib_L_27F-003   
  ∟    ◆   BW.06.4   
  ▶     ■    BW-06.6.Lib_L_27F-005   
  ∟    ◆   BW.06.6   
  ▶     ■    BW-06.7.Lib_L_27F-006   
  ∟    ◆   BW.06.7   
  ▶     ■    BW-06.9.Lib_L_27F-008   
  ∟    ◆   BW.06.9   
  ▶     ■    BW-07.3.Lib_L_27F-013   
  ∟    ◆   BW.07.3   
  ▶     ■    BW-07.4.Lib_L_27F-014   
  ∟    ◆   BW.07.4   
  ▶     ■    BW-07.5.Lib_L_27F-015   
  ∟    ◆   BW.07.5   
  ▶     ■    BW-07.8.Lib_L_27F-018   
  ∟    ◆   BW.07.8   
  ▶     ■    BW-09.3.Lib_L_27F-033   
  ∟    ◆   BW.09.3   
  ▶     ■    BW-09.4.Lib_L_27F-034   
  ∟    ◆   BW.09.4   
  ▶     ■    BW-09.9.Lib_L_27F-039   
  ∟    ◆   BW.09.9   
  ▶     ■    BW-10.10.Lib_L_27F-051   
  ∟    ◆   BW.10.10   
  ▶     ■    After   
  ∟    ◆   BW.02.10   
  ∟    ◆   BW.02.9   
  ∟    ◆   BW.03.10   
  ∟    ◆   BW.03.9   
  ∟    ◆   BW.04.10   
  ∟    ◆   BW.04.9   
  ∟    ◆   BW.05.9   
  ∟    ◆   BW.06.9   
  ∟    ◆   BW.09.9   
  ∟    ◆   BW.10.10   
  ▶     ■    Before   
  ∟    ◆   BW.01.1   
  ∟    ◆   BW.01.3   
  ∟    ◆   BW.02.1   
  ∟    ◆   BW.03.3   
  ∟    ◆   BW.05.1   
  ∟    ◆   BW.07.3   
  ∟    ◆   BW.09.3   
  ▶     ■    During   
  ∟    ◆   BW.01.4   
  ∟    ◆   BW.02.6   
  ∟    ◆   BW.02.7   
  ∟    ◆   BW.02.8   
  ∟    ◆   BW.03.5   
  ∟    ◆   BW.03.6   
  ∟    ◆   BW.04.4   
  ∟    ◆   BW.04.5   
  ∟    ◆   BW.04.6   
  ∟    ◆   BW.04.7   
  ∟    ◆   BW.06.4   
  ∟    ◆   BW.06.6   
  ∟    ◆   BW.06.7   
  ∟    ◆   BW.07.4   
  ∟    ◆   BW.07.5   
  ∟    ◆   BW.07.8   
  ∟    ◆   BW.09.4   
  ▶     ■    Lib_L_27F-001   
  ∟    ◆   BW.01.1   
  ▶     ■    Lib_L_27F-003   
  ∟    ◆   BW.01.3   
  ∟    ◆   BW.06.4   
  ▶     ■    Lib_L_27F-004   
  ∟    ◆   BW.01.4   
  ▶     ■    Lib_L_27F-005   
  ∟    ◆   BW.06.6   
  ▶     ■    Lib_L_27F-006   
  ∟    ◆   BW.06.7   
  ▶     ■    Lib_L_27F-008   
  ∟    ◆   BW.06.9   
  ▶     ■    Lib_L_27F-011   
  ∟    ◆   BW.02.1   
  ▶     ■    Lib_L_27F-013   
  ∟    ◆   BW.07.3   
  ▶     ■    Lib_L_27F-014   
  ∟    ◆   BW.07.4   
  ▶     ■    Lib_L_27F-015   
  ∟    ◆   BW.07.5   
  ▶     ■    Lib_L_27F-017   
  ∟    ◆   BW.02.6   
  ▶     ■    Lib_L_27F-018   
  ∟    ◆   BW.02.7   
  ∟    ◆   BW.07.8   
  ▶     ■    Lib_L_27F-019   
  ∟    ◆   BW.02.8   
  ▶     ■    Lib_L_27F-020   
  ∟    ◆   BW.02.9   
  ▶     ■    Lib_L_27F-021   
  ∟    ◆   BW.02.10   
  ▶     ■    Lib_L_27F-024   
  ∟    ◆   BW.03.3   
  ▶     ■    Lib_L_27F-026   
  ∟    ◆   BW.03.5   
  ▶     ■    Lib_L_27F-027   
  ∟    ◆   BW.03.6   
  ▶     ■    Lib_L_27F-030   
  ∟    ◆   BW.03.9   
  ▶     ■    Lib_L_27F-031   
  ∟    ◆   BW.03.10   
  ▶     ■    Lib_L_27F-033   
  ∟    ◆   BW.09.3   
  ▶     ■    Lib_L_27F-034   
  ∟    ◆   BW.09.4   
  ▶     ■    Lib_L_27F-035   
  ∟    ◆   BW.04.4   
  ▶     ■    Lib_L_27F-036   
  ∟    ◆   BW.04.5   
  ▶     ■    Lib_L_27F-037   
  ∟    ◆   BW.04.6   
  ▶     ■    Lib_L_27F-038   
  ∟    ◆   BW.04.7   
  ▶     ■    Lib_L_27F-039   
  ∟    ◆   BW.09.9   
  ▶     ■    Lib_L_27F-040   
  ∟    ◆   BW.04.9   
  ▶     ■    Lib_L_27F-041   
  ∟    ◆   BW.04.10   
  ▶     ■    Lib_L_27F-042   
  ∟    ◆   BW.05.1   
  ▶     ■    Lib_L_27F-051   
  ∟    ◆   BW.05.9   
  ∟    ◆   BW.10.10   
  ▶     ■    BW.01.1   
  ∟    ◆   BW.01.1   
  ▶     ■    BW.01.3   
  ∟    ◆   BW.01.3   
  ▶     ■    BW.01.4   
  ∟    ◆   BW.01.4   
  ▶     ■    BW.02.1   
  ∟    ◆   BW.02.1   
  ▶     ■    BW.02.10   
  ∟    ◆   BW.02.10   
  ▶     ■    BW.02.6   
  ∟    ◆   BW.02.6   
  ▶     ■    BW.02.7   
  ∟    ◆   BW.02.7   
  ▶     ■    BW.02.8   
  ∟    ◆   BW.02.8   
  ▶     ■    BW.02.9   
  ∟    ◆   BW.02.9   
  ▶     ■    BW.03.10   
  ∟    ◆   BW.03.10   
  ▶     ■    BW.03.3   
  ∟    ◆   BW.03.3   
  ▶     ■    BW.03.5   
  ∟    ◆   BW.03.5   
  ▶     ■    BW.03.6   
  ∟    ◆   BW.03.6   
  ▶     ■    BW.03.9   
  ∟    ◆   BW.03.9   
  ▶     ■    BW.04.10   
  ∟    ◆   BW.04.10   
  ▶     ■    BW.04.4   
  ∟    ◆   BW.04.4   
  ▶     ■    BW.04.5   
  ∟    ◆   BW.04.5   
  ▶     ■    BW.04.6   
  ∟    ◆   BW.04.6   
  ▶     ■    BW.04.7   
  ∟    ◆   BW.04.7   
  ▶     ■    BW.04.9   
  ∟    ◆   BW.04.9   
  ▶     ■    BW.05.1   
  ∟    ◆   BW.05.1   
  ▶     ■    BW.05.9   
  ∟    ◆   BW.05.9   
  ▶     ■    BW.06.4   
  ∟    ◆   BW.06.4   
  ▶     ■    BW.06.6   
  ∟    ◆   BW.06.6   
  ▶     ■    BW.06.7   
  ∟    ◆   BW.06.7   
  ▶     ■    BW.06.9   
  ∟    ◆   BW.06.9   
  ▶     ■    BW.07.3   
  ∟    ◆   BW.07.3   
  ▶     ■    BW.07.4   
  ∟    ◆   BW.07.4   
  ▶     ■    BW.07.5   
  ∟    ◆   BW.07.5   
  ▶     ■    BW.07.8   
  ∟    ◆   BW.07.8   
  ▶     ■    BW.09.3   
  ∟    ◆   BW.09.3   
  ▶     ■    BW.09.4   
  ∟    ◆   BW.09.4   
  ▶     ■    BW.09.9   
  ∟    ◆   BW.09.9   
  ▶     ■    BW.10.10   
  ∟    ◆   BW.10.10   
  ▶     ■    01   
  ∟    ◆   BW.01.1   
  ∟    ◆   BW.01.3   
  ∟    ◆   BW.01.4   
  ▶     ■    02   
  ∟    ◆   BW.02.1   
  ∟    ◆   BW.02.10   
  ∟    ◆   BW.02.6   
  ∟    ◆   BW.02.7   
  ∟    ◆   BW.02.8   
  ∟    ◆   BW.02.9   
  ▶     ■    03   
  ∟    ◆   BW.03.10   
  ∟    ◆   BW.03.3   
  ∟    ◆   BW.03.5   
  ∟    ◆   BW.03.6   
  ∟    ◆   BW.03.9   
  ▶     ■    04   
  ∟    ◆   BW.04.10   
  ∟    ◆   BW.04.4   
  ∟    ◆   BW.04.5   
  ∟    ◆   BW.04.6   
  ∟    ◆   BW.04.7   
  ∟    ◆   BW.04.9   
  ▶     ■    05   
  ∟    ◆   BW.05.1   
  ∟    ◆   BW.05.9   
  ▶     ■    06   
  ∟    ◆   BW.06.4   
  ∟    ◆   BW.06.6   
  ∟    ◆   BW.06.7   
  ∟    ◆   BW.06.9   
  ▶     ■    07   
  ∟    ◆   BW.07.3   
  ∟    ◆   BW.07.4   
  ∟    ◆   BW.07.5   
  ∟    ◆   BW.07.8   
  ▶     ■    09   
  ∟    ◆   BW.09.3   
  ∟    ◆   BW.09.4   
  ∟    ◆   BW.09.9   
  ▶     ■    10   
  ∟    ◆   BW.10.10   
  ▶     ■    1   
  ∟    ◆   BW.01.1   
  ∟    ◆   BW.02.1   
  ∟    ◆   BW.05.1   
  ▶     ■    3   
  ∟    ◆   BW.01.3   
  ∟    ◆   BW.03.3   
  ∟    ◆   BW.07.3   
  ∟    ◆   BW.09.3   
  ▶     ■    4   
  ∟    ◆   BW.01.4   
  ∟    ◆   BW.04.4   
  ∟    ◆   BW.06.4   
  ∟    ◆   BW.07.4   
  ∟    ◆   BW.09.4   
  ▶     ■    5   
  ∟    ◆   BW.03.5   
  ∟    ◆   BW.04.5   
  ∟    ◆   BW.07.5   
  ▶     ■    6   
  ∟    ◆   BW.02.6   
  ∟    ◆   BW.03.6   
  ∟    ◆   BW.04.6   
  ∟    ◆   BW.06.6   
  ▶     ■    7   
  ∟    ◆   BW.02.7   
  ∟    ◆   BW.04.7   
  ∟    ◆   BW.06.7   
  ▶     ■    8   
  ∟    ◆   BW.02.8   
  ∟    ◆   BW.07.8   
  ▶     ■    9   
  ∟    ◆   BW.02.9   
  ∟    ◆   BW.03.9   
  ∟    ◆   BW.04.9   
  ∟    ◆   BW.05.9   
  ∟    ◆   BW.06.9   
  ∟    ◆   BW.09.9   
  ▶     ■    10   
  ∟    ◆   BW.02.10   
  ∟    ◆   BW.03.10   
  ∟    ◆   BW.04.10   
  ∟    ◆   BW.10.10   
   
 
 
 If the lines for some categories do not extend all the way to the right end of the x-axis, that means that at least one of the samples in that category does not have that many samples. 
 
  
 
  LibCodes  Seqs/Sample 
 chao1 Ave.  chao1 Err. 
 observed_species Ave.  observed_species Err. 
 shannon Ave.  shannon Err. 
 
 
 Lib_L_27F-001  10.0 
      8.932         nan 
      5.450         nan 
      2.144         nan 
 
 Lib_L_27F-001  111.0 
     38.424         nan 
     20.010         nan 
      3.040         nan 
 
 Lib_L_27F-001  212.0 
     53.999         nan 
     28.290         nan 
      3.175         nan 
 
 Lib_L_27F-001  313.0 
     60.567         nan 
     34.140         nan 
      3.235         nan 
 
 Lib_L_27F-001  414.0 
     70.652         nan 
     39.050         nan 
      3.271         nan 
 
 Lib_L_27F-001  515.0 
     74.640         nan 
     43.260         nan 
      3.289         nan 
 
 Lib_L_27F-001  616.0 
     77.557         nan 
     46.770         nan 
      3.301         nan 
 
 Lib_L_27F-001  717.0 
     87.518         nan 
     50.210         nan 
      3.313         nan 
 
 Lib_L_27F-001  818.0 
     86.669         nan 
     53.450         nan 
      3.327         nan 
 
 Lib_L_27F-001  919.0 
     89.987         nan 
     56.030         nan 
      3.328         nan 
 
 Lib_L_27F-001  1020.0 
     95.425         nan 
     58.990         nan 
      3.341         nan 
 
 Lib_L_27F-003  10.0 
     16.875       1.600 
      7.040       0.670 
      2.568       0.243 
 
 Lib_L_27F-003  111.0 
    134.970       0.047 
     41.545       1.415 
      4.154       0.295 
 
 Lib_L_27F-003  212.0 
    187.467       1.157 
     64.785       2.445 
      4.416       0.320 
 
 Lib_L_27F-003  313.0 
    205.231       4.590 
     83.970       3.840 
      4.534       0.337 
 
 Lib_L_27F-003  414.0 
    230.468      10.485 
    101.570       3.630 
      4.637       0.313 
 
 Lib_L_27F-003  515.0 
    241.464       4.458 
    116.040       3.920 
      4.684       0.322 
 
 Lib_L_27F-003  616.0 
    254.296       8.300 
    129.170       4.730 
      4.739       0.331 
 
 Lib_L_27F-003  717.0 
    265.427      11.959 
    142.480       5.240 
      4.787       0.337 
 
 Lib_L_27F-003  818.0 
    276.649      10.066 
    152.890       6.070 
      4.803       0.344 
 
 Lib_L_27F-003  919.0 
    282.791      12.195 
    163.115       5.645 
      4.832       0.337 
 
 Lib_L_27F-003  1020.0 
    294.138      15.346 
    172.780       5.980 
      4.853       0.336 
 
 Lib_L_27F-004  10.0 
      2.140         nan 
      1.810         nan 
      0.379         nan 
 
 Lib_L_27F-004  111.0 
     22.669         nan 
      8.780         nan 
      0.657         nan 
 
 Lib_L_27F-004  212.0 
     36.394         nan 
     13.140         nan 
      0.645         nan 
 
 Lib_L_27F-004  313.0 
     41.327         nan 
     17.790         nan 
      0.702         nan 
 
 Lib_L_27F-004  414.0 
     45.250         nan 
     21.890         nan 
      0.731         nan 
 
 Lib_L_27F-004  515.0 
     46.259         nan 
     24.490         nan 
      0.728         nan 
 
 Lib_L_27F-004  616.0 
     47.142         nan 
     27.600         nan 
      0.748         nan 
 
 Lib_L_27F-004  717.0 
     50.037         nan 
     30.370         nan 
      0.763         nan 
 
 Lib_L_27F-004  818.0 
     49.868         nan 
     32.610         nan 
      0.763         nan 
 
 Lib_L_27F-004  919.0 
     50.341         nan 
     34.460         nan 
      0.769         nan 
 
 Lib_L_27F-004  1020.0 
     51.062         nan 
     35.950         nan 
      0.769         nan 
 
 Lib_L_27F-005  10.0 
     13.500         nan 
      6.380         nan 
      2.376         nan 
 
 Lib_L_27F-005  111.0 
    113.583         nan 
     35.900         nan 
      3.832         nan 
 
 Lib_L_27F-005  212.0 
    151.590         nan 
     54.640         nan 
      4.032         nan 
 
 Lib_L_27F-005  313.0 
    195.678         nan 
     72.730         nan 
      4.176         nan 
 
 Lib_L_27F-005  414.0 
    226.829         nan 
     87.780         nan 
      4.258         nan 
 
 Lib_L_27F-005  515.0 
    248.135         nan 
    102.090         nan 
      4.326         nan 
 
 Lib_L_27F-005  616.0 
    253.505         nan 
    113.340         nan 
      4.352         nan 
 
 Lib_L_27F-005  717.0 
    260.694         nan 
    124.080         nan 
      4.373         nan 
 
 Lib_L_27F-005  818.0 
    280.385         nan 
    135.170         nan 
      4.409         nan 
 
 Lib_L_27F-005  919.0 
    278.097         nan 
    144.800         nan 
      4.433         nan 
 
 Lib_L_27F-005  1020.0 
    280.351         nan 
    153.820         nan 
      4.450         nan 
 
 Lib_L_27F-006  10.0 
     14.378         nan 
      6.570         nan 
      2.448         nan 
 
 Lib_L_27F-006  111.0 
    107.749         nan 
     35.320         nan 
      3.868         nan 
 
 Lib_L_27F-006  212.0 
    152.383         nan 
     55.540         nan 
      4.125         nan 
 
 Lib_L_27F-006  313.0 
    189.175         nan 
     70.740         nan 
      4.196         nan 
 
 Lib_L_27F-006  414.0 
    213.498         nan 
     85.970         nan 
      4.287         nan 
 
 Lib_L_27F-006  515.0 
    214.121         nan 
     99.370         nan 
      4.357         nan 
 
 Lib_L_27F-006  616.0 
    227.671         nan 
    110.260         nan 
      4.381         nan 
 
 Lib_L_27F-006  717.0 
    229.488         nan 
    121.280         nan 
      4.420         nan 
 
 Lib_L_27F-006  818.0 
    236.445         nan 
    130.410         nan 
      4.437         nan 
 
 Lib_L_27F-006  919.0 
    235.584         nan 
    139.610         nan 
      4.461         nan 
 
 Lib_L_27F-006  1020.0 
    241.387         nan 
    147.840         nan 
      4.482         nan 
 
 Lib_L_27F-008  10.0 
     13.723         nan 
      5.870         nan 
      2.147         nan 
 
 Lib_L_27F-008  111.0 
     90.891         nan 
     33.470         nan 
      3.388         nan 
 
 Lib_L_27F-008  212.0 
    140.029         nan 
     51.450         nan 
      3.581         nan 
 
 Lib_L_27F-008  313.0 
    170.023         nan 
     65.400         nan 
      3.672         nan 
 
 Lib_L_27F-008  414.0 
    187.999         nan 
     78.780         nan 
      3.749         nan 
 
 Lib_L_27F-008  515.0 
    211.538         nan 
     90.470         nan 
      3.805         nan 
 
 Lib_L_27F-008  616.0 
    227.754         nan 
    100.900         nan 
      3.852         nan 
 
 Lib_L_27F-008  717.0 
    247.142         nan 
    110.650         nan 
      3.880         nan 
 
 Lib_L_27F-008  818.0 
    270.222         nan 
    119.610         nan 
      3.900         nan 
 
 Lib_L_27F-008  919.0 
    282.354         nan 
    129.070         nan 
      3.931         nan 
 
 Lib_L_27F-008  1020.0 
    298.684         nan 
    136.850         nan 
      3.941         nan 
 
 Lib_L_27F-011  10.0 
      5.957         nan 
      3.920         nan 
      1.456         nan 
 
 Lib_L_27F-011  111.0 
     34.947         nan 
     15.230         nan 
      2.029         nan 
 
 Lib_L_27F-011  212.0 
     43.986         nan 
     22.460         nan 
      2.137         nan 
 
 Lib_L_27F-011  313.0 
     57.696         nan 
     28.260         nan 
      2.181         nan 
 
 Lib_L_27F-011  414.0 
     73.314         nan 
     33.200         nan 
      2.227         nan 
 
 Lib_L_27F-011  515.0 
     73.949         nan 
     36.890         nan 
      2.228         nan 
 
 Lib_L_27F-011  616.0 
     90.677         nan 
     40.940         nan 
      2.245         nan 
 
 Lib_L_27F-011  717.0 
     91.648         nan 
     44.310         nan 
      2.251         nan 
 
 Lib_L_27F-011  818.0 
     93.969         nan 
     47.660         nan 
      2.257         nan 
 
 Lib_L_27F-011  919.0 
     97.062         nan 
     50.940         nan 
      2.272         nan 
 
 Lib_L_27F-011  1020.0 
     94.643         nan 
     53.950         nan 
      2.278         nan 
 
 Lib_L_27F-013  10.0 
      5.467         nan 
      3.790         nan 
      1.450         nan 
 
 Lib_L_27F-013  111.0 
     31.941         nan 
     15.530         nan 
      2.069         nan 
 
 Lib_L_27F-013  212.0 
     42.161         nan 
     21.830         nan 
      2.115         nan 
 
 Lib_L_27F-013  313.0 
     52.391         nan 
     27.610         nan 
      2.161         nan 
 
 Lib_L_27F-013  414.0 
     59.756         nan 
     31.600         nan 
      2.161         nan 
 
 Lib_L_27F-013  515.0 
     60.125         nan 
     35.440         nan 
      2.195         nan 
 
 Lib_L_27F-013  616.0 
     63.686         nan 
     38.760         nan 
      2.205         nan 
 
 Lib_L_27F-013  717.0 
     67.033         nan 
     41.790         nan 
      2.225         nan 
 
 Lib_L_27F-013  818.0 
     67.816         nan 
     44.600         nan 
      2.235         nan 
 
 Lib_L_27F-013  919.0 
     68.491         nan 
     46.650         nan 
      2.240         nan 
 
 Lib_L_27F-013  1020.0 
     69.985         nan 
     48.980         nan 
      2.244         nan 
 
 Lib_L_27F-014  10.0 
      8.358         nan 
      4.910         nan 
      1.897         nan 
 
 Lib_L_27F-014  111.0 
     55.237         nan 
     22.330         nan 
      2.740         nan 
 
 Lib_L_27F-014  212.0 
     68.257         nan 
     33.670         nan 
      2.888         nan 
 
 Lib_L_27F-014  313.0 
     75.808         nan 
     42.020         nan 
      2.943         nan 
 
 Lib_L_27F-014  414.0 
     85.018         nan 
     48.580         nan 
      2.973         nan 
 
 Lib_L_27F-014  515.0 
     94.497         nan 
     54.580         nan 
      2.993         nan 
 
 Lib_L_27F-014  616.0 
    106.808         nan 
     60.420         nan 
      3.030         nan 
 
 Lib_L_27F-014  717.0 
    114.999         nan 
     65.130         nan 
      3.045         nan 
 
 Lib_L_27F-014  818.0 
    119.053         nan 
     68.960         nan 
      3.054         nan 
 
 Lib_L_27F-014  919.0 
    131.723         nan 
     73.180         nan 
      3.064         nan 
 
 Lib_L_27F-014  1020.0 
    142.857         nan 
     76.930         nan 
      3.079         nan 
 
 Lib_L_27F-015  10.0 
      6.550         nan 
      4.380         nan 
      1.769         nan 
 
 Lib_L_27F-015  111.0 
     46.050         nan 
     19.620         nan 
      2.587         nan 
 
 Lib_L_27F-015  212.0 
     70.754         nan 
     28.300         nan 
      2.666         nan 
 
 Lib_L_27F-015  313.0 
     87.388         nan 
     35.540         nan 
      2.733         nan 
 
 Lib_L_27F-015  414.0 
     99.452         nan 
     42.440         nan 
      2.774         nan 
 
 Lib_L_27F-015  515.0 
    107.519         nan 
     49.130         nan 
      2.804         nan 
 
 Lib_L_27F-015  616.0 
    111.082         nan 
     54.150         nan 
      2.812         nan 
 
 Lib_L_27F-015  717.0 
    107.190         nan 
     58.810         nan 
      2.825         nan 
 
 Lib_L_27F-015  818.0 
    103.966         nan 
     63.060         nan 
      2.841         nan 
 
 Lib_L_27F-015  919.0 
    101.009         nan 
     67.090         nan 
      2.847         nan 
 
 Lib_L_27F-015  1020.0 
    100.734         nan 
     70.940         nan 
      2.856         nan 
 
 Lib_L_27F-017  10.0 
      4.285         nan 
      2.950         nan 
      0.922         nan 
 
 Lib_L_27F-017  111.0 
     34.790         nan 
     14.980         nan 
      1.349         nan 
 
 Lib_L_27F-017  212.0 
     53.743         nan 
     23.200         nan 
      1.433         nan 
 
 Lib_L_27F-017  313.0 
     70.492         nan 
     30.240         nan 
      1.503         nan 
 
 Lib_L_27F-017  414.0 
     78.128         nan 
     36.310         nan 
      1.547         nan 
 
 Lib_L_27F-017  515.0 
     92.622         nan 
     40.580         nan 
      1.545         nan 
 
 Lib_L_27F-017  616.0 
    100.248         nan 
     44.500         nan 
      1.542         nan 
 
 Lib_L_27F-017  717.0 
    104.564         nan 
     49.400         nan 
      1.571         nan 
 
 Lib_L_27F-017  818.0 
    103.588         nan 
     53.350         nan 
      1.584         nan 
 
 Lib_L_27F-017  919.0 
    103.897         nan 
     56.620         nan 
      1.586         nan 
 
 Lib_L_27F-017  1020.0 
    100.680         nan 
     59.930         nan 
      1.594         nan 
 
 Lib_L_27F-018  10.0 
      4.469       0.164 
      3.290       0.340 
      1.214       0.299 
 
 Lib_L_27F-018  111.0 
     28.380       0.978 
     13.165       0.425 
      1.628       0.345 
 
 Lib_L_27F-018  212.0 
     42.562       4.150 
     19.300       0.440 
      1.713       0.338 
 
 Lib_L_27F-018  313.0 
     51.391       2.145 
     24.410       0.450 
      1.753       0.342 
 
 Lib_L_27F-018  414.0 
     54.974       0.607 
     28.165       0.655 
      1.768       0.336 
 
 Lib_L_27F-018  515.0 
     60.332       3.583 
     31.955       0.595 
      1.789       0.332 
 
 Lib_L_27F-018  616.0 
     63.084       4.010 
     34.765       0.705 
      1.793       0.339 
 
 Lib_L_27F-018  717.0 
     69.037       2.674 
     38.040       0.720 
      1.814       0.334 
 
 Lib_L_27F-018  818.0 
     73.341       4.948 
     40.750       0.580 
      1.818       0.337 
 
 Lib_L_27F-018  919.0 
     79.277       5.793 
     43.265       0.605 
      1.824       0.341 
 
 Lib_L_27F-018  1020.0 
     81.785       2.832 
     45.470       0.500 
      1.828       0.339 
 
 Lib_L_27F-019  10.0 
      5.965         nan 
      3.580         nan 
      1.212         nan 
 
 Lib_L_27F-019  111.0 
     42.434         nan 
     18.610         nan 
      1.994         nan 
 
 Lib_L_27F-019  212.0 
     53.681         nan 
     26.370         nan 
      2.125         nan 
 
 Lib_L_27F-019  313.0 
     65.351         nan 
     32.040         nan 
      2.141         nan 
 
 Lib_L_27F-019  414.0 
     67.423         nan 
     36.510         nan 
      2.169         nan 
 
 Lib_L_27F-019  515.0 
     77.934         nan 
     41.880         nan 
      2.196         nan 
 
 Lib_L_27F-019  616.0 
     83.123         nan 
     45.350         nan 
      2.214         nan 
 
 Lib_L_27F-019  717.0 
     93.775         nan 
     48.980         nan 
      2.211         nan 
 
 Lib_L_27F-019  818.0 
    100.879         nan 
     52.730         nan 
      2.235         nan 
 
 Lib_L_27F-019  919.0 
    104.109         nan 
     55.870         nan 
      2.243         nan 
 
 Lib_L_27F-019  1020.0 
    113.083         nan 
     58.950         nan 
      2.246         nan 
 
 Lib_L_27F-020  10.0 
      1.980         nan 
      1.740         nan 
      0.346         nan 
 
 Lib_L_27F-020  111.0 
     13.235         nan 
      7.390         nan 
      0.603         nan 
 
 Lib_L_27F-020  212.0 
     19.492         nan 
     10.840         nan 
      0.647         nan 
 
 Lib_L_27F-020  313.0 
     23.328         nan 
     13.190         nan 
      0.648         nan 
 
 Lib_L_27F-020  414.0 
     30.843         nan 
     15.580         nan 
      0.666         nan 
 
 Lib_L_27F-020  515.0 
     32.561         nan 
     17.570         nan 
      0.675         nan 
 
 Lib_L_27F-020  616.0 
     32.885         nan 
     19.620         nan 
      0.690         nan 
 
 Lib_L_27F-020  717.0 
     35.522         nan 
     20.800         nan 
      0.688         nan 
 
 Lib_L_27F-020  818.0 
     34.169         nan 
     22.330         nan 
      0.696         nan 
 
 Lib_L_27F-020  919.0 
     37.696         nan 
     23.700         nan 
      0.696         nan 
 
 Lib_L_27F-020  1020.0 
     38.080         nan 
     24.990         nan 
      0.701         nan 
 
 Lib_L_27F-021  10.0 
      9.133         nan 
      4.830         nan 
      1.796         nan 
 
 Lib_L_27F-021  111.0 
     69.459         nan 
     25.430         nan 
      2.633         nan 
 
 Lib_L_27F-021  212.0 
    103.522         nan 
     40.860         nan 
      2.840         nan 
 
 Lib_L_27F-021  313.0 
    119.800         nan 
     51.670         nan 
      2.871         nan 
 
 Lib_L_27F-021  414.0 
    129.456         nan 
     61.850         nan 
      2.915         nan 
 
 Lib_L_27F-021  515.0 
    130.565         nan 
     70.180         nan 
      2.964         nan 
 
 Lib_L_27F-021  616.0 
    143.092         nan 
     77.940         nan 
      2.983         nan 
 
 Lib_L_27F-021  717.0 
    150.467         nan 
     84.590         nan 
      3.002         nan 
 
 Lib_L_27F-021  818.0 
    157.851         nan 
     91.100         nan 
      3.024         nan 
 
 Lib_L_27F-021  919.0 
    160.994         nan 
     96.570         nan 
      3.037         nan 
 
 Lib_L_27F-021  1020.0 
    165.686         nan 
    101.940         nan 
      3.052         nan 
 
 Lib_L_27F-024  10.0 
      2.220         nan 
      1.870         nan 
      0.405         nan 
 
 Lib_L_27F-024  111.0 
     20.246         nan 
      8.420         nan 
      0.639         nan 
 
 Lib_L_27F-024  212.0 
     33.517         nan 
     13.590         nan 
      0.706         nan 
 
 Lib_L_27F-024  313.0 
     39.976         nan 
     17.790         nan 
      0.737         nan 
 
 Lib_L_27F-024  414.0 
     43.512         nan 
     21.340         nan 
      0.765         nan 
 
 Lib_L_27F-024  515.0 
     51.468         nan 
     24.860         nan 
      0.785         nan 
 
 Lib_L_27F-024  616.0 
     51.627         nan 
     26.810         nan 
      0.785         nan 
 
 Lib_L_27F-024  717.0 
     53.828         nan 
     29.730         nan 
      0.794         nan 
 
 Lib_L_27F-024  818.0 
     57.726         nan 
     32.080         nan 
      0.800         nan 
 
 Lib_L_27F-024  919.0 
     55.957         nan 
     34.080         nan 
      0.804         nan 
 
 Lib_L_27F-024  1020.0 
     57.508         nan 
     35.970         nan 
      0.808         nan 
 
 Lib_L_27F-026  10.0 
      8.458         nan 
      4.360         nan 
      1.533         nan 
 
 Lib_L_27F-026  111.0 
     48.030         nan 
     21.970         nan 
      2.413         nan 
 
 Lib_L_27F-026  212.0 
     62.898         nan 
     31.220         nan 
      2.515         nan 
 
 Lib_L_27F-026  313.0 
     81.406         nan 
     38.960         nan 
      2.570         nan 
 
 Lib_L_27F-026  414.0 
     97.041         nan 
     46.020         nan 
      2.623         nan 
 
 Lib_L_27F-026  515.0 
    103.858         nan 
     51.820         nan 
      2.620         nan 
 
 Lib_L_27F-026  616.0 
    115.595         nan 
     56.830         nan 
      2.660         nan 
 
 Lib_L_27F-026  717.0 
    127.255         nan 
     61.020         nan 
      2.674         nan 
 
 Lib_L_27F-026  818.0 
    134.682         nan 
     65.910         nan 
      2.676         nan 
 
 Lib_L_27F-026  919.0 
    139.092         nan 
     69.980         nan 
      2.691         nan 
 
 Lib_L_27F-026  1020.0 
    148.180         nan 
     73.940         nan 
      2.700         nan 
 
 Lib_L_27F-027  10.0 
      9.062         nan 
      4.910         nan 
      1.803         nan 
 
 Lib_L_27F-027  111.0 
     53.906         nan 
     23.840         nan 
      2.712         nan 
 
 Lib_L_27F-027  212.0 
     83.449         nan 
     35.270         nan 
      2.860         nan 
 
 Lib_L_27F-027  313.0 
    101.474         nan 
     44.940         nan 
      2.932         nan 
 
 Lib_L_27F-027  414.0 
    111.865         nan 
     52.480         nan 
      2.968         nan 
 
 Lib_L_27F-027  515.0 
    128.644         nan 
     60.030         nan 
      3.007         nan 
 
 Lib_L_27F-027  616.0 
    134.414         nan 
     65.870         nan 
      3.022         nan 
 
 Lib_L_27F-027  717.0 
    152.323         nan 
     73.070         nan 
      3.048         nan 
 
 Lib_L_27F-027  818.0 
    164.916         nan 
     78.480         nan 
      3.064         nan 
 
 Lib_L_27F-027  919.0 
    182.043         nan 
     84.160         nan 
      3.088         nan 
 
 Lib_L_27F-027  1020.0 
    200.065         nan 
     88.930         nan 
      3.093         nan 
 
 Lib_L_27F-030  10.0 
      8.050         nan 
      5.230         nan 
      2.101         nan 
 
 Lib_L_27F-030  111.0 
     56.439         nan 
     22.980         nan 
      3.023         nan 
 
 Lib_L_27F-030  212.0 
     75.439         nan 
     35.800         nan 
      3.223         nan 
 
 Lib_L_27F-030  313.0 
     83.578         nan 
     43.990         nan 
      3.266         nan 
 
 Lib_L_27F-030  414.0 
    100.656         nan 
     51.350         nan 
      3.300         nan 
 
 Lib_L_27F-030  515.0 
    116.510         nan 
     57.820         nan 
      3.318         nan 
 
 Lib_L_27F-030  616.0 
    132.851         nan 
     63.580         nan 
      3.345         nan 
 
 Lib_L_27F-030  717.0 
    136.433         nan 
     69.020         nan 
      3.377         nan 
 
 Lib_L_27F-030  818.0 
    143.582         nan 
     74.050         nan 
      3.390         nan 
 
 Lib_L_27F-030  919.0 
    150.550         nan 
     78.640         nan 
      3.396         nan 
 
 Lib_L_27F-030  1020.0 
    152.723         nan 
     82.910         nan 
      3.408         nan 
 
 Lib_L_27F-031  10.0 
      8.508         nan 
      4.900         nan 
      1.892         nan 
 
 Lib_L_27F-031  111.0 
     40.724         nan 
     20.990         nan 
      2.697         nan 
 
 Lib_L_27F-031  212.0 
     65.028         nan 
     30.940         nan 
      2.856         nan 
 
 Lib_L_27F-031  313.0 
     70.933         nan 
     37.000         nan 
      2.891         nan 
 
 Lib_L_27F-031  414.0 
     84.148         nan 
     43.620         nan 
      2.926         nan 
 
 Lib_L_27F-031  515.0 
     97.748         nan 
     48.320         nan 
      2.949         nan 
 
 Lib_L_27F-031  616.0 
    103.428         nan 
     53.100         nan 
      2.966         nan 
 
 Lib_L_27F-031  717.0 
    106.703         nan 
     57.520         nan 
      2.984         nan 
 
 Lib_L_27F-031  818.0 
    120.400         nan 
     61.700         nan 
      2.999         nan 
 
 Lib_L_27F-031  919.0 
    125.316         nan 
     65.540         nan 
      3.003         nan 
 
 Lib_L_27F-031  1020.0 
    128.304         nan 
     68.920         nan 
      3.013         nan 
 
 Lib_L_27F-033  10.0 
     13.557         nan 
      6.380         nan 
      2.402         nan 
 
 Lib_L_27F-033  111.0 
    107.452         nan 
     34.990         nan 
      3.780         nan 
 
 Lib_L_27F-033  212.0 
    138.971         nan 
     54.000         nan 
      3.968         nan 
 
 Lib_L_27F-033  313.0 
    171.448         nan 
     71.360         nan 
      4.108         nan 
 
 Lib_L_27F-033  414.0 
    183.803         nan 
     83.630         nan 
      4.162         nan 
 
 Lib_L_27F-033  515.0 
    202.388         nan 
     95.410         nan 
      4.216         nan 
 
 Lib_L_27F-033  616.0 
    208.528         nan 
    105.410         nan 
      4.241         nan 
 
 Lib_L_27F-033  717.0 
    223.689         nan 
    115.780         nan 
      4.279         nan 
 
 Lib_L_27F-033  818.0 
    235.559         nan 
    124.990         nan 
      4.313         nan 
 
 Lib_L_27F-033  919.0 
    251.040         nan 
    133.530         nan 
      4.324         nan 
 
 Lib_L_27F-033  1020.0 
    254.594         nan 
    140.830         nan 
      4.343         nan 
 
 Lib_L_27F-034  10.0 
     11.012         nan 
      6.120         nan 
      2.367         nan 
 
 Lib_L_27F-034  111.0 
     97.595         nan 
     28.710         nan 
      3.463         nan 
 
 Lib_L_27F-034  212.0 
    122.103         nan 
     44.880         nan 
      3.644         nan 
 
 Lib_L_27F-034  313.0 
    142.655         nan 
     59.010         nan 
      3.747         nan 
 
 Lib_L_27F-034  414.0 
    157.261         nan 
     70.520         nan 
      3.802         nan 
 
 Lib_L_27F-034  515.0 
    166.979         nan 
     80.930         nan 
      3.838         nan 
 
 Lib_L_27F-034  616.0 
    175.510         nan 
     90.330         nan 
      3.875         nan 
 
 Lib_L_27F-034  717.0 
    183.228         nan 
     99.310         nan 
      3.907         nan 
 
 Lib_L_27F-034  818.0 
    191.016         nan 
    107.860         nan 
      3.938         nan 
 
 Lib_L_27F-034  919.0 
    188.544         nan 
    114.160         nan 
      3.944         nan 
 
 Lib_L_27F-034  1020.0 
    190.769         nan 
    120.830         nan 
      3.962         nan 
 
 Lib_L_27F-035  10.0 
      5.810         nan 
      3.820         nan 
      1.425         nan 
 
 Lib_L_27F-035  111.0 
     39.924         nan 
     18.090         nan 
      2.080         nan 
 
 Lib_L_27F-035  212.0 
     59.661         nan 
     27.510         nan 
      2.213         nan 
 
 Lib_L_27F-035  313.0 
     73.312         nan 
     33.950         nan 
      2.261         nan 
 
 Lib_L_27F-035  414.0 
     94.949         nan 
     39.990         nan 
      2.295         nan 
 
 Lib_L_27F-035  515.0 
    102.657         nan 
     44.870         nan 
      2.318         nan 
 
 Lib_L_27F-035  616.0 
    105.447         nan 
     49.750         nan 
      2.337         nan 
 
 Lib_L_27F-035  717.0 
    109.861         nan 
     53.860         nan 
      2.340         nan 
 
 Lib_L_27F-035  818.0 
    114.001         nan 
     58.380         nan 
      2.355         nan 
 
 Lib_L_27F-035  919.0 
    117.204         nan 
     62.000         nan 
      2.362         nan 
 
 Lib_L_27F-035  1020.0 
    123.416         nan 
     65.960         nan 
      2.373         nan 
 
 Lib_L_27F-036  10.0 
      5.570         nan 
      4.080         nan 
      1.671         nan 
 
 Lib_L_27F-036  111.0 
     34.220         nan 
     15.200         nan 
      2.208         nan 
 
 Lib_L_27F-036  212.0 
     53.576         nan 
     23.580         nan 
      2.354         nan 
 
 Lib_L_27F-036  313.0 
     61.141         nan 
     28.450         nan 
      2.355         nan 
 
 Lib_L_27F-036  414.0 
     71.864         nan 
     34.580         nan 
      2.402         nan 
 
 Lib_L_27F-036  515.0 
     78.584         nan 
     39.420         nan 
      2.417         nan 
 
 Lib_L_27F-036  616.0 
     78.673         nan 
     43.780         nan 
      2.437         nan 
 
 Lib_L_27F-036  717.0 
     80.728         nan 
     47.170         nan 
      2.440         nan 
 
 Lib_L_27F-036  818.0 
     86.383         nan 
     50.840         nan 
      2.457         nan 
 
 Lib_L_27F-036  919.0 
     86.392         nan 
     53.880         nan 
      2.463         nan 
 
 Lib_L_27F-036  1020.0 
     88.325         nan 
     56.950         nan 
      2.473         nan 
 
 Lib_L_27F-037  10.0 
     12.245         nan 
      6.250         nan 
      2.388         nan 
 
 Lib_L_27F-037  111.0 
    110.047         nan 
     34.790         nan 
      3.668         nan 
 
 Lib_L_27F-037  212.0 
    139.497         nan 
     55.290         nan 
      3.920         nan 
 
 Lib_L_27F-037  313.0 
    161.900         nan 
     71.960         nan 
      4.045         nan 
 
 Lib_L_27F-037  414.0 
    176.660         nan 
     84.690         nan 
      4.092         nan 
 
 Lib_L_27F-037  515.0 
    197.600         nan 
     97.130         nan 
      4.157         nan 
 
 Lib_L_27F-037  616.0 
    214.821         nan 
    108.060         nan 
      4.185         nan 
 
 Lib_L_27F-037  717.0 
    231.937         nan 
    118.010         nan 
      4.220         nan 
 
 Lib_L_27F-037  818.0 
    232.853         nan 
    126.820         nan 
      4.248         nan 
 
 Lib_L_27F-037  919.0 
    245.406         nan 
    135.130         nan 
      4.268         nan 
 
 Lib_L_27F-037  1020.0 
    252.232         nan 
    142.830         nan 
      4.283         nan 
 
 Lib_L_27F-038  10.0 
      6.345         nan 
      4.010         nan 
      1.483         nan 
 
 Lib_L_27F-038  111.0 
     29.746         nan 
     16.160         nan 
      2.160         nan 
 
 Lib_L_27F-038  212.0 
     44.191         nan 
     22.160         nan 
      2.237         nan 
 
 Lib_L_27F-038  313.0 
     55.093         nan 
     26.730         nan 
      2.279         nan 
 
 Lib_L_27F-038  414.0 
     62.980         nan 
     30.860         nan 
      2.294         nan 
 
 Lib_L_27F-038  515.0 
     70.077         nan 
     34.820         nan 
      2.325         nan 
 
 Lib_L_27F-038  616.0 
     80.735         nan 
     38.650         nan 
      2.340         nan 
 
 Lib_L_27F-038  717.0 
     87.611         nan 
     41.460         nan 
      2.342         nan 
 
 Lib_L_27F-038  818.0 
     88.859         nan 
     44.430         nan 
      2.353         nan 
 
 Lib_L_27F-038  919.0 
     87.912         nan 
     47.280         nan 
      2.362         nan 
 
 Lib_L_27F-038  1020.0 
     91.072         nan 
     49.970         nan 
      2.368         nan 
 
 Lib_L_27F-039  10.0 
      5.783         nan 
      4.140         nan 
      1.692         nan 
 
 Lib_L_27F-039  111.0 
     54.538         nan 
     18.250         nan 
      2.359         nan 
 
 Lib_L_27F-039  212.0 
     70.568         nan 
     27.490         nan 
      2.454         nan 
 
 Lib_L_27F-039  313.0 
     84.073         nan 
     36.270         nan 
      2.513         nan 
 
 Lib_L_27F-039  414.0 
     85.183         nan 
     43.350         nan 
      2.565         nan 
 
 Lib_L_27F-039  515.0 
     89.642         nan 
     49.660         nan 
      2.590         nan 
 
 Lib_L_27F-039  616.0 
     86.395         nan 
     54.560         nan 
      2.608         nan 
 
 Lib_L_27F-039  717.0 
     90.032         nan 
     59.300         nan 
      2.631         nan 
 
 Lib_L_27F-039  818.0 
     91.484         nan 
     63.440         nan 
      2.638         nan 
 
 Lib_L_27F-039  919.0 
     90.262         nan 
     66.900         nan 
      2.647         nan 
 
 Lib_L_27F-039  1020.0 
     90.407         nan 
     69.960         nan 
      2.655         nan 
 
 Lib_L_27F-040  10.0 
      7.863         nan 
      4.430         nan 
      1.670         nan 
 
 Lib_L_27F-040  111.0 
     57.776         nan 
     23.600         nan 
      2.653         nan 
 
 Lib_L_27F-040  212.0 
     76.485         nan 
     36.060         nan 
      2.778         nan 
 
 Lib_L_27F-040  313.0 
     86.717         nan 
     45.550         nan 
      2.887         nan 
 
 Lib_L_27F-040  414.0 
    106.526         nan 
     54.200         nan 
      2.921         nan 
 
 Lib_L_27F-040  515.0 
    119.204         nan 
     60.220         nan 
      2.949         nan 
 
 Lib_L_27F-040  616.0 
    135.464         nan 
     66.670         nan 
      2.971         nan 
 
 Lib_L_27F-040  717.0 
    142.388         nan 
     71.850         nan 
      2.971         nan 
 
 Lib_L_27F-040  818.0 
    151.896         nan 
     77.000         nan 
      2.993         nan 
 
 Lib_L_27F-040  919.0 
    161.466         nan 
     82.010         nan 
      3.007         nan 
 
 Lib_L_27F-040  1020.0 
    173.352         nan 
     86.940         nan 
      3.019         nan 
 
 Lib_L_27F-041  10.0 
      9.718         nan 
      5.080         nan 
      1.899         nan 
 
 Lib_L_27F-041  111.0 
     77.801         nan 
     25.970         nan 
      2.882         nan 
 
 Lib_L_27F-041  212.0 
    110.192         nan 
     40.780         nan 
      3.075         nan 
 
 Lib_L_27F-041  313.0 
    127.549         nan 
     52.330         nan 
      3.125         nan 
 
 Lib_L_27F-041  414.0 
    145.060         nan 
     63.030         nan 
      3.202         nan 
 
 Lib_L_27F-041  515.0 
    158.675         nan 
     72.810         nan 
      3.242         nan 
 
 Lib_L_27F-041  616.0 
    172.471         nan 
     81.010         nan 
      3.264         nan 
 
 Lib_L_27F-041  717.0 
    180.183         nan 
     88.820         nan 
      3.290         nan 
 
 Lib_L_27F-041  818.0 
    187.330         nan 
     95.820         nan 
      3.306         nan 
 
 Lib_L_27F-041  919.0 
    194.999         nan 
    102.950         nan 
      3.329         nan 
 
 Lib_L_27F-041  1020.0 
    205.074         nan 
    108.830         nan 
      3.338         nan 
 
 Lib_L_27F-042  10.0 
      5.583         nan 
      3.460         nan 
      1.154         nan 
 
 Lib_L_27F-042  111.0 
     39.535         nan 
     17.870         nan 
      1.808         nan 
 
 Lib_L_27F-042  212.0 
     52.523         nan 
     25.890         nan 
      1.908         nan 
 
 Lib_L_27F-042  313.0 
     65.529         nan 
     32.670         nan 
      1.940         nan 
 
 Lib_L_27F-042  414.0 
     82.514         nan 
     39.060         nan 
      2.008         nan 
 
 Lib_L_27F-042  515.0 
     88.046         nan 
     43.790         nan 
      2.025         nan 
 
 Lib_L_27F-042  616.0 
     91.828         nan 
     48.330         nan 
      2.038         nan 
 
 Lib_L_27F-042  717.0 
     99.071         nan 
     52.280         nan 
      2.046         nan 
 
 Lib_L_27F-042  818.0 
    104.453         nan 
     56.010         nan 
      2.055         nan 
 
 Lib_L_27F-042  919.0 
    113.388         nan 
     59.740         nan 
      2.066         nan 
 
 Lib_L_27F-042  1020.0 
    121.633         nan 
     62.930         nan 
      2.070         nan 
 
 Lib_L_27F-051  10.0 
      9.227       4.062 
      4.785       1.385 
      1.735       0.560 
 
 Lib_L_27F-051  111.0 
     64.347      24.567 
     24.325       9.155 
      2.670       0.933 
 
 Lib_L_27F-051  212.0 
     78.366      23.139 
     36.585      13.285 
      2.799       0.983 
 
 Lib_L_27F-051  313.0 
     95.417      34.216 
     46.670      16.490 
      2.888       1.003 
 
 Lib_L_27F-051  414.0 
    102.638      39.263 
     54.600      18.600 
      2.945       1.008 
 
 Lib_L_27F-051  515.0 
    112.910      47.077 
     61.570      21.370 
      2.965       1.022 
 
 Lib_L_27F-051  616.0 
    119.944      54.090 
     67.030      23.110 
      2.983       1.026 
 
 Lib_L_27F-051  717.0 
    132.646      64.014 
     73.355      26.115 
      3.012       1.045 
 
 Lib_L_27F-051  818.0 
    139.209      71.344 
     77.820      27.660 
      3.018       1.042 
 
 Lib_L_27F-051  919.0 
    143.736      77.391 
     82.765      29.905 
      3.034       1.052 
 
 Lib_L_27F-051  1020.0 
    147.650      82.021 
     86.895      31.915 
      3.043       1.055 
 
  Innoculation  Seqs/Sample 
 chao1 Ave.  chao1 Err. 
 observed_species Ave.  observed_species Err. 
 shannon Ave.  shannon Err. 
 
 
 After  10.0 
      8.322       3.368 
      4.579       1.213 
      1.701       0.539 
 
 After  111.0 
     58.956      22.805 
     22.673       7.517 
      2.558       0.817 
 
 After  212.0 
     81.749      31.814 
     34.739      11.636 
      2.705       0.864 
 
 After  313.0 
     95.684      39.591 
     43.874      14.848 
      2.765       0.885 
 
 After  414.0 
    107.515      42.978 
     52.096      17.658 
      2.813       0.896 
 
 After  515.0 
    118.226      48.542 
     59.019      20.380 
      2.842       0.907 
 
 After  616.0 
    127.423      54.401 
     65.144      22.591 
      2.864       0.913 
 
 After  717.0 
    135.416      59.864 
     70.926      25.085 
      2.885       0.924 
 
 After  818.0 
    143.535      66.080 
     76.069      27.031 
      2.898       0.926 
 
 After  919.0 
    149.111      69.738 
     81.091      29.253 
      2.911       0.933 
 
 After  1020.0 
    154.761      74.650 
     85.513      31.111 
      2.921       0.935 
 
 Before  10.0 
      8.599       5.193 
      4.654       1.829 
      1.689       0.758 
 
 Before  111.0 
     58.223      40.947 
     22.144      11.346 
      2.545       1.199 
 
 Before  212.0 
     78.781      54.889 
     33.327      18.116 
      2.678       1.267 
 
 Before  313.0 
     93.918      62.460 
     42.806      24.163 
      2.748       1.307 
 
 Before  414.0 
    107.786      68.823 
     50.440      28.930 
      2.792       1.320 
 
 Before  515.0 
    113.791      71.569 
     57.087      33.176 
      2.821       1.334 
 
 Before  616.0 
    120.928      75.086 
     62.989      37.202 
      2.841       1.351 
 
 Before  717.0 
    128.596      79.719 
     68.831      41.248 
      2.862       1.365 
 
 Before  818.0 
    133.273      83.278 
     73.964      44.529 
      2.876       1.374 
 
 Before  919.0 
    138.702      87.479 
     78.533      47.478 
      2.886       1.377 
 
 Before  1020.0 
    143.325      90.922 
     82.916      50.294 
      2.896       1.384 
 
 During  10.0 
      8.111       3.796 
      4.534       1.369 
      1.672       0.582 
 
 During  111.0 
     60.451      35.479 
     22.398       9.082 
      2.520       0.940 
 
 During  212.0 
     83.878      46.186 
     33.865      14.632 
      2.657       1.003 
 
 During  313.0 
    100.331      53.393 
     43.179      19.360 
      2.723       1.027 
 
 During  414.0 
    112.832      59.840 
     51.346      23.655 
      2.769       1.048 
 
 During  515.0 
    122.774      63.161 
     58.657      27.510 
      2.798       1.066 
 
 During  616.0 
    129.820      65.636 
     64.874      30.709 
      2.819       1.075 
 
 During  717.0 
    136.778      67.015 
     70.898      33.904 
      2.839       1.082 
 
 During  818.0 
    142.362      70.557 
     76.314      36.591 
      2.854       1.088 
 
 During  919.0 
    145.912      70.989 
     81.307      39.289 
      2.866       1.095 
 
 During  1020.0 
    150.975      72.722 
     85.971      41.730 
      2.877       1.101 
 
  BarcodeSequence  Seqs/Sample 
 chao1 Ave.  chao1 Err. 
 observed_species Ave.  observed_species Err. 
 shannon Ave.  shannon Err. 
 
 
 ACATACGCGTAG  10.0 
      8.458         nan 
      4.360         nan 
      1.533         nan 
 
 ACATACGCGTAG  111.0 
     48.030         nan 
     21.970         nan 
      2.413         nan 
 
 ACATACGCGTAG  212.0 
     62.898         nan 
     31.220         nan 
      2.515         nan 
 
 ACATACGCGTAG  313.0 
     81.406         nan 
     38.960         nan 
      2.570         nan 
 
 ACATACGCGTAG  414.0 
     97.041         nan 
     46.020         nan 
      2.623         nan 
 
 ACATACGCGTAG  515.0 
    103.858         nan 
     51.820         nan 
      2.620         nan 
 
 ACATACGCGTAG  616.0 
    115.595         nan 
     56.830         nan 
      2.660         nan 
 
 ACATACGCGTAG  717.0 
    127.255         nan 
     61.020         nan 
      2.674         nan 
 
 ACATACGCGTAG  818.0 
    134.682         nan 
     65.910         nan 
      2.676         nan 
 
 ACATACGCGTAG  919.0 
    139.092         nan 
     69.980         nan 
      2.691         nan 
 
 ACATACGCGTAG  1020.0 
    148.180         nan 
     73.940         nan 
      2.700         nan 
 
 ACGACTACAGAG  10.0 
      1.980         nan 
      1.740         nan 
      0.346         nan 
 
 ACGACTACAGAG  111.0 
     13.235         nan 
      7.390         nan 
      0.603         nan 
 
 ACGACTACAGAG  212.0 
     19.492         nan 
     10.840         nan 
      0.647         nan 
 
 ACGACTACAGAG  313.0 
     23.328         nan 
     13.190         nan 
      0.648         nan 
 
 ACGACTACAGAG  414.0 
     30.843         nan 
     15.580         nan 
      0.666         nan 
 
 ACGACTACAGAG  515.0 
     32.561         nan 
     17.570         nan 
      0.675         nan 
 
 ACGACTACAGAG  616.0 
     32.885         nan 
     19.620         nan 
      0.690         nan 
 
 ACGACTACAGAG  717.0 
     35.522         nan 
     20.800         nan 
      0.688         nan 
 
 ACGACTACAGAG  818.0 
     34.169         nan 
     22.330         nan 
      0.696         nan 
 
 ACGACTACAGAG  919.0 
     37.696         nan 
     23.700         nan 
      0.696         nan 
 
 ACGACTACAGAG  1020.0 
     38.080         nan 
     24.990         nan 
      0.701         nan 
 
 ACGAGTGCGTAG  10.0 
      8.932         nan 
      5.450         nan 
      2.144         nan 
 
 ACGAGTGCGTAG  111.0 
     38.424         nan 
     20.010         nan 
      3.040         nan 
 
 ACGAGTGCGTAG  212.0 
     53.999         nan 
     28.290         nan 
      3.175         nan 
 
 ACGAGTGCGTAG  313.0 
     60.567         nan 
     34.140         nan 
      3.235         nan 
 
 ACGAGTGCGTAG  414.0 
     70.652         nan 
     39.050         nan 
      3.271         nan 
 
 ACGAGTGCGTAG  515.0 
     74.640         nan 
     43.260         nan 
      3.289         nan 
 
 ACGAGTGCGTAG  616.0 
     77.557         nan 
     46.770         nan 
      3.301         nan 
 
 ACGAGTGCGTAG  717.0 
     87.518         nan 
     50.210         nan 
      3.313         nan 
 
 ACGAGTGCGTAG  818.0 
     86.669         nan 
     53.450         nan 
      3.327         nan 
 
 ACGAGTGCGTAG  919.0 
     89.987         nan 
     56.030         nan 
      3.328         nan 
 
 ACGAGTGCGTAG  1020.0 
     95.425         nan 
     58.990         nan 
      3.341         nan 
 
 ACGCGAGTATAG  10.0 
      9.062         nan 
      4.910         nan 
      1.803         nan 
 
 ACGCGAGTATAG  111.0 
     53.906         nan 
     23.840         nan 
      2.712         nan 
 
 ACGCGAGTATAG  212.0 
     83.449         nan 
     35.270         nan 
      2.860         nan 
 
 ACGCGAGTATAG  313.0 
    101.474         nan 
     44.940         nan 
      2.932         nan 
 
 ACGCGAGTATAG  414.0 
    111.865         nan 
     52.480         nan 
      2.968         nan 
 
 ACGCGAGTATAG  515.0 
    128.644         nan 
     60.030         nan 
      3.007         nan 
 
 ACGCGAGTATAG  616.0 
    134.414         nan 
     65.870         nan 
      3.022         nan 
 
 ACGCGAGTATAG  717.0 
    152.323         nan 
     73.070         nan 
      3.048         nan 
 
 ACGCGAGTATAG  818.0 
    164.916         nan 
     78.480         nan 
      3.064         nan 
 
 ACGCGAGTATAG  919.0 
    182.043         nan 
     84.160         nan 
      3.088         nan 
 
 ACGCGAGTATAG  1020.0 
    200.065         nan 
     88.930         nan 
      3.093         nan 
 
 AGACGCACTCAG  10.0 
     16.875       1.600 
      7.040       0.670 
      2.568       0.243 
 
 AGACGCACTCAG  111.0 
    134.970       0.047 
     41.545       1.415 
      4.154       0.295 
 
 AGACGCACTCAG  212.0 
    187.467       1.157 
     64.785       2.445 
      4.416       0.320 
 
 AGACGCACTCAG  313.0 
    205.231       4.590 
     83.970       3.840 
      4.534       0.337 
 
 AGACGCACTCAG  414.0 
    230.468      10.485 
    101.570       3.630 
      4.637       0.313 
 
 AGACGCACTCAG  515.0 
    241.464       4.458 
    116.040       3.920 
      4.684       0.322 
 
 AGACGCACTCAG  616.0 
    254.296       8.300 
    129.170       4.730 
      4.739       0.331 
 
 AGACGCACTCAG  717.0 
    265.427      11.959 
    142.480       5.240 
      4.787       0.337 
 
 AGACGCACTCAG  818.0 
    276.649      10.066 
    152.890       6.070 
      4.803       0.344 
 
 AGACGCACTCAG  919.0 
    282.791      12.195 
    163.115       5.645 
      4.832       0.337 
 
 AGACGCACTCAG  1020.0 
    294.138      15.346 
    172.780       5.980 
      4.853       0.336 
 
 AGACTATACTAG  10.0 
      8.050         nan 
      5.230         nan 
      2.101         nan 
 
 AGACTATACTAG  111.0 
     56.439         nan 
     22.980         nan 
      3.023         nan 
 
 AGACTATACTAG  212.0 
     75.439         nan 
     35.800         nan 
      3.223         nan 
 
 AGACTATACTAG  313.0 
     83.578         nan 
     43.990         nan 
      3.266         nan 
 
 AGACTATACTAG  414.0 
    100.656         nan 
     51.350         nan 
      3.300         nan 
 
 AGACTATACTAG  515.0 
    116.510         nan 
     57.820         nan 
      3.318         nan 
 
 AGACTATACTAG  616.0 
    132.851         nan 
     63.580         nan 
      3.345         nan 
 
 AGACTATACTAG  717.0 
    136.433         nan 
     69.020         nan 
      3.377         nan 
 
 AGACTATACTAG  818.0 
    143.582         nan 
     74.050         nan 
      3.390         nan 
 
 AGACTATACTAG  919.0 
    150.550         nan 
     78.640         nan 
      3.396         nan 
 
 AGACTATACTAG  1020.0 
    152.723         nan 
     82.910         nan 
      3.408         nan 
 
 AGCACTGTAGAG  10.0 
      2.140         nan 
      1.810         nan 
      0.379         nan 
 
 AGCACTGTAGAG  111.0 
     22.669         nan 
      8.780         nan 
      0.657         nan 
 
 AGCACTGTAGAG  212.0 
     36.394         nan 
     13.140         nan 
      0.645         nan 
 
 AGCACTGTAGAG  313.0 
     41.327         nan 
     17.790         nan 
      0.702         nan 
 
 AGCACTGTAGAG  414.0 
     45.250         nan 
     21.890         nan 
      0.731         nan 
 
 AGCACTGTAGAG  515.0 
     46.259         nan 
     24.490         nan 
      0.728         nan 
 
 AGCACTGTAGAG  616.0 
     47.142         nan 
     27.600         nan 
      0.748         nan 
 
 AGCACTGTAGAG  717.0 
     50.037         nan 
     30.370         nan 
      0.763         nan 
 
 AGCACTGTAGAG  818.0 
     49.868         nan 
     32.610         nan 
      0.763         nan 
 
 AGCACTGTAGAG  919.0 
     50.341         nan 
     34.460         nan 
      0.769         nan 
 
 AGCACTGTAGAG  1020.0 
     51.062         nan 
     35.950         nan 
      0.769         nan 
 
 AGCGTCGTCTAG  10.0 
      8.508         nan 
      4.900         nan 
      1.892         nan 
 
 AGCGTCGTCTAG  111.0 
     40.724         nan 
     20.990         nan 
      2.697         nan 
 
 AGCGTCGTCTAG  212.0 
     65.028         nan 
     30.940         nan 
      2.856         nan 
 
 AGCGTCGTCTAG  313.0 
     70.933         nan 
     37.000         nan 
      2.891         nan 
 
 AGCGTCGTCTAG  414.0 
     84.148         nan 
     43.620         nan 
      2.926         nan 
 
 AGCGTCGTCTAG  515.0 
     97.748         nan 
     48.320         nan 
      2.949         nan 
 
 AGCGTCGTCTAG  616.0 
    103.428         nan 
     53.100         nan 
      2.966         nan 
 
 AGCGTCGTCTAG  717.0 
    106.703         nan 
     57.520         nan 
      2.984         nan 
 
 AGCGTCGTCTAG  818.0 
    120.400         nan 
     61.700         nan 
      2.999         nan 
 
 AGCGTCGTCTAG  919.0 
    125.316         nan 
     65.540         nan 
      3.003         nan 
 
 AGCGTCGTCTAG  1020.0 
    128.304         nan 
     68.920         nan 
      3.013         nan 
 
 AGCTCACGTAAG  10.0 
      9.227       4.062 
      4.785       1.385 
      1.735       0.560 
 
 AGCTCACGTAAG  111.0 
     64.347      24.567 
     24.325       9.155 
      2.670       0.933 
 
 AGCTCACGTAAG  212.0 
     78.366      23.139 
     36.585      13.285 
      2.799       0.983 
 
 AGCTCACGTAAG  313.0 
     95.417      34.216 
     46.670      16.490 
      2.888       1.003 
 
 AGCTCACGTAAG  414.0 
    102.638      39.263 
     54.600      18.600 
      2.945       1.008 
 
 AGCTCACGTAAG  515.0 
    112.910      47.077 
     61.570      21.370 
      2.965       1.022 
 
 AGCTCACGTAAG  616.0 
    119.944      54.090 
     67.030      23.110 
      2.983       1.026 
 
 AGCTCACGTAAG  717.0 
    132.646      64.014 
     73.355      26.115 
      3.012       1.045 
 
 AGCTCACGTAAG  818.0 
    139.209      71.344 
     77.820      27.660 
      3.018       1.042 
 
 AGCTCACGTAAG  919.0 
    143.736      77.391 
     82.765      29.905 
      3.034       1.052 
 
 AGCTCACGTAAG  1020.0 
    147.650      82.021 
     86.895      31.915 
      3.043       1.055 
 
 ATACGACGTAAG  10.0 
      6.550         nan 
      4.380         nan 
      1.769         nan 
 
 ATACGACGTAAG  111.0 
     46.050         nan 
     19.620         nan 
      2.587         nan 
 
 ATACGACGTAAG  212.0 
     70.754         nan 
     28.300         nan 
      2.666         nan 
 
 ATACGACGTAAG  313.0 
     87.388         nan 
     35.540         nan 
      2.733         nan 
 
 ATACGACGTAAG  414.0 
     99.452         nan 
     42.440         nan 
      2.774         nan 
 
 ATACGACGTAAG  515.0 
    107.519         nan 
     49.130         nan 
      2.804         nan 
 
 ATACGACGTAAG  616.0 
    111.082         nan 
     54.150         nan 
      2.812         nan 
 
 ATACGACGTAAG  717.0 
    107.190         nan 
     58.810         nan 
      2.825         nan 
 
 ATACGACGTAAG  818.0 
    103.966         nan 
     63.060         nan 
      2.841         nan 
 
 ATACGACGTAAG  919.0 
    101.009         nan 
     67.090         nan 
      2.847         nan 
 
 ATACGACGTAAG  1020.0 
    100.734         nan 
     70.940         nan 
      2.856         nan 
 
 ATAGAGTACTAG  10.0 
     13.557         nan 
      6.380         nan 
      2.402         nan 
 
 ATAGAGTACTAG  111.0 
    107.452         nan 
     34.990         nan 
      3.780         nan 
 
 ATAGAGTACTAG  212.0 
    138.971         nan 
     54.000         nan 
      3.968         nan 
 
 ATAGAGTACTAG  313.0 
    171.448         nan 
     71.360         nan 
      4.108         nan 
 
 ATAGAGTACTAG  414.0 
    183.803         nan 
     83.630         nan 
      4.162         nan 
 
 ATAGAGTACTAG  515.0 
    202.388         nan 
     95.410         nan 
      4.216         nan 
 
 ATAGAGTACTAG  616.0 
    208.528         nan 
    105.410         nan 
      4.241         nan 
 
 ATAGAGTACTAG  717.0 
    223.689         nan 
    115.780         nan 
      4.279         nan 
 
 ATAGAGTACTAG  818.0 
    235.559         nan 
    124.990         nan 
      4.313         nan 
 
 ATAGAGTACTAG  919.0 
    251.040         nan 
    133.530         nan 
      4.324         nan 
 
 ATAGAGTACTAG  1020.0 
    254.594         nan 
    140.830         nan 
      4.343         nan 
 
 ATATCGCGAGAG  10.0 
     14.378         nan 
      6.570         nan 
      2.448         nan 
 
 ATATCGCGAGAG  111.0 
    107.749         nan 
     35.320         nan 
      3.868         nan 
 
 ATATCGCGAGAG  212.0 
    152.383         nan 
     55.540         nan 
      4.125         nan 
 
 ATATCGCGAGAG  313.0 
    189.175         nan 
     70.740         nan 
      4.196         nan 
 
 ATATCGCGAGAG  414.0 
    213.498         nan 
     85.970         nan 
      4.287         nan 
 
 ATATCGCGAGAG  515.0 
    214.121         nan 
     99.370         nan 
      4.357         nan 
 
 ATATCGCGAGAG  616.0 
    227.671         nan 
    110.260         nan 
      4.381         nan 
 
 ATATCGCGAGAG  717.0 
    229.488         nan 
    121.280         nan 
      4.420         nan 
 
 ATATCGCGAGAG  818.0 
    236.445         nan 
    130.410         nan 
      4.437         nan 
 
 ATATCGCGAGAG  919.0 
    235.584         nan 
    139.610         nan 
      4.461         nan 
 
 ATATCGCGAGAG  1020.0 
    241.387         nan 
    147.840         nan 
      4.482         nan 
 
 ATCAGACACGAG  10.0 
     13.500         nan 
      6.380         nan 
      2.376         nan 
 
 ATCAGACACGAG  111.0 
    113.583         nan 
     35.900         nan 
      3.832         nan 
 
 ATCAGACACGAG  212.0 
    151.590         nan 
     54.640         nan 
      4.032         nan 
 
 ATCAGACACGAG  313.0 
    195.678         nan 
     72.730         nan 
      4.176         nan 
 
 ATCAGACACGAG  414.0 
    226.829         nan 
     87.780         nan 
      4.258         nan 
 
 ATCAGACACGAG  515.0 
    248.135         nan 
    102.090         nan 
      4.326         nan 
 
 ATCAGACACGAG  616.0 
    253.505         nan 
    113.340         nan 
      4.352         nan 
 
 ATCAGACACGAG  717.0 
    260.694         nan 
    124.080         nan 
      4.373         nan 
 
 ATCAGACACGAG  818.0 
    280.385         nan 
    135.170         nan 
      4.409         nan 
 
 ATCAGACACGAG  919.0 
    278.097         nan 
    144.800         nan 
      4.433         nan 
 
 ATCAGACACGAG  1020.0 
    280.351         nan 
    153.820         nan 
      4.450         nan 
 
 CACGCTACGTAG  10.0 
     11.012         nan 
      6.120         nan 
      2.367         nan 
 
 CACGCTACGTAG  111.0 
     97.595         nan 
     28.710         nan 
      3.463         nan 
 
 CACGCTACGTAG  212.0 
    122.103         nan 
     44.880         nan 
      3.644         nan 
 
 CACGCTACGTAG  313.0 
    142.655         nan 
     59.010         nan 
      3.747         nan 
 
 CACGCTACGTAG  414.0 
    157.261         nan 
     70.520         nan 
      3.802         nan 
 
 CACGCTACGTAG  515.0 
    166.979         nan 
     80.930         nan 
      3.838         nan 
 
 CACGCTACGTAG  616.0 
    175.510         nan 
     90.330         nan 
      3.875         nan 
 
 CACGCTACGTAG  717.0 
    183.228         nan 
     99.310         nan 
      3.907         nan 
 
 CACGCTACGTAG  818.0 
    191.016         nan 
    107.860         nan 
      3.938         nan 
 
 CACGCTACGTAG  919.0 
    188.544         nan 
    114.160         nan 
      3.944         nan 
 
 CACGCTACGTAG  1020.0 
    190.769         nan 
    120.830         nan 
      3.962         nan 
 
 CAGTAGACGTAG  10.0 
      5.810         nan 
      3.820         nan 
      1.425         nan 
 
 CAGTAGACGTAG  111.0 
     39.924         nan 
     18.090         nan 
      2.080         nan 
 
 CAGTAGACGTAG  212.0 
     59.661         nan 
     27.510         nan 
      2.213         nan 
 
 CAGTAGACGTAG  313.0 
     73.312         nan 
     33.950         nan 
      2.261         nan 
 
 CAGTAGACGTAG  414.0 
     94.949         nan 
     39.990         nan 
      2.295         nan 
 
 CAGTAGACGTAG  515.0 
    102.657         nan 
     44.870         nan 
      2.318         nan 
 
 CAGTAGACGTAG  616.0 
    105.447         nan 
     49.750         nan 
      2.337         nan 
 
 CAGTAGACGTAG  717.0 
    109.861         nan 
     53.860         nan 
      2.340         nan 
 
 CAGTAGACGTAG  818.0 
    114.001         nan 
     58.380         nan 
      2.355         nan 
 
 CAGTAGACGTAG  919.0 
    117.204         nan 
     62.000         nan 
      2.362         nan 
 
 CAGTAGACGTAG  1020.0 
    123.416         nan 
     65.960         nan 
      2.373         nan 
 
 CATAGTAGTGAG  10.0 
      5.467         nan 
      3.790         nan 
      1.450         nan 
 
 CATAGTAGTGAG  111.0 
     31.941         nan 
     15.530         nan 
      2.069         nan 
 
 CATAGTAGTGAG  212.0 
     42.161         nan 
     21.830         nan 
      2.115         nan 
 
 CATAGTAGTGAG  313.0 
     52.391         nan 
     27.610         nan 
      2.161         nan 
 
 CATAGTAGTGAG  414.0 
     59.756         nan 
     31.600         nan 
      2.161         nan 
 
 CATAGTAGTGAG  515.0 
     60.125         nan 
     35.440         nan 
      2.195         nan 
 
 CATAGTAGTGAG  616.0 
     63.686         nan 
     38.760         nan 
      2.205         nan 
 
 CATAGTAGTGAG  717.0 
     67.033         nan 
     41.790         nan 
      2.225         nan 
 
 CATAGTAGTGAG  818.0 
     67.816         nan 
     44.600         nan 
      2.235         nan 
 
 CATAGTAGTGAG  919.0 
     68.491         nan 
     46.650         nan 
      2.240         nan 
 
 CATAGTAGTGAG  1020.0 
     69.985         nan 
     48.980         nan 
      2.244         nan 
 
 CGACGTGACTAG  10.0 
      5.570         nan 
      4.080         nan 
      1.671         nan 
 
 CGACGTGACTAG  111.0 
     34.220         nan 
     15.200         nan 
      2.208         nan 
 
 CGACGTGACTAG  212.0 
     53.576         nan 
     23.580         nan 
      2.354         nan 
 
 CGACGTGACTAG  313.0 
     61.141         nan 
     28.450         nan 
      2.355         nan 
 
 CGACGTGACTAG  414.0 
     71.864         nan 
     34.580         nan 
      2.402         nan 
 
 CGACGTGACTAG  515.0 
     78.584         nan 
     39.420         nan 
      2.417         nan 
 
 CGACGTGACTAG  616.0 
     78.673         nan 
     43.780         nan 
      2.437         nan 
 
 CGACGTGACTAG  717.0 
     80.728         nan 
     47.170         nan 
      2.440         nan 
 
 CGACGTGACTAG  818.0 
     86.383         nan 
     50.840         nan 
      2.457         nan 
 
 CGACGTGACTAG  919.0 
     86.392         nan 
     53.880         nan 
      2.463         nan 
 
 CGACGTGACTAG  1020.0 
     88.325         nan 
     56.950         nan 
      2.473         nan 
 
 CGAGAGATACAG  10.0 
      8.358         nan 
      4.910         nan 
      1.897         nan 
 
 CGAGAGATACAG  111.0 
     55.237         nan 
     22.330         nan 
      2.740         nan 
 
 CGAGAGATACAG  212.0 
     68.257         nan 
     33.670         nan 
      2.888         nan 
 
 CGAGAGATACAG  313.0 
     75.808         nan 
     42.020         nan 
      2.943         nan 
 
 CGAGAGATACAG  414.0 
     85.018         nan 
     48.580         nan 
      2.973         nan 
 
 CGAGAGATACAG  515.0 
     94.497         nan 
     54.580         nan 
      2.993         nan 
 
 CGAGAGATACAG  616.0 
    106.808         nan 
     60.420         nan 
      3.030         nan 
 
 CGAGAGATACAG  717.0 
    114.999         nan 
     65.130         nan 
      3.045         nan 
 
 CGAGAGATACAG  818.0 
    119.053         nan 
     68.960         nan 
      3.054         nan 
 
 CGAGAGATACAG  919.0 
    131.723         nan 
     73.180         nan 
      3.064         nan 
 
 CGAGAGATACAG  1020.0 
    142.857         nan 
     76.930         nan 
      3.079         nan 
 
 CGTAGACTAGAG  10.0 
      9.133         nan 
      4.830         nan 
      1.796         nan 
 
 CGTAGACTAGAG  111.0 
     69.459         nan 
     25.430         nan 
      2.633         nan 
 
 CGTAGACTAGAG  212.0 
    103.522         nan 
     40.860         nan 
      2.840         nan 
 
 CGTAGACTAGAG  313.0 
    119.800         nan 
     51.670         nan 
      2.871         nan 
 
 CGTAGACTAGAG  414.0 
    129.456         nan 
     61.850         nan 
      2.915         nan 
 
 CGTAGACTAGAG  515.0 
    130.565         nan 
     70.180         nan 
      2.964         nan 
 
 CGTAGACTAGAG  616.0 
    143.092         nan 
     77.940         nan 
      2.983         nan 
 
 CGTAGACTAGAG  717.0 
    150.467         nan 
     84.590         nan 
      3.002         nan 
 
 CGTAGACTAGAG  818.0 
    157.851         nan 
     91.100         nan 
      3.024         nan 
 
 CGTAGACTAGAG  919.0 
    160.994         nan 
     96.570         nan 
      3.037         nan 
 
 CGTAGACTAGAG  1020.0 
    165.686         nan 
    101.940         nan 
      3.052         nan 
 
 CGTCTAGTACAG  10.0 
      4.285         nan 
      2.950         nan 
      0.922         nan 
 
 CGTCTAGTACAG  111.0 
     34.790         nan 
     14.980         nan 
      1.349         nan 
 
 CGTCTAGTACAG  212.0 
     53.743         nan 
     23.200         nan 
      1.433         nan 
 
 CGTCTAGTACAG  313.0 
     70.492         nan 
     30.240         nan 
      1.503         nan 
 
 CGTCTAGTACAG  414.0 
     78.128         nan 
     36.310         nan 
      1.547         nan 
 
 CGTCTAGTACAG  515.0 
     92.622         nan 
     40.580         nan 
      1.545         nan 
 
 CGTCTAGTACAG  616.0 
    100.248         nan 
     44.500         nan 
      1.542         nan 
 
 CGTCTAGTACAG  717.0 
    104.564         nan 
     49.400         nan 
      1.571         nan 
 
 CGTCTAGTACAG  818.0 
    103.588         nan 
     53.350         nan 
      1.584         nan 
 
 CGTCTAGTACAG  919.0 
    103.897         nan 
     56.620         nan 
      1.586         nan 
 
 CGTCTAGTACAG  1020.0 
    100.680         nan 
     59.930         nan 
      1.594         nan 
 
 CTCGCGTGTCAG  10.0 
     13.723         nan 
      5.870         nan 
      2.147         nan 
 
 CTCGCGTGTCAG  111.0 
     90.891         nan 
     33.470         nan 
      3.388         nan 
 
 CTCGCGTGTCAG  212.0 
    140.029         nan 
     51.450         nan 
      3.581         nan 
 
 CTCGCGTGTCAG  313.0 
    170.023         nan 
     65.400         nan 
      3.672         nan 
 
 CTCGCGTGTCAG  414.0 
    187.999         nan 
     78.780         nan 
      3.749         nan 
 
 CTCGCGTGTCAG  515.0 
    211.538         nan 
     90.470         nan 
      3.805         nan 
 
 CTCGCGTGTCAG  616.0 
    227.754         nan 
    100.900         nan 
      3.852         nan 
 
 CTCGCGTGTCAG  717.0 
    247.142         nan 
    110.650         nan 
      3.880         nan 
 
 CTCGCGTGTCAG  818.0 
    270.222         nan 
    119.610         nan 
      3.900         nan 
 
 CTCGCGTGTCAG  919.0 
    282.354         nan 
    129.070         nan 
      3.931         nan 
 
 CTCGCGTGTCAG  1020.0 
    298.684         nan 
    136.850         nan 
      3.941         nan 
 
 TACACACACTAG  10.0 
     12.245         nan 
      6.250         nan 
      2.388         nan 
 
 TACACACACTAG  111.0 
    110.047         nan 
     34.790         nan 
      3.668         nan 
 
 TACACACACTAG  212.0 
    139.497         nan 
     55.290         nan 
      3.920         nan 
 
 TACACACACTAG  313.0 
    161.900         nan 
     71.960         nan 
      4.045         nan 
 
 TACACACACTAG  414.0 
    176.660         nan 
     84.690         nan 
      4.092         nan 
 
 TACACACACTAG  515.0 
    197.600         nan 
     97.130         nan 
      4.157         nan 
 
 TACACACACTAG  616.0 
    214.821         nan 
    108.060         nan 
      4.185         nan 
 
 TACACACACTAG  717.0 
    231.937         nan 
    118.010         nan 
      4.220         nan 
 
 TACACACACTAG  818.0 
    232.853         nan 
    126.820         nan 
      4.248         nan 
 
 TACACACACTAG  919.0 
    245.406         nan 
    135.130         nan 
      4.268         nan 
 
 TACACACACTAG  1020.0 
    252.232         nan 
    142.830         nan 
      4.283         nan 
 
 TACACGTGATAG  10.0 
      6.345         nan 
      4.010         nan 
      1.483         nan 
 
 TACACGTGATAG  111.0 
     29.746         nan 
     16.160         nan 
      2.160         nan 
 
 TACACGTGATAG  212.0 
     44.191         nan 
     22.160         nan 
      2.237         nan 
 
 TACACGTGATAG  313.0 
     55.093         nan 
     26.730         nan 
      2.279         nan 
 
 TACACGTGATAG  414.0 
     62.980         nan 
     30.860         nan 
      2.294         nan 
 
 TACACGTGATAG  515.0 
     70.077         nan 
     34.820         nan 
      2.325         nan 
 
 TACACGTGATAG  616.0 
     80.735         nan 
     38.650         nan 
      2.340         nan 
 
 TACACGTGATAG  717.0 
     87.611         nan 
     41.460         nan 
      2.342         nan 
 
 TACACGTGATAG  818.0 
     88.859         nan 
     44.430         nan 
      2.353         nan 
 
 TACACGTGATAG  919.0 
     87.912         nan 
     47.280         nan 
      2.362         nan 
 
 TACACGTGATAG  1020.0 
     91.072         nan 
     49.970         nan 
      2.368         nan 
 
 TACAGATCGTAG  10.0 
      5.783         nan 
      4.140         nan 
      1.692         nan 
 
 TACAGATCGTAG  111.0 
     54.538         nan 
     18.250         nan 
      2.359         nan 
 
 TACAGATCGTAG  212.0 
     70.568         nan 
     27.490         nan 
      2.454         nan 
 
 TACAGATCGTAG  313.0 
     84.073         nan 
     36.270         nan 
      2.513         nan 
 
 TACAGATCGTAG  414.0 
     85.183         nan 
     43.350         nan 
      2.565         nan 
 
 TACAGATCGTAG  515.0 
     89.642         nan 
     49.660         nan 
      2.590         nan 
 
 TACAGATCGTAG  616.0 
     86.395         nan 
     54.560         nan 
      2.608         nan 
 
 TACAGATCGTAG  717.0 
     90.032         nan 
     59.300         nan 
      2.631         nan 
 
 TACAGATCGTAG  818.0 
     91.484         nan 
     63.440         nan 
      2.638         nan 
 
 TACAGATCGTAG  919.0 
     90.262         nan 
     66.900         nan 
      2.647         nan 
 
 TACAGATCGTAG  1020.0 
     90.407         nan 
     69.960         nan 
      2.655         nan 
 
 TACGCTGTCTAG  10.0 
      7.863         nan 
      4.430         nan 
      1.670         nan 
 
 TACGCTGTCTAG  111.0 
     57.776         nan 
     23.600         nan 
      2.653         nan 
 
 TACGCTGTCTAG  212.0 
     76.485         nan 
     36.060         nan 
      2.778         nan 
 
 TACGCTGTCTAG  313.0 
     86.717         nan 
     45.550         nan 
      2.887         nan 
 
 TACGCTGTCTAG  414.0 
    106.526         nan 
     54.200         nan 
      2.921         nan 
 
 TACGCTGTCTAG  515.0 
    119.204         nan 
     60.220         nan 
      2.949         nan 
 
 TACGCTGTCTAG  616.0 
    135.464         nan 
     66.670         nan 
      2.971         nan 
 
 TACGCTGTCTAG  717.0 
    142.388         nan 
     71.850         nan 
      2.971         nan 
 
 TACGCTGTCTAG  818.0 
    151.896         nan 
     77.000         nan 
      2.993         nan 
 
 TACGCTGTCTAG  919.0 
    161.466         nan 
     82.010         nan 
      3.007         nan 
 
 TACGCTGTCTAG  1020.0 
    173.352         nan 
     86.940         nan 
      3.019         nan 
 
 TAGAGACGAGAG  10.0 
      2.220         nan 
      1.870         nan 
      0.405         nan 
 
 TAGAGACGAGAG  111.0 
     20.246         nan 
      8.420         nan 
      0.639         nan 
 
 TAGAGACGAGAG  212.0 
     33.517         nan 
     13.590         nan 
      0.706         nan 
 
 TAGAGACGAGAG  313.0 
     39.976         nan 
     17.790         nan 
      0.737         nan 
 
 TAGAGACGAGAG  414.0 
     43.512         nan 
     21.340         nan 
      0.765         nan 
 
 TAGAGACGAGAG  515.0 
     51.468         nan 
     24.860         nan 
      0.785         nan 
 
 TAGAGACGAGAG  616.0 
     51.627         nan 
     26.810         nan 
      0.785         nan 
 
 TAGAGACGAGAG  717.0 
     53.828         nan 
     29.730         nan 
      0.794         nan 
 
 TAGAGACGAGAG  818.0 
     57.726         nan 
     32.080         nan 
      0.800         nan 
 
 TAGAGACGAGAG  919.0 
     55.957         nan 
     34.080         nan 
      0.804         nan 
 
 TAGAGACGAGAG  1020.0 
     57.508         nan 
     35.970         nan 
      0.808         nan 
 
 TAGTGTAGATAG  10.0 
      9.718         nan 
      5.080         nan 
      1.899         nan 
 
 TAGTGTAGATAG  111.0 
     77.801         nan 
     25.970         nan 
      2.882         nan 
 
 TAGTGTAGATAG  212.0 
    110.192         nan 
     40.780         nan 
      3.075         nan 
 
 TAGTGTAGATAG  313.0 
    127.549         nan 
     52.330         nan 
      3.125         nan 
 
 TAGTGTAGATAG  414.0 
    145.060         nan 
     63.030         nan 
      3.202         nan 
 
 TAGTGTAGATAG  515.0 
    158.675         nan 
     72.810         nan 
      3.242         nan 
 
 TAGTGTAGATAG  616.0 
    172.471         nan 
     81.010         nan 
      3.264         nan 
 
 TAGTGTAGATAG  717.0 
    180.183         nan 
     88.820         nan 
      3.290         nan 
 
 TAGTGTAGATAG  818.0 
    187.330         nan 
     95.820         nan 
      3.306         nan 
 
 TAGTGTAGATAG  919.0 
    194.999         nan 
    102.950         nan 
      3.329         nan 
 
 TAGTGTAGATAG  1020.0 
    205.074         nan 
    108.830         nan 
      3.338         nan 
 
 TCGATCACGTAG  10.0 
      5.583         nan 
      3.460         nan 
      1.154         nan 
 
 TCGATCACGTAG  111.0 
     39.535         nan 
     17.870         nan 
      1.808         nan 
 
 TCGATCACGTAG  212.0 
     52.523         nan 
     25.890         nan 
      1.908         nan 
 
 TCGATCACGTAG  313.0 
     65.529         nan 
     32.670         nan 
      1.940         nan 
 
 TCGATCACGTAG  414.0 
     82.514         nan 
     39.060         nan 
      2.008         nan 
 
 TCGATCACGTAG  515.0 
     88.046         nan 
     43.790         nan 
      2.025         nan 
 
 TCGATCACGTAG  616.0 
     91.828         nan 
     48.330         nan 
      2.038         nan 
 
 TCGATCACGTAG  717.0 
     99.071         nan 
     52.280         nan 
      2.046         nan 
 
 TCGATCACGTAG  818.0 
    104.453         nan 
     56.010         nan 
      2.055         nan 
 
 TCGATCACGTAG  919.0 
    113.388         nan 
     59.740         nan 
      2.066         nan 
 
 TCGATCACGTAG  1020.0 
    121.633         nan 
     62.930         nan 
      2.070         nan 
 
 TCTACGTAGCAG  10.0 
      4.469       0.164 
      3.290       0.340 
      1.214       0.299 
 
 TCTACGTAGCAG  111.0 
     28.380       0.978 
     13.165       0.425 
      1.628       0.345 
 
 TCTACGTAGCAG  212.0 
     42.562       4.150 
     19.300       0.440 
      1.713       0.338 
 
 TCTACGTAGCAG  313.0 
     51.391       2.145 
     24.410       0.450 
      1.753       0.342 
 
 TCTACGTAGCAG  414.0 
     54.974       0.607 
     28.165       0.655 
      1.768       0.336 
 
 TCTACGTAGCAG  515.0 
     60.332       3.583 
     31.955       0.595 
      1.789       0.332 
 
 TCTACGTAGCAG  616.0 
     63.084       4.010 
     34.765       0.705 
      1.793       0.339 
 
 TCTACGTAGCAG  717.0 
     69.037       2.674 
     38.040       0.720 
      1.814       0.334 
 
 TCTACGTAGCAG  818.0 
     73.341       4.948 
     40.750       0.580 
      1.818       0.337 
 
 TCTACGTAGCAG  919.0 
     79.277       5.793 
     43.265       0.605 
      1.824       0.341 
 
 TCTACGTAGCAG  1020.0 
     81.785       2.832 
     45.470       0.500 
      1.828       0.339 
 
 TGATACGTCTAG  10.0 
      5.957         nan 
      3.920         nan 
      1.456         nan 
 
 TGATACGTCTAG  111.0 
     34.947         nan 
     15.230         nan 
      2.029         nan 
 
 TGATACGTCTAG  212.0 
     43.986         nan 
     22.460         nan 
      2.137         nan 
 
 TGATACGTCTAG  313.0 
     57.696         nan 
     28.260         nan 
      2.181         nan 
 
 TGATACGTCTAG  414.0 
     73.314         nan 
     33.200         nan 
      2.227         nan 
 
 TGATACGTCTAG  515.0 
     73.949         nan 
     36.890         nan 
      2.228         nan 
 
 TGATACGTCTAG  616.0 
     90.677         nan 
     40.940         nan 
      2.245         nan 
 
 TGATACGTCTAG  717.0 
     91.648         nan 
     44.310         nan 
      2.251         nan 
 
 TGATACGTCTAG  818.0 
     93.969         nan 
     47.660         nan 
      2.257         nan 
 
 TGATACGTCTAG  919.0 
     97.062         nan 
     50.940         nan 
      2.272         nan 
 
 TGATACGTCTAG  1020.0 
     94.643         nan 
     53.950         nan 
      2.278         nan 
 
 TGTACTACTCAG  10.0 
      5.965         nan 
      3.580         nan 
      1.212         nan 
 
 TGTACTACTCAG  111.0 
     42.434         nan 
     18.610         nan 
      1.994         nan 
 
 TGTACTACTCAG  212.0 
     53.681         nan 
     26.370         nan 
      2.125         nan 
 
 TGTACTACTCAG  313.0 
     65.351         nan 
     32.040         nan 
      2.141         nan 
 
 TGTACTACTCAG  414.0 
     67.423         nan 
     36.510         nan 
      2.169         nan 
 
 TGTACTACTCAG  515.0 
     77.934         nan 
     41.880         nan 
      2.196         nan 
 
 TGTACTACTCAG  616.0 
     83.123         nan 
     45.350         nan 
      2.214         nan 
 
 TGTACTACTCAG  717.0 
     93.775         nan 
     48.980         nan 
      2.211         nan 
 
 TGTACTACTCAG  818.0 
    100.879         nan 
     52.730         nan 
      2.235         nan 
 
 TGTACTACTCAG  919.0 
    104.109         nan 
     55.870         nan 
      2.243         nan 
 
 TGTACTACTCAG  1020.0 
    113.083         nan 
     58.950         nan 
      2.246         nan 
 
  SampleID  Seqs/Sample 
 chao1 Ave.  chao1 Err. 
 observed_species Ave.  observed_species Err. 
 shannon Ave.  shannon Err. 
 
 
 BW.01.1  10.0 
      8.932         nan 
      5.450         nan 
      2.144         nan 
 
 BW.01.1  111.0 
     38.424         nan 
     20.010         nan 
      3.040         nan 
 
 BW.01.1  212.0 
     53.999         nan 
     28.290         nan 
      3.175         nan 
 
 BW.01.1  313.0 
     60.567         nan 
     34.140         nan 
      3.235         nan 
 
 BW.01.1  414.0 
     70.652         nan 
     39.050         nan 
      3.271         nan 
 
 BW.01.1  515.0 
     74.640         nan 
     43.260         nan 
      3.289         nan 
 
 BW.01.1  616.0 
     77.557         nan 
     46.770         nan 
      3.301         nan 
 
 BW.01.1  717.0 
     87.518         nan 
     50.210         nan 
      3.313         nan 
 
 BW.01.1  818.0 
     86.669         nan 
     53.450         nan 
      3.327         nan 
 
 BW.01.1  919.0 
     89.987         nan 
     56.030         nan 
      3.328         nan 
 
 BW.01.1  1020.0 
     95.425         nan 
     58.990         nan 
      3.341         nan 
 
 BW.01.3  10.0 
     18.475         nan 
      7.710         nan 
      2.811         nan 
 
 BW.01.3  111.0 
    135.017         nan 
     42.960         nan 
      4.448         nan 
 
 BW.01.3  212.0 
    186.310         nan 
     67.230         nan 
      4.736         nan 
 
 BW.01.3  313.0 
    209.821         nan 
     87.810         nan 
      4.870         nan 
 
 BW.01.3  414.0 
    240.953         nan 
    105.200         nan 
      4.950         nan 
 
 BW.01.3  515.0 
    245.922         nan 
    119.960         nan 
      5.006         nan 
 
 BW.01.3  616.0 
    262.596         nan 
    133.900         nan 
      5.070         nan 
 
 BW.01.3  717.0 
    277.386         nan 
    147.720         nan 
      5.124         nan 
 
 BW.01.3  818.0 
    286.716         nan 
    158.960         nan 
      5.148         nan 
 
 BW.01.3  919.0 
    294.986         nan 
    168.760         nan 
      5.169         nan 
 
 BW.01.3  1020.0 
    309.484         nan 
    178.760         nan 
      5.189         nan 
 
 BW.01.4  10.0 
      2.140         nan 
      1.810         nan 
      0.379         nan 
 
 BW.01.4  111.0 
     22.669         nan 
      8.780         nan 
      0.657         nan 
 
 BW.01.4  212.0 
     36.394         nan 
     13.140         nan 
      0.645         nan 
 
 BW.01.4  313.0 
     41.327         nan 
     17.790         nan 
      0.702         nan 
 
 BW.01.4  414.0 
     45.250         nan 
     21.890         nan 
      0.731         nan 
 
 BW.01.4  515.0 
     46.259         nan 
     24.490         nan 
      0.728         nan 
 
 BW.01.4  616.0 
     47.142         nan 
     27.600         nan 
      0.748         nan 
 
 BW.01.4  717.0 
     50.037         nan 
     30.370         nan 
      0.763         nan 
 
 BW.01.4  818.0 
     49.868         nan 
     32.610         nan 
      0.763         nan 
 
 BW.01.4  919.0 
     50.341         nan 
     34.460         nan 
      0.769         nan 
 
 BW.01.4  1020.0 
     51.062         nan 
     35.950         nan 
      0.769         nan 
 
 BW.02.1  10.0 
      5.957         nan 
      3.920         nan 
      1.456         nan 
 
 BW.02.1  111.0 
     34.947         nan 
     15.230         nan 
      2.029         nan 
 
 BW.02.1  212.0 
     43.986         nan 
     22.460         nan 
      2.137         nan 
 
 BW.02.1  313.0 
     57.696         nan 
     28.260         nan 
      2.181         nan 
 
 BW.02.1  414.0 
     73.314         nan 
     33.200         nan 
      2.227         nan 
 
 BW.02.1  515.0 
     73.949         nan 
     36.890         nan 
      2.228         nan 
 
 BW.02.1  616.0 
     90.677         nan 
     40.940         nan 
      2.245         nan 
 
 BW.02.1  717.0 
     91.648         nan 
     44.310         nan 
      2.251         nan 
 
 BW.02.1  818.0 
     93.969         nan 
     47.660         nan 
      2.257         nan 
 
 BW.02.1  919.0 
     97.062         nan 
     50.940         nan 
      2.272         nan 
 
 BW.02.1  1020.0 
     94.643         nan 
     53.950         nan 
      2.278         nan 
 
 BW.02.10  10.0 
      9.133         nan 
      4.830         nan 
      1.796         nan 
 
 BW.02.10  111.0 
     69.459         nan 
     25.430         nan 
      2.633         nan 
 
 BW.02.10  212.0 
    103.522         nan 
     40.860         nan 
      2.840         nan 
 
 BW.02.10  313.0 
    119.800         nan 
     51.670         nan 
      2.871         nan 
 
 BW.02.10  414.0 
    129.456         nan 
     61.850         nan 
      2.915         nan 
 
 BW.02.10  515.0 
    130.565         nan 
     70.180         nan 
      2.964         nan 
 
 BW.02.10  616.0 
    143.092         nan 
     77.940         nan 
      2.983         nan 
 
 BW.02.10  717.0 
    150.467         nan 
     84.590         nan 
      3.002         nan 
 
 BW.02.10  818.0 
    157.851         nan 
     91.100         nan 
      3.024         nan 
 
 BW.02.10  919.0 
    160.994         nan 
     96.570         nan 
      3.037         nan 
 
 BW.02.10  1020.0 
    165.686         nan 
    101.940         nan 
      3.052         nan 
 
 BW.02.6  10.0 
      4.285         nan 
      2.950         nan 
      0.922         nan 
 
 BW.02.6  111.0 
     34.790         nan 
     14.980         nan 
      1.349         nan 
 
 BW.02.6  212.0 
     53.743         nan 
     23.200         nan 
      1.433         nan 
 
 BW.02.6  313.0 
     70.492         nan 
     30.240         nan 
      1.503         nan 
 
 BW.02.6  414.0 
     78.128         nan 
     36.310         nan 
      1.547         nan 
 
 BW.02.6  515.0 
     92.622         nan 
     40.580         nan 
      1.545         nan 
 
 BW.02.6  616.0 
    100.248         nan 
     44.500         nan 
      1.542         nan 
 
 BW.02.6  717.0 
    104.564         nan 
     49.400         nan 
      1.571         nan 
 
 BW.02.6  818.0 
    103.588         nan 
     53.350         nan 
      1.584         nan 
 
 BW.02.6  919.0 
    103.897         nan 
     56.620         nan 
      1.586         nan 
 
 BW.02.6  1020.0 
    100.680         nan 
     59.930         nan 
      1.594         nan 
 
 BW.02.7  10.0 
      4.305         nan 
      2.950         nan 
      0.915         nan 
 
 BW.02.7  111.0 
     27.402         nan 
     12.740         nan 
      1.283         nan 
 
 BW.02.7  212.0 
     38.412         nan 
     18.860         nan 
      1.374         nan 
 
 BW.02.7  313.0 
     49.246         nan 
     23.960         nan 
      1.411         nan 
 
 BW.02.7  414.0 
     55.581         nan 
     27.510         nan 
      1.432         nan 
 
 BW.02.7  515.0 
     63.915         nan 
     31.360         nan 
      1.458         nan 
 
 BW.02.7  616.0 
     67.094         nan 
     34.060         nan 
      1.454         nan 
 
 BW.02.7  717.0 
     71.711         nan 
     37.320         nan 
      1.481         nan 
 
 BW.02.7  818.0 
     78.289         nan 
     40.170         nan 
      1.481         nan 
 
 BW.02.7  919.0 
     85.069         nan 
     42.660         nan 
      1.483         nan 
 
 BW.02.7  1020.0 
     84.617         nan 
     44.970         nan 
      1.489         nan 
 
 BW.02.8  10.0 
      5.965         nan 
      3.580         nan 
      1.212         nan 
 
 BW.02.8  111.0 
     42.434         nan 
     18.610         nan 
      1.994         nan 
 
 BW.02.8  212.0 
     53.681         nan 
     26.370         nan 
      2.125         nan 
 
 BW.02.8  313.0 
     65.351         nan 
     32.040         nan 
      2.141         nan 
 
 BW.02.8  414.0 
     67.423         nan 
     36.510         nan 
      2.169         nan 
 
 BW.02.8  515.0 
     77.934         nan 
     41.880         nan 
      2.196         nan 
 
 BW.02.8  616.0 
     83.123         nan 
     45.350         nan 
      2.214         nan 
 
 BW.02.8  717.0 
     93.775         nan 
     48.980         nan 
      2.211         nan 
 
 BW.02.8  818.0 
    100.879         nan 
     52.730         nan 
      2.235         nan 
 
 BW.02.8  919.0 
    104.109         nan 
     55.870         nan 
      2.243         nan 
 
 BW.02.8  1020.0 
    113.083         nan 
     58.950         nan 
      2.246         nan 
 
 BW.02.9  10.0 
      1.980         nan 
      1.740         nan 
      0.346         nan 
 
 BW.02.9  111.0 
     13.235         nan 
      7.390         nan 
      0.603         nan 
 
 BW.02.9  212.0 
     19.492         nan 
     10.840         nan 
      0.647         nan 
 
 BW.02.9  313.0 
     23.328         nan 
     13.190         nan 
      0.648         nan 
 
 BW.02.9  414.0 
     30.843         nan 
     15.580         nan 
      0.666         nan 
 
 BW.02.9  515.0 
     32.561         nan 
     17.570         nan 
      0.675         nan 
 
 BW.02.9  616.0 
     32.885         nan 
     19.620         nan 
      0.690         nan 
 
 BW.02.9  717.0 
     35.522         nan 
     20.800         nan 
      0.688         nan 
 
 BW.02.9  818.0 
     34.169         nan 
     22.330         nan 
      0.696         nan 
 
 BW.02.9  919.0 
     37.696         nan 
     23.700         nan 
      0.696         nan 
 
 BW.02.9  1020.0 
     38.080         nan 
     24.990         nan 
      0.701         nan 
 
 BW.03.10  10.0 
      8.508         nan 
      4.900         nan 
      1.892         nan 
 
 BW.03.10  111.0 
     40.724         nan 
     20.990         nan 
      2.697         nan 
 
 BW.03.10  212.0 
     65.028         nan 
     30.940         nan 
      2.856         nan 
 
 BW.03.10  313.0 
     70.933         nan 
     37.000         nan 
      2.891         nan 
 
 BW.03.10  414.0 
     84.148         nan 
     43.620         nan 
      2.926         nan 
 
 BW.03.10  515.0 
     97.748         nan 
     48.320         nan 
      2.949         nan 
 
 BW.03.10  616.0 
    103.428         nan 
     53.100         nan 
      2.966         nan 
 
 BW.03.10  717.0 
    106.703         nan 
     57.520         nan 
      2.984         nan 
 
 BW.03.10  818.0 
    120.400         nan 
     61.700         nan 
      2.999         nan 
 
 BW.03.10  919.0 
    125.316         nan 
     65.540         nan 
      3.003         nan 
 
 BW.03.10  1020.0 
    128.304         nan 
     68.920         nan 
      3.013         nan 
 
 BW.03.3  10.0 
      2.220         nan 
      1.870         nan 
      0.405         nan 
 
 BW.03.3  111.0 
     20.246         nan 
      8.420         nan 
      0.639         nan 
 
 BW.03.3  212.0 
     33.517         nan 
     13.590         nan 
      0.706         nan 
 
 BW.03.3  313.0 
     39.976         nan 
     17.790         nan 
      0.737         nan 
 
 BW.03.3  414.0 
     43.512         nan 
     21.340         nan 
      0.765         nan 
 
 BW.03.3  515.0 
     51.468         nan 
     24.860         nan 
      0.785         nan 
 
 BW.03.3  616.0 
     51.627         nan 
     26.810         nan 
      0.785         nan 
 
 BW.03.3  717.0 
     53.828         nan 
     29.730         nan 
      0.794         nan 
 
 BW.03.3  818.0 
     57.726         nan 
     32.080         nan 
      0.800         nan 
 
 BW.03.3  919.0 
     55.957         nan 
     34.080         nan 
      0.804         nan 
 
 BW.03.3  1020.0 
     57.508         nan 
     35.970         nan 
      0.808         nan 
 
 BW.03.5  10.0 
      8.458         nan 
      4.360         nan 
      1.533         nan 
 
 BW.03.5  111.0 
     48.030         nan 
     21.970         nan 
      2.413         nan 
 
 BW.03.5  212.0 
     62.898         nan 
     31.220         nan 
      2.515         nan 
 
 BW.03.5  313.0 
     81.406         nan 
     38.960         nan 
      2.570         nan 
 
 BW.03.5  414.0 
     97.041         nan 
     46.020         nan 
      2.623         nan 
 
 BW.03.5  515.0 
    103.858         nan 
     51.820         nan 
      2.620         nan 
 
 BW.03.5  616.0 
    115.595         nan 
     56.830         nan 
      2.660         nan 
 
 BW.03.5  717.0 
    127.255         nan 
     61.020         nan 
      2.674         nan 
 
 BW.03.5  818.0 
    134.682         nan 
     65.910         nan 
      2.676         nan 
 
 BW.03.5  919.0 
    139.092         nan 
     69.980         nan 
      2.691         nan 
 
 BW.03.5  1020.0 
    148.180         nan 
     73.940         nan 
      2.700         nan 
 
 BW.03.6  10.0 
      9.062         nan 
      4.910         nan 
      1.803         nan 
 
 BW.03.6  111.0 
     53.906         nan 
     23.840         nan 
      2.712         nan 
 
 BW.03.6  212.0 
     83.449         nan 
     35.270         nan 
      2.860         nan 
 
 BW.03.6  313.0 
    101.474         nan 
     44.940         nan 
      2.932         nan 
 
 BW.03.6  414.0 
    111.865         nan 
     52.480         nan 
      2.968         nan 
 
 BW.03.6  515.0 
    128.644         nan 
     60.030         nan 
      3.007         nan 
 
 BW.03.6  616.0 
    134.414         nan 
     65.870         nan 
      3.022         nan 
 
 BW.03.6  717.0 
    152.323         nan 
     73.070         nan 
      3.048         nan 
 
 BW.03.6  818.0 
    164.916         nan 
     78.480         nan 
      3.064         nan 
 
 BW.03.6  919.0 
    182.043         nan 
     84.160         nan 
      3.088         nan 
 
 BW.03.6  1020.0 
    200.065         nan 
     88.930         nan 
      3.093         nan 
 
 BW.03.9  10.0 
      8.050         nan 
      5.230         nan 
      2.101         nan 
 
 BW.03.9  111.0 
     56.439         nan 
     22.980         nan 
      3.023         nan 
 
 BW.03.9  212.0 
     75.439         nan 
     35.800         nan 
      3.223         nan 
 
 BW.03.9  313.0 
     83.578         nan 
     43.990         nan 
      3.266         nan 
 
 BW.03.9  414.0 
    100.656         nan 
     51.350         nan 
      3.300         nan 
 
 BW.03.9  515.0 
    116.510         nan 
     57.820         nan 
      3.318         nan 
 
 BW.03.9  616.0 
    132.851         nan 
     63.580         nan 
      3.345         nan 
 
 BW.03.9  717.0 
    136.433         nan 
     69.020         nan 
      3.377         nan 
 
 BW.03.9  818.0 
    143.582         nan 
     74.050         nan 
      3.390         nan 
 
 BW.03.9  919.0 
    150.550         nan 
     78.640         nan 
      3.396         nan 
 
 BW.03.9  1020.0 
    152.723         nan 
     82.910         nan 
      3.408         nan 
 
 BW.04.10  10.0 
      9.718         nan 
      5.080         nan 
      1.899         nan 
 
 BW.04.10  111.0 
     77.801         nan 
     25.970         nan 
      2.882         nan 
 
 BW.04.10  212.0 
    110.192         nan 
     40.780         nan 
      3.075         nan 
 
 BW.04.10  313.0 
    127.549         nan 
     52.330         nan 
      3.125         nan 
 
 BW.04.10  414.0 
    145.060         nan 
     63.030         nan 
      3.202         nan 
 
 BW.04.10  515.0 
    158.675         nan 
     72.810         nan 
      3.242         nan 
 
 BW.04.10  616.0 
    172.471         nan 
     81.010         nan 
      3.264         nan 
 
 BW.04.10  717.0 
    180.183         nan 
     88.820         nan 
      3.290         nan 
 
 BW.04.10  818.0 
    187.330         nan 
     95.820         nan 
      3.306         nan 
 
 BW.04.10  919.0 
    194.999         nan 
    102.950         nan 
      3.329         nan 
 
 BW.04.10  1020.0 
    205.074         nan 
    108.830         nan 
      3.338         nan 
 
 BW.04.4  10.0 
      5.810         nan 
      3.820         nan 
      1.425         nan 
 
 BW.04.4  111.0 
     39.924         nan 
     18.090         nan 
      2.080         nan 
 
 BW.04.4  212.0 
     59.661         nan 
     27.510         nan 
      2.213         nan 
 
 BW.04.4  313.0 
     73.312         nan 
     33.950         nan 
      2.261         nan 
 
 BW.04.4  414.0 
     94.949         nan 
     39.990         nan 
      2.295         nan 
 
 BW.04.4  515.0 
    102.657         nan 
     44.870         nan 
      2.318         nan 
 
 BW.04.4  616.0 
    105.447         nan 
     49.750         nan 
      2.337         nan 
 
 BW.04.4  717.0 
    109.861         nan 
     53.860         nan 
      2.340         nan 
 
 BW.04.4  818.0 
    114.001         nan 
     58.380         nan 
      2.355         nan 
 
 BW.04.4  919.0 
    117.204         nan 
     62.000         nan 
      2.362         nan 
 
 BW.04.4  1020.0 
    123.416         nan 
     65.960         nan 
      2.373         nan 
 
 BW.04.5  10.0 
      5.570         nan 
      4.080         nan 
      1.671         nan 
 
 BW.04.5  111.0 
     34.220         nan 
     15.200         nan 
      2.208         nan 
 
 BW.04.5  212.0 
     53.576         nan 
     23.580         nan 
      2.354         nan 
 
 BW.04.5  313.0 
     61.141         nan 
     28.450         nan 
      2.355         nan 
 
 BW.04.5  414.0 
     71.864         nan 
     34.580         nan 
      2.402         nan 
 
 BW.04.5  515.0 
     78.584         nan 
     39.420         nan 
      2.417         nan 
 
 BW.04.5  616.0 
     78.673         nan 
     43.780         nan 
      2.437         nan 
 
 BW.04.5  717.0 
     80.728         nan 
     47.170         nan 
      2.440         nan 
 
 BW.04.5  818.0 
     86.383         nan 
     50.840         nan 
      2.457         nan 
 
 BW.04.5  919.0 
     86.392         nan 
     53.880         nan 
      2.463         nan 
 
 BW.04.5  1020.0 
     88.325         nan 
     56.950         nan 
      2.473         nan 
 
 BW.04.6  10.0 
     12.245         nan 
      6.250         nan 
      2.388         nan 
 
 BW.04.6  111.0 
    110.047         nan 
     34.790         nan 
      3.668         nan 
 
 BW.04.6  212.0 
    139.497         nan 
     55.290         nan 
      3.920         nan 
 
 BW.04.6  313.0 
    161.900         nan 
     71.960         nan 
      4.045         nan 
 
 BW.04.6  414.0 
    176.660         nan 
     84.690         nan 
      4.092         nan 
 
 BW.04.6  515.0 
    197.600         nan 
     97.130         nan 
      4.157         nan 
 
 BW.04.6  616.0 
    214.821         nan 
    108.060         nan 
      4.185         nan 
 
 BW.04.6  717.0 
    231.937         nan 
    118.010         nan 
      4.220         nan 
 
 BW.04.6  818.0 
    232.853         nan 
    126.820         nan 
      4.248         nan 
 
 BW.04.6  919.0 
    245.406         nan 
    135.130         nan 
      4.268         nan 
 
 BW.04.6  1020.0 
    252.232         nan 
    142.830         nan 
      4.283         nan 
 
 BW.04.7  10.0 
      6.345         nan 
      4.010         nan 
      1.483         nan 
 
 BW.04.7  111.0 
     29.746         nan 
     16.160         nan 
      2.160         nan 
 
 BW.04.7  212.0 
     44.191         nan 
     22.160         nan 
      2.237         nan 
 
 BW.04.7  313.0 
     55.093         nan 
     26.730         nan 
      2.279         nan 
 
 BW.04.7  414.0 
     62.980         nan 
     30.860         nan 
      2.294         nan 
 
 BW.04.7  515.0 
     70.077         nan 
     34.820         nan 
      2.325         nan 
 
 BW.04.7  616.0 
     80.735         nan 
     38.650         nan 
      2.340         nan 
 
 BW.04.7  717.0 
     87.611         nan 
     41.460         nan 
      2.342         nan 
 
 BW.04.7  818.0 
     88.859         nan 
     44.430         nan 
      2.353         nan 
 
 BW.04.7  919.0 
     87.912         nan 
     47.280         nan 
      2.362         nan 
 
 BW.04.7  1020.0 
     91.072         nan 
     49.970         nan 
      2.368         nan 
 
 BW.04.9  10.0 
      7.863         nan 
      4.430         nan 
      1.670         nan 
 
 BW.04.9  111.0 
     57.776         nan 
     23.600         nan 
      2.653         nan 
 
 BW.04.9  212.0 
     76.485         nan 
     36.060         nan 
      2.778         nan 
 
 BW.04.9  313.0 
     86.717         nan 
     45.550         nan 
      2.887         nan 
 
 BW.04.9  414.0 
    106.526         nan 
     54.200         nan 
      2.921         nan 
 
 BW.04.9  515.0 
    119.204         nan 
     60.220         nan 
      2.949         nan 
 
 BW.04.9  616.0 
    135.464         nan 
     66.670         nan 
      2.971         nan 
 
 BW.04.9  717.0 
    142.388         nan 
     71.850         nan 
      2.971         nan 
 
 BW.04.9  818.0 
    151.896         nan 
     77.000         nan 
      2.993         nan 
 
 BW.04.9  919.0 
    161.466         nan 
     82.010         nan 
      3.007         nan 
 
 BW.04.9  1020.0 
    173.352         nan 
     86.940         nan 
      3.019         nan 
 
 BW.05.1  10.0 
      5.583         nan 
      3.460         nan 
      1.154         nan 
 
 BW.05.1  111.0 
     39.535         nan 
     17.870         nan 
      1.808         nan 
 
 BW.05.1  212.0 
     52.523         nan 
     25.890         nan 
      1.908         nan 
 
 BW.05.1  313.0 
     65.529         nan 
     32.670         nan 
      1.940         nan 
 
 BW.05.1  414.0 
     82.514         nan 
     39.060         nan 
      2.008         nan 
 
 BW.05.1  515.0 
     88.046         nan 
     43.790         nan 
      2.025         nan 
 
 BW.05.1  616.0 
     91.828         nan 
     48.330         nan 
      2.038         nan 
 
 BW.05.1  717.0 
     99.071         nan 
     52.280         nan 
      2.046         nan 
 
 BW.05.1  818.0 
    104.453         nan 
     56.010         nan 
      2.055         nan 
 
 BW.05.1  919.0 
    113.388         nan 
     59.740         nan 
      2.066         nan 
 
 BW.05.1  1020.0 
    121.633         nan 
     62.930         nan 
      2.070         nan 
 
 BW.05.9  10.0 
     13.290         nan 
      6.170         nan 
      2.295         nan 
 
 BW.05.9  111.0 
     88.914         nan 
     33.480         nan 
      3.602         nan 
 
 BW.05.9  212.0 
    101.506         nan 
     49.870         nan 
      3.782         nan 
 
 BW.05.9  313.0 
    129.633         nan 
     63.160         nan 
      3.891         nan 
 
 BW.05.9  414.0 
    141.900         nan 
     73.200         nan 
      3.952         nan 
 
 BW.05.9  515.0 
    159.987         nan 
     82.940         nan 
      3.986         nan 
 
 BW.05.9  616.0 
    174.034         nan 
     90.140         nan 
      4.009         nan 
 
 BW.05.9  717.0 
    196.661         nan 
     99.470         nan 
      4.057         nan 
 
 BW.05.9  818.0 
    210.552         nan 
    105.480         nan 
      4.060         nan 
 
 BW.05.9  919.0 
    221.127         nan 
    112.670         nan 
      4.086         nan 
 
 BW.05.9  1020.0 
    229.670         nan 
    118.810         nan 
      4.098         nan 
 
 BW.06.4  10.0 
     15.275         nan 
      6.370         nan 
      2.325         nan 
 
 BW.06.4  111.0 
    134.923         nan 
     40.130         nan 
      3.859         nan 
 
 BW.06.4  212.0 
    188.623         nan 
     62.340         nan 
      4.095         nan 
 
 BW.06.4  313.0 
    200.641         nan 
     80.130         nan 
      4.197         nan 
 
 BW.06.4  414.0 
    219.984         nan 
     97.940         nan 
      4.324         nan 
 
 BW.06.4  515.0 
    237.006         nan 
    112.120         nan 
      4.362         nan 
 
 BW.06.4  616.0 
    245.996         nan 
    124.440         nan 
      4.408         nan 
 
 BW.06.4  717.0 
    253.468         nan 
    137.240         nan 
      4.450         nan 
 
 BW.06.4  818.0 
    266.583         nan 
    146.820         nan 
      4.459         nan 
 
 BW.06.4  919.0 
    270.596         nan 
    157.470         nan 
      4.495         nan 
 
 BW.06.4  1020.0 
    278.792         nan 
    166.800         nan 
      4.517         nan 
 
 BW.06.6  10.0 
     13.500         nan 
      6.380         nan 
      2.376         nan 
 
 BW.06.6  111.0 
    113.583         nan 
     35.900         nan 
      3.832         nan 
 
 BW.06.6  212.0 
    151.590         nan 
     54.640         nan 
      4.032         nan 
 
 BW.06.6  313.0 
    195.678         nan 
     72.730         nan 
      4.176         nan 
 
 BW.06.6  414.0 
    226.829         nan 
     87.780         nan 
      4.258         nan 
 
 BW.06.6  515.0 
    248.135         nan 
    102.090         nan 
      4.326         nan 
 
 BW.06.6  616.0 
    253.505         nan 
    113.340         nan 
      4.352         nan 
 
 BW.06.6  717.0 
    260.694         nan 
    124.080         nan 
      4.373         nan 
 
 BW.06.6  818.0 
    280.385         nan 
    135.170         nan 
      4.409         nan 
 
 BW.06.6  919.0 
    278.097         nan 
    144.800         nan 
      4.433         nan 
 
 BW.06.6  1020.0 
    280.351         nan 
    153.820         nan 
      4.450         nan 
 
 BW.06.7  10.0 
     14.378         nan 
      6.570         nan 
      2.448         nan 
 
 BW.06.7  111.0 
    107.749         nan 
     35.320         nan 
      3.868         nan 
 
 BW.06.7  212.0 
    152.383         nan 
     55.540         nan 
      4.125         nan 
 
 BW.06.7  313.0 
    189.175         nan 
     70.740         nan 
      4.196         nan 
 
 BW.06.7  414.0 
    213.498         nan 
     85.970         nan 
      4.287         nan 
 
 BW.06.7  515.0 
    214.121         nan 
     99.370         nan 
      4.357         nan 
 
 BW.06.7  616.0 
    227.671         nan 
    110.260         nan 
      4.381         nan 
 
 BW.06.7  717.0 
    229.488         nan 
    121.280         nan 
      4.420         nan 
 
 BW.06.7  818.0 
    236.445         nan 
    130.410         nan 
      4.437         nan 
 
 BW.06.7  919.0 
    235.584         nan 
    139.610         nan 
      4.461         nan 
 
 BW.06.7  1020.0 
    241.387         nan 
    147.840         nan 
      4.482         nan 
 
 BW.06.9  10.0 
     13.723         nan 
      5.870         nan 
      2.147         nan 
 
 BW.06.9  111.0 
     90.891         nan 
     33.470         nan 
      3.388         nan 
 
 BW.06.9  212.0 
    140.029         nan 
     51.450         nan 
      3.581         nan 
 
 BW.06.9  313.0 
    170.023         nan 
     65.400         nan 
      3.672         nan 
 
 BW.06.9  414.0 
    187.999         nan 
     78.780         nan 
      3.749         nan 
 
 BW.06.9  515.0 
    211.538         nan 
     90.470         nan 
      3.805         nan 
 
 BW.06.9  616.0 
    227.754         nan 
    100.900         nan 
      3.852         nan 
 
 BW.06.9  717.0 
    247.142         nan 
    110.650         nan 
      3.880         nan 
 
 BW.06.9  818.0 
    270.222         nan 
    119.610         nan 
      3.900         nan 
 
 BW.06.9  919.0 
    282.354         nan 
    129.070         nan 
      3.931         nan 
 
 BW.06.9  1020.0 
    298.684         nan 
    136.850         nan 
      3.941         nan 
 
 BW.07.3  10.0 
      5.467         nan 
      3.790         nan 
      1.450         nan 
 
 BW.07.3  111.0 
     31.941         nan 
     15.530         nan 
      2.069         nan 
 
 BW.07.3  212.0 
     42.161         nan 
     21.830         nan 
      2.115         nan 
 
 BW.07.3  313.0 
     52.391         nan 
     27.610         nan 
      2.161         nan 
 
 BW.07.3  414.0 
     59.756         nan 
     31.600         nan 
      2.161         nan 
 
 BW.07.3  515.0 
     60.125         nan 
     35.440         nan 
      2.195         nan 
 
 BW.07.3  616.0 
     63.686         nan 
     38.760         nan 
      2.205         nan 
 
 BW.07.3  717.0 
     67.033         nan 
     41.790         nan 
      2.225         nan 
 
 BW.07.3  818.0 
     67.816         nan 
     44.600         nan 
      2.235         nan 
 
 BW.07.3  919.0 
     68.491         nan 
     46.650         nan 
      2.240         nan 
 
 BW.07.3  1020.0 
     69.985         nan 
     48.980         nan 
      2.244         nan 
 
 BW.07.4  10.0 
      8.358         nan 
      4.910         nan 
      1.897         nan 
 
 BW.07.4  111.0 
     55.237         nan 
     22.330         nan 
      2.740         nan 
 
 BW.07.4  212.0 
     68.257         nan 
     33.670         nan 
      2.888         nan 
 
 BW.07.4  313.0 
     75.808         nan 
     42.020         nan 
      2.943         nan 
 
 BW.07.4  414.0 
     85.018         nan 
     48.580         nan 
      2.973         nan 
 
 BW.07.4  515.0 
     94.497         nan 
     54.580         nan 
      2.993         nan 
 
 BW.07.4  616.0 
    106.808         nan 
     60.420         nan 
      3.030         nan 
 
 BW.07.4  717.0 
    114.999         nan 
     65.130         nan 
      3.045         nan 
 
 BW.07.4  818.0 
    119.053         nan 
     68.960         nan 
      3.054         nan 
 
 BW.07.4  919.0 
    131.723         nan 
     73.180         nan 
      3.064         nan 
 
 BW.07.4  1020.0 
    142.857         nan 
     76.930         nan 
      3.079         nan 
 
 BW.07.5  10.0 
      6.550         nan 
      4.380         nan 
      1.769         nan 
 
 BW.07.5  111.0 
     46.050         nan 
     19.620         nan 
      2.587         nan 
 
 BW.07.5  212.0 
     70.754         nan 
     28.300         nan 
      2.666         nan 
 
 BW.07.5  313.0 
     87.388         nan 
     35.540         nan 
      2.733         nan 
 
 BW.07.5  414.0 
     99.452         nan 
     42.440         nan 
      2.774         nan 
 
 BW.07.5  515.0 
    107.519         nan 
     49.130         nan 
      2.804         nan 
 
 BW.07.5  616.0 
    111.082         nan 
     54.150         nan 
      2.812         nan 
 
 BW.07.5  717.0 
    107.190         nan 
     58.810         nan 
      2.825         nan 
 
 BW.07.5  818.0 
    103.966         nan 
     63.060         nan 
      2.841         nan 
 
 BW.07.5  919.0 
    101.009         nan 
     67.090         nan 
      2.847         nan 
 
 BW.07.5  1020.0 
    100.734         nan 
     70.940         nan 
      2.856         nan 
 
 BW.07.8  10.0 
      4.633         nan 
      3.630         nan 
      1.513         nan 
 
 BW.07.8  111.0 
     29.357         nan 
     13.590         nan 
      1.972         nan 
 
 BW.07.8  212.0 
     46.711         nan 
     19.740         nan 
      2.051         nan 
 
 BW.07.8  313.0 
     53.536         nan 
     24.860         nan 
      2.096         nan 
 
 BW.07.8  414.0 
     54.367         nan 
     28.820         nan 
      2.104         nan 
 
 BW.07.8  515.0 
     56.749         nan 
     32.550         nan 
      2.121         nan 
 
 BW.07.8  616.0 
     59.073         nan 
     35.470         nan 
      2.131         nan 
 
 BW.07.8  717.0 
     66.363         nan 
     38.760         nan 
      2.148         nan 
 
 BW.07.8  818.0 
     68.393         nan 
     41.330         nan 
      2.155         nan 
 
 BW.07.8  919.0 
     73.484         nan 
     43.870         nan 
      2.164         nan 
 
 BW.07.8  1020.0 
     78.953         nan 
     45.970         nan 
      2.167         nan 
 
 BW.09.3  10.0 
     13.557         nan 
      6.380         nan 
      2.402         nan 
 
 BW.09.3  111.0 
    107.452         nan 
     34.990         nan 
      3.780         nan 
 
 BW.09.3  212.0 
    138.971         nan 
     54.000         nan 
      3.968         nan 
 
 BW.09.3  313.0 
    171.448         nan 
     71.360         nan 
      4.108         nan 
 
 BW.09.3  414.0 
    183.803         nan 
     83.630         nan 
      4.162         nan 
 
 BW.09.3  515.0 
    202.388         nan 
     95.410         nan 
      4.216         nan 
 
 BW.09.3  616.0 
    208.528         nan 
    105.410         nan 
      4.241         nan 
 
 BW.09.3  717.0 
    223.689         nan 
    115.780         nan 
      4.279         nan 
 
 BW.09.3  818.0 
    235.559         nan 
    124.990         nan 
      4.313         nan 
 
 BW.09.3  919.0 
    251.040         nan 
    133.530         nan 
      4.324         nan 
 
 BW.09.3  1020.0 
    254.594         nan 
    140.830         nan 
      4.343         nan 
 
 BW.09.4  10.0 
     11.012         nan 
      6.120         nan 
      2.367         nan 
 
 BW.09.4  111.0 
     97.595         nan 
     28.710         nan 
      3.463         nan 
 
 BW.09.4  212.0 
    122.103         nan 
     44.880         nan 
      3.644         nan 
 
 BW.09.4  313.0 
    142.655         nan 
     59.010         nan 
      3.747         nan 
 
 BW.09.4  414.0 
    157.261         nan 
     70.520         nan 
      3.802         nan 
 
 BW.09.4  515.0 
    166.979         nan 
     80.930         nan 
      3.838         nan 
 
 BW.09.4  616.0 
    175.510         nan 
     90.330         nan 
      3.875         nan 
 
 BW.09.4  717.0 
    183.228         nan 
     99.310         nan 
      3.907         nan 
 
 BW.09.4  818.0 
    191.016         nan 
    107.860         nan 
      3.938         nan 
 
 BW.09.4  919.0 
    188.544         nan 
    114.160         nan 
      3.944         nan 
 
 BW.09.4  1020.0 
    190.769         nan 
    120.830         nan 
      3.962         nan 
 
 BW.09.9  10.0 
      5.783         nan 
      4.140         nan 
      1.692         nan 
 
 BW.09.9  111.0 
     54.538         nan 
     18.250         nan 
      2.359         nan 
 
 BW.09.9  212.0 
     70.568         nan 
     27.490         nan 
      2.454         nan 
 
 BW.09.9  313.0 
     84.073         nan 
     36.270         nan 
      2.513         nan 
 
 BW.09.9  414.0 
     85.183         nan 
     43.350         nan 
      2.565         nan 
 
 BW.09.9  515.0 
     89.642         nan 
     49.660         nan 
      2.590         nan 
 
 BW.09.9  616.0 
     86.395         nan 
     54.560         nan 
      2.608         nan 
 
 BW.09.9  717.0 
     90.032         nan 
     59.300         nan 
      2.631         nan 
 
 BW.09.9  818.0 
     91.484         nan 
     63.440         nan 
      2.638         nan 
 
 BW.09.9  919.0 
     90.262         nan 
     66.900         nan 
      2.647         nan 
 
 BW.09.9  1020.0 
     90.407         nan 
     69.960         nan 
      2.655         nan 
 
 BW.10.10  10.0 
      5.165         nan 
      3.400         nan 
      1.176         nan 
 
 BW.10.10  111.0 
     39.781         nan 
     15.170         nan 
      1.737         nan 
 
 BW.10.10  212.0 
     55.227         nan 
     23.300         nan 
      1.817         nan 
 
 BW.10.10  313.0 
     61.202         nan 
     30.180         nan 
      1.885         nan 
 
 BW.10.10  414.0 
     63.375         nan 
     36.000         nan 
      1.937         nan 
 
 BW.10.10  515.0 
     65.833         nan 
     40.200         nan 
      1.943         nan 
 
 BW.10.10  616.0 
     65.854         nan 
     43.920         nan 
      1.956         nan 
 
 BW.10.10  717.0 
     68.632         nan 
     47.240         nan 
      1.967         nan 
 
 BW.10.10  818.0 
     67.865         nan 
     50.160         nan 
      1.976         nan 
 
 BW.10.10  919.0 
     66.345         nan 
     52.860         nan 
      1.982         nan 
 
 BW.10.10  1020.0 
     65.629         nan 
     54.980         nan 
      1.988         nan 
 
  TimePoint  Seqs/Sample 
 chao1 Ave.  chao1 Err. 
 observed_species Ave.  observed_species Err. 
 shannon Ave.  shannon Err. 
 
 
 1  10.0 
      6.824       1.498 
      4.277       0.851 
      1.585       0.414 
 
 1  111.0 
     37.636       1.954 
     17.703       1.955 
      2.292       0.536 
 
 1  212.0 
     50.169       4.414 
     25.547       2.392 
      2.407       0.551 
 
 1  313.0 
     61.264       3.236 
     31.690       2.499 
      2.452       0.563 
 
 1  414.0 
     75.493       5.082 
     37.103       2.760 
      2.502       0.551 
 
 1  515.0 
     78.878       6.489 
     41.313       3.135 
      2.514       0.554 
 
 1  616.0 
     86.687       6.473 
     45.347       3.180 
      2.528       0.553 
 
 1  717.0 
     92.745       4.780 
     48.933       3.377 
      2.537       0.556 
 
 1  818.0 
     95.031       7.299 
     52.373       3.493 
      2.546       0.558 
 
 1  919.0 
    100.146       9.799 
     55.570       3.607 
      2.556       0.553 
 
 1  1020.0 
    103.900      12.543 
     58.623       3.675 
      2.563       0.556 
 
 3  10.0 
      9.930       6.433 
      4.938       2.264 
      1.767       0.929 
 
 3  111.0 
     73.664      48.734 
     25.475      14.018 
      2.734       1.489 
 
 3  212.0 
    100.239      64.679 
     39.163      22.149 
      2.881       1.576 
 
 3  313.0 
    118.409      73.620 
     51.142      29.238 
      2.969       1.624 
 
 3  414.0 
    132.006      83.072 
     60.443      35.006 
      3.010       1.647 
 
 3  515.0 
    139.976      85.629 
     68.918      39.903 
      3.051       1.662 
 
 3  616.0 
    146.609      91.083 
     76.220      44.787 
      3.075       1.684 
 
 3  717.0 
    155.484      97.043 
     83.755      49.490 
      3.105       1.701 
 
 3  818.0 
    161.954     100.882 
     90.157      53.375 
      3.124       1.710 
 
 3  919.0 
    167.619     106.626 
     95.755      56.947 
      3.134       1.717 
 
 3  1020.0 
    172.893     110.946 
    101.135      60.349 
      3.146       1.724 
 
 4  10.0 
      8.519       4.470 
      4.606       1.669 
      1.678       0.734 
 
 4  111.0 
     70.070      40.839 
     23.608      10.497 
      2.560       1.130 
 
 4  212.0 
     95.008      54.587 
     36.308      16.567 
      2.697       1.211 
 
 4  313.0 
    106.749      57.377 
     46.580      21.398 
      2.770       1.229 
 
 4  414.0 
    120.492      61.356 
     55.784      26.247 
      2.825       1.256 
 
 4  515.0 
    129.479      66.086 
     63.398      30.384 
      2.848       1.270 
 
 4  616.0 
    136.181      68.325 
     70.508      33.691 
      2.880       1.279 
 
 4  717.0 
    142.319      69.776 
     77.182      37.355 
      2.901       1.290 
 
 4  818.0 
    148.104      74.211 
     82.926      40.083 
      2.914       1.296 
 
 4  919.0 
    151.682      73.968 
     88.254      43.063 
      2.927       1.303 
 
 4  1020.0 
    157.379      75.529 
     93.294      45.748 
      2.940       1.311 
 
 5  10.0 
      6.859       1.199 
      4.273       0.137 
      1.658       0.097 
 
 5  111.0 
     42.767       6.097 
     18.930       2.807 
      2.403       0.155 
 
 5  212.0 
     62.410       7.021 
     27.700       3.148 
      2.512       0.127 
 
 5  313.0 
     76.645      11.232 
     34.317       4.377 
      2.552       0.155 
 
 5  414.0 
     89.452      12.476 
     41.013       4.778 
      2.599       0.153 
 
 5  515.0 
     96.654      12.864 
     46.790       5.326 
      2.614       0.158 
 
 5  616.0 
    101.783      16.445 
     51.587       5.628 
      2.636       0.154 
 
 5  717.0 
    105.058      19.054 
     55.667       6.075 
      2.647       0.158 
 
 5  818.0 
    108.344      19.960 
     59.937       6.537 
      2.658       0.157 
 
 5  919.0 
    108.831      22.214 
     63.650       7.008 
      2.667       0.158 
 
 5  1020.0 
    112.413      25.794 
     67.277       7.404 
      2.676       0.157 
 
 6  10.0 
      9.773       3.558 
      5.122       1.380 
      1.872       0.597 
 
 6  111.0 
     78.082      34.427 
     27.378       8.570 
      2.890       0.987 
 
 6  212.0 
    107.070      40.110 
     42.100      13.556 
      3.061       1.045 
 
 6  313.0 
    132.386      49.151 
     54.968      18.140 
      3.164       1.074 
 
 6  414.0 
    148.370      57.495 
     65.315      21.715 
      3.216       1.084 
 
 6  515.0 
    166.750      60.254 
     74.957      25.654 
      3.259       1.112 
 
 6  616.0 
    175.747      61.200 
     82.942      28.828 
      3.275       1.124 
 
 6  717.0 
    187.379      62.145 
     91.140      31.128 
      3.303       1.123 
 
 6  818.0 
    195.435      67.052 
     98.455      33.860 
      3.327       1.132 
 
 6  919.0 
    202.361      66.515 
    105.177      36.286 
      3.344       1.139 
 
 6  1020.0 
    208.332      68.504 
    111.377      38.540 
      3.355       1.144 
 
 7  10.0 
      8.343       4.348 
      4.510       1.520 
      1.615       0.633 
 
 7  111.0 
     54.966      37.336 
     21.407       9.937 
      2.437       1.073 
 
 7  212.0 
     78.329      52.417 
     32.187      16.568 
      2.579       1.148 
 
 7  313.0 
     97.838      64.629 
     40.477      21.429 
      2.629       1.164 
 
 7  414.0 
    110.686      72.761 
     48.113      26.804 
      2.671       1.195 
 
 7  515.0 
    116.038      69.401 
     55.183      31.277 
      2.713       1.215 
 
 7  616.0 
    125.167      72.695 
     60.990      34.890 
      2.725       1.226 
 
 7  717.0 
    129.603      70.927 
     66.687      38.640 
      2.748       1.234 
 
 7  818.0 
    134.531      72.193 
     71.670      41.572 
      2.757       1.240 
 
 7  919.0 
    136.189      70.293 
     76.517      44.654 
      2.768       1.249 
 
 7  1020.0 
    139.025      72.428 
     80.927      47.359 
      2.780       1.256 
 
 8  10.0 
      5.299       0.666 
      3.605       0.025 
      1.363       0.150 
 
 8  111.0 
     35.896       6.538 
     16.100       2.510 
      1.983       0.011 
 
 8  212.0 
     50.196       3.485 
     23.055       3.315 
      2.088       0.037 
 
 8  313.0 
     59.444       5.908 
     28.450       3.590 
      2.118       0.023 
 
 8  414.0 
     60.895       6.528 
     32.665       3.845 
      2.136       0.033 
 
 8  515.0 
     67.342      10.593 
     37.215       4.665 
      2.159       0.037 
 
 8  616.0 
     71.098      12.025 
     40.410       4.940 
      2.173       0.041 
 
 8  717.0 
     80.069      13.706 
     43.870       5.110 
      2.179       0.031 
 
 8  818.0 
     84.636      16.243 
     47.030       5.700 
      2.195       0.040 
 
 8  919.0 
     88.796      15.313 
     49.870       6.000 
      2.204       0.039 
 
 8  1020.0 
     96.018      17.065 
     52.460       6.490 
      2.207       0.039 
 
 9  10.0 
      8.448       4.097 
      4.597       1.466 
      1.708       0.652 
 
 9  111.0 
     60.299      25.898 
     23.195       8.998 
      2.605       0.988 
 
 9  212.0 
     80.586      36.165 
     35.252      13.743 
      2.744       1.040 
 
 9  313.0 
     96.225      45.229 
     44.593      17.485 
      2.813       1.071 
 
 9  414.0 
    108.851      48.433 
     52.743      20.695 
      2.859       1.086 
 
 9  515.0 
    121.574      55.593 
     59.780      23.687 
      2.887       1.097 
 
 9  616.0 
    131.564      61.713 
     65.912      26.106 
      2.912       1.103 
 
 9  717.0 
    141.363      68.447 
     71.848      28.983 
      2.934       1.117 
 
 9  818.0 
    150.318      76.382 
     76.985      31.113 
      2.946       1.119 
 
 9  919.0 
    157.242      80.181 
     82.165      33.651 
      2.961       1.127 
 
 9  1020.0 
    163.819      85.575 
     86.743      35.704 
      2.970       1.130 
 
 10  10.0 
      8.131       1.765 
      4.553       0.672 
      1.691       0.300 
 
 10  111.0 
     56.941      16.951 
     21.890       4.334 
      2.487       0.443 
 
 10  212.0 
     83.492      23.738 
     33.970       7.363 
      2.647       0.488 
 
 10  313.0 
     94.871      29.137 
     42.795       9.518 
      2.693       0.477 
 
 10  414.0 
    105.510      33.051 
     51.125      11.639 
      2.745       0.481 
 
 10  515.0 
    113.205      34.828 
     57.877      13.948 
      2.774       0.494 
 
 10  616.0 
    121.211      40.271 
     63.992      15.856 
      2.792       0.497 
 
 10  717.0 
    126.496      42.417 
     69.543      17.607 
      2.811       0.502 
 
 10  818.0 
    133.361      44.638 
     74.695      19.276 
      2.826       0.506 
 
 10  919.0 
    136.913      47.614 
     79.480      20.892 
      2.838       0.510 
 
 10  1020.0 
    141.173      51.373 
     83.668      22.403 
      2.848       0.512 
 
  Description  Seqs/Sample 
 chao1 Ave.  chao1 Err. 
 observed_species Ave.  observed_species Err. 
 shannon Ave.  shannon Err. 
 
 
 BW-01.1.Lib_L_27F-001  10.0 
      8.932         nan 
      5.450         nan 
      2.144         nan 
 
 BW-01.1.Lib_L_27F-001  111.0 
     38.424         nan 
     20.010         nan 
      3.040         nan 
 
 BW-01.1.Lib_L_27F-001  212.0 
     53.999         nan 
     28.290         nan 
      3.175         nan 
 
 BW-01.1.Lib_L_27F-001  313.0 
     60.567         nan 
     34.140         nan 
      3.235         nan 
 
 BW-01.1.Lib_L_27F-001  414.0 
     70.652         nan 
     39.050         nan 
      3.271         nan 
 
 BW-01.1.Lib_L_27F-001  515.0 
     74.640         nan 
     43.260         nan 
      3.289         nan 
 
 BW-01.1.Lib_L_27F-001  616.0 
     77.557         nan 
     46.770         nan 
      3.301         nan 
 
 BW-01.1.Lib_L_27F-001  717.0 
     87.518         nan 
     50.210         nan 
      3.313         nan 
 
 BW-01.1.Lib_L_27F-001  818.0 
     86.669         nan 
     53.450         nan 
      3.327         nan 
 
 BW-01.1.Lib_L_27F-001  919.0 
     89.987         nan 
     56.030         nan 
      3.328         nan 
 
 BW-01.1.Lib_L_27F-001  1020.0 
     95.425         nan 
     58.990         nan 
      3.341         nan 
 
 BW-01.3.Lib_L_27F-003  10.0 
     18.475         nan 
      7.710         nan 
      2.811         nan 
 
 BW-01.3.Lib_L_27F-003  111.0 
    135.017         nan 
     42.960         nan 
      4.448         nan 
 
 BW-01.3.Lib_L_27F-003  212.0 
    186.310         nan 
     67.230         nan 
      4.736         nan 
 
 BW-01.3.Lib_L_27F-003  313.0 
    209.821         nan 
     87.810         nan 
      4.870         nan 
 
 BW-01.3.Lib_L_27F-003  414.0 
    240.953         nan 
    105.200         nan 
      4.950         nan 
 
 BW-01.3.Lib_L_27F-003  515.0 
    245.922         nan 
    119.960         nan 
      5.006         nan 
 
 BW-01.3.Lib_L_27F-003  616.0 
    262.596         nan 
    133.900         nan 
      5.070         nan 
 
 BW-01.3.Lib_L_27F-003  717.0 
    277.386         nan 
    147.720         nan 
      5.124         nan 
 
 BW-01.3.Lib_L_27F-003  818.0 
    286.716         nan 
    158.960         nan 
      5.148         nan 
 
 BW-01.3.Lib_L_27F-003  919.0 
    294.986         nan 
    168.760         nan 
      5.169         nan 
 
 BW-01.3.Lib_L_27F-003  1020.0 
    309.484         nan 
    178.760         nan 
      5.189         nan 
 
 BW-01.4.Lib_L_27F-004  10.0 
      2.140         nan 
      1.810         nan 
      0.379         nan 
 
 BW-01.4.Lib_L_27F-004  111.0 
     22.669         nan 
      8.780         nan 
      0.657         nan 
 
 BW-01.4.Lib_L_27F-004  212.0 
     36.394         nan 
     13.140         nan 
      0.645         nan 
 
 BW-01.4.Lib_L_27F-004  313.0 
     41.327         nan 
     17.790         nan 
      0.702         nan 
 
 BW-01.4.Lib_L_27F-004  414.0 
     45.250         nan 
     21.890         nan 
      0.731         nan 
 
 BW-01.4.Lib_L_27F-004  515.0 
     46.259         nan 
     24.490         nan 
      0.728         nan 
 
 BW-01.4.Lib_L_27F-004  616.0 
     47.142         nan 
     27.600         nan 
      0.748         nan 
 
 BW-01.4.Lib_L_27F-004  717.0 
     50.037         nan 
     30.370         nan 
      0.763         nan 
 
 BW-01.4.Lib_L_27F-004  818.0 
     49.868         nan 
     32.610         nan 
      0.763         nan 
 
 BW-01.4.Lib_L_27F-004  919.0 
     50.341         nan 
     34.460         nan 
      0.769         nan 
 
 BW-01.4.Lib_L_27F-004  1020.0 
     51.062         nan 
     35.950         nan 
      0.769         nan 
 
 BW-02.1.Lib_L_27F-011  10.0 
      5.957         nan 
      3.920         nan 
      1.456         nan 
 
 BW-02.1.Lib_L_27F-011  111.0 
     34.947         nan 
     15.230         nan 
      2.029         nan 
 
 BW-02.1.Lib_L_27F-011  212.0 
     43.986         nan 
     22.460         nan 
      2.137         nan 
 
 BW-02.1.Lib_L_27F-011  313.0 
     57.696         nan 
     28.260         nan 
      2.181         nan 
 
 BW-02.1.Lib_L_27F-011  414.0 
     73.314         nan 
     33.200         nan 
      2.227         nan 
 
 BW-02.1.Lib_L_27F-011  515.0 
     73.949         nan 
     36.890         nan 
      2.228         nan 
 
 BW-02.1.Lib_L_27F-011  616.0 
     90.677         nan 
     40.940         nan 
      2.245         nan 
 
 BW-02.1.Lib_L_27F-011  717.0 
     91.648         nan 
     44.310         nan 
      2.251         nan 
 
 BW-02.1.Lib_L_27F-011  818.0 
     93.969         nan 
     47.660         nan 
      2.257         nan 
 
 BW-02.1.Lib_L_27F-011  919.0 
     97.062         nan 
     50.940         nan 
      2.272         nan 
 
 BW-02.1.Lib_L_27F-011  1020.0 
     94.643         nan 
     53.950         nan 
      2.278         nan 
 
 BW-02.10.Lib_L_27F-021  10.0 
      9.133         nan 
      4.830         nan 
      1.796         nan 
 
 BW-02.10.Lib_L_27F-021  111.0 
     69.459         nan 
     25.430         nan 
      2.633         nan 
 
 BW-02.10.Lib_L_27F-021  212.0 
    103.522         nan 
     40.860         nan 
      2.840         nan 
 
 BW-02.10.Lib_L_27F-021  313.0 
    119.800         nan 
     51.670         nan 
      2.871         nan 
 
 BW-02.10.Lib_L_27F-021  414.0 
    129.456         nan 
     61.850         nan 
      2.915         nan 
 
 BW-02.10.Lib_L_27F-021  515.0 
    130.565         nan 
     70.180         nan 
      2.964         nan 
 
 BW-02.10.Lib_L_27F-021  616.0 
    143.092         nan 
     77.940         nan 
      2.983         nan 
 
 BW-02.10.Lib_L_27F-021  717.0 
    150.467         nan 
     84.590         nan 
      3.002         nan 
 
 BW-02.10.Lib_L_27F-021  818.0 
    157.851         nan 
     91.100         nan 
      3.024         nan 
 
 BW-02.10.Lib_L_27F-021  919.0 
    160.994         nan 
     96.570         nan 
      3.037         nan 
 
 BW-02.10.Lib_L_27F-021  1020.0 
    165.686         nan 
    101.940         nan 
      3.052         nan 
 
 BW-02.6.Lib_L_27F-017  10.0 
      4.285         nan 
      2.950         nan 
      0.922         nan 
 
 BW-02.6.Lib_L_27F-017  111.0 
     34.790         nan 
     14.980         nan 
      1.349         nan 
 
 BW-02.6.Lib_L_27F-017  212.0 
     53.743         nan 
     23.200         nan 
      1.433         nan 
 
 BW-02.6.Lib_L_27F-017  313.0 
     70.492         nan 
     30.240         nan 
      1.503         nan 
 
 BW-02.6.Lib_L_27F-017  414.0 
     78.128         nan 
     36.310         nan 
      1.547         nan 
 
 BW-02.6.Lib_L_27F-017  515.0 
     92.622         nan 
     40.580         nan 
      1.545         nan 
 
 BW-02.6.Lib_L_27F-017  616.0 
    100.248         nan 
     44.500         nan 
      1.542         nan 
 
 BW-02.6.Lib_L_27F-017  717.0 
    104.564         nan 
     49.400         nan 
      1.571         nan 
 
 BW-02.6.Lib_L_27F-017  818.0 
    103.588         nan 
     53.350         nan 
      1.584         nan 
 
 BW-02.6.Lib_L_27F-017  919.0 
    103.897         nan 
     56.620         nan 
      1.586         nan 
 
 BW-02.6.Lib_L_27F-017  1020.0 
    100.680         nan 
     59.930         nan 
      1.594         nan 
 
 BW-02.7.Lib_L_27F-018  10.0 
      4.305         nan 
      2.950         nan 
      0.915         nan 
 
 BW-02.7.Lib_L_27F-018  111.0 
     27.402         nan 
     12.740         nan 
      1.283         nan 
 
 BW-02.7.Lib_L_27F-018  212.0 
     38.412         nan 
     18.860         nan 
      1.374         nan 
 
 BW-02.7.Lib_L_27F-018  313.0 
     49.246         nan 
     23.960         nan 
      1.411         nan 
 
 BW-02.7.Lib_L_27F-018  414.0 
     55.581         nan 
     27.510         nan 
      1.432         nan 
 
 BW-02.7.Lib_L_27F-018  515.0 
     63.915         nan 
     31.360         nan 
      1.458         nan 
 
 BW-02.7.Lib_L_27F-018  616.0 
     67.094         nan 
     34.060         nan 
      1.454         nan 
 
 BW-02.7.Lib_L_27F-018  717.0 
     71.711         nan 
     37.320         nan 
      1.481         nan 
 
 BW-02.7.Lib_L_27F-018  818.0 
     78.289         nan 
     40.170         nan 
      1.481         nan 
 
 BW-02.7.Lib_L_27F-018  919.0 
     85.069         nan 
     42.660         nan 
      1.483         nan 
 
 BW-02.7.Lib_L_27F-018  1020.0 
     84.617         nan 
     44.970         nan 
      1.489         nan 
 
 BW-02.8.Lib_L_27F-019  10.0 
      5.965         nan 
      3.580         nan 
      1.212         nan 
 
 BW-02.8.Lib_L_27F-019  111.0 
     42.434         nan 
     18.610         nan 
      1.994         nan 
 
 BW-02.8.Lib_L_27F-019  212.0 
     53.681         nan 
     26.370         nan 
      2.125         nan 
 
 BW-02.8.Lib_L_27F-019  313.0 
     65.351         nan 
     32.040         nan 
      2.141         nan 
 
 BW-02.8.Lib_L_27F-019  414.0 
     67.423         nan 
     36.510         nan 
      2.169         nan 
 
 BW-02.8.Lib_L_27F-019  515.0 
     77.934         nan 
     41.880         nan 
      2.196         nan 
 
 BW-02.8.Lib_L_27F-019  616.0 
     83.123         nan 
     45.350         nan 
      2.214         nan 
 
 BW-02.8.Lib_L_27F-019  717.0 
     93.775         nan 
     48.980         nan 
      2.211         nan 
 
 BW-02.8.Lib_L_27F-019  818.0 
    100.879         nan 
     52.730         nan 
      2.235         nan 
 
 BW-02.8.Lib_L_27F-019  919.0 
    104.109         nan 
     55.870         nan 
      2.243         nan 
 
 BW-02.8.Lib_L_27F-019  1020.0 
    113.083         nan 
     58.950         nan 
      2.246         nan 
 
 BW-02.9.Lib_L_27F-020  10.0 
      1.980         nan 
      1.740         nan 
      0.346         nan 
 
 BW-02.9.Lib_L_27F-020  111.0 
     13.235         nan 
      7.390         nan 
      0.603         nan 
 
 BW-02.9.Lib_L_27F-020  212.0 
     19.492         nan 
     10.840         nan 
      0.647         nan 
 
 BW-02.9.Lib_L_27F-020  313.0 
     23.328         nan 
     13.190         nan 
      0.648         nan 
 
 BW-02.9.Lib_L_27F-020  414.0 
     30.843         nan 
     15.580         nan 
      0.666         nan 
 
 BW-02.9.Lib_L_27F-020  515.0 
     32.561         nan 
     17.570         nan 
      0.675         nan 
 
 BW-02.9.Lib_L_27F-020  616.0 
     32.885         nan 
     19.620         nan 
      0.690         nan 
 
 BW-02.9.Lib_L_27F-020  717.0 
     35.522         nan 
     20.800         nan 
      0.688         nan 
 
 BW-02.9.Lib_L_27F-020  818.0 
     34.169         nan 
     22.330         nan 
      0.696         nan 
 
 BW-02.9.Lib_L_27F-020  919.0 
     37.696         nan 
     23.700         nan 
      0.696         nan 
 
 BW-02.9.Lib_L_27F-020  1020.0 
     38.080         nan 
     24.990         nan 
      0.701         nan 
 
 BW-03.10.Lib_L_27F-031  10.0 
      8.508         nan 
      4.900         nan 
      1.892         nan 
 
 BW-03.10.Lib_L_27F-031  111.0 
     40.724         nan 
     20.990         nan 
      2.697         nan 
 
 BW-03.10.Lib_L_27F-031  212.0 
     65.028         nan 
     30.940         nan 
      2.856         nan 
 
 BW-03.10.Lib_L_27F-031  313.0 
     70.933         nan 
     37.000         nan 
      2.891         nan 
 
 BW-03.10.Lib_L_27F-031  414.0 
     84.148         nan 
     43.620         nan 
      2.926         nan 
 
 BW-03.10.Lib_L_27F-031  515.0 
     97.748         nan 
     48.320         nan 
      2.949         nan 
 
 BW-03.10.Lib_L_27F-031  616.0 
    103.428         nan 
     53.100         nan 
      2.966         nan 
 
 BW-03.10.Lib_L_27F-031  717.0 
    106.703         nan 
     57.520         nan 
      2.984         nan 
 
 BW-03.10.Lib_L_27F-031  818.0 
    120.400         nan 
     61.700         nan 
      2.999         nan 
 
 BW-03.10.Lib_L_27F-031  919.0 
    125.316         nan 
     65.540         nan 
      3.003         nan 
 
 BW-03.10.Lib_L_27F-031  1020.0 
    128.304         nan 
     68.920         nan 
      3.013         nan 
 
 BW-03.3.Lib_L_27F-024  10.0 
      2.220         nan 
      1.870         nan 
      0.405         nan 
 
 BW-03.3.Lib_L_27F-024  111.0 
     20.246         nan 
      8.420         nan 
      0.639         nan 
 
 BW-03.3.Lib_L_27F-024  212.0 
     33.517         nan 
     13.590         nan 
      0.706         nan 
 
 BW-03.3.Lib_L_27F-024  313.0 
     39.976         nan 
     17.790         nan 
      0.737         nan 
 
 BW-03.3.Lib_L_27F-024  414.0 
     43.512         nan 
     21.340         nan 
      0.765         nan 
 
 BW-03.3.Lib_L_27F-024  515.0 
     51.468         nan 
     24.860         nan 
      0.785         nan 
 
 BW-03.3.Lib_L_27F-024  616.0 
     51.627         nan 
     26.810         nan 
      0.785         nan 
 
 BW-03.3.Lib_L_27F-024  717.0 
     53.828         nan 
     29.730         nan 
      0.794         nan 
 
 BW-03.3.Lib_L_27F-024  818.0 
     57.726         nan 
     32.080         nan 
      0.800         nan 
 
 BW-03.3.Lib_L_27F-024  919.0 
     55.957         nan 
     34.080         nan 
      0.804         nan 
 
 BW-03.3.Lib_L_27F-024  1020.0 
     57.508         nan 
     35.970         nan 
      0.808         nan 
 
 BW-03.5.Lib_L_27F-026  10.0 
      8.458         nan 
      4.360         nan 
      1.533         nan 
 
 BW-03.5.Lib_L_27F-026  111.0 
     48.030         nan 
     21.970         nan 
      2.413         nan 
 
 BW-03.5.Lib_L_27F-026  212.0 
     62.898         nan 
     31.220         nan 
      2.515         nan 
 
 BW-03.5.Lib_L_27F-026  313.0 
     81.406         nan 
     38.960         nan 
      2.570         nan 
 
 BW-03.5.Lib_L_27F-026  414.0 
     97.041         nan 
     46.020         nan 
      2.623         nan 
 
 BW-03.5.Lib_L_27F-026  515.0 
    103.858         nan 
     51.820         nan 
      2.620         nan 
 
 BW-03.5.Lib_L_27F-026  616.0 
    115.595         nan 
     56.830         nan 
      2.660         nan 
 
 BW-03.5.Lib_L_27F-026  717.0 
    127.255         nan 
     61.020         nan 
      2.674         nan 
 
 BW-03.5.Lib_L_27F-026  818.0 
    134.682         nan 
     65.910         nan 
      2.676         nan 
 
 BW-03.5.Lib_L_27F-026  919.0 
    139.092         nan 
     69.980         nan 
      2.691         nan 
 
 BW-03.5.Lib_L_27F-026  1020.0 
    148.180         nan 
     73.940         nan 
      2.700         nan 
 
 BW-03.6.Lib_L_27F-027  10.0 
      9.062         nan 
      4.910         nan 
      1.803         nan 
 
 BW-03.6.Lib_L_27F-027  111.0 
     53.906         nan 
     23.840         nan 
      2.712         nan 
 
 BW-03.6.Lib_L_27F-027  212.0 
     83.449         nan 
     35.270         nan 
      2.860         nan 
 
 BW-03.6.Lib_L_27F-027  313.0 
    101.474         nan 
     44.940         nan 
      2.932         nan 
 
 BW-03.6.Lib_L_27F-027  414.0 
    111.865         nan 
     52.480         nan 
      2.968         nan 
 
 BW-03.6.Lib_L_27F-027  515.0 
    128.644         nan 
     60.030         nan 
      3.007         nan 
 
 BW-03.6.Lib_L_27F-027  616.0 
    134.414         nan 
     65.870         nan 
      3.022         nan 
 
 BW-03.6.Lib_L_27F-027  717.0 
    152.323         nan 
     73.070         nan 
      3.048         nan 
 
 BW-03.6.Lib_L_27F-027  818.0 
    164.916         nan 
     78.480         nan 
      3.064         nan 
 
 BW-03.6.Lib_L_27F-027  919.0 
    182.043         nan 
     84.160         nan 
      3.088         nan 
 
 BW-03.6.Lib_L_27F-027  1020.0 
    200.065         nan 
     88.930         nan 
      3.093         nan 
 
 BW-03.9.Lib_L_27F-030  10.0 
      8.050         nan 
      5.230         nan 
      2.101         nan 
 
 BW-03.9.Lib_L_27F-030  111.0 
     56.439         nan 
     22.980         nan 
      3.023         nan 
 
 BW-03.9.Lib_L_27F-030  212.0 
     75.439         nan 
     35.800         nan 
      3.223         nan 
 
 BW-03.9.Lib_L_27F-030  313.0 
     83.578         nan 
     43.990         nan 
      3.266         nan 
 
 BW-03.9.Lib_L_27F-030  414.0 
    100.656         nan 
     51.350         nan 
      3.300         nan 
 
 BW-03.9.Lib_L_27F-030  515.0 
    116.510         nan 
     57.820         nan 
      3.318         nan 
 
 BW-03.9.Lib_L_27F-030  616.0 
    132.851         nan 
     63.580         nan 
      3.345         nan 
 
 BW-03.9.Lib_L_27F-030  717.0 
    136.433         nan 
     69.020         nan 
      3.377         nan 
 
 BW-03.9.Lib_L_27F-030  818.0 
    143.582         nan 
     74.050         nan 
      3.390         nan 
 
 BW-03.9.Lib_L_27F-030  919.0 
    150.550         nan 
     78.640         nan 
      3.396         nan 
 
 BW-03.9.Lib_L_27F-030  1020.0 
    152.723         nan 
     82.910         nan 
      3.408         nan 
 
 BW-04.10.Lib_L_27F-041  10.0 
      9.718         nan 
      5.080         nan 
      1.899         nan 
 
 BW-04.10.Lib_L_27F-041  111.0 
     77.801         nan 
     25.970         nan 
      2.882         nan 
 
 BW-04.10.Lib_L_27F-041  212.0 
    110.192         nan 
     40.780         nan 
      3.075         nan 
 
 BW-04.10.Lib_L_27F-041  313.0 
    127.549         nan 
     52.330         nan 
      3.125         nan 
 
 BW-04.10.Lib_L_27F-041  414.0 
    145.060         nan 
     63.030         nan 
      3.202         nan 
 
 BW-04.10.Lib_L_27F-041  515.0 
    158.675         nan 
     72.810         nan 
      3.242         nan 
 
 BW-04.10.Lib_L_27F-041  616.0 
    172.471         nan 
     81.010         nan 
      3.264         nan 
 
 BW-04.10.Lib_L_27F-041  717.0 
    180.183         nan 
     88.820         nan 
      3.290         nan 
 
 BW-04.10.Lib_L_27F-041  818.0 
    187.330         nan 
     95.820         nan 
      3.306         nan 
 
 BW-04.10.Lib_L_27F-041  919.0 
    194.999         nan 
    102.950         nan 
      3.329         nan 
 
 BW-04.10.Lib_L_27F-041  1020.0 
    205.074         nan 
    108.830         nan 
      3.338         nan 
 
 BW-04.4.Lib_L_27F-035  10.0 
      5.810         nan 
      3.820         nan 
      1.425         nan 
 
 BW-04.4.Lib_L_27F-035  111.0 
     39.924         nan 
     18.090         nan 
      2.080         nan 
 
 BW-04.4.Lib_L_27F-035  212.0 
     59.661         nan 
     27.510         nan 
      2.213         nan 
 
 BW-04.4.Lib_L_27F-035  313.0 
     73.312         nan 
     33.950         nan 
      2.261         nan 
 
 BW-04.4.Lib_L_27F-035  414.0 
     94.949         nan 
     39.990         nan 
      2.295         nan 
 
 BW-04.4.Lib_L_27F-035  515.0 
    102.657         nan 
     44.870         nan 
      2.318         nan 
 
 BW-04.4.Lib_L_27F-035  616.0 
    105.447         nan 
     49.750         nan 
      2.337         nan 
 
 BW-04.4.Lib_L_27F-035  717.0 
    109.861         nan 
     53.860         nan 
      2.340         nan 
 
 BW-04.4.Lib_L_27F-035  818.0 
    114.001         nan 
     58.380         nan 
      2.355         nan 
 
 BW-04.4.Lib_L_27F-035  919.0 
    117.204         nan 
     62.000         nan 
      2.362         nan 
 
 BW-04.4.Lib_L_27F-035  1020.0 
    123.416         nan 
     65.960         nan 
      2.373         nan 
 
 BW-04.5.Lib_L_27F-036  10.0 
      5.570         nan 
      4.080         nan 
      1.671         nan 
 
 BW-04.5.Lib_L_27F-036  111.0 
     34.220         nan 
     15.200         nan 
      2.208         nan 
 
 BW-04.5.Lib_L_27F-036  212.0 
     53.576         nan 
     23.580         nan 
      2.354         nan 
 
 BW-04.5.Lib_L_27F-036  313.0 
     61.141         nan 
     28.450         nan 
      2.355         nan 
 
 BW-04.5.Lib_L_27F-036  414.0 
     71.864         nan 
     34.580         nan 
      2.402         nan 
 
 BW-04.5.Lib_L_27F-036  515.0 
     78.584         nan 
     39.420         nan 
      2.417         nan 
 
 BW-04.5.Lib_L_27F-036  616.0 
     78.673         nan 
     43.780         nan 
      2.437         nan 
 
 BW-04.5.Lib_L_27F-036  717.0 
     80.728         nan 
     47.170         nan 
      2.440         nan 
 
 BW-04.5.Lib_L_27F-036  818.0 
     86.383         nan 
     50.840         nan 
      2.457         nan 
 
 BW-04.5.Lib_L_27F-036  919.0 
     86.392         nan 
     53.880         nan 
      2.463         nan 
 
 BW-04.5.Lib_L_27F-036  1020.0 
     88.325         nan 
     56.950         nan 
      2.473         nan 
 
 BW-04.6.Lib_L_27F-037  10.0 
     12.245         nan 
      6.250         nan 
      2.388         nan 
 
 BW-04.6.Lib_L_27F-037  111.0 
    110.047         nan 
     34.790         nan 
      3.668         nan 
 
 BW-04.6.Lib_L_27F-037  212.0 
    139.497         nan 
     55.290         nan 
      3.920         nan 
 
 BW-04.6.Lib_L_27F-037  313.0 
    161.900         nan 
     71.960         nan 
      4.045         nan 
 
 BW-04.6.Lib_L_27F-037  414.0 
    176.660         nan 
     84.690         nan 
      4.092         nan 
 
 BW-04.6.Lib_L_27F-037  515.0 
    197.600         nan 
     97.130         nan 
      4.157         nan 
 
 BW-04.6.Lib_L_27F-037  616.0 
    214.821         nan 
    108.060         nan 
      4.185         nan 
 
 BW-04.6.Lib_L_27F-037  717.0 
    231.937         nan 
    118.010         nan 
      4.220         nan 
 
 BW-04.6.Lib_L_27F-037  818.0 
    232.853         nan 
    126.820         nan 
      4.248         nan 
 
 BW-04.6.Lib_L_27F-037  919.0 
    245.406         nan 
    135.130         nan 
      4.268         nan 
 
 BW-04.6.Lib_L_27F-037  1020.0 
    252.232         nan 
    142.830         nan 
      4.283         nan 
 
 BW-04.7.Lib_L_27F-038  10.0 
      6.345         nan 
      4.010         nan 
      1.483         nan 
 
 BW-04.7.Lib_L_27F-038  111.0 
     29.746         nan 
     16.160         nan 
      2.160         nan 
 
 BW-04.7.Lib_L_27F-038  212.0 
     44.191         nan 
     22.160         nan 
      2.237         nan 
 
 BW-04.7.Lib_L_27F-038  313.0 
     55.093         nan 
     26.730         nan 
      2.279         nan 
 
 BW-04.7.Lib_L_27F-038  414.0 
     62.980         nan 
     30.860         nan 
      2.294         nan 
 
 BW-04.7.Lib_L_27F-038  515.0 
     70.077         nan 
     34.820         nan 
      2.325         nan 
 
 BW-04.7.Lib_L_27F-038  616.0 
     80.735         nan 
     38.650         nan 
      2.340         nan 
 
 BW-04.7.Lib_L_27F-038  717.0 
     87.611         nan 
     41.460         nan 
      2.342         nan 
 
 BW-04.7.Lib_L_27F-038  818.0 
     88.859         nan 
     44.430         nan 
      2.353         nan 
 
 BW-04.7.Lib_L_27F-038  919.0 
     87.912         nan 
     47.280         nan 
      2.362         nan 
 
 BW-04.7.Lib_L_27F-038  1020.0 
     91.072         nan 
     49.970         nan 
      2.368         nan 
 
 BW-04.9.Lib_L_27F-040  10.0 
      7.863         nan 
      4.430         nan 
      1.670         nan 
 
 BW-04.9.Lib_L_27F-040  111.0 
     57.776         nan 
     23.600         nan 
      2.653         nan 
 
 BW-04.9.Lib_L_27F-040  212.0 
     76.485         nan 
     36.060         nan 
      2.778         nan 
 
 BW-04.9.Lib_L_27F-040  313.0 
     86.717         nan 
     45.550         nan 
      2.887         nan 
 
 BW-04.9.Lib_L_27F-040  414.0 
    106.526         nan 
     54.200         nan 
      2.921         nan 
 
 BW-04.9.Lib_L_27F-040  515.0 
    119.204         nan 
     60.220         nan 
      2.949         nan 
 
 BW-04.9.Lib_L_27F-040  616.0 
    135.464         nan 
     66.670         nan 
      2.971         nan 
 
 BW-04.9.Lib_L_27F-040  717.0 
    142.388         nan 
     71.850         nan 
      2.971         nan 
 
 BW-04.9.Lib_L_27F-040  818.0 
    151.896         nan 
     77.000         nan 
      2.993         nan 
 
 BW-04.9.Lib_L_27F-040  919.0 
    161.466         nan 
     82.010         nan 
      3.007         nan 
 
 BW-04.9.Lib_L_27F-040  1020.0 
    173.352         nan 
     86.940         nan 
      3.019         nan 
 
 BW-05.1.Lib_L_27F-042  10.0 
      5.583         nan 
      3.460         nan 
      1.154         nan 
 
 BW-05.1.Lib_L_27F-042  111.0 
     39.535         nan 
     17.870         nan 
      1.808         nan 
 
 BW-05.1.Lib_L_27F-042  212.0 
     52.523         nan 
     25.890         nan 
      1.908         nan 
 
 BW-05.1.Lib_L_27F-042  313.0 
     65.529         nan 
     32.670         nan 
      1.940         nan 
 
 BW-05.1.Lib_L_27F-042  414.0 
     82.514         nan 
     39.060         nan 
      2.008         nan 
 
 BW-05.1.Lib_L_27F-042  515.0 
     88.046         nan 
     43.790         nan 
      2.025         nan 
 
 BW-05.1.Lib_L_27F-042  616.0 
     91.828         nan 
     48.330         nan 
      2.038         nan 
 
 BW-05.1.Lib_L_27F-042  717.0 
     99.071         nan 
     52.280         nan 
      2.046         nan 
 
 BW-05.1.Lib_L_27F-042  818.0 
    104.453         nan 
     56.010         nan 
      2.055         nan 
 
 BW-05.1.Lib_L_27F-042  919.0 
    113.388         nan 
     59.740         nan 
      2.066         nan 
 
 BW-05.1.Lib_L_27F-042  1020.0 
    121.633         nan 
     62.930         nan 
      2.070         nan 
 
 BW-05.9.Lib_L_27F-051  10.0 
     13.290         nan 
      6.170         nan 
      2.295         nan 
 
 BW-05.9.Lib_L_27F-051  111.0 
     88.914         nan 
     33.480         nan 
      3.602         nan 
 
 BW-05.9.Lib_L_27F-051  212.0 
    101.506         nan 
     49.870         nan 
      3.782         nan 
 
 BW-05.9.Lib_L_27F-051  313.0 
    129.633         nan 
     63.160         nan 
      3.891         nan 
 
 BW-05.9.Lib_L_27F-051  414.0 
    141.900         nan 
     73.200         nan 
      3.952         nan 
 
 BW-05.9.Lib_L_27F-051  515.0 
    159.987         nan 
     82.940         nan 
      3.986         nan 
 
 BW-05.9.Lib_L_27F-051  616.0 
    174.034         nan 
     90.140         nan 
      4.009         nan 
 
 BW-05.9.Lib_L_27F-051  717.0 
    196.661         nan 
     99.470         nan 
      4.057         nan 
 
 BW-05.9.Lib_L_27F-051  818.0 
    210.552         nan 
    105.480         nan 
      4.060         nan 
 
 BW-05.9.Lib_L_27F-051  919.0 
    221.127         nan 
    112.670         nan 
      4.086         nan 
 
 BW-05.9.Lib_L_27F-051  1020.0 
    229.670         nan 
    118.810         nan 
      4.098         nan 
 
 BW-06.4.Lib_L_27F-003  10.0 
     15.275         nan 
      6.370         nan 
      2.325         nan 
 
 BW-06.4.Lib_L_27F-003  111.0 
    134.923         nan 
     40.130         nan 
      3.859         nan 
 
 BW-06.4.Lib_L_27F-003  212.0 
    188.623         nan 
     62.340         nan 
      4.095         nan 
 
 BW-06.4.Lib_L_27F-003  313.0 
    200.641         nan 
     80.130         nan 
      4.197         nan 
 
 BW-06.4.Lib_L_27F-003  414.0 
    219.984         nan 
     97.940         nan 
      4.324         nan 
 
 BW-06.4.Lib_L_27F-003  515.0 
    237.006         nan 
    112.120         nan 
      4.362         nan 
 
 BW-06.4.Lib_L_27F-003  616.0 
    245.996         nan 
    124.440         nan 
      4.408         nan 
 
 BW-06.4.Lib_L_27F-003  717.0 
    253.468         nan 
    137.240         nan 
      4.450         nan 
 
 BW-06.4.Lib_L_27F-003  818.0 
    266.583         nan 
    146.820         nan 
      4.459         nan 
 
 BW-06.4.Lib_L_27F-003  919.0 
    270.596         nan 
    157.470         nan 
      4.495         nan 
 
 BW-06.4.Lib_L_27F-003  1020.0 
    278.792         nan 
    166.800         nan 
      4.517         nan 
 
 BW-06.6.Lib_L_27F-005  10.0 
     13.500         nan 
      6.380         nan 
      2.376         nan 
 
 BW-06.6.Lib_L_27F-005  111.0 
    113.583         nan 
     35.900         nan 
      3.832         nan 
 
 BW-06.6.Lib_L_27F-005  212.0 
    151.590         nan 
     54.640         nan 
      4.032         nan 
 
 BW-06.6.Lib_L_27F-005  313.0 
    195.678         nan 
     72.730         nan 
      4.176         nan 
 
 BW-06.6.Lib_L_27F-005  414.0 
    226.829         nan 
     87.780         nan 
      4.258         nan 
 
 BW-06.6.Lib_L_27F-005  515.0 
    248.135         nan 
    102.090         nan 
      4.326         nan 
 
 BW-06.6.Lib_L_27F-005  616.0 
    253.505         nan 
    113.340         nan 
      4.352         nan 
 
 BW-06.6.Lib_L_27F-005  717.0 
    260.694         nan 
    124.080         nan 
      4.373         nan 
 
 BW-06.6.Lib_L_27F-005  818.0 
    280.385         nan 
    135.170         nan 
      4.409         nan 
 
 BW-06.6.Lib_L_27F-005  919.0 
    278.097         nan 
    144.800         nan 
      4.433         nan 
 
 BW-06.6.Lib_L_27F-005  1020.0 
    280.351         nan 
    153.820         nan 
      4.450         nan 
 
 BW-06.7.Lib_L_27F-006  10.0 
     14.378         nan 
      6.570         nan 
      2.448         nan 
 
 BW-06.7.Lib_L_27F-006  111.0 
    107.749         nan 
     35.320         nan 
      3.868         nan 
 
 BW-06.7.Lib_L_27F-006  212.0 
    152.383         nan 
     55.540         nan 
      4.125         nan 
 
 BW-06.7.Lib_L_27F-006  313.0 
    189.175         nan 
     70.740         nan 
      4.196         nan 
 
 BW-06.7.Lib_L_27F-006  414.0 
    213.498         nan 
     85.970         nan 
      4.287         nan 
 
 BW-06.7.Lib_L_27F-006  515.0 
    214.121         nan 
     99.370         nan 
      4.357         nan 
 
 BW-06.7.Lib_L_27F-006  616.0 
    227.671         nan 
    110.260         nan 
      4.381         nan 
 
 BW-06.7.Lib_L_27F-006  717.0 
    229.488         nan 
    121.280         nan 
      4.420         nan 
 
 BW-06.7.Lib_L_27F-006  818.0 
    236.445         nan 
    130.410         nan 
      4.437         nan 
 
 BW-06.7.Lib_L_27F-006  919.0 
    235.584         nan 
    139.610         nan 
      4.461         nan 
 
 BW-06.7.Lib_L_27F-006  1020.0 
    241.387         nan 
    147.840         nan 
      4.482         nan 
 
 BW-06.9.Lib_L_27F-008  10.0 
     13.723         nan 
      5.870         nan 
      2.147         nan 
 
 BW-06.9.Lib_L_27F-008  111.0 
     90.891         nan 
     33.470         nan 
      3.388         nan 
 
 BW-06.9.Lib_L_27F-008  212.0 
    140.029         nan 
     51.450         nan 
      3.581         nan 
 
 BW-06.9.Lib_L_27F-008  313.0 
    170.023         nan 
     65.400         nan 
      3.672         nan 
 
 BW-06.9.Lib_L_27F-008  414.0 
    187.999         nan 
     78.780         nan 
      3.749         nan 
 
 BW-06.9.Lib_L_27F-008  515.0 
    211.538         nan 
     90.470         nan 
      3.805         nan 
 
 BW-06.9.Lib_L_27F-008  616.0 
    227.754         nan 
    100.900         nan 
      3.852         nan 
 
 BW-06.9.Lib_L_27F-008  717.0 
    247.142         nan 
    110.650         nan 
      3.880         nan 
 
 BW-06.9.Lib_L_27F-008  818.0 
    270.222         nan 
    119.610         nan 
      3.900         nan 
 
 BW-06.9.Lib_L_27F-008  919.0 
    282.354         nan 
    129.070         nan 
      3.931         nan 
 
 BW-06.9.Lib_L_27F-008  1020.0 
    298.684         nan 
    136.850         nan 
      3.941         nan 
 
 BW-07.3.Lib_L_27F-013  10.0 
      5.467         nan 
      3.790         nan 
      1.450         nan 
 
 BW-07.3.Lib_L_27F-013  111.0 
     31.941         nan 
     15.530         nan 
      2.069         nan 
 
 BW-07.3.Lib_L_27F-013  212.0 
     42.161         nan 
     21.830         nan 
      2.115         nan 
 
 BW-07.3.Lib_L_27F-013  313.0 
     52.391         nan 
     27.610         nan 
      2.161         nan 
 
 BW-07.3.Lib_L_27F-013  414.0 
     59.756         nan 
     31.600         nan 
      2.161         nan 
 
 BW-07.3.Lib_L_27F-013  515.0 
     60.125         nan 
     35.440         nan 
      2.195         nan 
 
 BW-07.3.Lib_L_27F-013  616.0 
     63.686         nan 
     38.760         nan 
      2.205         nan 
 
 BW-07.3.Lib_L_27F-013  717.0 
     67.033         nan 
     41.790         nan 
      2.225         nan 
 
 BW-07.3.Lib_L_27F-013  818.0 
     67.816         nan 
     44.600         nan 
      2.235         nan 
 
 BW-07.3.Lib_L_27F-013  919.0 
     68.491         nan 
     46.650         nan 
      2.240         nan 
 
 BW-07.3.Lib_L_27F-013  1020.0 
     69.985         nan 
     48.980         nan 
      2.244         nan 
 
 BW-07.4.Lib_L_27F-014  10.0 
      8.358         nan 
      4.910         nan 
      1.897         nan 
 
 BW-07.4.Lib_L_27F-014  111.0 
     55.237         nan 
     22.330         nan 
      2.740         nan 
 
 BW-07.4.Lib_L_27F-014  212.0 
     68.257         nan 
     33.670         nan 
      2.888         nan 
 
 BW-07.4.Lib_L_27F-014  313.0 
     75.808         nan 
     42.020         nan 
      2.943         nan 
 
 BW-07.4.Lib_L_27F-014  414.0 
     85.018         nan 
     48.580         nan 
      2.973         nan 
 
 BW-07.4.Lib_L_27F-014  515.0 
     94.497         nan 
     54.580         nan 
      2.993         nan 
 
 BW-07.4.Lib_L_27F-014  616.0 
    106.808         nan 
     60.420         nan 
      3.030         nan 
 
 BW-07.4.Lib_L_27F-014  717.0 
    114.999         nan 
     65.130         nan 
      3.045         nan 
 
 BW-07.4.Lib_L_27F-014  818.0 
    119.053         nan 
     68.960         nan 
      3.054         nan 
 
 BW-07.4.Lib_L_27F-014  919.0 
    131.723         nan 
     73.180         nan 
      3.064         nan 
 
 BW-07.4.Lib_L_27F-014  1020.0 
    142.857         nan 
     76.930         nan 
      3.079         nan 
 
 BW-07.5.Lib_L_27F-015  10.0 
      6.550         nan 
      4.380         nan 
      1.769         nan 
 
 BW-07.5.Lib_L_27F-015  111.0 
     46.050         nan 
     19.620         nan 
      2.587         nan 
 
 BW-07.5.Lib_L_27F-015  212.0 
     70.754         nan 
     28.300         nan 
      2.666         nan 
 
 BW-07.5.Lib_L_27F-015  313.0 
     87.388         nan 
     35.540         nan 
      2.733         nan 
 
 BW-07.5.Lib_L_27F-015  414.0 
     99.452         nan 
     42.440         nan 
      2.774         nan 
 
 BW-07.5.Lib_L_27F-015  515.0 
    107.519         nan 
     49.130         nan 
      2.804         nan 
 
 BW-07.5.Lib_L_27F-015  616.0 
    111.082         nan 
     54.150         nan 
      2.812         nan 
 
 BW-07.5.Lib_L_27F-015  717.0 
    107.190         nan 
     58.810         nan 
      2.825         nan 
 
 BW-07.5.Lib_L_27F-015  818.0 
    103.966         nan 
     63.060         nan 
      2.841         nan 
 
 BW-07.5.Lib_L_27F-015  919.0 
    101.009         nan 
     67.090         nan 
      2.847         nan 
 
 BW-07.5.Lib_L_27F-015  1020.0 
    100.734         nan 
     70.940         nan 
      2.856         nan 
 
 BW-07.8.Lib_L_27F-018  10.0 
      4.633         nan 
      3.630         nan 
      1.513         nan 
 
 BW-07.8.Lib_L_27F-018  111.0 
     29.357         nan 
     13.590         nan 
      1.972         nan 
 
 BW-07.8.Lib_L_27F-018  212.0 
     46.711         nan 
     19.740         nan 
      2.051         nan 
 
 BW-07.8.Lib_L_27F-018  313.0 
     53.536         nan 
     24.860         nan 
      2.096         nan 
 
 BW-07.8.Lib_L_27F-018  414.0 
     54.367         nan 
     28.820         nan 
      2.104         nan 
 
 BW-07.8.Lib_L_27F-018  515.0 
     56.749         nan 
     32.550         nan 
      2.121         nan 
 
 BW-07.8.Lib_L_27F-018  616.0 
     59.073         nan 
     35.470         nan 
      2.131         nan 
 
 BW-07.8.Lib_L_27F-018  717.0 
     66.363         nan 
     38.760         nan 
      2.148         nan 
 
 BW-07.8.Lib_L_27F-018  818.0 
     68.393         nan 
     41.330         nan 
      2.155         nan 
 
 BW-07.8.Lib_L_27F-018  919.0 
     73.484         nan 
     43.870         nan 
      2.164         nan 
 
 BW-07.8.Lib_L_27F-018  1020.0 
     78.953         nan 
     45.970         nan 
      2.167         nan 
 
 BW-09.3.Lib_L_27F-033  10.0 
     13.557         nan 
      6.380         nan 
      2.402         nan 
 
 BW-09.3.Lib_L_27F-033  111.0 
    107.452         nan 
     34.990         nan 
      3.780         nan 
 
 BW-09.3.Lib_L_27F-033  212.0 
    138.971         nan 
     54.000         nan 
      3.968         nan 
 
 BW-09.3.Lib_L_27F-033  313.0 
    171.448         nan 
     71.360         nan 
      4.108         nan 
 
 BW-09.3.Lib_L_27F-033  414.0 
    183.803         nan 
     83.630         nan 
      4.162         nan 
 
 BW-09.3.Lib_L_27F-033  515.0 
    202.388         nan 
     95.410         nan 
      4.216         nan 
 
 BW-09.3.Lib_L_27F-033  616.0 
    208.528         nan 
    105.410         nan 
      4.241         nan 
 
 BW-09.3.Lib_L_27F-033  717.0 
    223.689         nan 
    115.780         nan 
      4.279         nan 
 
 BW-09.3.Lib_L_27F-033  818.0 
    235.559         nan 
    124.990         nan 
      4.313         nan 
 
 BW-09.3.Lib_L_27F-033  919.0 
    251.040         nan 
    133.530         nan 
      4.324         nan 
 
 BW-09.3.Lib_L_27F-033  1020.0 
    254.594         nan 
    140.830         nan 
      4.343         nan 
 
 BW-09.4.Lib_L_27F-034  10.0 
     11.012         nan 
      6.120         nan 
      2.367         nan 
 
 BW-09.4.Lib_L_27F-034  111.0 
     97.595         nan 
     28.710         nan 
      3.463         nan 
 
 BW-09.4.Lib_L_27F-034  212.0 
    122.103         nan 
     44.880         nan 
      3.644         nan 
 
 BW-09.4.Lib_L_27F-034  313.0 
    142.655         nan 
     59.010         nan 
      3.747         nan 
 
 BW-09.4.Lib_L_27F-034  414.0 
    157.261         nan 
     70.520         nan 
      3.802         nan 
 
 BW-09.4.Lib_L_27F-034  515.0 
    166.979         nan 
     80.930         nan 
      3.838         nan 
 
 BW-09.4.Lib_L_27F-034  616.0 
    175.510         nan 
     90.330         nan 
      3.875         nan 
 
 BW-09.4.Lib_L_27F-034  717.0 
    183.228         nan 
     99.310         nan 
      3.907         nan 
 
 BW-09.4.Lib_L_27F-034  818.0 
    191.016         nan 
    107.860         nan 
      3.938         nan 
 
 BW-09.4.Lib_L_27F-034  919.0 
    188.544         nan 
    114.160         nan 
      3.944         nan 
 
 BW-09.4.Lib_L_27F-034  1020.0 
    190.769         nan 
    120.830         nan 
      3.962         nan 
 
 BW-09.9.Lib_L_27F-039  10.0 
      5.783         nan 
      4.140         nan 
      1.692         nan 
 
 BW-09.9.Lib_L_27F-039  111.0 
     54.538         nan 
     18.250         nan 
      2.359         nan 
 
 BW-09.9.Lib_L_27F-039  212.0 
     70.568         nan 
     27.490         nan 
      2.454         nan 
 
 BW-09.9.Lib_L_27F-039  313.0 
     84.073         nan 
     36.270         nan 
      2.513         nan 
 
 BW-09.9.Lib_L_27F-039  414.0 
     85.183         nan 
     43.350         nan 
      2.565         nan 
 
 BW-09.9.Lib_L_27F-039  515.0 
     89.642         nan 
     49.660         nan 
      2.590         nan 
 
 BW-09.9.Lib_L_27F-039  616.0 
     86.395         nan 
     54.560         nan 
      2.608         nan 
 
 BW-09.9.Lib_L_27F-039  717.0 
     90.032         nan 
     59.300         nan 
      2.631         nan 
 
 BW-09.9.Lib_L_27F-039  818.0 
     91.484         nan 
     63.440         nan 
      2.638         nan 
 
 BW-09.9.Lib_L_27F-039  919.0 
     90.262         nan 
     66.900         nan 
      2.647         nan 
 
 BW-09.9.Lib_L_27F-039  1020.0 
     90.407         nan 
     69.960         nan 
      2.655         nan 
 
 BW-10.10.Lib_L_27F-051  10.0 
      5.165         nan 
      3.400         nan 
      1.176         nan 
 
 BW-10.10.Lib_L_27F-051  111.0 
     39.781         nan 
     15.170         nan 
      1.737         nan 
 
 BW-10.10.Lib_L_27F-051  212.0 
     55.227         nan 
     23.300         nan 
      1.817         nan 
 
 BW-10.10.Lib_L_27F-051  313.0 
     61.202         nan 
     30.180         nan 
      1.885         nan 
 
 BW-10.10.Lib_L_27F-051  414.0 
     63.375         nan 
     36.000         nan 
      1.937         nan 
 
 BW-10.10.Lib_L_27F-051  515.0 
     65.833         nan 
     40.200         nan 
      1.943         nan 
 
 BW-10.10.Lib_L_27F-051  616.0 
     65.854         nan 
     43.920         nan 
      1.956         nan 
 
 BW-10.10.Lib_L_27F-051  717.0 
     68.632         nan 
     47.240         nan 
      1.967         nan 
 
 BW-10.10.Lib_L_27F-051  818.0 
     67.865         nan 
     50.160         nan 
      1.976         nan 
 
 BW-10.10.Lib_L_27F-051  919.0 
     66.345         nan 
     52.860         nan 
      1.982         nan 
 
 BW-10.10.Lib_L_27F-051  1020.0 
     65.629         nan 
     54.980         nan 
      1.988         nan 
 
  Subject  Seqs/Sample 
 chao1 Ave.  chao1 Err. 
 observed_species Ave.  observed_species Err. 
 shannon Ave.  shannon Err. 
 
 
 01  10.0 
      9.849       6.700 
      4.990       2.431 
      1.778       1.026 
 
 01  111.0 
     65.370      49.666 
     23.917      14.225 
      2.715       1.565 
 
 01  212.0 
     92.234      66.909 
     36.220      22.783 
      2.852       1.686 
 
 01  313.0 
    103.905      75.305 
     46.580      29.908 
      2.936       1.715 
 
 01  414.0 
    118.952      86.889 
     55.380      35.918 
      2.984       1.734 
 
 01  515.0 
    122.273      88.197 
     62.570      41.298 
      3.008       1.758 
 
 01  616.0 
    129.098      95.210 
     69.423      46.259 
      3.040       1.774 
 
 01  717.0 
    138.314      99.523 
     76.100      51.287 
      3.067       1.789 
 
 01  818.0 
    141.084     104.067 
     81.673      55.308 
      3.079       1.799 
 
 01  919.0 
    145.105     107.211 
     86.417      58.888 
      3.089       1.804 
 
 01  1020.0 
    151.991     112.828 
     91.233      62.601 
      3.099       1.813 
 
 02  10.0 
      5.271       2.183 
      3.328       0.955 
      1.108       0.458 
 
 02  111.0 
     37.045      17.074 
     15.730       5.503 
      1.648       0.652 
 
 02  212.0 
     52.139      25.709 
     23.765       9.057 
      1.759       0.700 
 
 02  313.0 
     64.319      29.065 
     29.893      11.517 
      1.792       0.704 
 
 02  414.0 
     72.458      29.787 
     35.160      13.912 
      1.826       0.712 
 
 02  515.0 
     78.591      29.592 
     39.743      15.821 
      1.844       0.722 
 
 02  616.0 
     86.186      33.345 
     43.735      17.588 
      1.855       0.727 
 
 02  717.0 
     91.281      34.588 
     47.567      19.184 
      1.867       0.728 
 
 02  818.0 
     94.791      36.604 
     51.223      20.683 
      1.879       0.733 
 
 02  919.0 
     98.138      36.159 
     54.393      21.900 
      1.886       0.738 
 
 02  1020.0 
     99.465      37.824 
     57.455      23.128 
      1.893       0.741 
 
 03  10.0 
      7.260       2.540 
      4.254       1.224 
      1.547       0.599 
 
 03  111.0 
     43.869      12.992 
     19.640       5.691 
      2.297       0.851 
 
 03  212.0 
     64.066      16.974 
     29.364       8.137 
      2.432       0.892 
 
 03  313.0 
     75.474      20.286 
     36.536       9.836 
      2.479       0.898 
 
 03  414.0 
     87.444      23.681 
     42.962      11.298 
      2.517       0.902 
 
 03  515.0 
     99.645      26.335 
     48.570      12.567 
      2.536       0.903 
 
 03  616.0 
    107.583      30.235 
     53.238      13.985 
      2.556       0.912 
 
 03  717.0 
    115.308      34.097 
     58.072      15.212 
      2.576       0.918 
 
 03  818.0 
    124.261      36.267 
     62.444      16.287 
      2.586       0.921 
 
 03  919.0 
    130.592      41.752 
     66.480      17.455 
      2.596       0.924 
 
 03  1020.0 
    137.356      46.354 
     70.134      18.437 
      2.604       0.926 
 
 04  10.0 
      7.925       2.394 
      4.612       0.837 
      1.756       0.321 
 
 04  111.0 
     58.252      28.258 
     22.302       6.795 
      2.609       0.554 
 
 04  212.0 
     80.600      33.803 
     34.230      11.504 
      2.763       0.603 
 
 04  313.0 
     94.285      38.319 
     43.162      15.761 
      2.825       0.635 
 
 04  414.0 
    109.673      39.950 
     51.225      18.646 
      2.868       0.644 
 
 04  515.0 
    121.133      44.756 
     58.212      21.641 
      2.901       0.659 
 
 04  616.0 
    131.269      49.469 
     64.653      24.117 
      2.922       0.662 
 
 04  717.0 
    138.785      53.588 
     70.195      26.641 
      2.934       0.674 
 
 04  818.0 
    143.554      53.376 
     75.548      28.622 
      2.952       0.679 
 
 04  919.0 
    148.897      58.063 
     80.542      30.673 
      2.965       0.685 
 
 04  1020.0 
    155.578      60.313 
     85.247      32.383 
      2.976       0.686 
 
 05  10.0 
      9.437       3.853 
      4.815       1.355 
      1.724       0.571 
 
 05  111.0 
     64.225      24.689 
     25.675       7.805 
      2.705       0.897 
 
 05  212.0 
     77.014      24.491 
     37.880      11.990 
      2.845       0.937 
 
 05  313.0 
     97.581      32.052 
     47.915      15.245 
      2.916       0.976 
 
 05  414.0 
    112.207      29.693 
     56.130      17.070 
      2.980       0.972 
 
 05  515.0 
    124.017      35.971 
     63.365      19.575 
      3.006       0.981 
 
 05  616.0 
    132.931      41.103 
     69.235      20.905 
      3.023       0.985 
 
 05  717.0 
    147.866      48.795 
     75.875      23.595 
      3.051       1.006 
 
 05  818.0 
    157.503      53.049 
     80.745      24.735 
      3.057       1.003 
 
 05  919.0 
    167.258      53.870 
     86.205      26.465 
      3.076       1.010 
 
 05  1020.0 
    175.652      54.019 
     90.870      27.940 
      3.084       1.014 
 
 06  10.0 
     14.219       0.690 
      6.298       0.259 
      2.324       0.111 
 
 06  111.0 
    111.787      15.744 
     36.205       2.437 
      3.737       0.202 
 
 06  212.0 
    158.156      18.257 
     55.992       3.967 
      3.958       0.220 
 
 06  313.0 
    188.879      11.621 
     72.250       5.280 
      4.060       0.224 
 
 06  414.0 
    212.077      14.679 
     87.618       6.845 
      4.154       0.235 
 
 06  515.0 
    227.700      15.409 
    101.013       7.720 
      4.213       0.236 
 
 06  616.0 
    238.732      11.334 
    112.235       8.405 
      4.248       0.230 
 
 06  717.0 
    247.698      11.555 
    123.312       9.474 
      4.281       0.233 
 
 06  818.0 
    263.409      16.369 
    133.002       9.769 
      4.301       0.232 
 
 06  919.0 
    266.658      18.427 
    142.738      10.221 
      4.330       0.231 
 
 06  1020.0 
    274.804      20.819 
    151.327      10.809 
      4.348       0.236 
 
 07  10.0 
      6.252       1.393 
      4.178       0.507 
      1.657       0.183 
 
 07  111.0 
     40.646      10.551 
     17.767       3.417 
      2.342       0.328 
 
 07  212.0 
     56.971      12.668 
     25.885       5.492 
      2.430       0.356 
 
 07  313.0 
     67.281      14.897 
     32.508       6.748 
      2.483       0.363 
 
 07  414.0 
     74.648      18.411 
     37.860       8.013 
      2.503       0.378 
 
 07  515.0 
     79.723      21.810 
     42.925       9.192 
      2.528       0.377 
 
 07  616.0 
     85.162      23.886 
     47.200      10.391 
      2.544       0.385 
 
 07  717.0 
     88.897      22.370 
     51.123      11.127 
      2.561       0.383 
 
 07  818.0 
     89.807      22.349 
     54.487      11.767 
      2.571       0.385 
 
 07  919.0 
     93.677      25.216 
     57.697      12.661 
      2.579       0.385 
 
 07  1020.0 
     98.132      28.139 
     60.705      13.441 
      2.587       0.390 
 
 09  10.0 
     10.117       3.236 
      5.547       1.000 
      2.154       0.327 
 
 09  111.0 
     86.528      22.976 
     27.317       6.905 
      3.200       0.609 
 
 09  212.0 
    110.547      29.096 
     42.123      10.997 
      3.355       0.651 
 
 09  313.0 
    132.725      36.355 
     55.547      14.533 
      3.456       0.683 
 
 09  414.0 
    142.082      41.667 
     65.833      16.775 
      3.510       0.684 
 
 09  515.0 
    153.003      47.077 
     75.333      19.092 
      3.548       0.695 
 
 09  616.0 
    156.811      51.584 
     83.433      21.325 
      3.575       0.700 
 
 09  717.0 
    165.650      55.963 
     91.463      23.716 
      3.606       0.706 
 
 09  818.0 
    172.687      60.229 
     98.763      25.938 
      3.630       0.718 
 
 09  919.0 
    176.615      66.177 
    104.863      27.985 
      3.638       0.718 
 
 09  1020.0 
    178.590      67.580 
    110.540      29.833 
      3.653       0.723 
 
 10  10.0 
      5.165         nan 
      3.400         nan 
      1.176         nan 
 
 10  111.0 
     39.781         nan 
     15.170         nan 
      1.737         nan 
 
 10  212.0 
     55.227         nan 
     23.300         nan 
      1.817         nan 
 
 10  313.0 
     61.202         nan 
     30.180         nan 
      1.885         nan 
 
 10  414.0 
     63.375         nan 
     36.000         nan 
      1.937         nan 
 
 10  515.0 
     65.833         nan 
     40.200         nan 
      1.943         nan 
 
 10  616.0 
     65.854         nan 
     43.920         nan 
      1.956         nan 
 
 10  717.0 
     68.632         nan 
     47.240         nan 
      1.967         nan 
 
 10  818.0 
     67.865         nan 
     50.160         nan 
      1.976         nan 
 
 10  919.0 
     66.345         nan 
     52.860         nan 
      1.982         nan 
 
 10  1020.0 
     65.629         nan 
     54.980         nan 
      1.988         nan
